# Mass spectrometry of the white adipose metabolome in a hibernating mammal reveals seasonal changes in alternate fuels and carnitine derivatives

**DOI:** 10.3389/fphys.2023.1214087

**Published:** 2023-06-28

**Authors:** Frazer I. Heinis, Sophie Alvarez, Matthew T. Andrews

**Affiliations:** ^1^ School of Natural Resources, University of Nebraska-Lincoln, Lincoln, NE, United States; ^2^ Proteomics and Metabolomics Facility, Nebraska Center for Biotechnology, University of Nebraska-Lincoln, Lincoln, NE, United States

**Keywords:** hibernation, polar metabolites, hypothermia, white adipose, metabolomics, HILIC-MS, ground squirrels

## Abstract

Mammalian hibernators undergo substantial changes in metabolic function throughout the seasonal hibernation cycle. We report here the polar metabolomic profile of white adipose tissue isolated from active and hibernating thirteen-lined ground squirrels (*Ictidomys tridecemlineatus*). Polar compounds in white adipose tissue were extracted from five groups representing different timepoints throughout the seasonal activity-torpor cycle and analyzed using hydrophilic interaction liquid chromatography-mass spectrometry in both the positive and negative ion modes. A total of 224 compounds out of 660 features detected after curation were annotated. Unsupervised clustering using principal component analysis revealed discrete clusters representing the different seasonal timepoints throughout hibernation. One-way analysis of variance and feature intensity heatmaps revealed metabolites that varied in abundance between active and torpid timepoints. Pathway analysis compared against the KEGG database demonstrated enrichment of amino acid metabolism, purine metabolism, glycerophospholipid metabolism, and coenzyme A biosynthetic pathways among our identified compounds. Numerous carnitine derivatives and a ketone that serves as an alternate fuel source, beta-hydroxybutyrate (BHB), were among molecules found to be elevated during torpor. Elevated levels of the BHB-carnitine conjugate during torpor suggests the synthesis of beta-hydroxybutyrate in white adipose mitochondria, which may contribute directly to elevated levels of circulating BHB during hibernation.

## 1 Introduction

As an adaptation to inhospitable environmental conditions, mammalian hibernators undergo seasonal heterothermy. Throughout this process, hibernating mammals enter multi-day bouts of torpor (TOR), during which their core body temperature drops to near ambient temperatures, which can be as low as −3°C for Arctic ground squirrels (Barnes, 1989). Hibernating mammals have adapted to survive severe environmental and physiological stresses that would be lethal to individuals not adapted for hibernation, such as the lack of food for up to 5–6 months, prolonged hypothermia, decreases in metabolic rate, and substantial decreases in heart and respiratory rate (reviewed in Carey et al., 2003; Geiser, 2004). The molecular and physiological adaptations that allow deep hibernators to survive such stresses are of significant biomedical interest (reviewed in Andrews, 2019).

The thirteen-lined ground squirrel (*Ictidomys tridecemlineatus*) is a deep hibernator that does not feed during hibernation and therefore relies on stored calorie reserves. White adipose tissue (WAT) serves as a crucial calorie depot throughout the hibernation season. During the active summer months, ground squirrels gain weight as they store dietary energy as lipids in WAT. WAT mass increases during late summer and early fall when hibernators are near their peak body weight (Schwartz et al., 2015). After the onset of torpor in mid to late fall, WAT mass and adipocyte size progressively decrease as stored triglycerides are mobilized (Florant et al., 2004). During torpor, ground squirrels undergo a shift in metabolism away from the utilization of circulating glucose towards the catabolism of stored fat (Bauer et al., 2001; Buck et al., 2002).

Torpor bouts are interrupted by brief periods of rewarming and activity, termed interbout arousals (IBAs), in which a hibernator warms from near-freezing hypothermia to approximately 37°C for 12–24 h before returning to torpor (Hampton et al., 2010). Although the function of IBAs has not been definitively identified, these regular periods of elevated body temperature and activity may serve several key functions including new mRNA and protein synthesis and turnover; facilitating mobilization of metabolites from tissues into circulation; allow for the conversion of toxic metabolites that accumulated during torpor; and permit chemical reactions to occur that cannot proceed at low temperature (Galster and Morrison, 1975; van Breukelen and Martin, 2001; van Breukelen and Martin, 2002; Prendergast et al., 2002; Epperson et al., 2011; D’Alessandro et al., 2017; Wiersma et al., 2018; Rice et al., 2020).

The circannual cycle of adiposity in hibernating animals, and the role of hibernator WAT in supporting the survival of animals during prolonged starvation, are potentially significant for improving human health and biomedical science. The transcriptome profile of hibernator tissues has been comprehensively studied throughout the seasonal hibernation cycle (Hampton et al., 2011; Hampton et al., 2013; Schwartz et al., 2013; Cooper et al., 2016; Luan et al., 2018). Several groups have previously investigated the metabolome of the hibernator brain (Henry et al., 2007), plasma (Epperson et al., 2011; D’Alessandro et al., 2017), erythrocyte (Gehrke et al., 2019), and liver (Serkova et al., 2007; Nelson et al., 2009) at different times throughout the circannual hibernation cycle. Understanding the ways in which different hibernator organ systems produce, utilize, release, and take up metabolites, and the ways that those processes are influenced by the hibernation cycle, are crucial for understanding hibernator physiology and using that knowledge to improve non-hibernator health, such as a hibernation-based treatment for hemorrhagic shock (Klein et al., 2010; Perez de Lara Rodriguez et al., 2017).

In this study we interrogated the white adipose polar metabolome to evaluate the contribution of this tissue to torpor-associated metabolic changes, and to determine adipose tissue-specific adaptations during hibernation. Rather than a lipidomic study that would focus on hydrophobic calorie-storage molecules such as triacylglycerols, we sought to determine the identity of polar metabolites and their possible role in fuel mobilization throughout the hibernation season. We collected abdominal white adipose tissue at different times during the seasonal hibernation cycle, performed liquid-liquid extraction of WAT samples to enrich for polar compounds, and evaluated those extracts by hydrophilic interaction chromatography-tandem mass spectrometry (HILIC-MS) in both the positive and negative ion modes.

## 2 Materials and methods

### 2.1 Animal care and handling

All animal use in this study was approved by the Institutional Animal Care and Use Committee at the University of Nebraska-Lincoln (UNL Project #1927).

Thirteen-lined ground squirrels were trapped near Lincoln, Nebraska during June, July, and August. Ground squirrels were housed at UNL veterinary facilities under the care of UNL Institutional Animal Care Program veterinary staff. Squirrels were singly housed at 21°C–22°C and 12h–12h light-dark cycle with chow (Envigo #2016) and sunflower seeds available *ad libitum*.

On October 1, room temperature was decreased to 12°C to facilitate the entry to torpor. On November 1, ground squirrels were transferred to new cages with extra bedding material and no food and moved to a 24 h dark environmental chamber at 5°C to facilitate deep torpor. Ground squirrels were housed in this environmental chamber until early March, when they were transferred to new cages at 21°C–22°C with chow and sunflower seeds available *ad libitum* and a 12h–12h light-dark cycle. Water remained available *ad libitum* at all times.

At different times throughout the hibernation season (Table 1; Supplementary Table S1), ground squirrels were transferred to a decapicone restraint and euthanized by rapid decapitation via guillotine for tissue collection. Collection timepoints represented the following points in the hibernation season: September Active (1_SEPT), in mid-September; Fall Torpor (2_OCT), in mid-October; Winter Torpor (3_TOR), in December and January; Interbout Arousal (4_IBA), in December, January, and February; and March Active (5_MAR), in late March. Rectal body temperatures were taken at the time of sacrifice during OCT, TOR, and IBA timepoints from October to February (Table 1). IBA status was determined after animals were placed in torpor conditions by investigator assessment of animal activity and body temperature at the time of sacrifice. In particular, IBA animals display movement not seen in torpid animals. This includes shivering, heavy breathing, body extension, and movement of limbs. These various behaviors can occur at a wide range of body temperatures that were recorded at the time of sacrifice. We cannot determine whether these animals are arousing or entering torpor because they are not telemetered. Ground squirrel tissues were surgically collected after euthanasia and snap-frozen using liquid nitrogen, then stored at −80°C until use. White adipose tissues used in this study were taken from the abdomen. Aliquots of WAT from each animal (*N* = 47 biological replicates, 87% female, distributed amongst five timepoints, Table 1) were weighed using a chilled aluminum block and transferred to the UNL Proteomics and Metabolomics Facility on dry ice for metabolomics analysis.

**TABLE 1:** Animal characteristics summary.

Group	1_SEPT	2_OCT	3_TOR	4_IBA	5_MAR	Total
N total	9	9	13	8	8	47
(N male)	1	0	3	2	0	6
Average Body Temp at Sacrifice (T_b_, °C)	N/A (Active)	14.3 ± 3.4	5.7 ± 0.3	26.2 ± 13.8	N/A (Active)	N/A
Average Weight at Capture (W_i_, g)	144.6 ± 31.4	111.6 ± 21.3	161.0 ± 51.4	117.4 ± 38.6	165.3 ± 11.9	151.9 ± 41.0
Average Weight at Sacrifice (W_f_, g)	232.2 ± 42.7	213.6 ± 32.0	176.2 ± 16.5	187.8 ± 33.6	179.4 ± 25.8	196.6 ± 36.4
Average Weight in Late October (W_oct_, g)	N/A	N/A	199.1 ± 22.5	246.8 ± 48.8	269.4 ± 32.0	231.6 ± 45.1

Summary of ground squirrel characteristics. Detailed information on group assignment, body weight, sex, body temperature, date of collection, and date of sacrifice for all 47 ground squirrels used in this study is located in Supplementary Table S1. Values indicate group mean ± standard deviation for all rows except N total and N male.

### 2.2 Untargeted metabolomics using liquid chromatography—tandem mass spectrometry (LC-MS/MS) and data analysis

For each animal an aliquot of 150 mg of white adipose tissue was extracted using a chilled solution of water:chloroform:methanol (3:5:12) spiked with 10 µL of 100 µM of CUDA (12-[(cyclohexylcarbamoyl)amino]dodecanoic acid) as internal standard. The samples were disrupted and homogenized by adding two stainless steel beads (SSB 32) using the TissueLyserII (Qiagen, Germantown, MD, United States) at 25 Hz for 8 min. After centrifugation at 16,000 g for 5 min at 4°C, the supernatants were collected and transferred to a new tube. The samples were back extracted using the same solution and the supernatant collected after centrifugation was combined to the first one. To separate the water/methanol phase from the chloroform, a chilled solution of water: chloroform (3:4.5) was added to the supernatant, vortexed and centrifuged for 5 min at 16,000 g at 4°C. The upper phase was transferred to a new tube and dried down using a speed-vac. The pellets were re-dissolved in 15% methanol. Blank tubes were extracted alongside the samples to remove contaminant background from the data analysis. In addition, an aliquot of the samples was pooled to make a quality control (QC) sample which was run every 10 samples and samples were run using a randomized sequence order (Supplementary Table S4) to correct for batch effect.

A HILIC (Hydrophilic Interaction Liquid Chromatography)-MS/MS workflow running on a Vanquish LC system interfaced with a QE-HF mass spectrometer was used to profile the metabolites in negative and positive ion mode, as previously described in (Grzybowski et al., 2022). Data from LC-MS/MS analysis were processed with Compound Discoverer software v3.1 (ThermoFisher Scientific, Waltham, MA, United States) for peak detection, deconvolution, alignment, quantification, normalization, and identification/annotation. The annotation was done using several mass spectral libraries; the Thermo mzCloud library (20,905 entries), the MoNA (MassBank of North America) LC-MS/MS library for positive (98,154 entries) and MS/MS negative (43,457 entries), and in house library created using authentic standards run with the same conditions as the samples. The annotated peaks were manually reviewed for peak shape, chromatogram alignment integrity and MS/MS match. The annotated metabolites (Supplementary Table S2) were classified according to (Schymanski et al., 2014) in accordance with the Metabolomics Standards Initiative guidelines (Sumner et al., 2007). After processing the data as described in the next section, some unknown features (including MS/MS data) with interesting abundance pattern between the different groups were further analyzed using MS-Finder v 3.5 (Tsugawa et al., 2016; Lai et al., 2018), a software for structure elucidation using MS and MS/MS spectra of unknown compounds, and SIRIUS 4 (Dührkop et al., 2019) which integrates CSI:FingerID for searching molecular structure databases.

### 2.3 Statistical analysis

Feature intensity tables for all compounds were analyzed using MetaboAnalyst 5.0 (https://www.metaboanalyst.ca/). Feature intensity tables were log_10_ transformed and auto scaled (mean-centered and divided by the standard deviation of each variable). Boxplot representations of annotated feature intensities display group mean (yellow pip), group median (horizontal line), 25th-75th percentiles (box), and 95% confidence interval of the median (whiskers). Boxplots displayed in Figure 3 display log_10_-transformed and auto scaled feature peak intensity values, and boxplots in the Supplementary Data (Supplementary Figures S2, S3) display both the input feature intensities (original) and normalized intensity values as described for Figure 3. Annotated feature names were trimmed of any notation indicating chirality. Metabolite names were searched using the Human Metabolome Database (https://hmdb.ca/), PubChem (https://pubchem.ncbi.nlm.nih.gov/), and ChemSpider (Royal Society of Chemistry, http://www.chemspider.com/) to identify chemical synonyms of annotated features.

One-way analysis of variance and principal component analysis were calculated separately for features in the positive and negative ion modes. Tukey’s HSD test was used to identify between-timepoint differences. One-way ANOVA and Tukey’s HSD results were considered significant for *p* < 0.05 after Benjamini–Hochberg FDR correction. A hierarchical clustering heatmap (Figure 2) was generated using a list of annotated metabolites merged from both ion modes. Subgroup analysis for pairwise volcano plots was conducted separately for each ion mode (Figure 5), with a *t*-test threshold for significance of *p* < 0.01 after B-H FDR correction. Pathway and enrichment analysis were conducted using a unified list of annotated compounds corrected for features found in both ion modes and compared against RefMet chemical super class (Metabolomics Workbench, https://www.metabolomicsworkbench.org) and KEGG metabolite pathways (https://www.genome.jp/kegg/). Figures were assembled using Illustrator (Adobe) and Prism 9 (GraphPad).

## 3 Results

**FIGURE 1:**
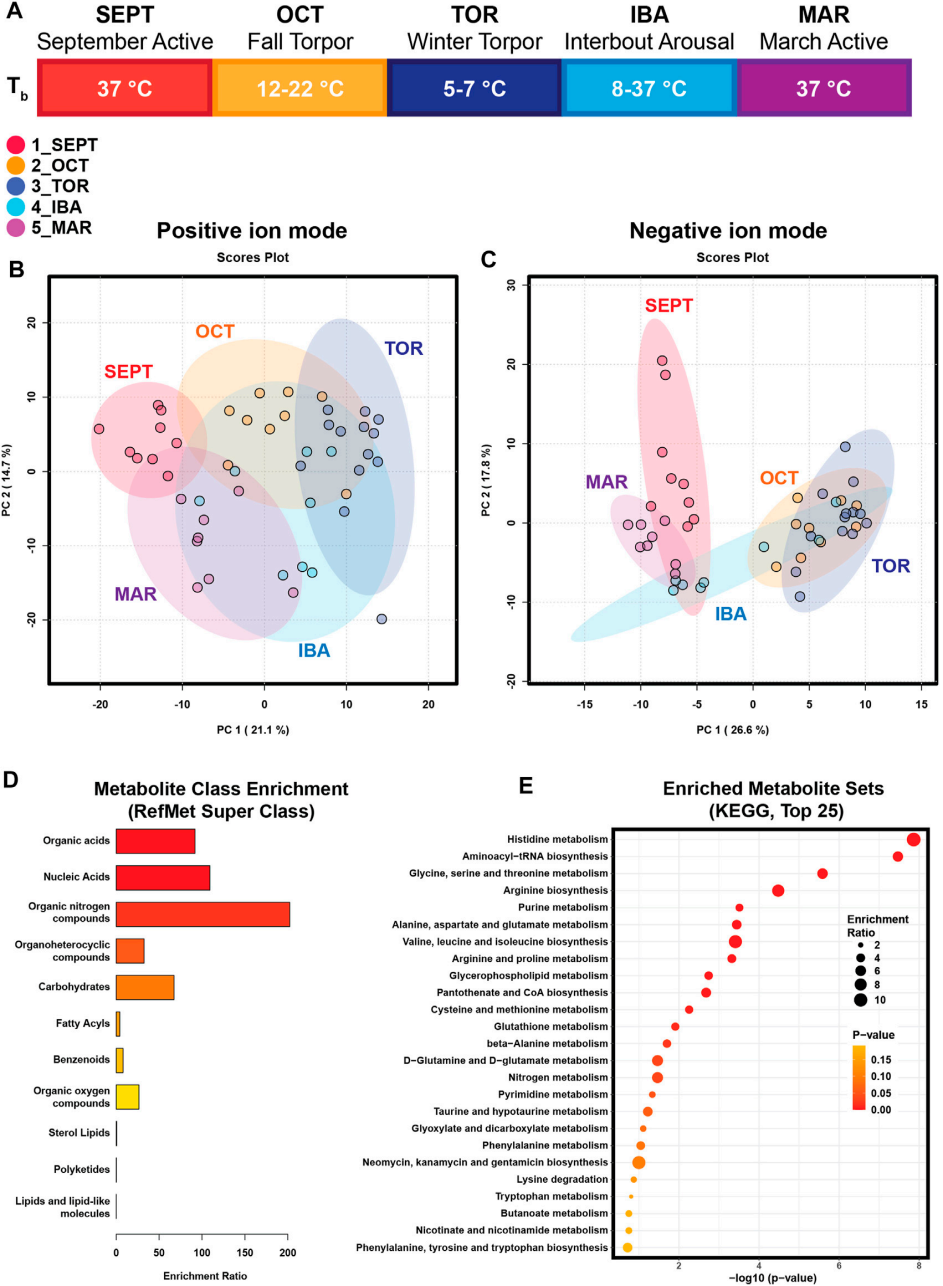
Principal Component Analysis and Enrichment Summaries. **(A)** Schematic of the hibernation season and study timepoints. SEPT (September Active); OCT (October, Fall Torpor); TOR (December-February, Winter Torpor); IBA (December-February, Interbout Arousal); MAR (March Active). Body temperatures (Tb) for ground squirrels at these timepoints are indicated. **(B)** Principal Component Analysis of annotated features from positive-mode HILIC MS. **(C)** Principal Component Analysis of annotated features from negative-mode HILIC MS. **(D)** Enrichment analysis output for chemical super-class. Annotated metabolite lists from positive and negative ion modes were combined and submitted to MetaboAnalyst for metabolite class enrichment analysis, compared against Metabolomics Workbench RefMet for chemical Super Class. Enrichment Ratio indicates the ratio of annotated features found to expected. **(E)** Enrichment analysis output for KEGG pathways enriched in annotated white adipose polar metabolites. Annotated metabolite lists were unified and submitted to MetaboAnalyst 5.0 for metabolite pathway enrichment analysis, compared against KEGG metabolic pathways. Enrichment ratio represents the ratio of observed metabolites in a given KEGG pathway to an expected number of observations.

To evaluate the WAT metabolome throughout the hibernation season, animals from timepoints corresponding to five conditions in the seasonal progression of hibernation were euthanized for tissue collection (Figure 1A; Table 1; Supplementary Table S1). September Active (1_SEPT) animals were sacrificed in September, during euthermic weight gain. Body temperatures in the Fall Torpor (2_OCT) group ranged from 12°C to 22°C (OCT T_b_ mean and SD 14.3°C ± 3.4°C), indicating that these animals were entering shallow bouts of torpor in preparation for deep winter torpor. Winter Torpor (3_TOR) body temperatures at sacrifice ranged from 5.2°C to 6.3°C (TOR T_b_ 5.7°C ± 0.3°C). Interbout arousal (4_IBA) animals had highly variable body temperatures ranging from 8.2°C to 37°C (IBA T_b_ 26.2°C ± 13.8°C). IBA status was differentiated from TOR at the time of sacrifice by investigators’ judgment of body temperature and activity level (see Section 2). March Active (5_MAR) ground squirrels were sacrificed in mid-late March after being returned to normal housing conditions and provided food *ad libitum*.

Body weight at the time of capture for all ground squirrels in this study was 151.9 ± 41.0 g (mean ± SD). Ground squirrels sacrificed in September and October gained an average of 87.7 ± 56.0 g and 102.0 ± 40.8 g total body weight, respectively, from capture to sacrifice. Animals weighed in late October (those in the Torpor, IBA, and March Active groups) weighed 231.6 ± 45.1 g, having gained an average of 64.9 ± 58.8 g after capture. Hibernating ground squirrels progressively lost body mass somewhat proportionate to the time spent in hibernation: ground squirrels sacrificed in December and January (3_TOR) were 22.9 ± 21.5 g lighter at sacrifice than in October; IBA animals sacrificed in December, January, and February were 59.0 ± 34.3 g lighter at sacrifice than October; and March active ground squirrels sacrificed in March were 90.0 ± 38.5 g lighter at sacrifice than in October (Table 1; Supplementary Table S1). High variance in individual weight gain in this cohort may reflect differences in animal age, total time of housing, or animals’ acclimation to housing.

WAT samples were extracted via liquid-liquid extraction using chilled water: chloroform: methanol for untargeted profiling of polar metabolites using hydrophilic interaction liquid chromatography-mass spectrometry (HILIC-MS).

A total of 454 features were detected in positive ion mode and 206 in negative ion mode using untargeted HILIC-MS, after manual curation of the data. The log of octanol-water partition coefficient, log K_ow_, showed a negative correlation when plotted against the retention time of the HILIC-MS metabolites (Supplementary Figure S1) as expected according to (Theodoridis et al., 2023). Amongst the features detected, 166 compounds were annotated in positive-mode analysis and 79 in negative-mode analysis. A total of 21 annotated compounds overlapped between both modes. MS peak intensity lists from positive and negative modes were analyzed separately for ANOVA and principal component analysis rather than being summed or averaged. Compounds annotated in both ion modes were separately included in statistical analysis of both positive and negative ion mode feature lists. Most of the features annotated in both ion modes were either concordant or partially concordant in seasonal intensity profile (Supplementary Figure S5). Only two annotated features, serine and 2-aminonicotinic acid, were non-concordant in seasonal feature intensity profile.

Principal component analysis (PCA) revealed clustering of samples in accordance with seasonal timepoints in both the positive and negative ion modes (Figures 1B, C). In both ion modes, timepoint groups were clustered and adjacent or overlapping with groups of similar activity status: SEPT and MAR active timepoints were adjacent in both ion modes, OCT and TOR groups were adjacent or clustered together, and IBA groups were dispersed relative to the other groups, possibly reflecting a brief normothermic phenotype flanked by multi-day hypothermic torpor bouts. Annotated feature lists from both ion modes were unified and submitted to MetaboAnalyst for chemical class and metabolic pathway enrichment analysis. Metabolite class analysis compared against the Metabolomics Workbench RefMet utility for chemical super class demonstrated enrichment of organic acids, nucleic acids, organic nitrogen compounds, carbohydrates, and organoheterocyclic compounds (Figure 1D). Notably, lipids were mostly absent from the annotated features, suggesting that liquid-liquid extraction and hydrophilic interaction chromatography successfully enriched WAT extracts for polar compounds. Pathway analysis comparing the unified annotated feature list against KEGG metabolic pathways demonstrated enrichment of amino acid metabolism, purine metabolism, phospholipid synthesis, and pantothenate-coenzyme A biosynthetic pathways (Figure 1E).

**FIGURE 2:**
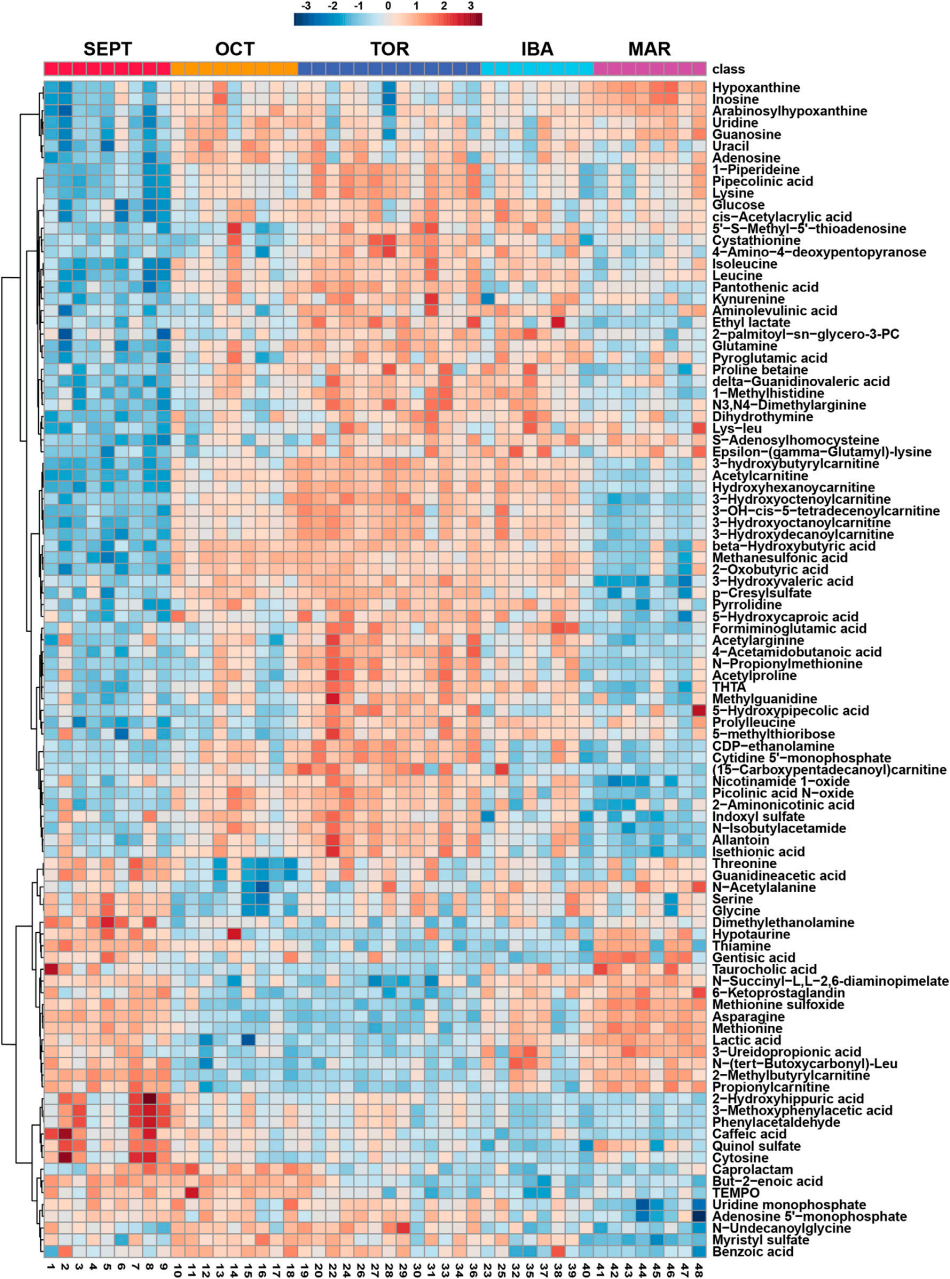
Hierarchical Clustering Heatmaps for Top 100 Features. Hierarchical clustering of the top 100 annotated metabolites from a merged list of both positive and negative ion modes, as assessed by one-way ANOVA *p*-value. Groups were not reordered to preserve the seasonal progression of timepoints through hibernation. All biological replicates are shown (sample IDs are displayed at the bottom of each column). Normalized, log_10_ transformed, and auto scaled feature intensity values ranged from −3 to 3.

Amongst annotated features, one-way analysis of variance identified 118 significant features from positive mode and 56 significant features from negative mode. A heatmap representing the hierarchical clustering of the top 100 annotated features from a merged list of both ion modes (as assessed by ANOVA *p*-value) is shown in Figure 2. Boxplots for every annotated feature in this study are separated by ion mode and shown in Supplementary Figures S2, S3. Statistical summary tables including all annotated and unknown HILIC-MS features, separated in tabs by ion mode and analysis type, are located in Supplementary Table S3.

**FIGURE 3:**
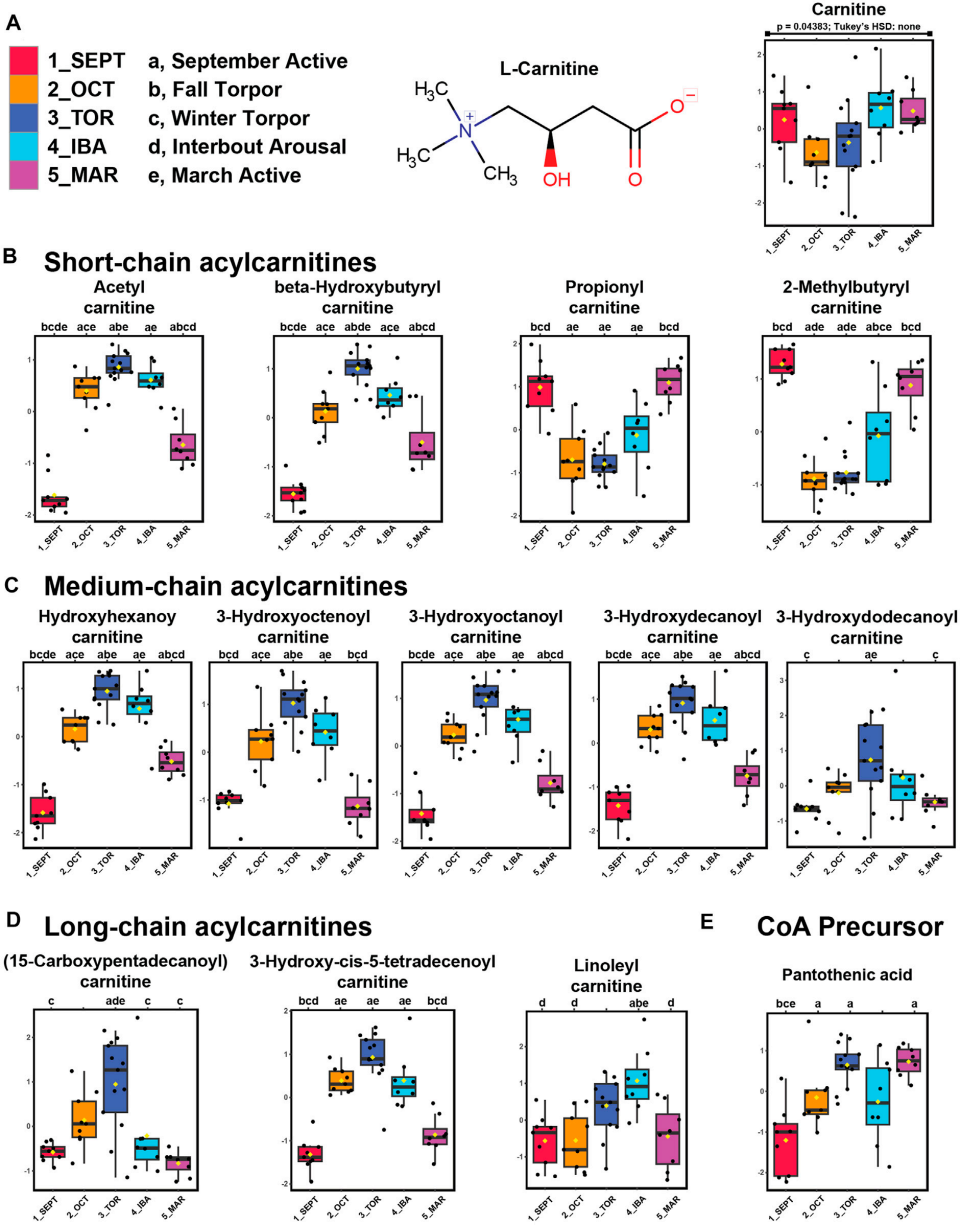
Acylcarnitines and pantothenic acid are elevated in WAT during torpor. Feature intensity boxplots of carnitine, short-chain acylcarnitines, medium-chain acylcarnitines, long-chain acylcarnitines, and pantothenic acid throughout the hibernation season. Panels: boxplot representations of feature intensity. Normalized feature intensities were log_10_ transformed and auto scaled (mean-centered and divided by the standard deviation of each variable). Yellow pip: mean; horizontal central line: median; boxes: 25th-75th percentiles; whiskers: 95% confidence interval of the median. Boxplot Y-axes represent the fold change of feature intensity after log_10_ transformation and auto scaling. Statistical marks: each timepoint is assigned a letter from a through e in seasonal order: SEPT = a; OCT = b; TOR = c; IBA = d; MAR = e (Figure 3A). Significant Tukey’s HSD results (*p* < 0.05) between groups are indicated by the presence of the opposing group letter(s) over each boxplot position. Supplementary Table S3 contains comprehensive ANOVA and Tukey’s results for all features. **(A–D)** Most acylcarnitines except propionyl- and 2-methylbutyrylcarnitine increased in abundance during October fall torpor, winter torpor, and interbout arousal timepoints. **(E)** The coenzyme A precursor, pantothenic acid, was elevated during OCT, TOR, and MAR relative to SEPT.

We identified carnitine and twelve acylcarnitine derivatives from positive-mode analysis of the ground squirrel WAT metabolome. Of these, the majority were elevated during torpor and torpor-associated timepoints (Figures 3A–D). Free carnitine remained at consistent levels throughout the seasonal timepoints. Acetylcarnitine, hydroxybutyrylcarnitine, and all medium- and long-chain acylcarnitines were found to be elevated during OCT, TOR, and IBA relative to active timepoints. Only propionylcarnitine and 2-methylbutyrylcarnitine, both short-chain odd-numbered acylcarnitines, were higher during active timepoints relative to OCT and TOR. The coenzyme A precursor, pantothenic acid (vitamin B5) increased in abundance during TOR relative to SEPT and IBA timepoints (Figure 3E).

**FIGURE 4:**
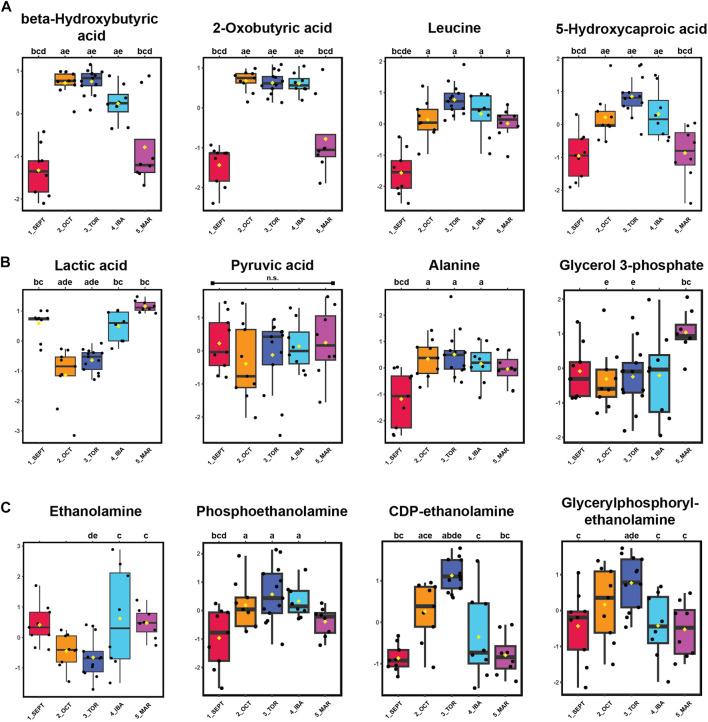
Ketones, Ketogenic Amino Acids, and Glycerophospholipid Precursors are Elevated in WAT During Torpor. Panels: log_10_ transformed and auto-scaled boxplot representations of feature intensity, as in Figure 3. Statistical marks: each timepoint is assigned a letter from a through e in seasonal order: SEPT = a; OCT = b; TOR = c; IBA = d; MAR = e. Significant Tukey’s HSD results (*p* < 0.05) between groups are indicated by the presence of the opposing group letter(s) over each boxplot position. Supplementary Table S3 contains comprehensive ANOVA and Tukey’s results for all features. **(A)** Feature intensity boxplots of ketones, ketogenic metabolites, and medium-chain fatty acids. **(B)** Glycolytic by-products and transamination pathway metabolites. **(C)** Glycerophospholipid precursors.

Alternative fuels such as ketones beta-hydroxybutyrate (BHB) and 2-oxobutyrate, as well as the ketogenic amino acids leucine and isoleucine, were elevated during torpor relative to active timepoints. An increase in 5-hydroxycaproic acid, a medium-chain fatty acid, during torpor-associated timepoints suggests increased mobilization of intracellular lipids during that time (Figure 4A). Pyruvate abundance remained consistent throughout the hibernation season, whereas its corresponding transamination metabolite, alanine, became elevated during torpor. Lactic acid levels decreased substantially during OCT and TOR timepoints (Figure 4B) and show the opposite profile of BHB and 2-oxobutyrate for those timepoints, indicating decreased glucose utilization and elevated ketone synthesis and/or uptake in WAT during torpor.

Phospholipid precursors ethanolamine, phosphoethanolamine, and CDP-ethanolamine were annotated. CDP-ethanolamine and the phospholipid breakdown product, glycerylphosphorylethanolamine, were elevated during torpor relative to active and IBA states (Figure 4C). For the fifteen alpha-amino acids identified in this study, most were elevated during torpor-associated timepoints except for glutamic acid, aspartic acid, asparagine, and methionine, which decreased in abundance during torpor to varying degrees (Supplementary Figures S2, S3). Valine, histidine, arginine, tyrosine, and cysteine were not annotated here, although 1-methylhistidine was found in positive-mode analysis.

**FIGURE 5:**
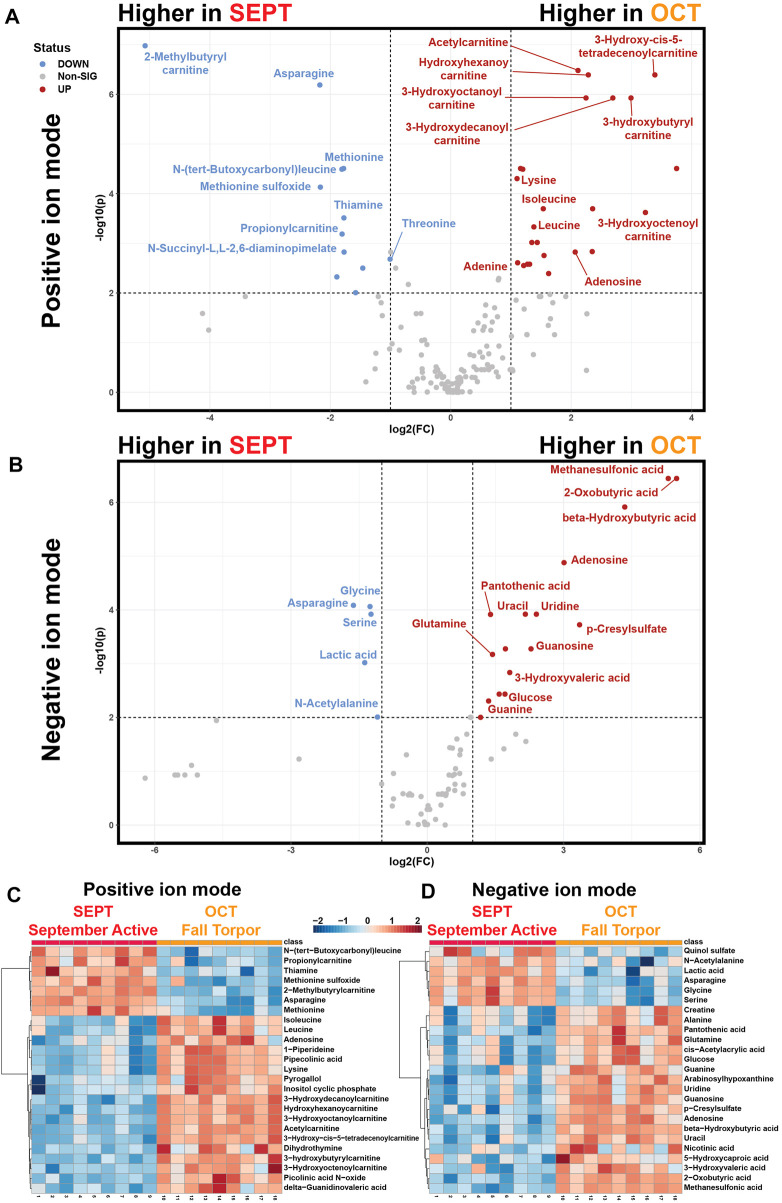
Pairwise Comparisons between September Active and Fall Torpor WAT. Pairwise comparison between feature intensities from the September active (SEPT) and fall torpor (OCT) timepoints. **(A)** Positive-mode volcano plot for SEPT vs. OCT. *x*-axis: log2 fold change (FC) of normalized feature intensity. *y*-axis: −log_10_(p) from pairwise *t*-test. Significance threshold was set to *p* < 0.01 for pairwise subgroup comparisons. Statistical tables for Figure 5 are included in Supplementary Table S5. Volcano plot is arranged so that features with higher intensity in SEPT are to the left side and features higher in OCT are to the right. **(B)** Negative-mode volcano plot for SEPT vs. OCT, as in 5A. **(C)** Positive ion mode heatmap for the pairwise comparison between SEPT and OCT. All biological replicates (1–18) from 1_SEPT and 2_OCT groups are shown. Normalized feature intensity values ranged from −2 to +2. Top 25 features as assessed by *t*-test are shown. **(D)** Negative ion mode heatmap for the pairwise comparison between SEPT and OCT, as in 5C.

Pairwise analysis of neighboring active timepoints (Figure 5, SEPT vs. OCT) showed that short- and medium-chain acylcarnitines, purines, and ketones were elevated during fall torpor relative to late summer active timepoints (Figures 5A, B). Asparagine, glycine, serine, lactic acid, and odd-numbered short-chain acylcarnitines were less abundant in OCT relative to SEPT. Glucose was more abundant in OCT WAT samples, coinciding with the shift away from circulating glucose utilization in torpor (Buck et al., 2002). Heatmaps representing the top 25 annotated metabolites assessed by *t*-test for each MS ion mode are shown in Figures 5C, D. Volcano plot tables including both annotated and unknown features are located in Supplementary Table S3.

## 4 Discussion

As the main fat storage depot, white adipose tissue (WAT) provides hibernating mammals with a reliable source of energy to survive prolonged periods of starvation and near-freezing body temperatures (Florant et al., 1990; Florant et al., 2004; Dark, 2005). Energy is stored in WAT as triacylglycerol molecules that contain three fatty acid chains. Several hibernating species rely on these lipid stores to meet their energy needs in the absence of feeding over a span of several months. This exclusive reliance on WAT over a 4–5-month period demands a tissue that has the versatility to release carbon compounds that can be used as fuel in multiple organs over a wide range of body temperatures.

To identify molecules that facilitate WAT function and serve as part of the supply chain from triacylglycerol stores to a mobilized fuel source, we used a metabolomic approach to identify polar metabolites in ground squirrels at five points throughout the hibernation season. Our findings show that ground squirrel WAT is an active metabolic organ that may contribute to whole-body metabolism in addition to serving as a source of circulating glycerol and non-esterified fatty acids. We found that fatty acid beta-oxidation precursors, in the form of varying acylcarnitines and coenzyme A precursors, were nearly uniformly elevated during fall and winter torpor timepoints.

Conversion of triacylglycerols to non-esterified fatty acids requires the activity of various lipases. To help with this process during torpor, WAT in the thirteen-lined ground squirrel expresses pancreatic triacylglycerol lipase which can liberate fatty acids at temperatures as low as 0°C (Andrews et al., 1998; Bauer et al., 2001; Squire et al., 2003). The unexpected finding of a pancreatic lipase in white adipose over 20 years ago underscores the versatility of ground squirrel WAT and suggests that there could be other novel mechanisms at play. This includes the generation of numerous carnitine derivatives found in this study that can be transported in or out of the mitochondria as fuel for adipocytes, as well as the generation of smaller carbon compounds that be transported and metabolized in other tissues.

We show that alternative fuels such as ketones beta-hydroxybutyrate (BHB) and 2-oxobutyrate, as well as the ketogenic amino acids leucine and isoleucine, were elevated in WAT during torpor relative to active timepoints. Unlike the larger fatty acids from which it is derived, BHB is a 4-carbon carboxylic acid that passes through the blood and provides an alternative fuel to various organs throughout the body (Cuenoud et al., 2020). The D-stereoisomer of BHB is elevated in ground squirrel blood during torpor and is the preferred fuel over glucose in both the brain and heart during hibernation (Andrews et al., 2009). We previously used a proteomic approach (Russeth et al., 2006) to show that the ground squirrel heart increases its capacity to use ketones during hibernation by up-regulating Succinyl CoA transferase (SCOT). SCOT is an important enzyme because it catalyzes the rate-limiting step in ketone metabolism by producing acetoacetyl-CoA, which is further metabolized to form two molecules of acetyl-CoA that enter the TCA cycle and lead to the production of ATP. SCOT mRNA (encoded by the OXCT1 gene) is present in WAT and other ground squirrel tissues throughout the year (Hampton et al., 2013; https://www.d.umn.edu/∼mhampton/GB18.html).

**FIGURE 6:**
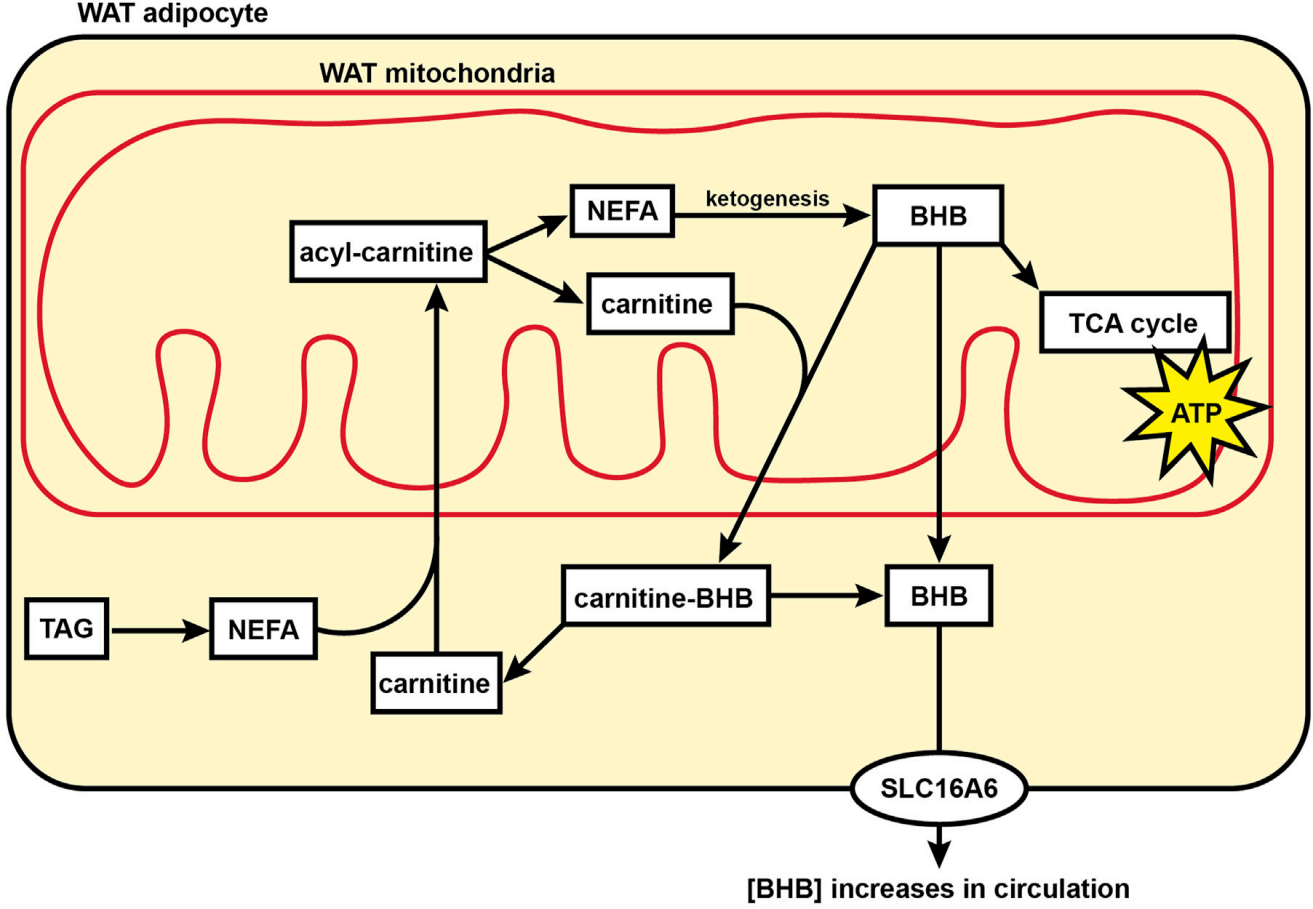
Model showing fuel utilization and transport in the mitochondria of hibernator WAT during torpor. During torpor, acylcarnitine content substantially increases for many acyl carbon chain lengths including NEFAs, indicating an increase in mitochondrial acyl carbon import and supporting a transition to fatty acid oxidation for cellular energy supply relative to glucose utilization. BHB levels in WAT increase substantially during torpor, supporting the hypothesis that WAT may be utilized by WAT mitochondria or directly contribute to circulating ketones during torpor. Abbreviations: ATP, adenosine triphosphate; BHB, beta-hydroxybutyrate; NEFA, non-esterified fatty acid; SLC16A6, solute carrier family 16 member 6; TAG, triacylglycerol; TCA cycle, tricarboxylic acid cycle; WAT, white adipose tissue.

The formation of ketones such as BHB was originally thought to occur exclusively in the liver of mammals. However, it was recently shown in mice that WAT can directly produce and secrete BHB (Nishitani et al., 2022). Our finding that BHB, BHB-carnitine, and ketogenic amino acids are elevated in the WAT metabolome during torpor suggests an alternate mechanism to produce BHB and support whole-body organ function in the absence of feeding during hibernation (Figure 6). The direct formation of BHB in WAT dispenses with a sole reliance on liver ketogenesis and provides an alternative source of this important 4-carbon fuel that can cross the blood-brain barrier and support brain function when brain glycolytic activity has been halted and circulating glucose levels are low (Andrews et al., 2009).

We selected a pairwise comparison between the September Active (SEPT) and Fall Torpor (OCT) timepoints for subgroup analysis (Figure 5). The comparison between these timepoints was particularly interesting, because the timepoints under comparison occurred before and during ground squirrel preparation for deep torpor. SEPT animals were housed at 21°C–22°C room air with food and 12h–12 h light-dark, whereas OCT animals received the same food and light-dark timing, but room air had been decreased to 12°C prior to OCT sacrifice. Throughout the mid-to-late fall season, hibernators are undergoing shallow torpor bouts (Russell et al., 2010), and are approaching or have reached their maximal body weight (Schwartz et al., 2015).

We found that OCT ground squirrel WAT, relative to SEPT, had numerous signs of torpor preparation. Elevated OCT levels of BHB and decreased lactate indicate that torpor-associated metabolic changes have been initiated, and we likewise observed an increase in OCT acylcarnitine abundance. Adenosine, also elevated in OCT, is closely associated with the onset of torpor (Jinka et al., 2011). Significant decreases in methionine, methionine sulfoxide, asparagine, and the dietary supplement thiamine may indicate ground squirrel metabolism has shifted towards torpor conditions (D’Alessandro et al., 2017) or that feeding has ceased following the OCT room temperature shift to 12°C.

It is worth noting that the CoA precursor, pantothenate, is generally thought of as a dietary-derived metabolite not synthesized directly in animal tissues (Depeint et al., 2006), yet we observed it to increase during torpor (Figure 3E) when ground squirrels are no longer feeding. Previous metabolomic investigations of ground squirrel plasma likewise found circulating pantothenic acid to be elevated during torpor relative to active timepoints (Nelson et al., 2010; Epperson et al., 2011). Although the precise cause cannot be determined from this study, this may result from pantothenate redistribution from other tissues during torpor. Alternatively, this may reflect activity of the ground squirrel gut microbiome, which has recently been shown to be important for other functions during torpor, including nitrogen recycling (Regan et al., 2022).

Phospholipids and phospholipid precursors were increased in pre-torpor active and winter torpor states (certain ethanolamines, particularly phosphoethanolamine and CDP-ethanolamine, see Figure 4). We hypothesize that these metabolites are incorporated into the growing lipid droplet during summer activity and feeding, and as lipids become mobilized from the WAT lipid droplet during winter torpor, these membrane components become newly available and thus increase in abundance. CDP-ethanolamine and glycerylphosphorylethanolamine levels decreased from TOR to IBA (Figure 4C), which may indicate the consumption and incorporation of these metabolites into new membrane synthesis or other recycling pathways during IBAs. Interestingly, previous liver metabolomics found ethanolamines as well as phosphocholine and phosphatidylcholine to be elevated late in IBAs (“entry to torpor” timepoints in Serkova et al., 2007), which could reflect tissue differences or could be attributable to differences in the specificity with which torpor-arousal timepoints were collected.

We profiled the abundance of fifteen alpha-amino acids (AAs) throughout the hibernation season in WAT (Supplementary Figures S2, S3; Supplementary Tables S2, S3). These AAs followed a variety of abundance patterns throughout hibernation. AAs that increased in abundance from SEPT through OCT into TOR, followed by a decrease in IBA and MAR included glutamine, proline, lysine, leucine, and isoleucine. Asparagine and methionine abundance dropped sharply from SEPT to OCT and TOR, increasing in IBA and MAR. Glycine and threonine abundance decreased from SEPT to OCT, but increased again in TOR, IBA, and MAR. Other alpha-AAs were found to be present in WAT but had less variation throughout their seasonal abundance profiles. In general, these findings concur with metabolomics analysis from other hibernator tissues including plasma (Epperson et al., 2011; D’Alessandro et al., 2017) and liver (Serkova et al., 2007). The branched-chain amino acids leucine and isoleucine were elevated in OCT, TOR, and IBA relative to SEPT in WAT, which differed from previous findings in liver where these were highest in summer timepoints (Serkova et al., 2007), possibly reflecting tissue differences in branched chain aminotransferase enzyme expression (Gillen et al., 2021).

Several modified amino acids were elevated in TOR relative to other timepoints, including acetylproline and acetylarginine. Previous work on hibernator plasma metabolomics (Epperson et al., 2011) identified acetylated amino acids as elevated in late torpor and the arousal from torpor to activity, followed by decreased abundance in IBAs, and suggested that these metabolites may be selectively salvaged during torpor in order to facilitate their reuse during interbout arousals. Although our annotated acetyl-AAs are distinct from those found in that previous study, they have a comparable abundance pattern and may reflect a similar mechanism in hibernator adipose tissue. This result therefore supports a role for interbout arousals in cellular maintenance during hibernation, as a necessary euthermic period where essential reactions can occur at a permissive temperature.

Further supporting the hypothesis that IBAs support hibernation by enabling cellular maintenance, we annotated several uremic toxins in the hibernator WAT metabolome (e.g., p-Cresylsulfate and N3,N4-dimethylarginine, see Figure 2 and Supplementary Material). These features became strongly elevated during TOR timepoints, with diminished abundance during IBAs. Although these steady-state measurements represent a snapshot in time and do not allow us to define the source and destination for these metabolites, this TOR-IBA abundance pattern suggests that IBAs are important for the clearance or recycling of metabolic by-products. Supporting the findings of Rice et al. (2020), we found urea to be relatively unchanged throughout the hibernation cycle which may indicate either the suppression of the urea cycle during torpor or relative balance in its steady-state inputs and outputs.

Environmental conditions impose extreme demands on the physiology of hibernating animals throughout the circannual hibernation cycle. To survive, hibernators must enact a whole-body hibernation phenotype in which every organ system must adapt and coordinate with the rest of the body. White adipose tissue undergoes some of the most striking changes in its configuration throughout the season, representing the majority of a hibernator’s summer weight gain and subsequent winter weight loss. WAT serves a crucial role as a major calorie depot for hibernating animals. Our findings demonstrate that WAT is a highly dynamic organ throughout the hibernation cycle that may contribute more directly to whole-body metabolic regulation than previously recognized.

## Data Availability

The original contributions presented in the study are included in the article/Supplementary Material, further inquiries can be directed to the corresponding author.
