# Supplementary figures and images for: Mass spectrometry of the white adipose metabolome in a hibernating mammal reveals seasonal changes in alternate fuels and carnitine derivatives
Source: Front Physiol. 2023 Jun 28;14:1214087. doi: 10.3389/fphys.2023.1214087 (PMC10337995; doi:10.3389/fphys.2023.1214087)

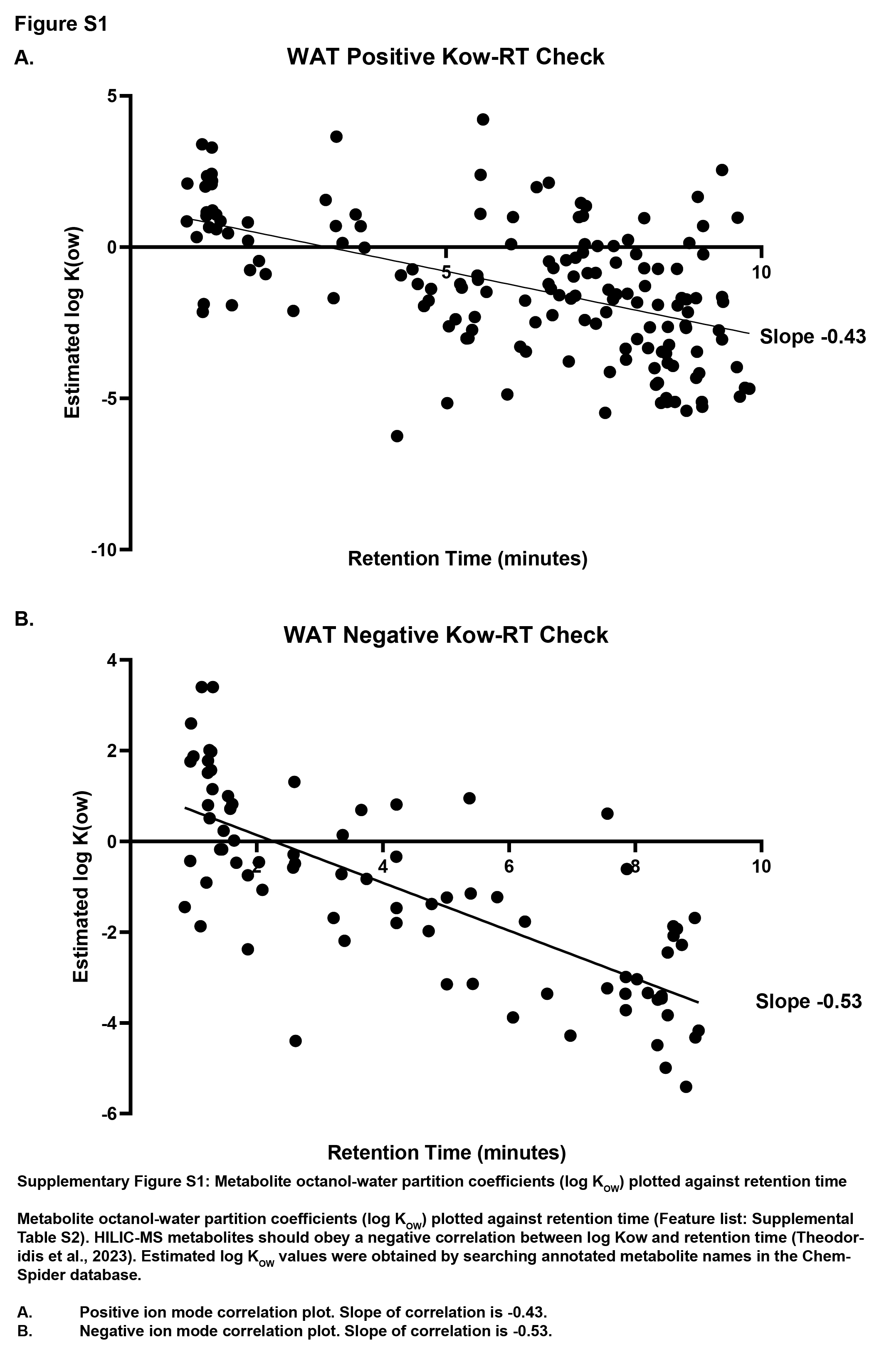

Supplement: Supplementary file 3 [file Image1.JPEG]
