# Supplementary material for: Mass spectrometry of the white adipose metabolome in a hibernating mammal reveals seasonal changes in alternate fuels and carnitine derivatives
Source: Front Physiol. 2023 Jun 28;14:1214087. doi: 10.3389/fphys.2023.1214087 (PMC10337995; doi:10.3389/fphys.2023.1214087)
Supplement: Supplementary file 4 [file Image2.pdf]

Figure S2

Supplementary Figure 2: Boxplot Representations of all Annotated Features from the Positive Ion Mode

Collated feature intensity box plots for all annotated features in the positive ion mode. Left panel: original feature intensities from peak intensity table (Supplemental Table S2, tab 1). Right panel: feature intensities after $\log_{10}$ transformation and auto scaling (values mean-centered and divided by the standard deviation).

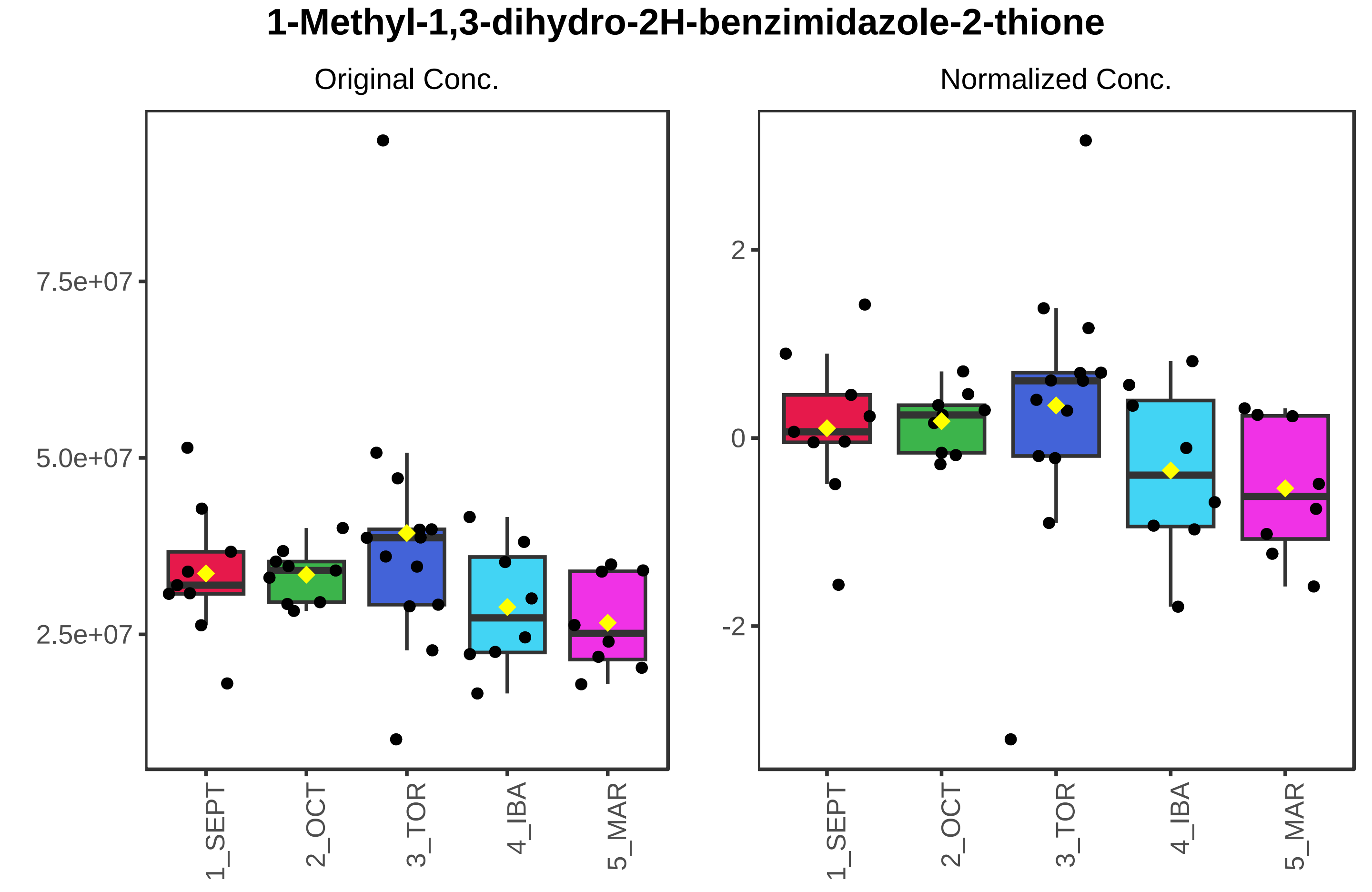

1-Methyl-1,3-dihydro-2H-benzimidazole-2-thione
Original Conc.
Normalized Conc.
7.5e+07
5.0e+07
2.5e+07
2
0
-2
1_SEPT
2_OCT
3_TOR
4_IBA
5_MAR

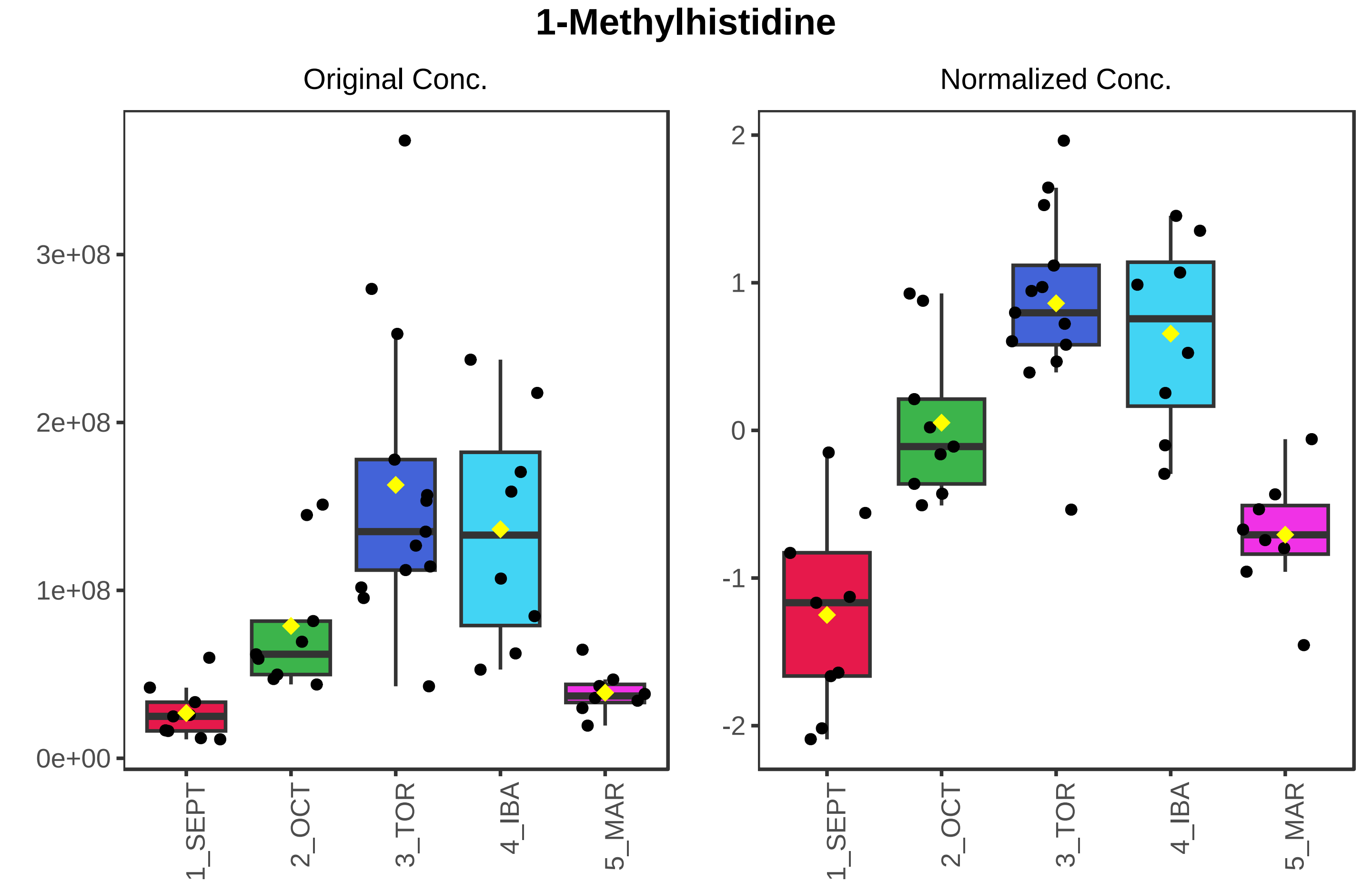
1-Methylhistidine
Original Conc.
Normalized Conc.
3e+08
2e+08
1e+08
0e+00
2
1
0
-1
-2
1_SEPT
2_OCT
3_TOR
4_IBA
5_MAR
1_SEPT
2_OCT
3_TOR
4_IBA
5_MAR

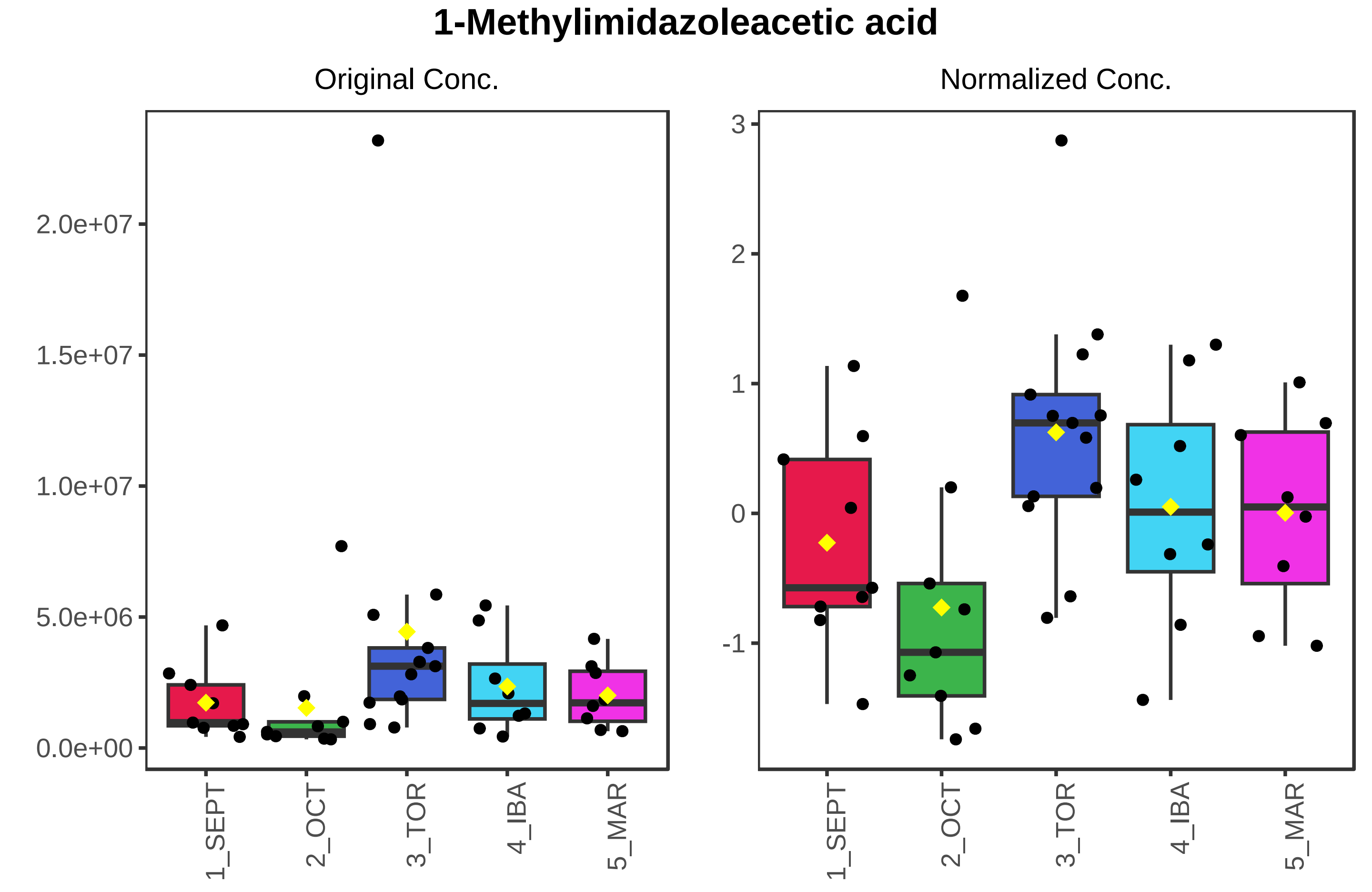

1-Methylimidazoleacetic acid
Original Conc.
Normalized Conc.
2.0e+07
1.5e+07
1.0e+07
5.0e+06
0.0e+00
3
2
1
0
-1
1_SEPT
2_OCT
3_TOR
4_IBA
5_MAR

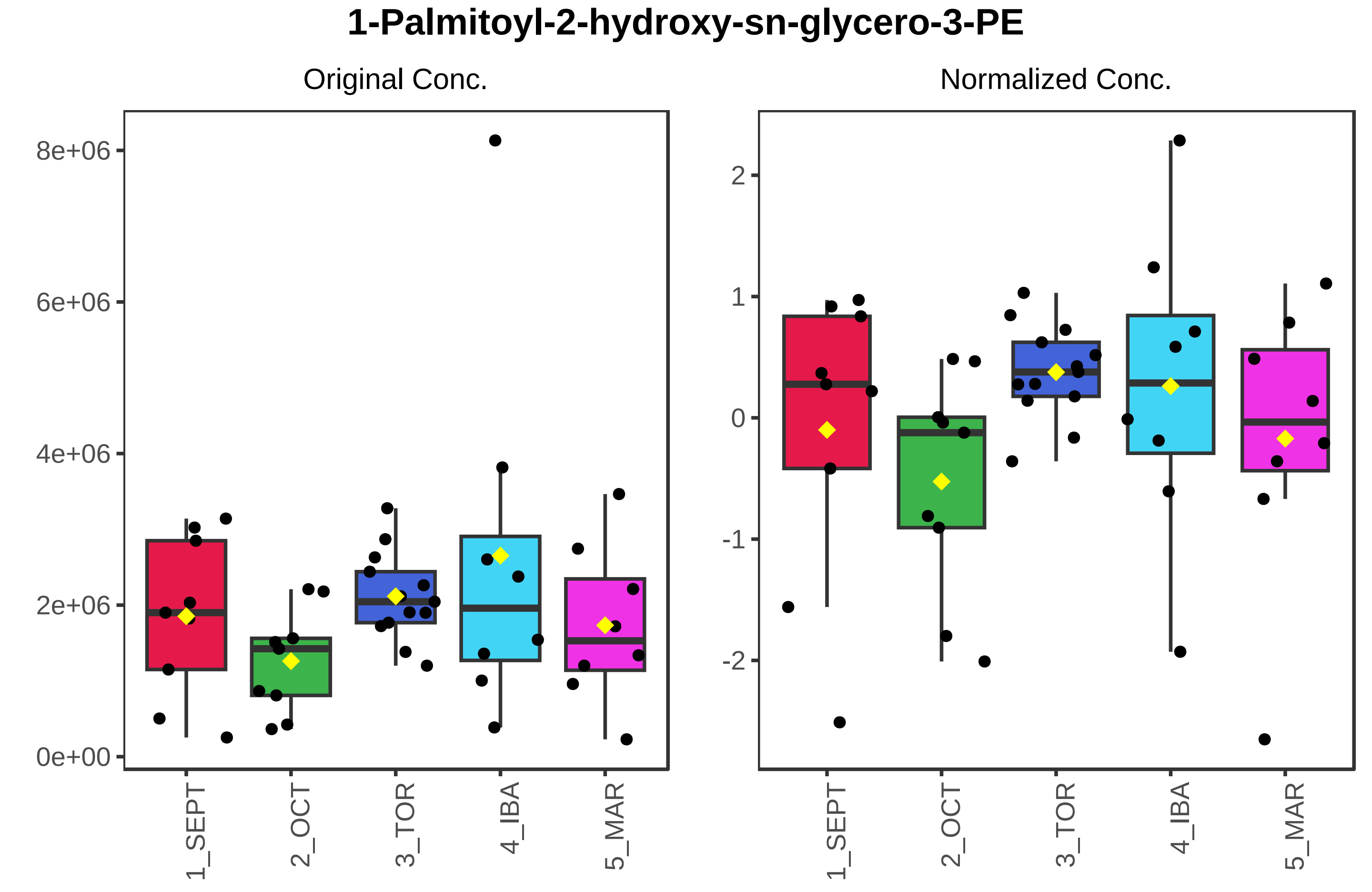

1-Palmitoyl-2-hydroxy-sn-glycero-3-PE
Original Conc.
Normalized Conc.
8e+06
6e+06
4e+06
2e+06
0e+00
2
1
0
-1
-2
1_SEPT
2_OCT
3_TOR
4_IBA
5_MAR

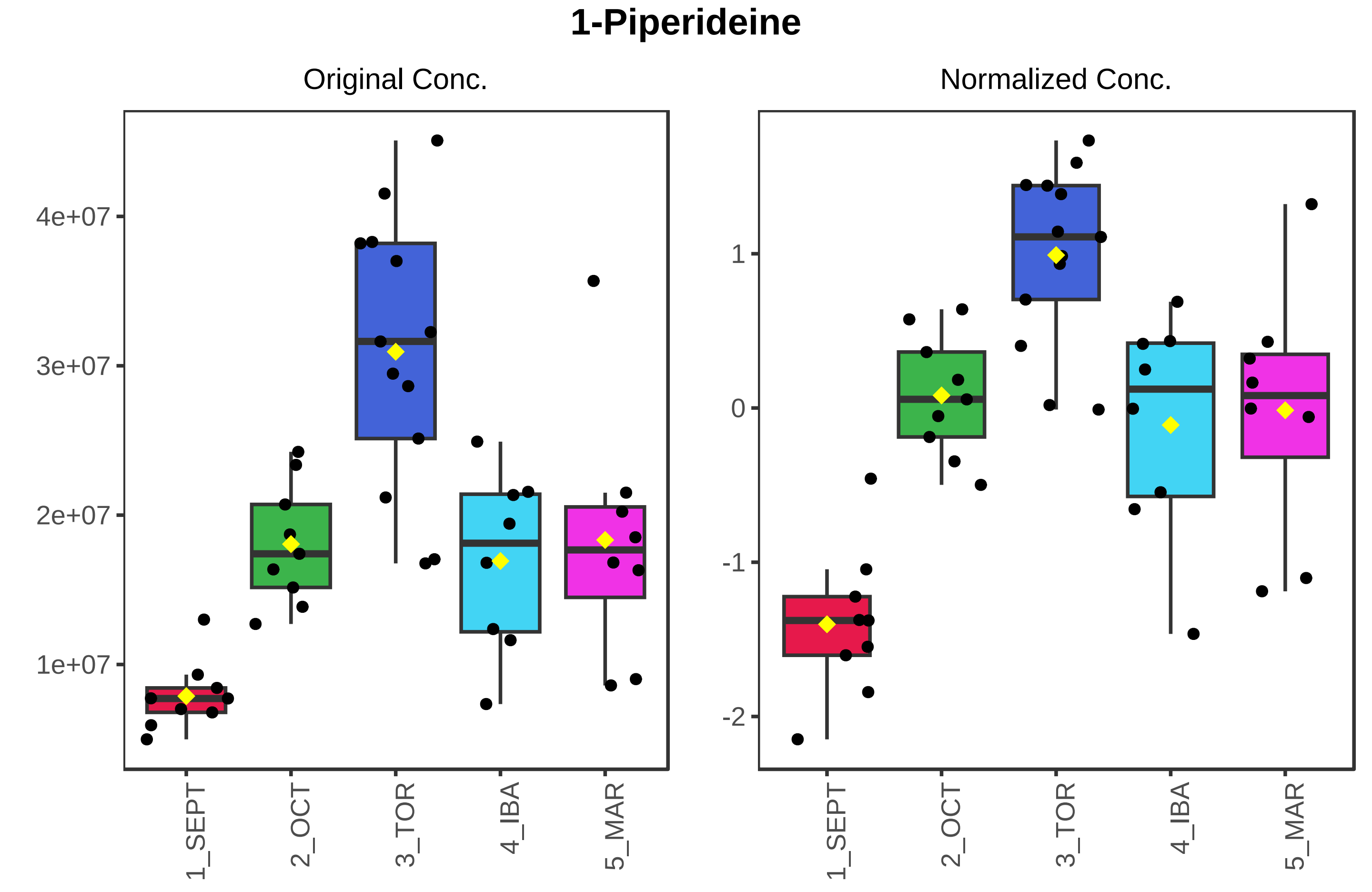

1-Piperideine
Original Conc.
Normalized Conc.
4e+07
3e+07
2e+07
1e+07
1
0
-1
-2
1_SEPT
2_OCT
3_TOR
4_IBA
5_MAR

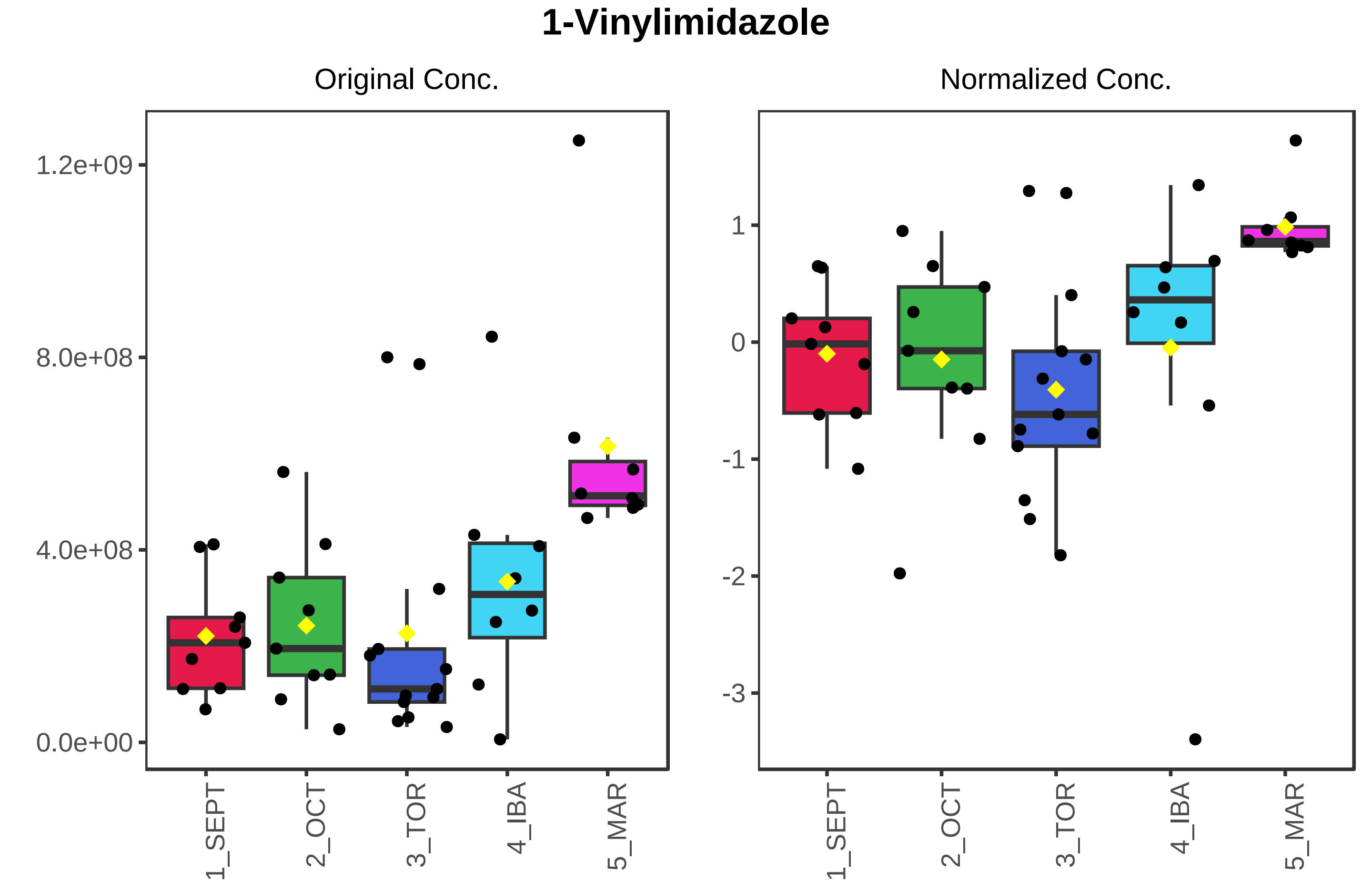

1-Vinylimidazole
Original Conc.
Normalized Conc.
1.2e+09
8.0e+08
4.0e+08
0.0e+00
1
0
-1
-2
-3
1_SEPT
2_OCT
3_TOR
4_IBA
5_MAR

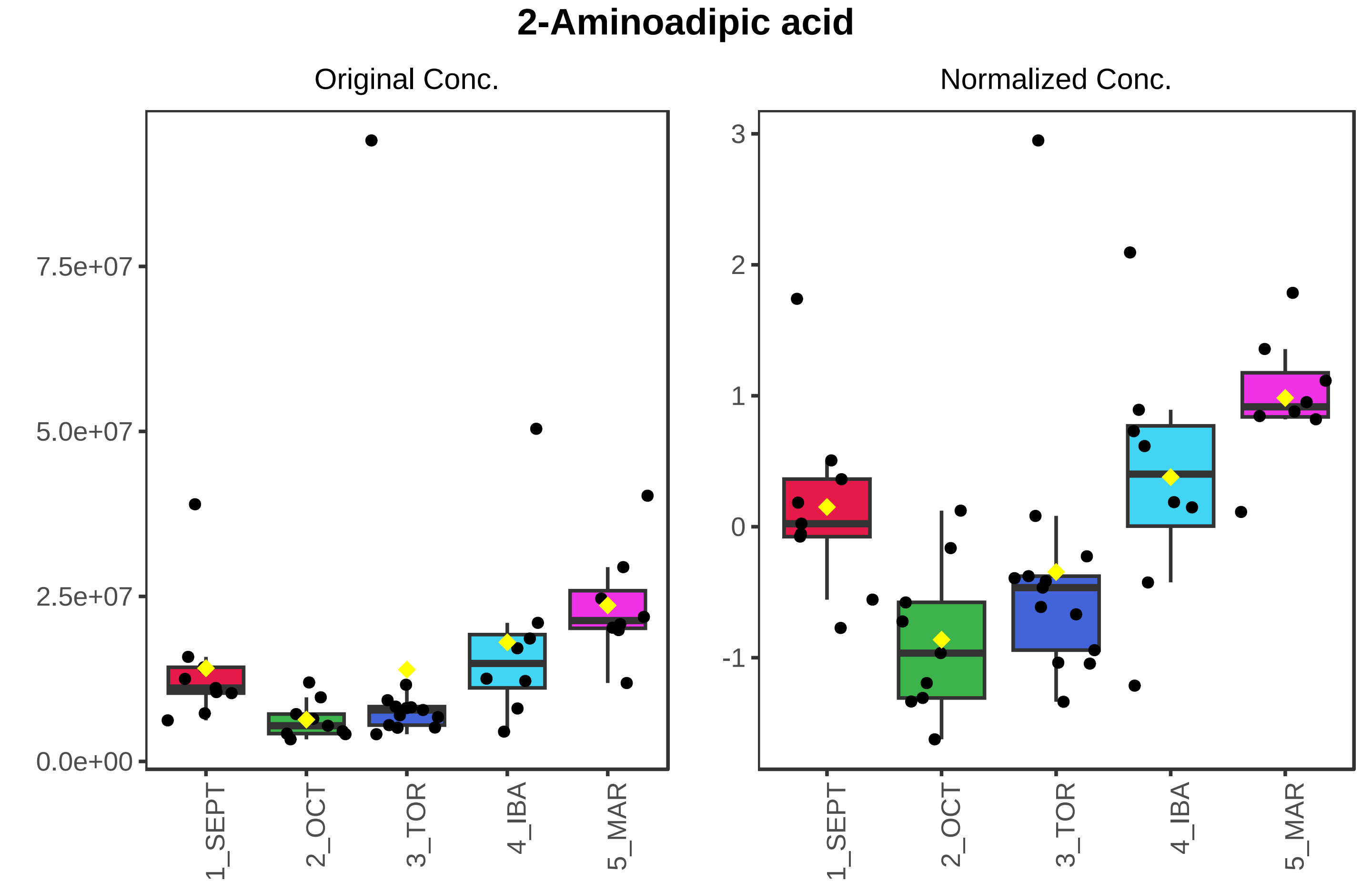
2-Aminoadipic acid
Original Conc.
Normalized Conc.
7.5e+07
5.0e+07
2.5e+07
0.0e+00
3
2
1
0
-1
1_SEPT
2_OCT
3_TOR
4_IBA
5_MAR
1_SEPT
2_OCT
3_TOR
4_IBA
5_MAR

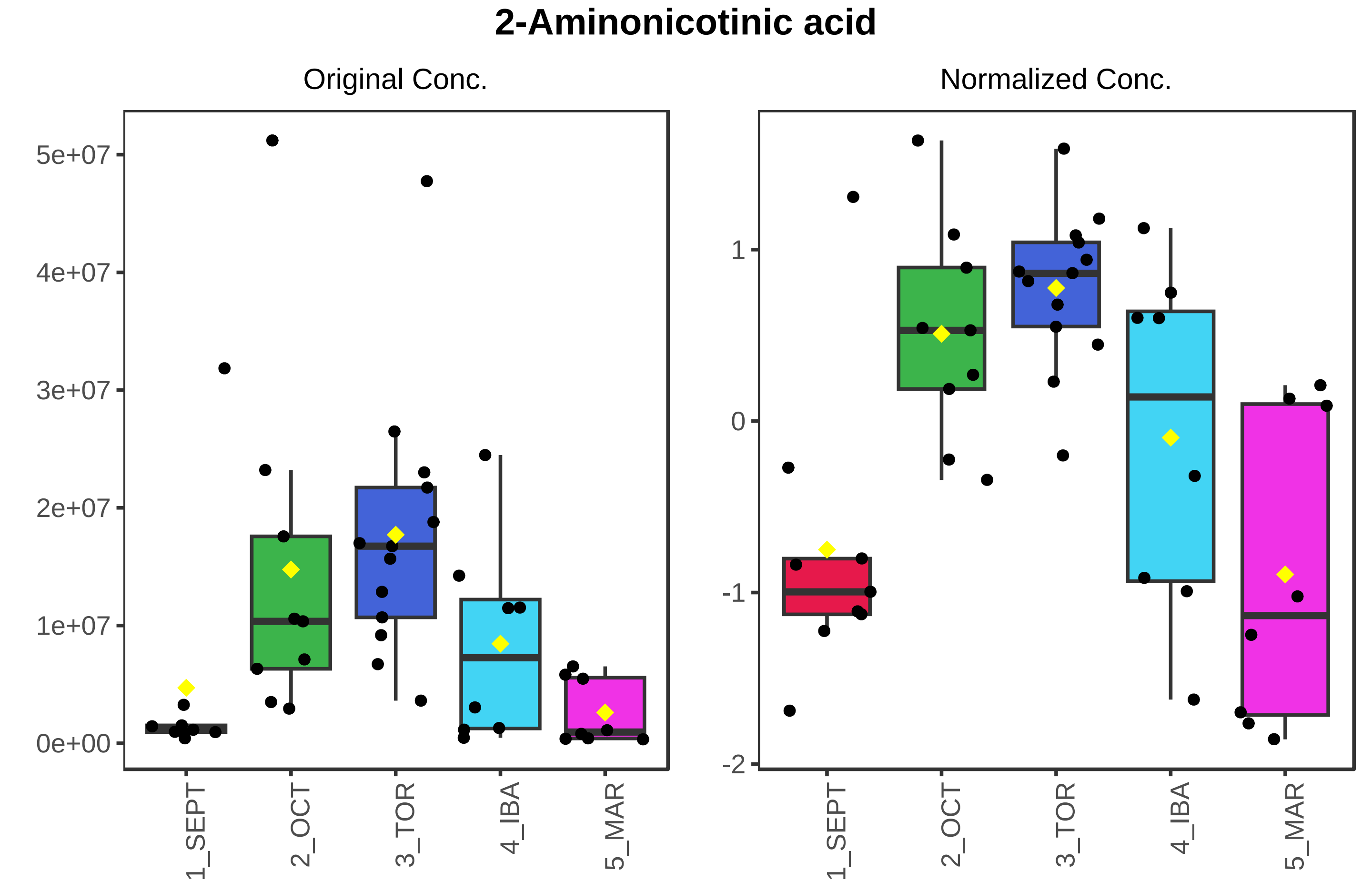

2-Aminonicotinic acid
Original Conc.
Normalized Conc.
5e+07
4e+07
3e+07
2e+07
1e+07
0e+00
1
0
-1
-2
1_SEPT
2_OCT
3_TOR
4_IBA
5_MAR

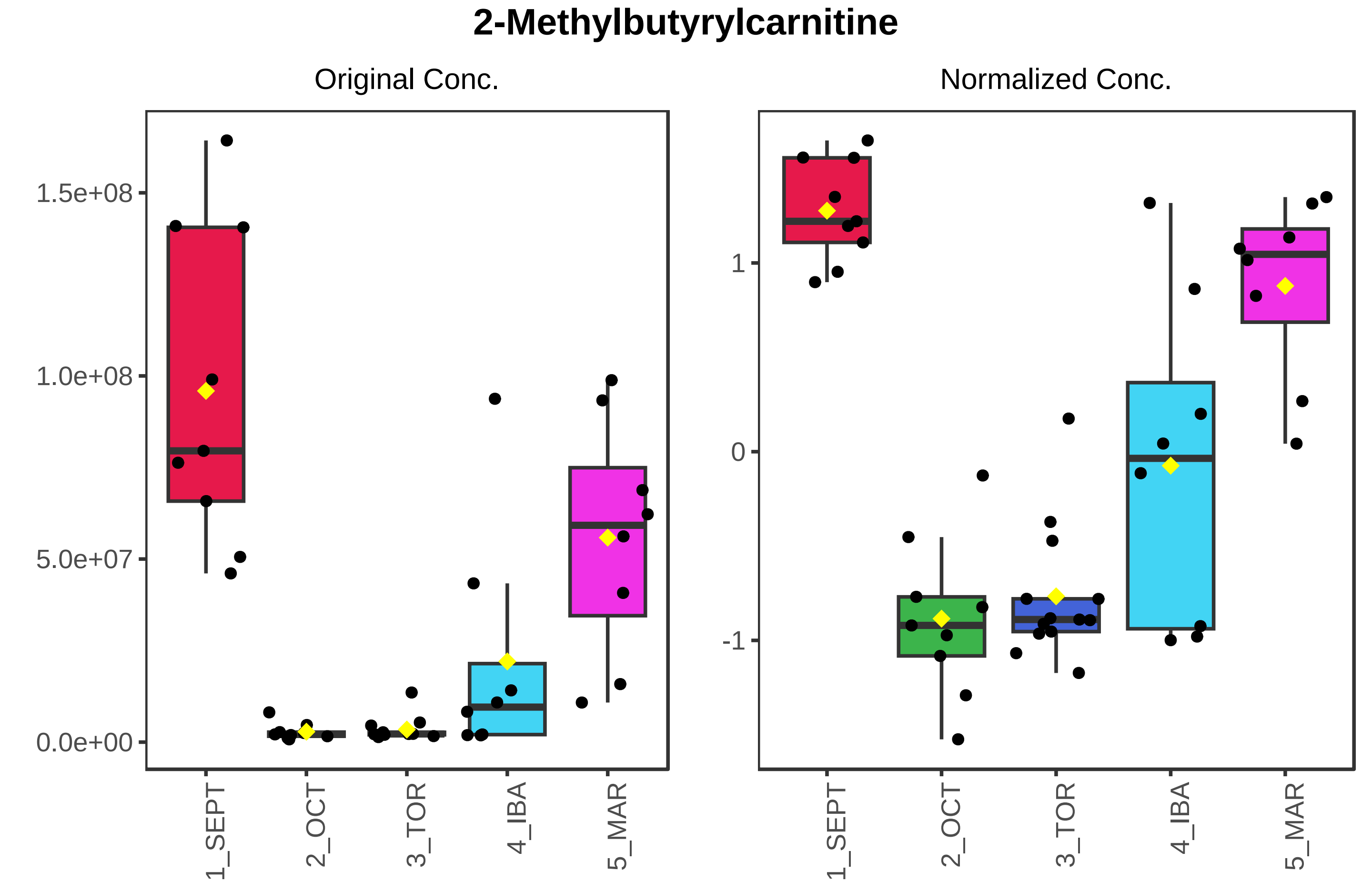

2-Methylbutyrylcarnitine
Original Conc.
Normalized Conc.
1.5e+08
1.0e+08
5.0e+07
0.0e+00
1
0
-1
1_SEPT
2_OCT
3_TOR
4_IBA
5_MAR

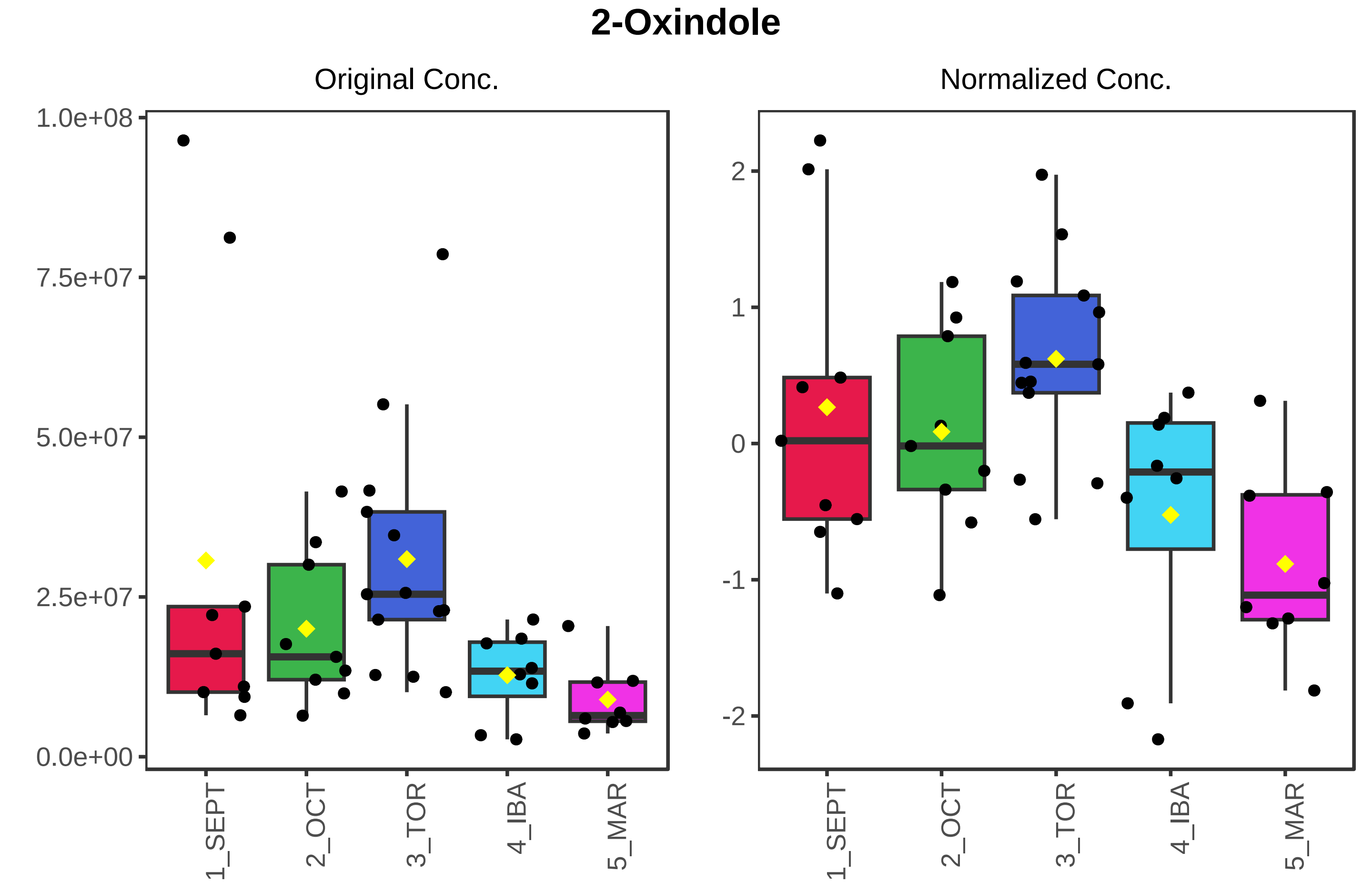

2-Oxindole
Original Conc.
Normalized Conc.
1.0e+08
7.5e+07
5.0e+07
2.5e+07
0.0e+00
2
1
0
-1
-2
1_SEPT
2_OCT
3_TOR
4_IBA
5_MAR

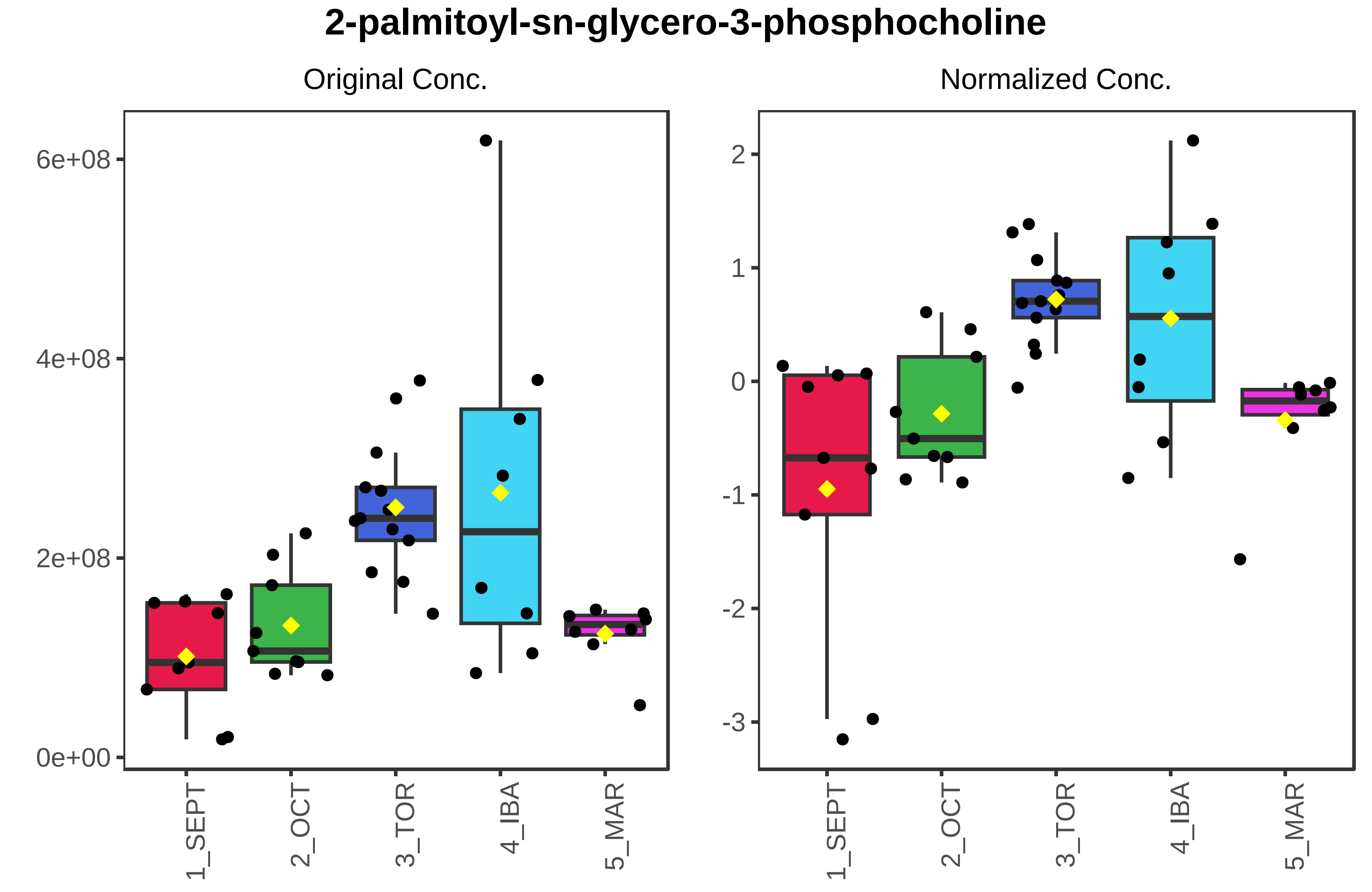

2-palmitoyl-sn-glycero-3-phosphocholine
Original Conc.
Normalized Conc.
6e+08
4e+08
2e+08
0e+00
2
1
0
-1
-2
-3
1_SEPT
2_OCT
3_TOR
4_IBA
5_MAR
1_SEPT
2_OCT
3_TOR
4_IBA
5_MAR

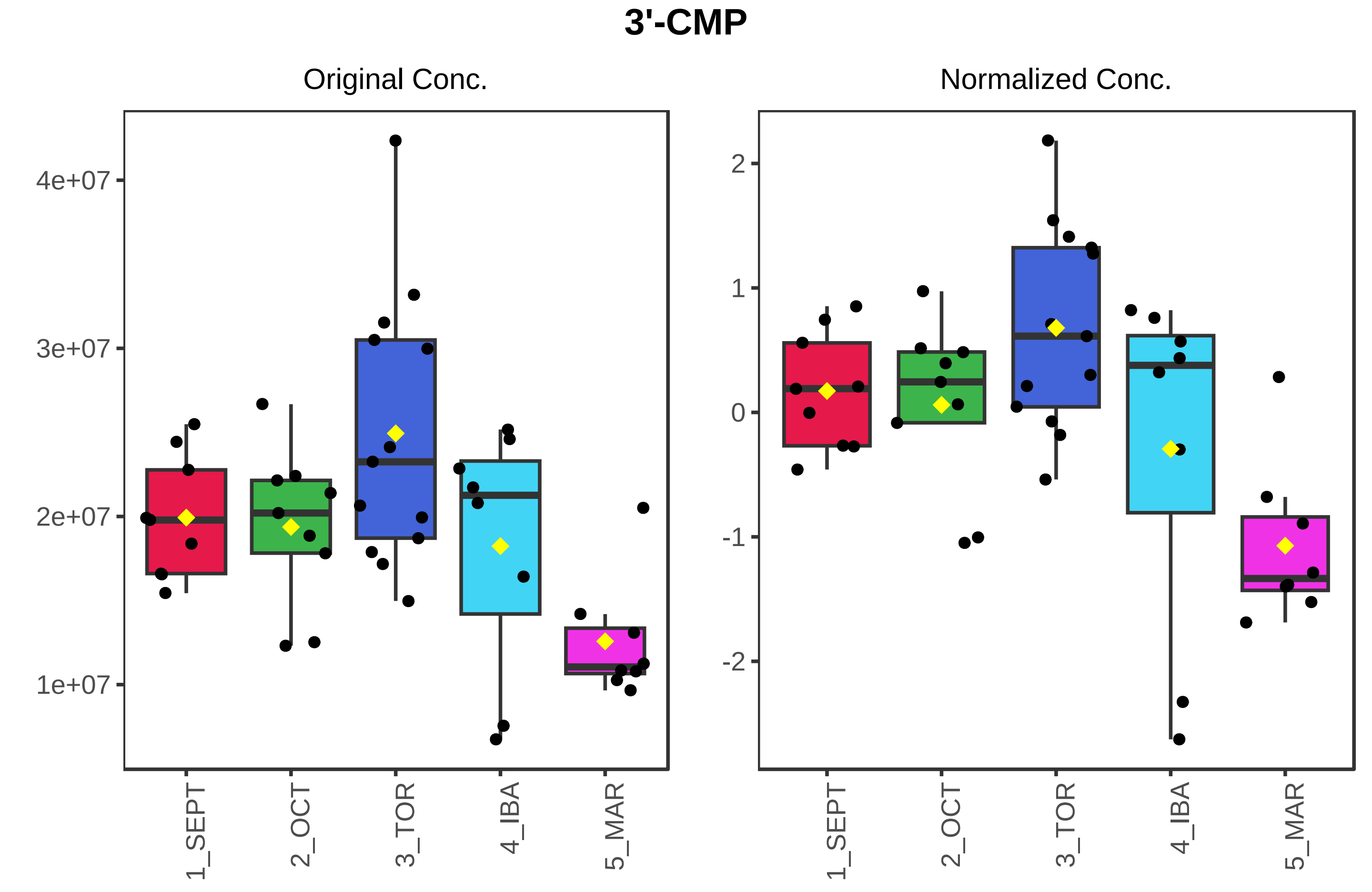
3'-CMP
Original Conc.
Normalized Conc.
4e+07
3e+07
2e+07
1e+07
2
1
0
-1
-2
1_SEPT
2_OCT
3_TOR
4_IBA
5_MAR
1_SEPT
2_OCT
3_TOR
4_IBA
5_MAR

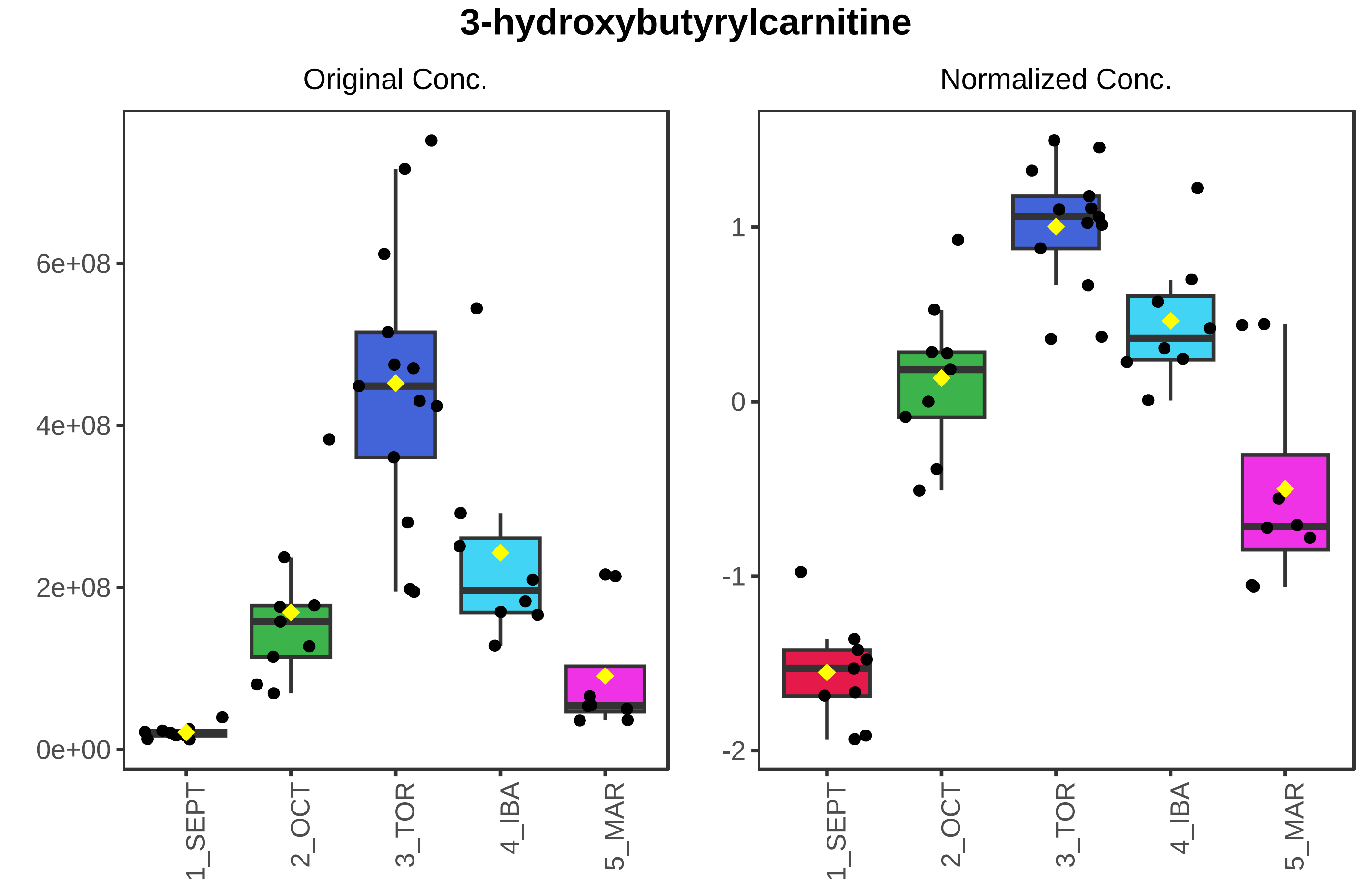
3-hydroxybutyrylcarnitine
Original Conc.
Normalized Conc.
6e+08
4e+08
2e+08
0e+00
1
0
-1
-2
1_SEPT
2_OCT
3_TOR
4_IBA
5_MAR
1_SEPT
2_OCT
3_TOR
4_IBA
5_MAR

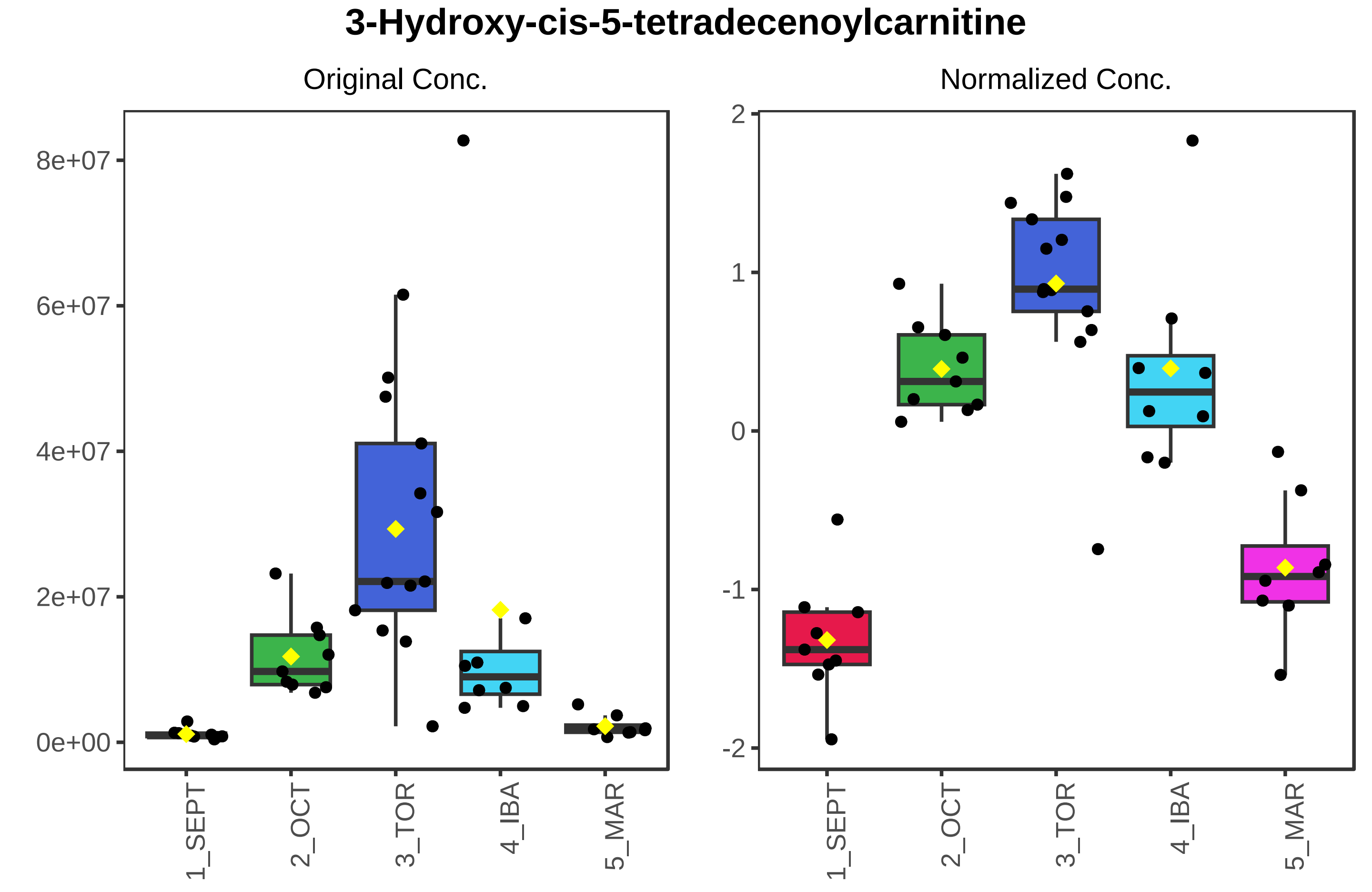

3-Hydroxy-cis-5-tetradecenoylcarnitine
Original Conc.
Normalized Conc.
8e+07
6e+07
4e+07
2e+07
0e+00
2
1
0
-1
-2
1_SEPT
2_OCT
3_TOR
4_IBA
5_MAR

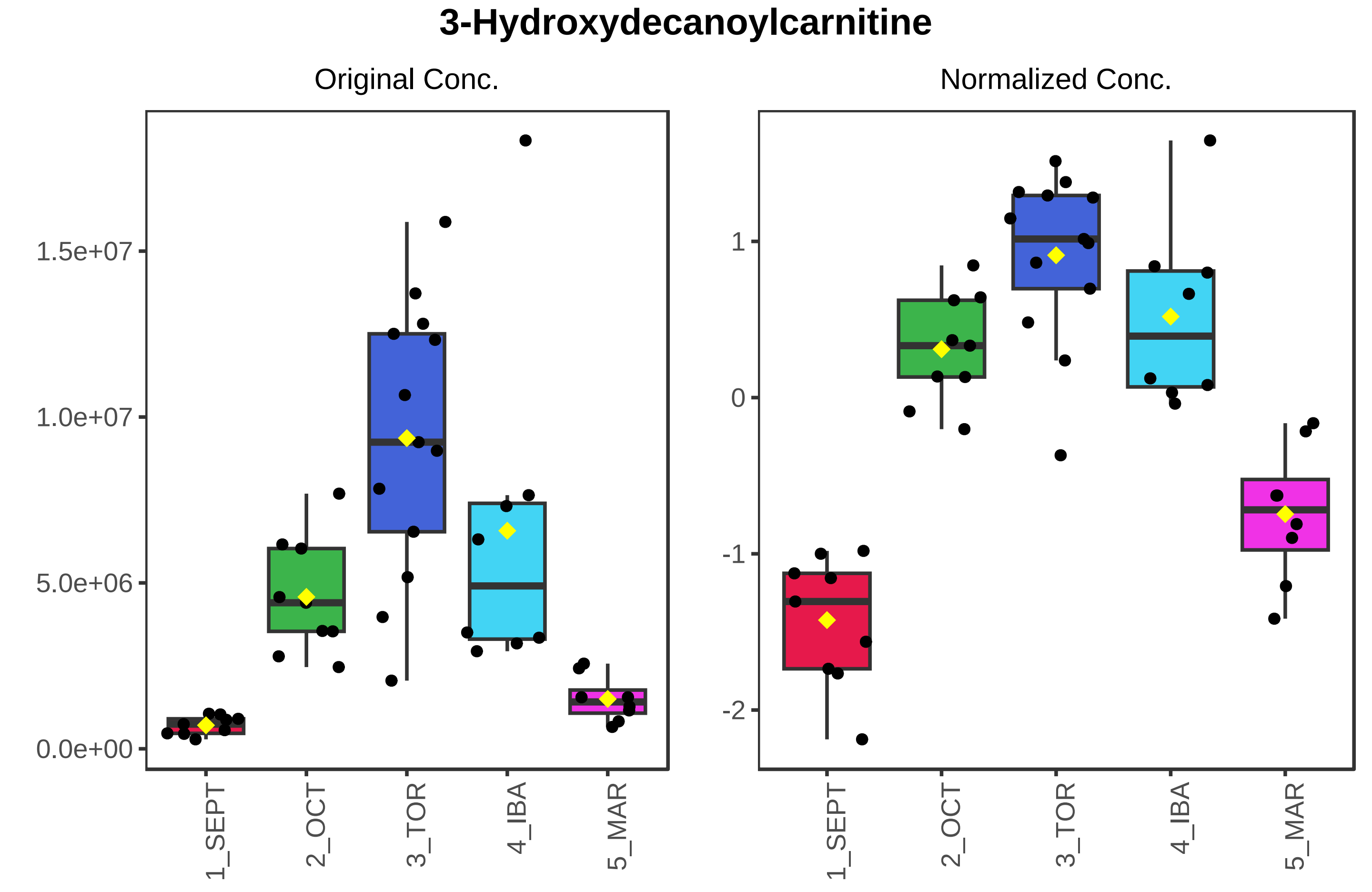

3-Hydroxydecanoylcarnitine
Original Conc.
Normalized Conc.
1.5e+07
1.0e+07
5.0e+06
0.0e+00
1
0
-1
-2
1_SEPT
2_OCT
3_TOR
4_IBA
5_MAR

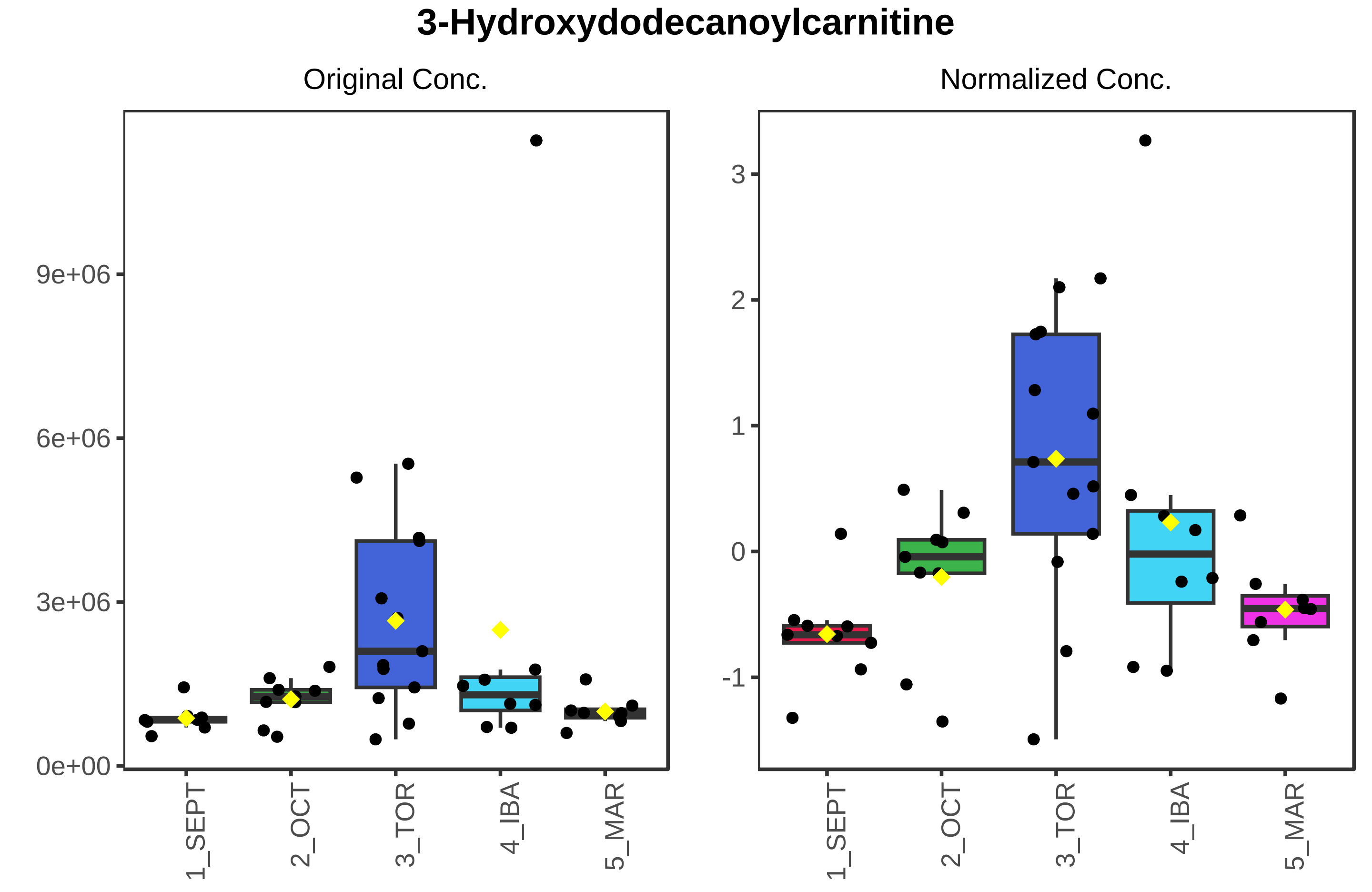
3-Hydroxydodecanoylcarnitine
Original Conc.
Normalized Conc.
9e+06
6e+06
3e+06
0e+00
3
2
1
0
-1
1_SEPT
2_OCT
3_TOR
4_IBA
5_MAR

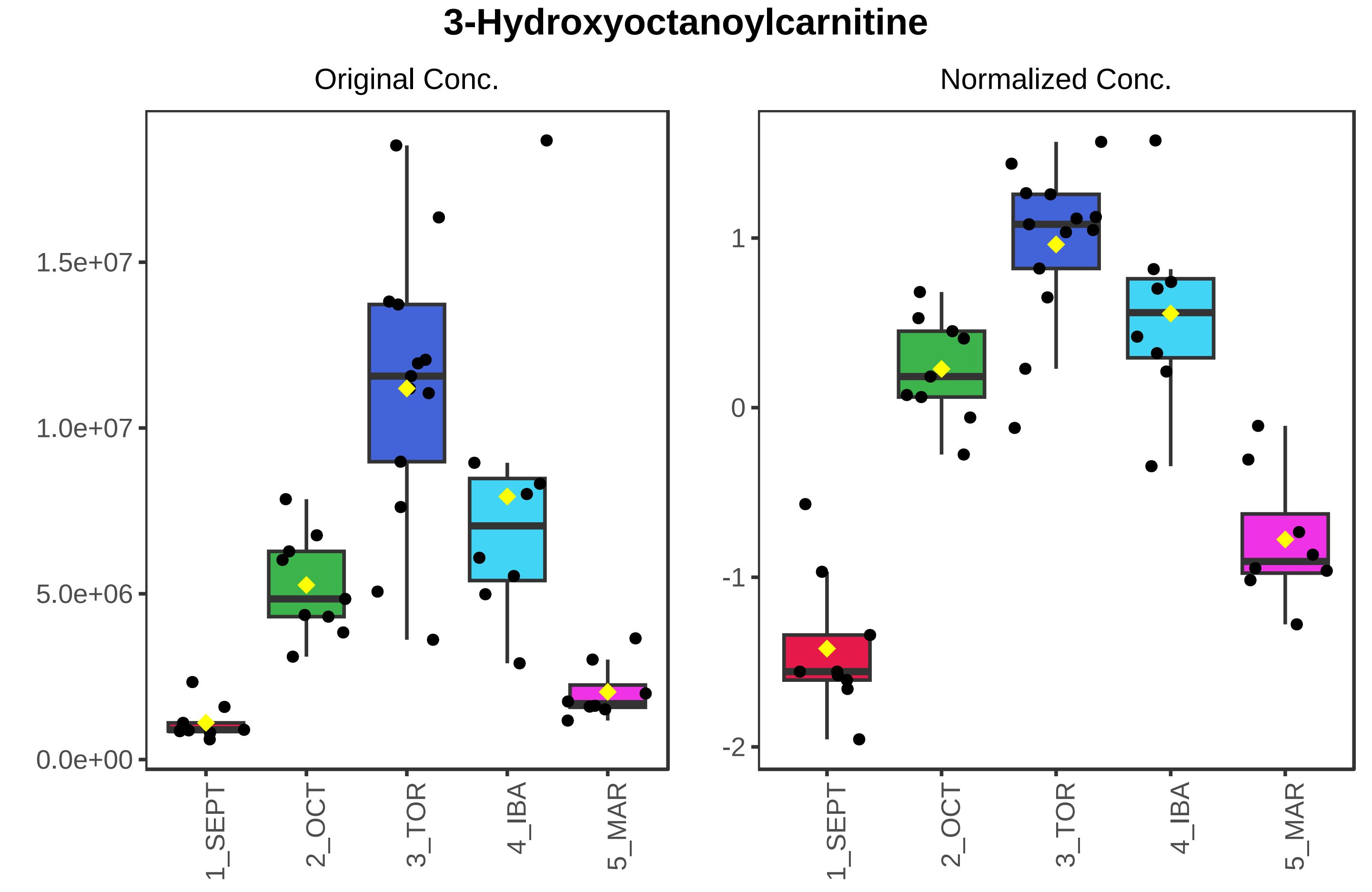

3-Hydroxyoctanoylcarnitine
Original Conc.
Normalized Conc.
1.5e+07
1.0e+07
5.0e+06
0.0e+00
1
0
-1
-2
1_SEPT
2_OCT
3_TOR
4_IBA
5_MAR

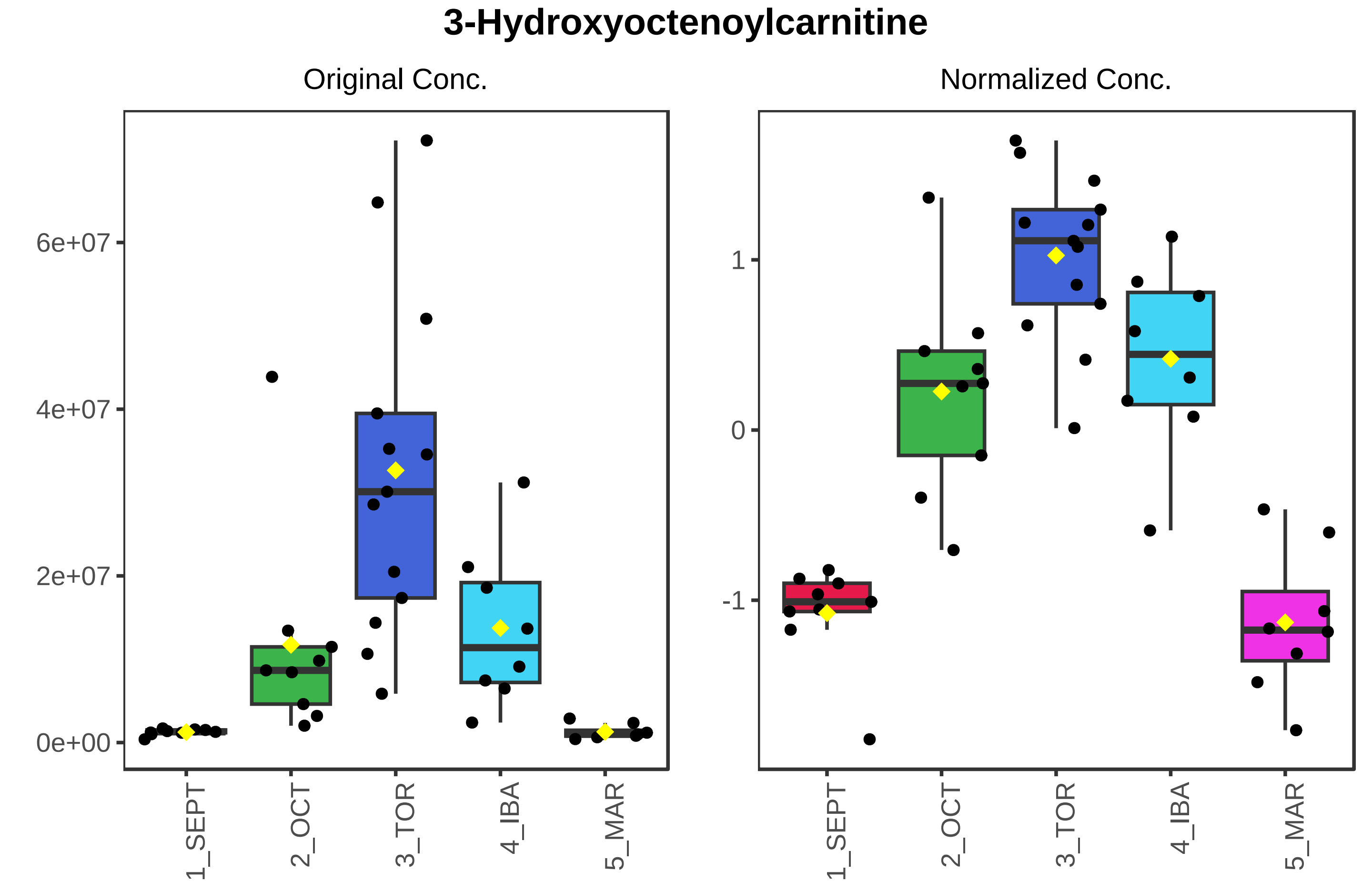

3-Hydroxyoctenoylcarnitine
Original Conc.
Normalized Conc.
6e+07
4e+07
2e+07
0e+00
1
0
-1
1_SEPT
2_OCT
3_TOR
4_IBA
5_MAR

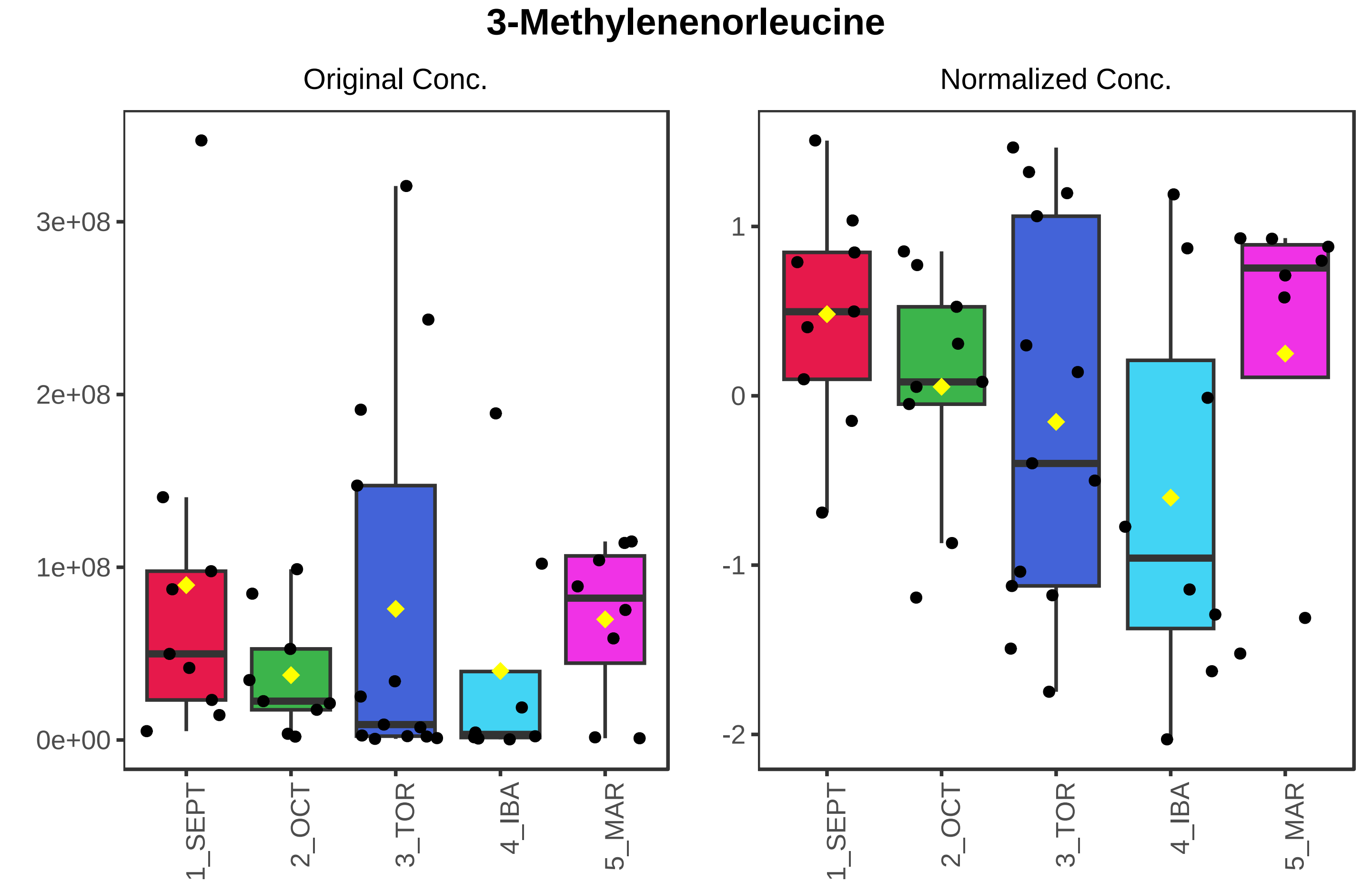
3-Methylenenorleucine
Original Conc.
Normalized Conc.
3e+08
2e+08
1e+08
0e+00
1
0
-1
-2
1_SEPT
2_OCT
3_TOR
4_IBA
5_MAR

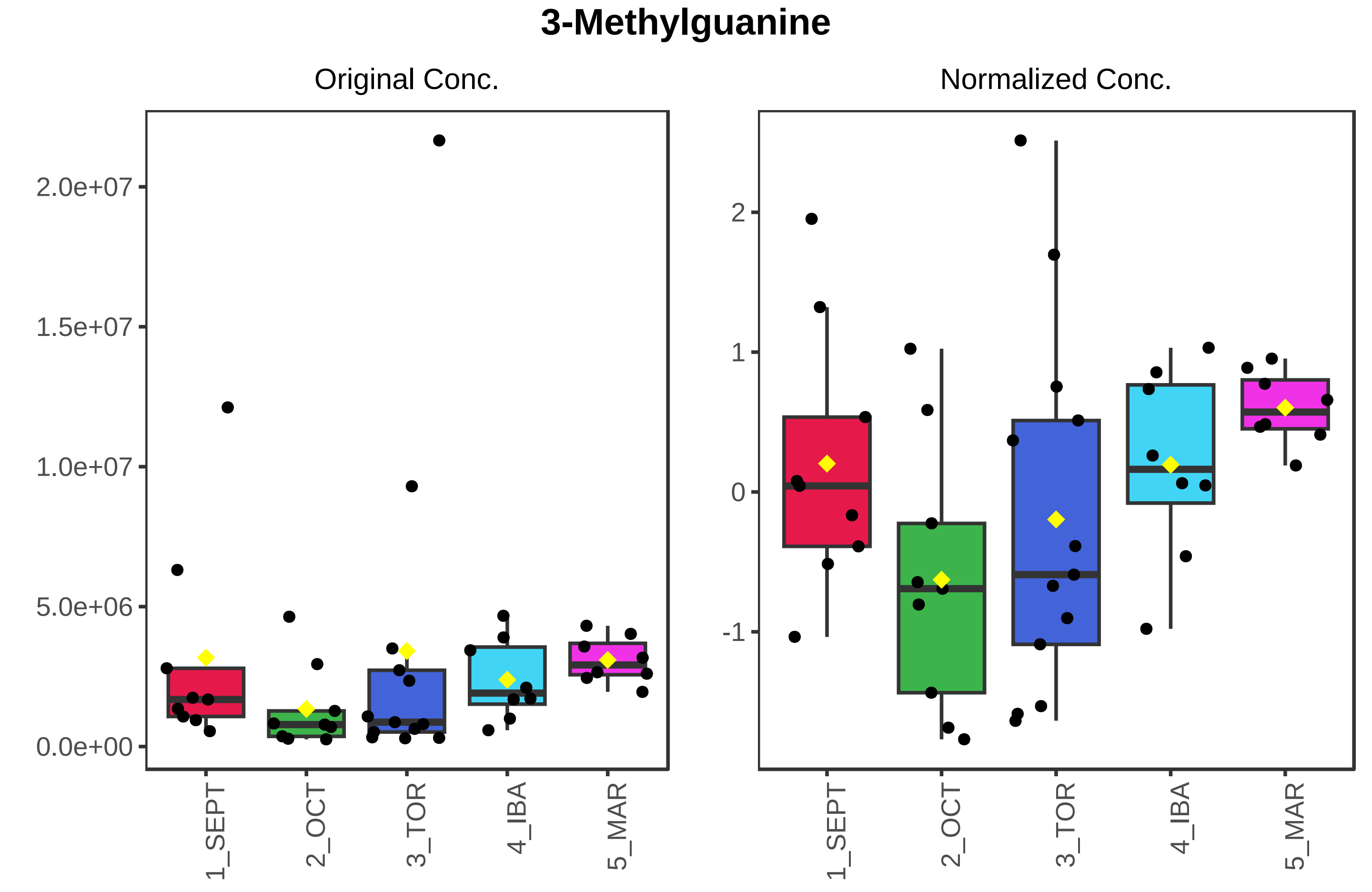
3-Methylguanine
Original Conc.
Normalized Conc.
2.0e+07
1.5e+07
1.0e+07
5.0e+06
0.0e+00
2
1
0
-1
1_SEPT
2_OCT
3_TOR
4_IBA
5_MAR
1_SEPT
2_OCT
3_TOR
4_IBA
5_MAR

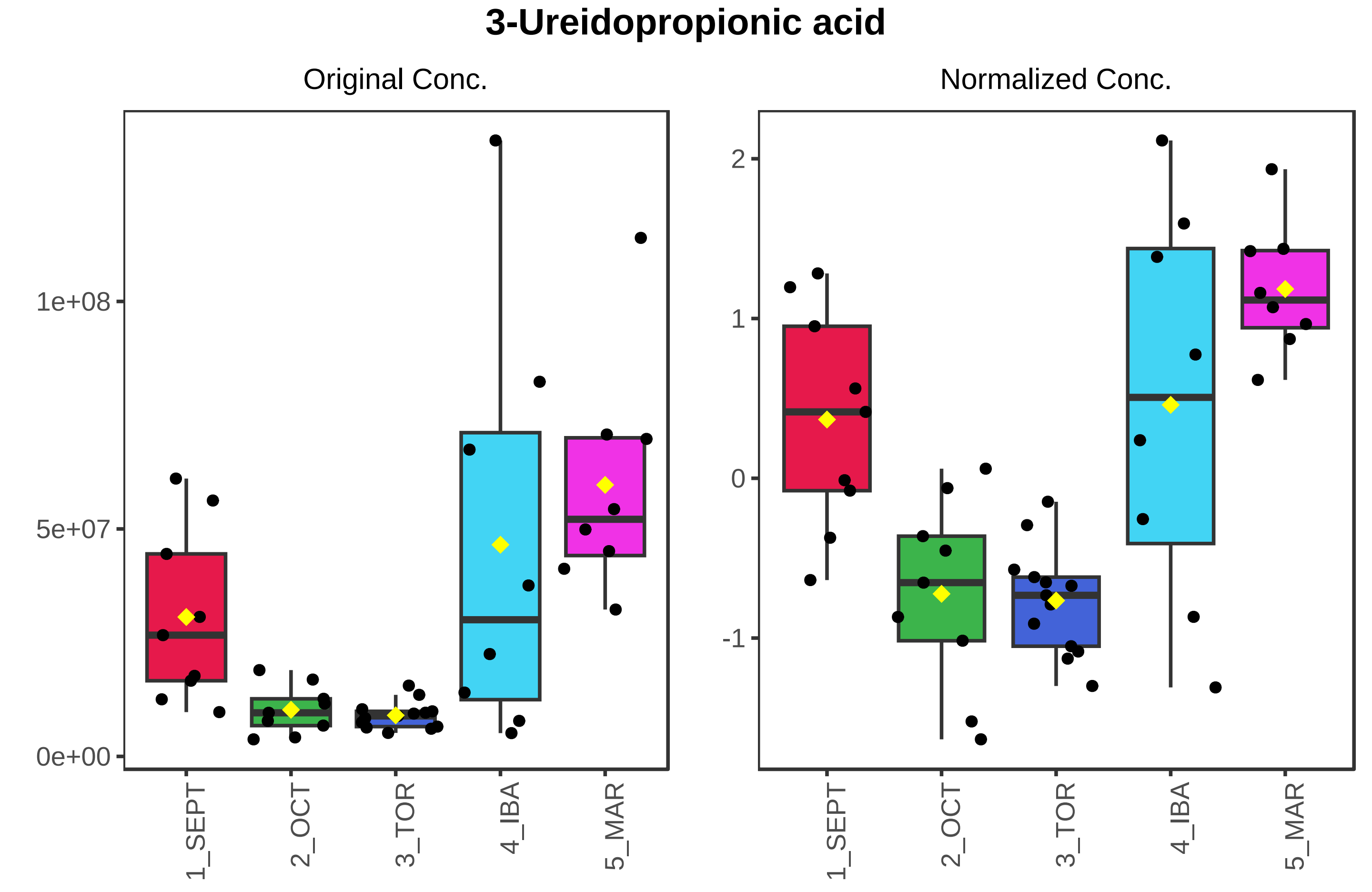

3-Ureidopropionic acid
Original Conc.
Normalized Conc.
1e+08
5e+07
0e+00
2
1
0
-1
1_SEPT
2_OCT
3_TOR
4_IBA
5_MAR

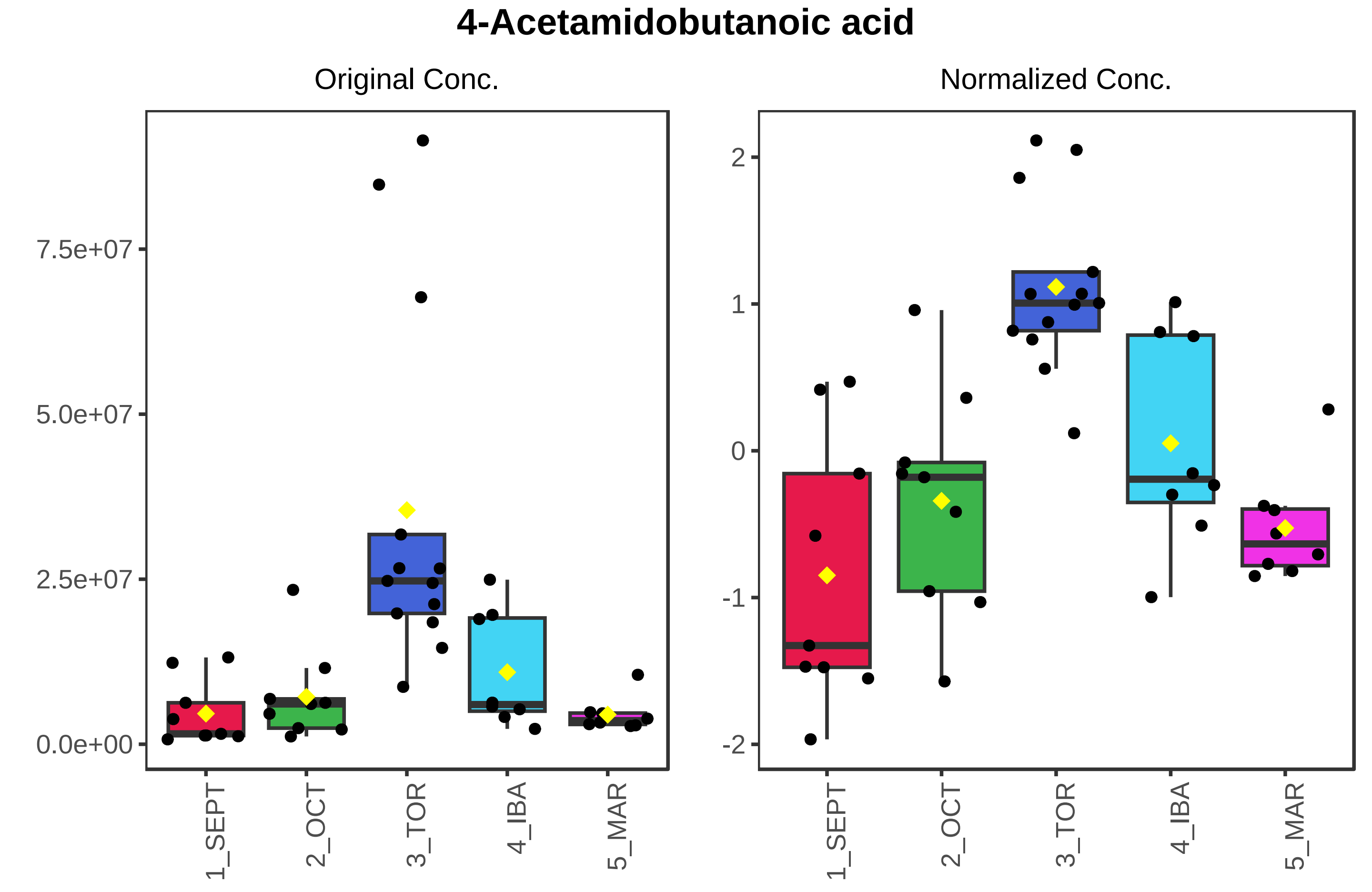

4-Acetamidobutanoic acid
Original Conc.
Normalized Conc.
7.5e+07
5.0e+07
2.5e+07
0.0e+00
2
1
0
-1
-2
1_SEPT
2_OCT
3_TOR
4_IBA
5_MAR

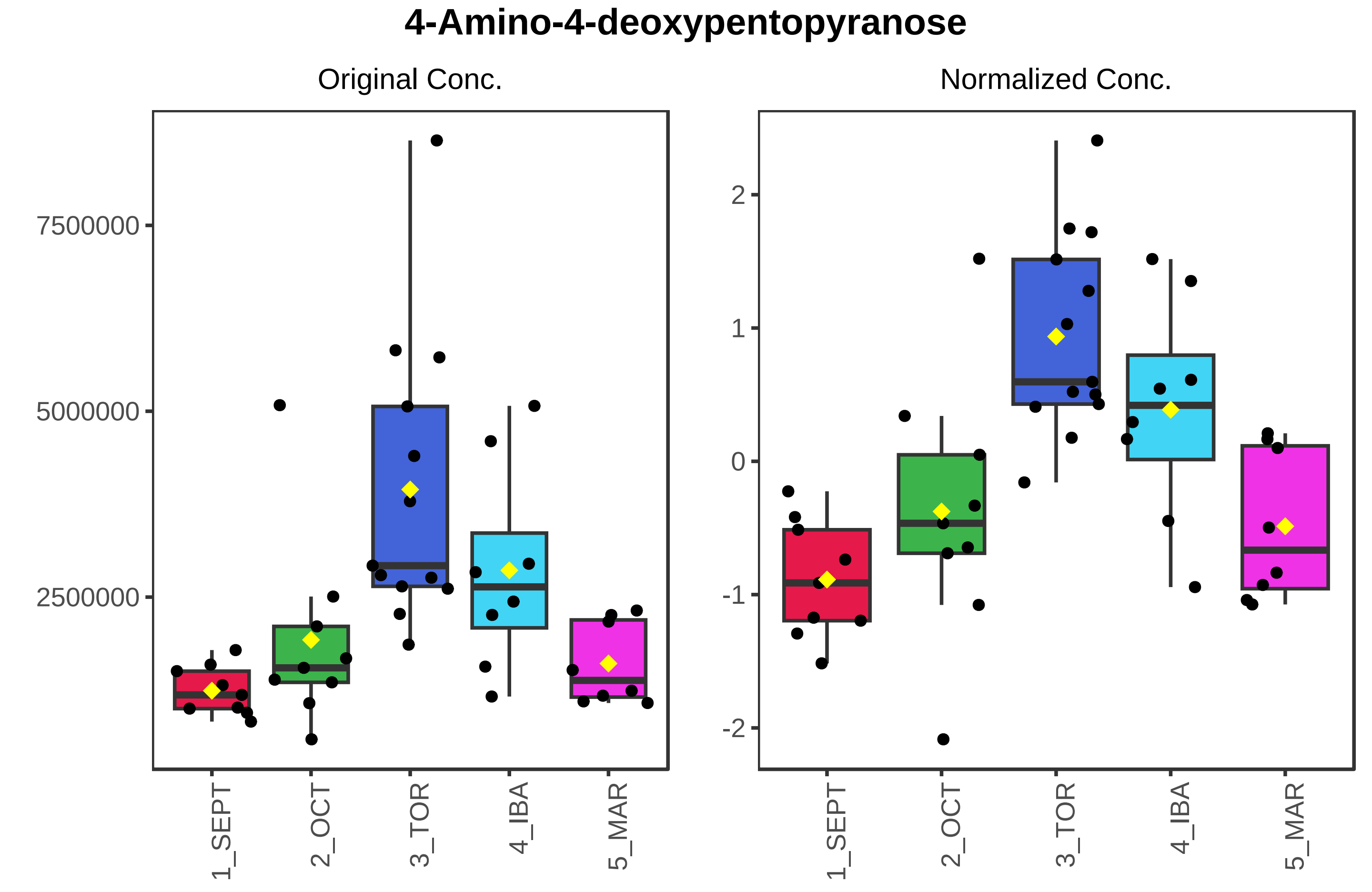
4-Amino-4-deoxypentopyranose
Original Conc.
Normalized Conc.
7500000
5000000
2500000
2
1
0
-1
-2
1_SEPT
2_OCT
3_TOR
4_IBA
5_MAR

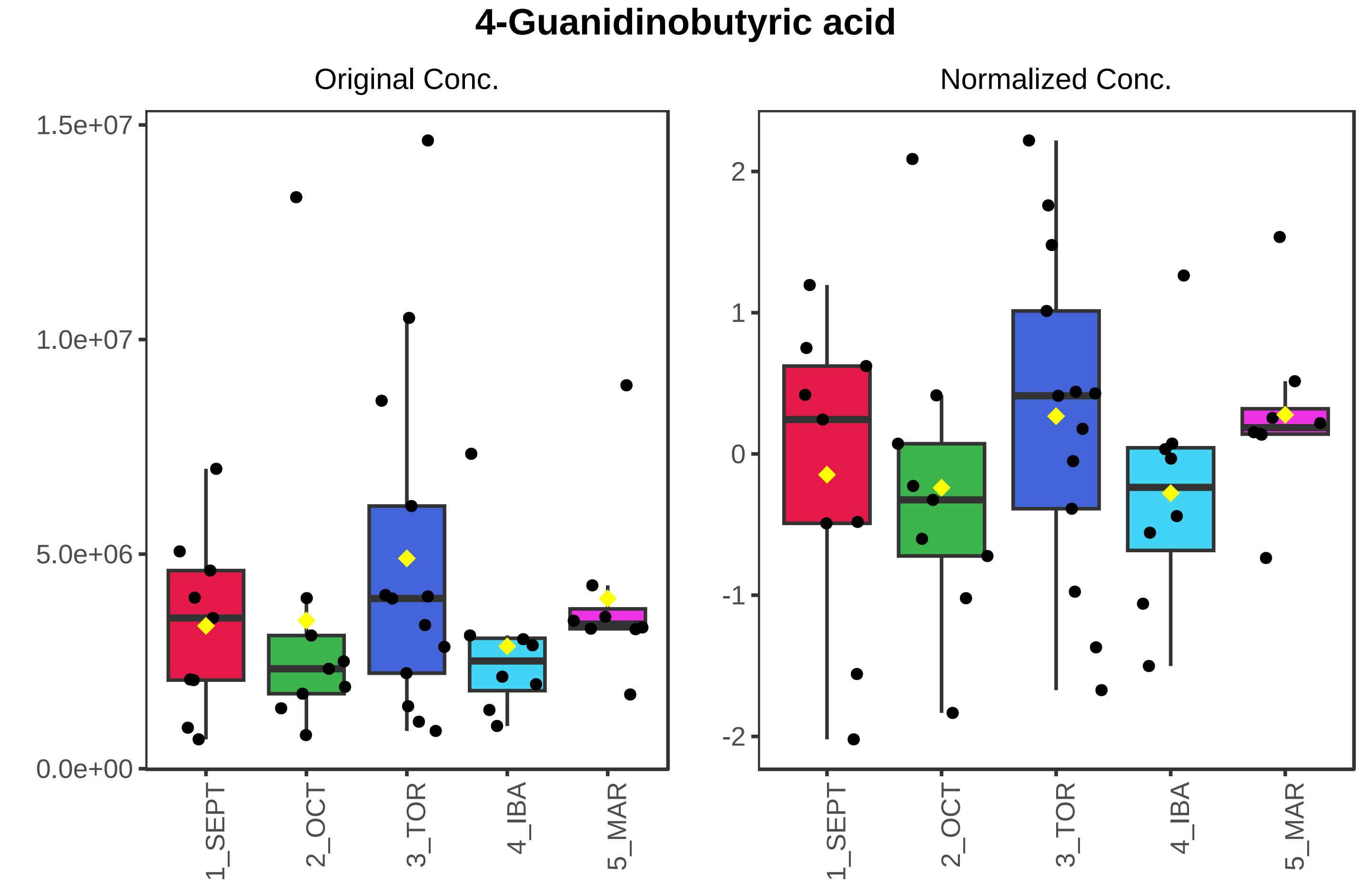

4-Guanidinobutyric acid
Original Conc.
Normalized Conc.
1.5e+07
1.0e+07
5.0e+06
0.0e+00
2
1
0
-1
-2
1_SEPT
2_OCT
3_TOR
4_IBA
5_MAR

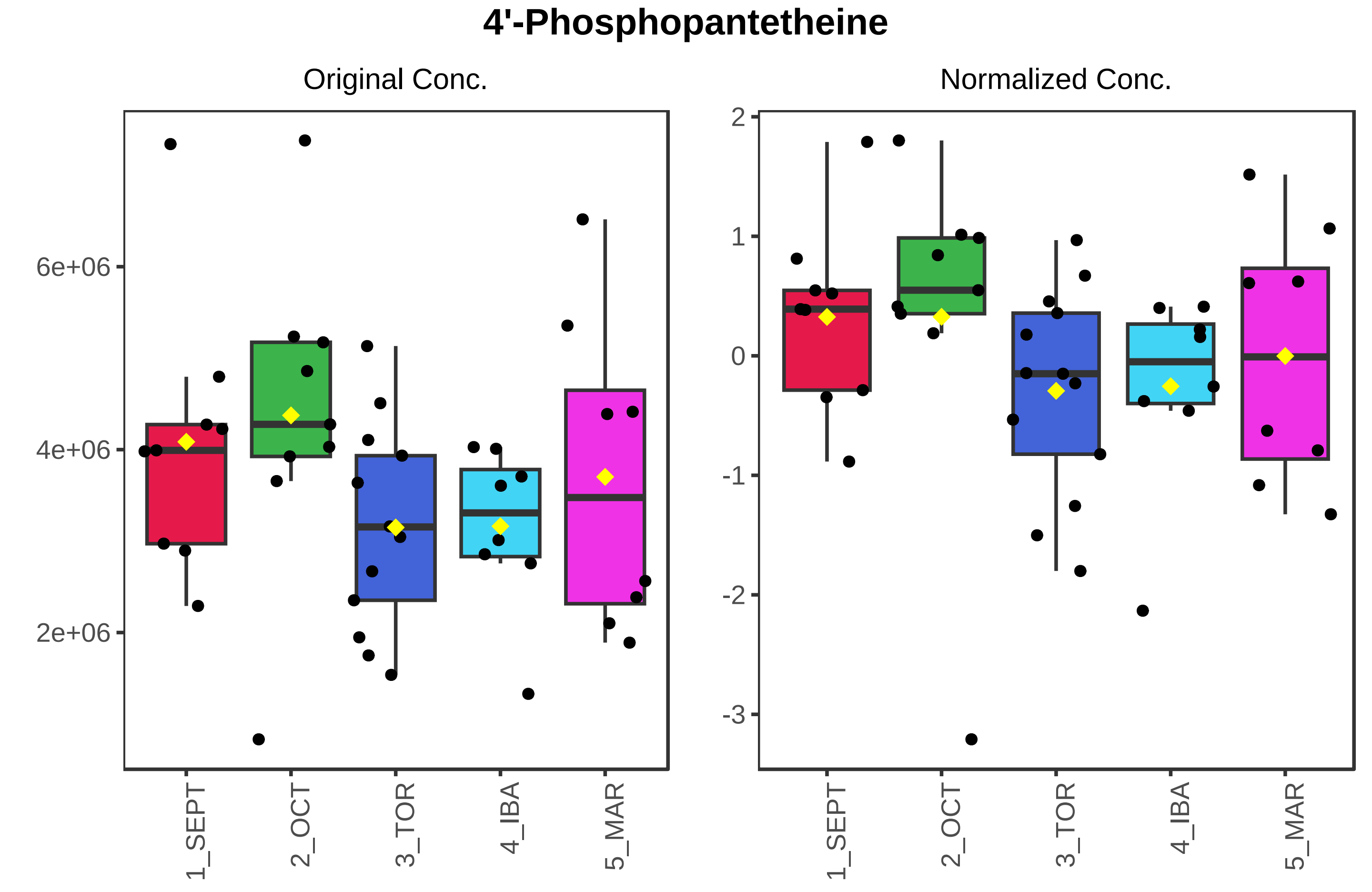
4'-Phosphopantetheine
Original Conc.
Normalized Conc.
6e+06
4e+06
2e+06
2
1
0
-1
-2
-3
1_SEPT
2_OCT
3_TOR
4_IBA
5_MAR

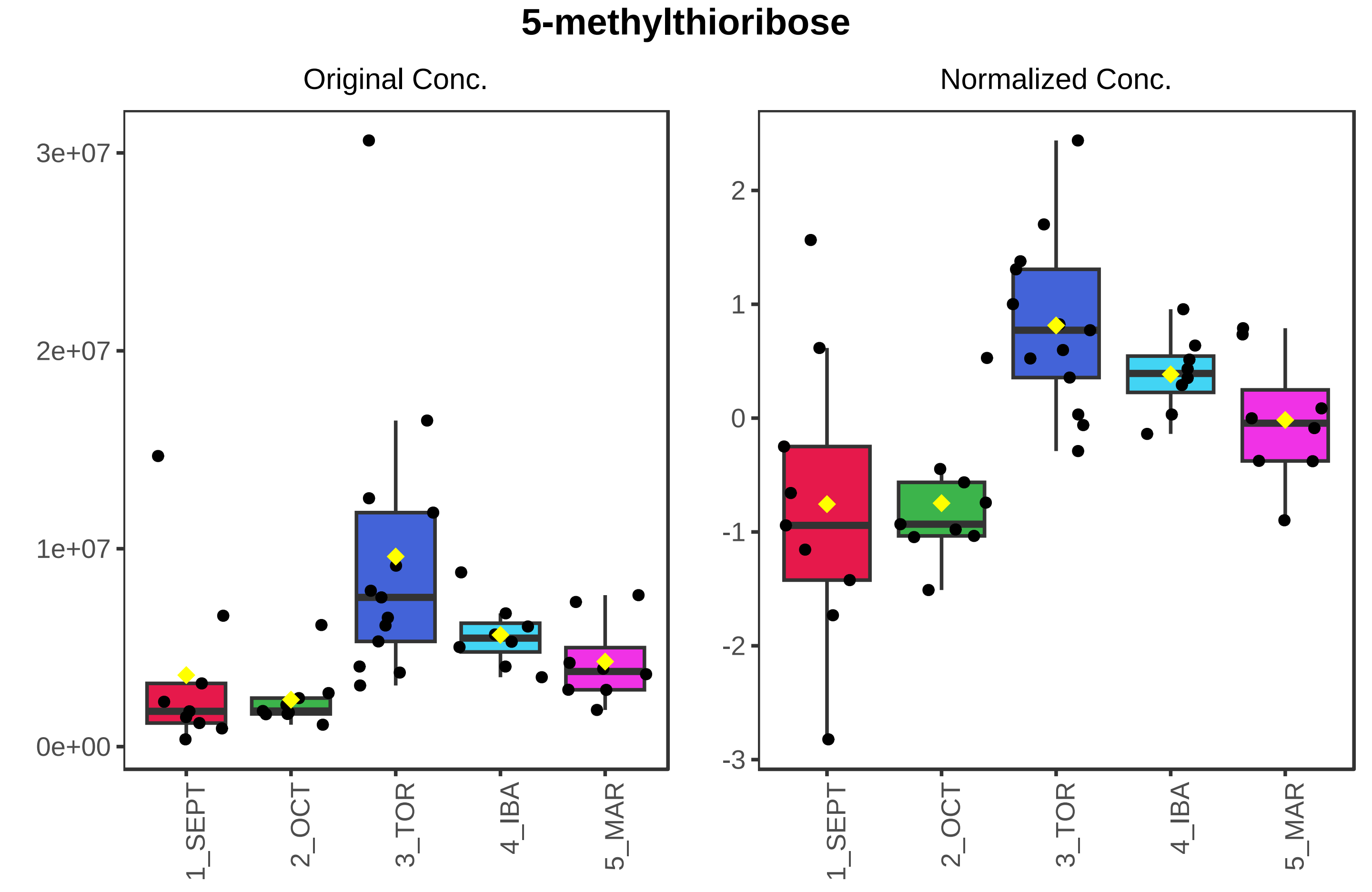
5-methylthioribose
Original Conc.
Normalized Conc.
3e+07
2e+07
1e+07
0e+00
2
1
0
-1
-2
-3
1_SEPT
2_OCT
3_TOR
4_IBA
5_MAR
1_SEPT
2_OCT
3_TOR
4_IBA
5_MAR

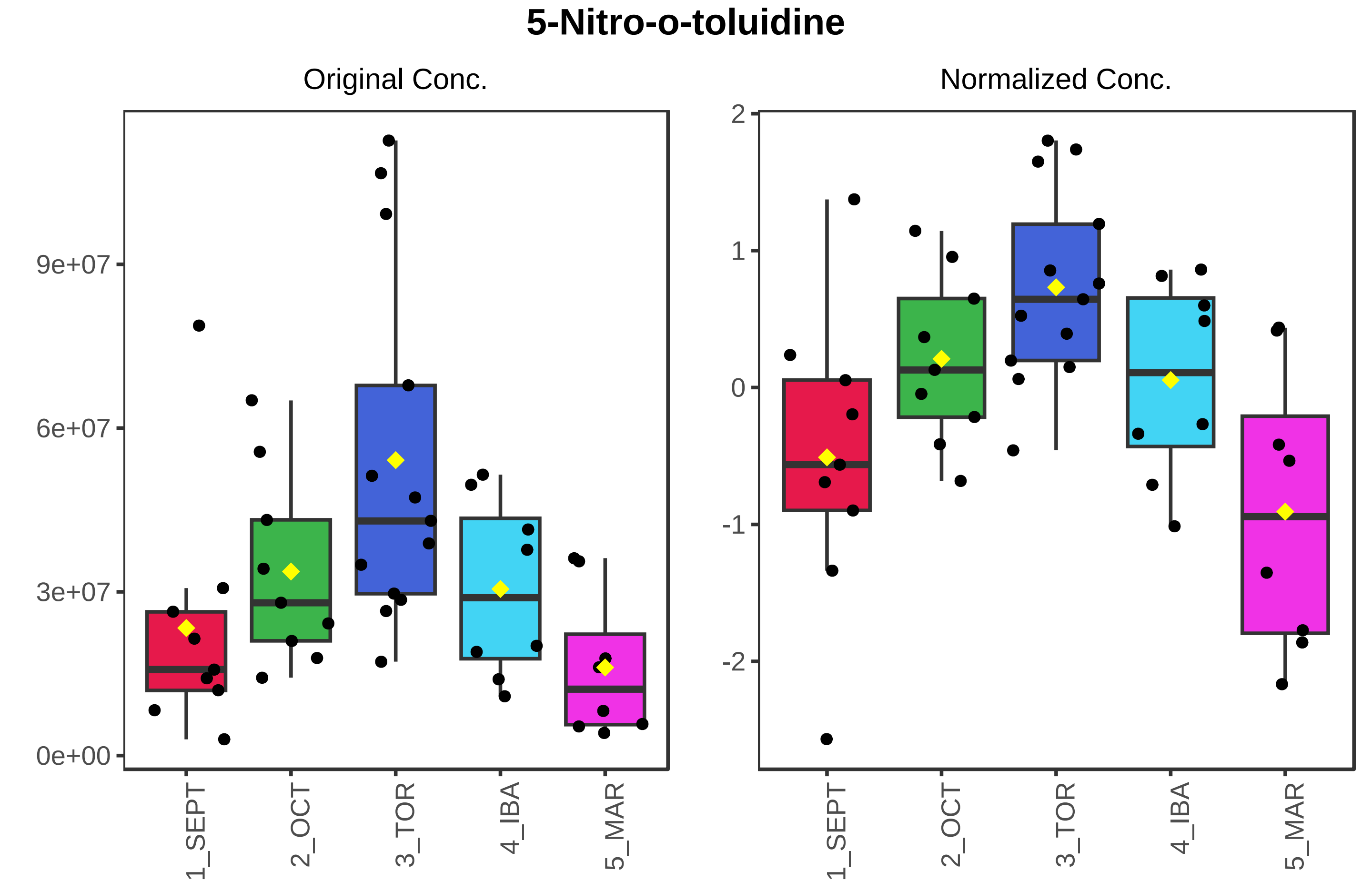

5-Nitro-o-toluidine
Original Conc.
Normalized Conc.
9e+07
6e+07
3e+07
0e+00
2
1
0
-1
-2
1_SEPT
2_OCT
3_TOR
4_IBA
5_MAR

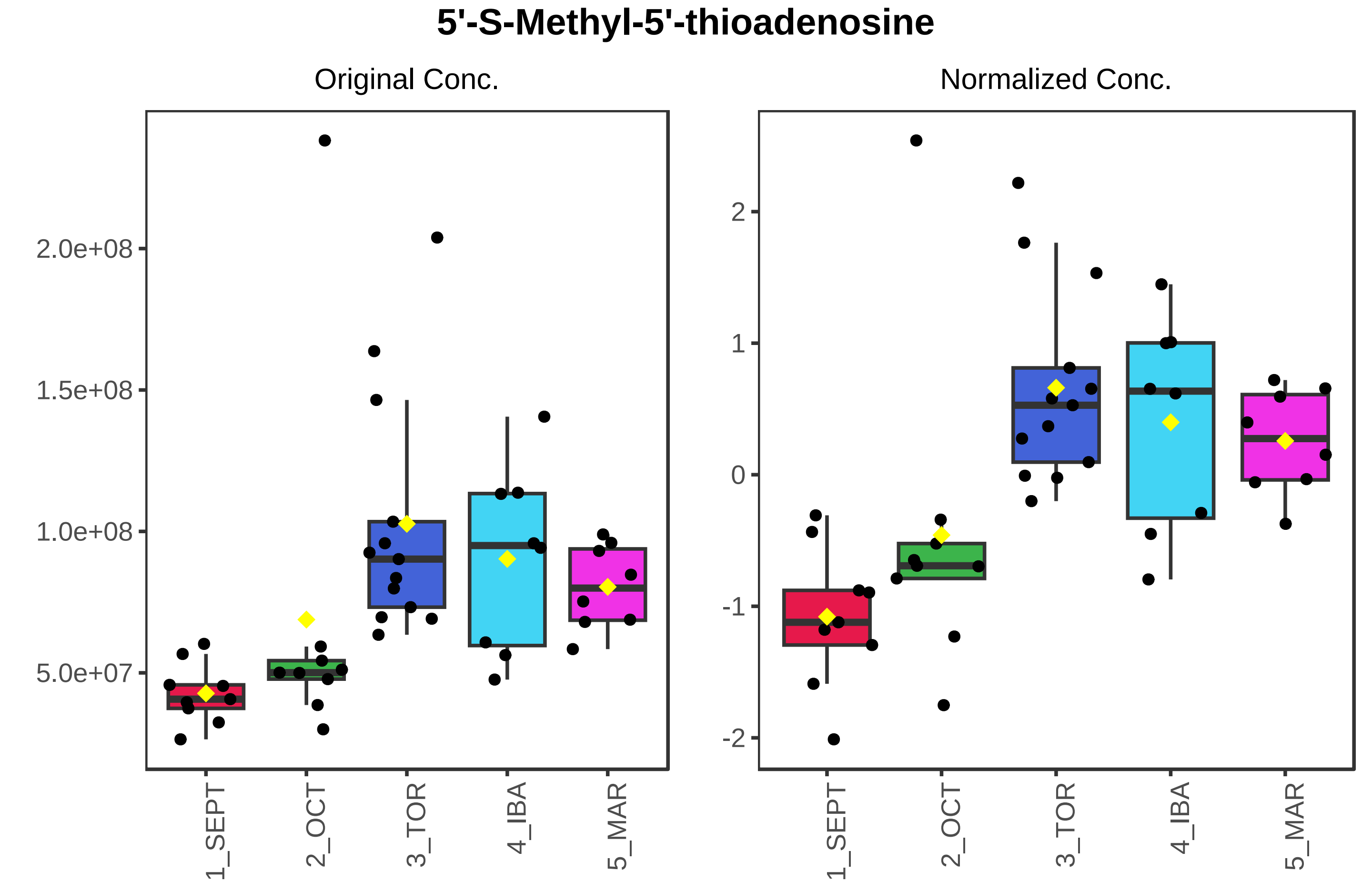

5'-S-Methyl-5'-thioadenosine
Original Conc.
Normalized Conc.
2.0e+08
1.5e+08
1.0e+08
5.0e+07
2
1
0
-1
-2
1_SEPT
2_OCT
3_TOR
4_IBA
5_MAR

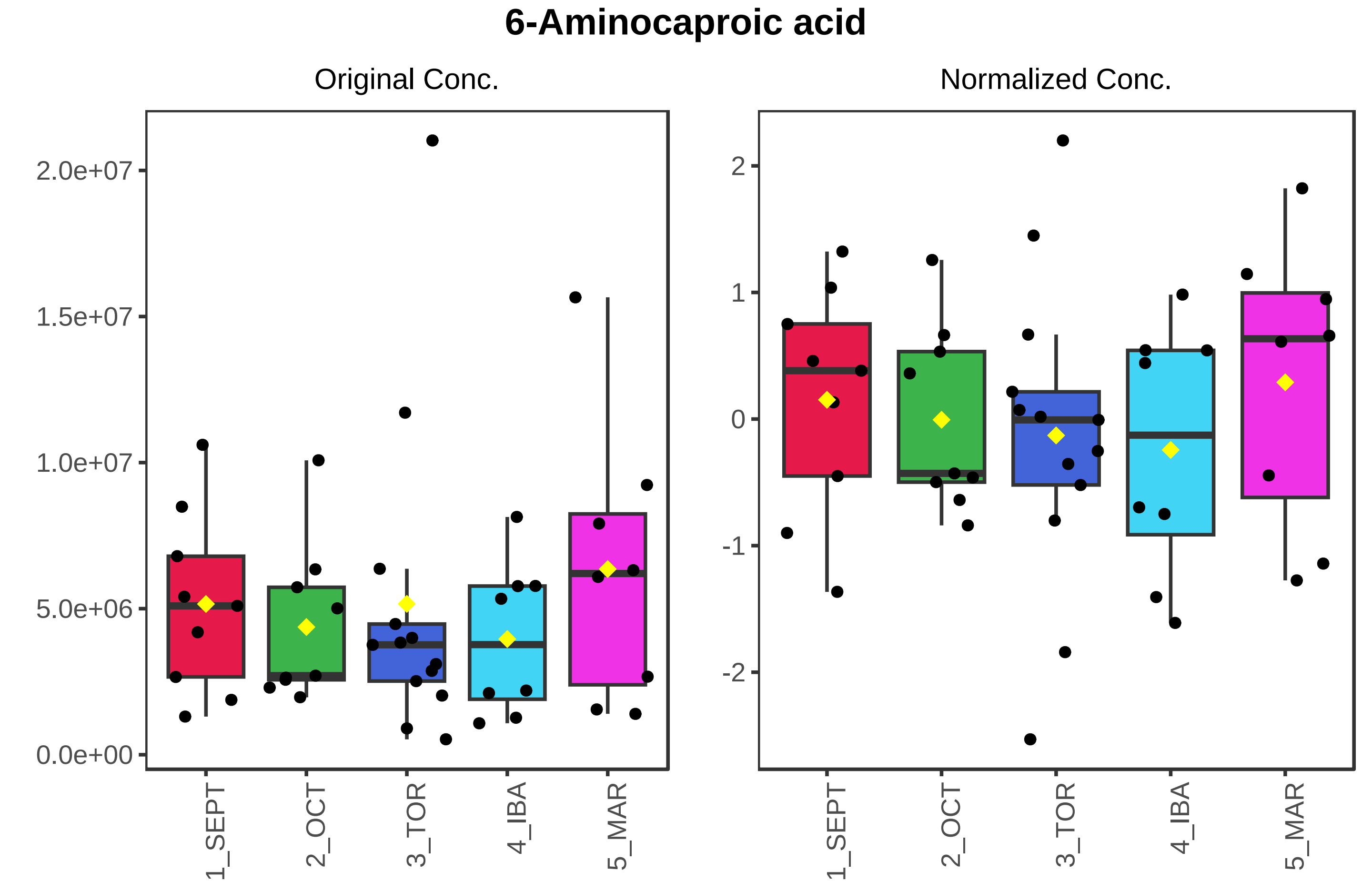

6-Aminocaproic acid
Original Conc.
Normalized Conc.
2.0e+07
1.5e+07
1.0e+07
5.0e+06
0.0e+00
2
1
0
-1
-2
1_SEPT
2_OCT
3_TOR
4_IBA
5_MAR

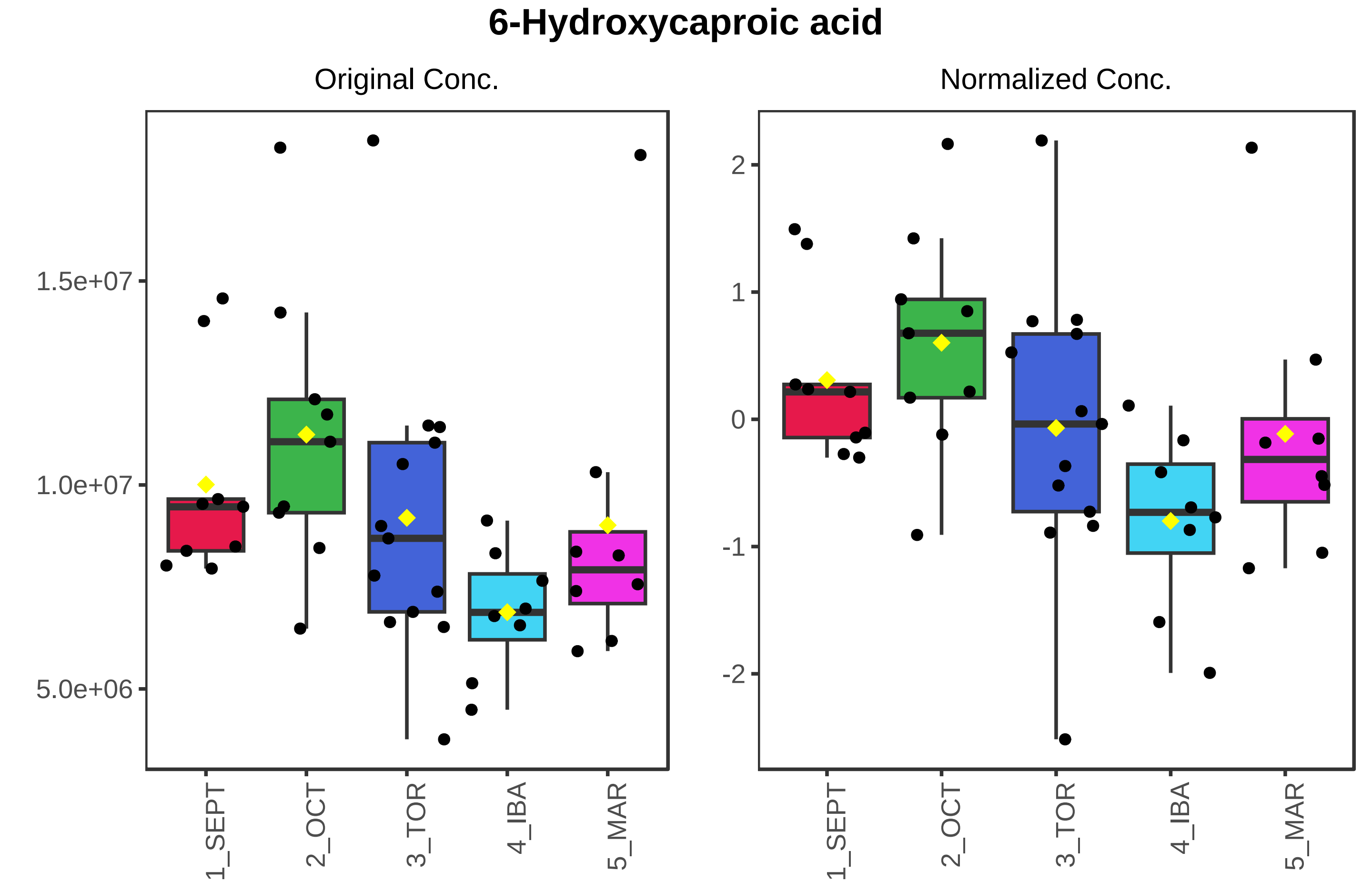

6-Hydroxycaproic acid
Original Conc.
Normalized Conc.
1.5e+07
1.0e+07
5.0e+06
2
1
0
-1
-2
1_SEPT
2_OCT
3_TOR
4_IBA
5_MAR

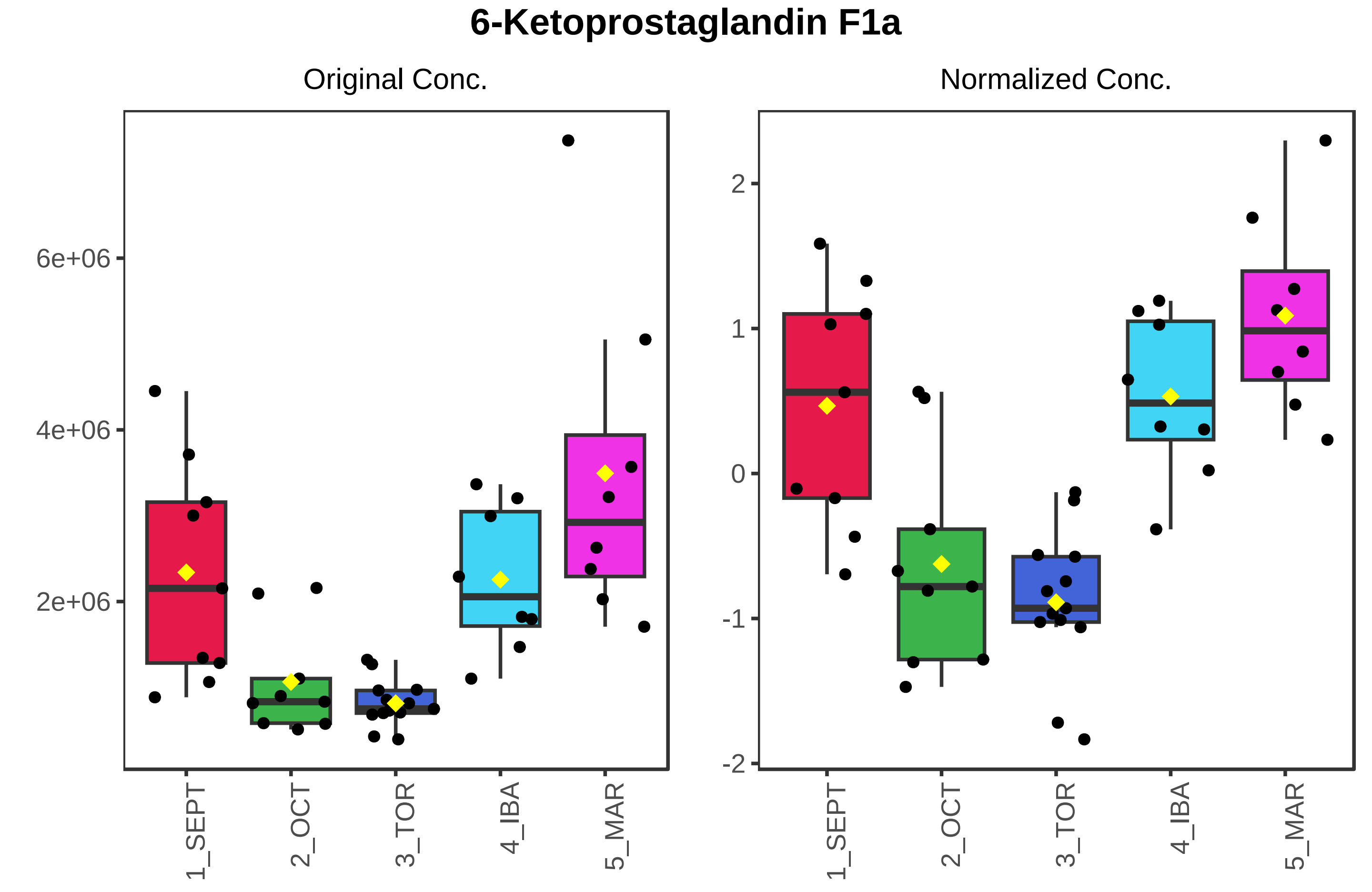

6-Ketoprostaglandin F1a
Original Conc.
Normalized Conc.
6e+06
4e+06
2e+06
2
1
0
-1
-2
1_SEPT
2_OCT
3_TOR
4_IBA
5_MAR
1_SEPT
2_OCT
3_TOR
4_IBA
5_MAR

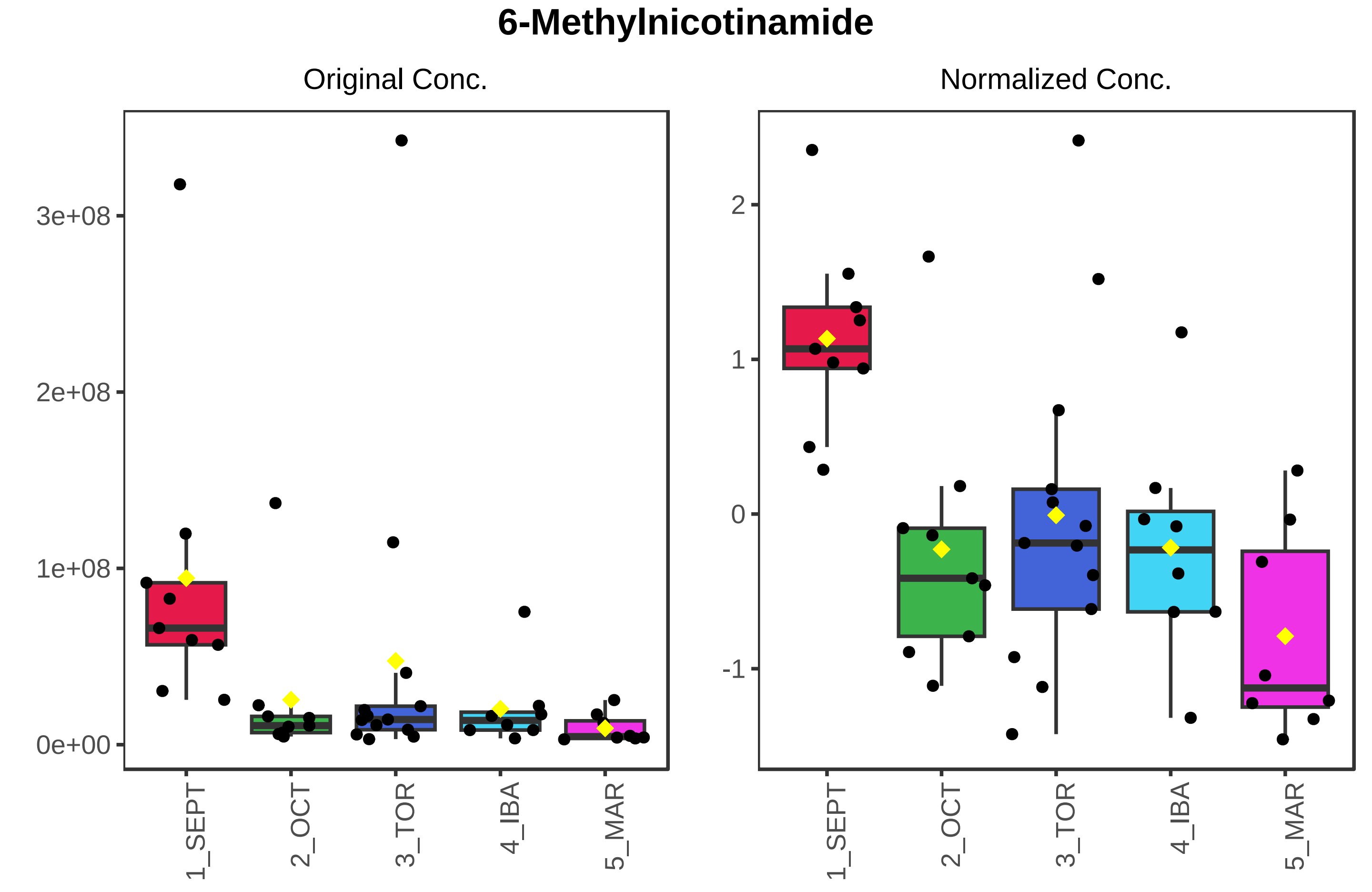
6-Methylnicotinamide
Original Conc.
Normalized Conc.
3e+08
2e+08
1e+08
0e+00
2
1
0
-1
1_SEPT
2_OCT
3_TOR
4_IBA
5_MAR

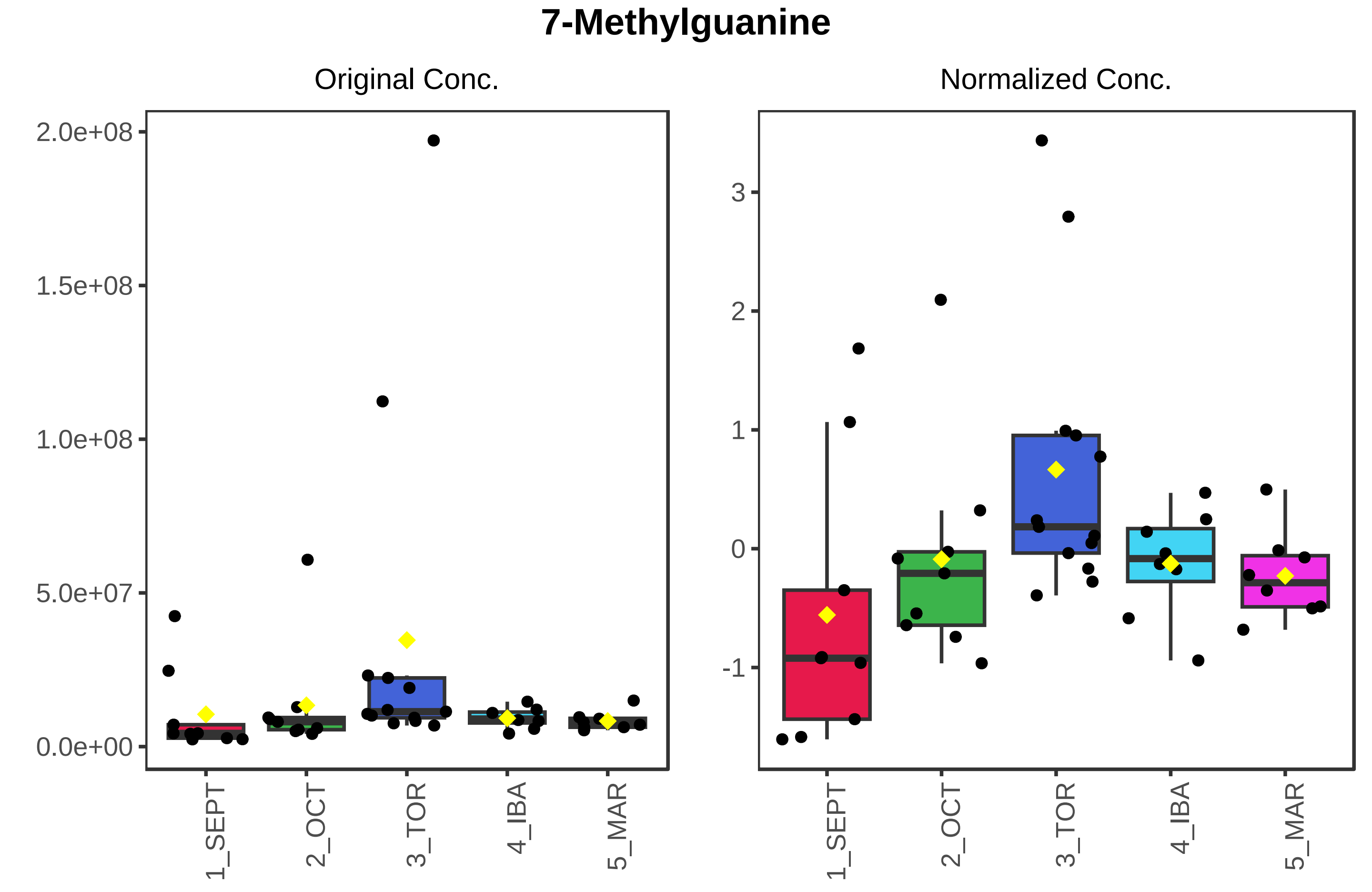

7-Methylguanine
Original Conc.
Normalized Conc.
2.0e+08
1.5e+08
1.0e+08
5.0e+07
0.0e+00
3
2
1
0
-1
1_SEPT
2_OCT
3_TOR
4_IBA
5_MAR

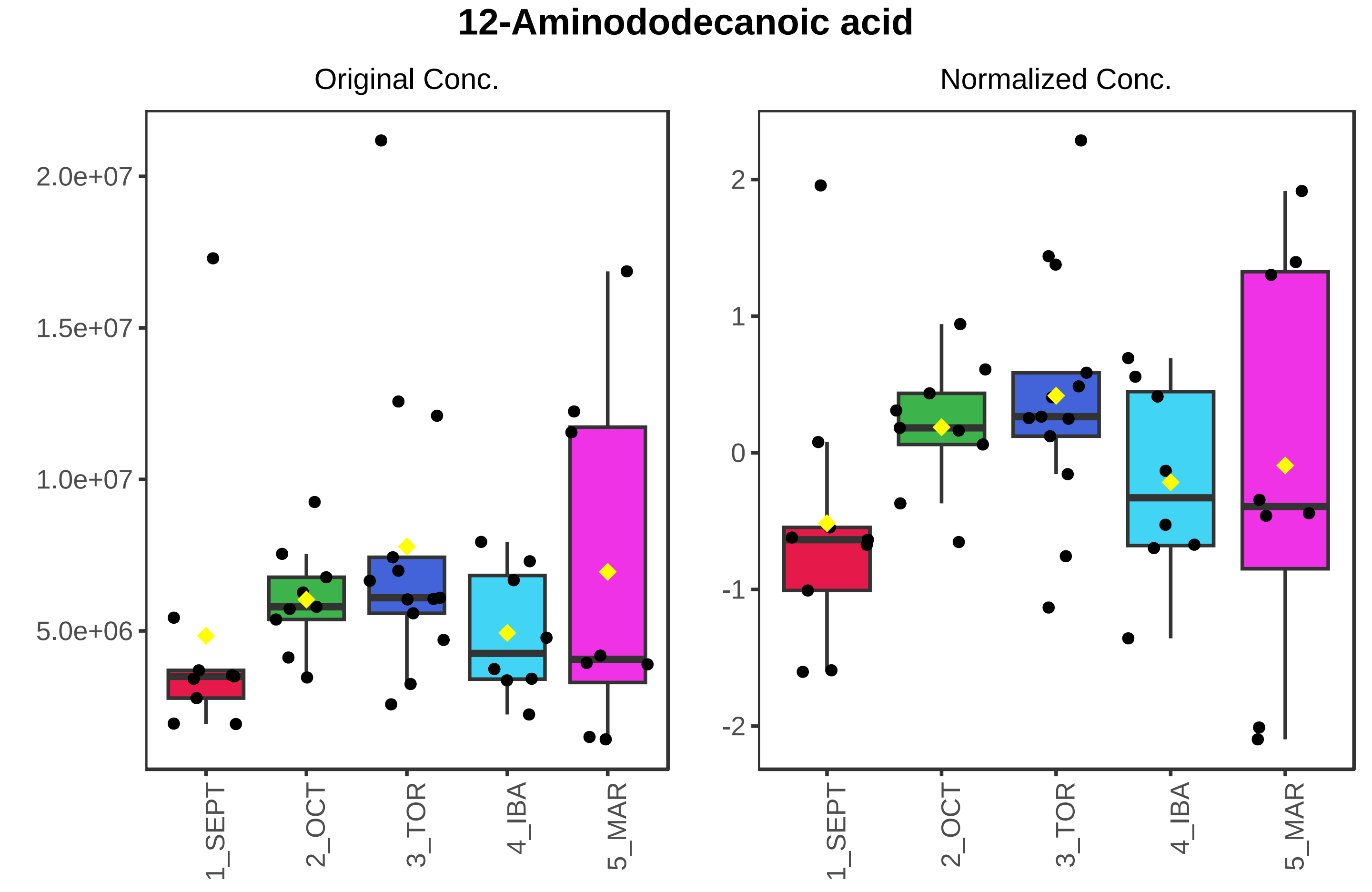
12-Aminododecanoic acid
Original Conc.
Normalized Conc.
2.0e+07
1.5e+07
1.0e+07
5.0e+06
2
1
0
-1
-2
1_SEPT
2_OCT
3_TOR
4_IBA
5_MAR
1_SEPT
2_OCT
3_TOR
4_IBA
5_MAR

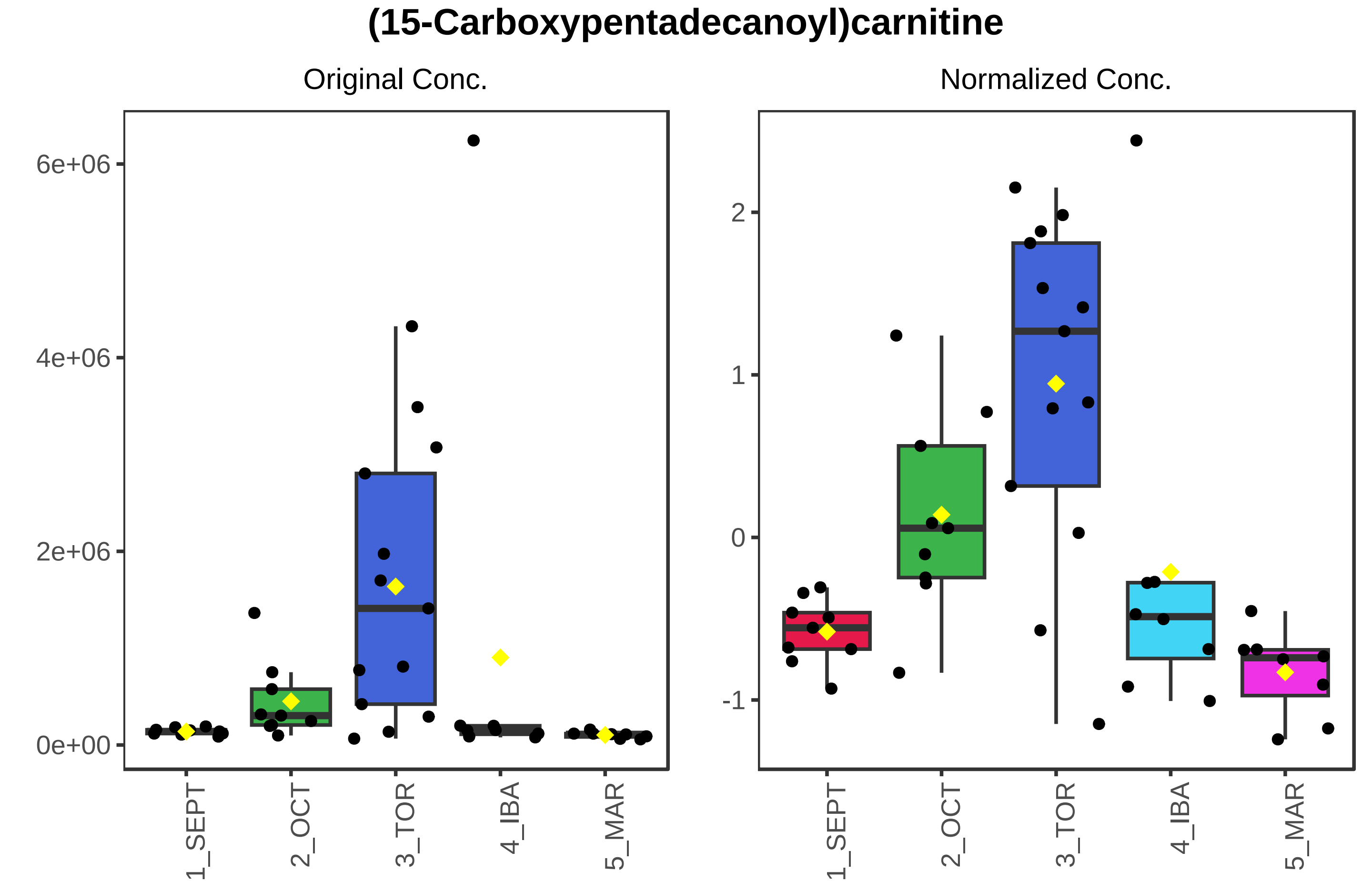
(15-Carboxypentadecanoyl)carnitine
Original Conc.
Normalized Conc.
6e+06
4e+06
2e+06
0e+00
2
1
0
-1
1_SEPT
2_OCT
3_TOR
4_IBA
5_MAR

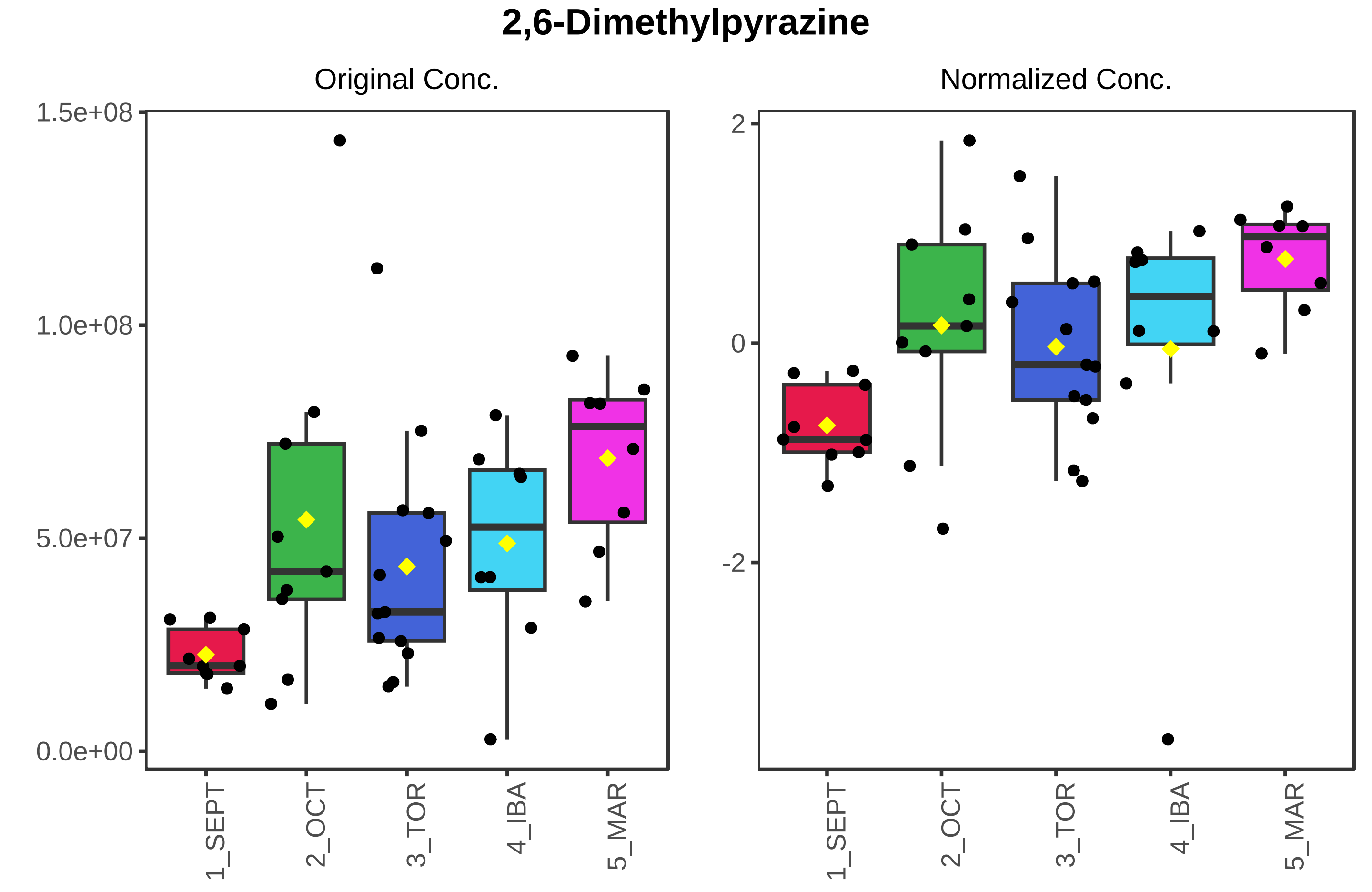

2,6-Dimethylpyrazine
Original Conc.
Normalized Conc.
1.5e+08
1.0e+08
5.0e+07
0.0e+00
2
0
-2
1_SEPT
2_OCT
3_TOR
4_IBA
5_MAR

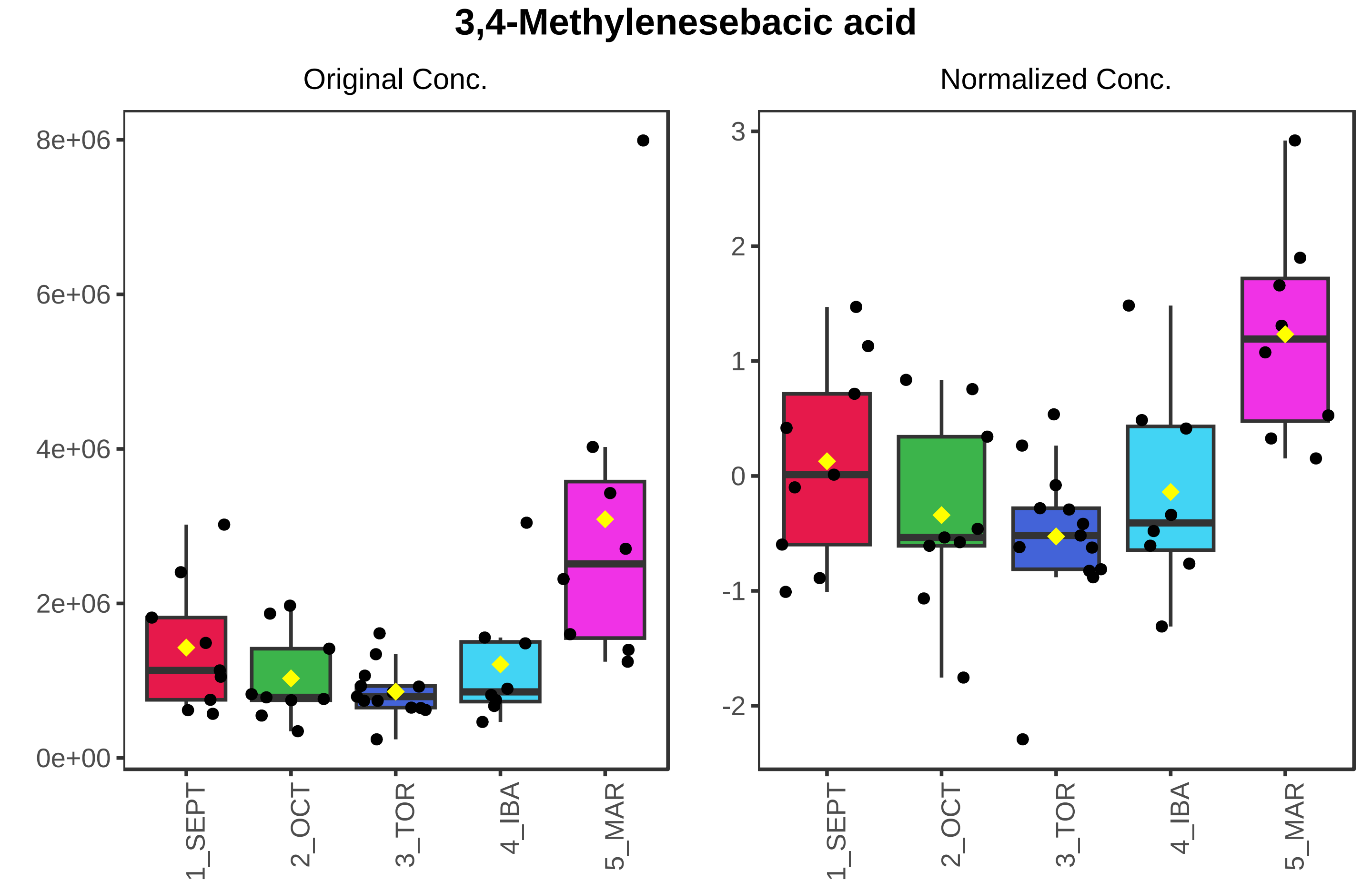

3,4-Methylenesebacic acid
Original Conc.
Normalized Conc.
8e+06
6e+06
4e+06
2e+06
0e+00
3
2
1
0
-1
-2
1_SEPT
2_OCT
3_TOR
4_IBA
5_MAR

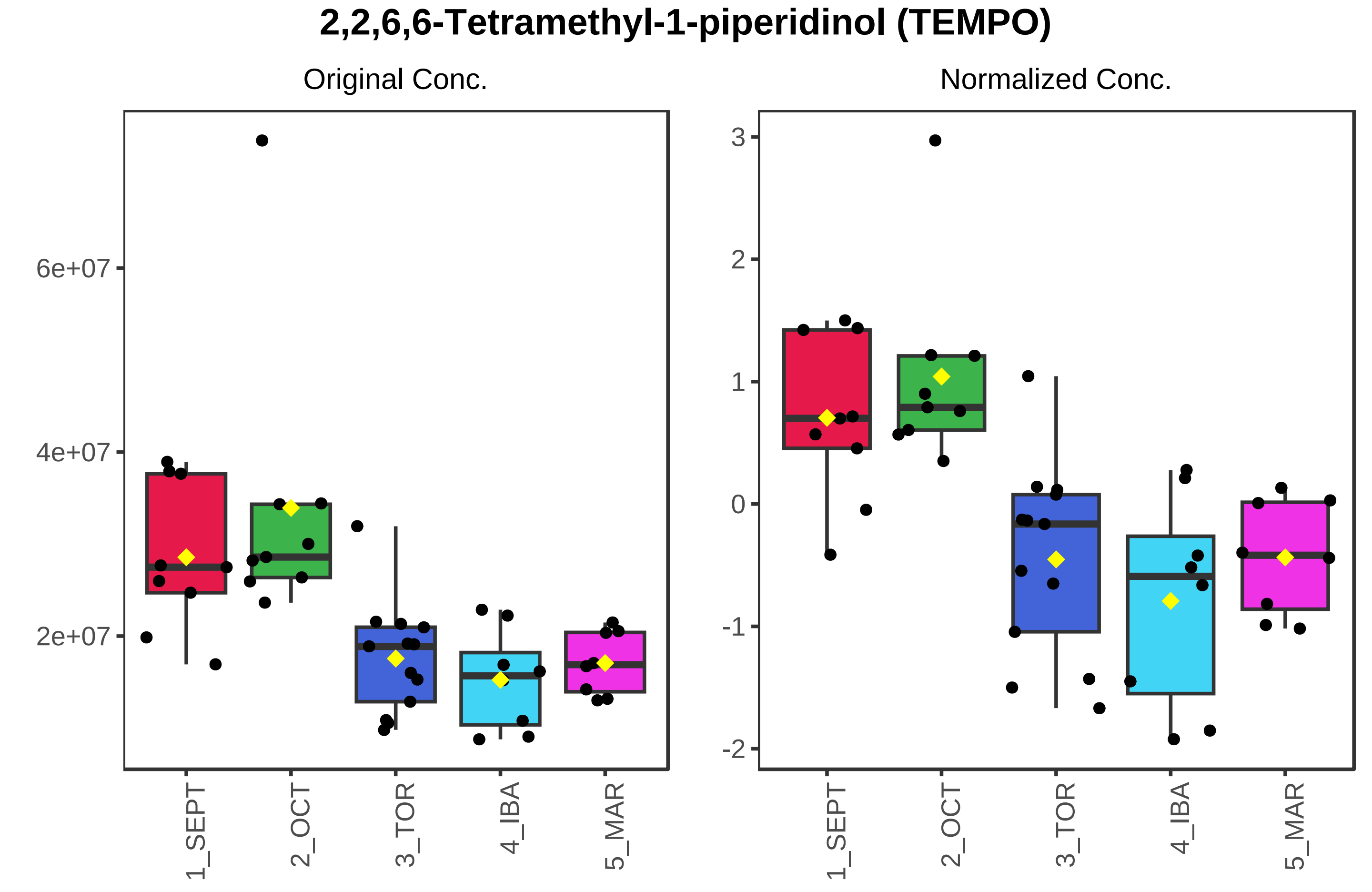

2,2,6,6-Tetramethyl-1-piperidinol (TEMPO)
Original Conc.
Normalized Conc.
6e+07
4e+07
2e+07
3
2
1
0
-1
-2
1_SEPT
2_OCT
3_TOR
4_IBA
5_MAR

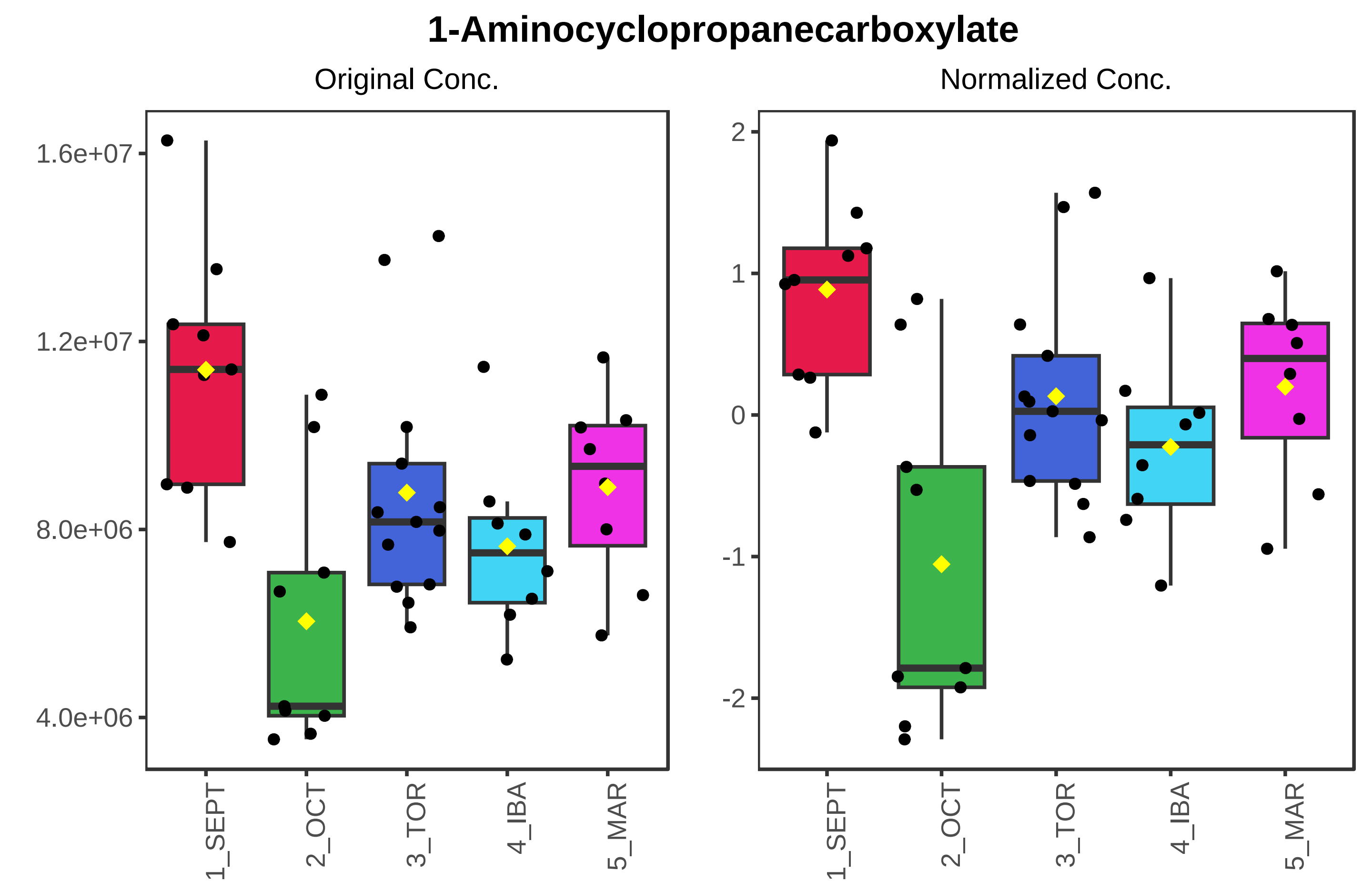

1-Aminocyclopropanecarboxylate
Original Conc.
Normalized Conc.
1.6e+07
1.2e+07
8.0e+06
4.0e+06
2
1
0
-1
-2
1_SEPT
2_OCT
3_TOR
4_IBA
5_MAR

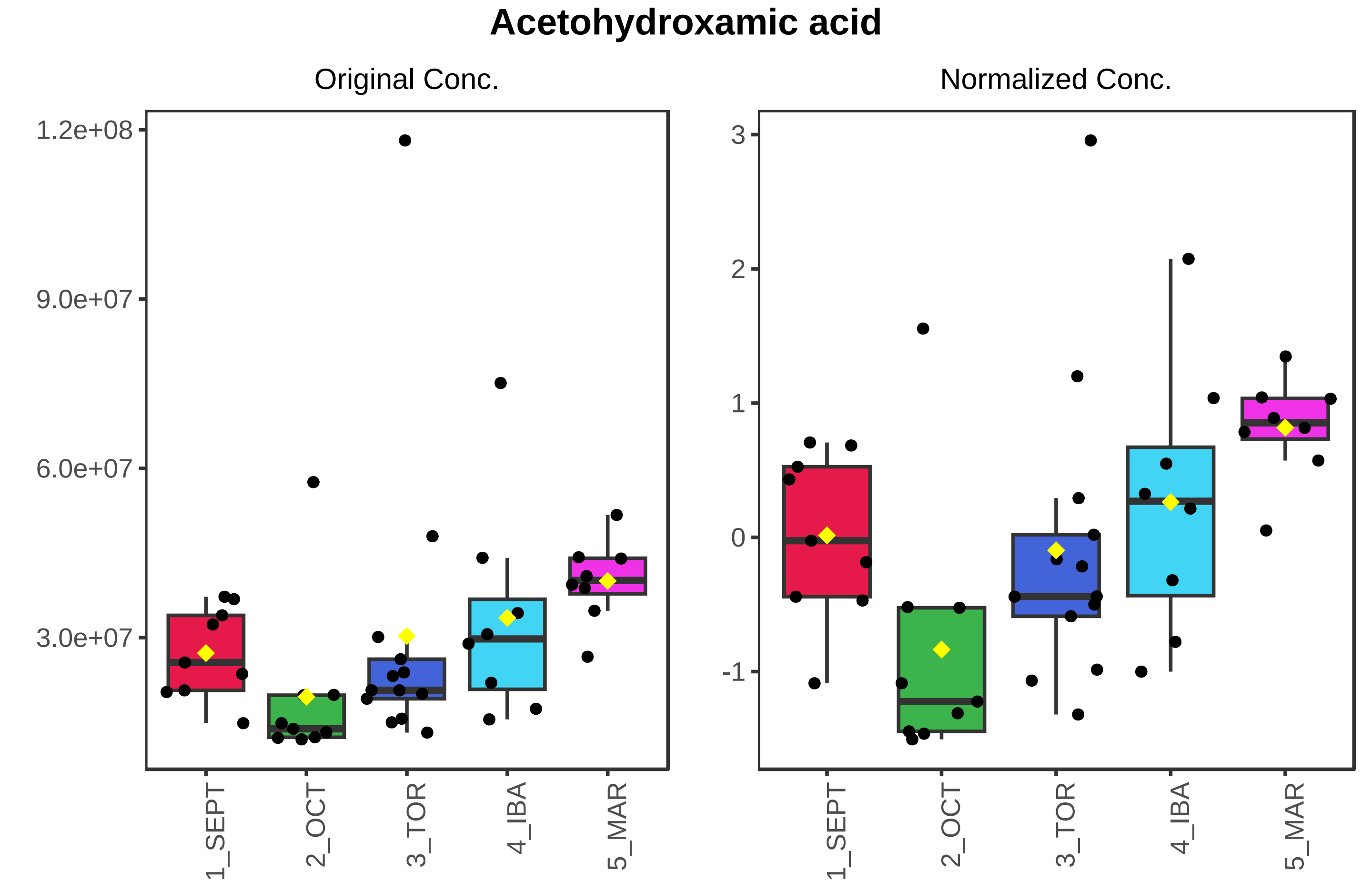

Acetohydroxamic acid
Original Conc.
Normalized Conc.
1.2e+08
9.0e+07
6.0e+07
3.0e+07
3
2
1
0
-1
1_SEPT
2_OCT
3_TOR
4_IBA
5_MAR

Acetylarginine

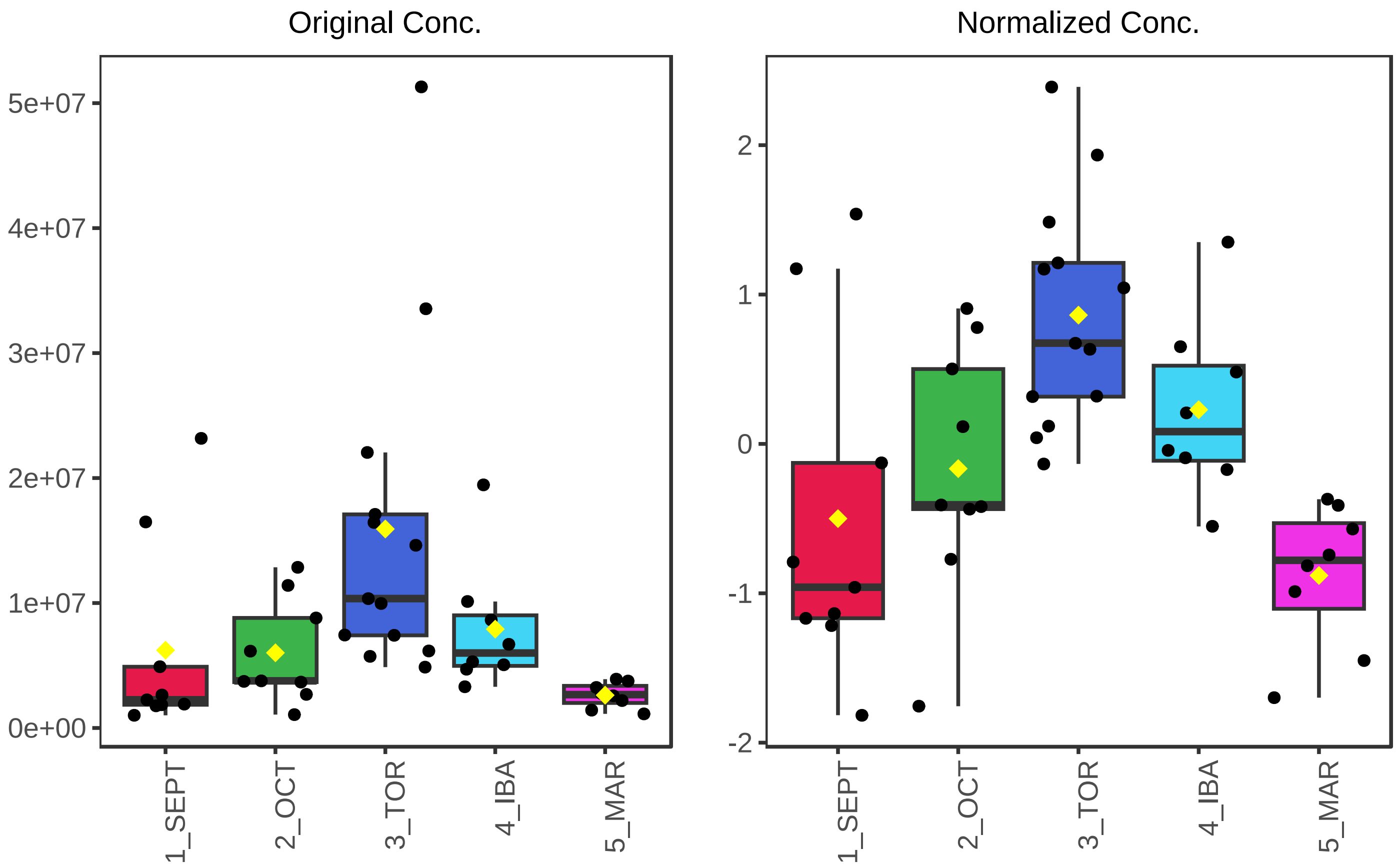

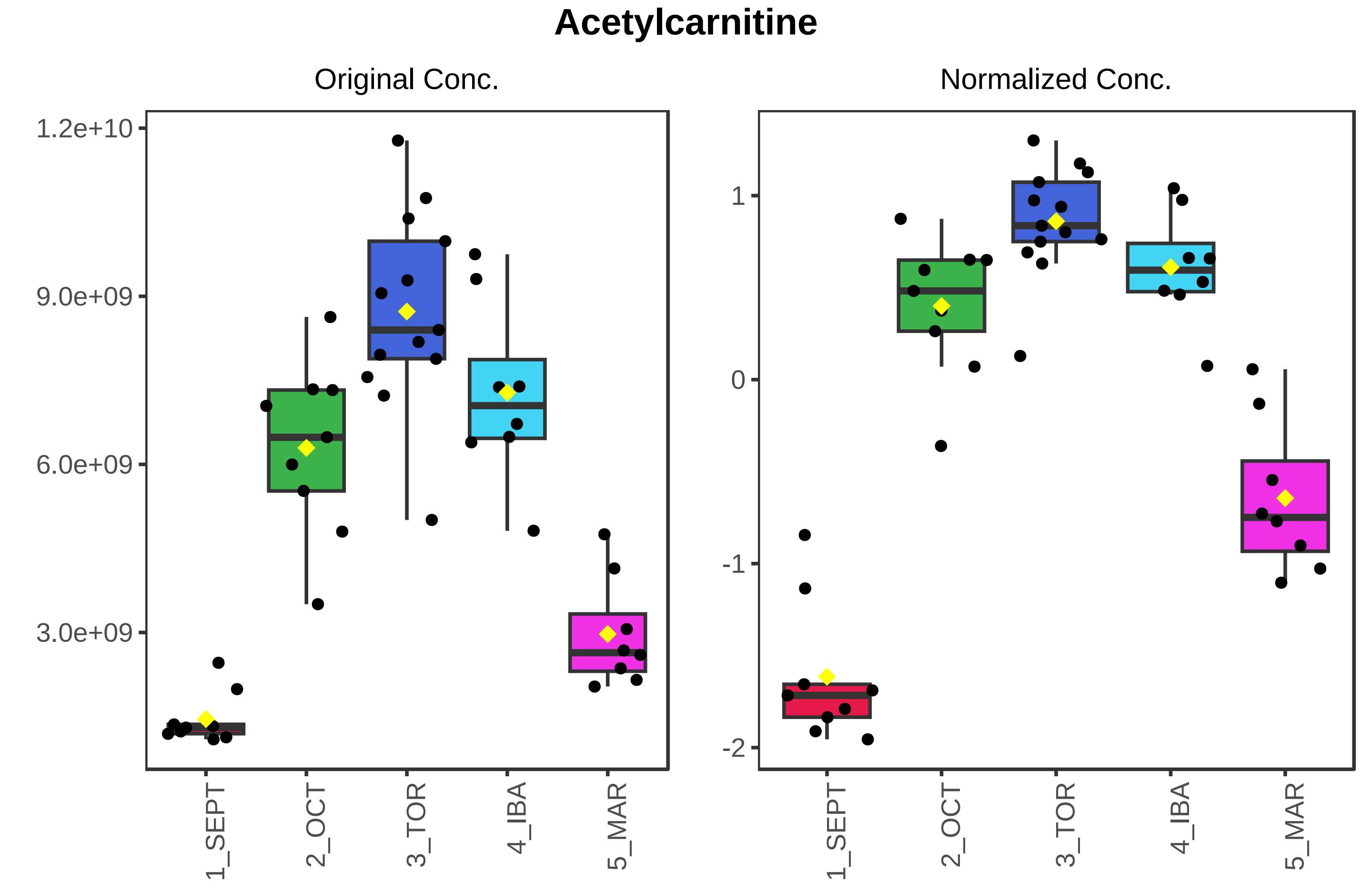

Acetylcarnitine
Original Conc.
Normalized Conc.
1.2e+10
9.0e+09
6.0e+09
3.0e+09
1
0
-1
-2
1_SEPT
2_OCT
3_TOR
4_IBA
5_MAR

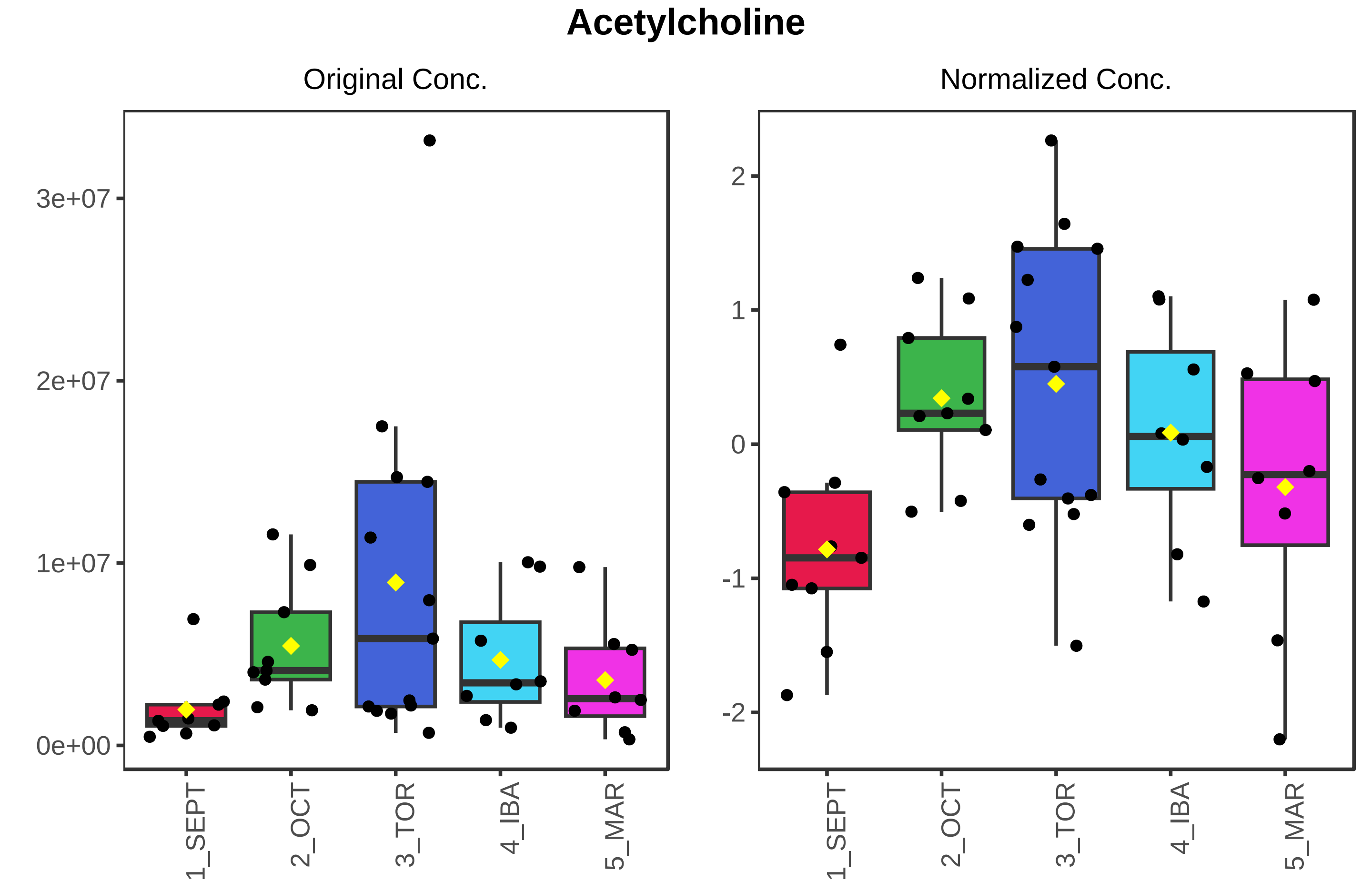

Acetylcholine
Original Conc.
Normalized Conc.
3e+07
2e+07
1e+07
0e+00
2
1
0
-1
-2
1_SEPT
2_OCT
3_TOR
4_IBA
5_MAR

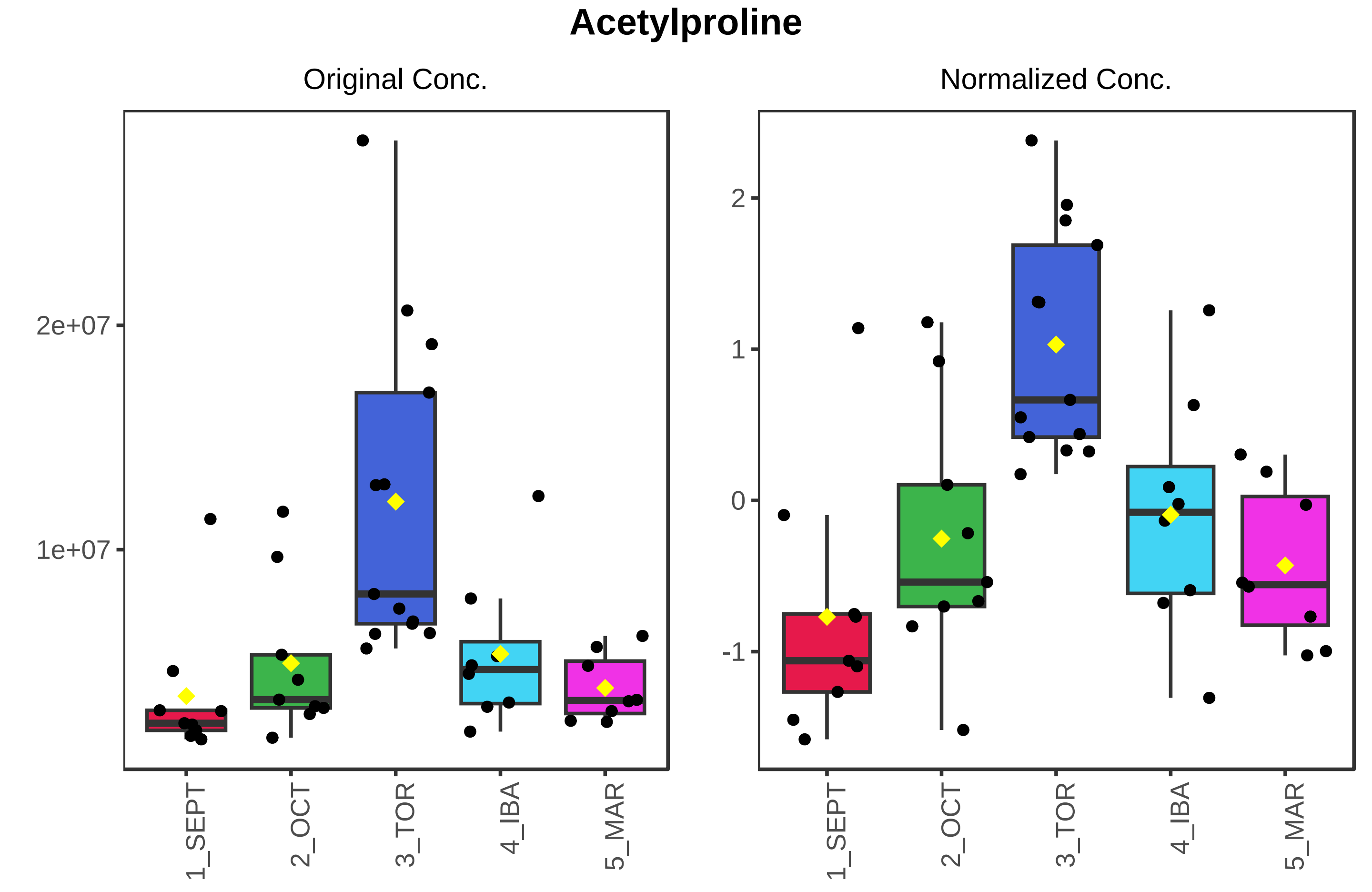
Acetylproline
Original Conc.
Normalized Conc.
2e+07
1e+07
2
1
0
-1
1_SEPT
2_OCT
3_TOR
4_IBA
5_MAR
1_SEPT
2_OCT
3_TOR
4_IBA
5_MAR

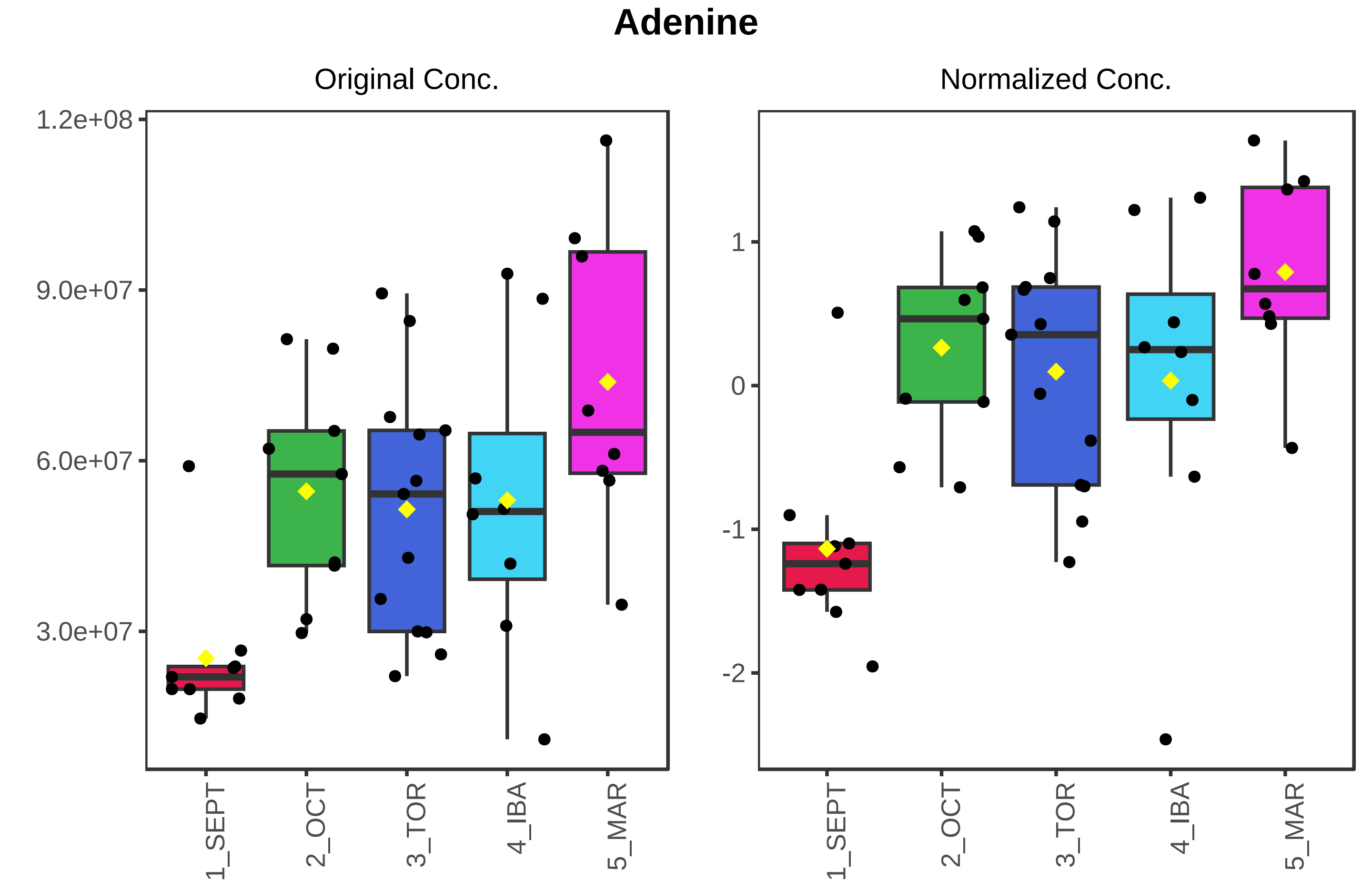

Adenine
Original Conc.
Normalized Conc.
1.2e+08
9.0e+07
6.0e+07
3.0e+07
1
0
-1
-2
1_SEPT
2_OCT
3_TOR
4_IBA
5_MAR

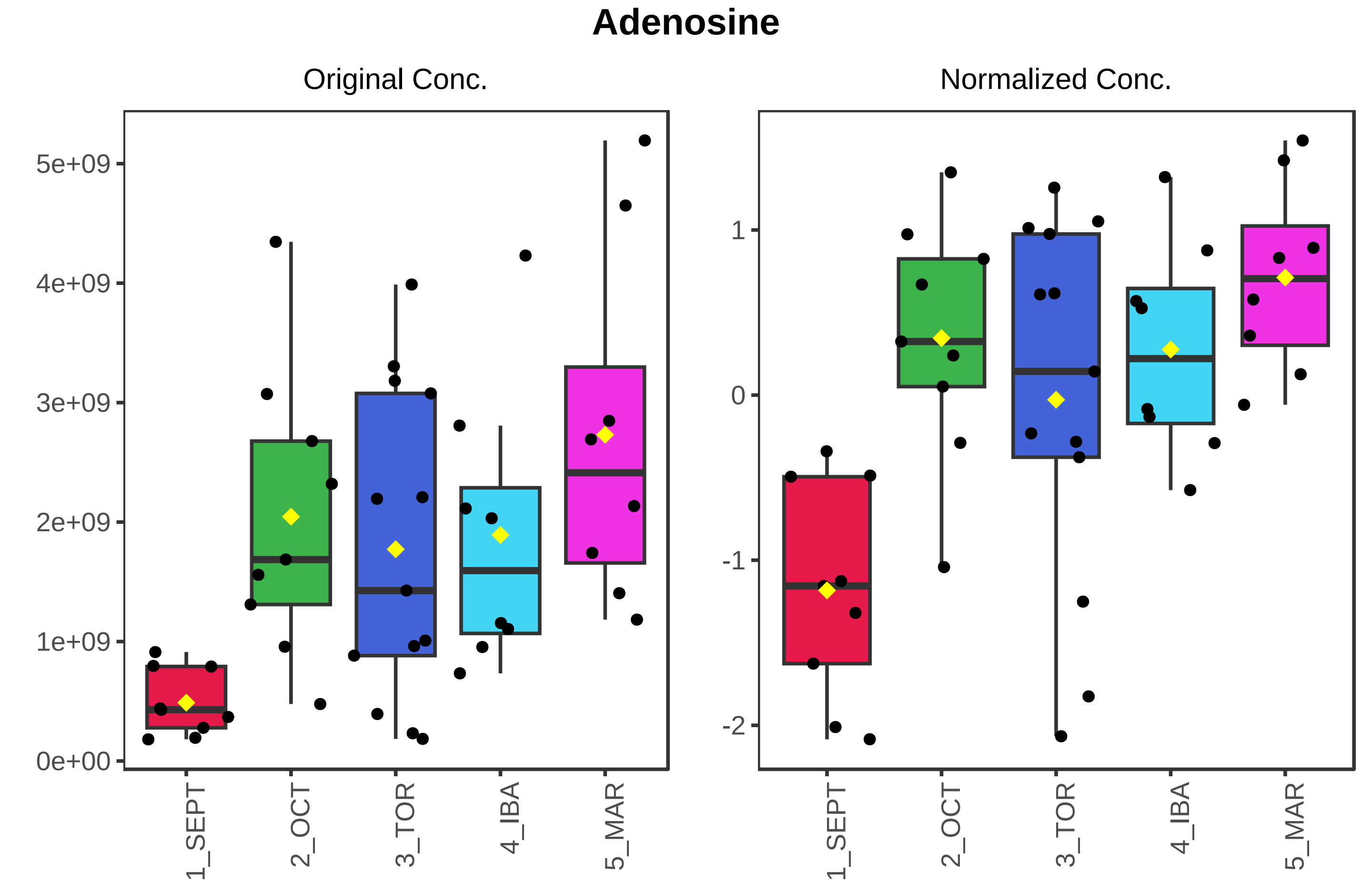
Adenosine
Original Conc.
Normalized Conc.
5e+09
4e+09
3e+09
2e+09
1e+09
0e+00
1
0
-1
-2
1_SEPT
2_OCT
3_TOR
4_IBA
5_MAR

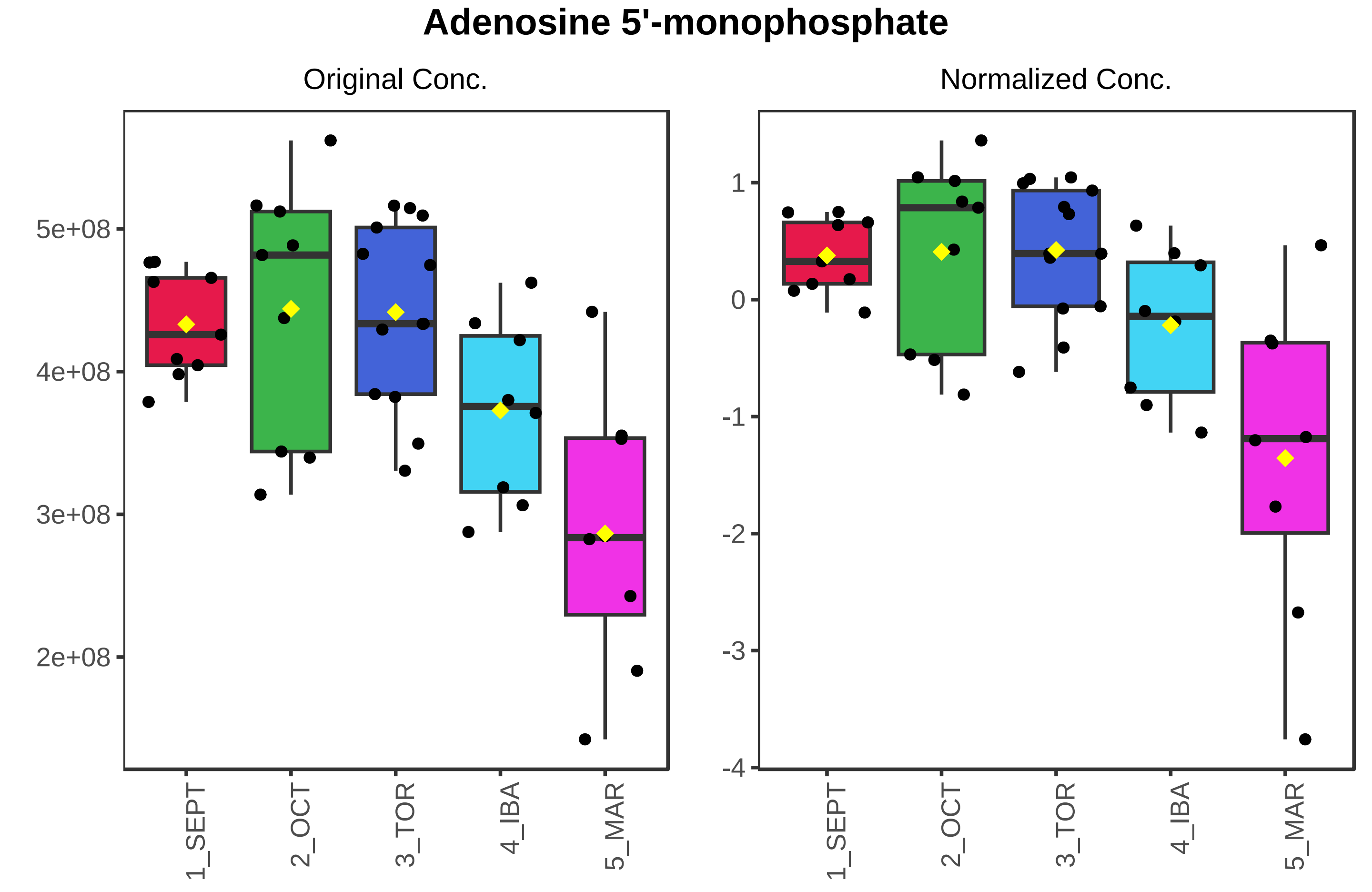

Adenosine 5'-monophosphate
Original Conc.
Normalized Conc.
5e+08
4e+08
3e+08
2e+08
1
0
-1
-2
-3
-4
1_SEPT
2_OCT
3_TOR
4_IBA
5_MAR

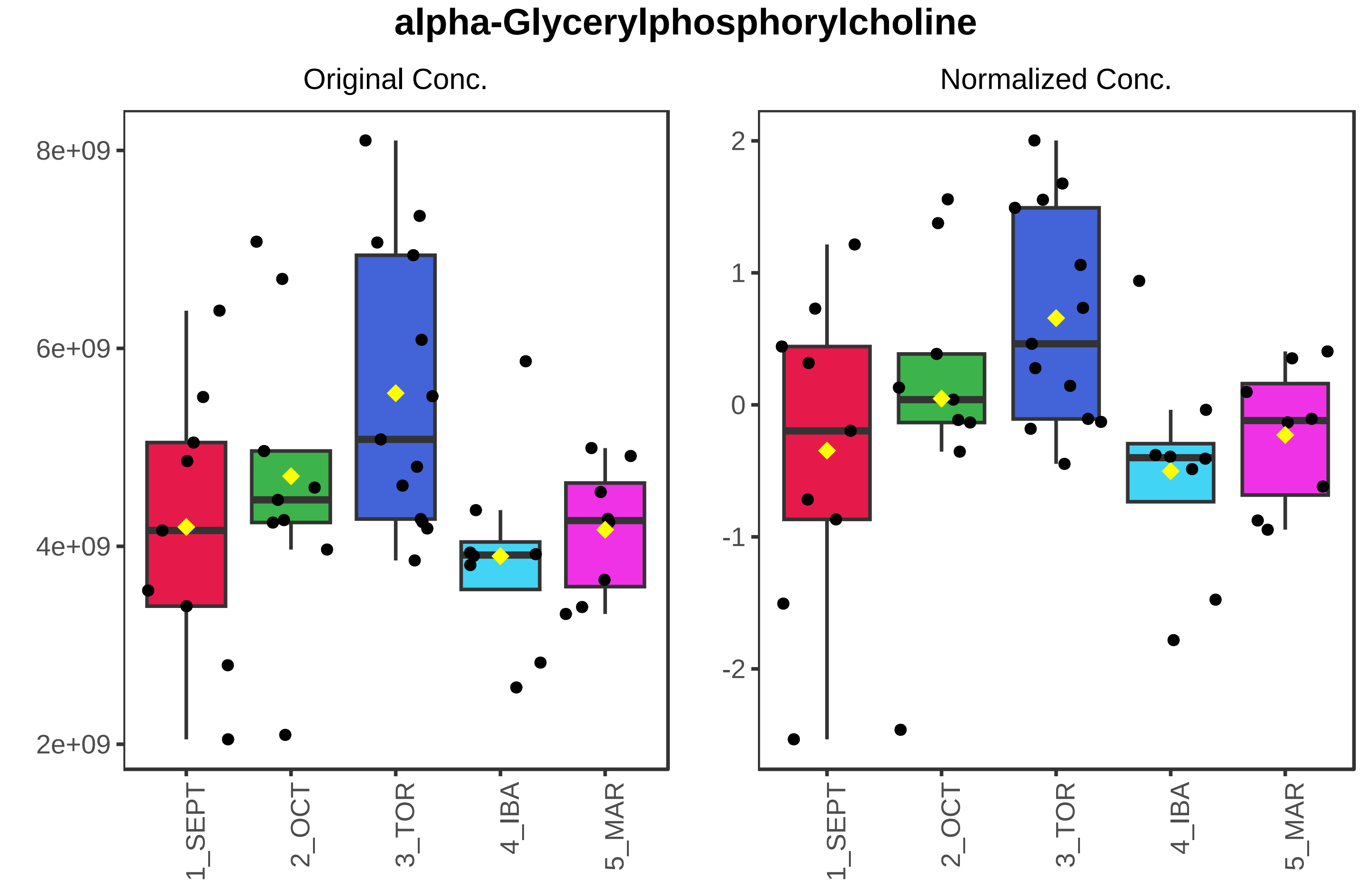

alpha-Glycerylphosphorylcholine
Original Conc.
Normalized Conc.
8e+09
6e+09
4e+09
2e+09
2
1
0
-1
-2
1_SEPT
2_OCT
3_TOR
4_IBA
5_MAR

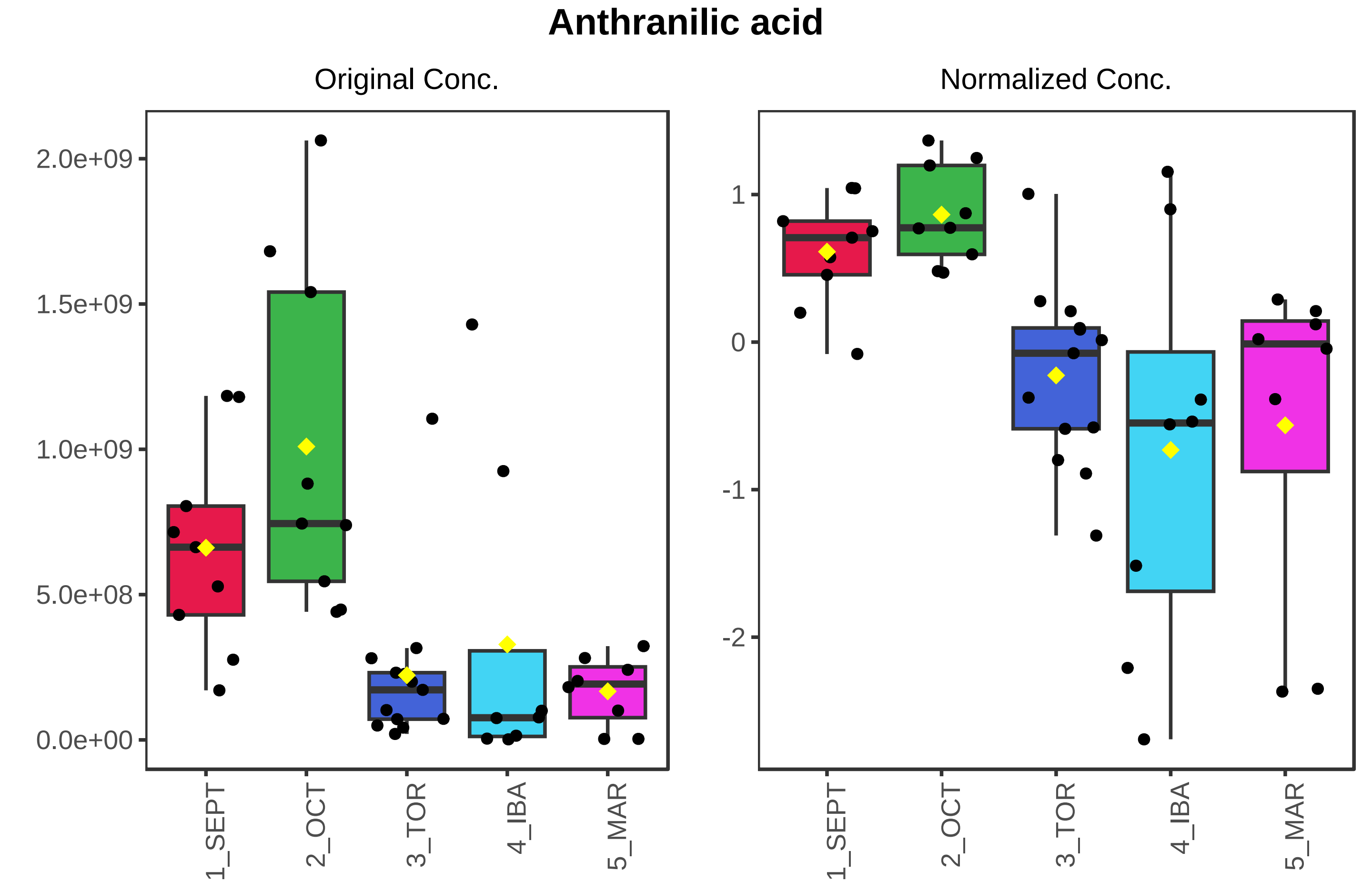
Anthranilic acid
Original Conc.
Normalized Conc.
2.0e+09
1.5e+09
1.0e+09
5.0e+08
0.0e+00
1
0
-1
-2
1_SEPT
2_OCT
3_TOR
4_IBA
5_MAR

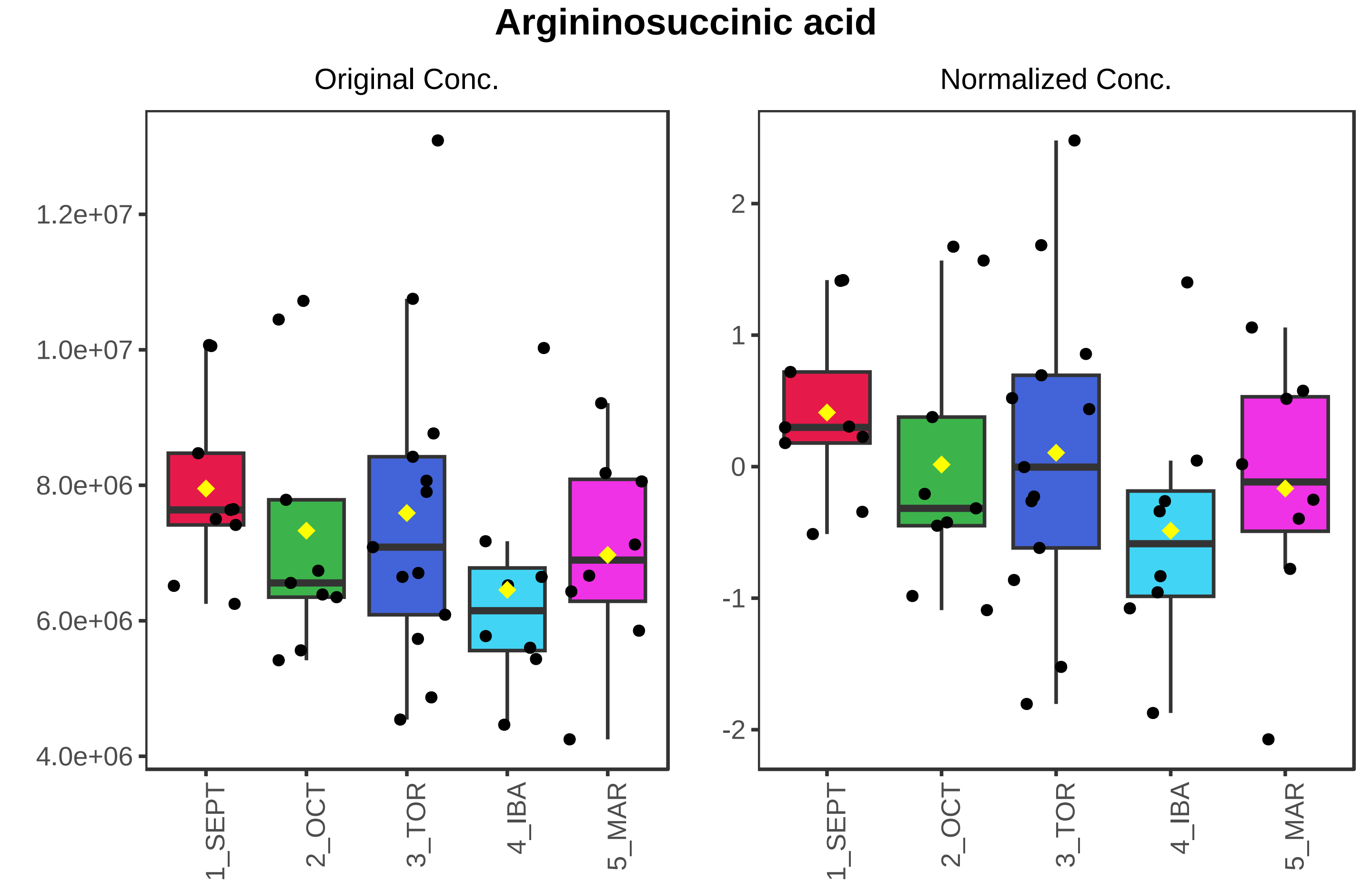

Argininosuccinic acid
Original Conc.
Normalized Conc.
1.2e+07
1.0e+07
8.0e+06
6.0e+06
4.0e+06
2
1
0
-1
-2
1_SEPT
2_OCT
3_TOR
4_IBA
5_MAR

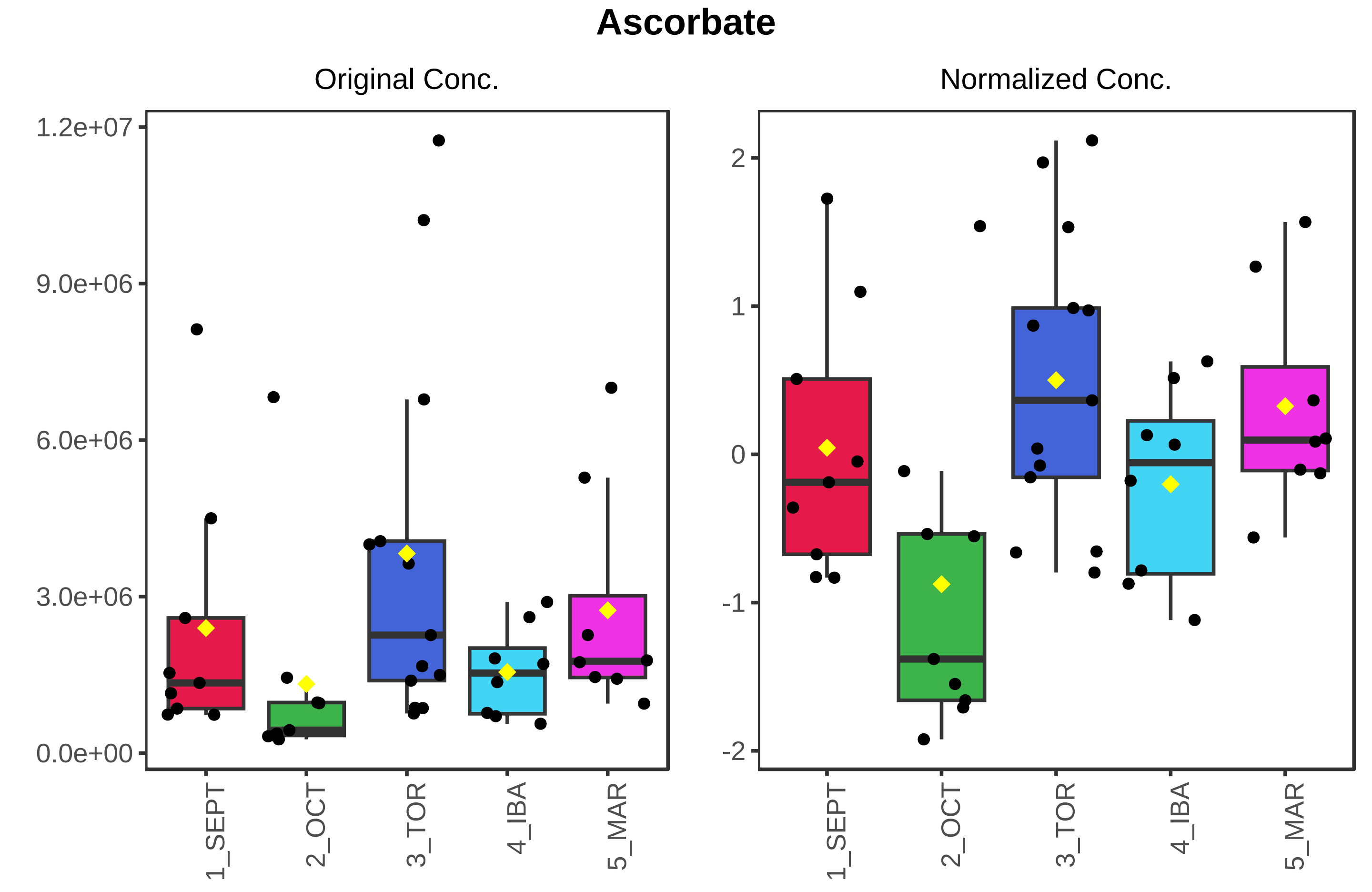

Ascorbate
Original Conc.
Normalized Conc.
1.2e+07
9.0e+06
6.0e+06
3.0e+06
0.0e+00
2
1
0
-1
-2
1_SEPT
2_OCT
3_TOR
4_IBA
5_MAR

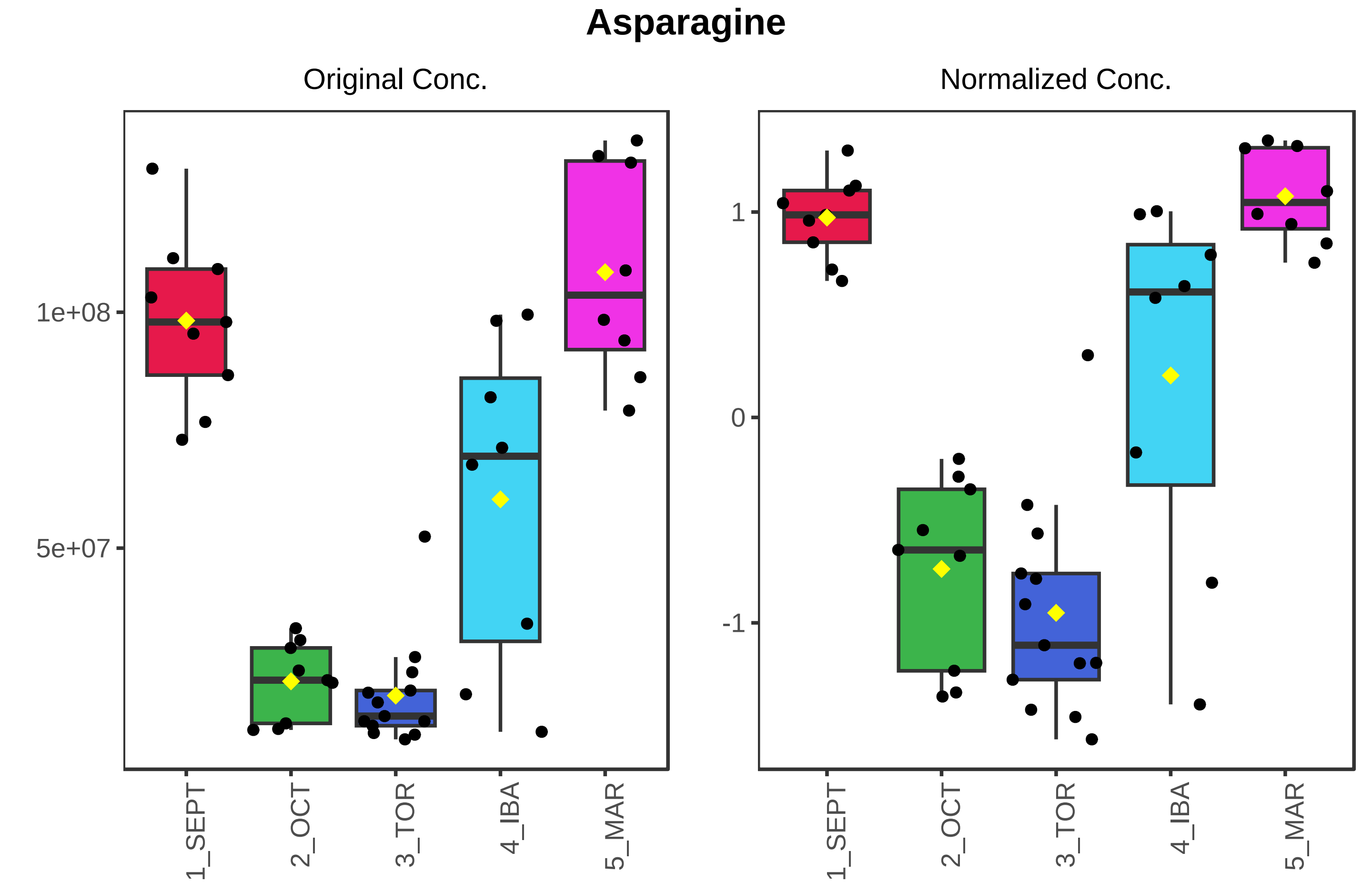

Asparagine
Original Conc.
Normalized Conc.
1e+08
5e+07
1
0
-1
1_SEPT
2_OCT
3_TOR
4_IBA
5_MAR

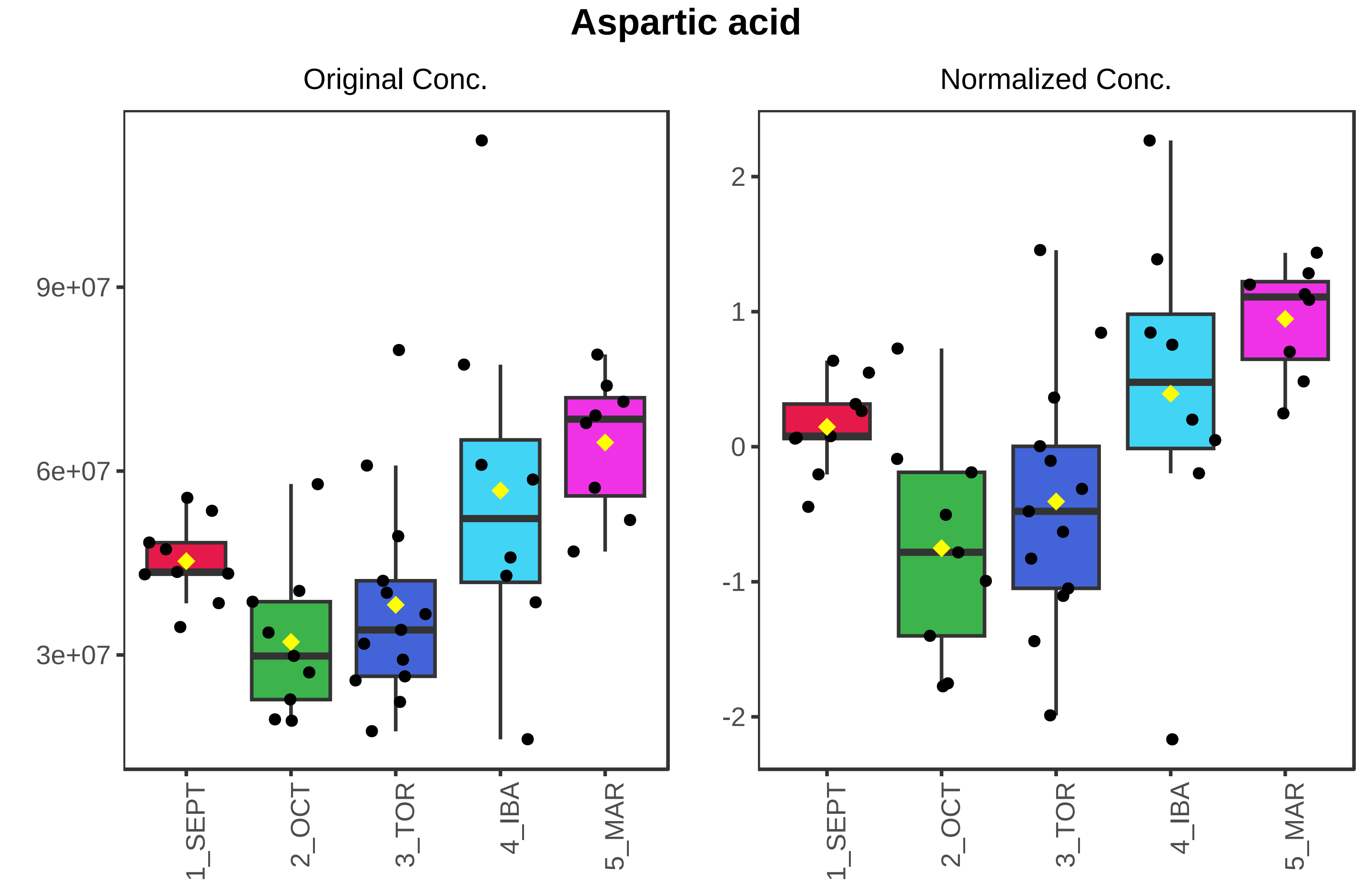
Aspartic acid
Original Conc.
Normalized Conc.
9e+07
6e+07
3e+07
2
1
0
-1
-2
1_SEPT
2_OCT
3_TOR
4_IBA
5_MAR
1_SEPT
2_OCT
3_TOR
4_IBA
5_MAR

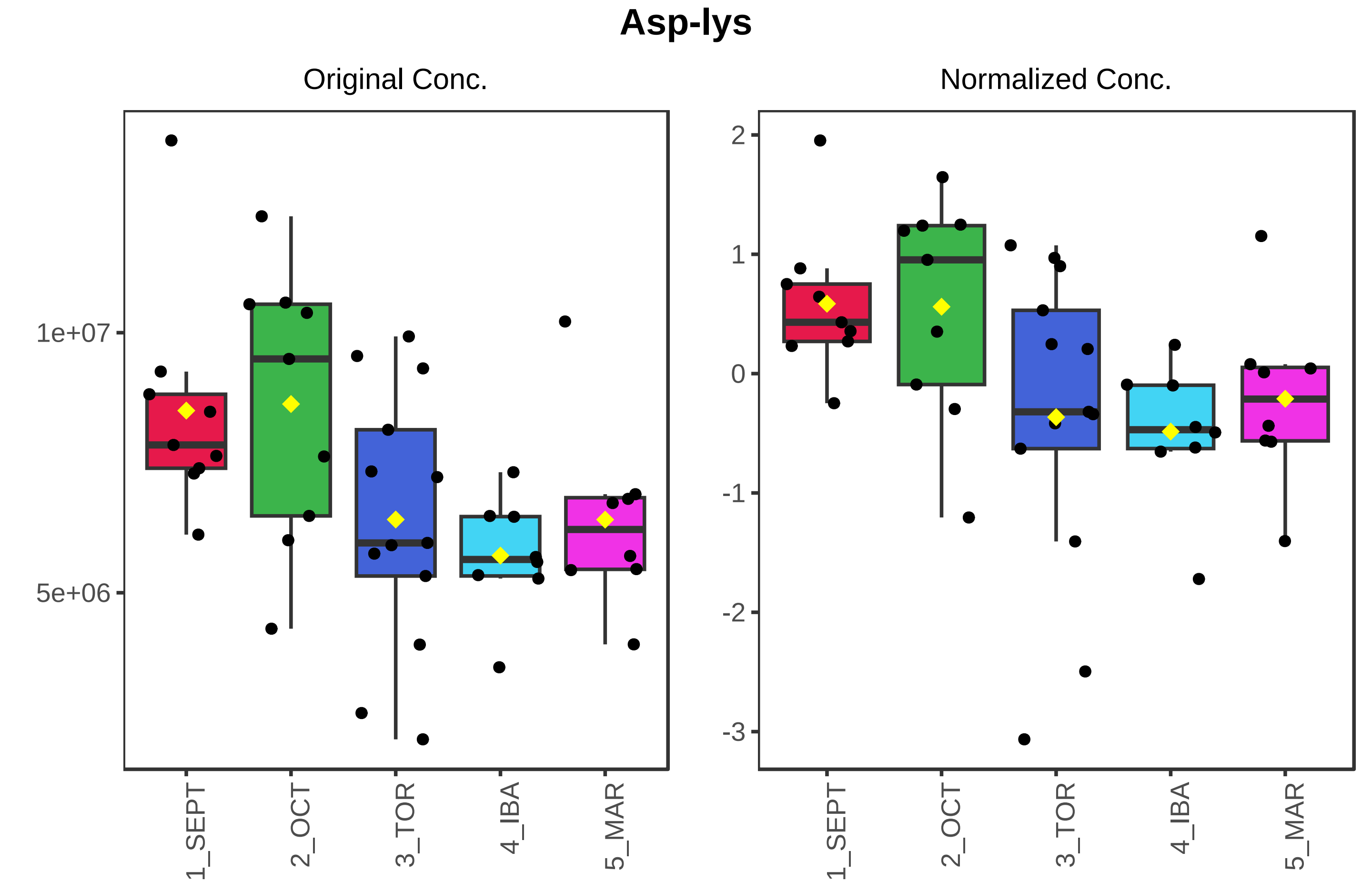

Asp-lys
Original Conc.
Normalized Conc.
1e+07
5e+06
2
1
0
-1
-2
-3
1_SEPT
2_OCT
3_TOR
4_IBA
5_MAR

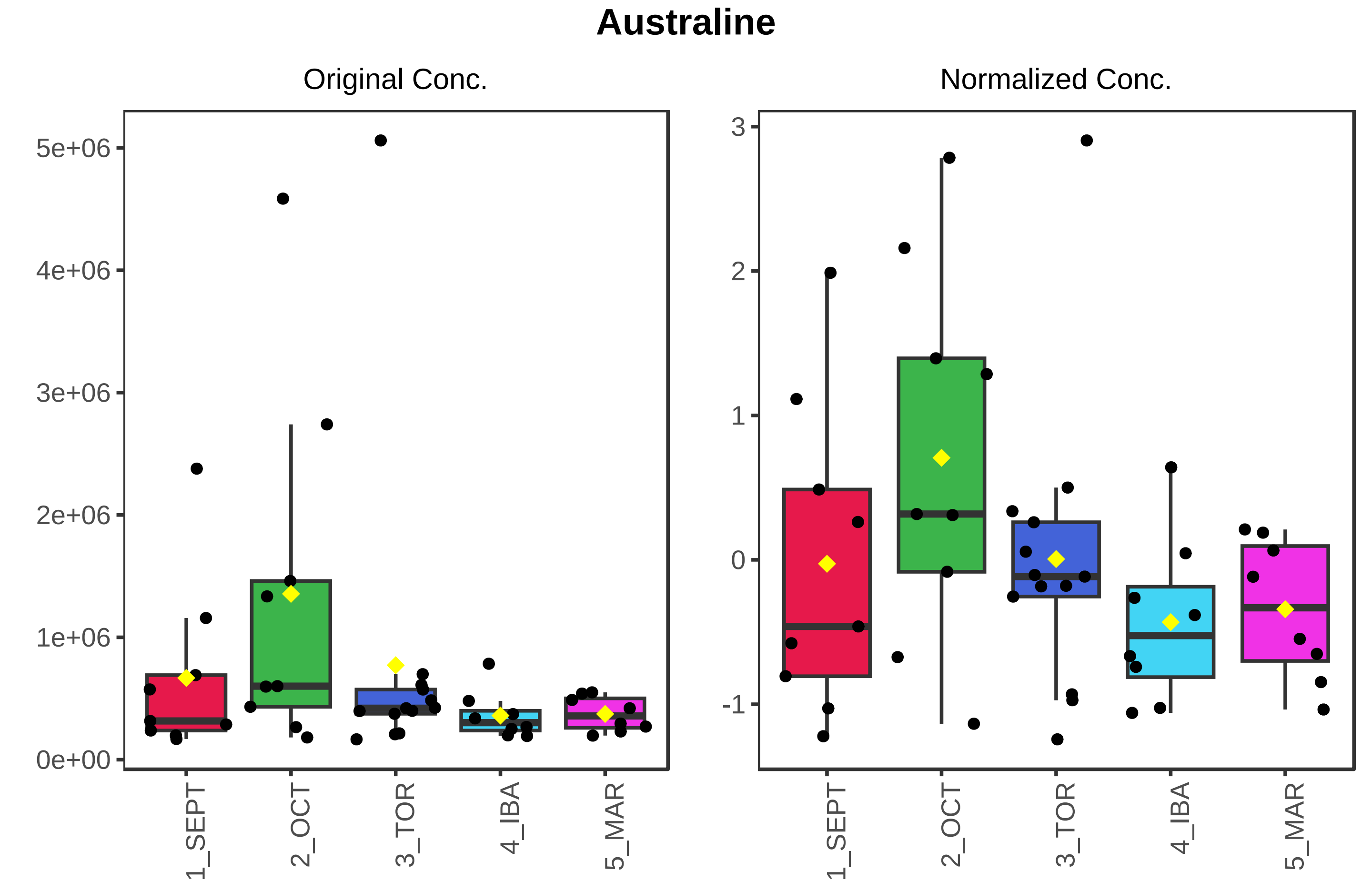

Australine
Original Conc.
Normalized Conc.
5e+06
4e+06
3e+06
2e+06
1e+06
0e+00
3
2
1
0
-1
1_SEPT
2_OCT
3_TOR
4_IBA
5_MAR
1_SEPT
2_OCT
3_TOR
4_IBA
5_MAR

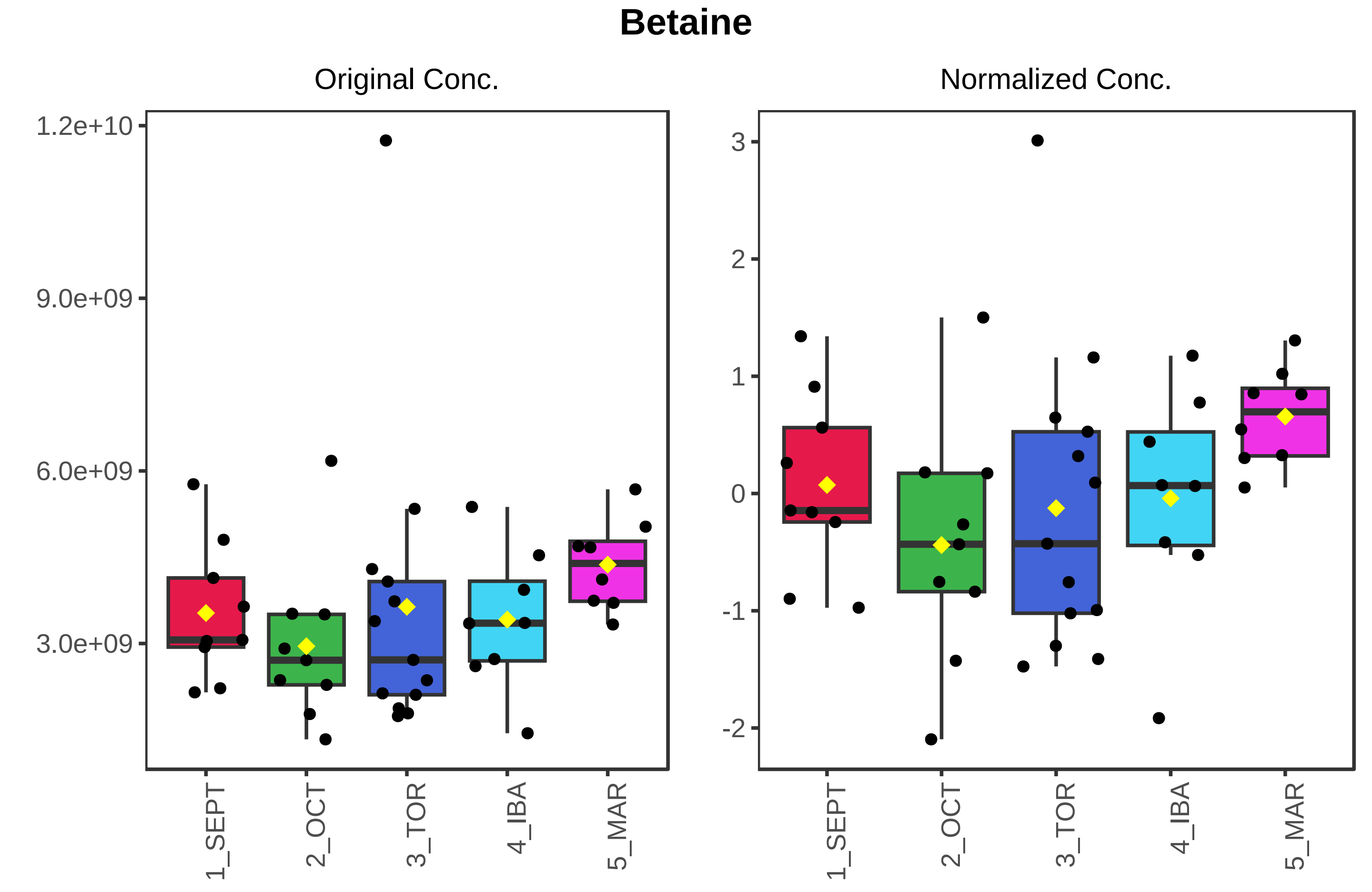

Betaine
Original Conc.
Normalized Conc.
1.2e+10
9.0e+09
6.0e+09
3.0e+09
3
2
1
0
-1
-2
1_SEPT
2_OCT
3_TOR
4_IBA
5_MAR

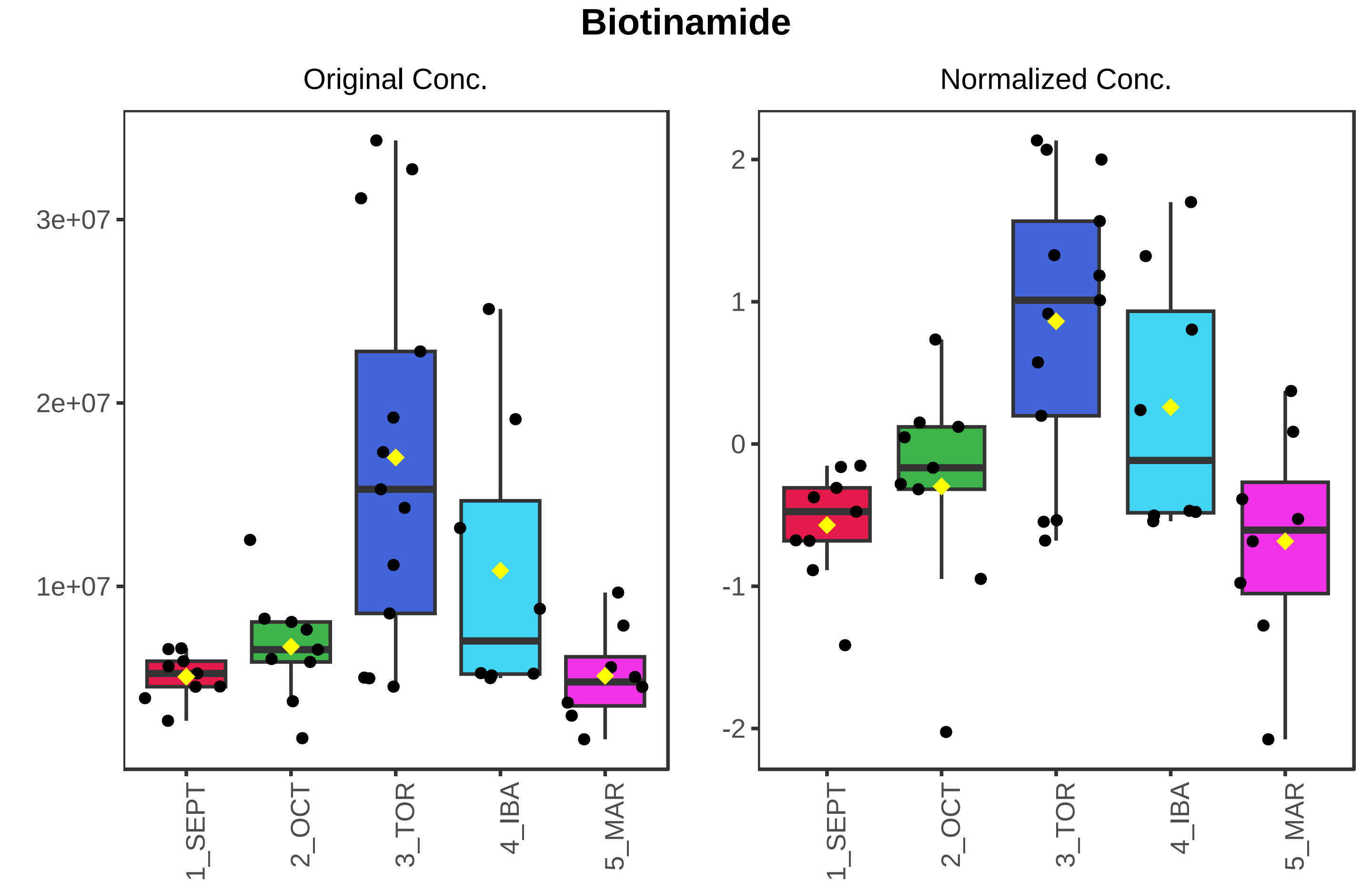

Biotinamide
Original Conc.
Normalized Conc.
3e+07
2e+07
1e+07
2
1
0
-1
-2
1_SEPT
2_OCT
3_TOR
4_IBA
5_MAR

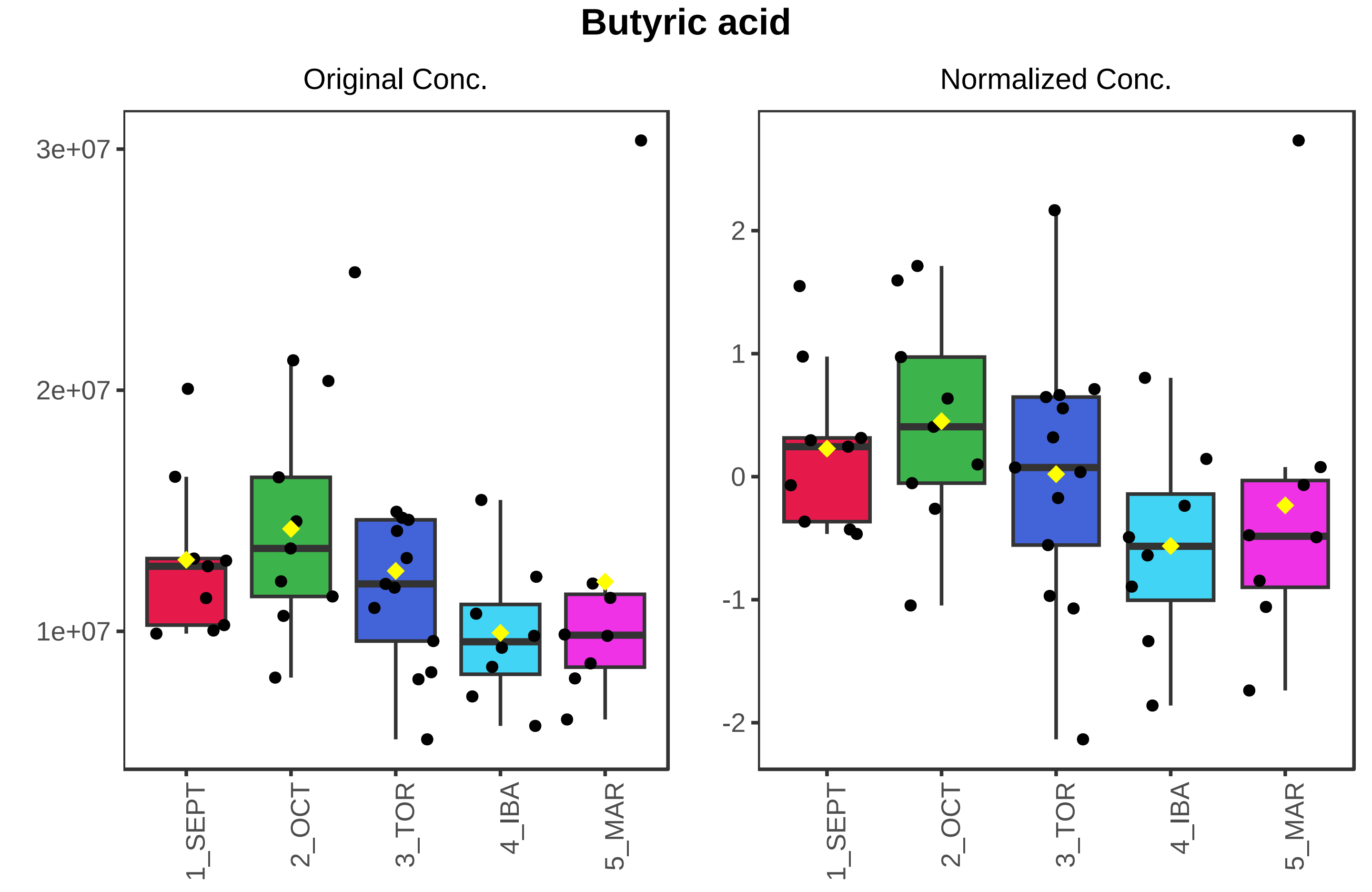

Butyric acid
Original Conc.
Normalized Conc.
3e+07
2e+07
1e+07
2
1
0
-1
-2
1_SEPT
2_OCT
3_TOR
4_IBA
5_MAR

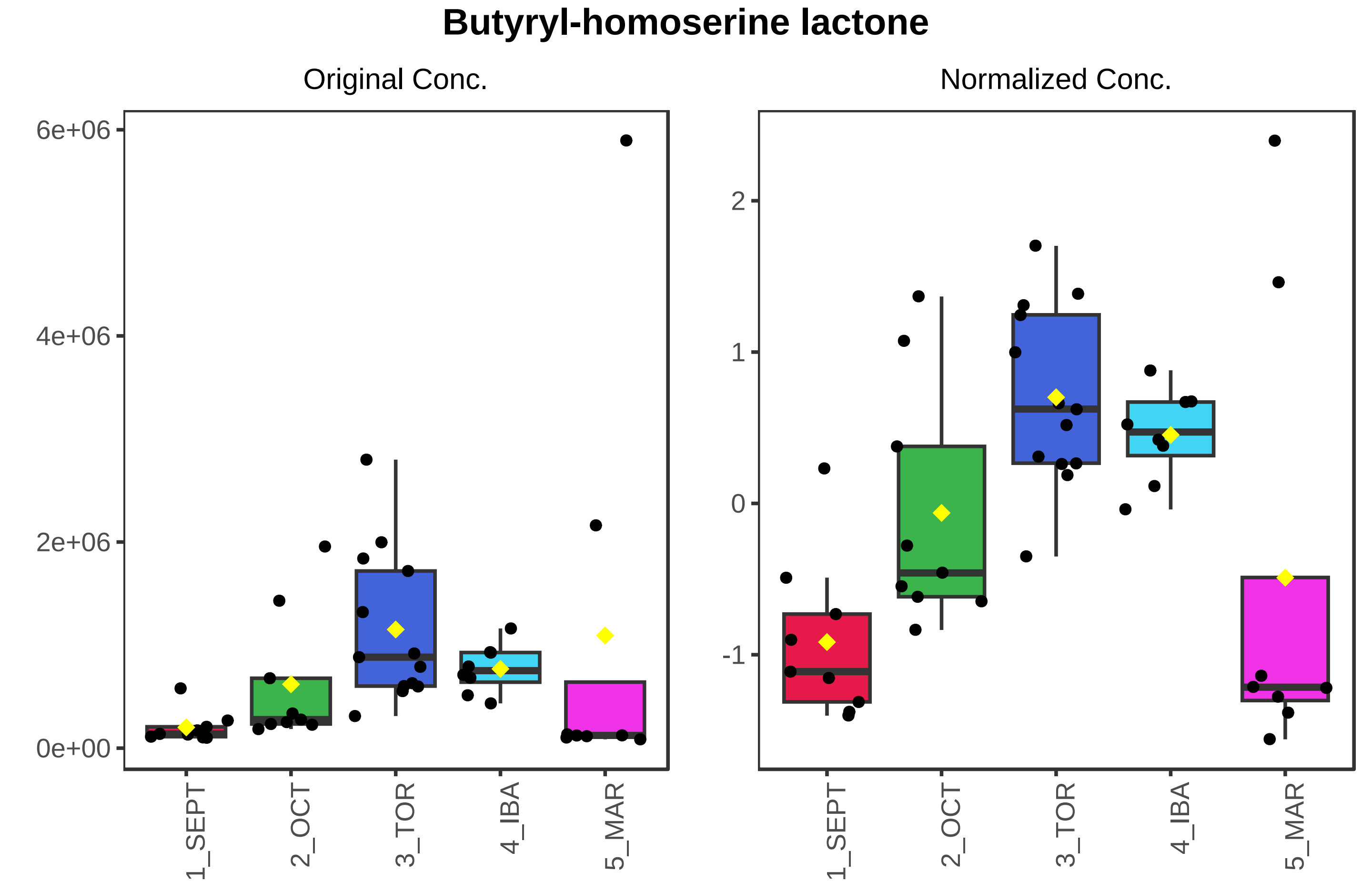
Butyryl-homoserine lactone
Original Conc.
Normalized Conc.
6e+06
4e+06
2e+06
0e+00
2
1
0
-1
1_SEPT
2_OCT
3_TOR
4_IBA
5_MAR

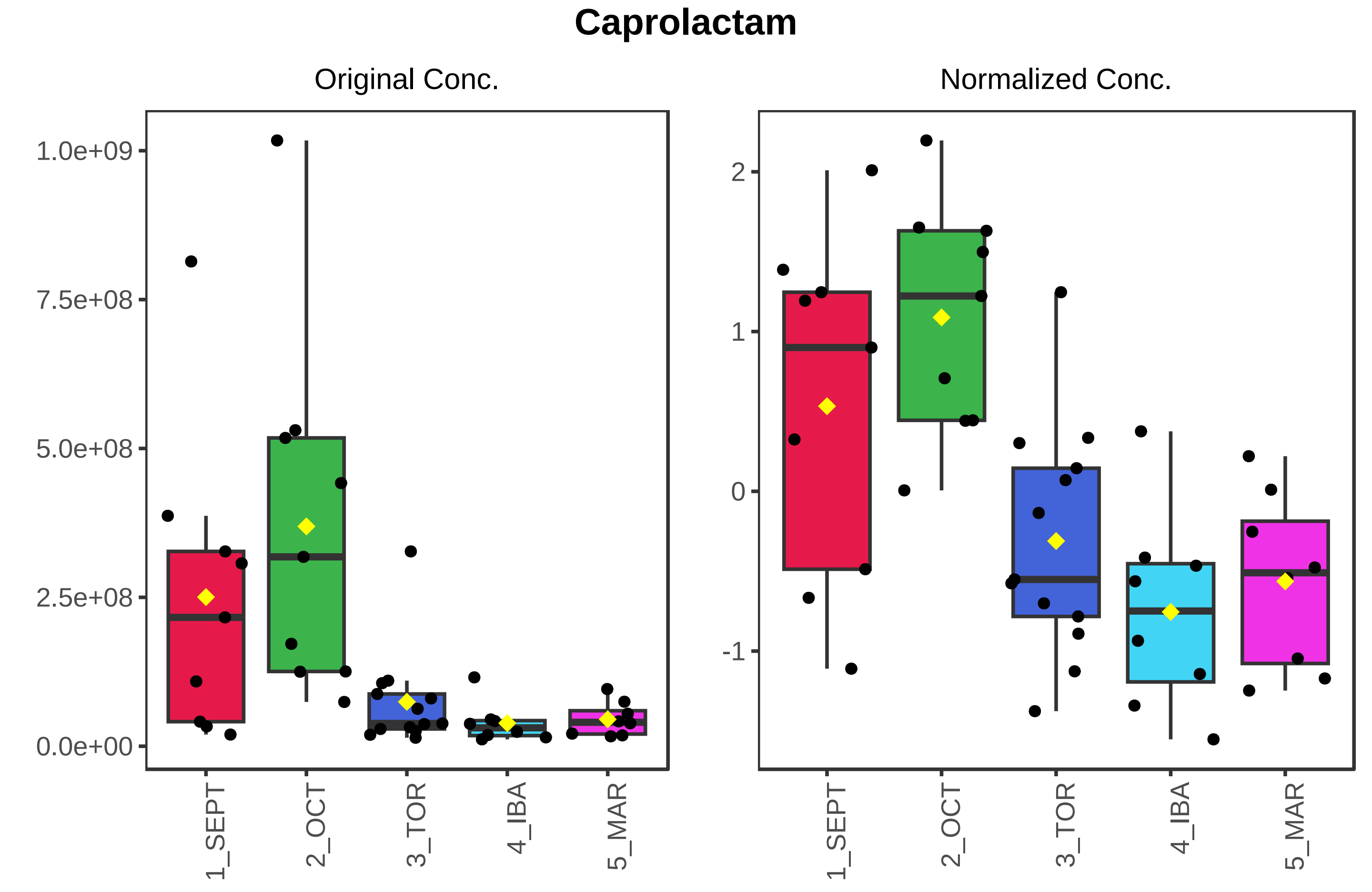
Caprolactam
Original Conc.
Normalized Conc.
1.0e+09
7.5e+08
5.0e+08
2.5e+08
0.0e+00
2
1
0
-1
1_SEPT
2_OCT
3_TOR
4_IBA
5_MAR

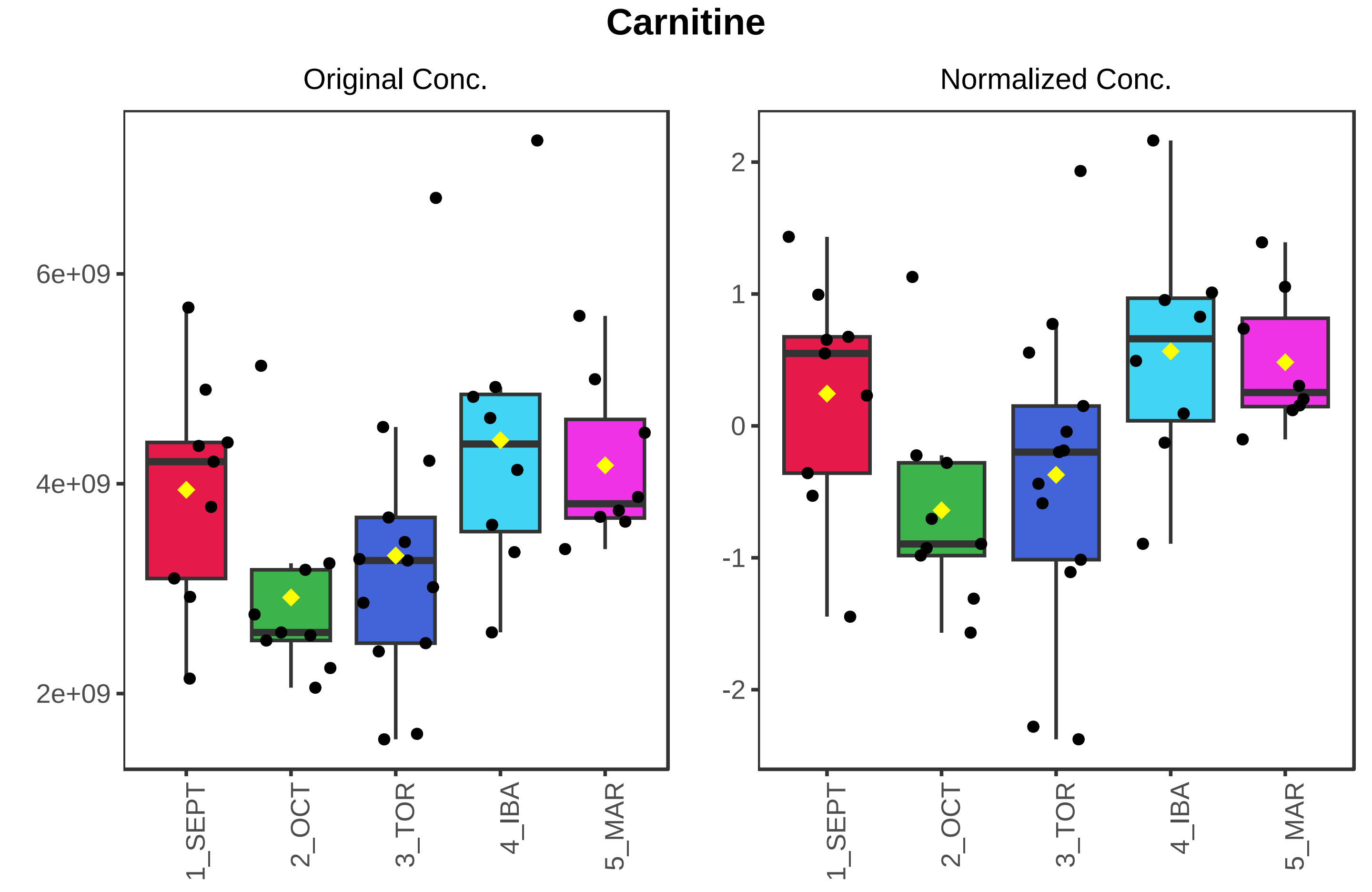
Carnitine
Original Conc.
Normalized Conc.
6e+09
4e+09
2e+09
2
1
0
-1
-2
1_SEPT
2_OCT
3_TOR
4_IBA
5_MAR
1_SEPT
2_OCT
3_TOR
4_IBA
5_MAR

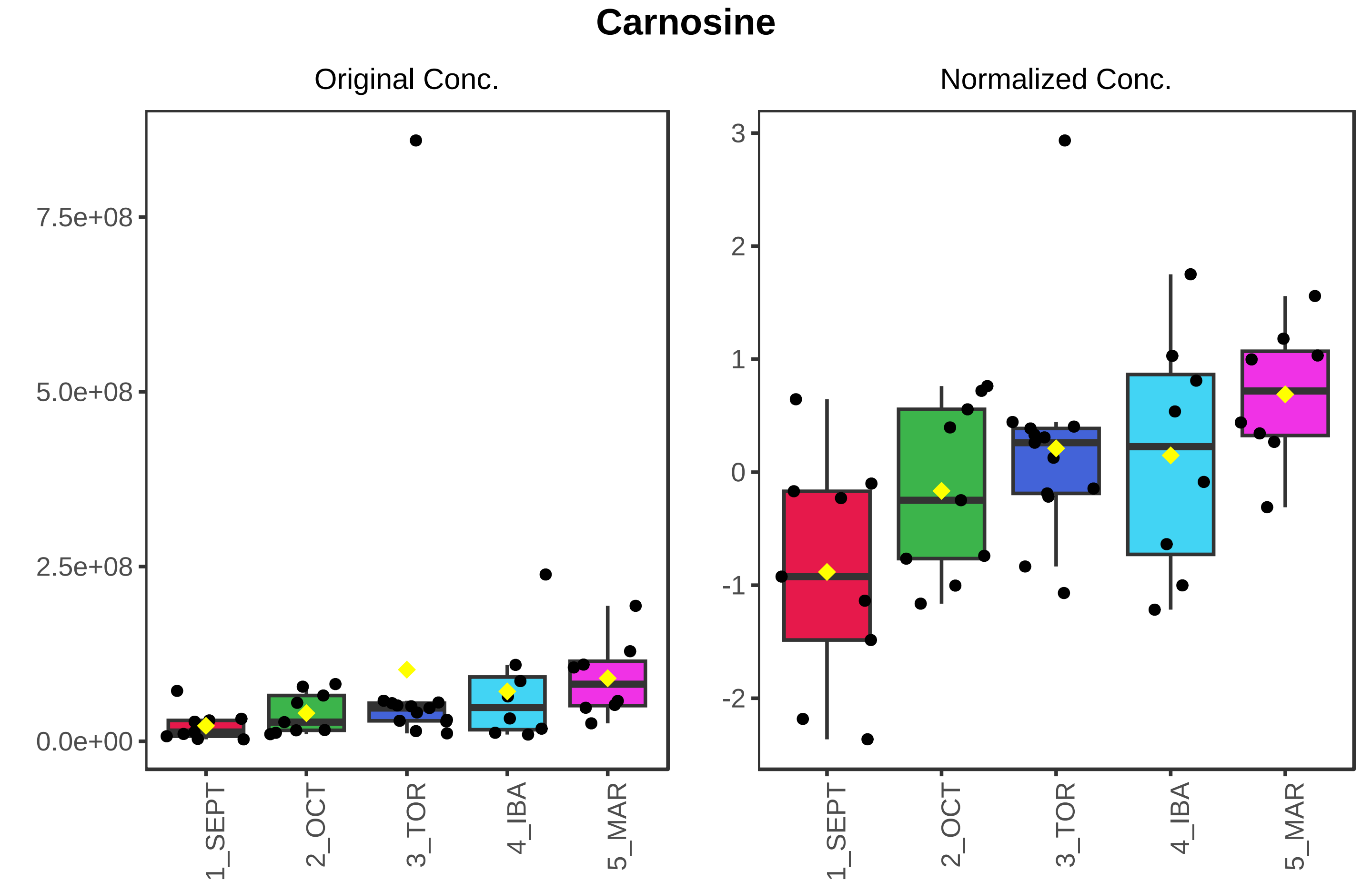
Carnosine
Original Conc.
Normalized Conc.
7.5e+08
5.0e+08
2.5e+08
0.0e+00
3
2
1
0
-1
-2
1_SEPT
2_OCT
3_TOR
4_IBA
5_MAR

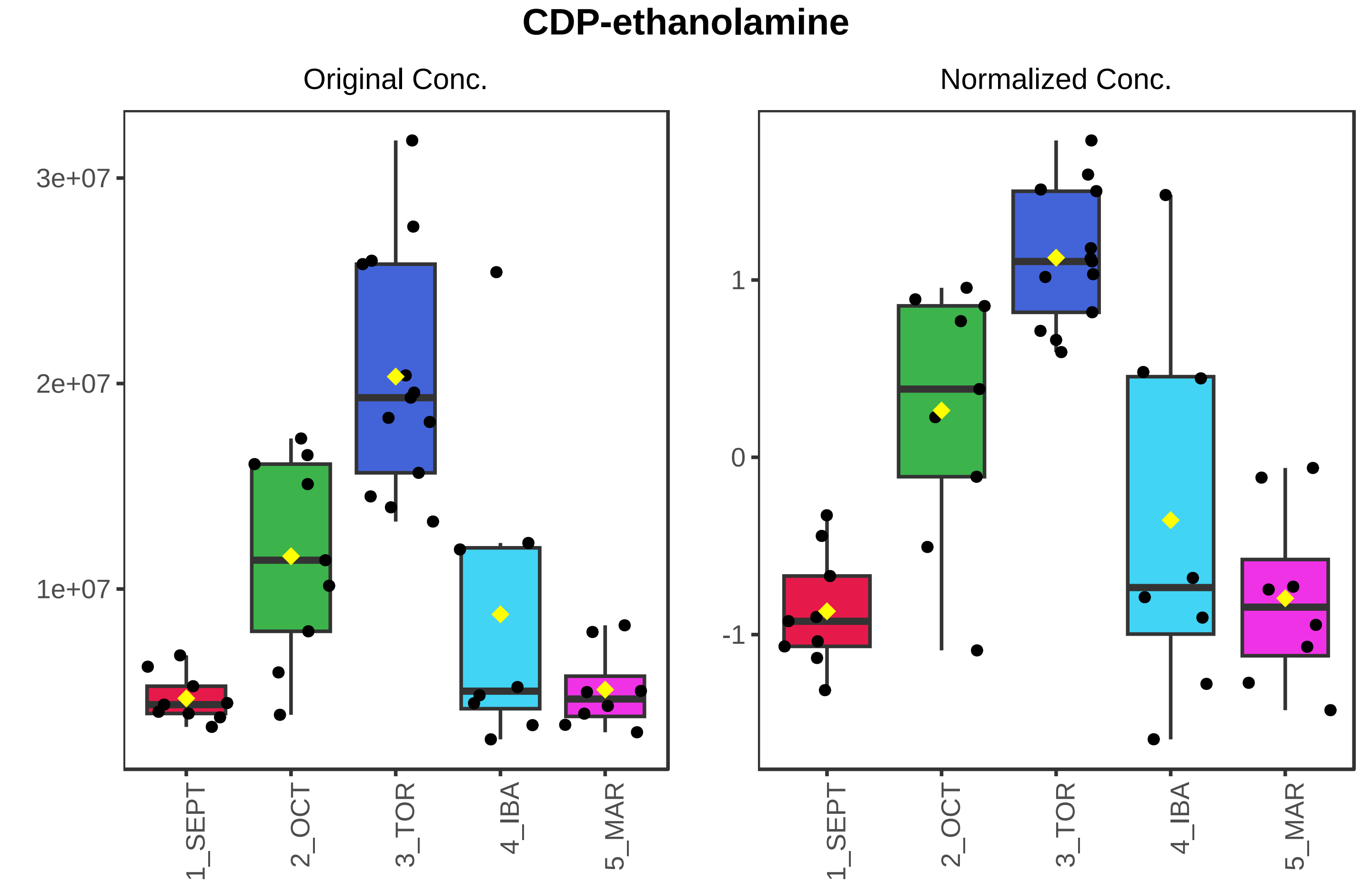
CDP-ethanolamine
Original Conc.
Normalized Conc.
3e+07
2e+07
1e+07
1
0
-1
1_SEPT
2_OCT
3_TOR
4_IBA
5_MAR
1_SEPT
2_OCT
3_TOR
4_IBA
5_MAR

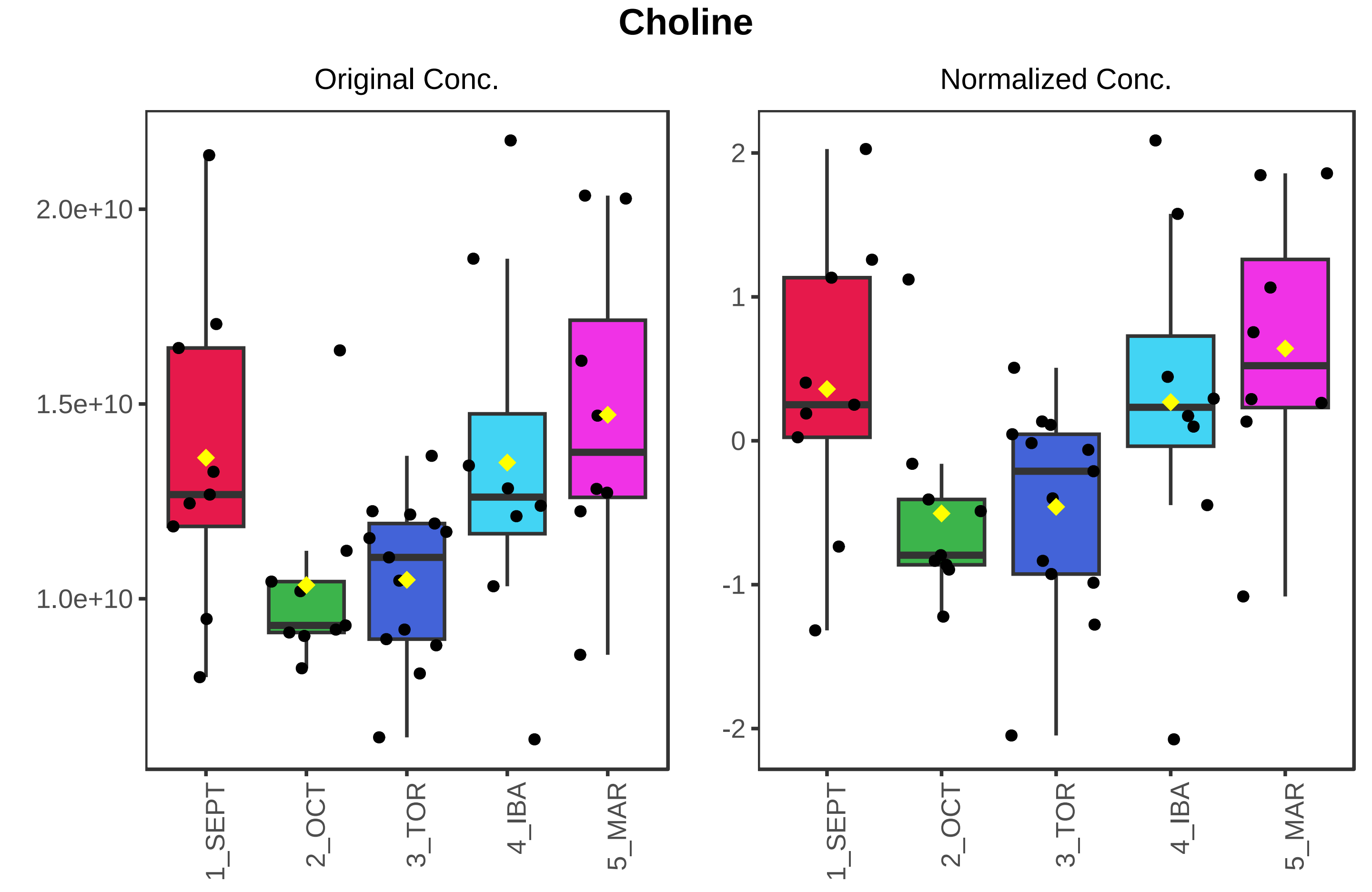
Choline
Original Conc.
Normalized Conc.
2.0e+10
1.5e+10
1.0e+10
2
1
0
-1
-2
1_SEPT
2_OCT
3_TOR
4_IBA
5_MAR
1_SEPT
2_OCT
3_TOR
4_IBA
5_MAR

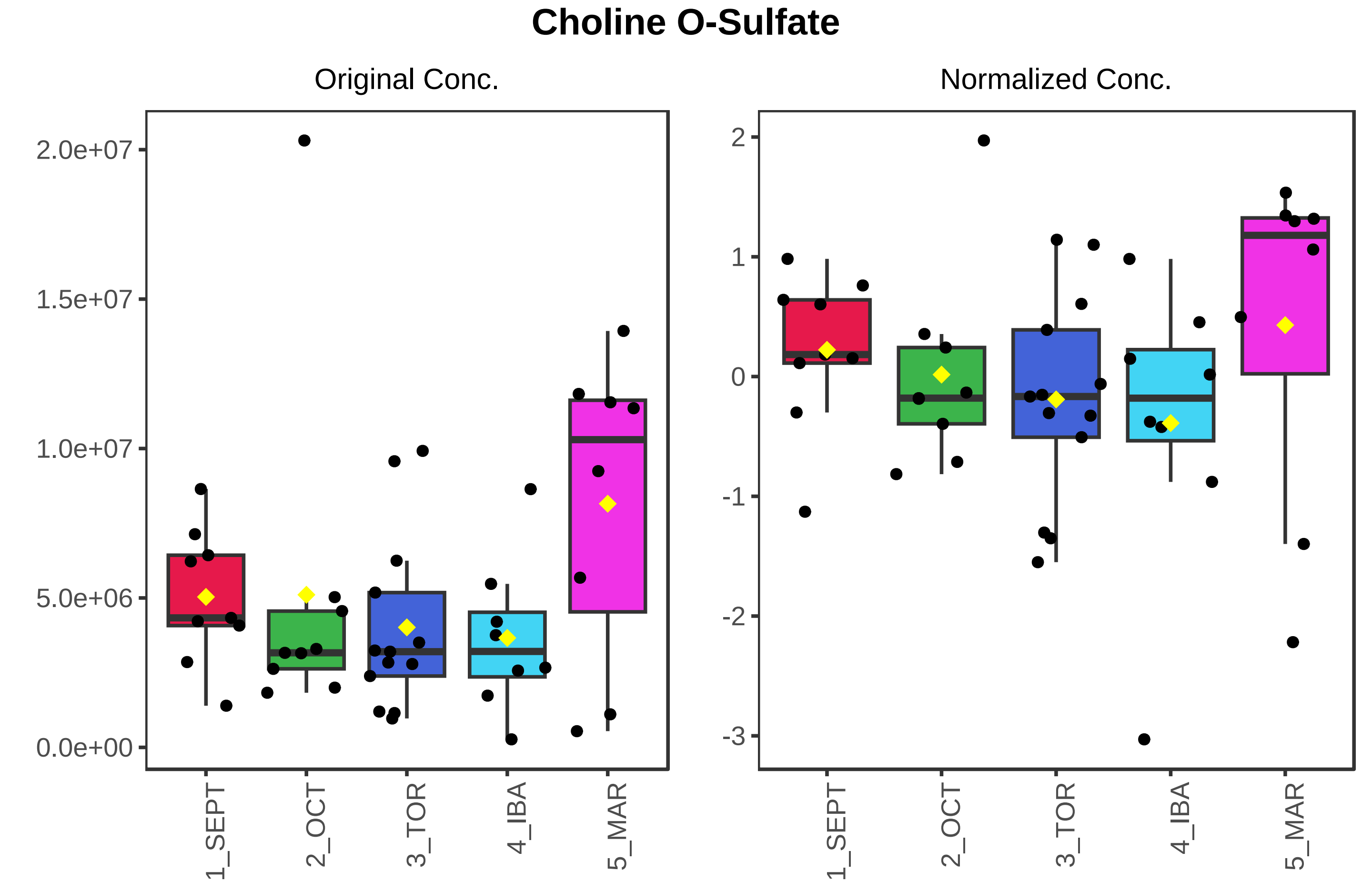

Choline O-Sulfate
Original Conc.
Normalized Conc.
2.0e+07
1.5e+07
1.0e+07
5.0e+06
0.0e+00
2
1
0
-1
-2
-3
1_SEPT
2_OCT
3_TOR
4_IBA
5_MAR

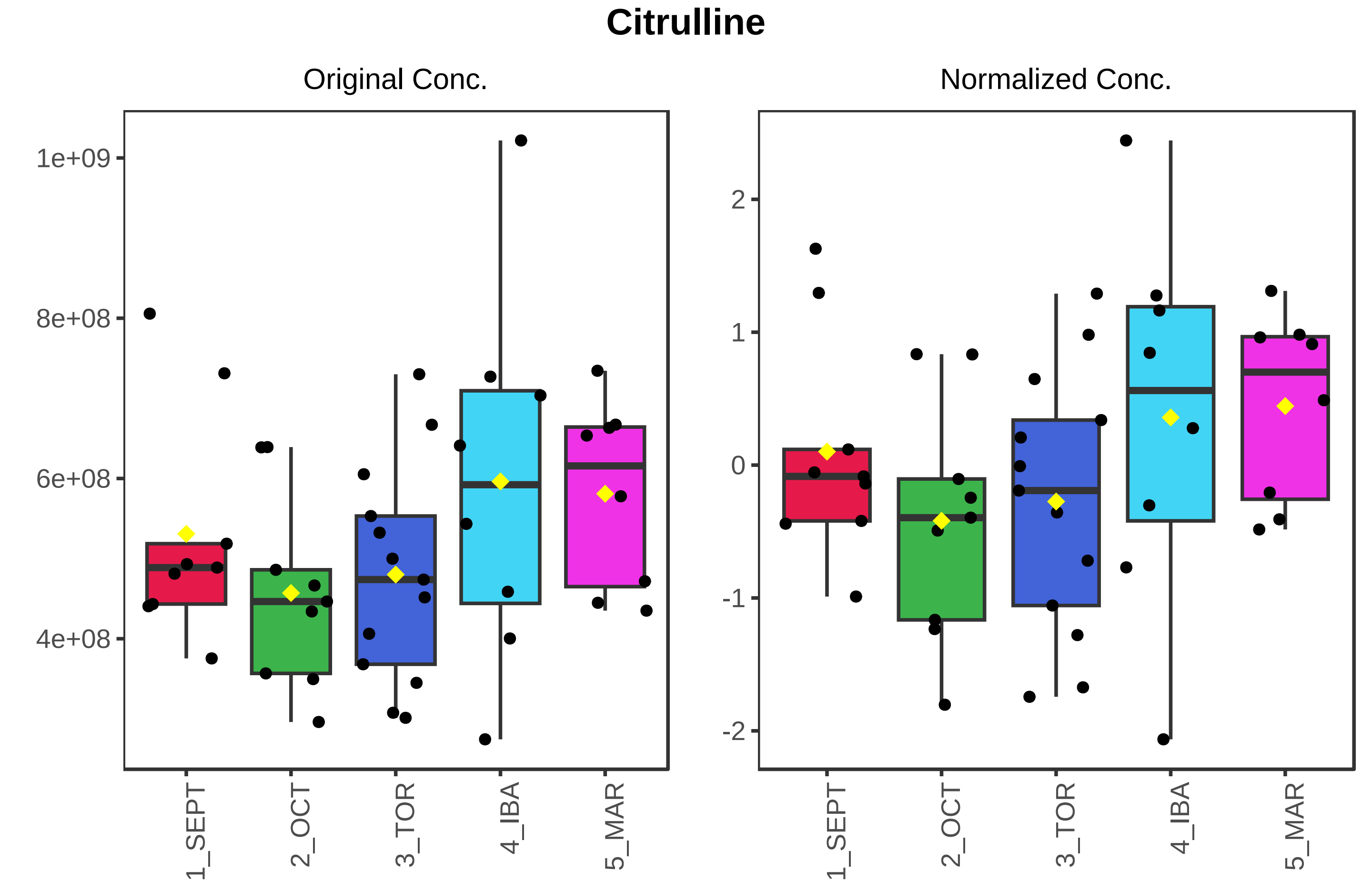

Citrulline
Original Conc.
Normalized Conc.
1e+09
8e+08
6e+08
4e+08
2
1
0
-1
-2
1_SEPT
2_OCT
3_TOR
4_IBA
5_MAR

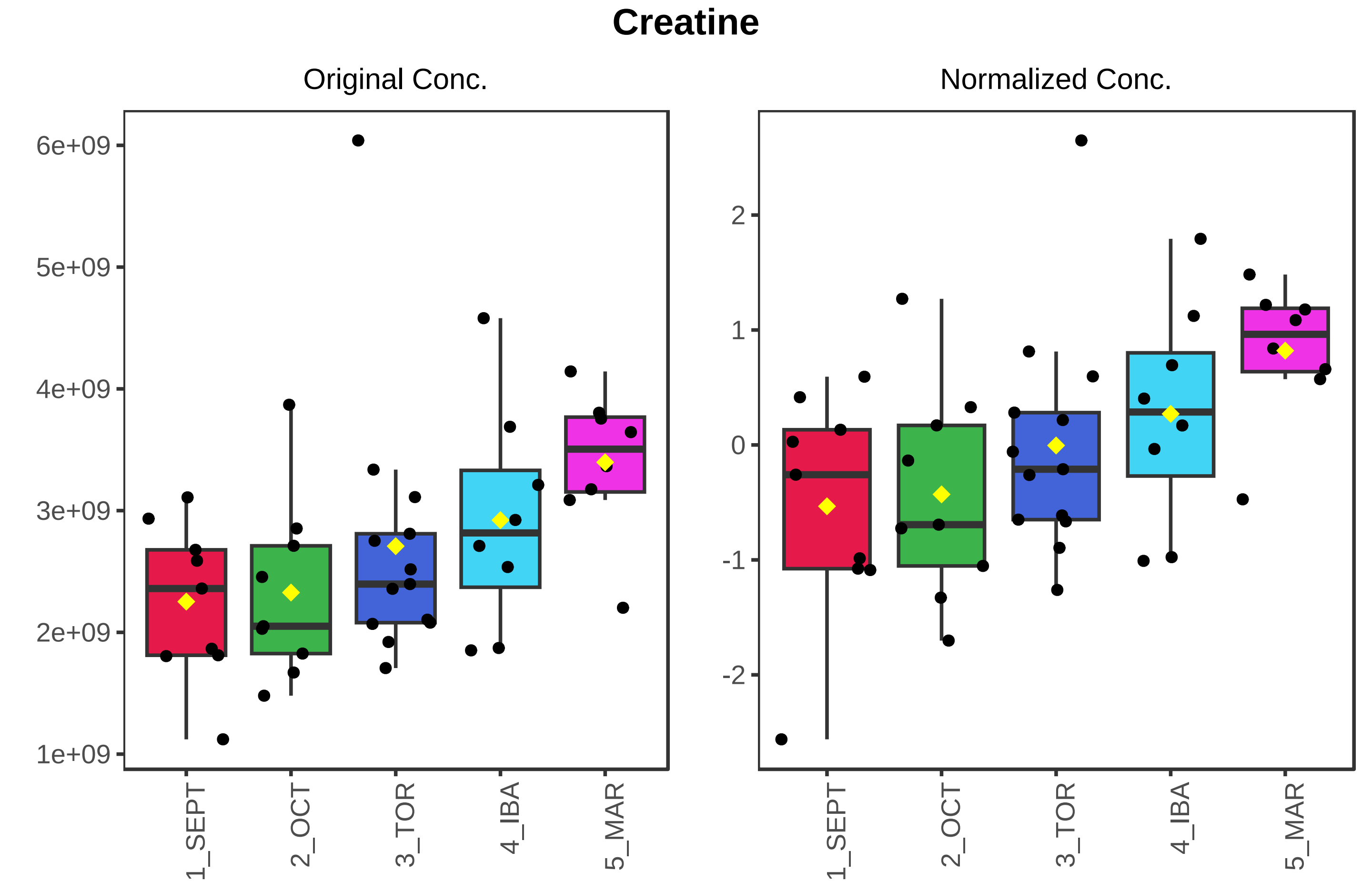

Creatine
Original Conc.
Normalized Conc.
6e+09
5e+09
4e+09
3e+09
2e+09
1e+09
2
1
0
-1
-2
1_SEPT
2_OCT
3_TOR
4_IBA
5_MAR

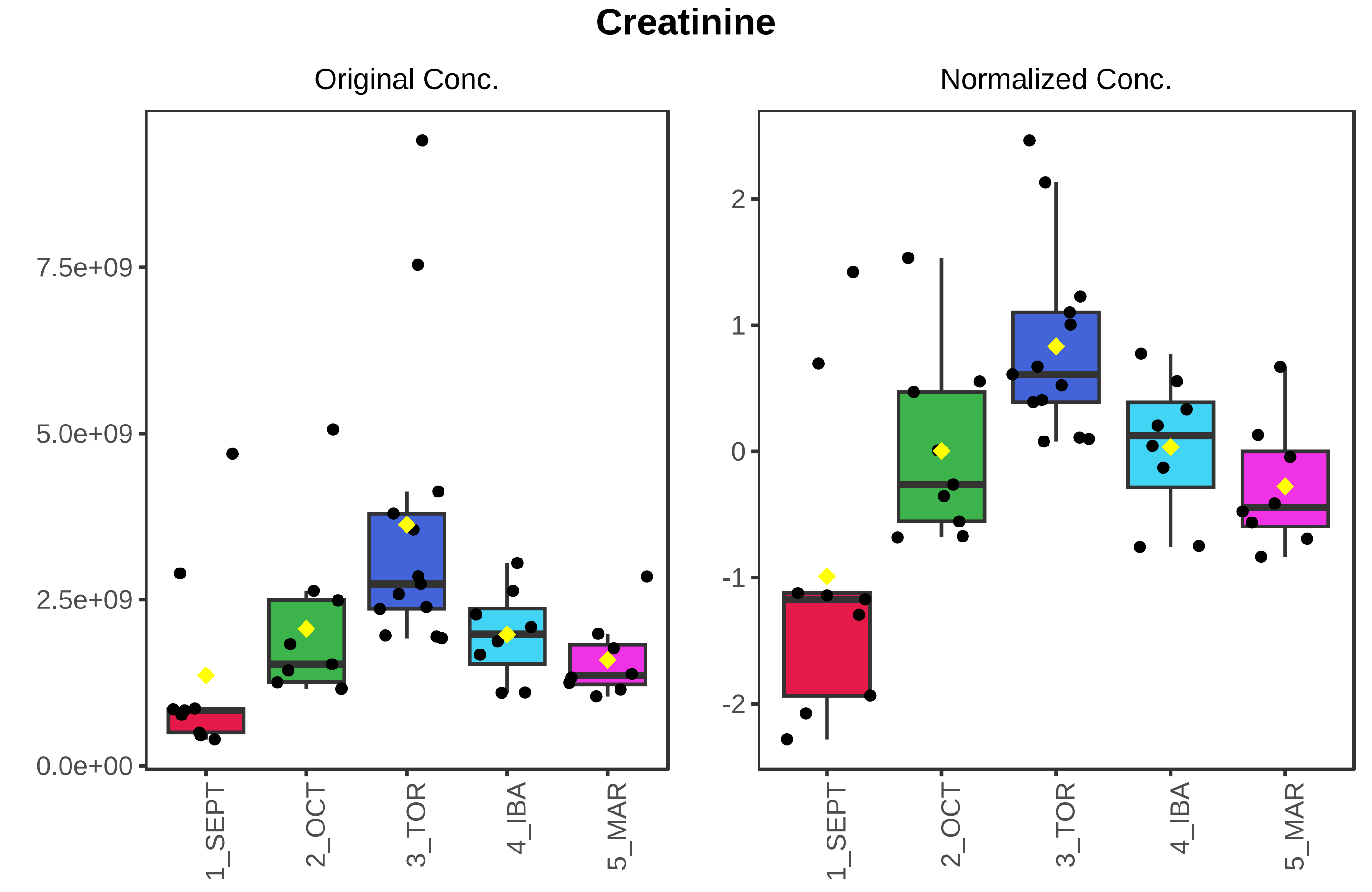

Creatinine
Original Conc.
Normalized Conc.
7.5e+09
5.0e+09
2.5e+09
0.0e+00
2
1
0
-1
-2
1_SEPT
2_OCT
3_TOR
4_IBA
5_MAR

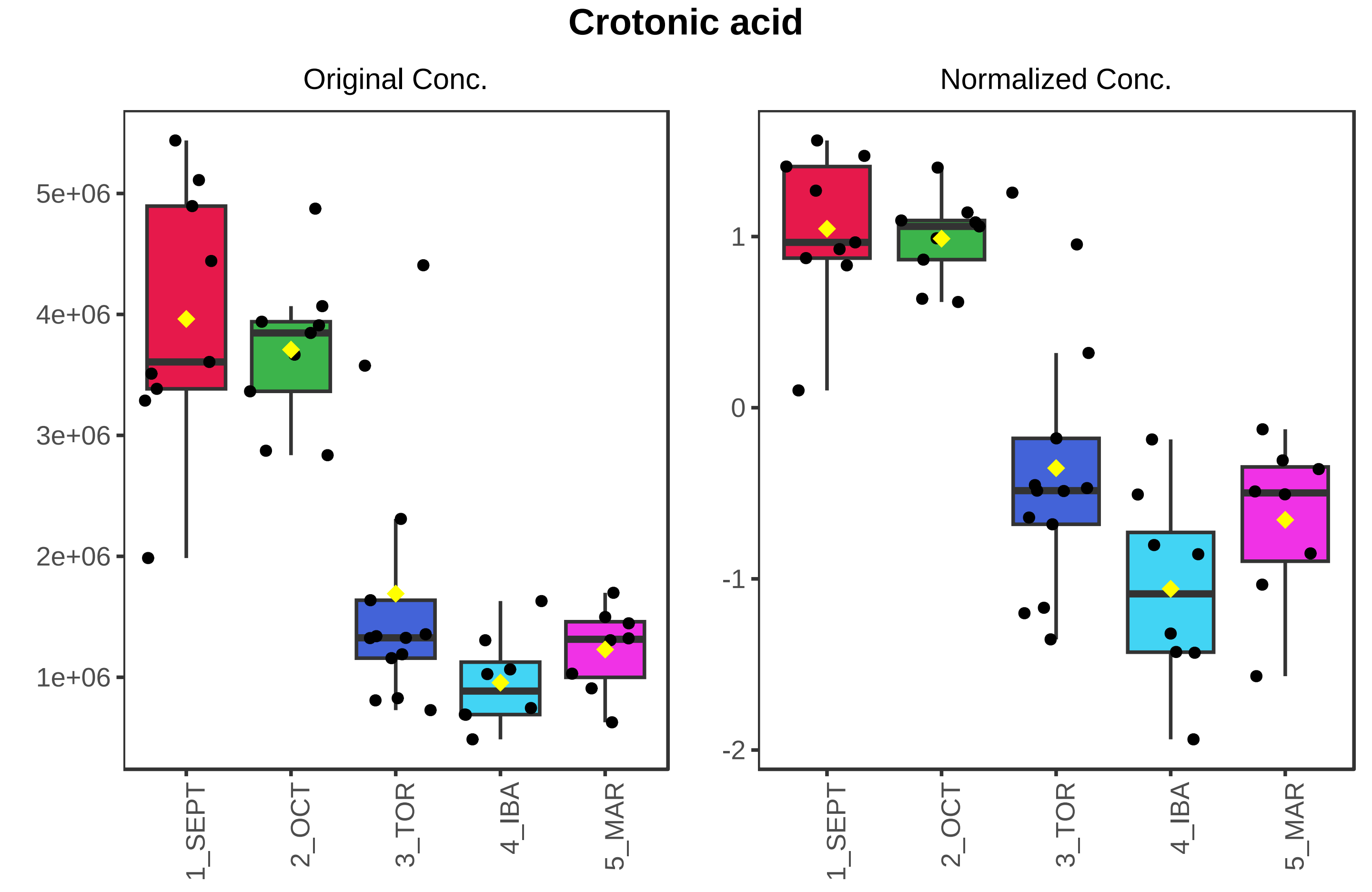

Crotonic acid
Original Conc.
Normalized Conc.
5e+06
4e+06
3e+06
2e+06
1e+06
1
0
-1
-2
1_SEPT
2_OCT
3_TOR
4_IBA
5_MAR

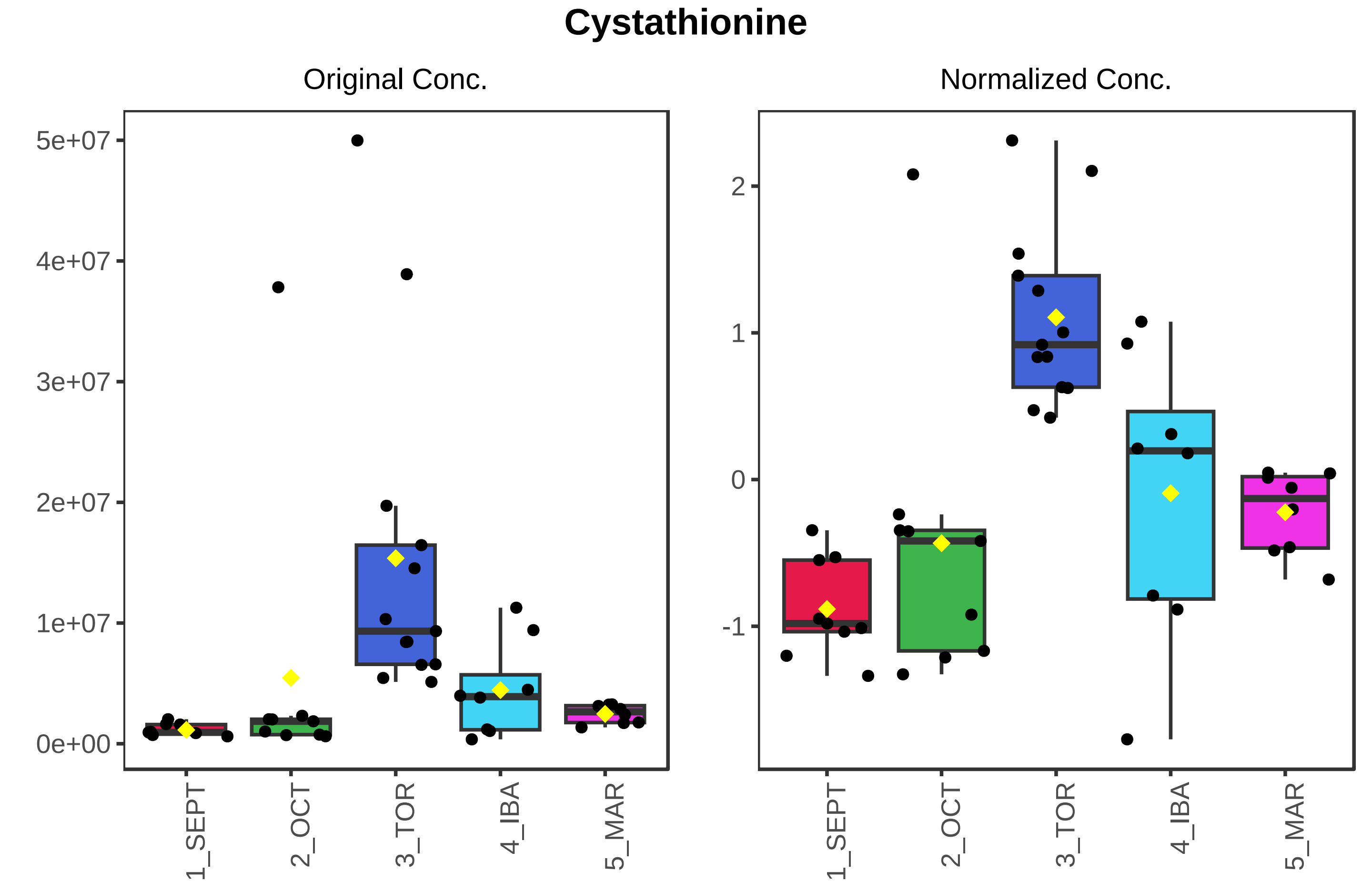

Cystathionine
Original Conc.
Normalized Conc.
5e+07
4e+07
3e+07
2e+07
1e+07
0e+00
2
1
0
-1
1_SEPT
2_OCT
3_TOR
4_IBA
5_MAR

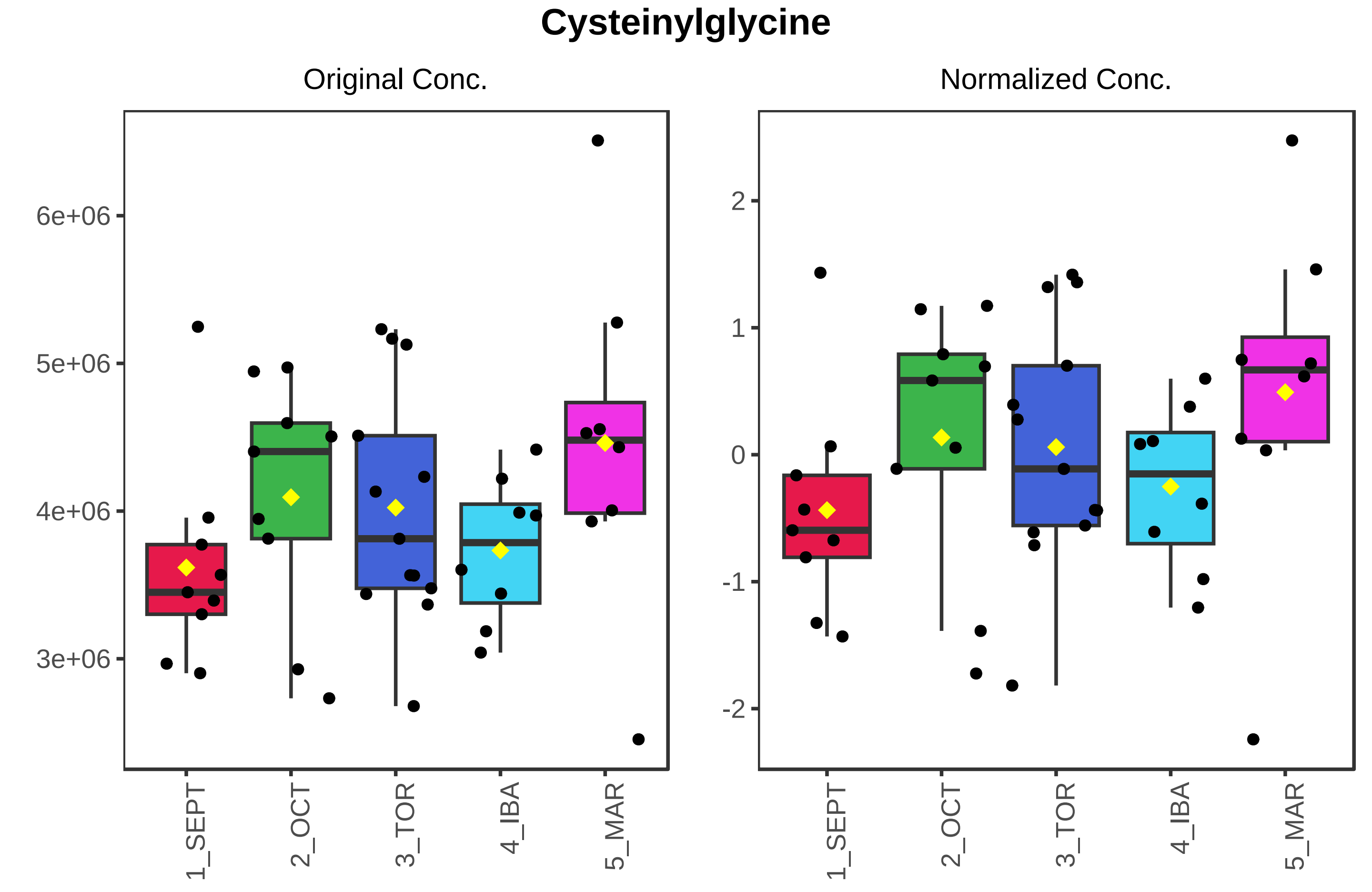

Cysteinylglycine
Original Conc.
Normalized Conc.
6e+06
5e+06
4e+06
3e+06
2
1
0
-1
-2
1_SEPT
2_OCT
3_TOR
4_IBA
5_MAR

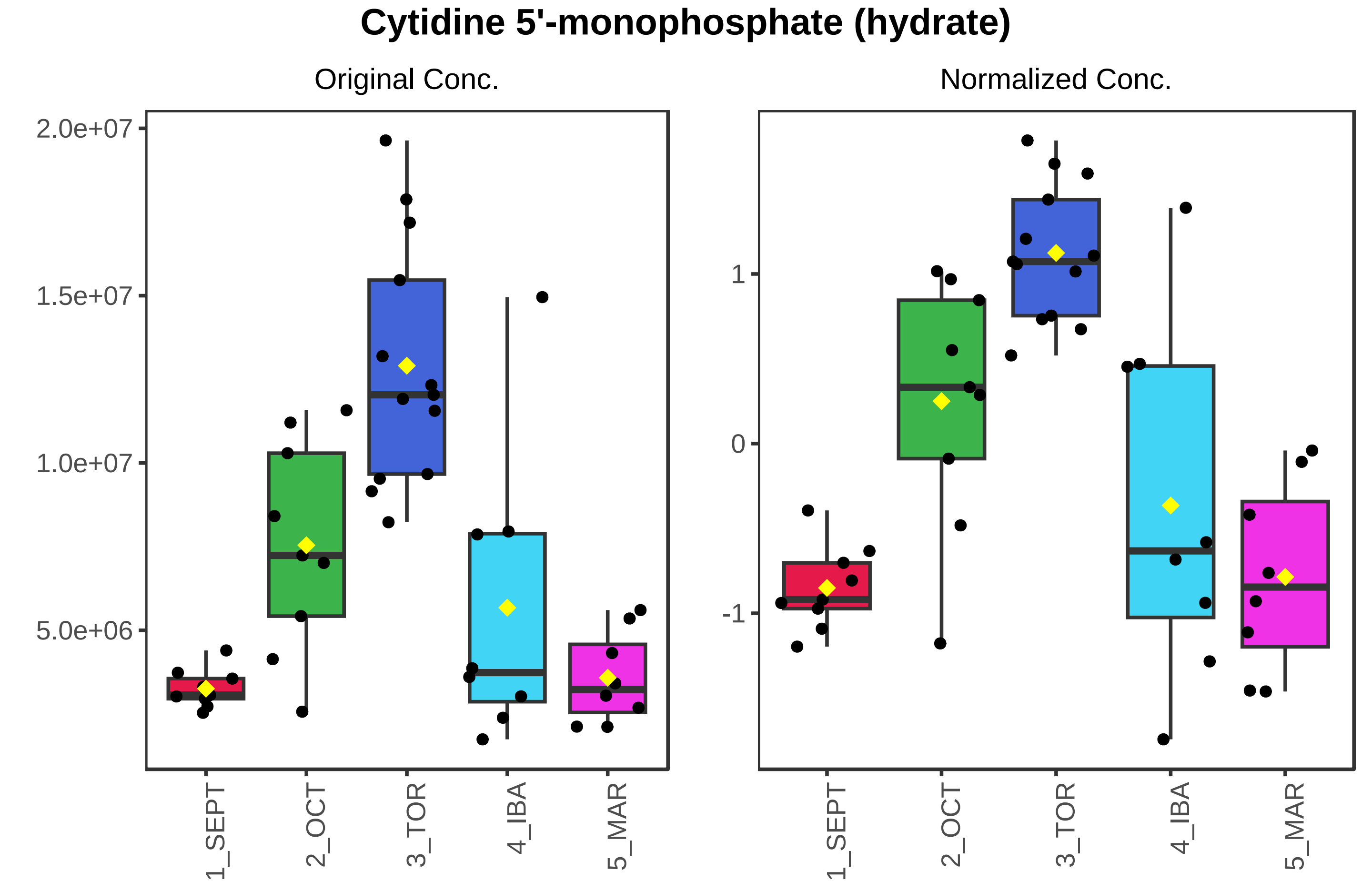

Cytidine 5'-monophosphate (hydrate)
Original Conc.
Normalized Conc.
2.0e+07
1.5e+07
1.0e+07
5.0e+06
1
0
-1
1_SEPT
2_OCT
3_TOR
4_IBA
5_MAR

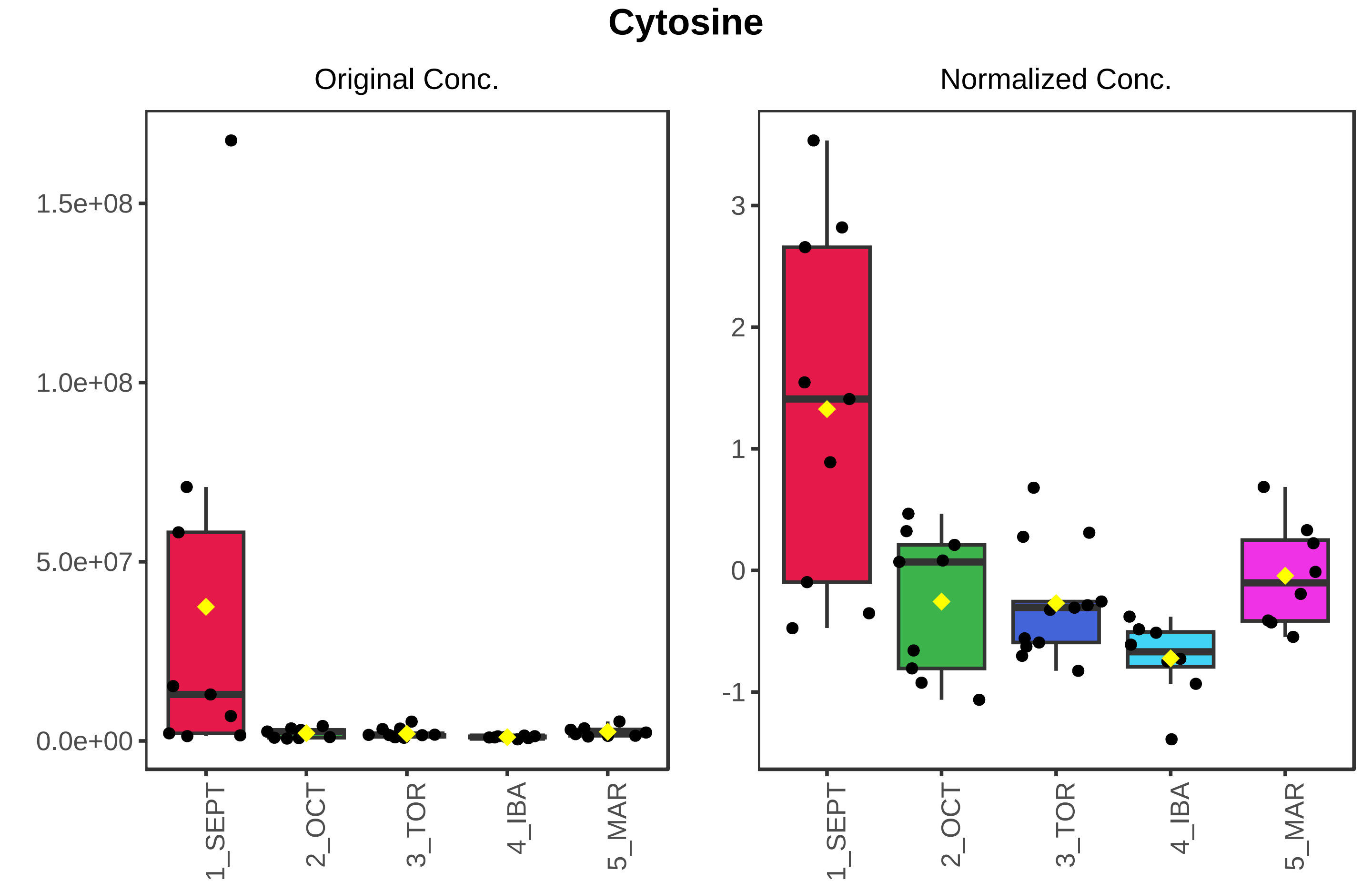

Cytosine
Original Conc.
Normalized Conc.
1.5e+08
1.0e+08
5.0e+07
0.0e+00
3
2
1
0
-1
1_SEPT
2_OCT
3_TOR
4_IBA
5_MAR

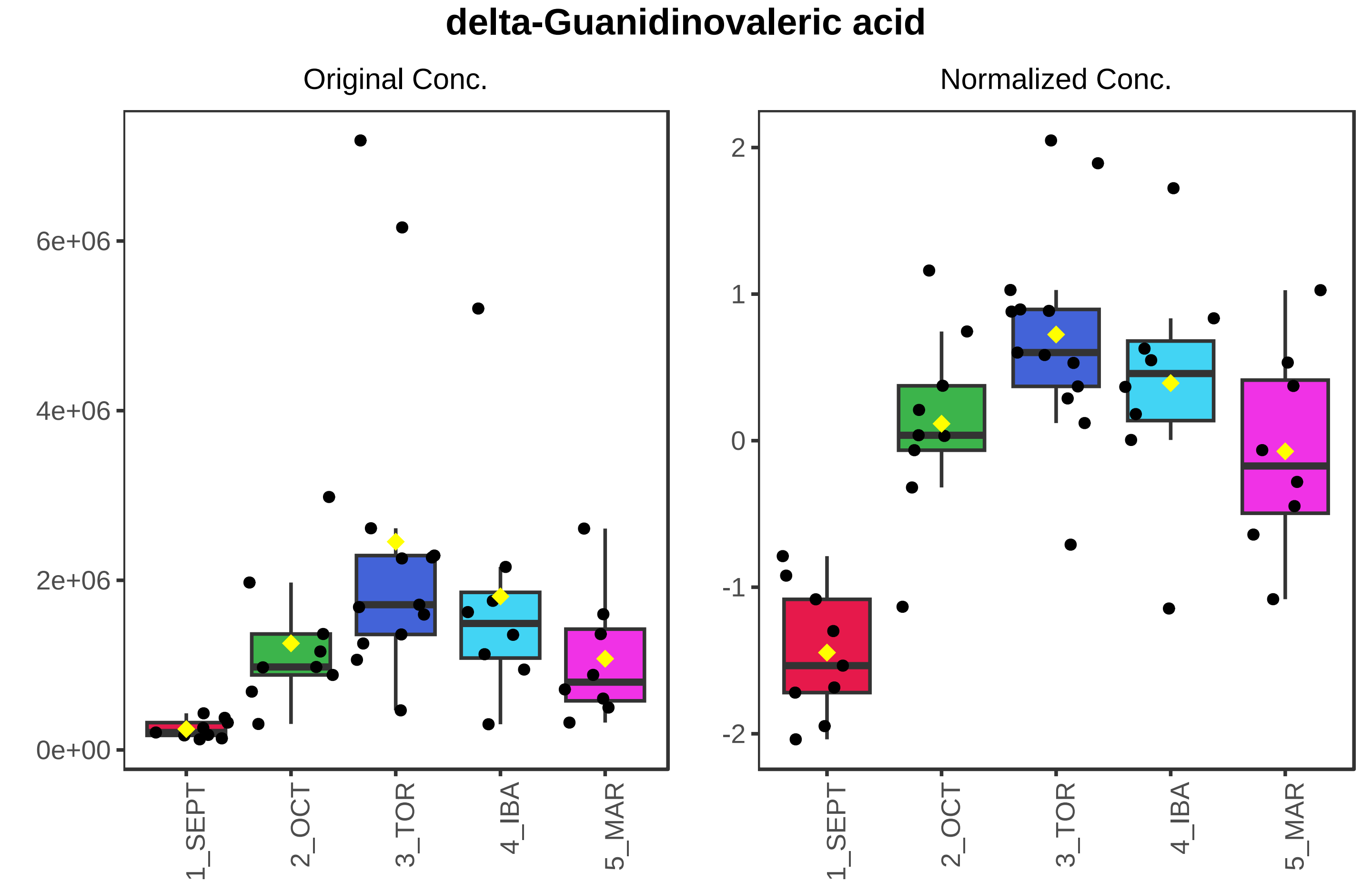

delta-Guanidinovaleric acid
Original Conc.
Normalized Conc.
6e+06
4e+06
2e+06
0e+00
2
1
0
-1
-2
1_SEPT
2_OCT
3_TOR
4_IBA
5_MAR

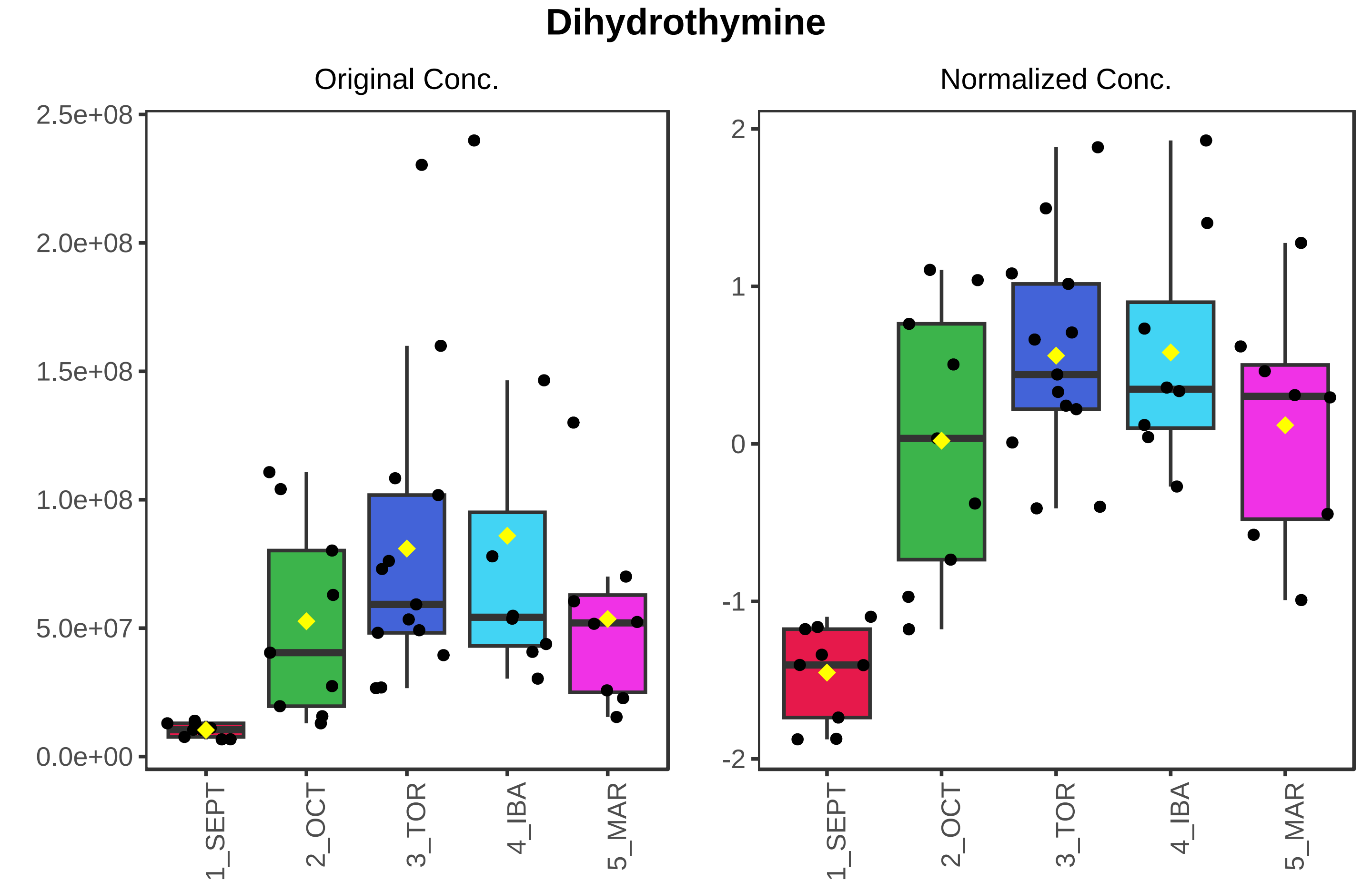

Dihydrothymine
Original Conc.
Normalized Conc.
2.5e+08
2.0e+08
1.5e+08
1.0e+08
5.0e+07
0.0e+00
2
1
0
-1
-2
1_SEPT
2_OCT
3_TOR
4_IBA
5_MAR

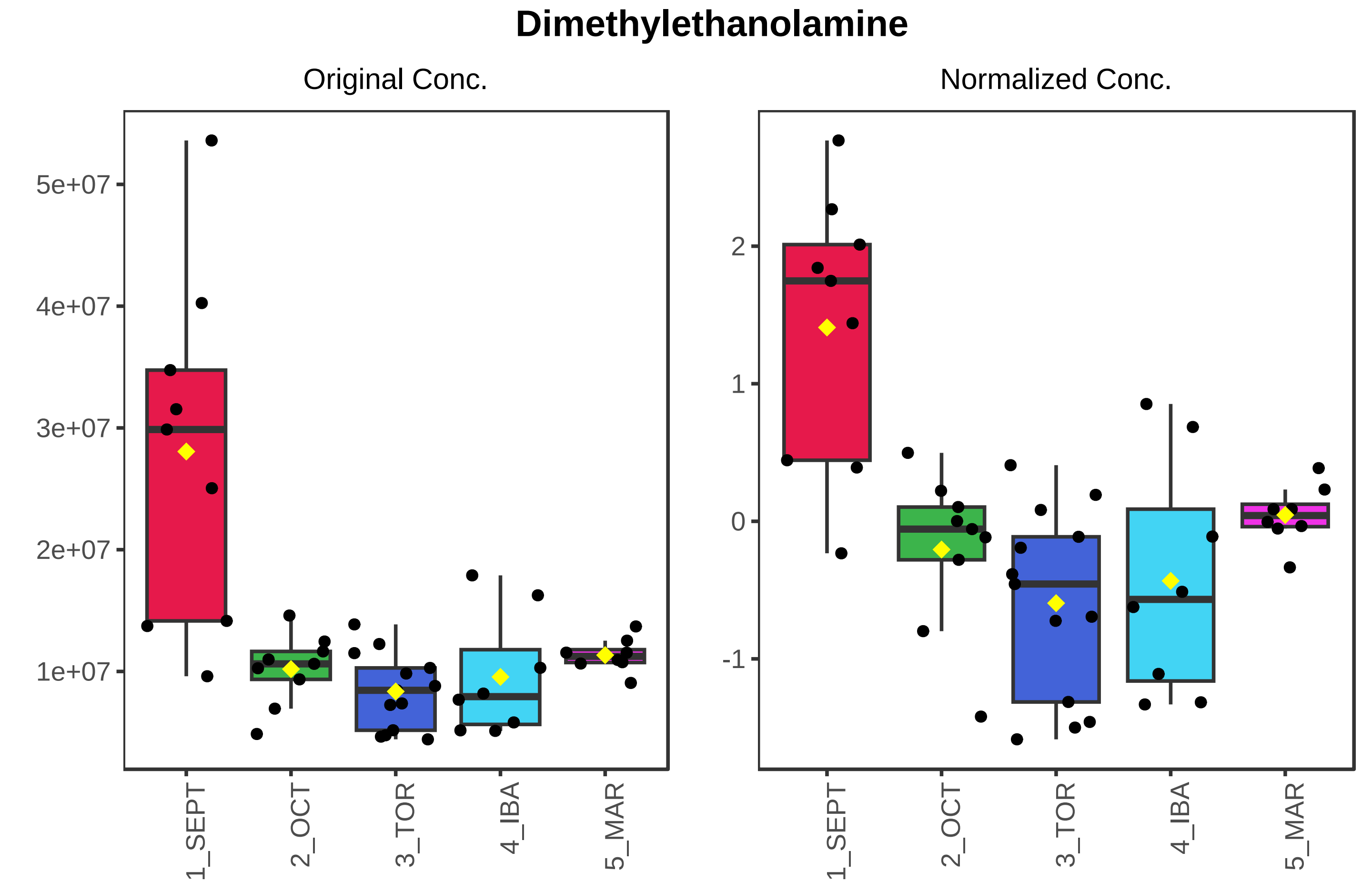
Dimethylethanolamine
Original Conc.
Normalized Conc.
5e+07
4e+07
3e+07
2e+07
1e+07
2
1
0
-1
1_SEPT
2_OCT
3_TOR
4_IBA
5_MAR
1_SEPT
2_OCT
3_TOR
4_IBA
5_MAR

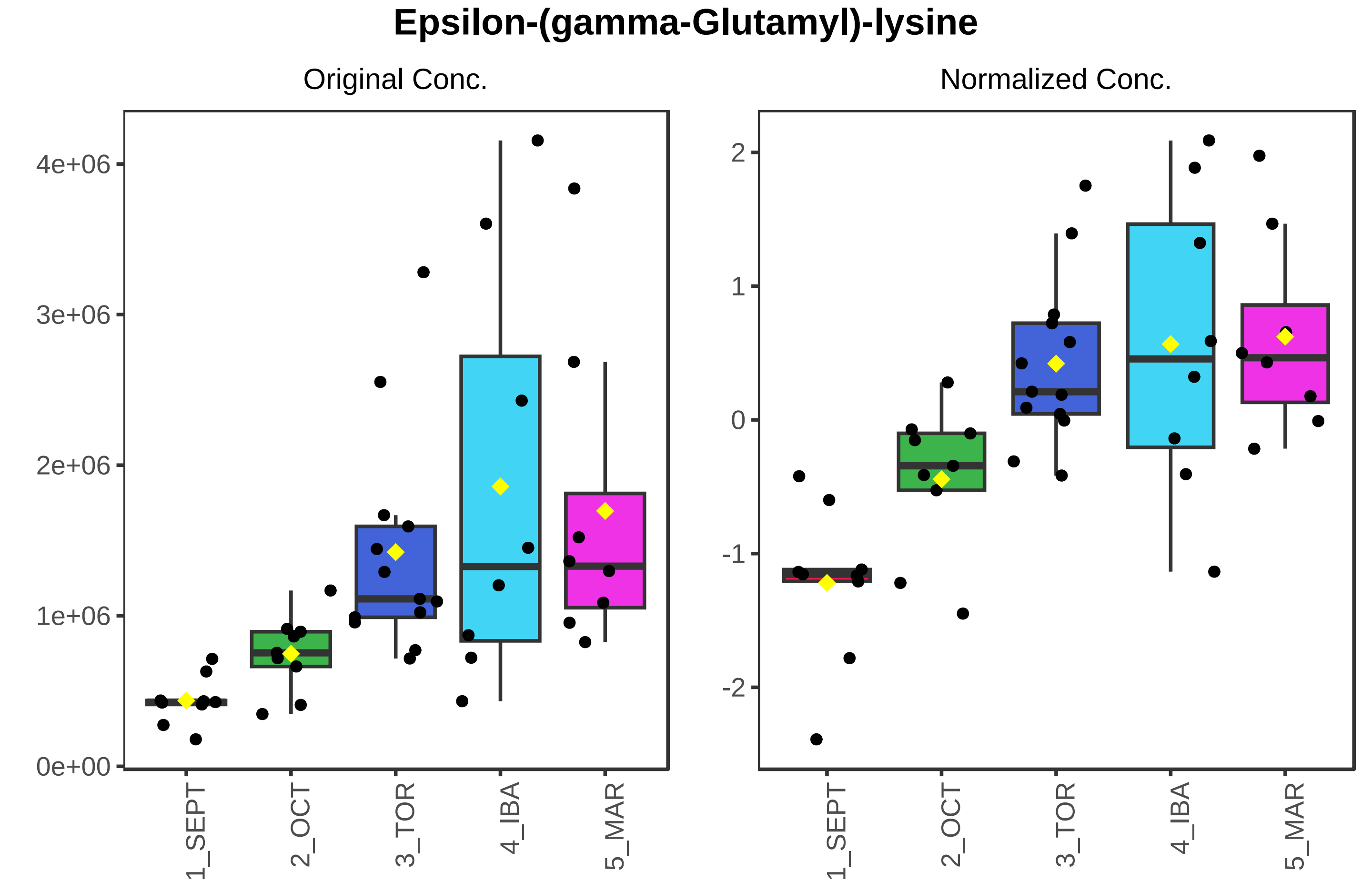

Epsilon-(gamma-Glutamyl)-lysine
Original Conc.
Normalized Conc.
4e+06
3e+06
2e+06
1e+06
0e+00
2
1
0
-1
-2
1_SEPT
2_OCT
3_TOR
4_IBA
5_MAR

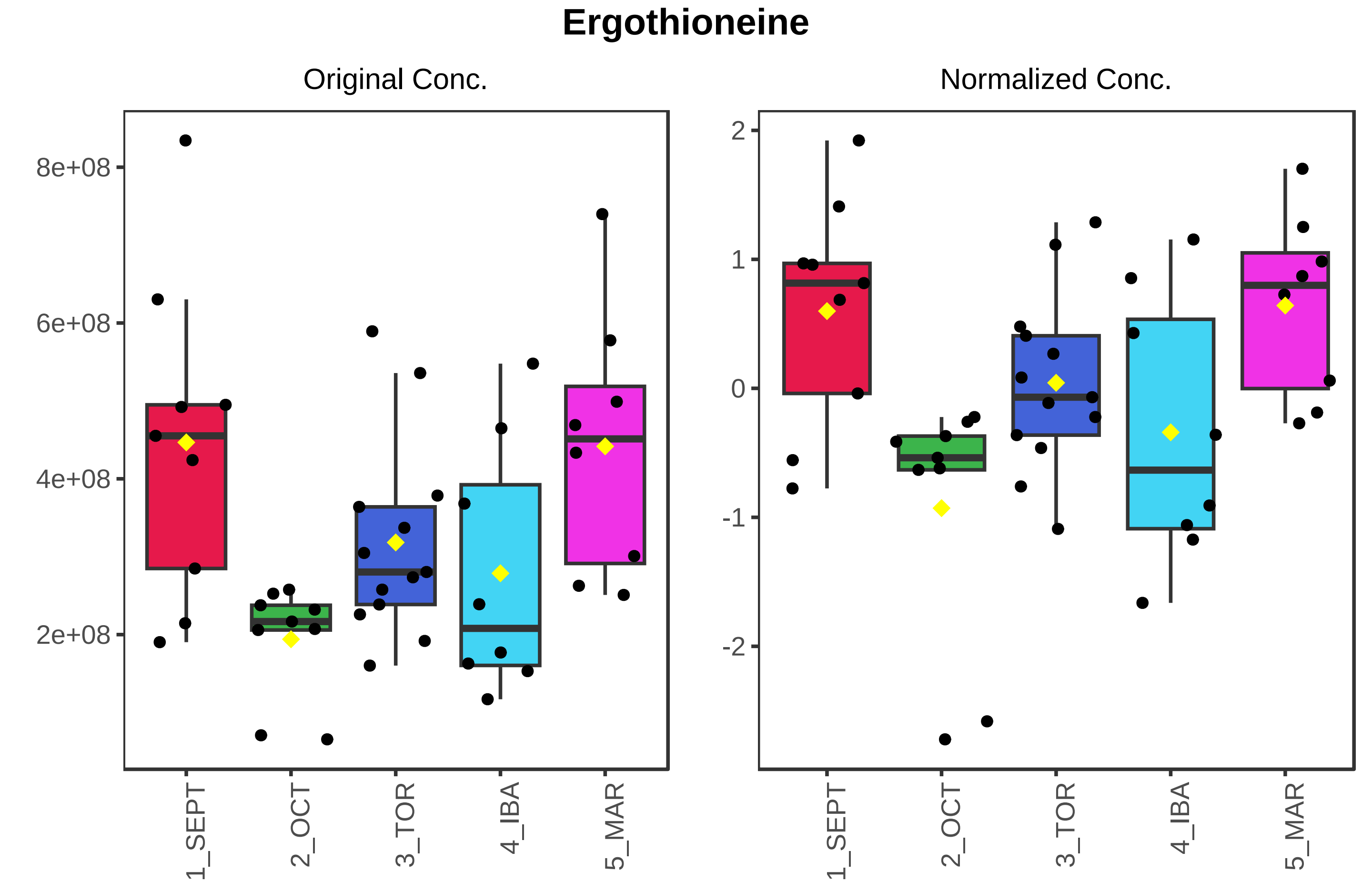

Ergothioneine
Original Conc.
Normalized Conc.
8e+08
6e+08
4e+08
2e+08
2
1
0
-1
-2
1_SEPT
2_OCT
3_TOR
4_IBA
5_MAR
1_SEPT
2_OCT
3_TOR
4_IBA
5_MAR

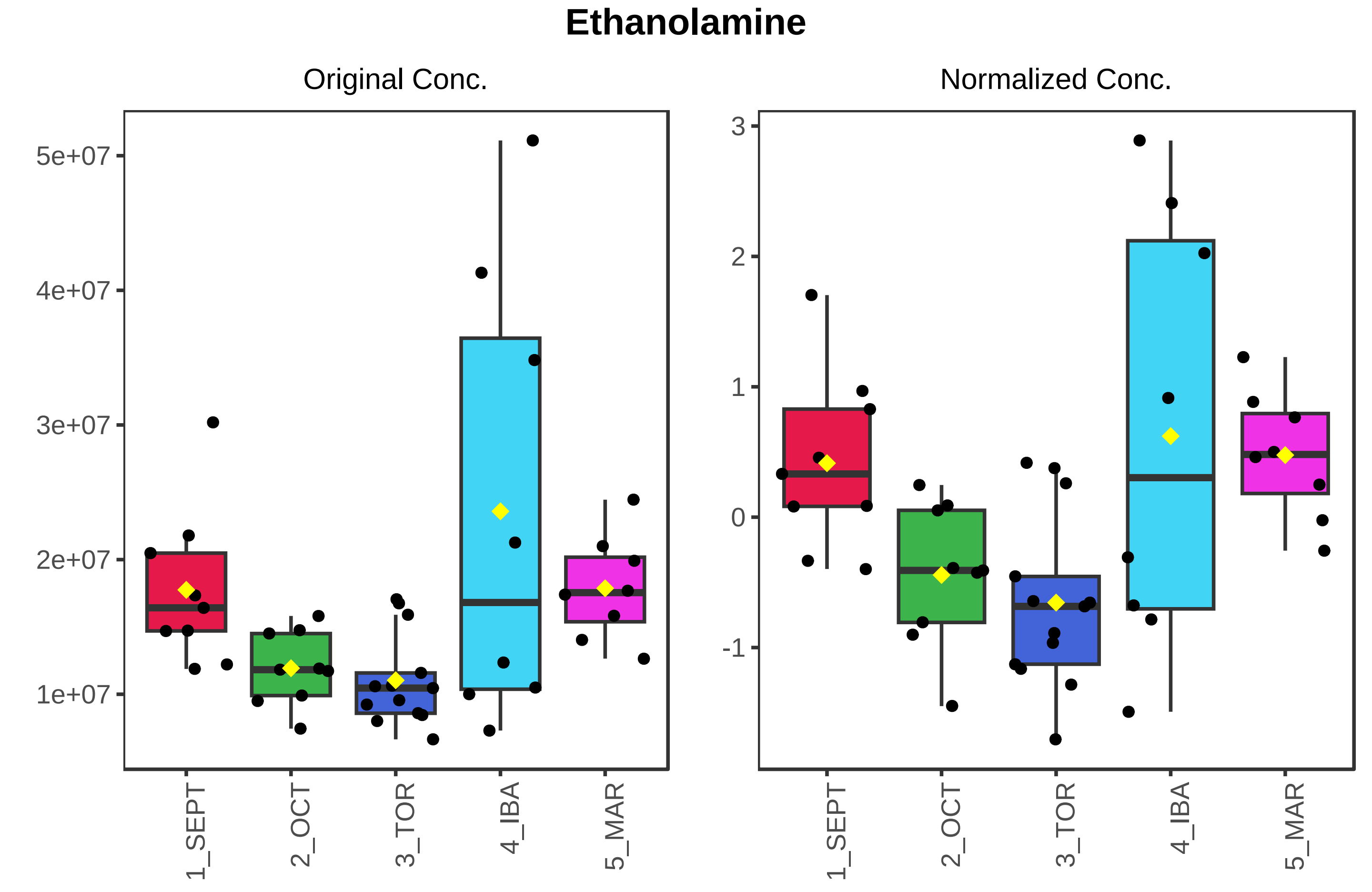
Ethanolamine
Original Conc.
Normalized Conc.
5e+07
4e+07
3e+07
2e+07
1e+07
3
2
1
0
-1
1_SEPT
2_OCT
3_TOR
4_IBA
5_MAR

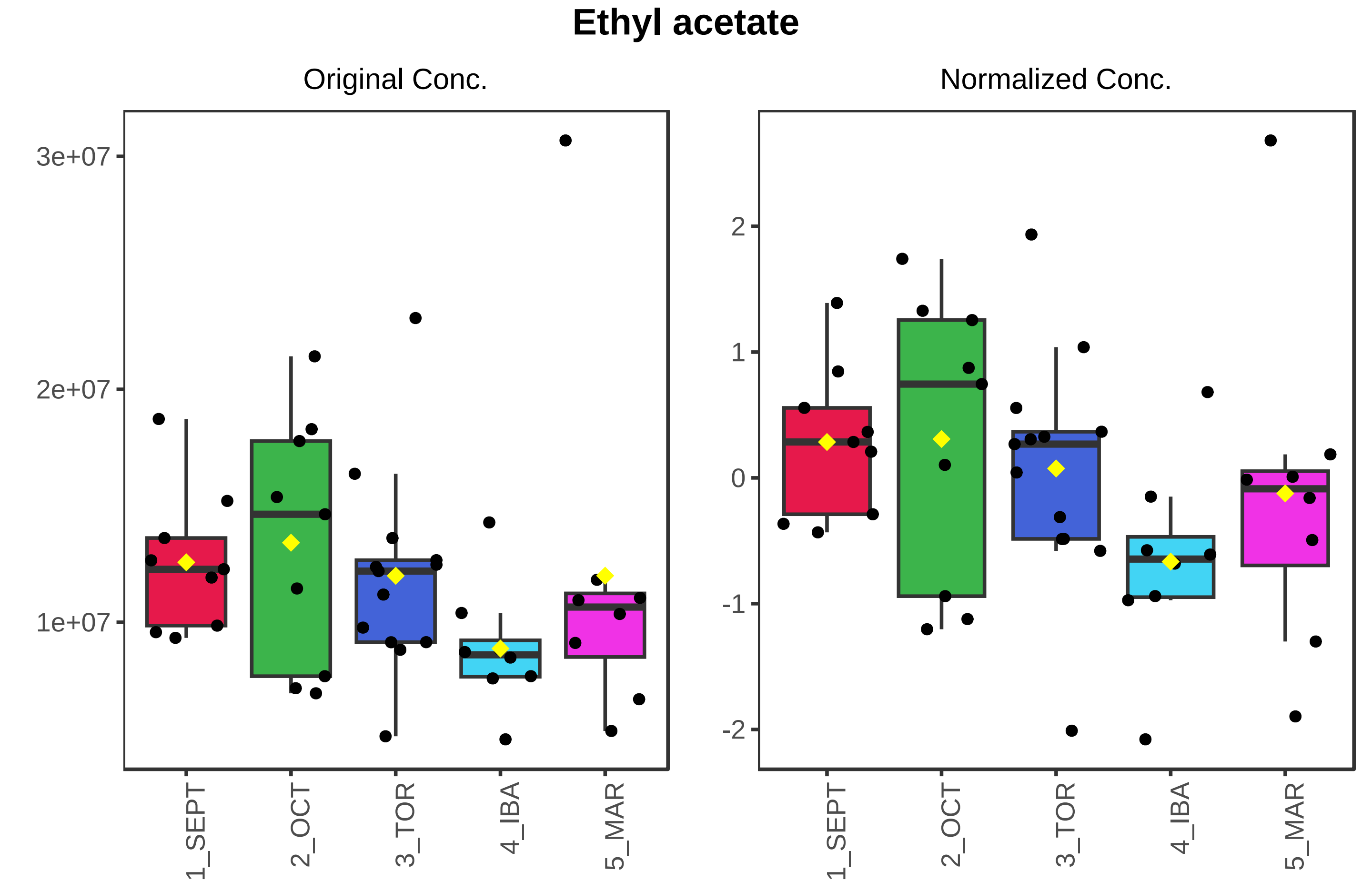

Ethyl acetate
Original Conc.
Normalized Conc.
3e+07
2e+07
1e+07
2
1
0
-1
-2
1_SEPT
2_OCT
3_TOR
4_IBA
5_MAR
1_SEPT
2_OCT
3_TOR
4_IBA
5_MAR

Formiminoglutamic Acid

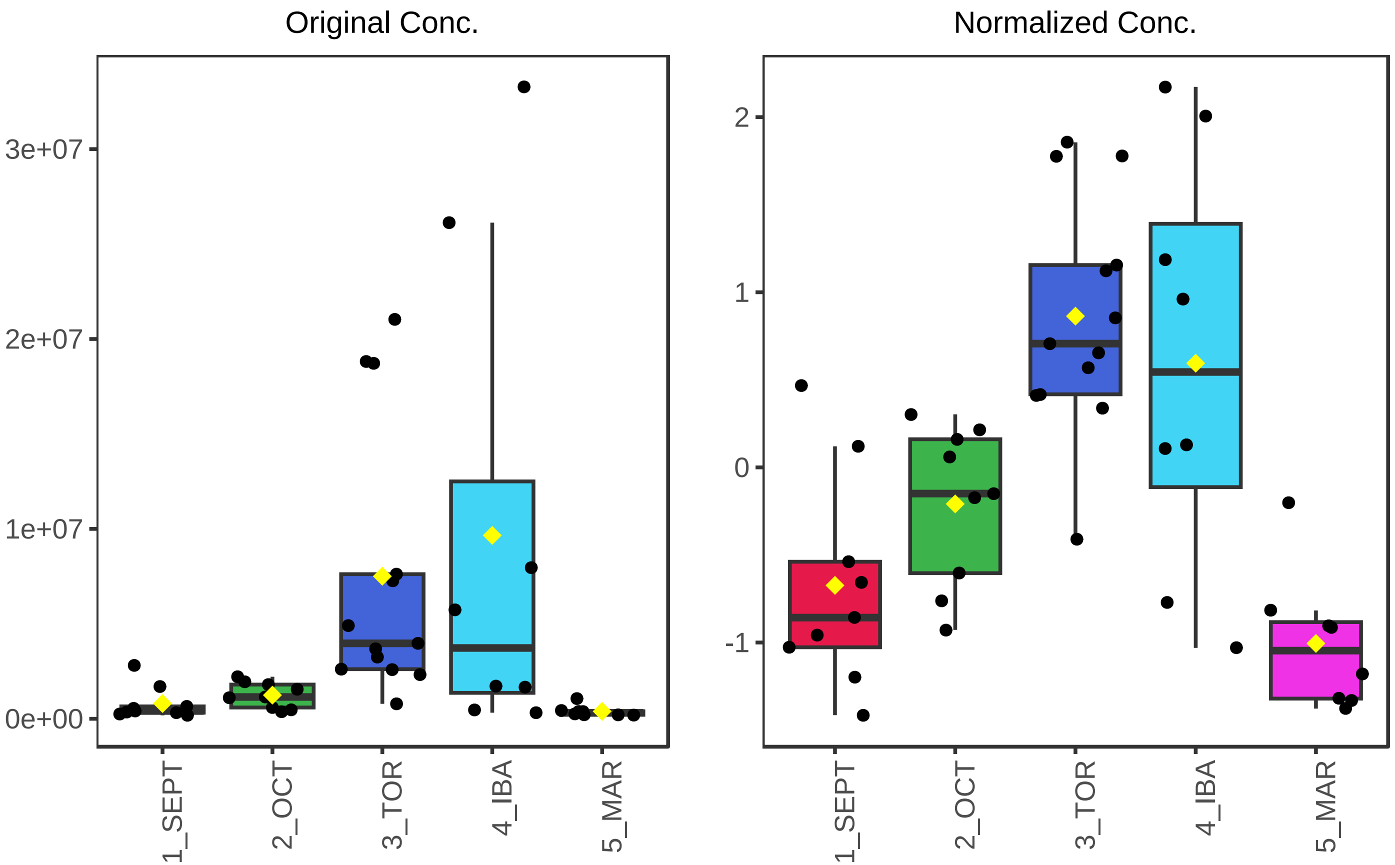

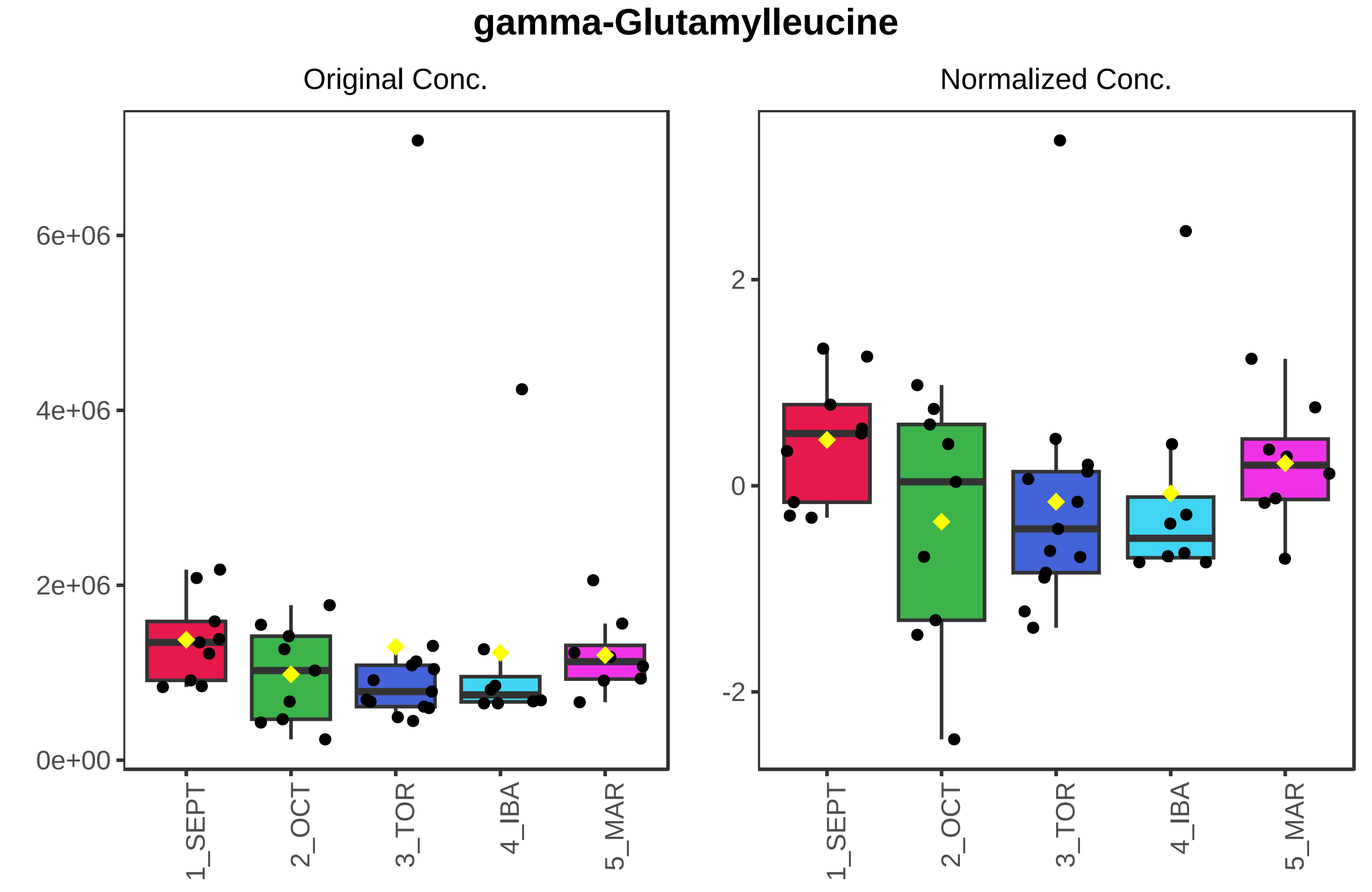
gamma-Glutamylleucine
Original Conc.
Normalized Conc.
6e+06
4e+06
2e+06
0e+00
2
0
-2
1_SEPT
2_OCT
3_TOR
4_IBA
5_MAR
1_SEPT
2_OCT
3_TOR
4_IBA
5_MAR

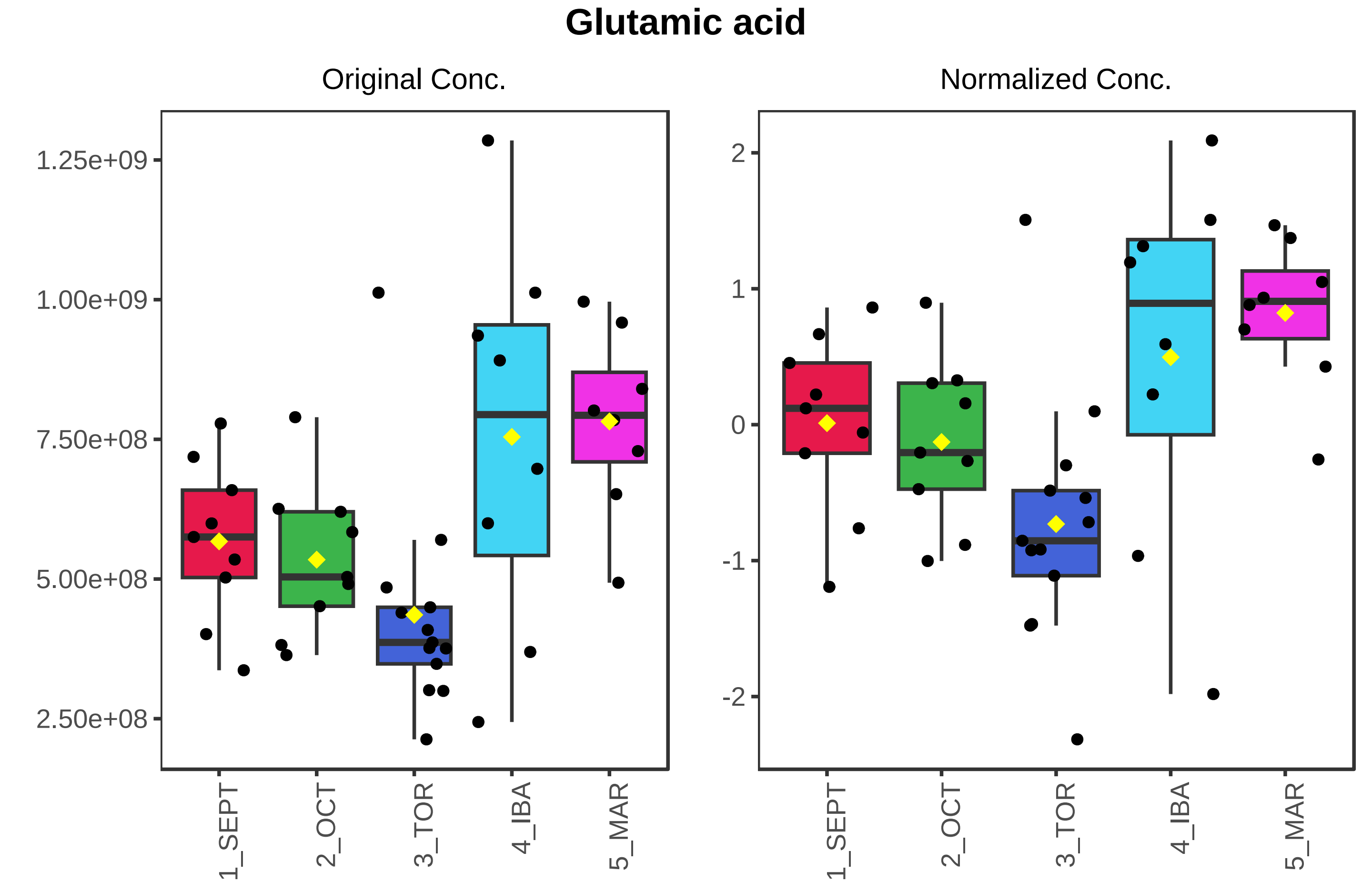

Glutamic acid
Original Conc.
Normalized Conc.
1.25e+09
1.00e+09
7.50e+08
5.00e+08
2.50e+08
2
1
0
-1
-2
1_SEPT
2_OCT
3_TOR
4_IBA
5_MAR

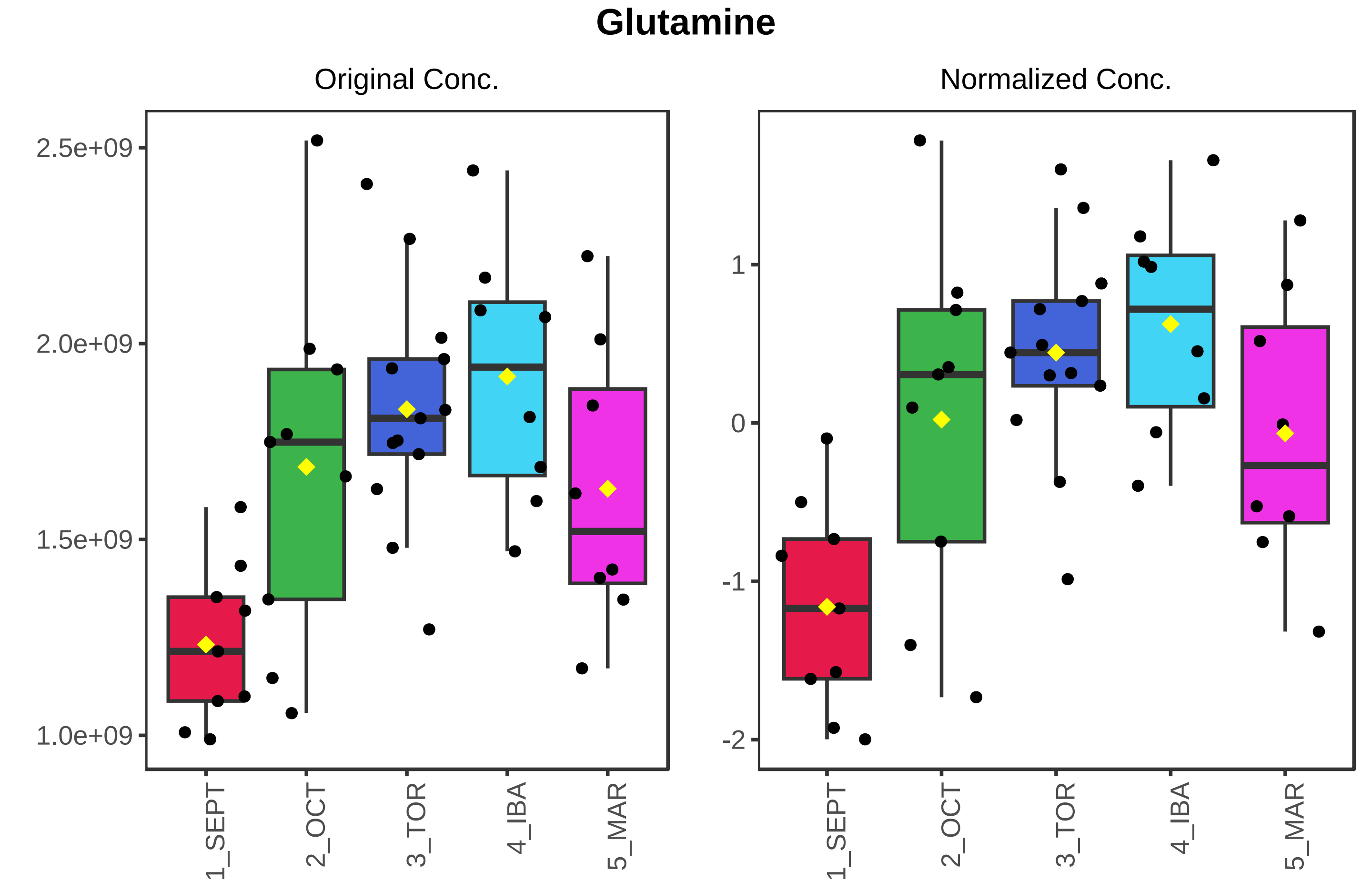

Glutamine
Original Conc.
Normalized Conc.
2.5e+09
2.0e+09
1.5e+09
1.0e+09
1
0
-1
-2
1_SEPT
2_OCT
3_TOR
4_IBA
5_MAR

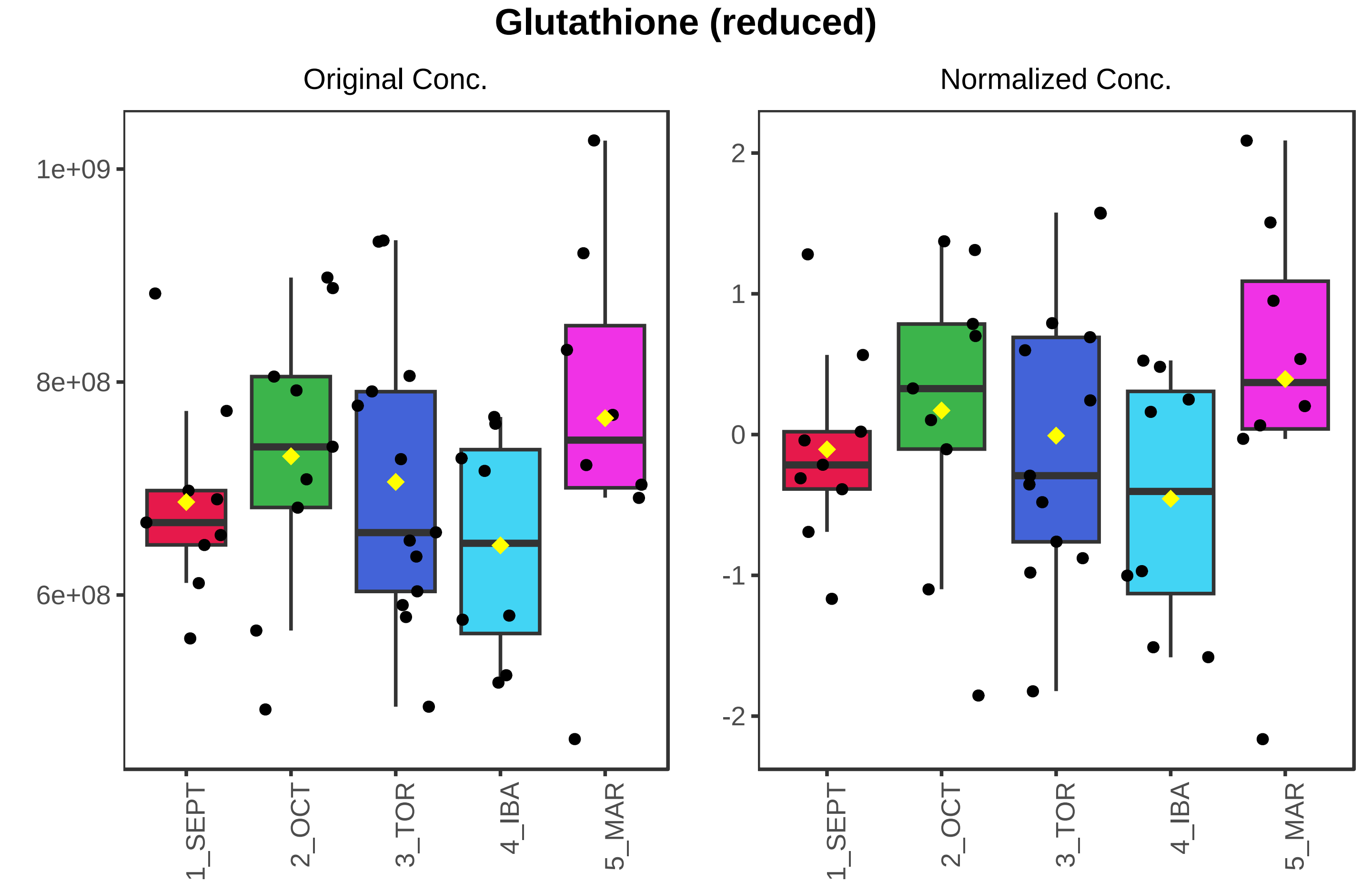

Glutathione (reduced)
Original Conc.
Normalized Conc.
1e+09
8e+08
6e+08
2
1
0
-1
-2
1_SEPT
2_OCT
3_TOR
4_IBA
5_MAR

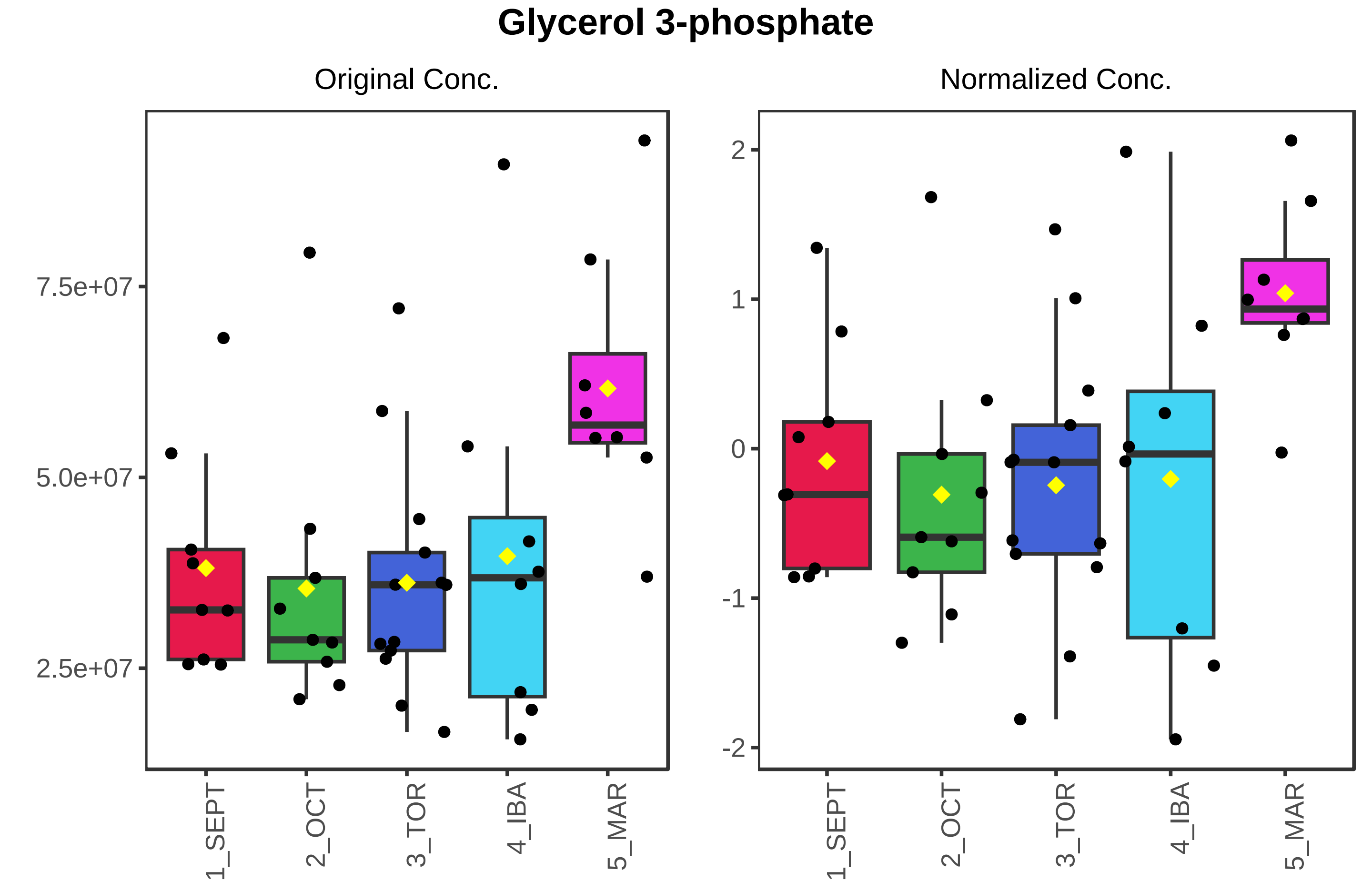

Glycerol 3-phosphate
Original Conc.
Normalized Conc.
7.5e+07
5.0e+07
2.5e+07
2
1
0
-1
-2
1_SEPT
2_OCT
3_TOR
4_IBA
5_MAR

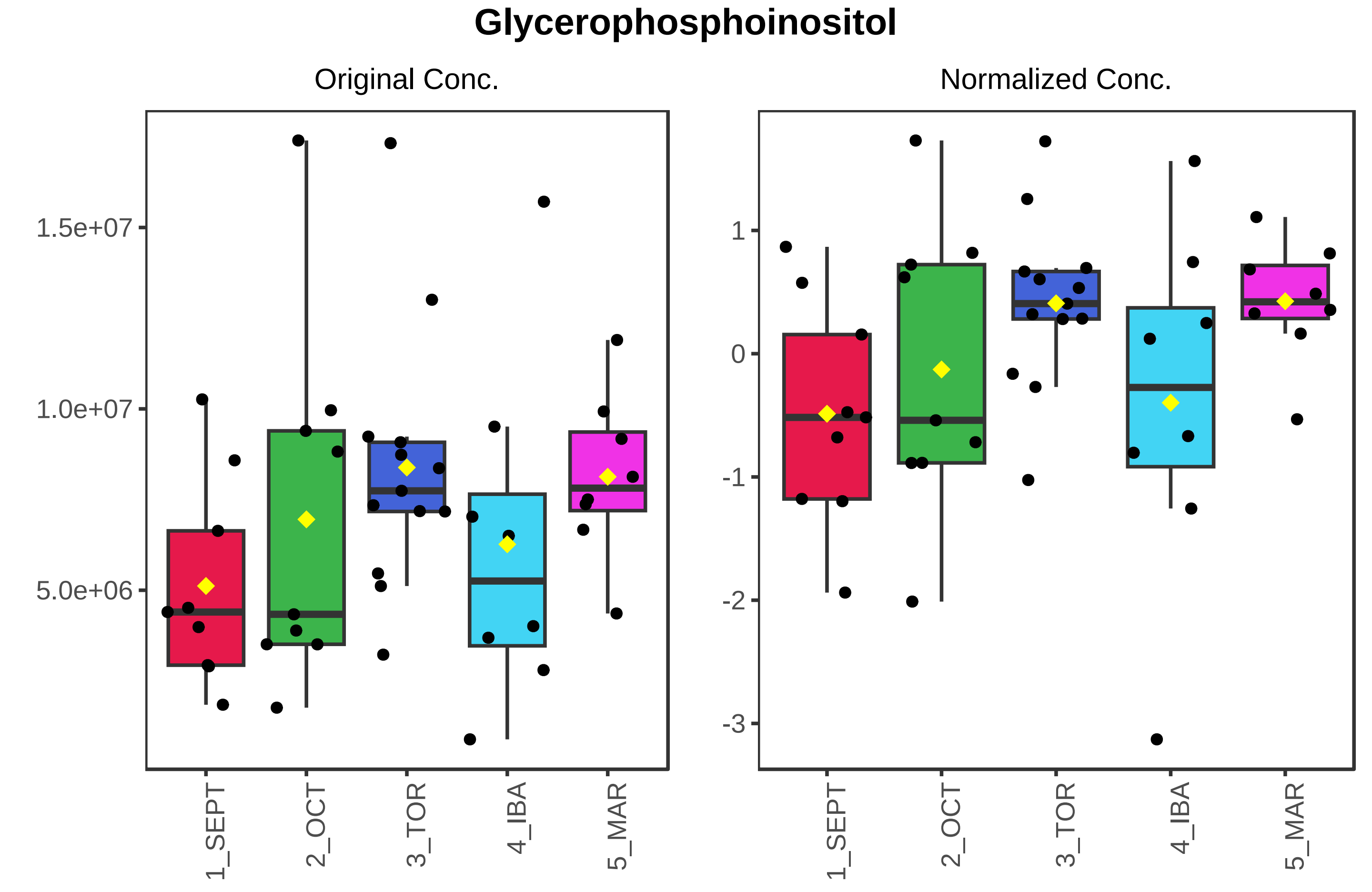

Glycerophosphoinositol
Original Conc.
Normalized Conc.
1.5e+07
1.0e+07
5.0e+06
1
0
-1
-2
-3
1_SEPT
2_OCT
3_TOR
4_IBA
5_MAR

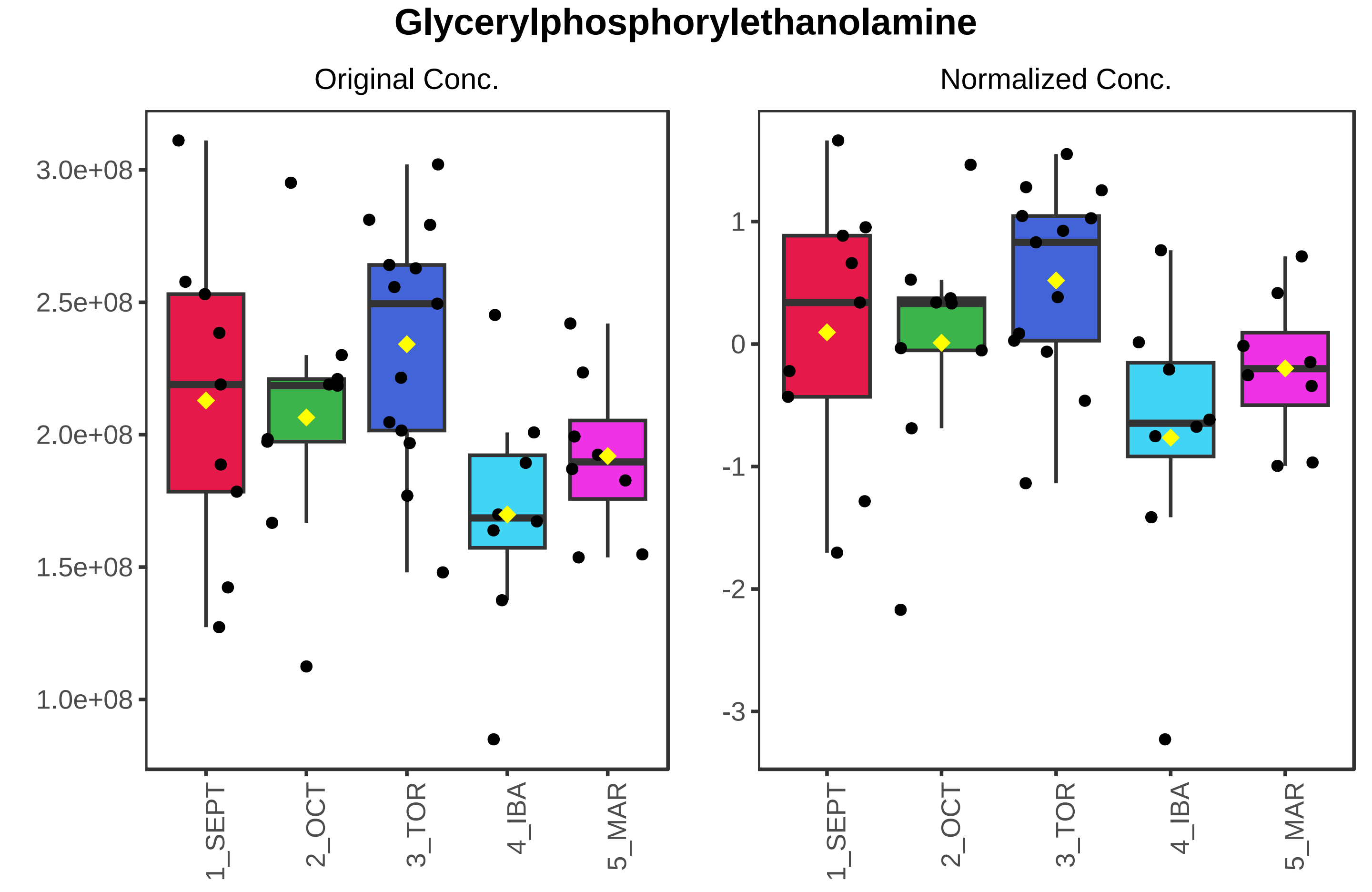
Glycerylphosphorylethanolamine
Original Conc.
Normalized Conc.
3.0e+08
2.5e+08
2.0e+08
1.5e+08
1.0e+08
1
0
-1
-2
-3
1_SEPT
2_OCT
3_TOR
4_IBA
5_MAR
1_SEPT
2_OCT
3_TOR
4_IBA
5_MAR

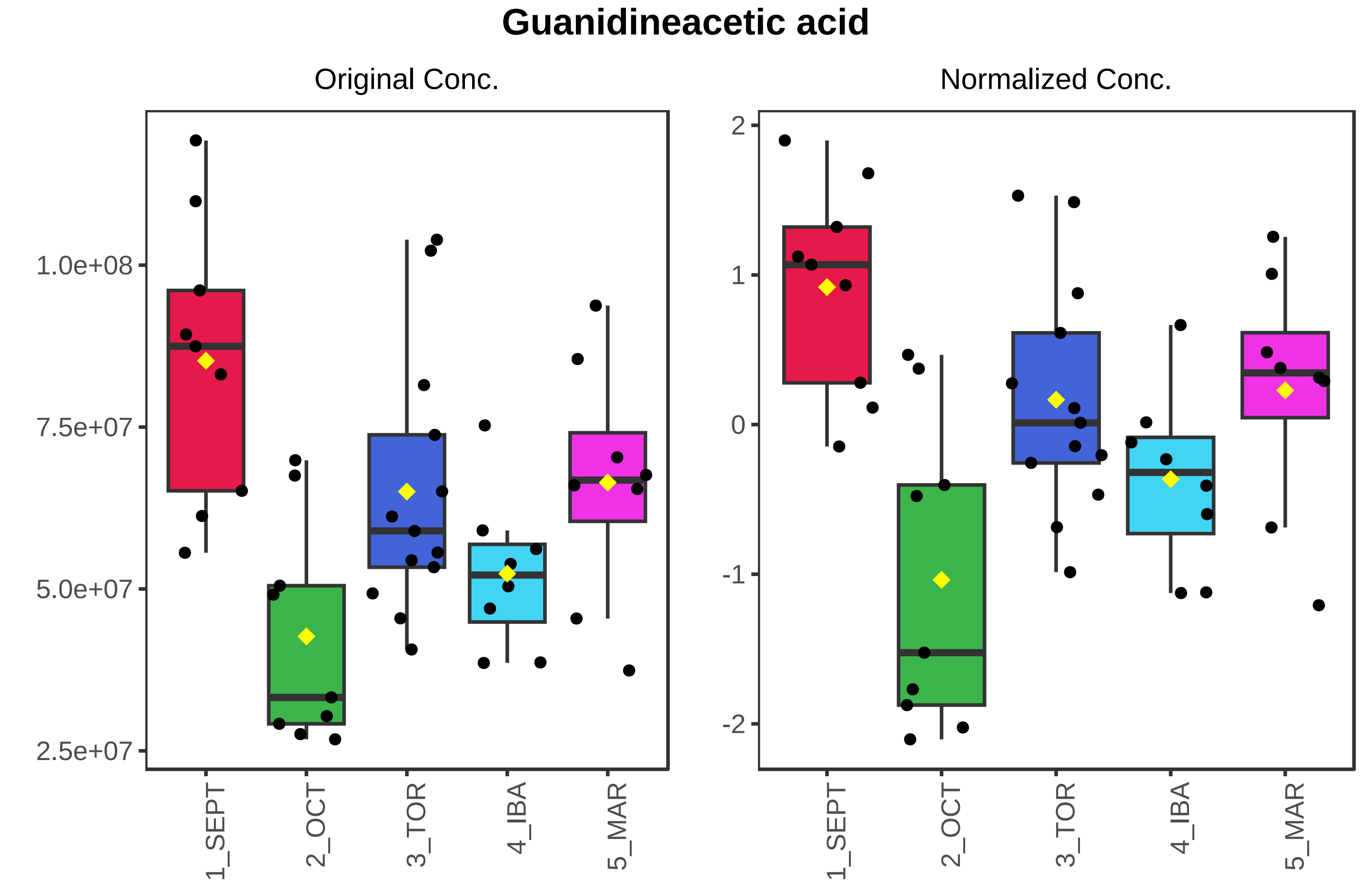

Guanidineacetic acid
Original Conc.
Normalized Conc.
1.0e+08
7.5e+07
5.0e+07
2.5e+07
2
1
0
-1
-2
1_SEPT
2_OCT
3_TOR
4_IBA
5_MAR

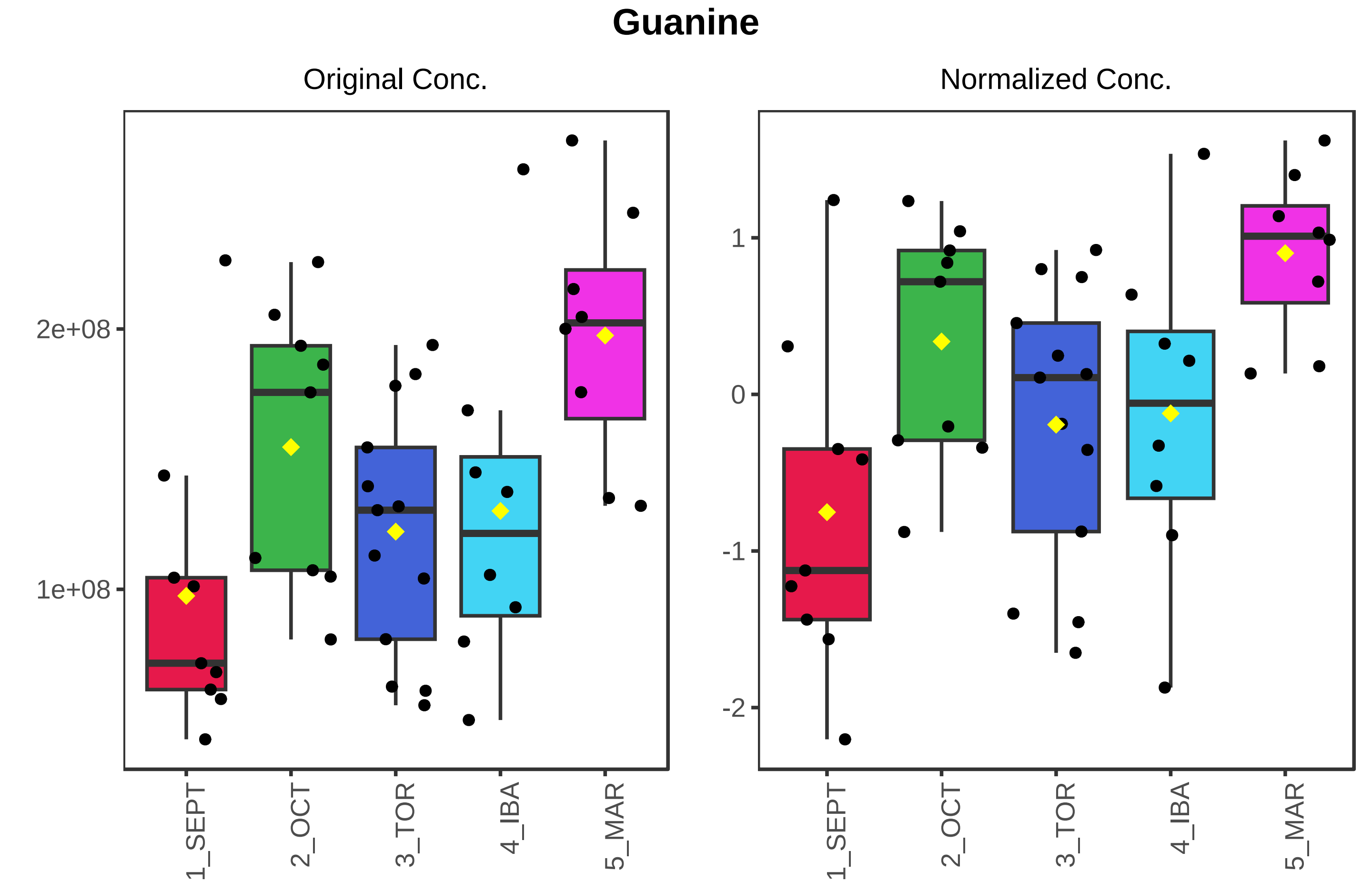

Guanine
Original Conc.
Normalized Conc.
2e+08
1e+08
1
0
-1
-2
1_SEPT
2_OCT
3_TOR
4_IBA
5_MAR
1_SEPT
2_OCT
3_TOR
4_IBA
5_MAR

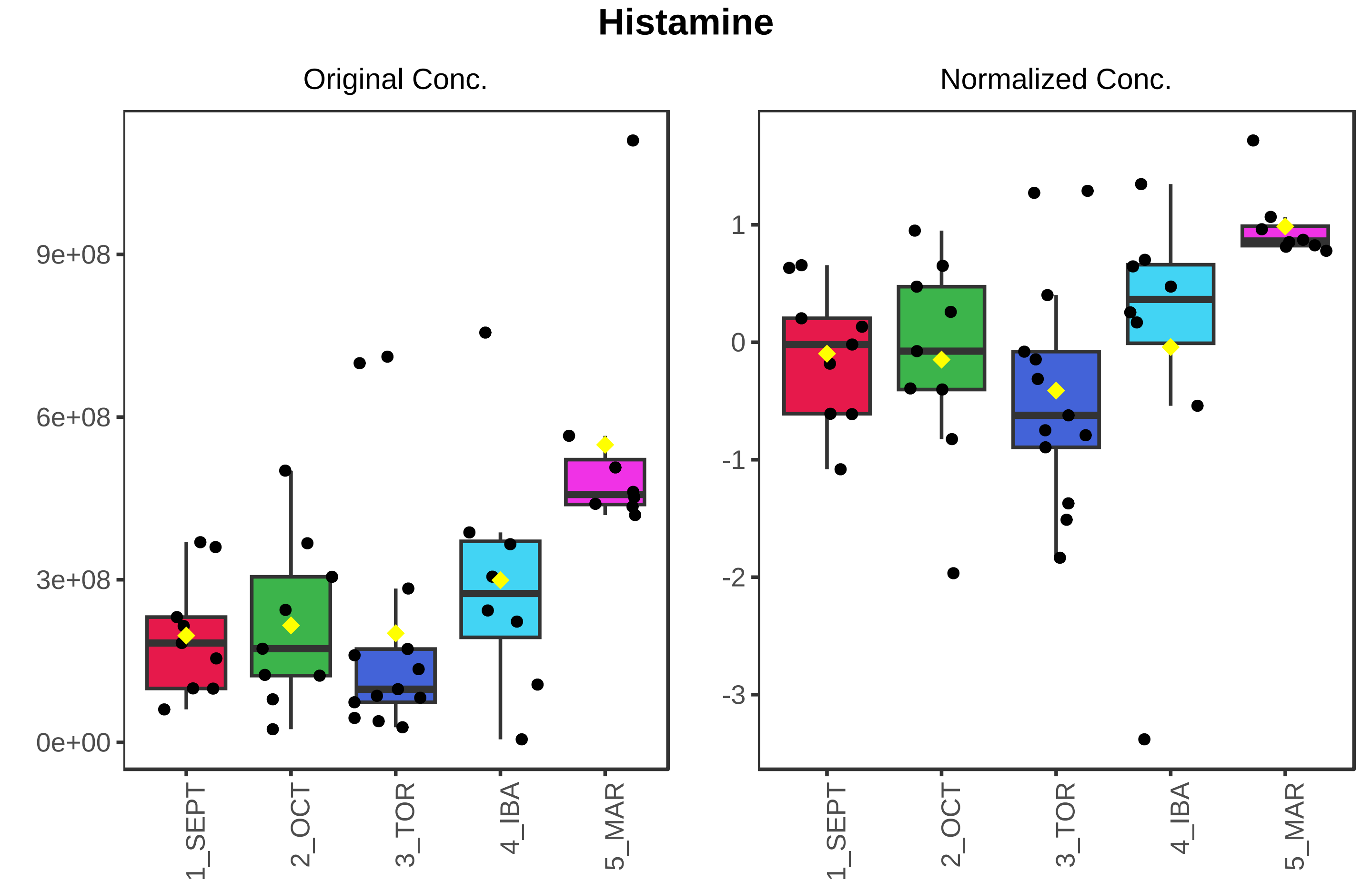
Histamine
Original Conc.
Normalized Conc.
9e+08
6e+08
3e+08
0e+00
1
0
-1
-2
-3
1_SEPT
2_OCT
3_TOR
4_IBA
5_MAR

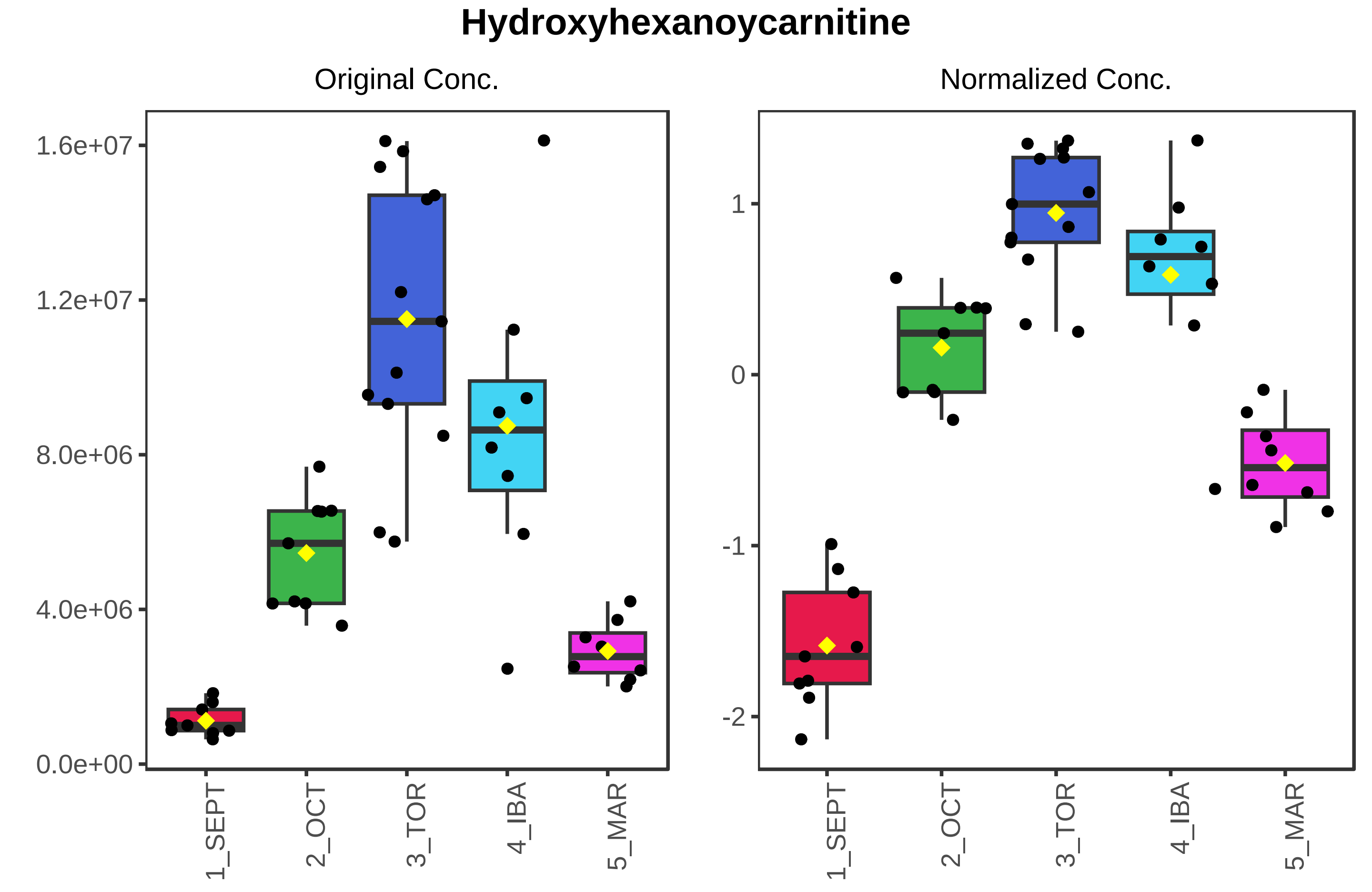

Hydroxyhexanoycarnitine
Original Conc.
Normalized Conc.
1.6e+07
1.2e+07
8.0e+06
4.0e+06
0.0e+00
1
0
-1
-2
1_SEPT
2_OCT
3_TOR
4_IBA
5_MAR

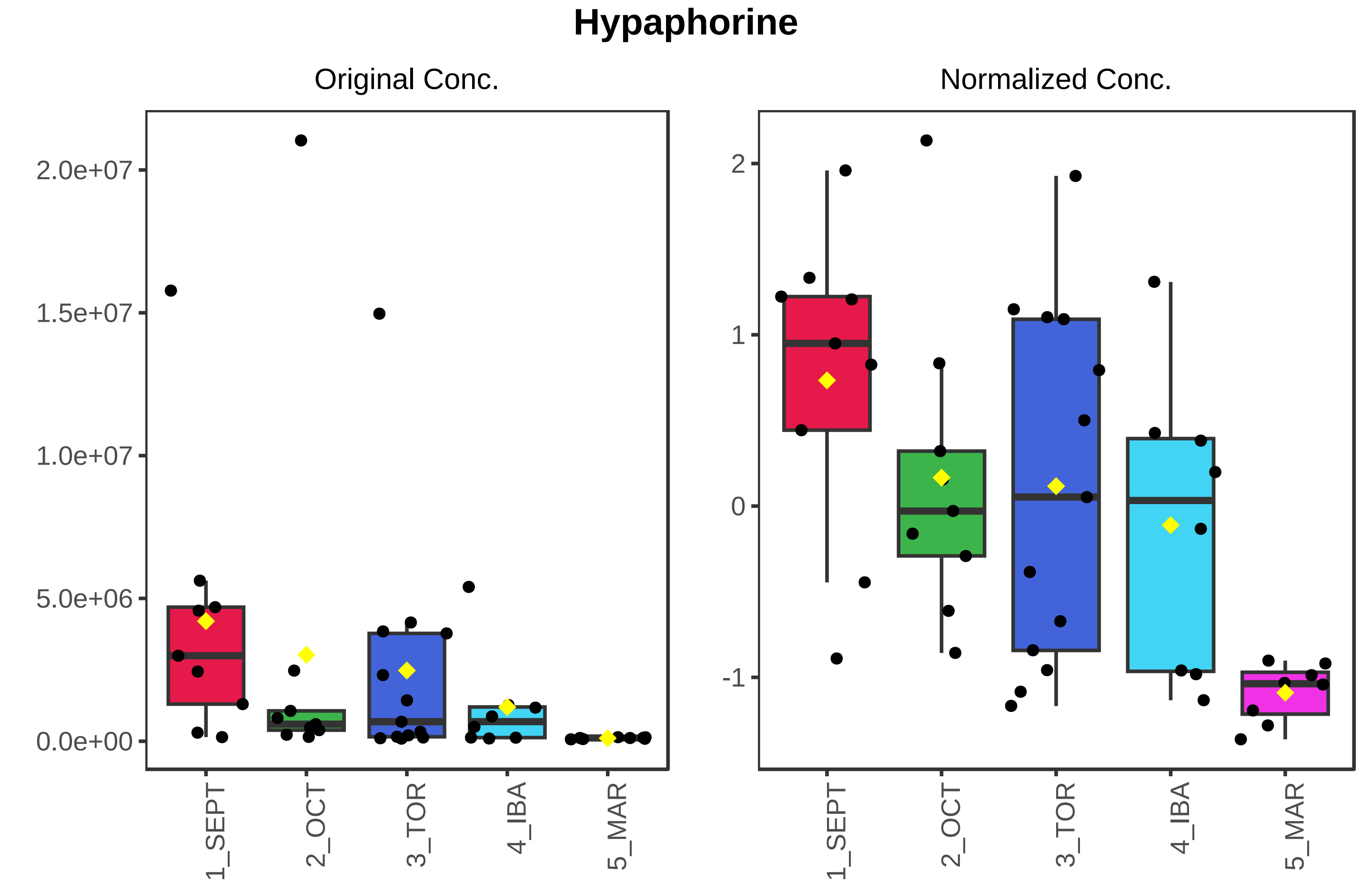

Hypaphorine
Original Conc.
Normalized Conc.
2.0e+07
1.5e+07
1.0e+07
5.0e+06
0.0e+00
2
1
0
-1
1_SEPT
2_OCT
3_TOR
4_IBA
5_MAR

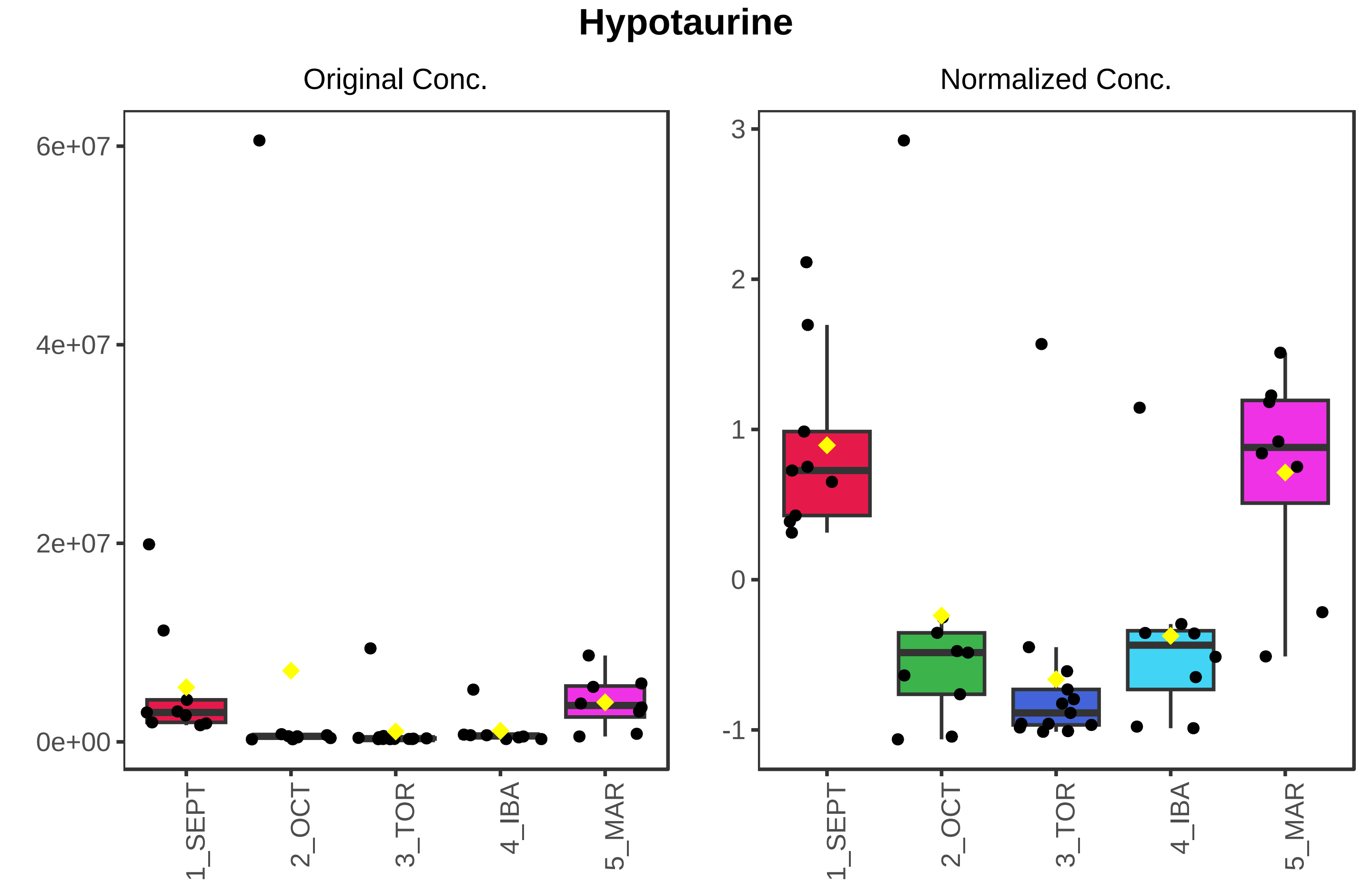

Hypotaurine
Original Conc.
Normalized Conc.
6e+07
4e+07
2e+07
0e+00
3
2
1
0
-1
1_SEPT
2_OCT
3_TOR
4_IBA
5_MAR

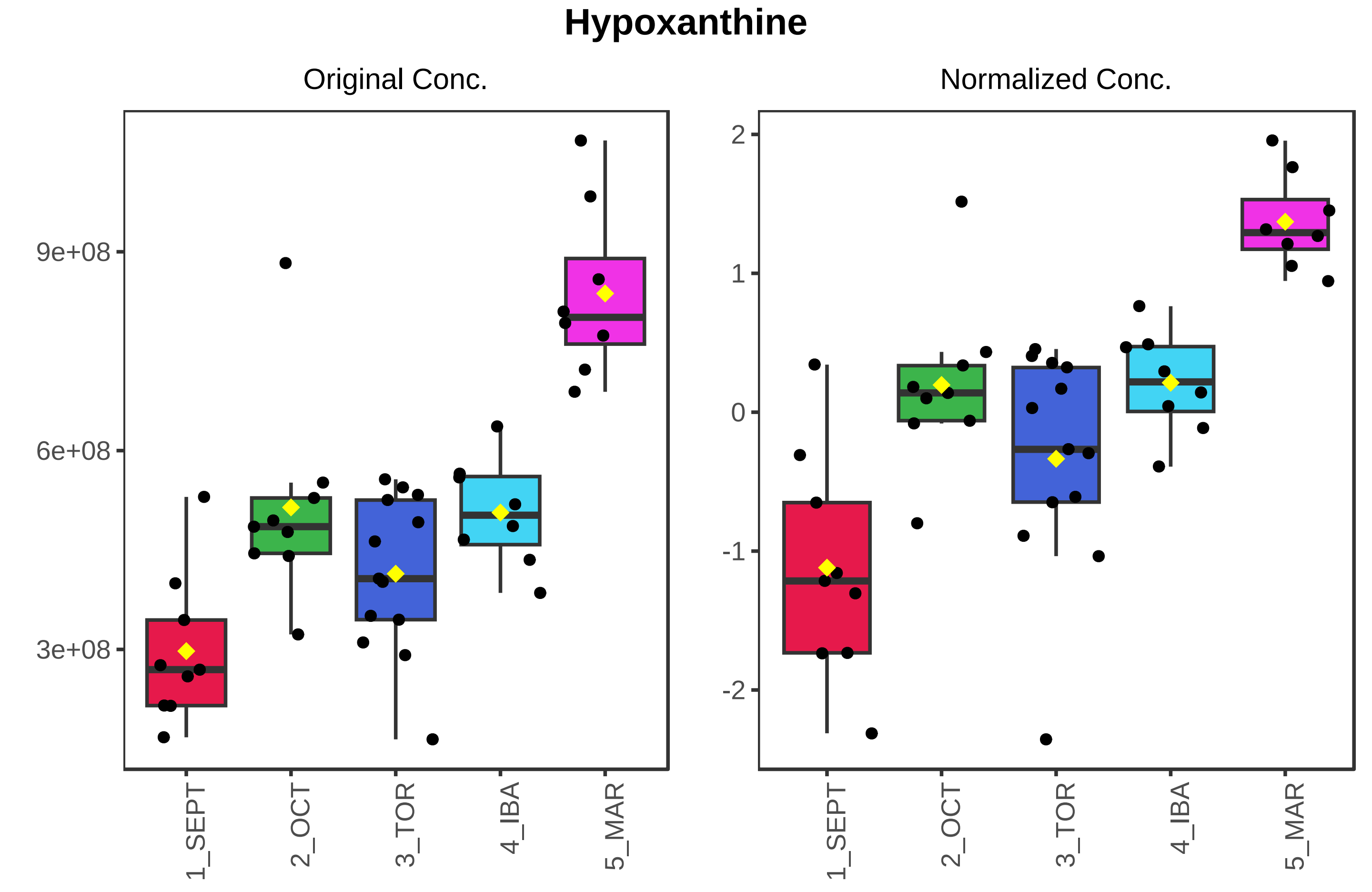

Hypoxanthine
Original Conc.
Normalized Conc.
9e+08
6e+08
3e+08
2
1
0
-1
-2
1_SEPT
2_OCT
3_TOR
4_IBA
5_MAR
1_SEPT
2_OCT
3_TOR
4_IBA
5_MAR

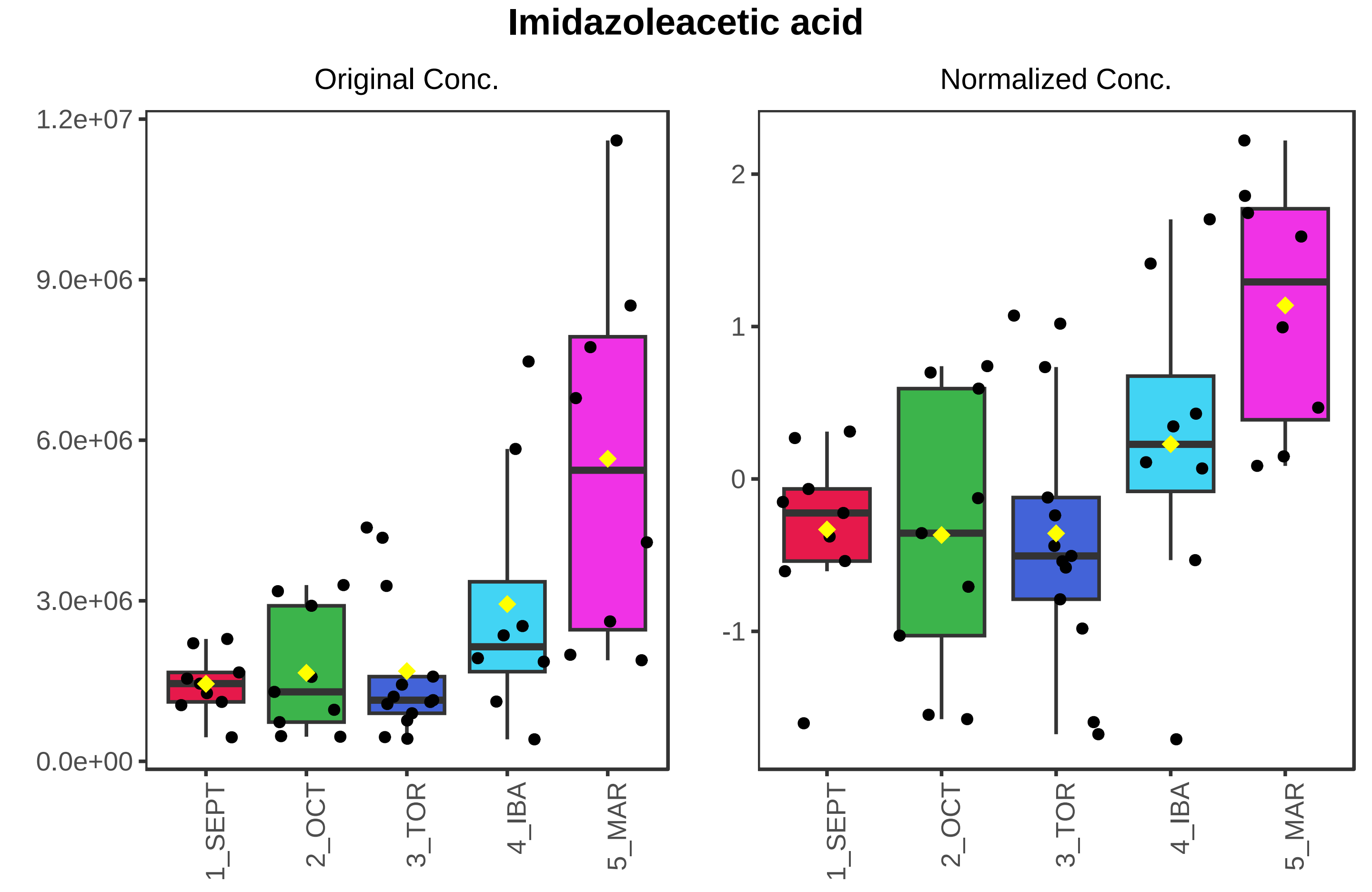

Imidazoleacetic acid
Original Conc.
Normalized Conc.
1.2e+07
9.0e+06
6.0e+06
3.0e+06
0.0e+00
2
1
0
-1
1_SEPT
2_OCT
3_TOR
4_IBA
5_MAR

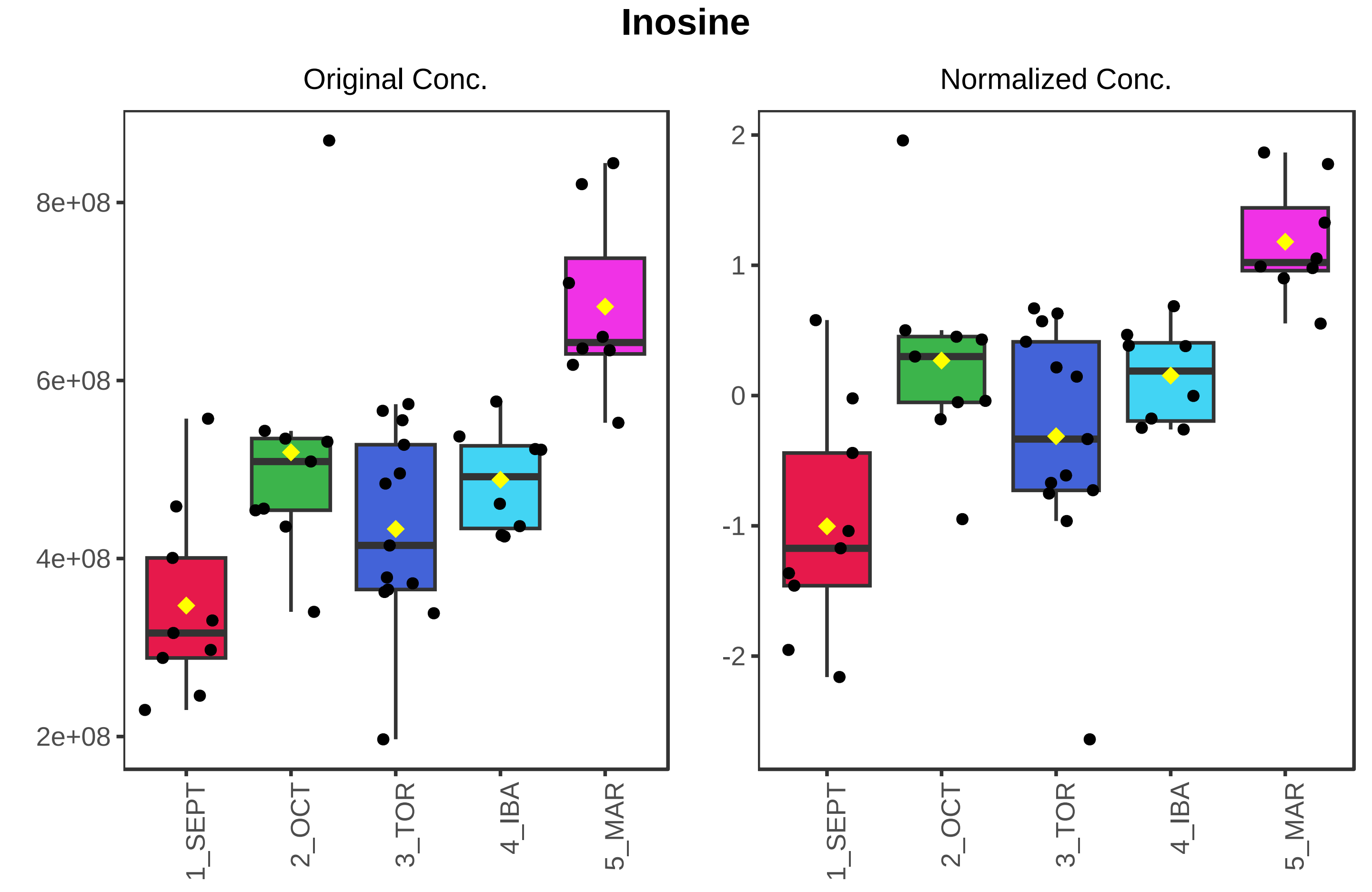

Inosine
Original Conc.
Normalized Conc.
8e+08
6e+08
4e+08
2e+08
2
1
0
-1
-2
1_SEPT
2_OCT
3_TOR
4_IBA
5_MAR

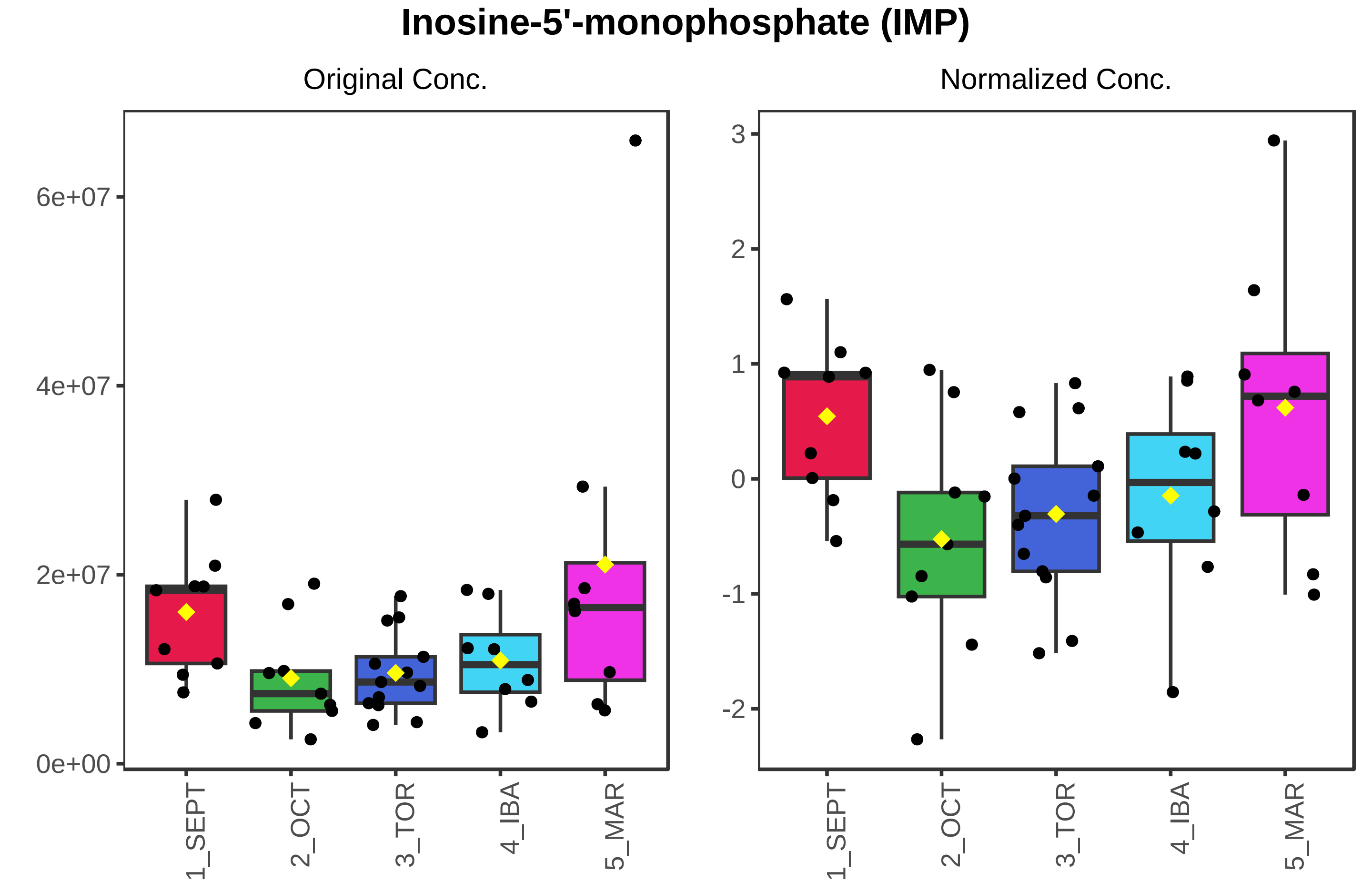

Inosine-5'-monophosphate (IMP)
Original Conc.
Normalized Conc.
6e+07
4e+07
2e+07
0e+00
3
2
1
0
-1
-2
1_SEPT
2_OCT
3_TOR
4_IBA
5_MAR

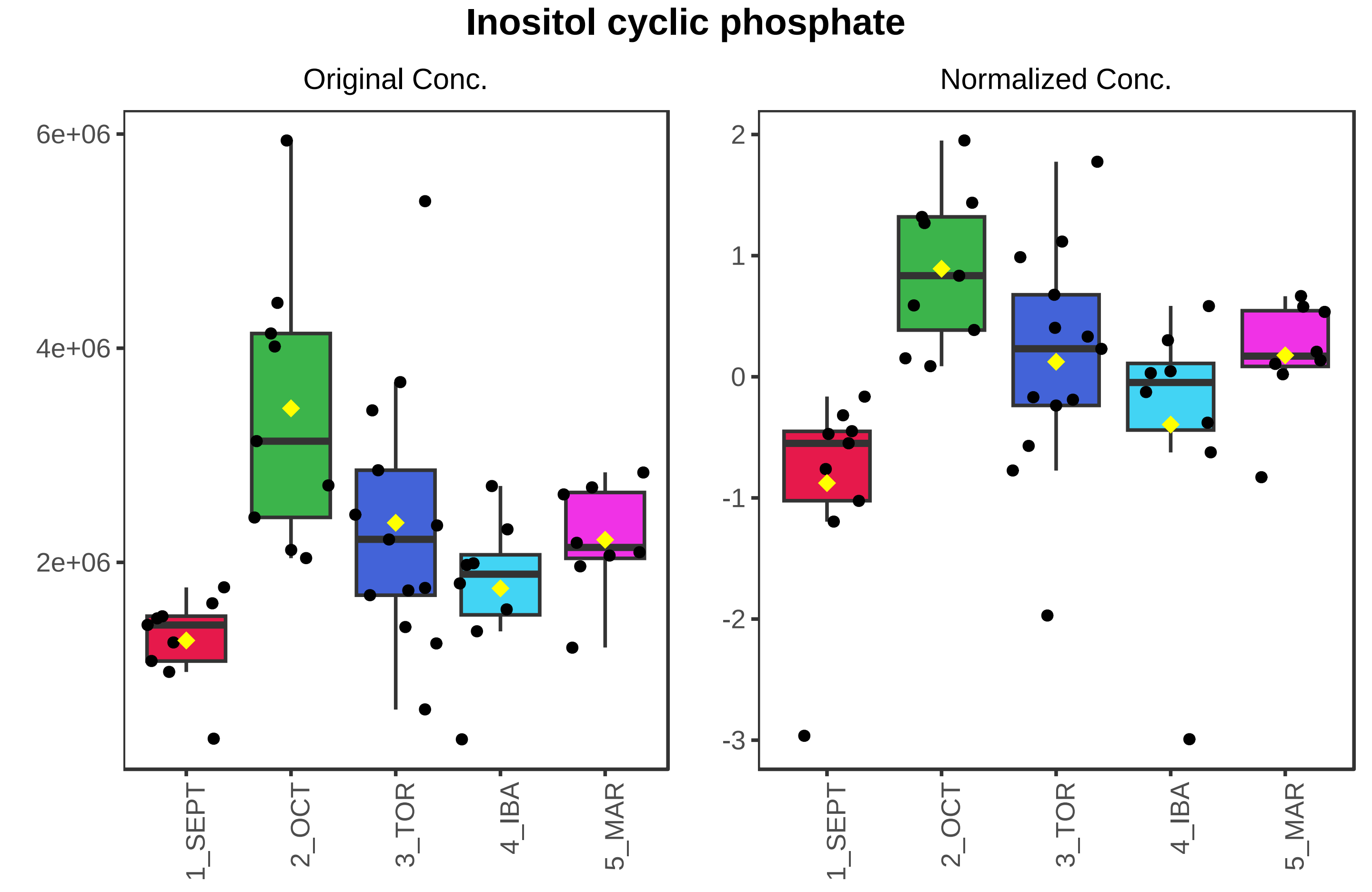

Inositol cyclic phosphate
Original Conc.
Normalized Conc.
6e+06
4e+06
2e+06
2
1
0
-1
-2
-3
1_SEPT
2_OCT
3_TOR
4_IBA
5_MAR

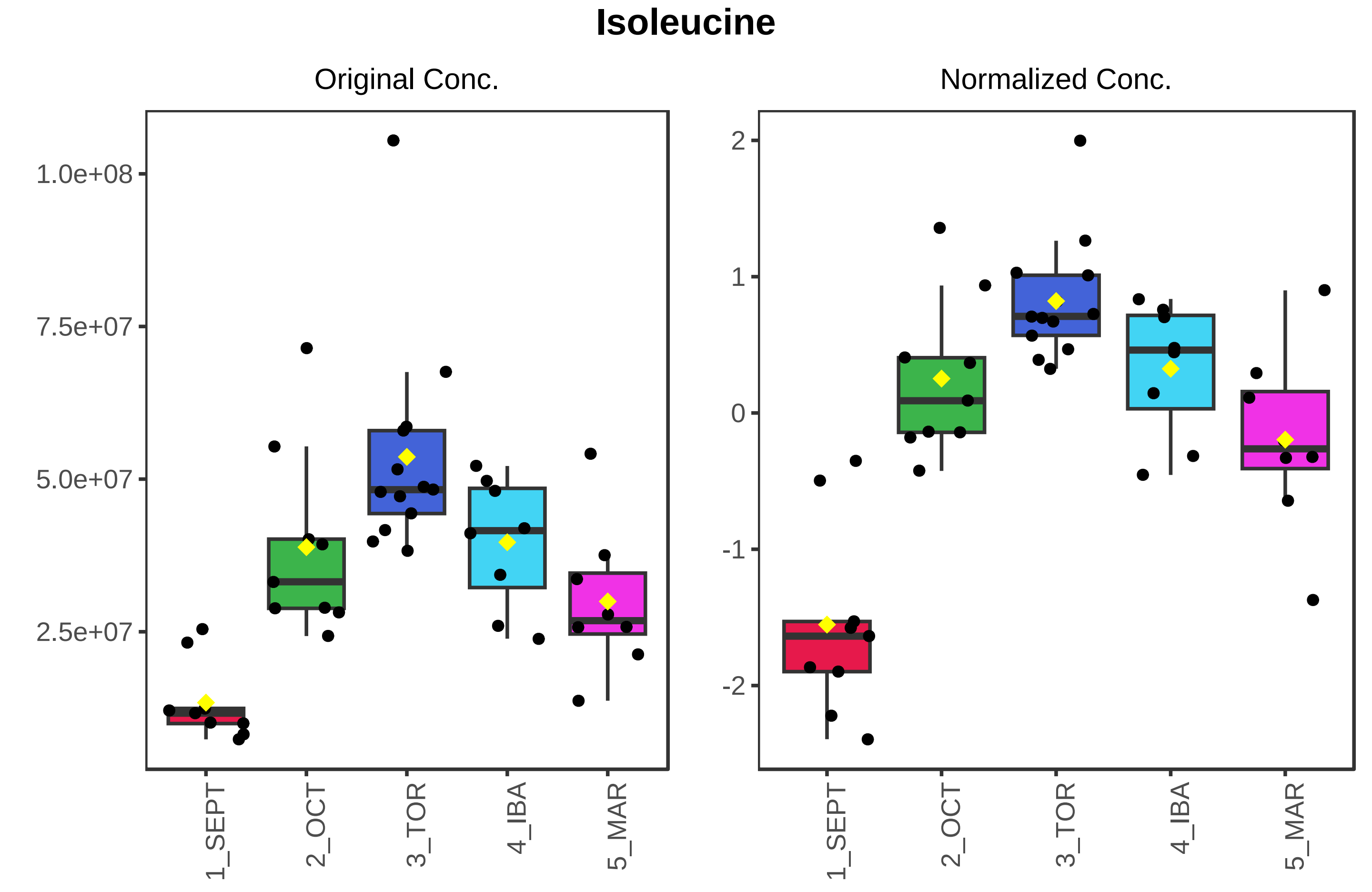

Isoleucine
Original Conc.
Normalized Conc.
1.0e+08
7.5e+07
5.0e+07
2.5e+07
2
1
0
-1
-2
1_SEPT
2_OCT
3_TOR
4_IBA
5_MAR
1_SEPT
2_OCT
3_TOR
4_IBA
5_MAR

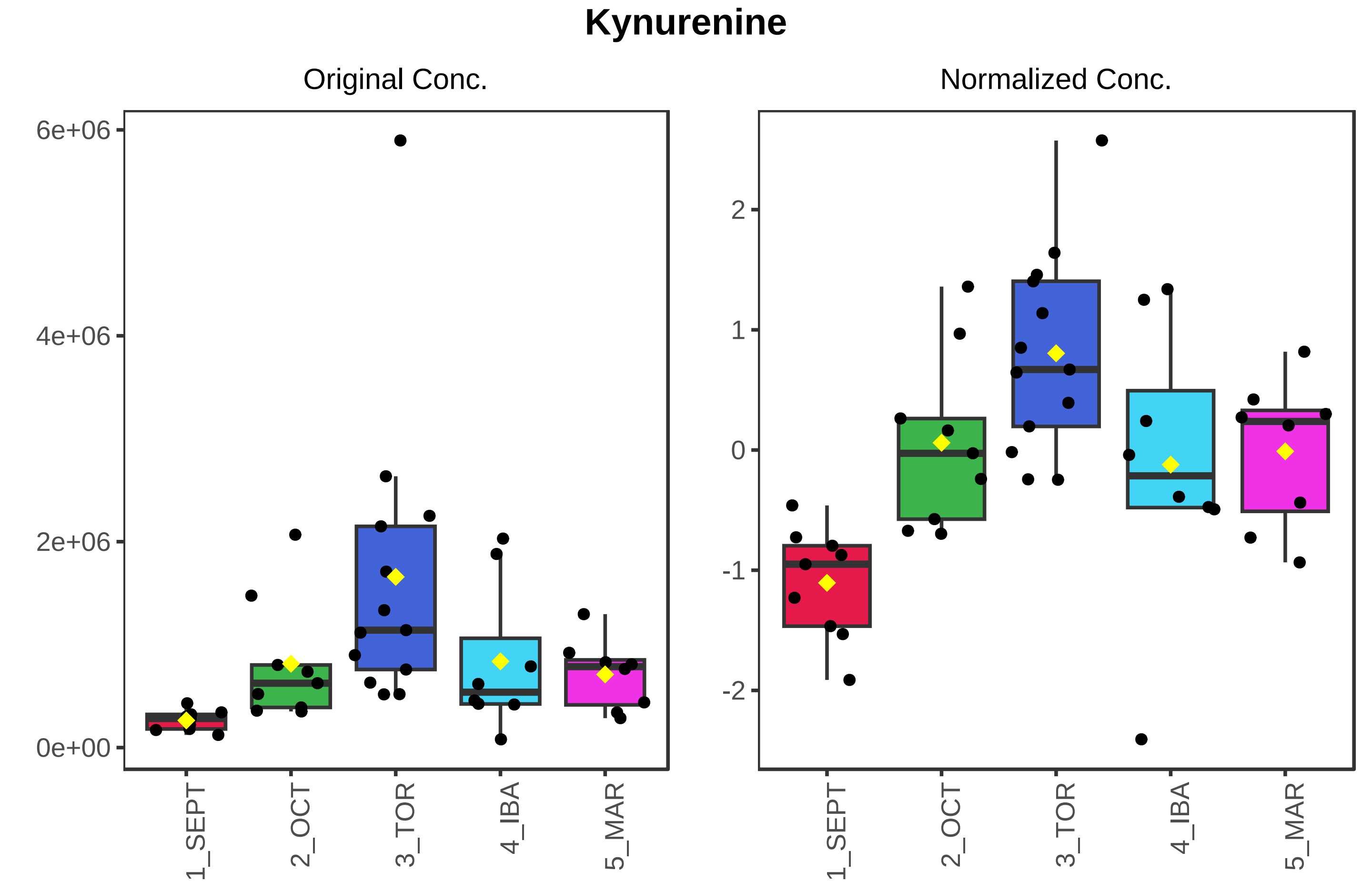
Kynurenine
Original Conc.
Normalized Conc.
6e+06
4e+06
2e+06
0e+00
2
1
0
-1
-2
1_SEPT
2_OCT
3_TOR
4_IBA
5_MAR
1_SEPT
2_OCT
3_TOR
4_IBA
5_MAR

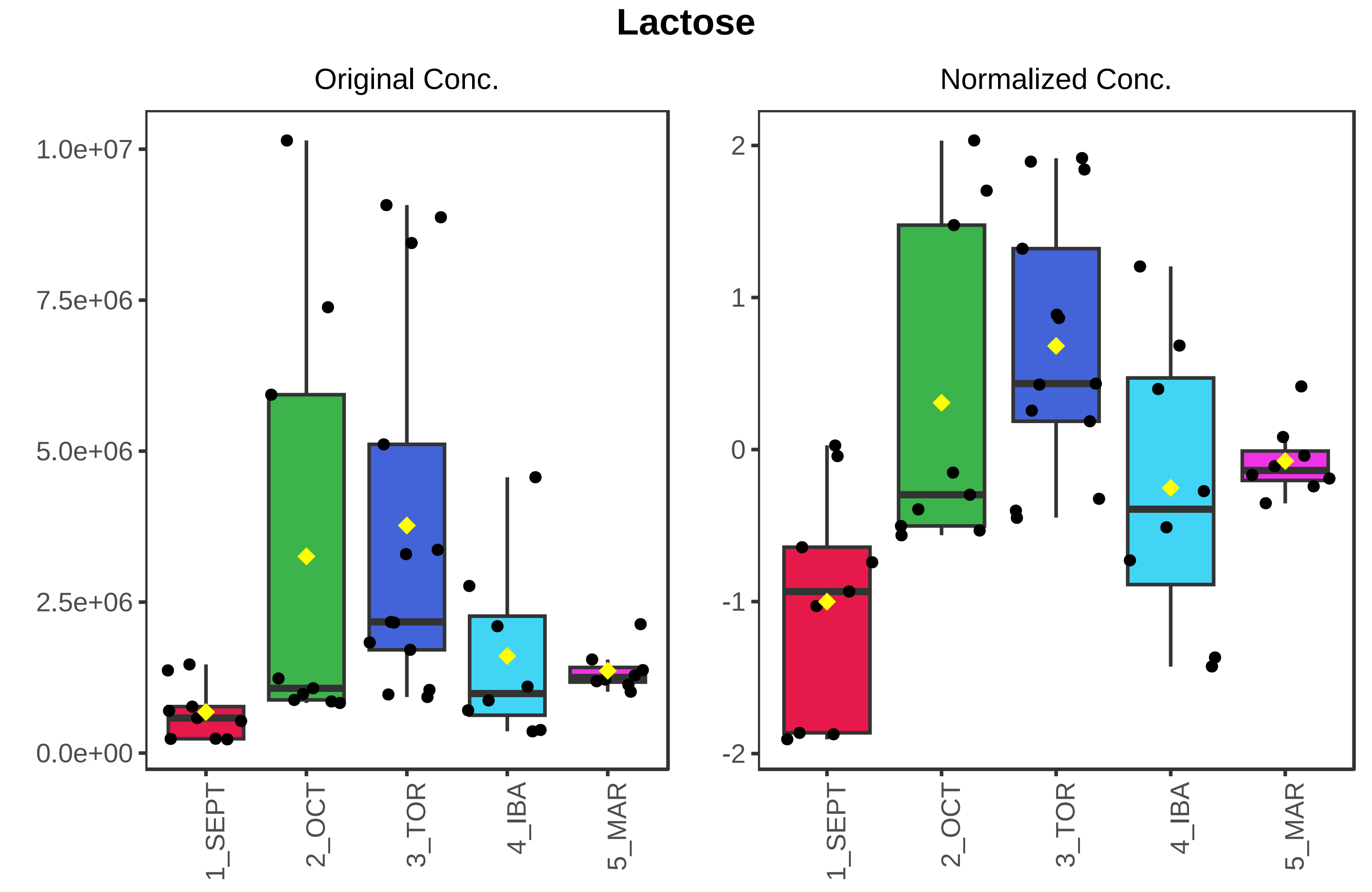

Lactose
Original Conc.
Normalized Conc.
1.0e+07
7.5e+06
5.0e+06
2.5e+06
0.0e+00
2
1
0
-1
-2
1_SEPT
2_OCT
3_TOR
4_IBA
5_MAR

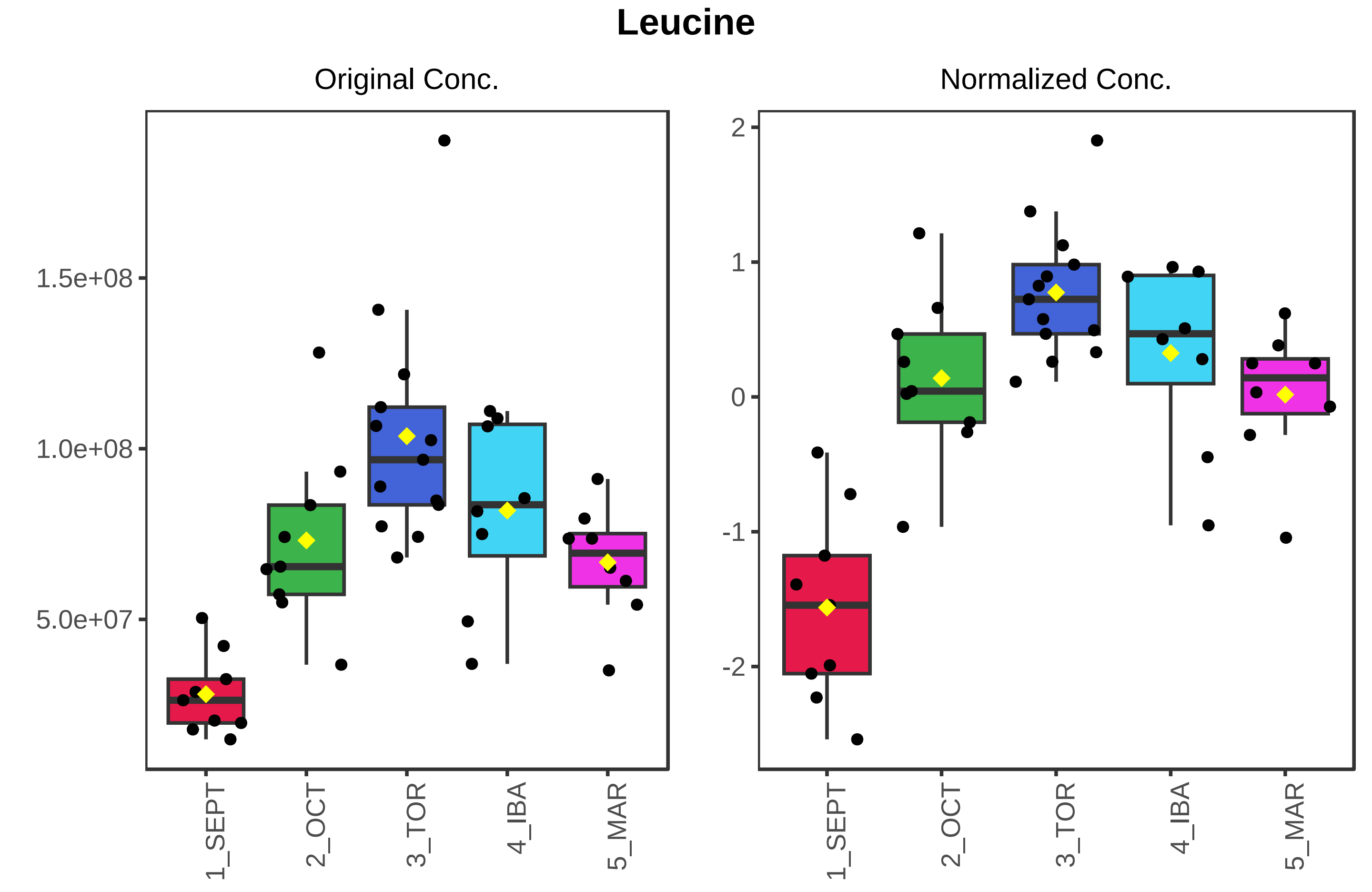
Leucine
Original Conc.
Normalized Conc.
1.5e+08
1.0e+08
5.0e+07
2
1
0
-1
-2
1_SEPT
2_OCT
3_TOR
4_IBA
5_MAR

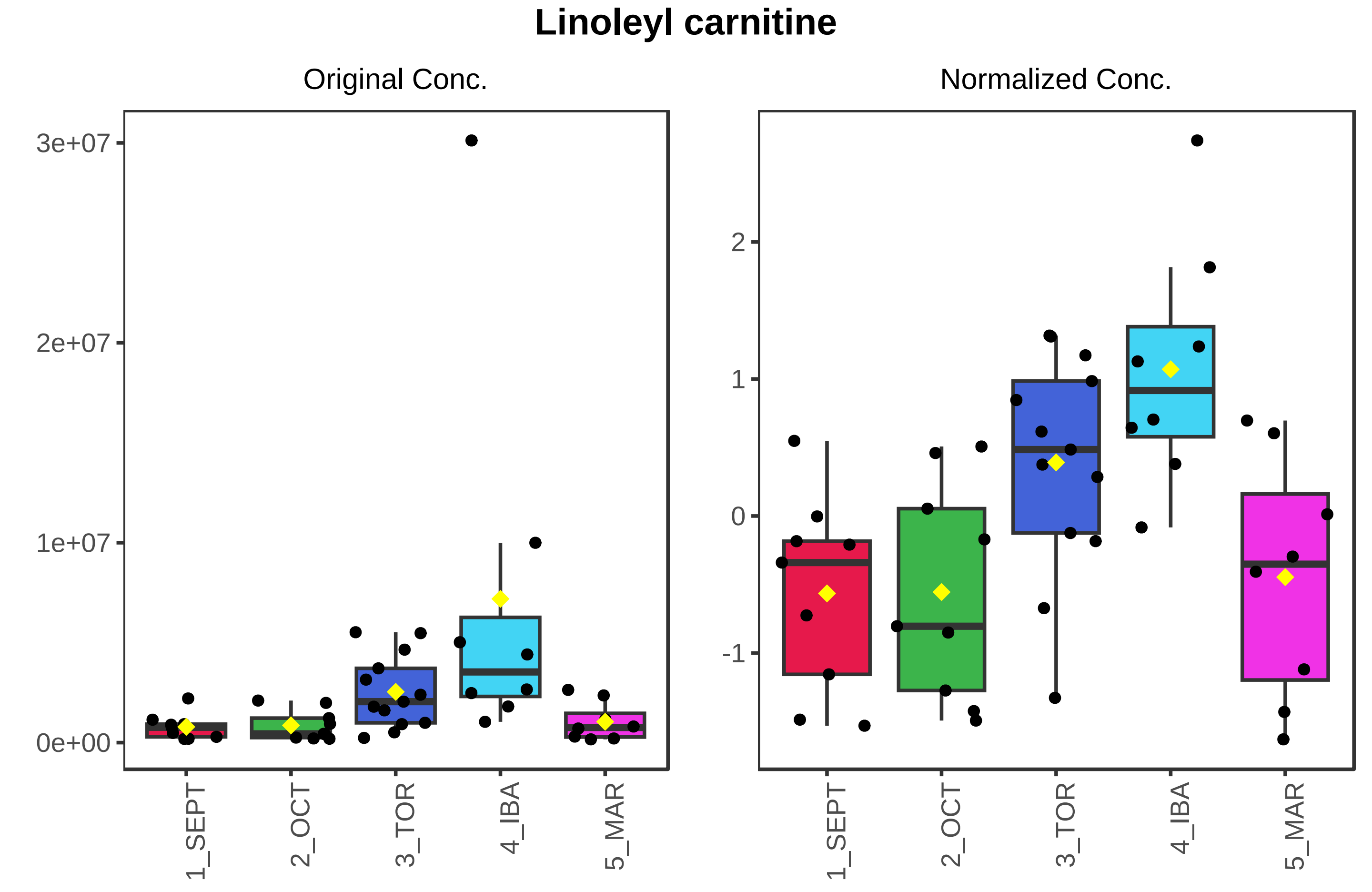

Linoleyl carnitine
Original Conc.
Normalized Conc.
3e+07
2e+07
1e+07
0e+00
2
1
0
-1
1_SEPT
2_OCT
3_TOR
4_IBA
5_MAR

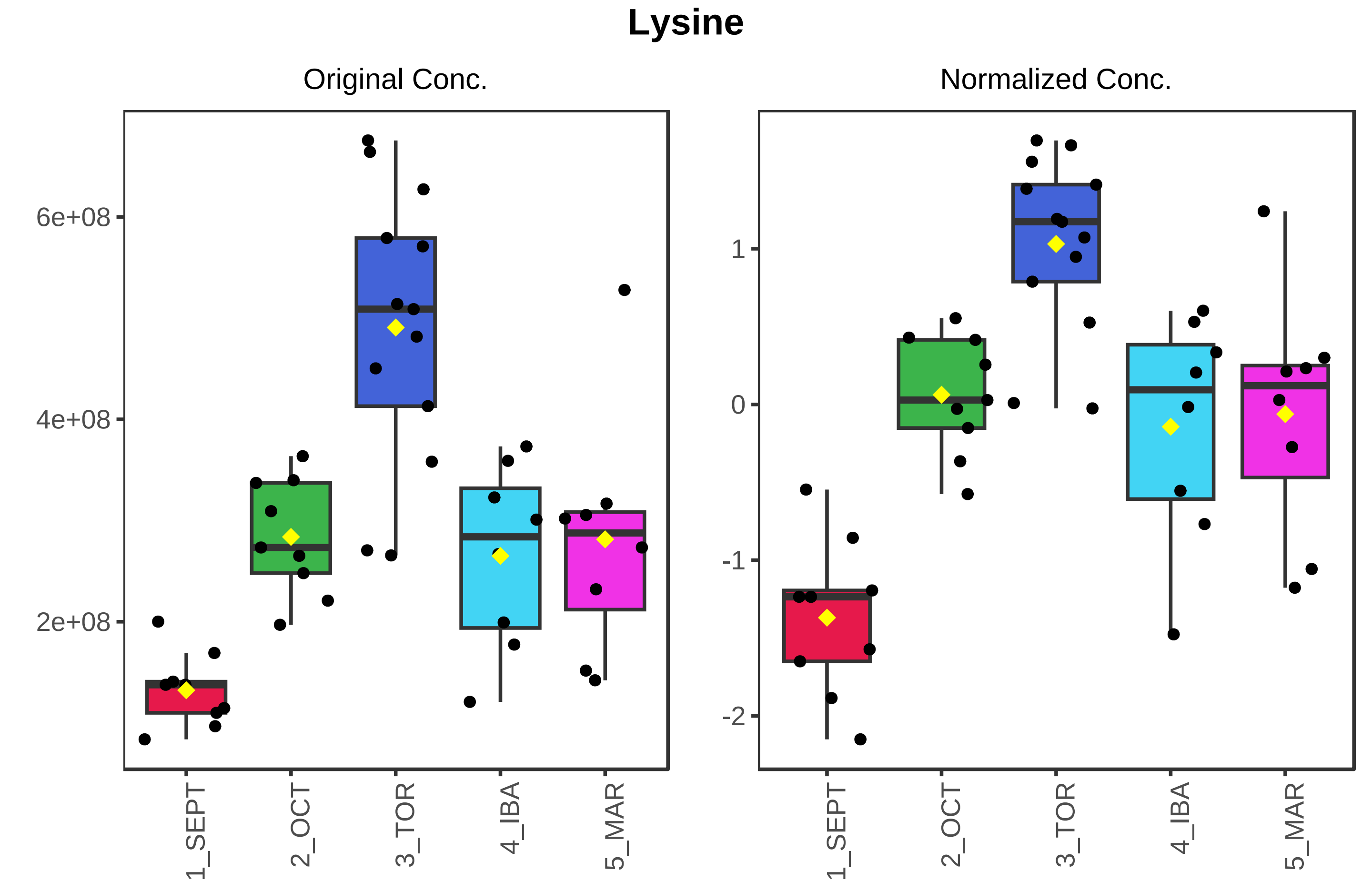

Lysine
Original Conc.
Normalized Conc.
6e+08
4e+08
2e+08
1
0
-1
-2
1_SEPT
2_OCT
3_TOR
4_IBA
5_MAR
1_SEPT
2_OCT
3_TOR
4_IBA
5_MAR

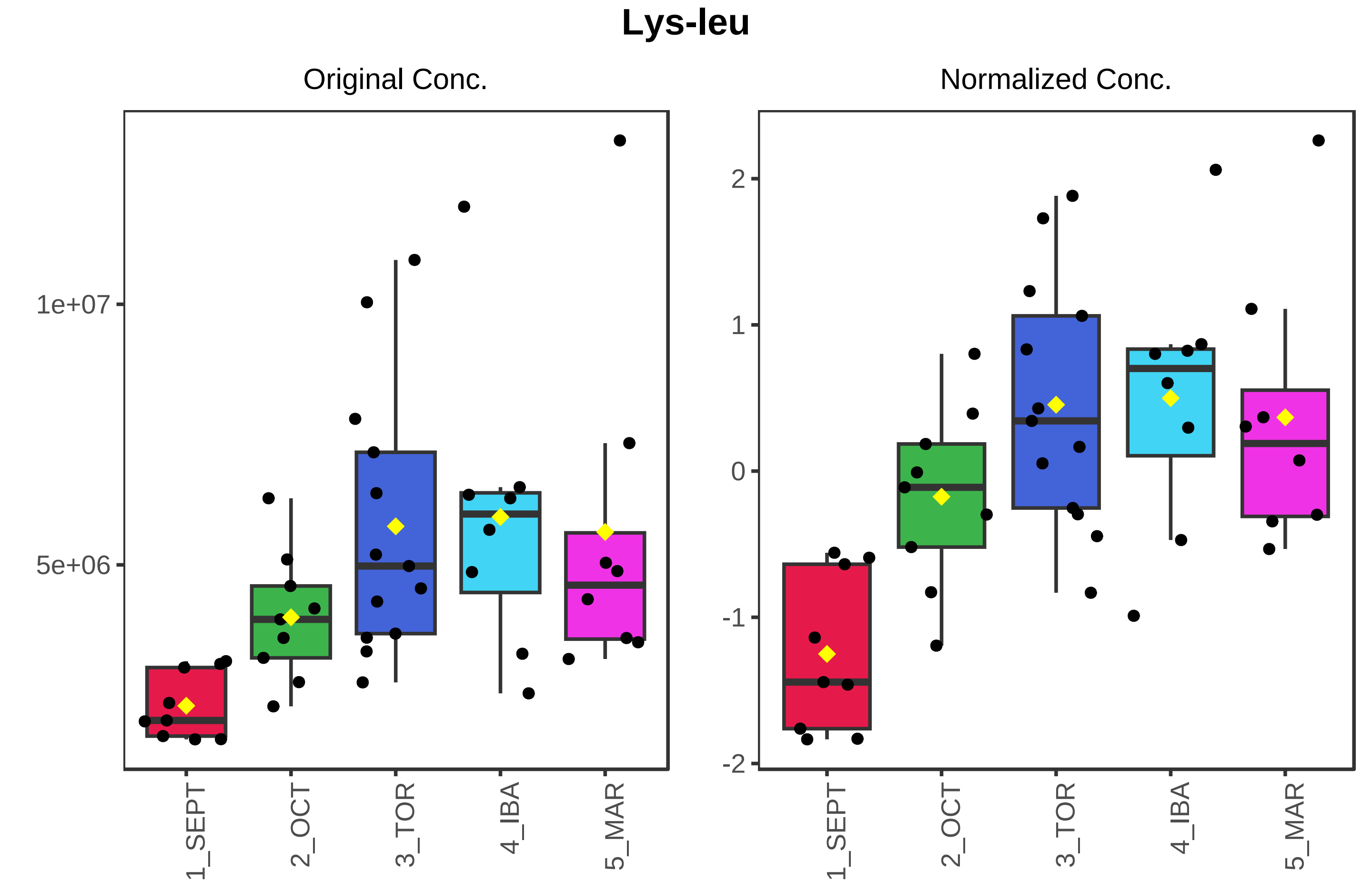

Lys-leu
Original Conc.
Normalized Conc.
1e+07
5e+06
2
1
0
-1
-2
1_SEPT
2_OCT
3_TOR
4_IBA
5_MAR

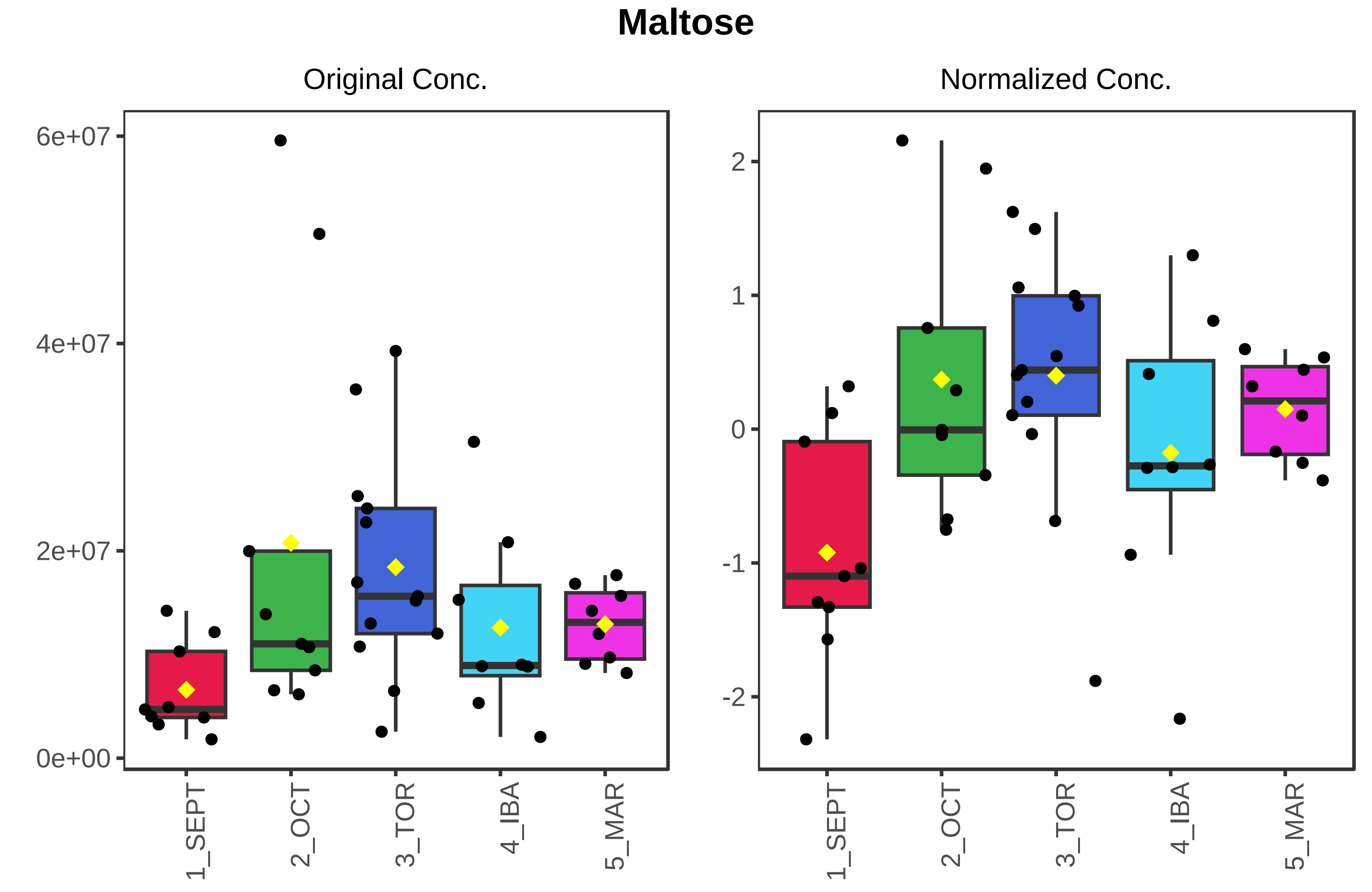

Maltose
Original Conc.
Normalized Conc.
6e+07
4e+07
2e+07
0e+00
2
1
0
-1
-2
1_SEPT
2_OCT
3_TOR
4_IBA
5_MAR

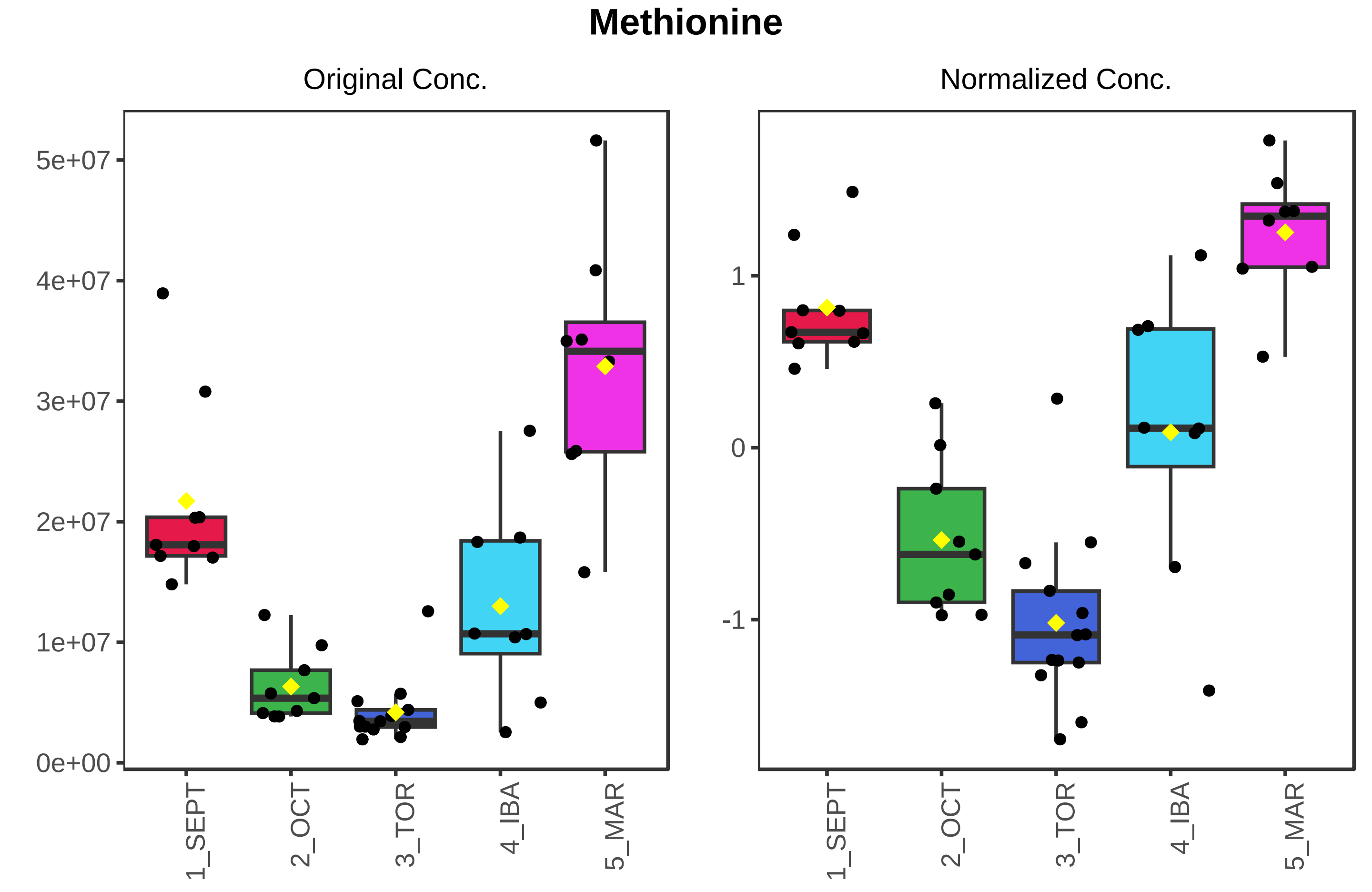

Methionine
Original Conc.
Normalized Conc.
5e+07
4e+07
3e+07
2e+07
1e+07
0e+00
1
0
-1
1_SEPT
2_OCT
3_TOR
4_IBA
5_MAR

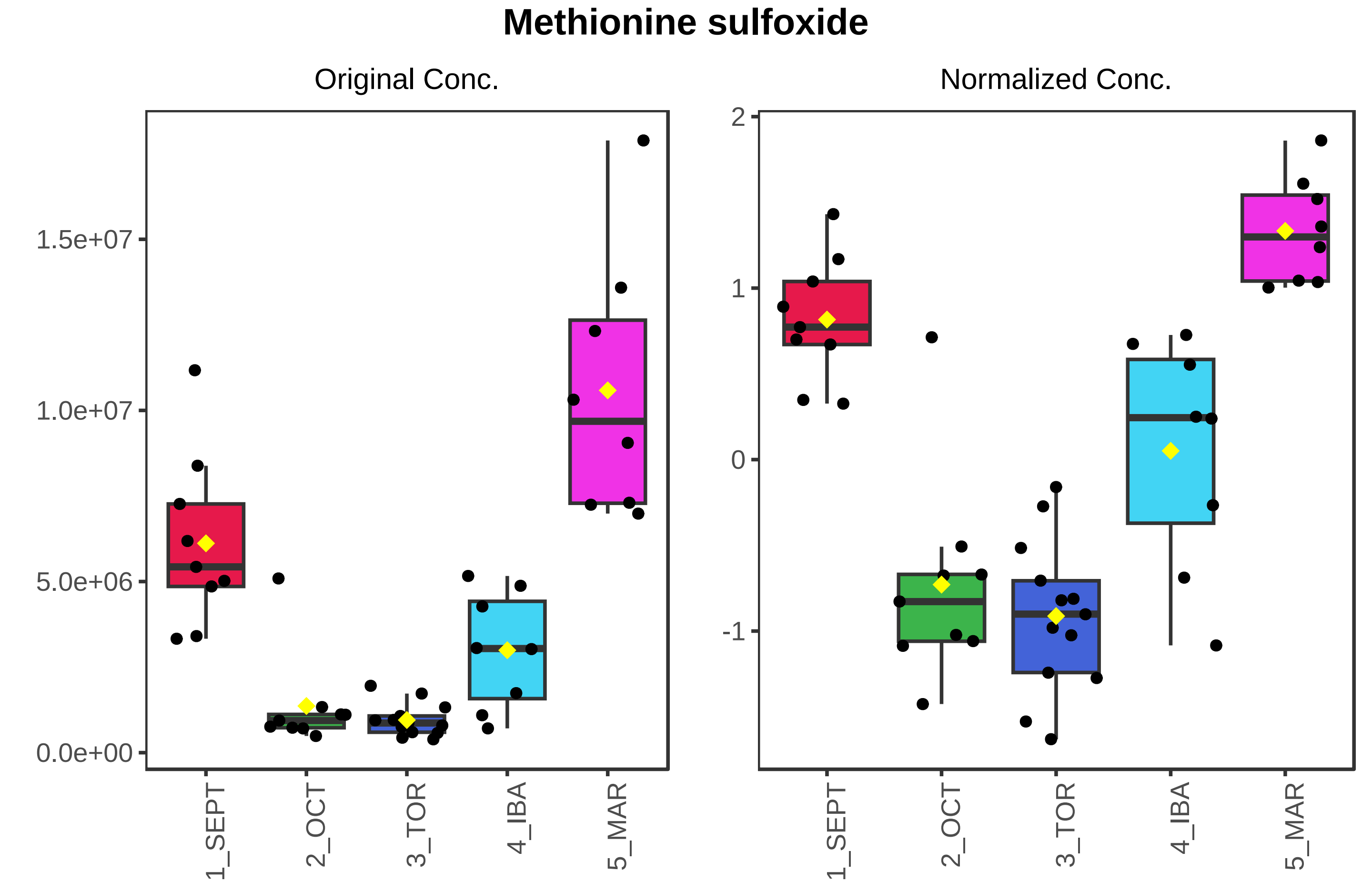
Methionine sulfoxide
Original Conc.
Normalized Conc.
1.5e+07
1.0e+07
5.0e+06
0.0e+00
2
1
0
-1
1_SEPT
2_OCT
3_TOR
4_IBA
5_MAR

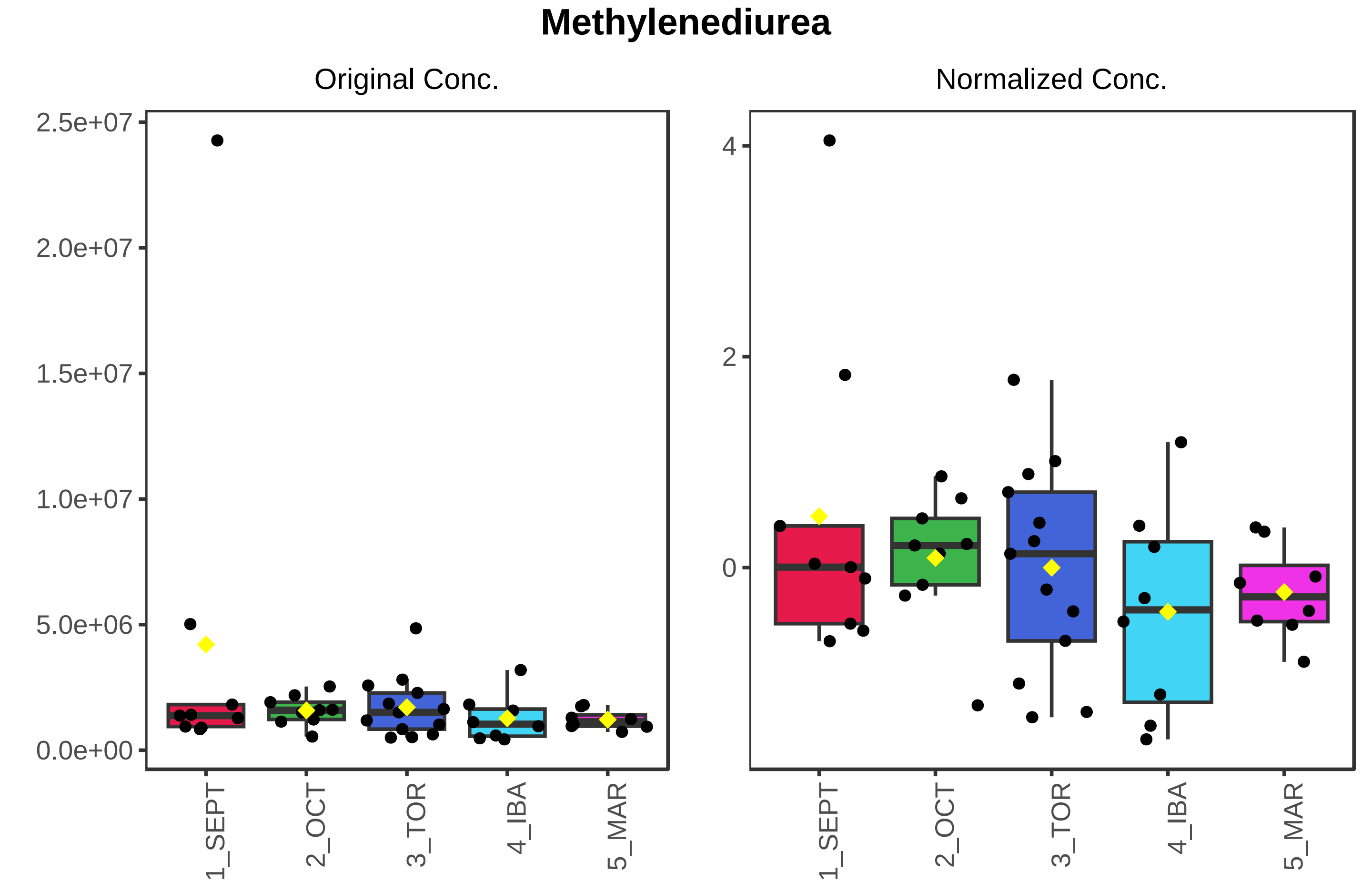

Methylenediurea
Original Conc.
Normalized Conc.
2.5e+07
2.0e+07
1.5e+07
1.0e+07
5.0e+06
0.0e+00
4
2
0
1_SEPT
2_OCT
3_TOR
4_IBA
5_MAR

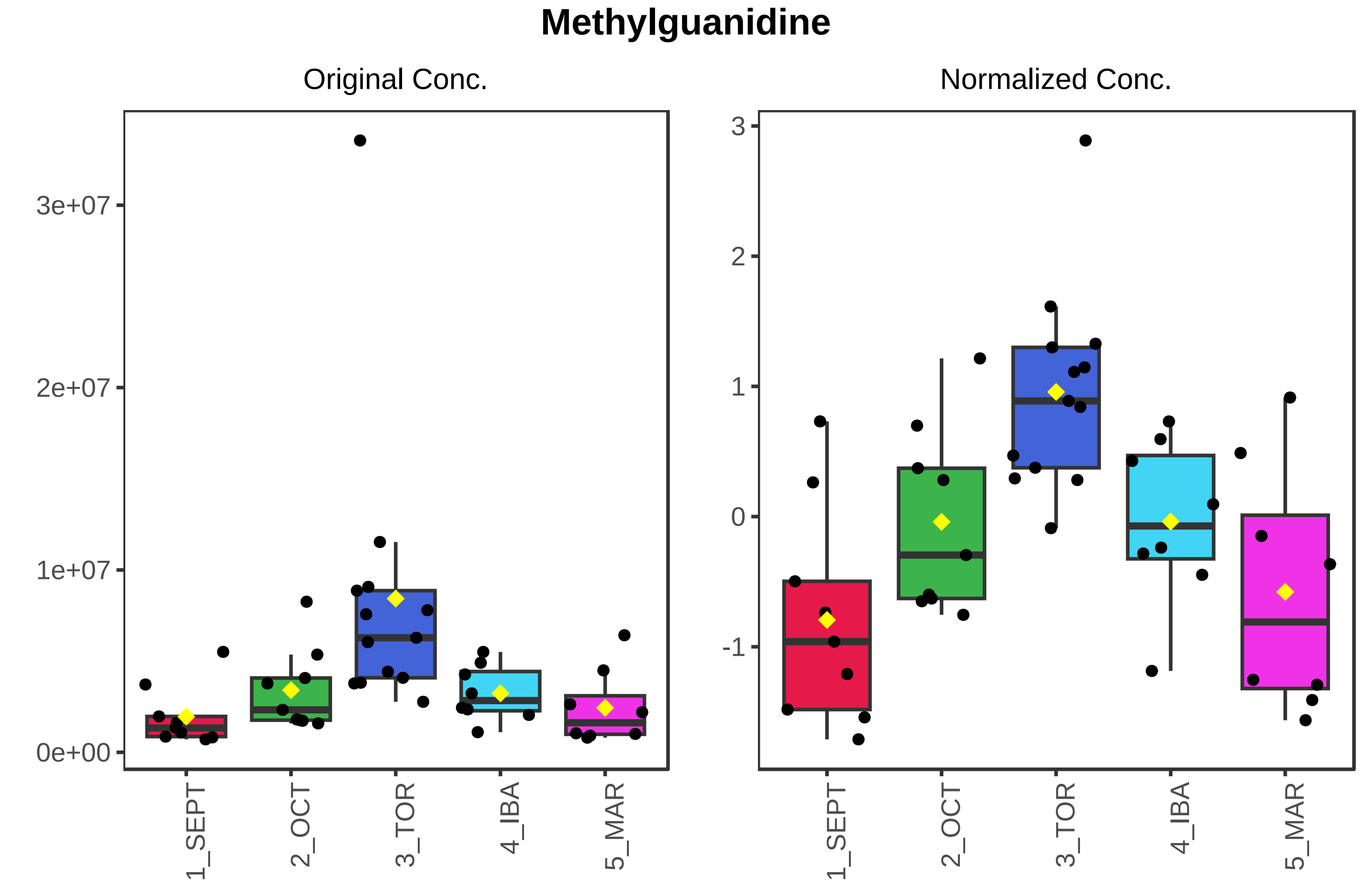

Methylguanidine
Original Conc.
Normalized Conc.
3e+07
2e+07
1e+07
0e+00
3
2
1
0
-1
1_SEPT
2_OCT
3_TOR
4_IBA
5_MAR

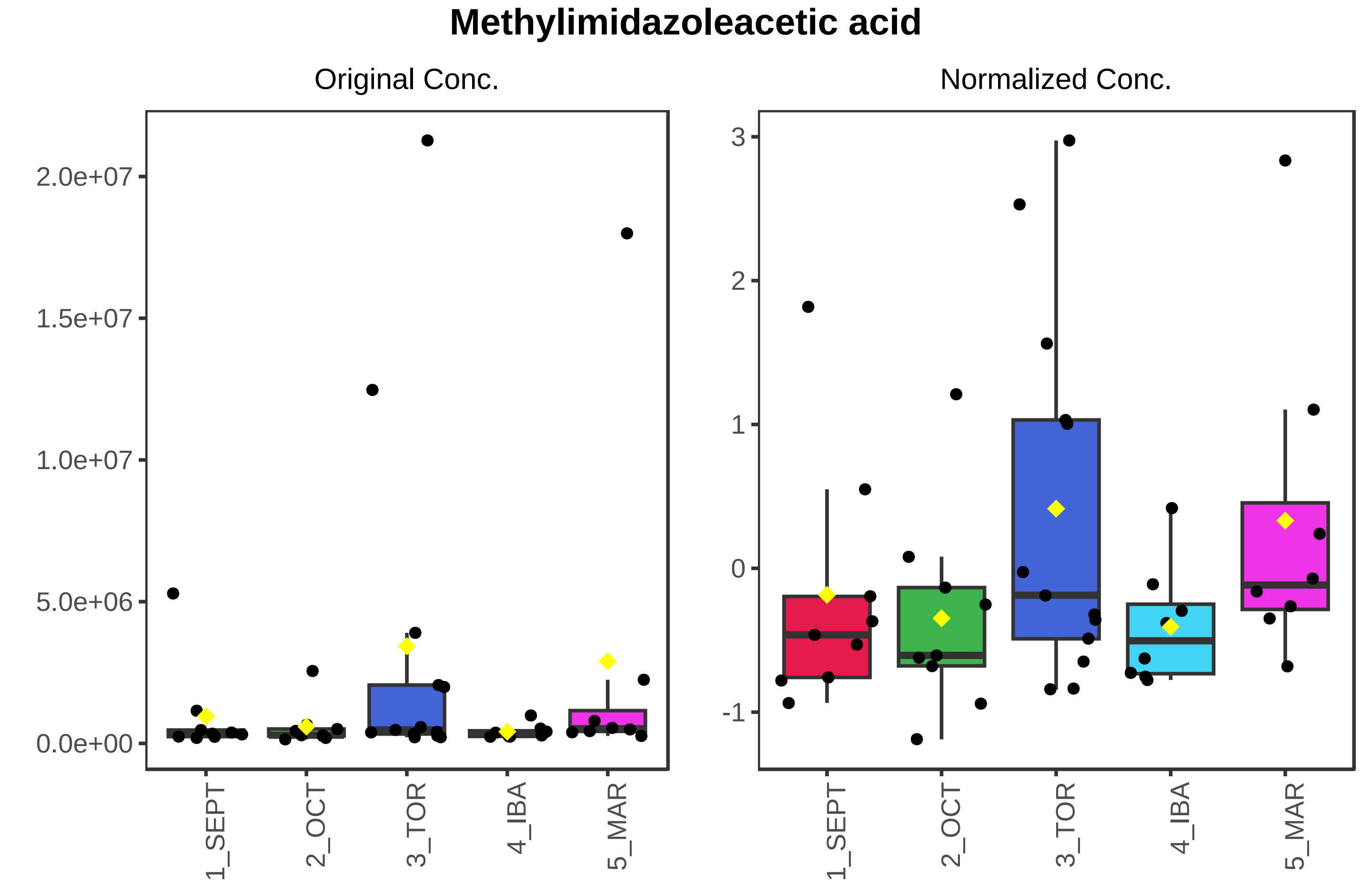

Methylimidazoleacetic acid
Original Conc.
Normalized Conc.
2.0e+07
1.5e+07
1.0e+07
5.0e+06
0.0e+00
3
2
1
0
-1
1_SEPT
2_OCT
3_TOR
4_IBA
5_MAR

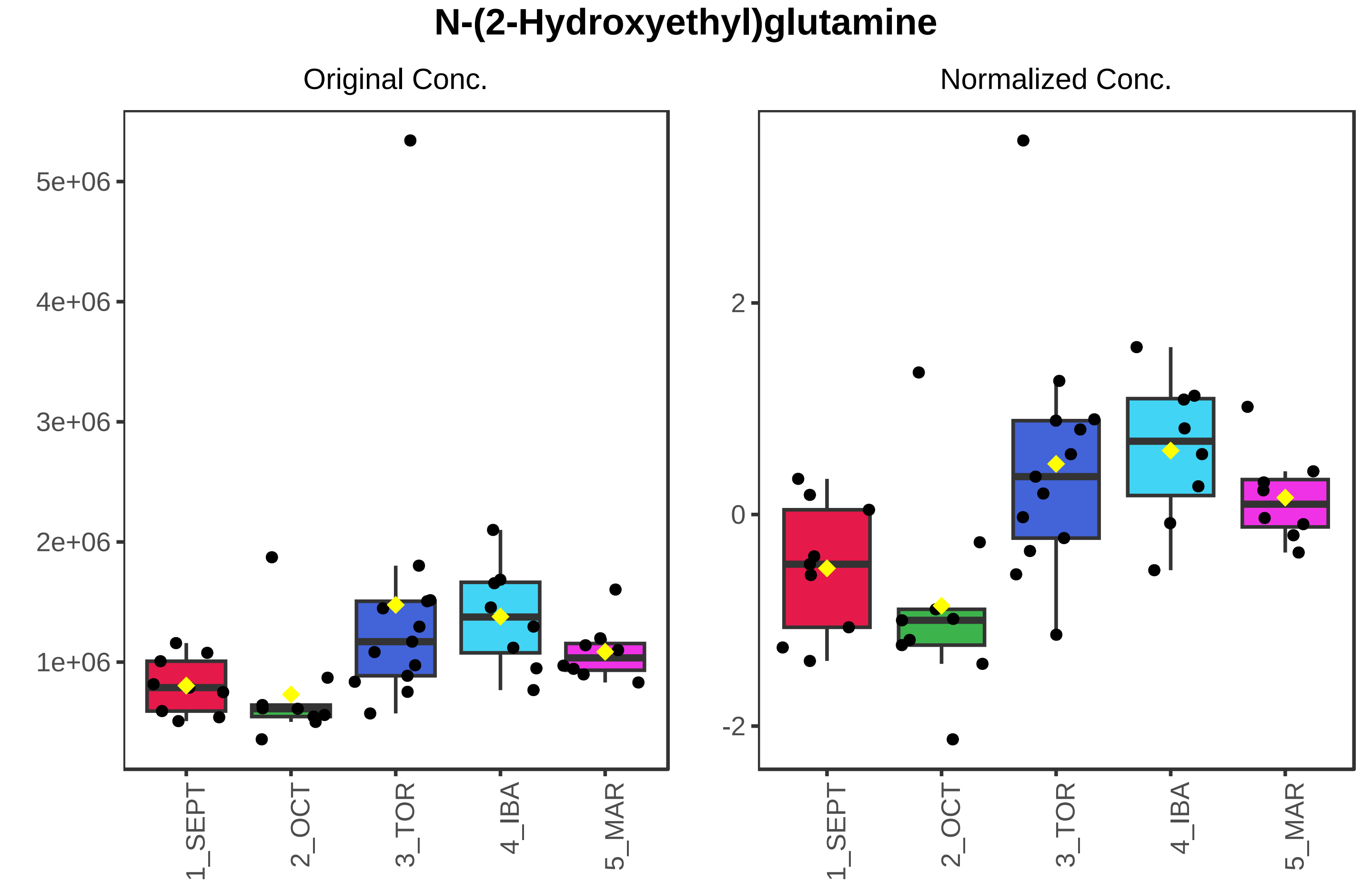

N-(2-Hydroxyethyl)glutamine
Original Conc.
Normalized Conc.
5e+06
4e+06
3e+06
2e+06
1e+06
2
0
-2
1_SEPT
2_OCT
3_TOR
4_IBA
5_MAR

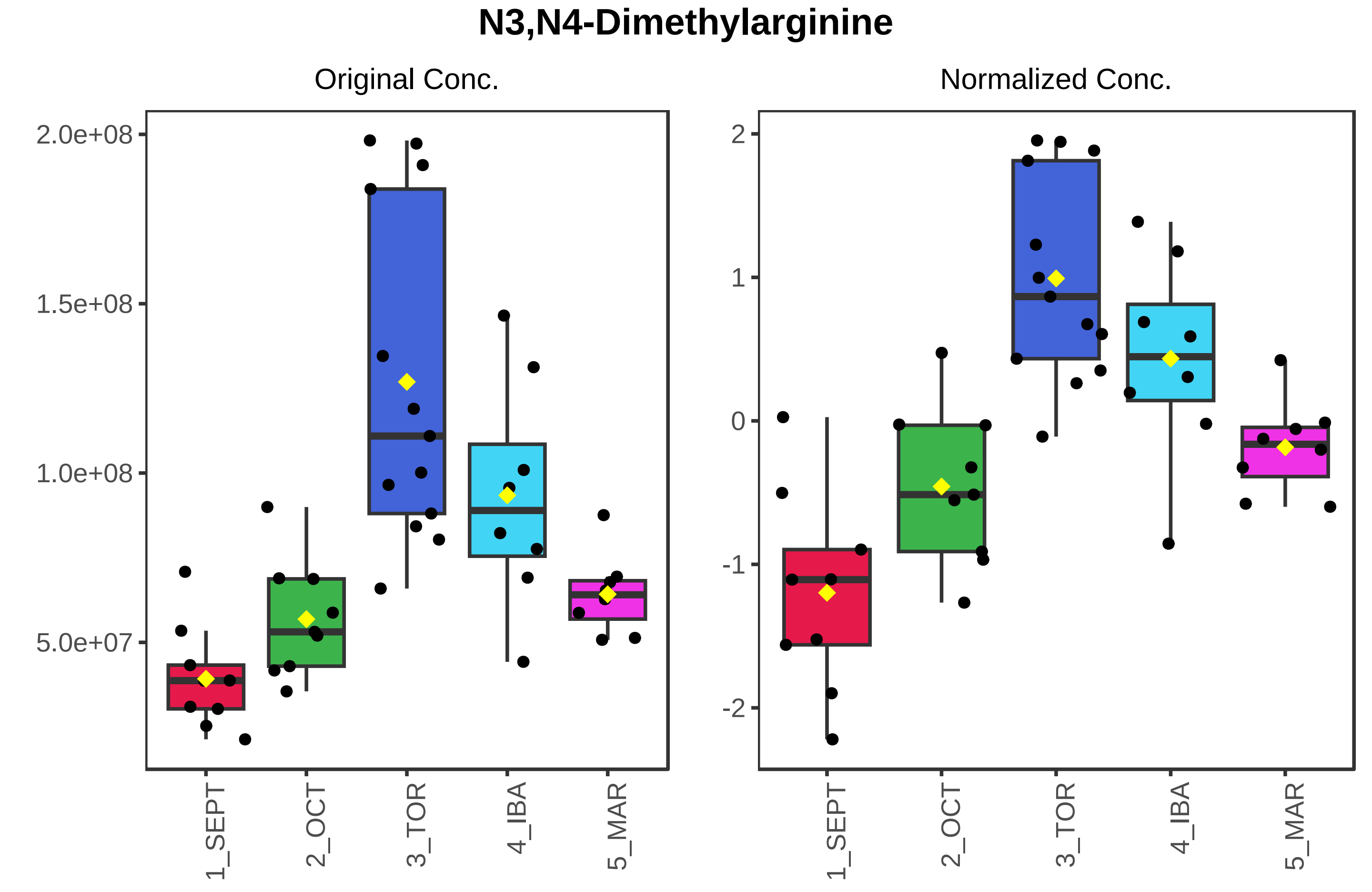
N3,N4-Dimethylarginine
Original Conc.
Normalized Conc.
2.0e+08
1.5e+08
1.0e+08
5.0e+07
2
1
0
-1
-2
1_SEPT
2_OCT
3_TOR
4_IBA
5_MAR

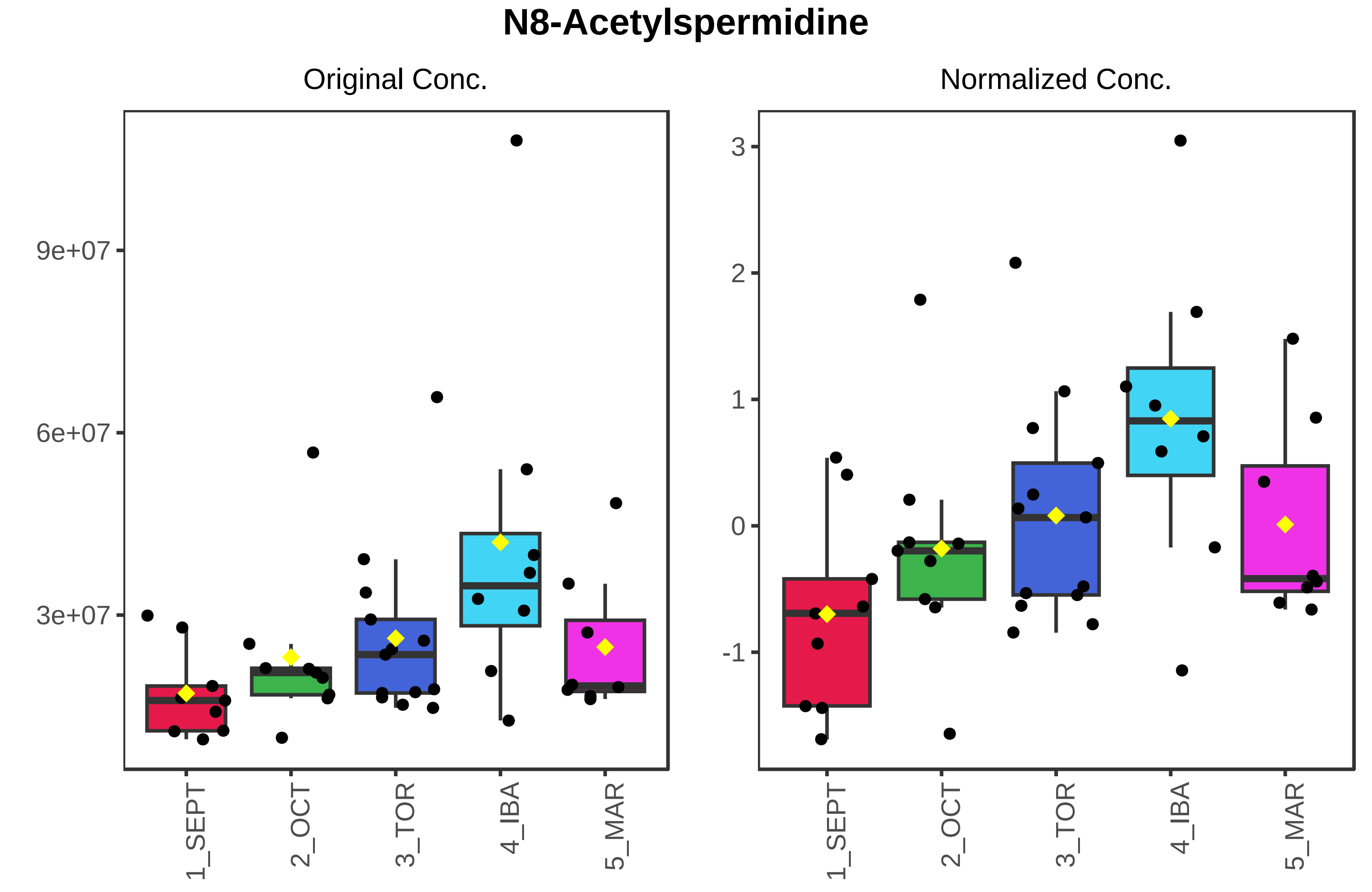
N8-Acetylspermidine
Original Conc.
Normalized Conc.
9e+07
6e+07
3e+07
3
2
1
0
-1
1_SEPT
2_OCT
3_TOR
4_IBA
5_MAR
1_SEPT
2_OCT
3_TOR
4_IBA
5_MAR

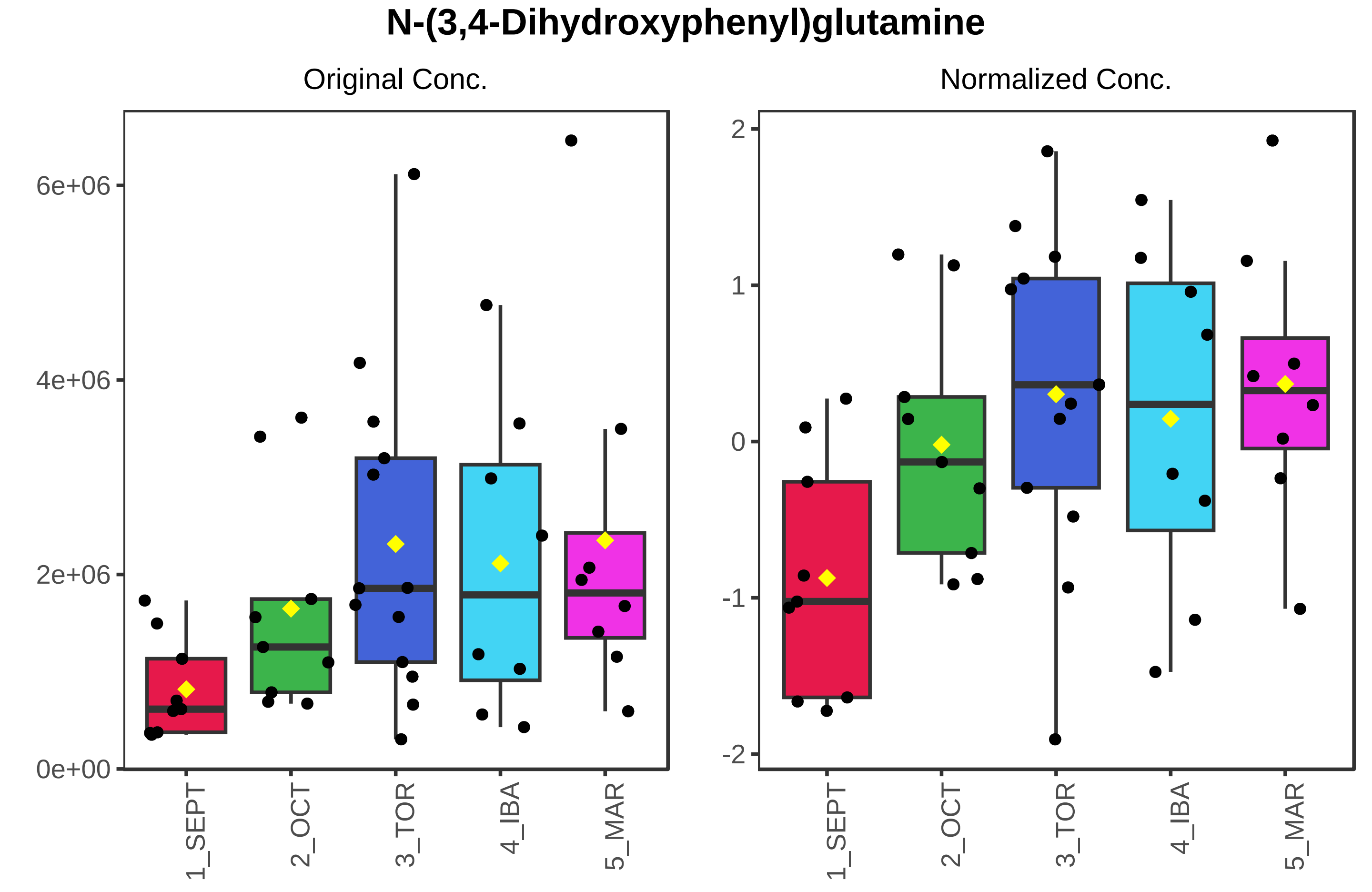

N-(3,4-Dihydroxyphenyl)glutamine
Original Conc.
Normalized Conc.
6e+06
4e+06
2e+06
0e+00
2
1
0
-1
-2
1_SEPT
2_OCT
3_TOR
4_IBA
5_MAR

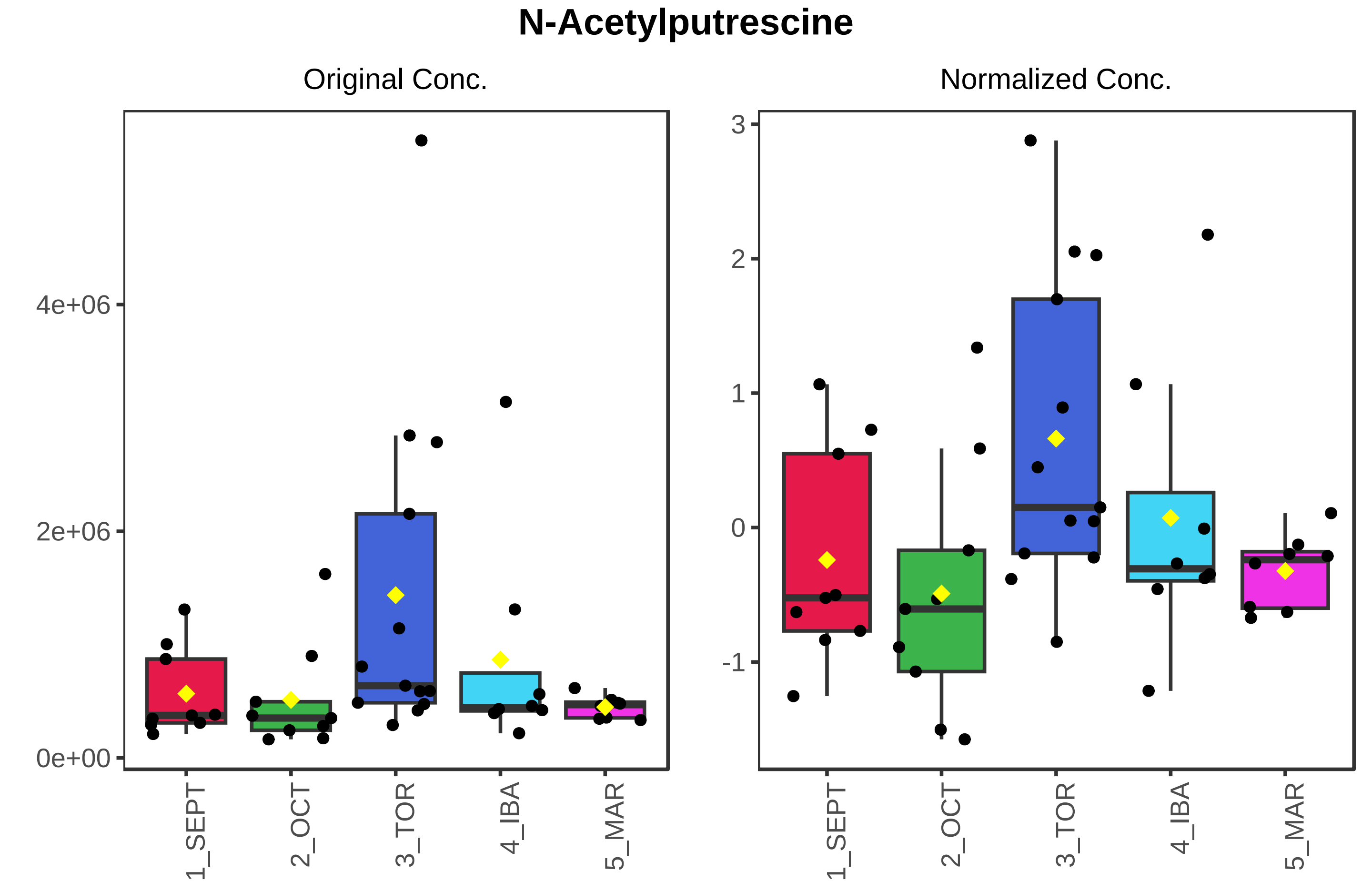

N-Acetylputrescine
Original Conc.
Normalized Conc.
4e+06
2e+06
0e+00
3
2
1
0
-1
1_SEPT
2_OCT
3_TOR
4_IBA
5_MAR

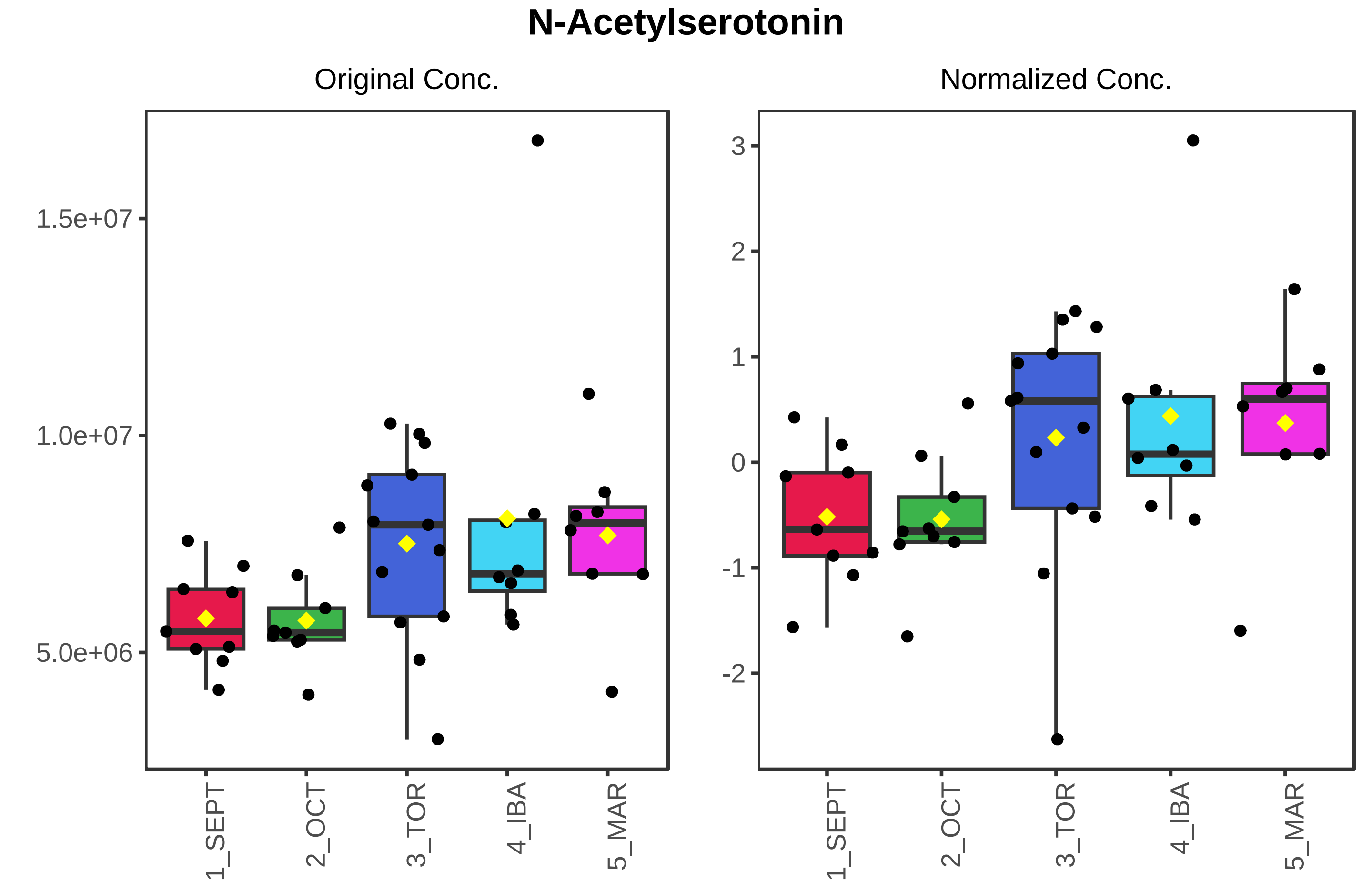
N-Acetylserotonin
Original Conc.
Normalized Conc.
1.5e+07
1.0e+07
5.0e+06
3
2
1
0
-1
-2
1_SEPT
2_OCT
3_TOR
4_IBA
5_MAR

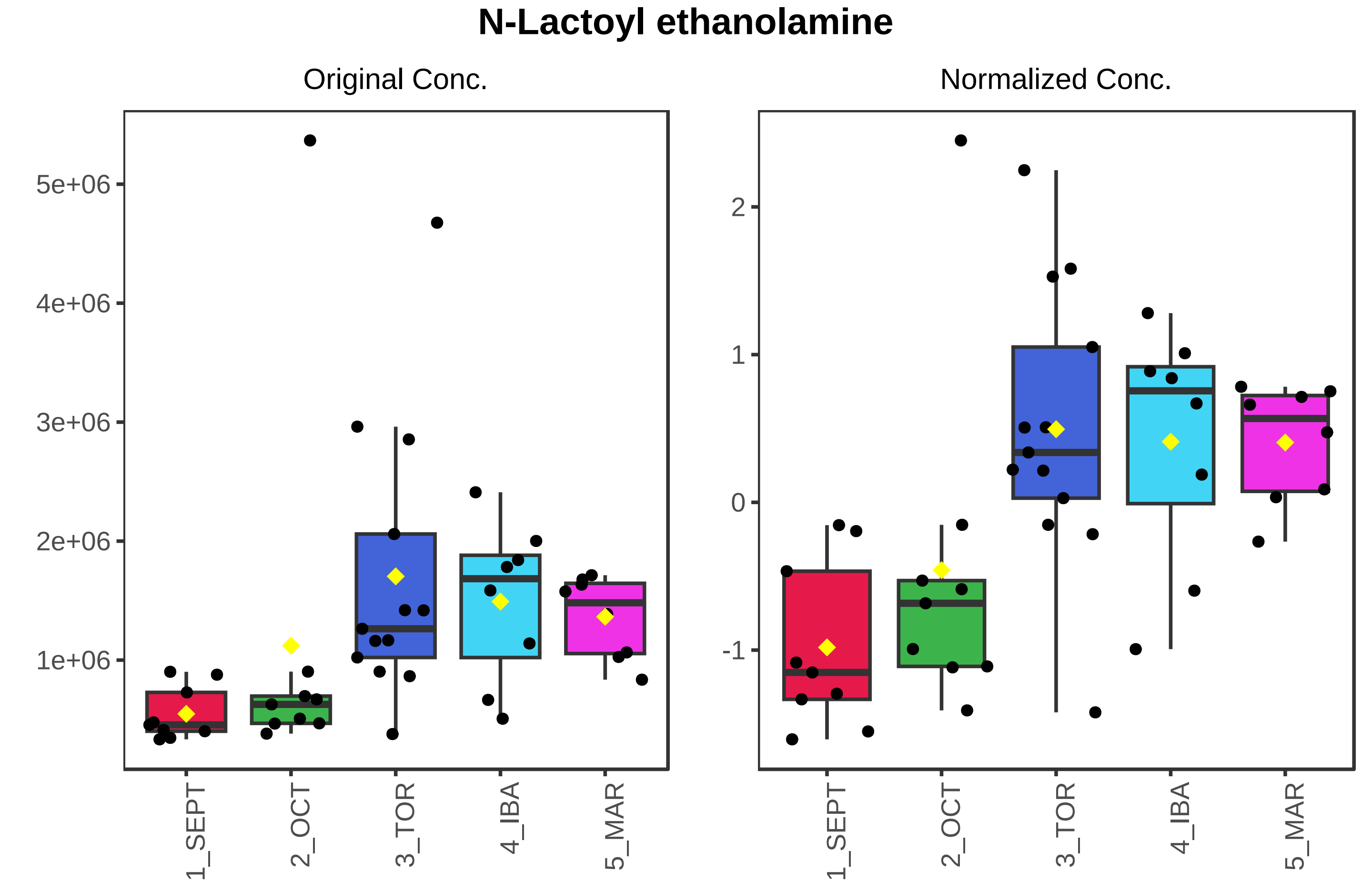

N-Lactoyl ethanolamine
Original Conc.
Normalized Conc.
5e+06
4e+06
3e+06
2e+06
1e+06
2
1
0
-1
1_SEPT
2_OCT
3_TOR
4_IBA
5_MAR

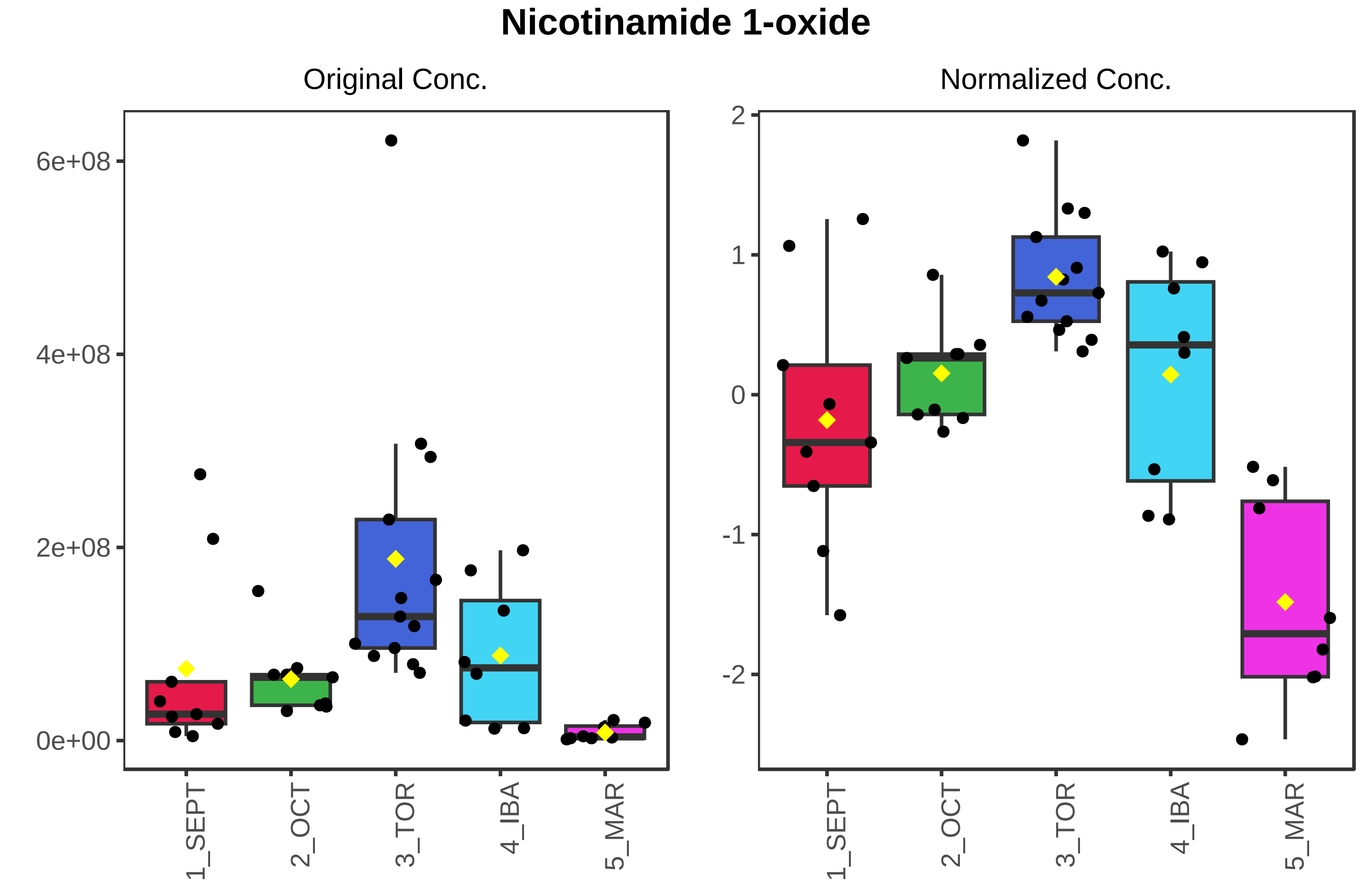

Nicotinamide 1-oxide
Original Conc.
Normalized Conc.
6e+08
4e+08
2e+08
0e+00
2
1
0
-1
-2
1_SEPT
2_OCT
3_TOR
4_IBA
5_MAR

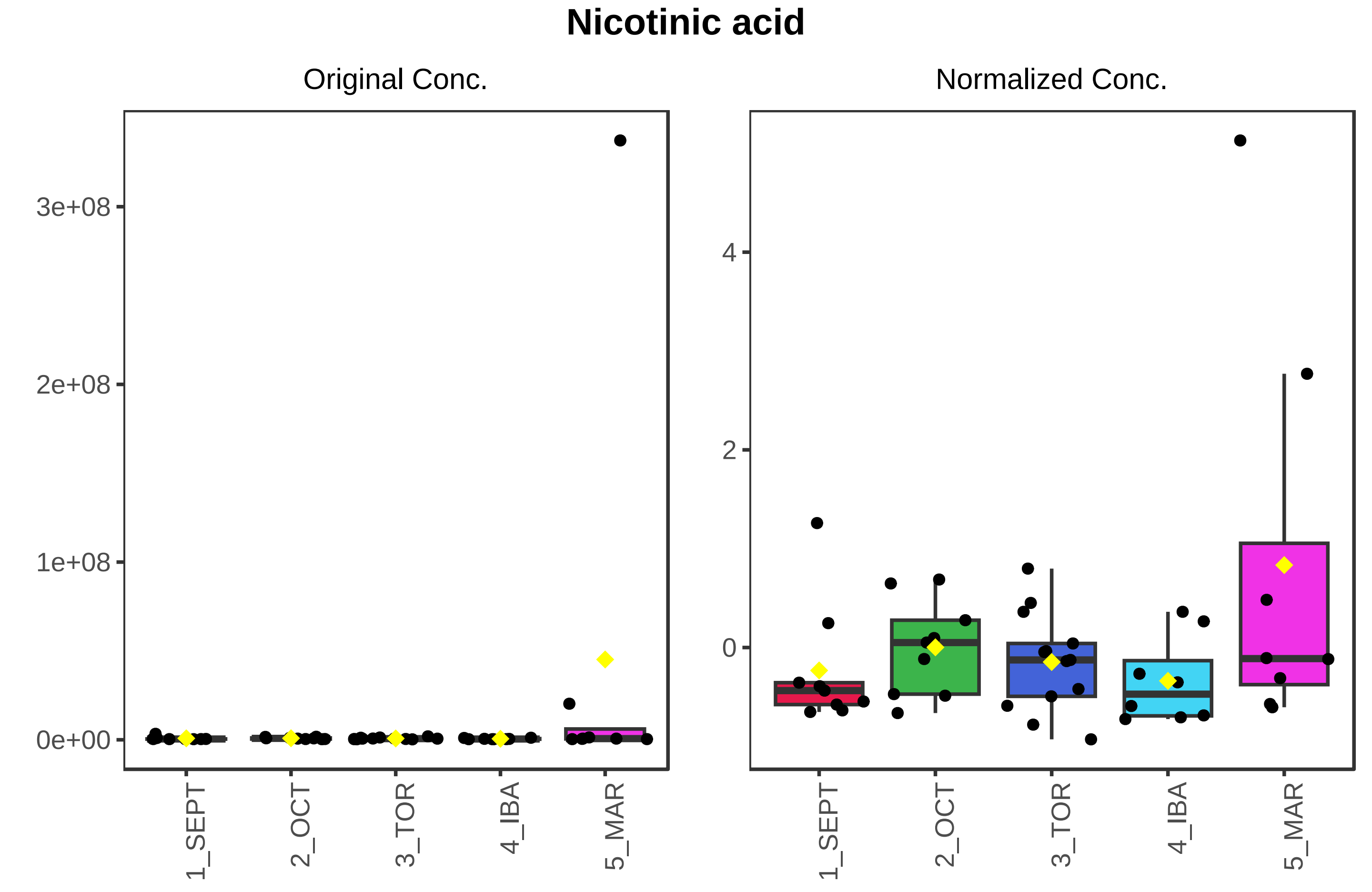
Nicotinic acid
Original Conc.
Normalized Conc.
3e+08
2e+08
1e+08
0e+00
4
2
0
1_SEPT
2_OCT
3_TOR
4_IBA
5_MAR

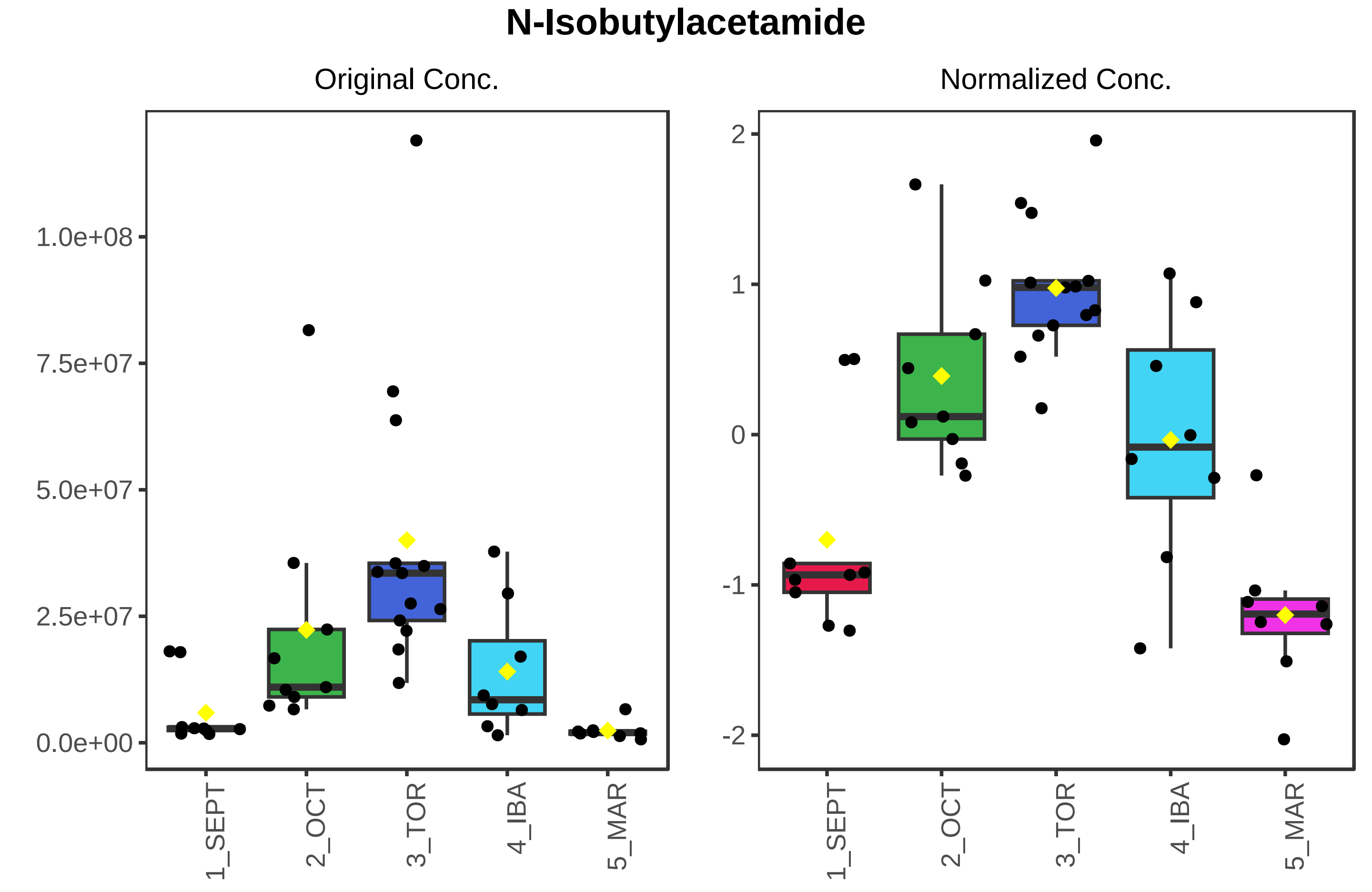
N-Isobutylacetamide
Original Conc.
Normalized Conc.
1.0e+08
7.5e+07
5.0e+07
2.5e+07
0.0e+00
2
1
0
-1
-2
1_SEPT
2_OCT
3_TOR
4_IBA
5_MAR

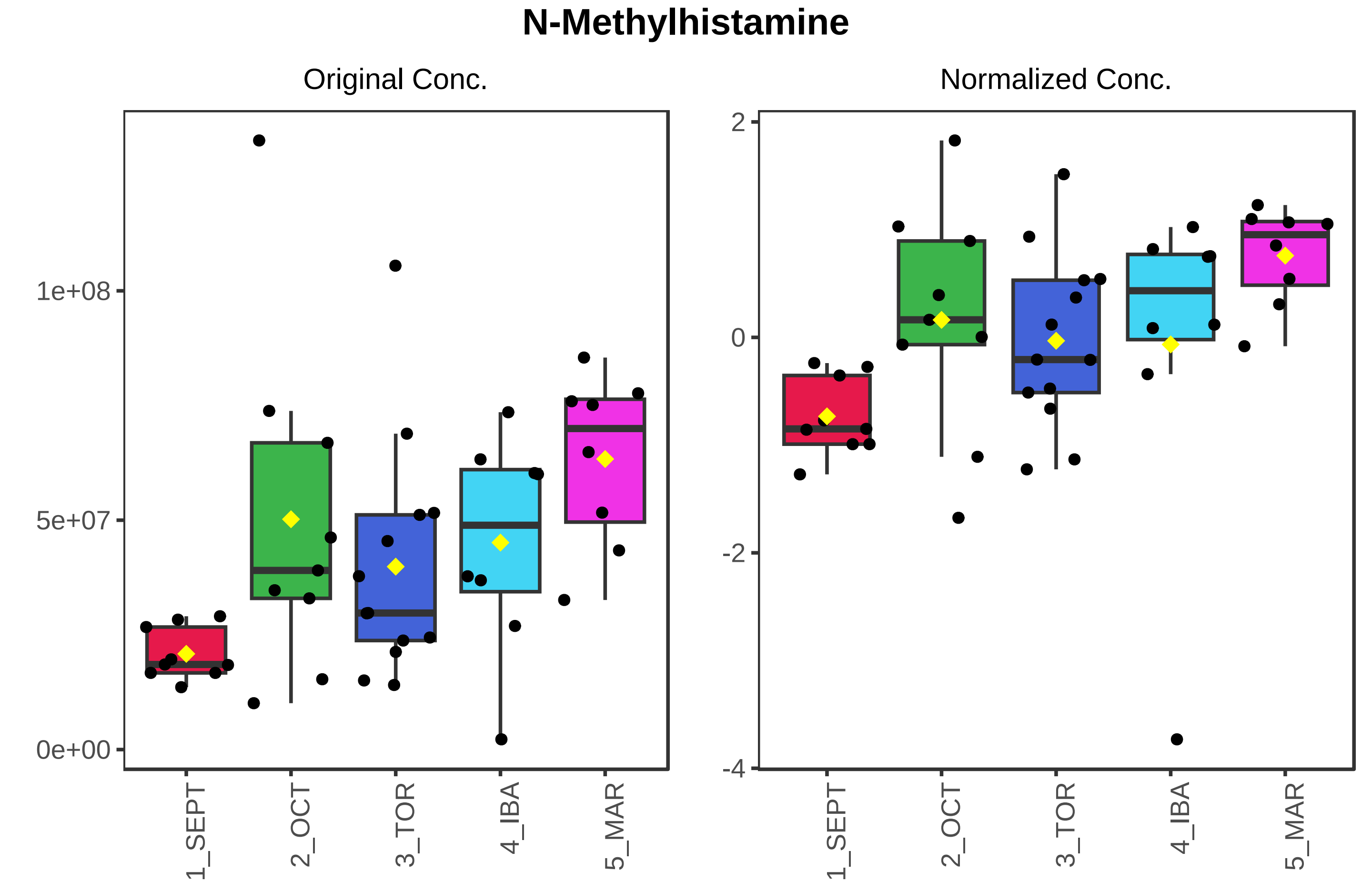

N-Methylhistamine
Original Conc.
Normalized Conc.
1e+08
5e+07
0e+00
2
0
-2
-4
1_SEPT
2_OCT
3_TOR
4_IBA
5_MAR
1_SEPT
2_OCT
3_TOR
4_IBA
5_MAR

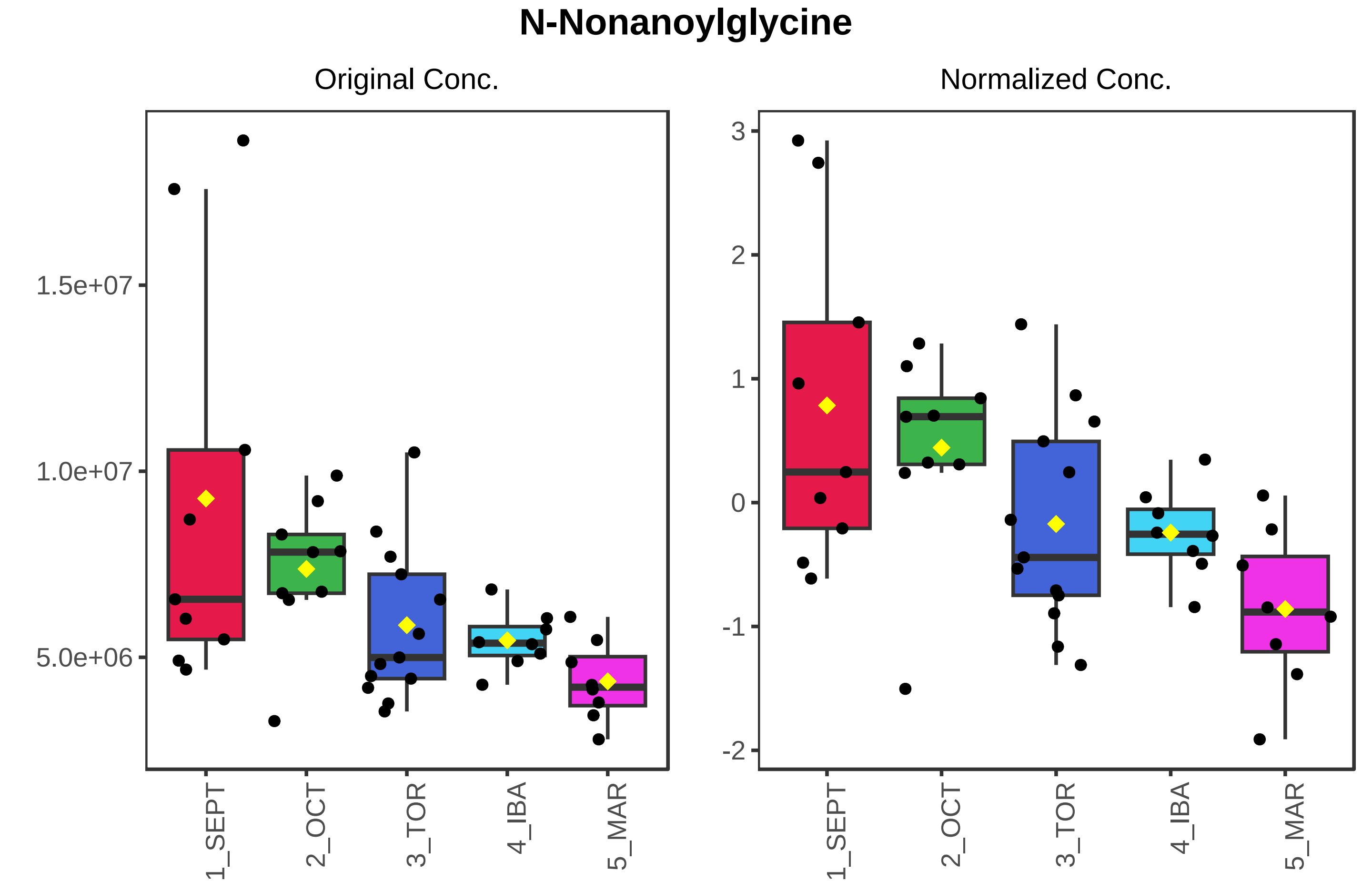
N-Nonanoylglycine
Original Conc.
Normalized Conc.
1.5e+07
1.0e+07
5.0e+06
3
2
1
0
-1
-2
1_SEPT
2_OCT
3_TOR
4_IBA
5_MAR

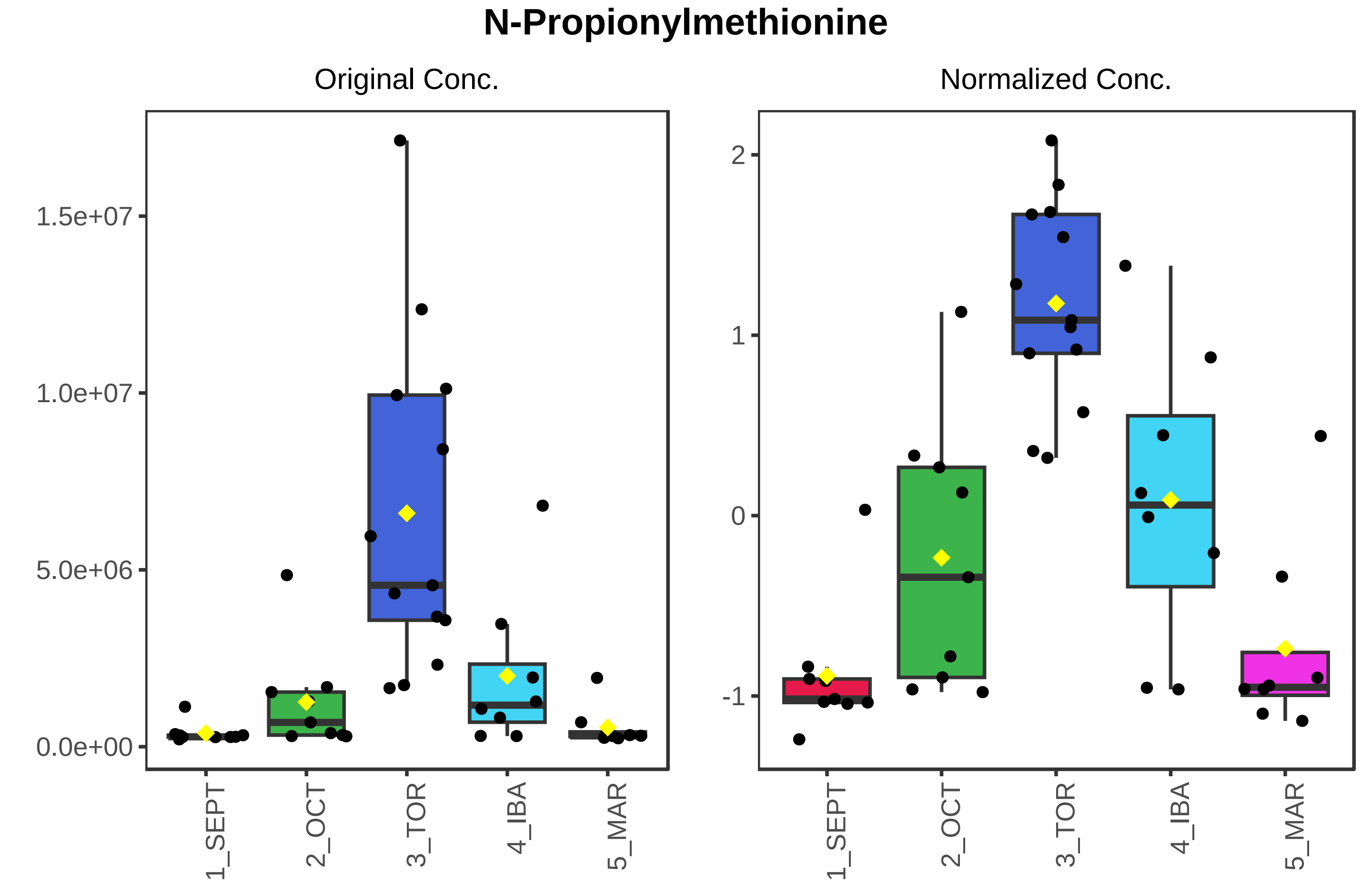

N-Propionylmethionine
Original Conc.
Normalized Conc.
1.5e+07
1.0e+07
5.0e+06
0.0e+00
2
1
0
-1
1_SEPT
2_OCT
3_TOR
4_IBA
5_MAR

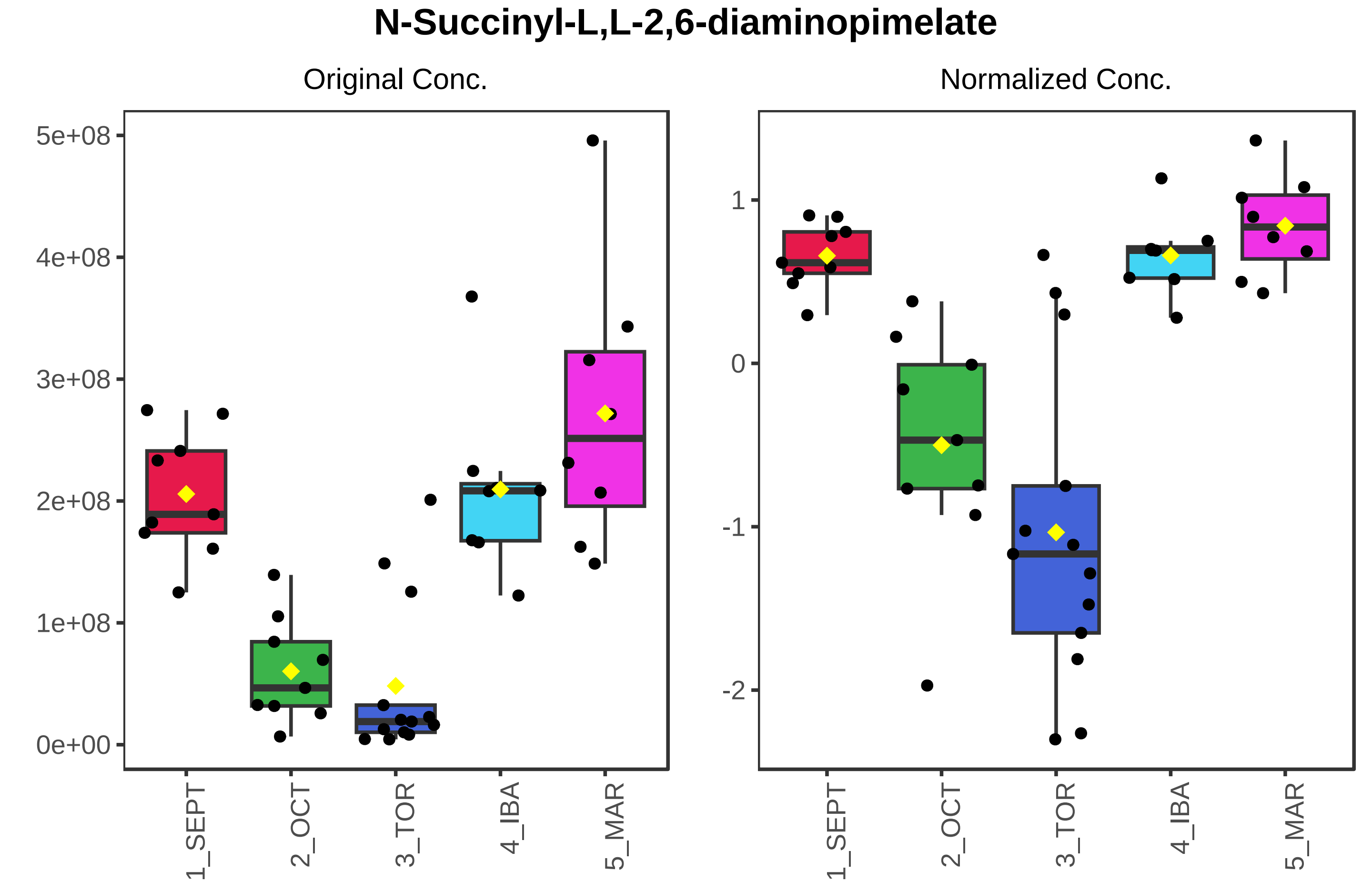

N-Succinyl-L,L-2,6-diaminopimelate
Original Conc.
Normalized Conc.
5e+08
4e+08
3e+08
2e+08
1e+08
0e+00
1
0
-1
-2
1_SEPT
2_OCT
3_TOR
4_IBA
5_MAR

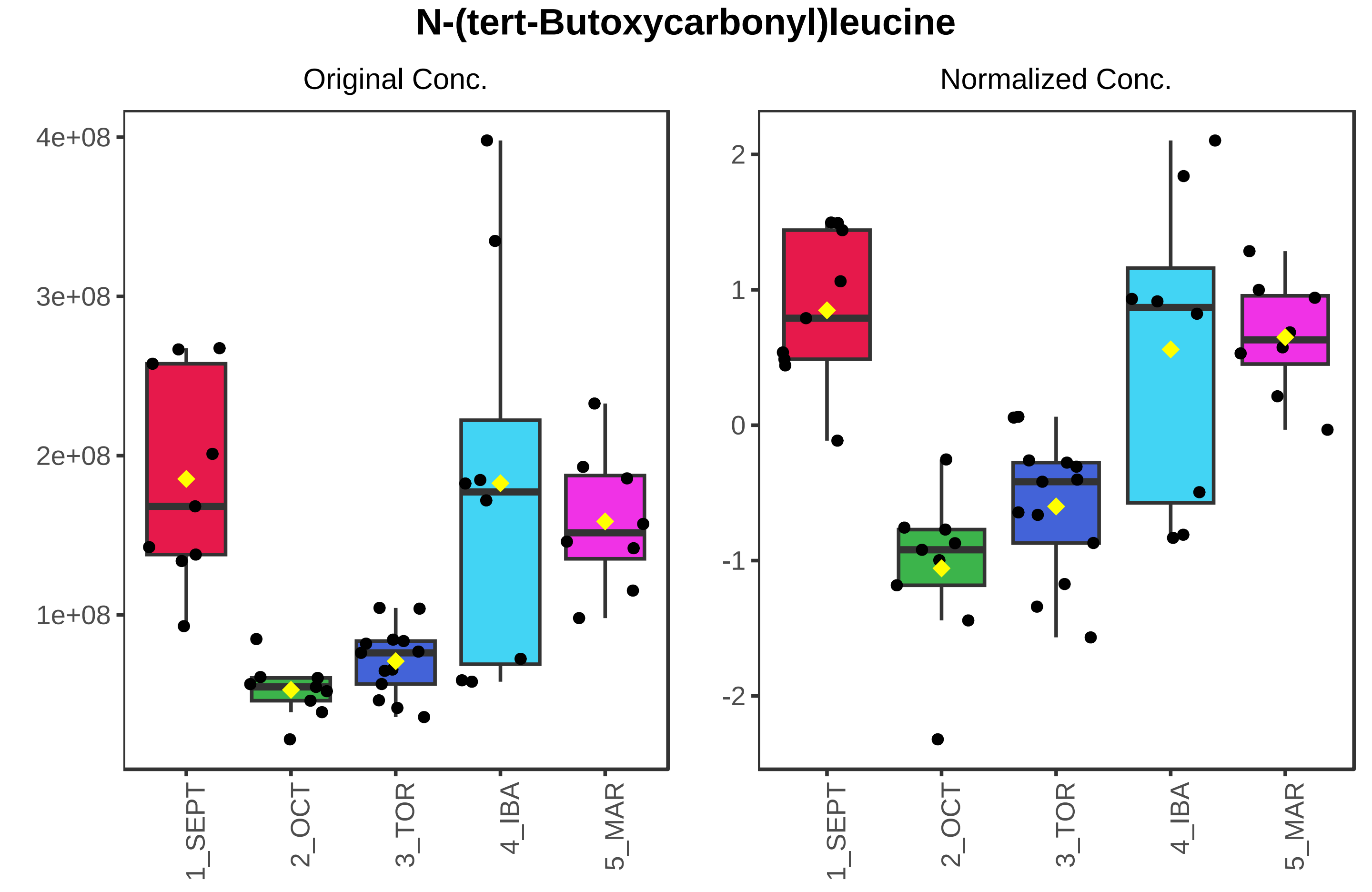

N-(tert-Butoxycarbonyl)leucine
Original Conc.
Normalized Conc.
4e+08
3e+08
2e+08
1e+08
2
1
0
-1
-2
1_SEPT
2_OCT
3_TOR
4_IBA
5_MAR

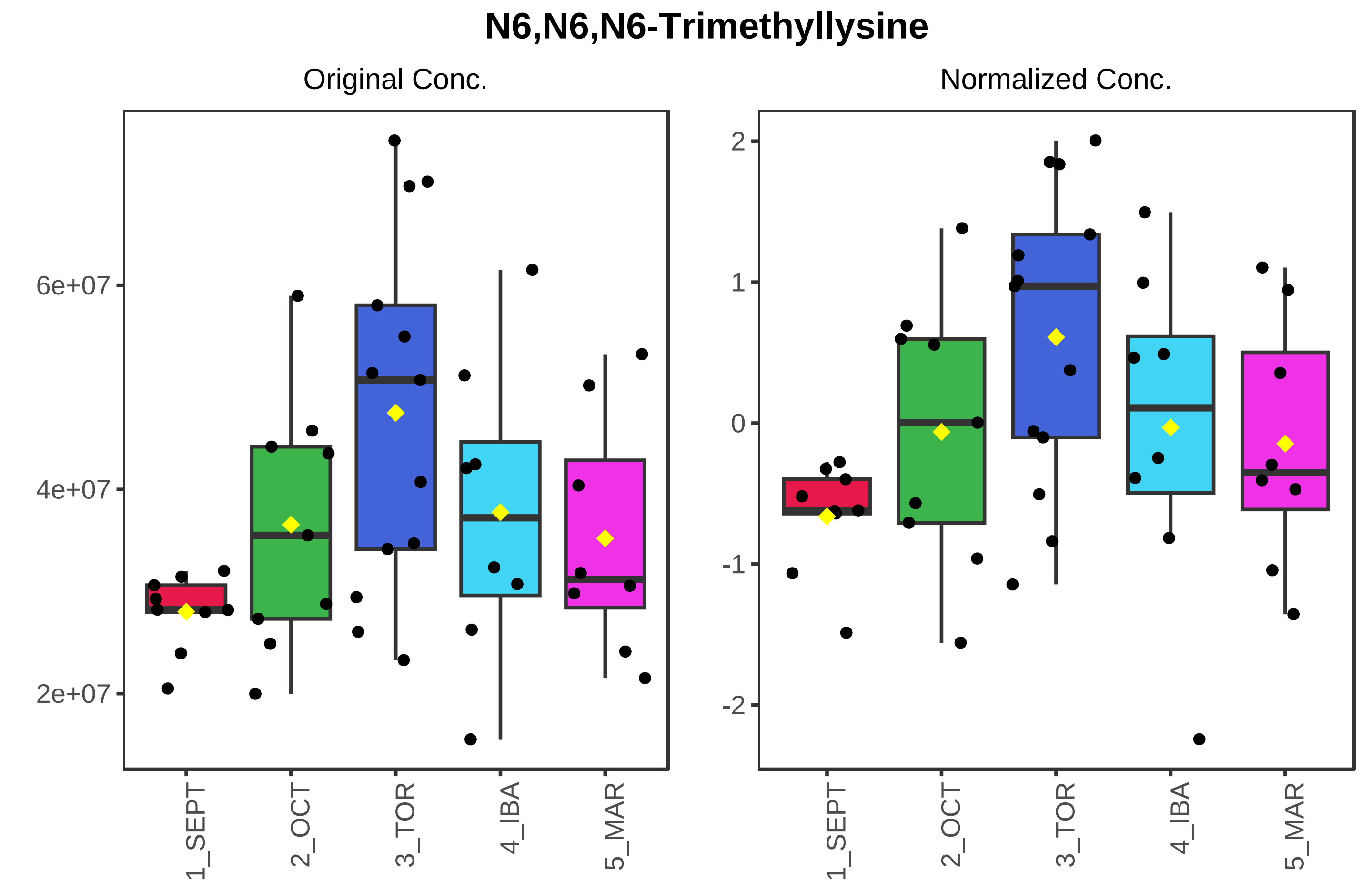
N6,N6,N6-Trimethyllysine
Original Conc.
Normalized Conc.
6e+07
4e+07
2e+07
2
1
0
-1
-2
1_SEPT
2_OCT
3_TOR
4_IBA
5_MAR
1_SEPT
2_OCT
3_TOR
4_IBA
5_MAR

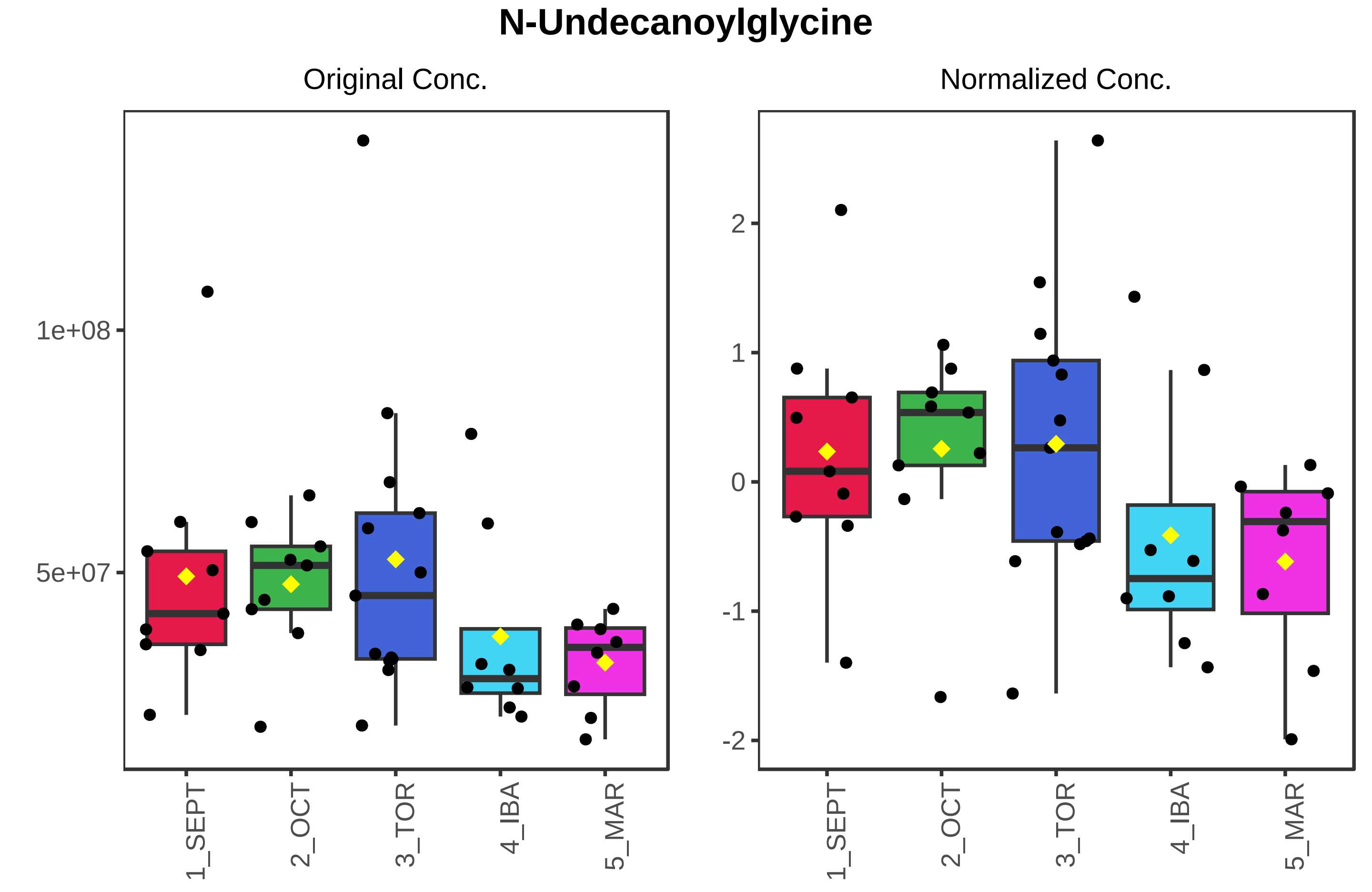
N-Undecanoylglycine
Original Conc.
Normalized Conc.
1e+08
5e+07
2
1
0
-1
-2
1_SEPT
2_OCT
3_TOR
4_IBA
5_MAR
1_SEPT
2_OCT
3_TOR
4_IBA
5_MAR

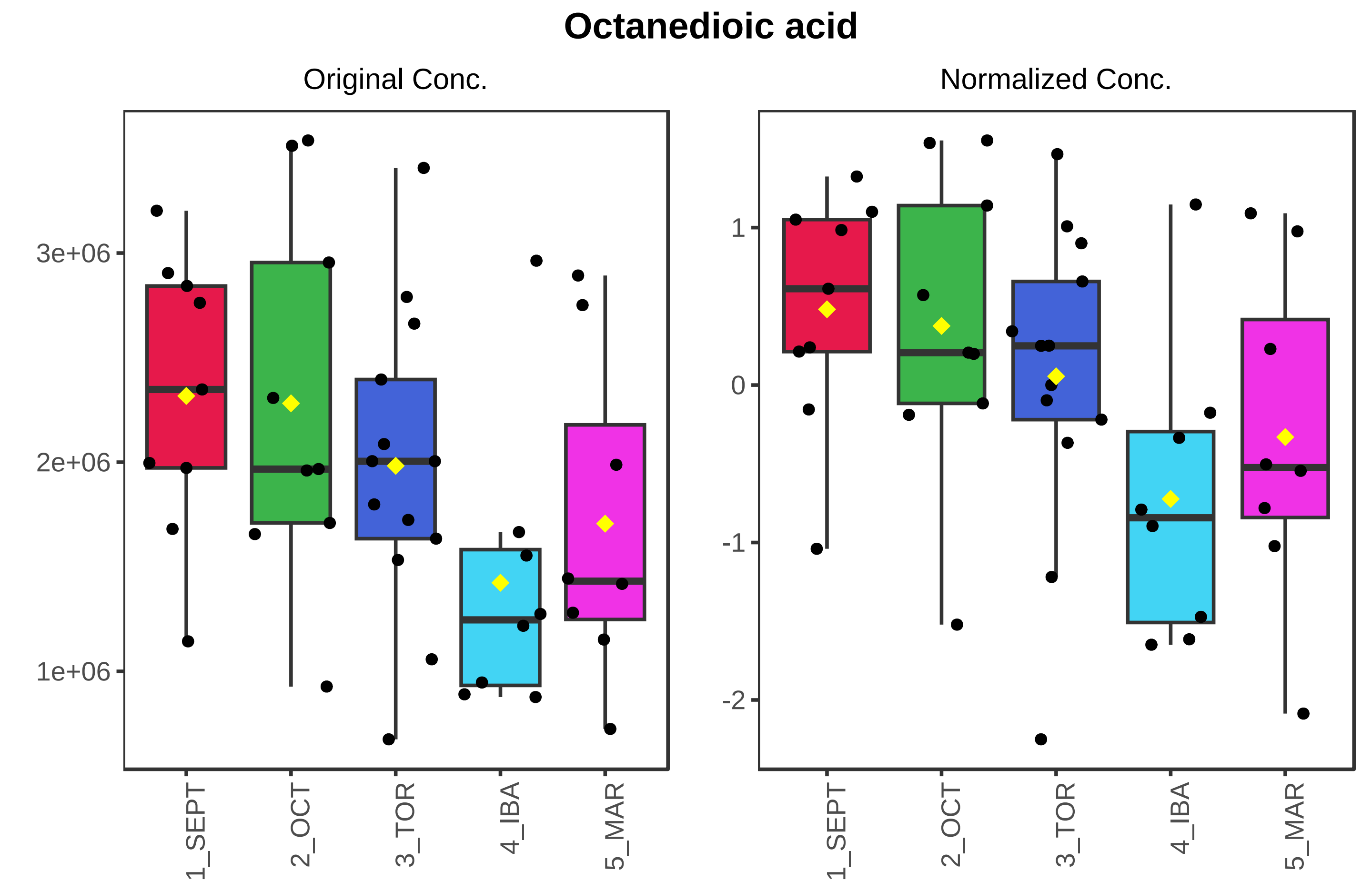

Octanedioic acid
Original Conc.
Normalized Conc.
3e+06
2e+06
1e+06
1
0
-1
-2
1_SEPT
2_OCT
3_TOR
4_IBA
5_MAR
1_SEPT
2_OCT
3_TOR
4_IBA
5_MAR

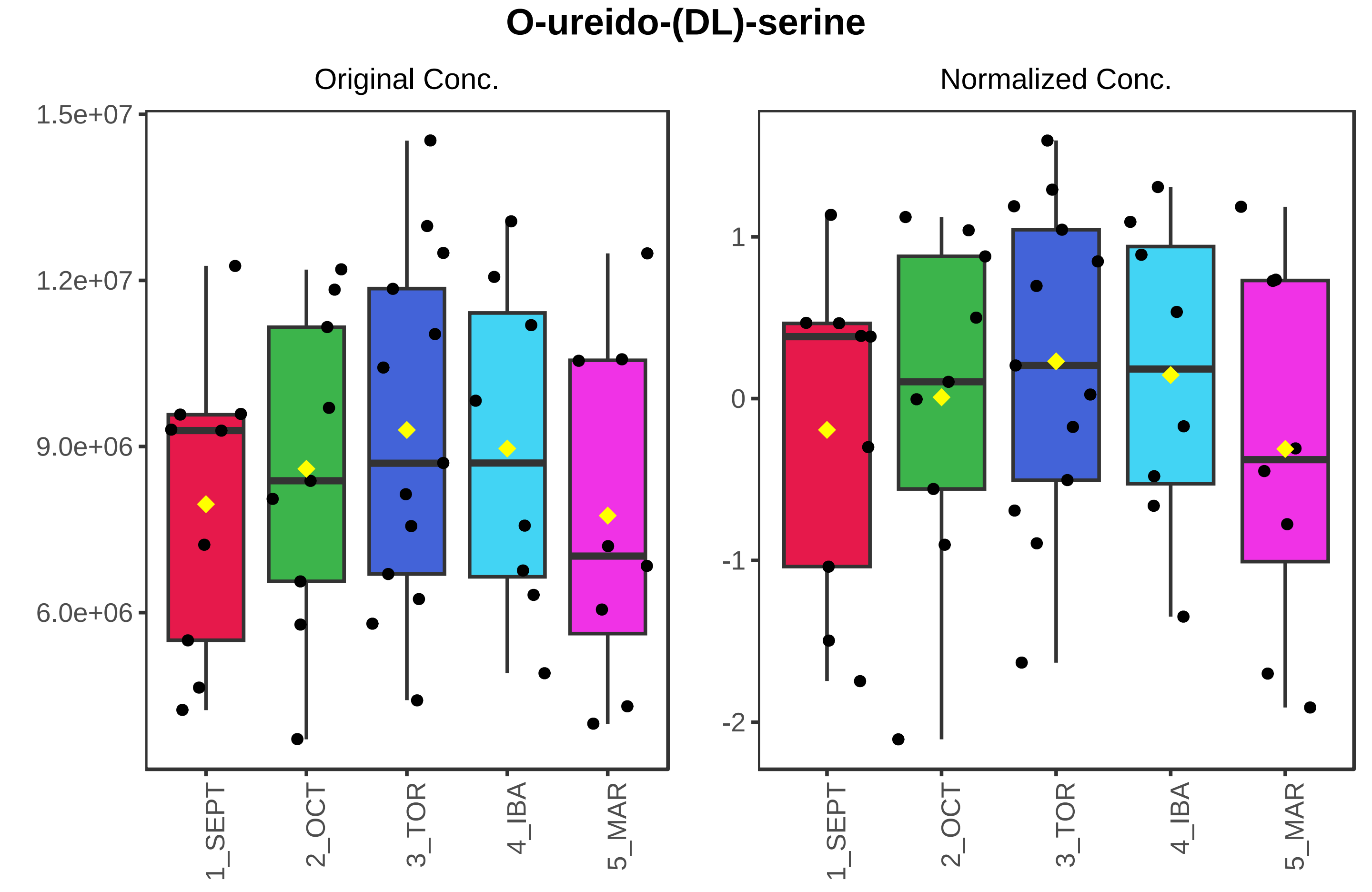

O-ureido-(DL)-serine
Original Conc.
Normalized Conc.
1.5e+07
1.2e+07
9.0e+06
6.0e+06
1
0
-1
-2
1_SEPT
2_OCT
3_TOR
4_IBA
5_MAR

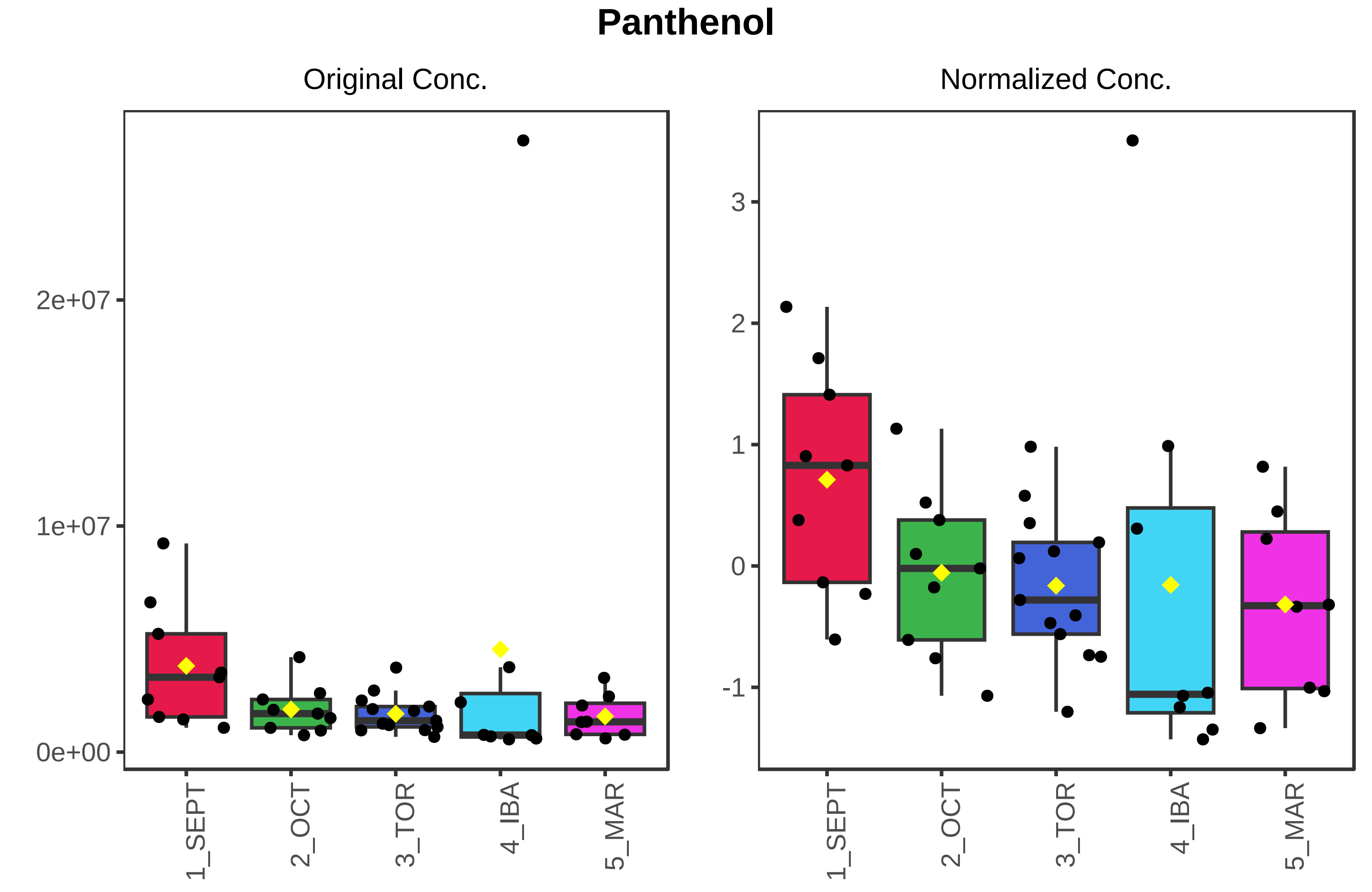

Panthenol
Original Conc.
Normalized Conc.
2e+07
1e+07
0e+00
3
2
1
0
-1
1_SEPT
2_OCT
3_TOR
4_IBA
5_MAR

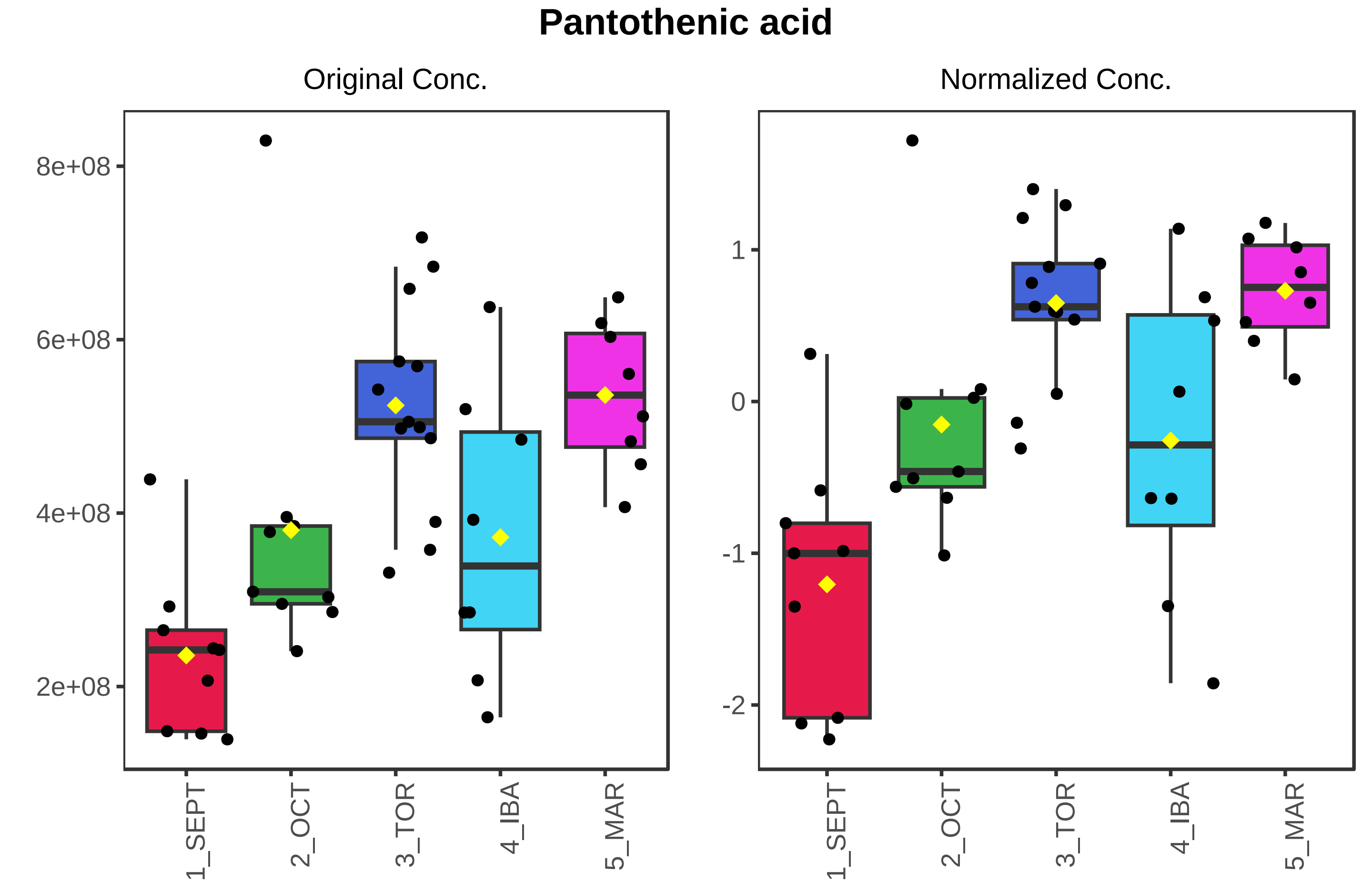
Pantothenic acid
Original Conc.
Normalized Conc.
8e+08
6e+08
4e+08
2e+08
1
0
-1
-2
1_SEPT
2_OCT
3_TOR
4_IBA
5_MAR
1_SEPT
2_OCT
3_TOR
4_IBA
5_MAR

Pentahomomethionine

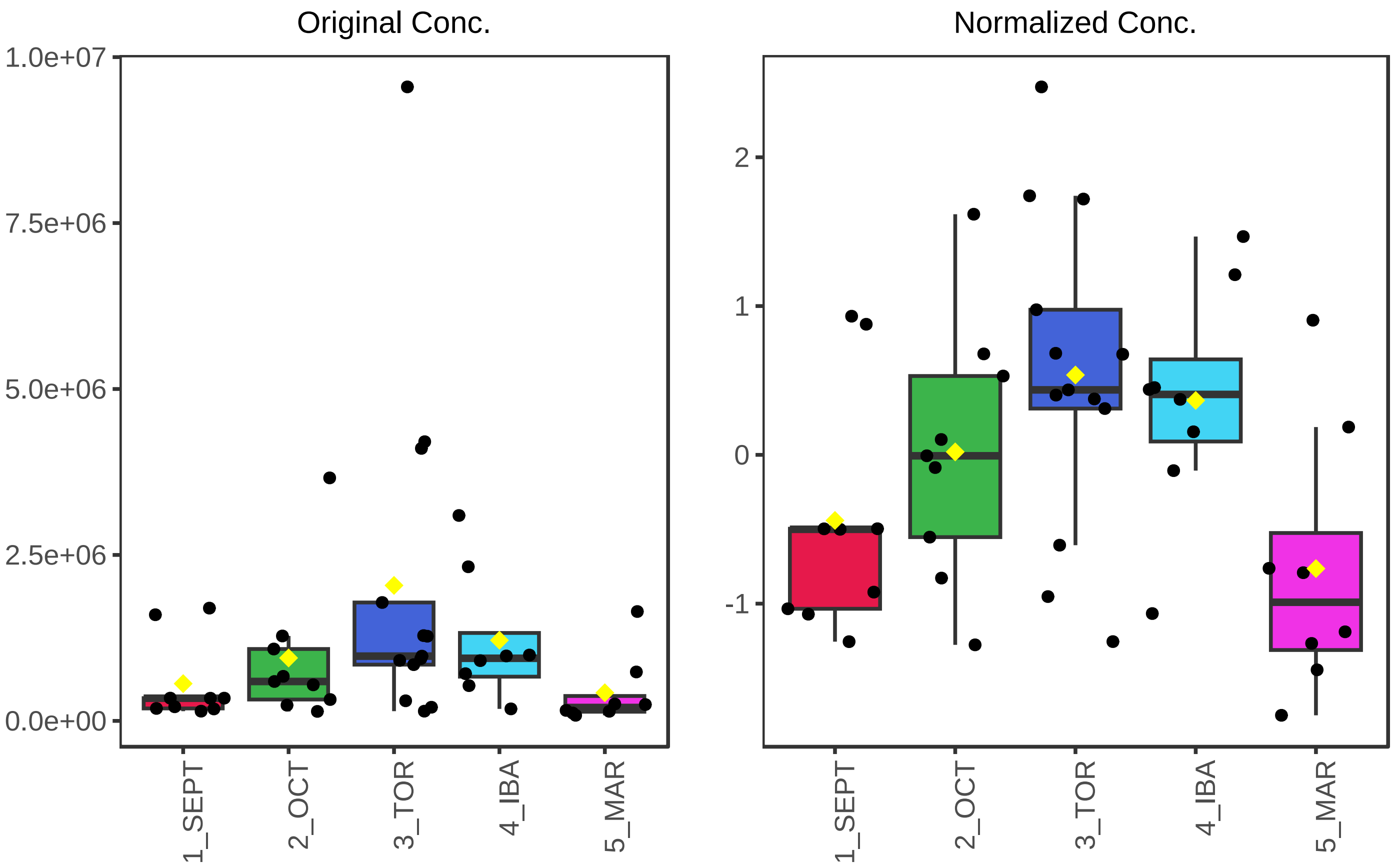

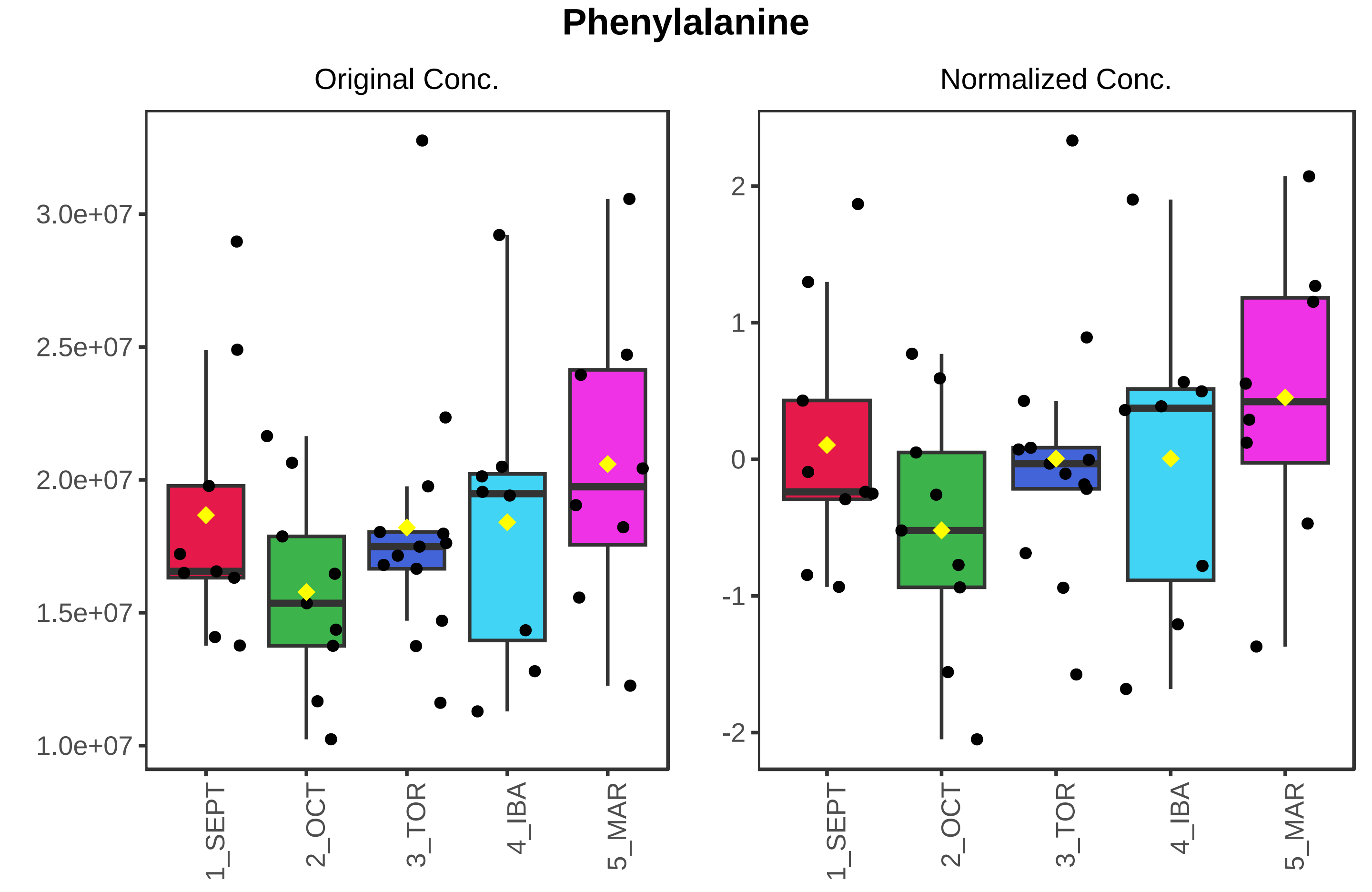

Phenylalanine
Original Conc.
Normalized Conc.
3.0e+07
2.5e+07
2.0e+07
1.5e+07
1.0e+07
2
1
0
-1
-2
1_SEPT
2_OCT
3_TOR
4_IBA
5_MAR

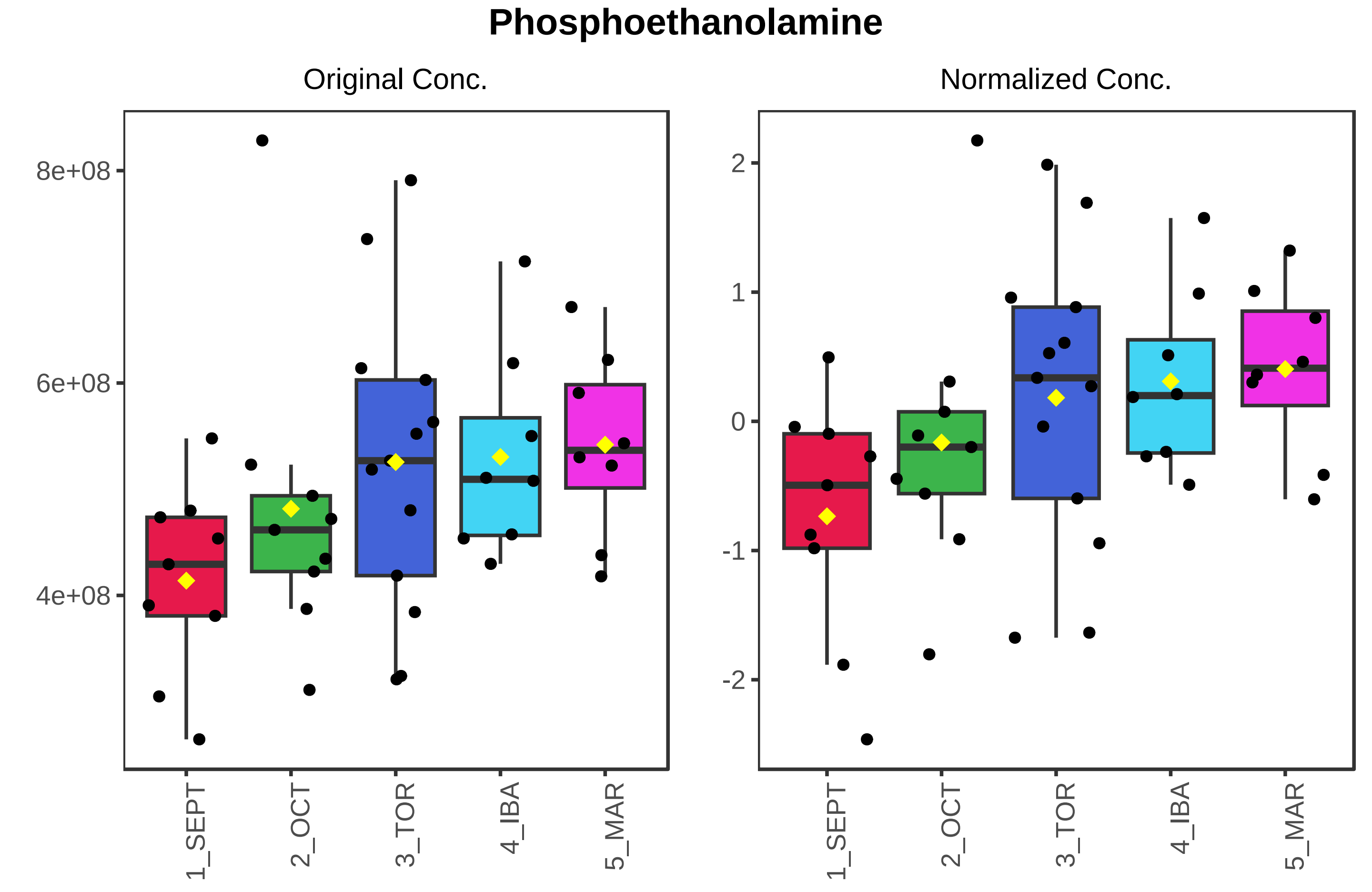
Phosphoethanolamine
Original Conc.
Normalized Conc.
8e+08
6e+08
4e+08
2
1
0
-1
-2
1_SEPT
2_OCT
3_TOR
4_IBA
5_MAR
1_SEPT
2_OCT
3_TOR
4_IBA
5_MAR

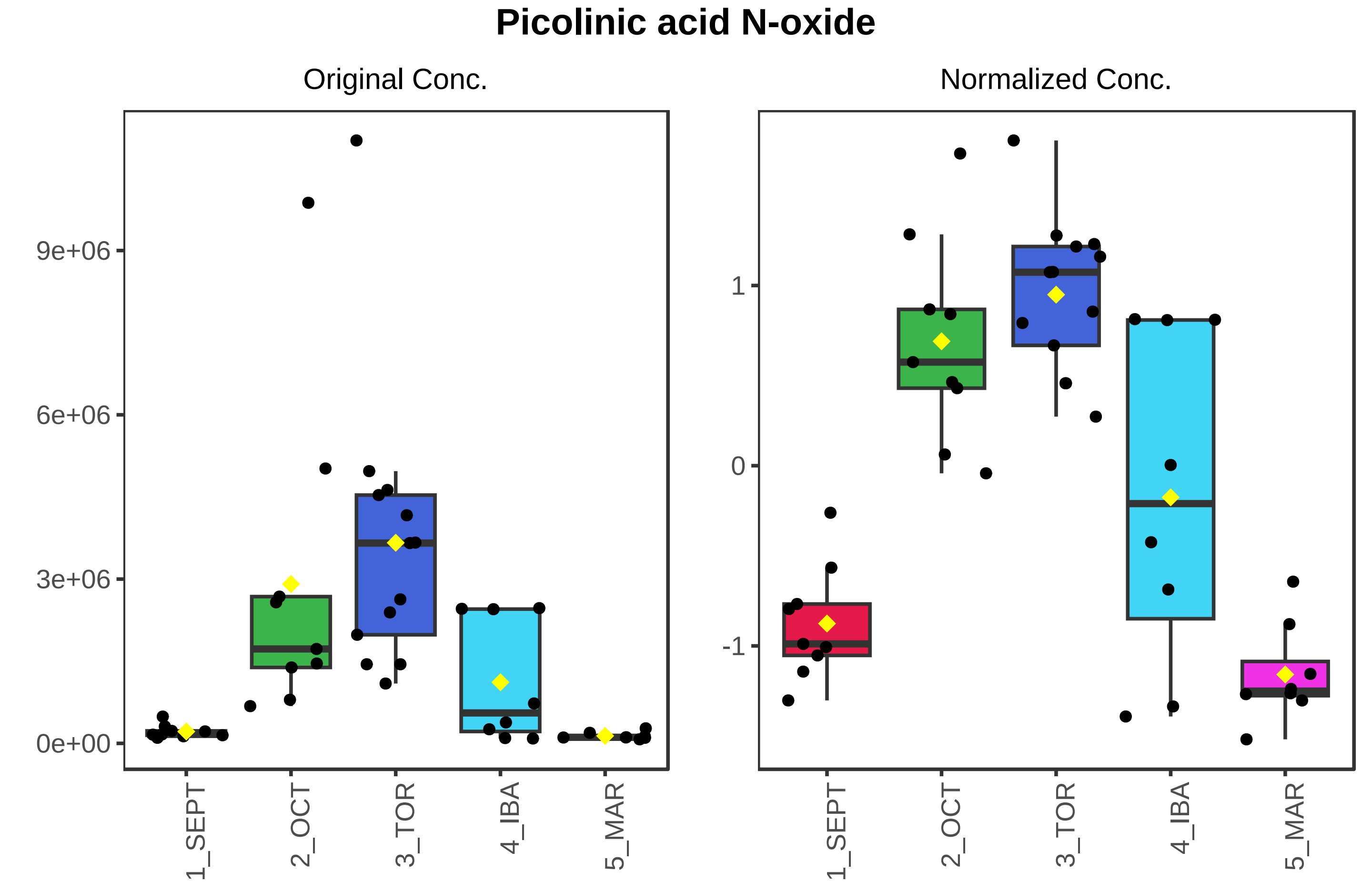
Picolinic acid N-oxide
Original Conc.
Normalized Conc.
9e+06
6e+06
3e+06
0e+00
1
0
-1
1_SEPT
2_OCT
3_TOR
4_IBA
5_MAR
1_SEPT
2_OCT
3_TOR
4_IBA
5_MAR

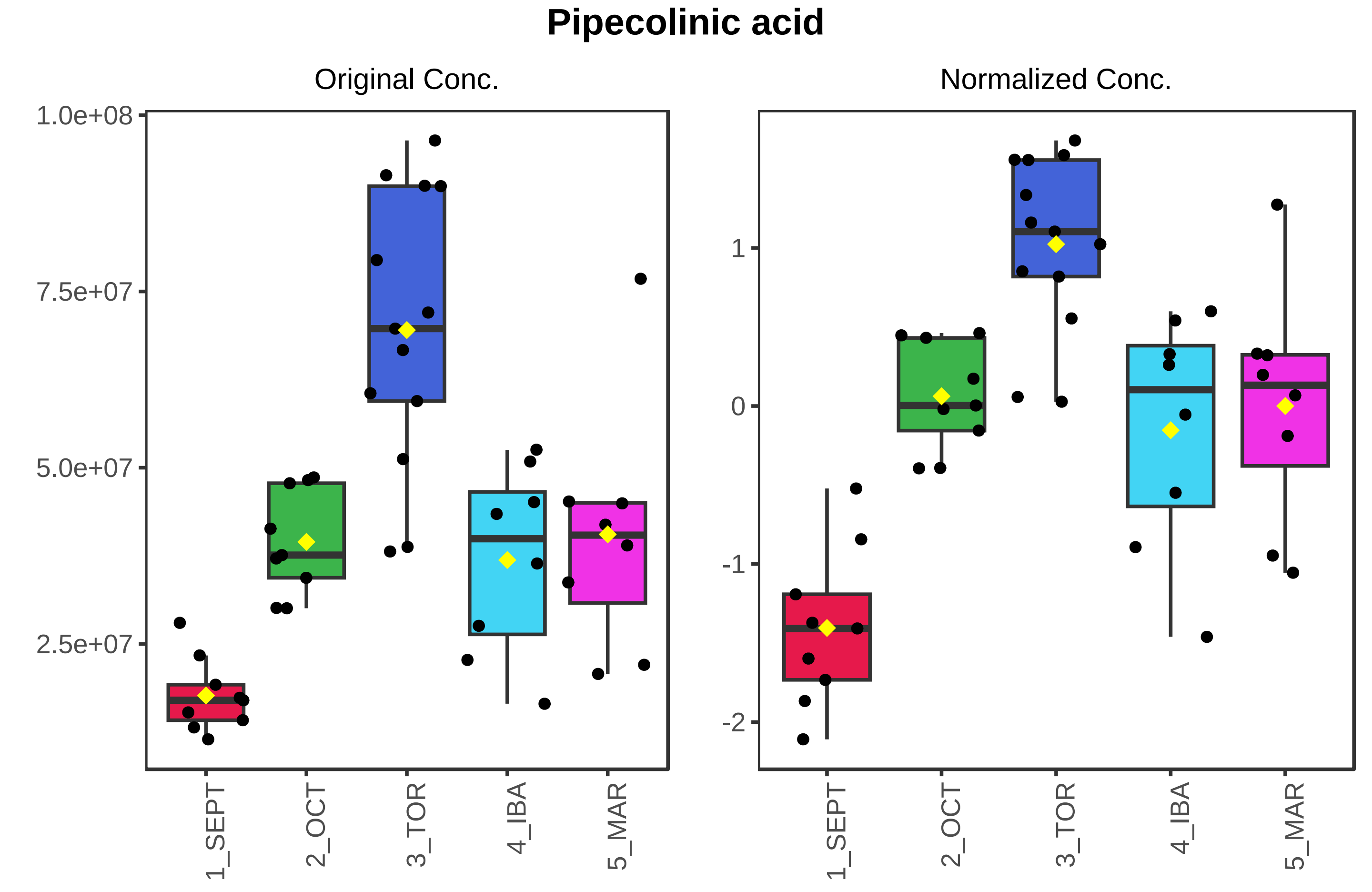

Pipecolinic acid
Original Conc.
Normalized Conc.
1.0e+08
7.5e+07
5.0e+07
2.5e+07
1
0
-1
-2
1_SEPT
2_OCT
3_TOR
4_IBA
5_MAR

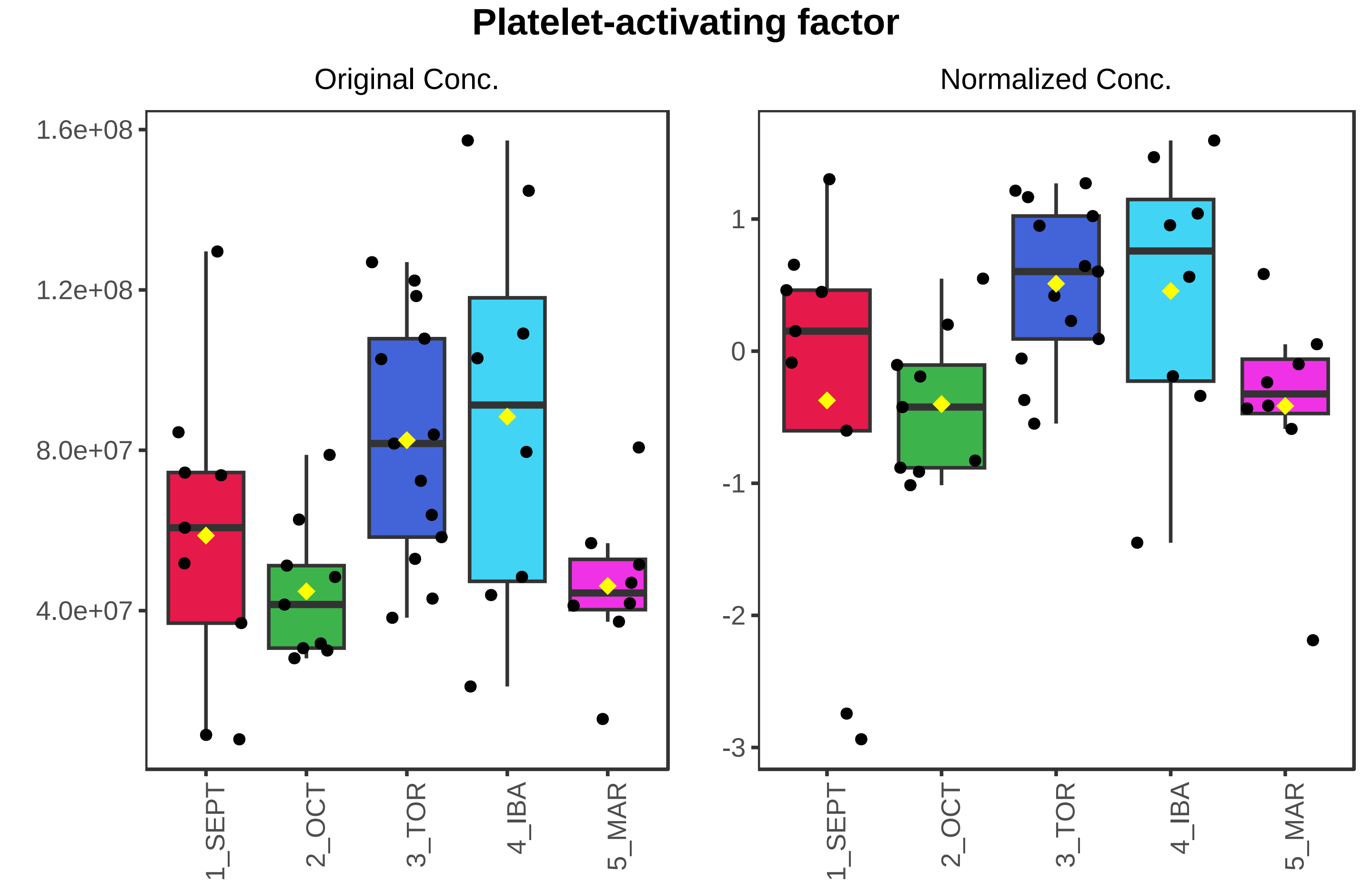

Platelet-activating factor
Original Conc.
Normalized Conc.
1.6e+08
1.2e+08
8.0e+07
4.0e+07
1
0
-1
-2
-3
1_SEPT
2_OCT
3_TOR
4_IBA
5_MAR
1_SEPT
2_OCT
3_TOR
4_IBA
5_MAR

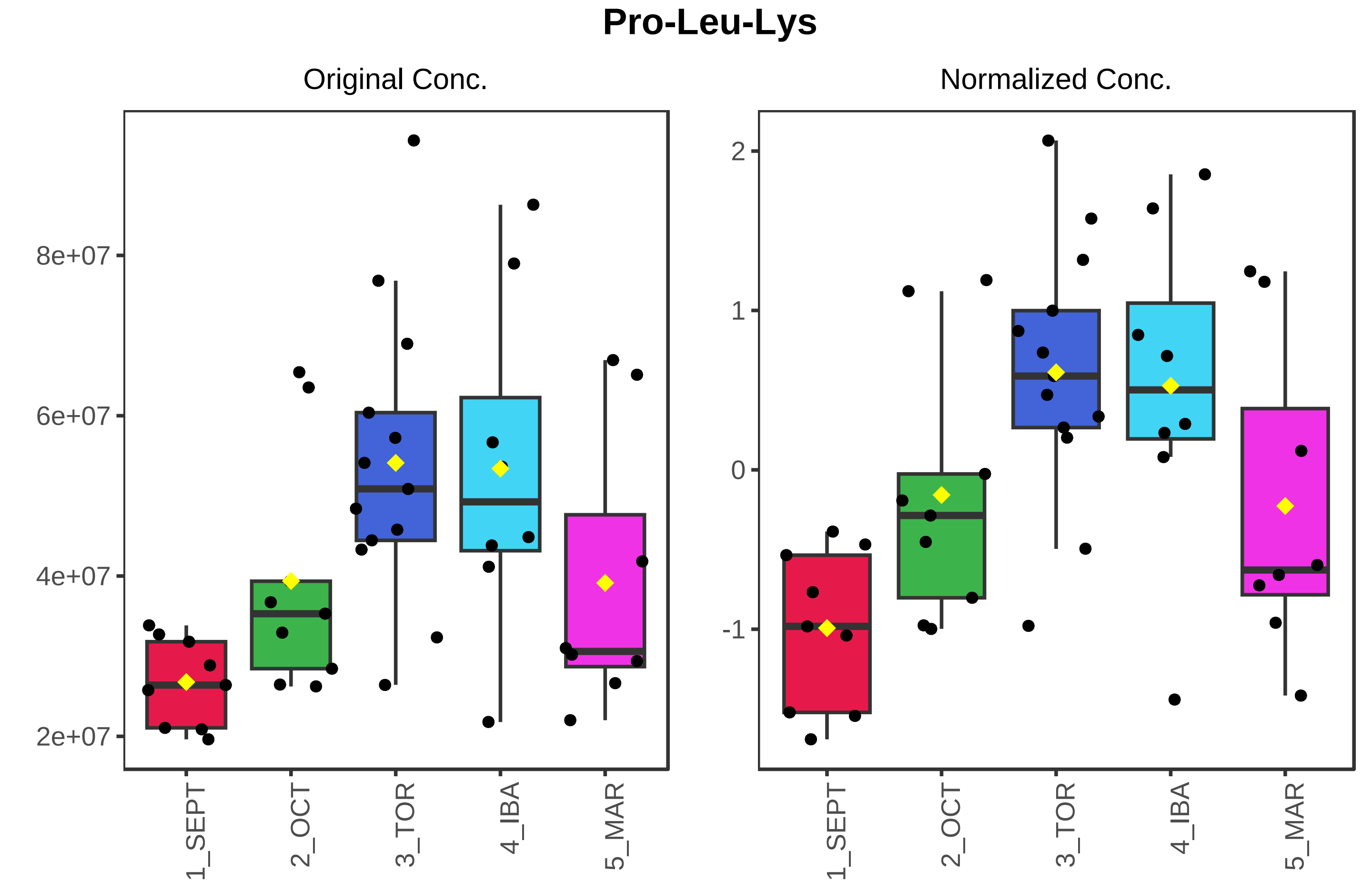
Pro-Leu-Lys
Original Conc.
Normalized Conc.
8e+07
6e+07
4e+07
2e+07
2
1
0
-1
1_SEPT
2_OCT
3_TOR
4_IBA
5_MAR

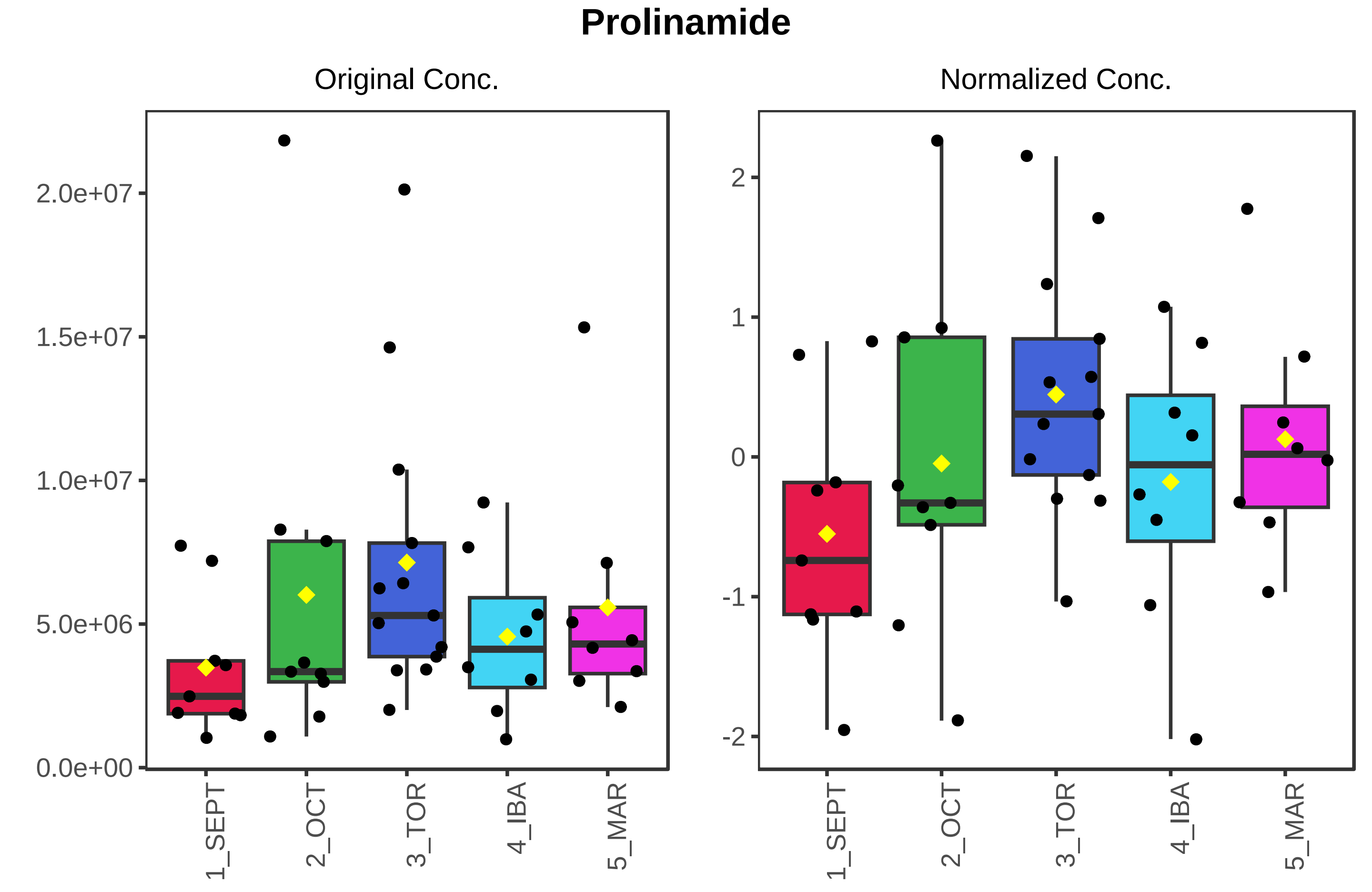
Prolinamide
Original Conc.
Normalized Conc.
2.0e+07
1.5e+07
1.0e+07
5.0e+06
0.0e+00
2
1
0
-1
-2
1_SEPT
2_OCT
3_TOR
4_IBA
5_MAR

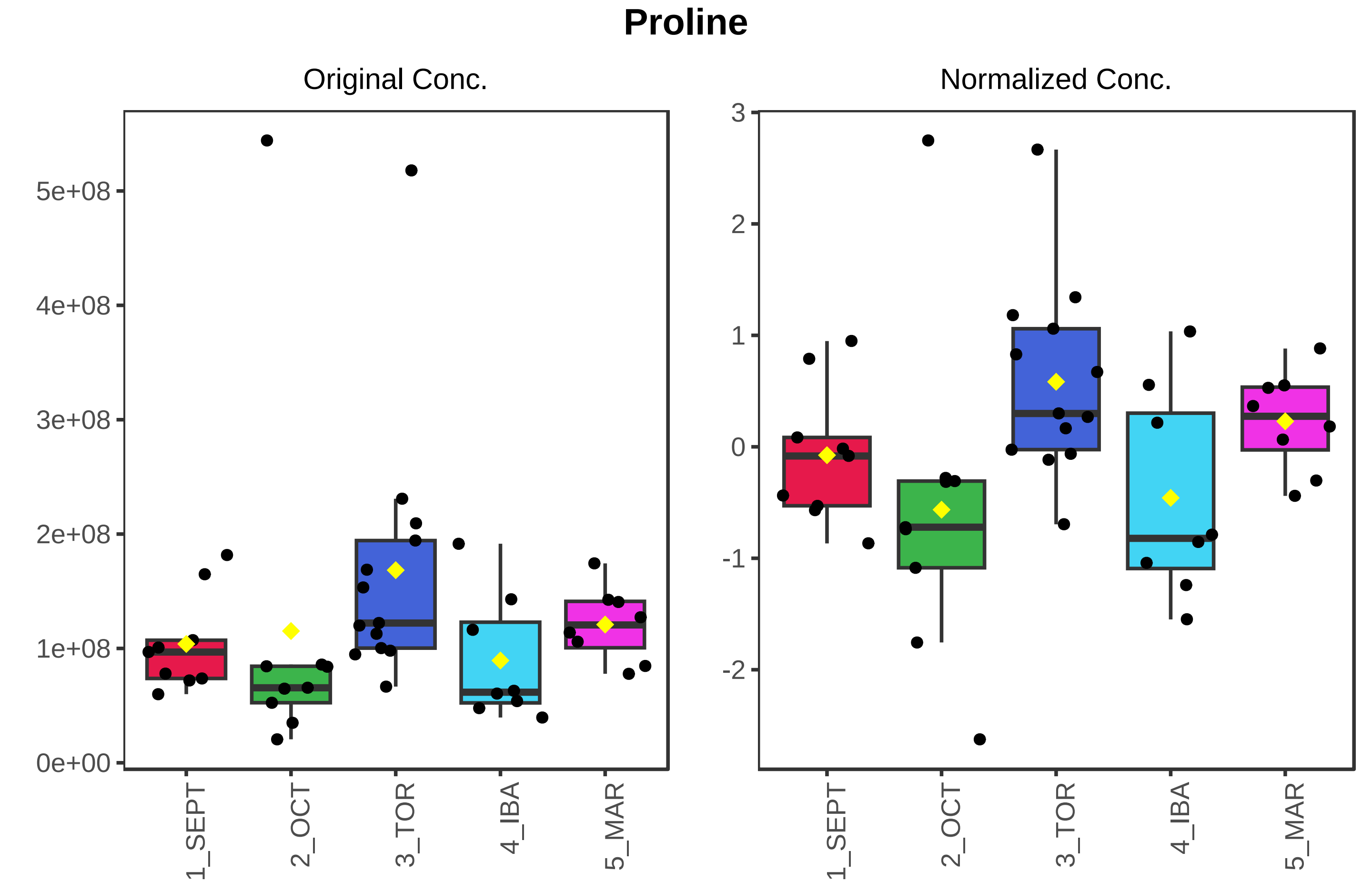

Proline
Original Conc.
Normalized Conc.
5e+08
4e+08
3e+08
2e+08
1e+08
0e+00
3
2
1
0
-1
-2
1_SEPT
2_OCT
3_TOR
4_IBA
5_MAR

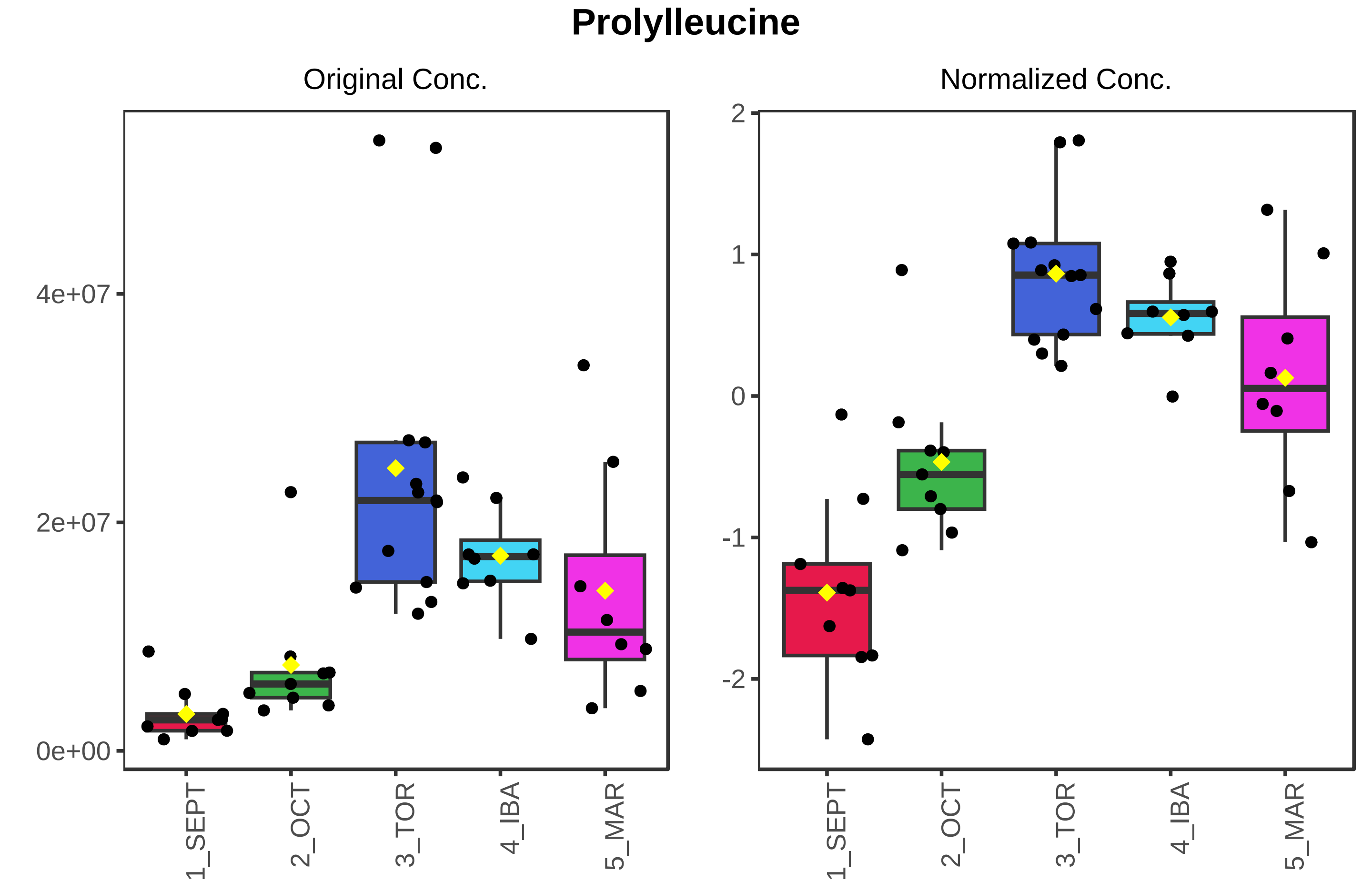

Prolylleucine
Original Conc.
Normalized Conc.
4e+07
2e+07
0e+00
2
1
0
-1
-2
1_SEPT
2_OCT
3_TOR
4_IBA
5_MAR

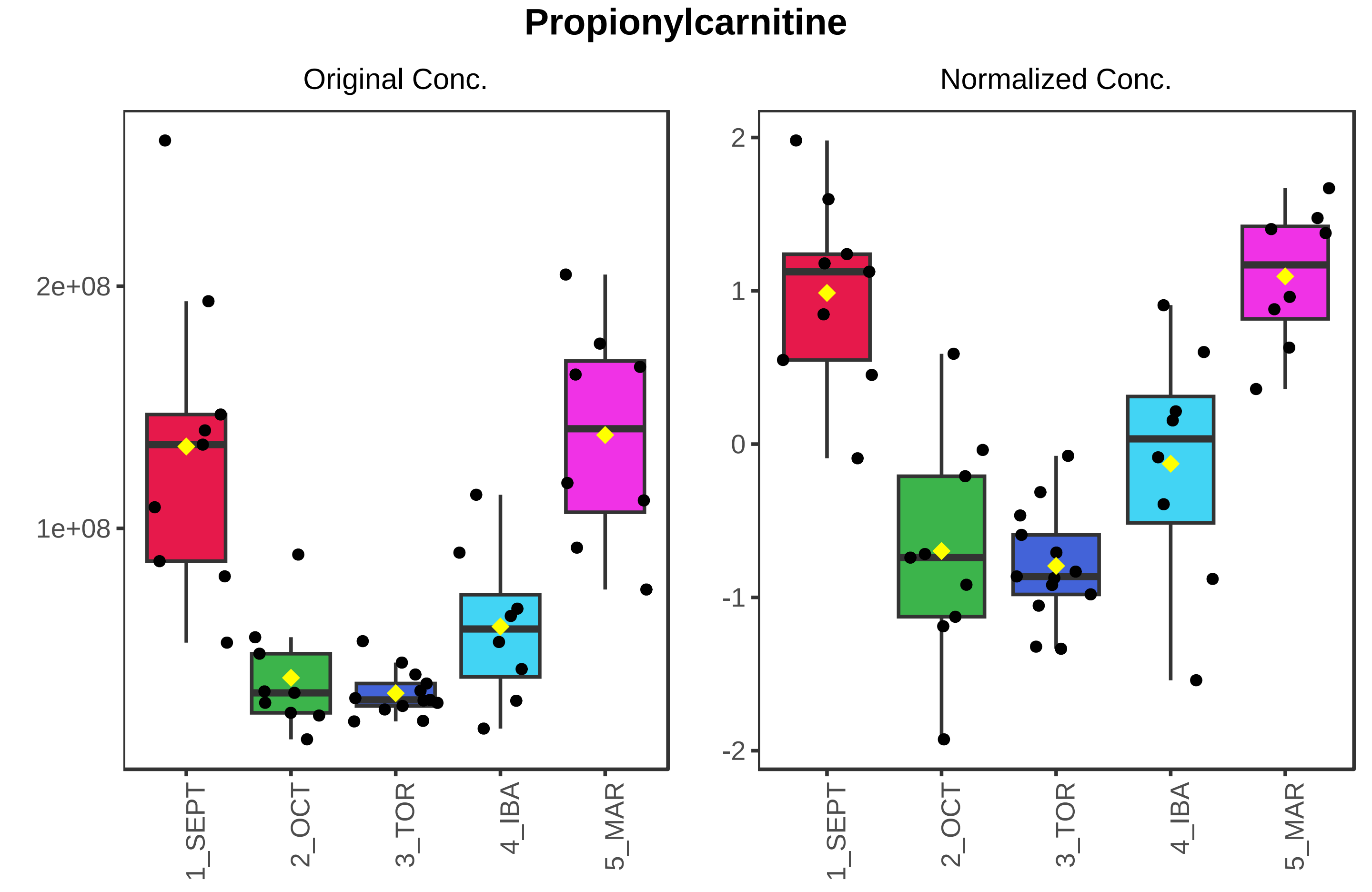

Propionylcarnitine
Original Conc.
Normalized Conc.
2e+08
1e+08
2
1
0
-1
-2
1_SEPT
2_OCT
3_TOR
4_IBA
5_MAR

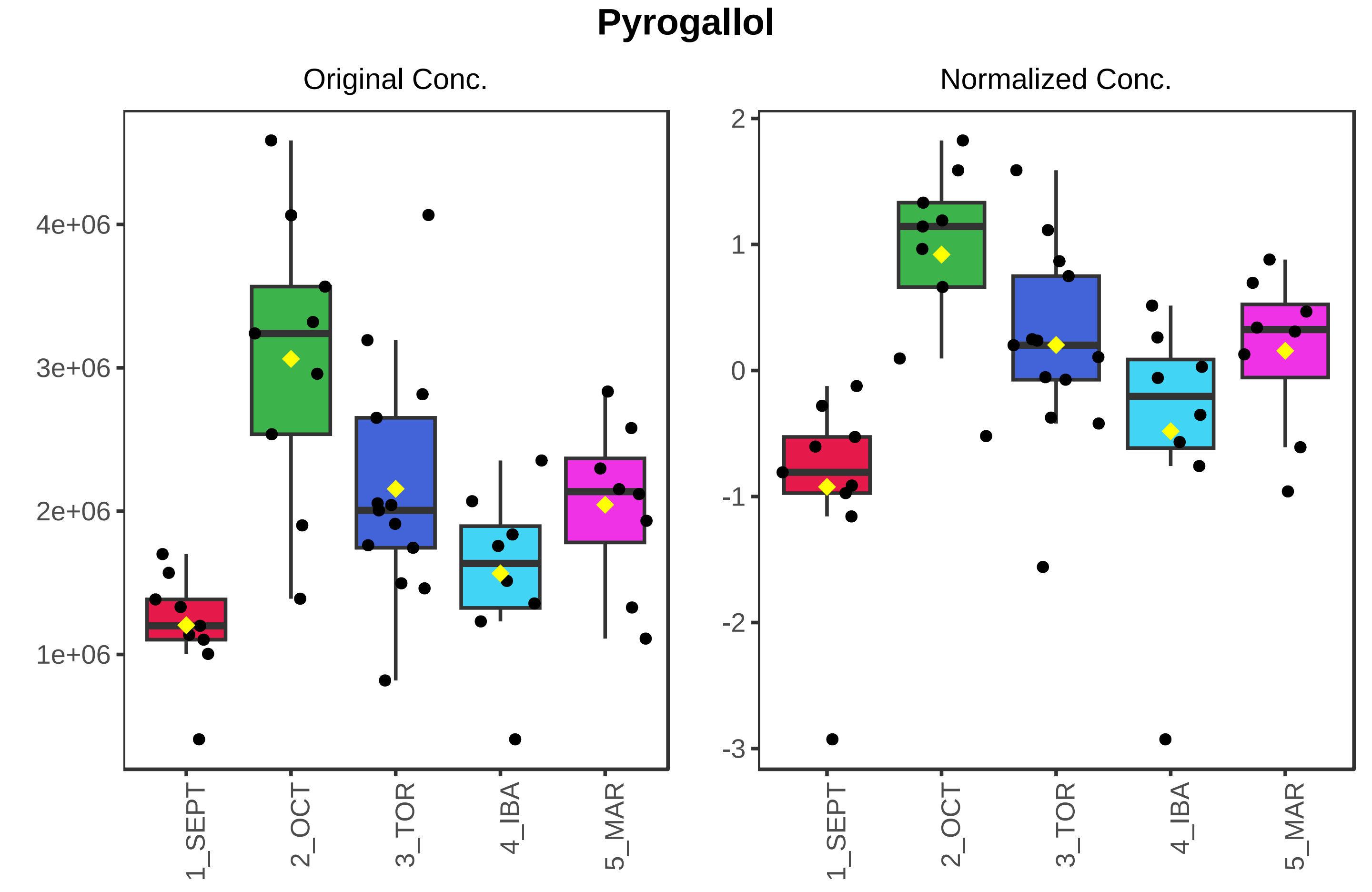

Pyrogallol
Original Conc.
Normalized Conc.
4e+06
3e+06
2e+06
1e+06
2
1
0
-1
-2
-3
1_SEPT
2_OCT
3_TOR
4_IBA
5_MAR

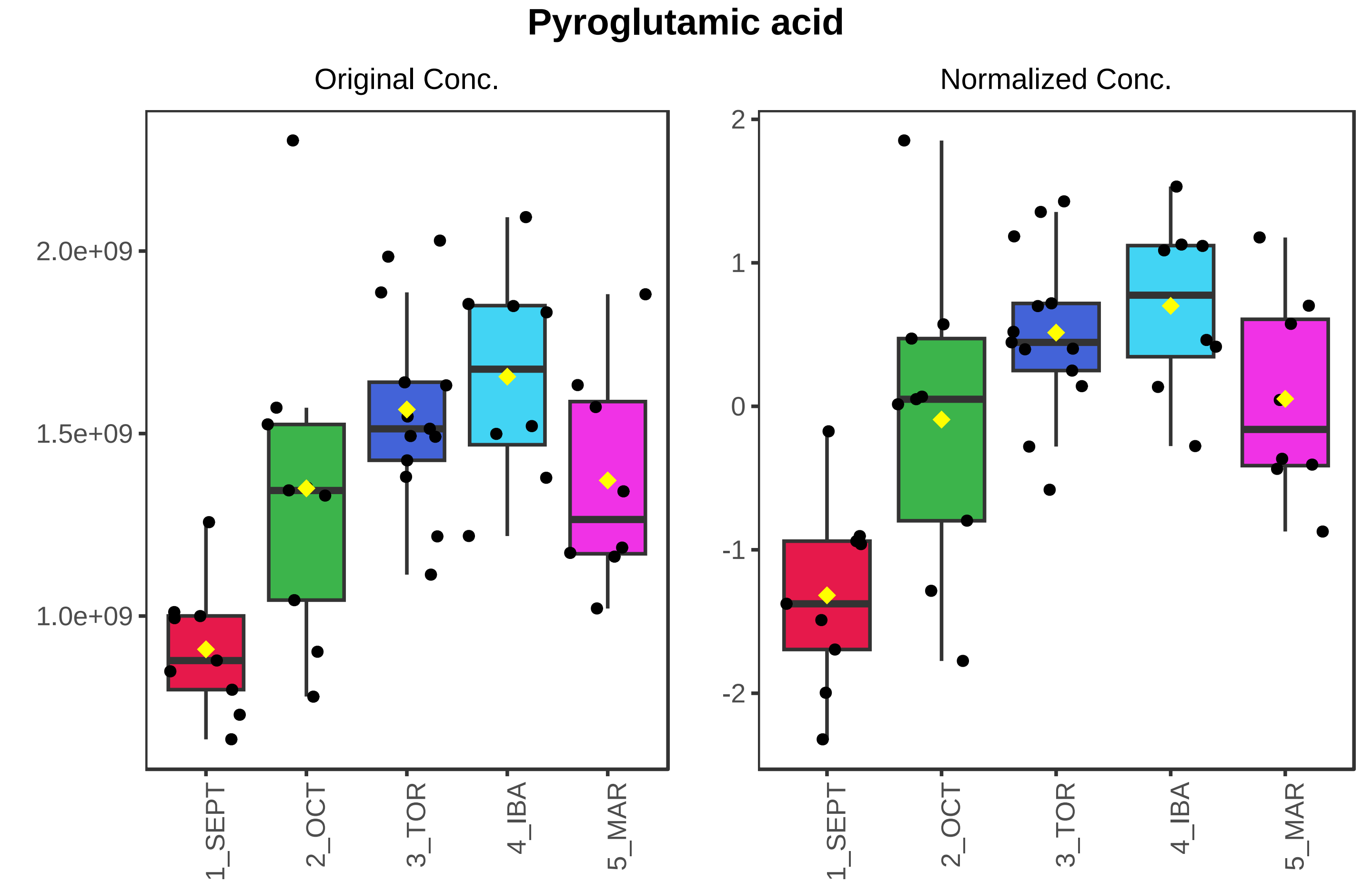
Pyroglutamic acid
Original Conc.
Normalized Conc.
2.0e+09
1.5e+09
1.0e+09
2
1
0
-1
-2
1_SEPT
2_OCT
3_TOR
4_IBA
5_MAR
1_SEPT
2_OCT
3_TOR
4_IBA
5_MAR

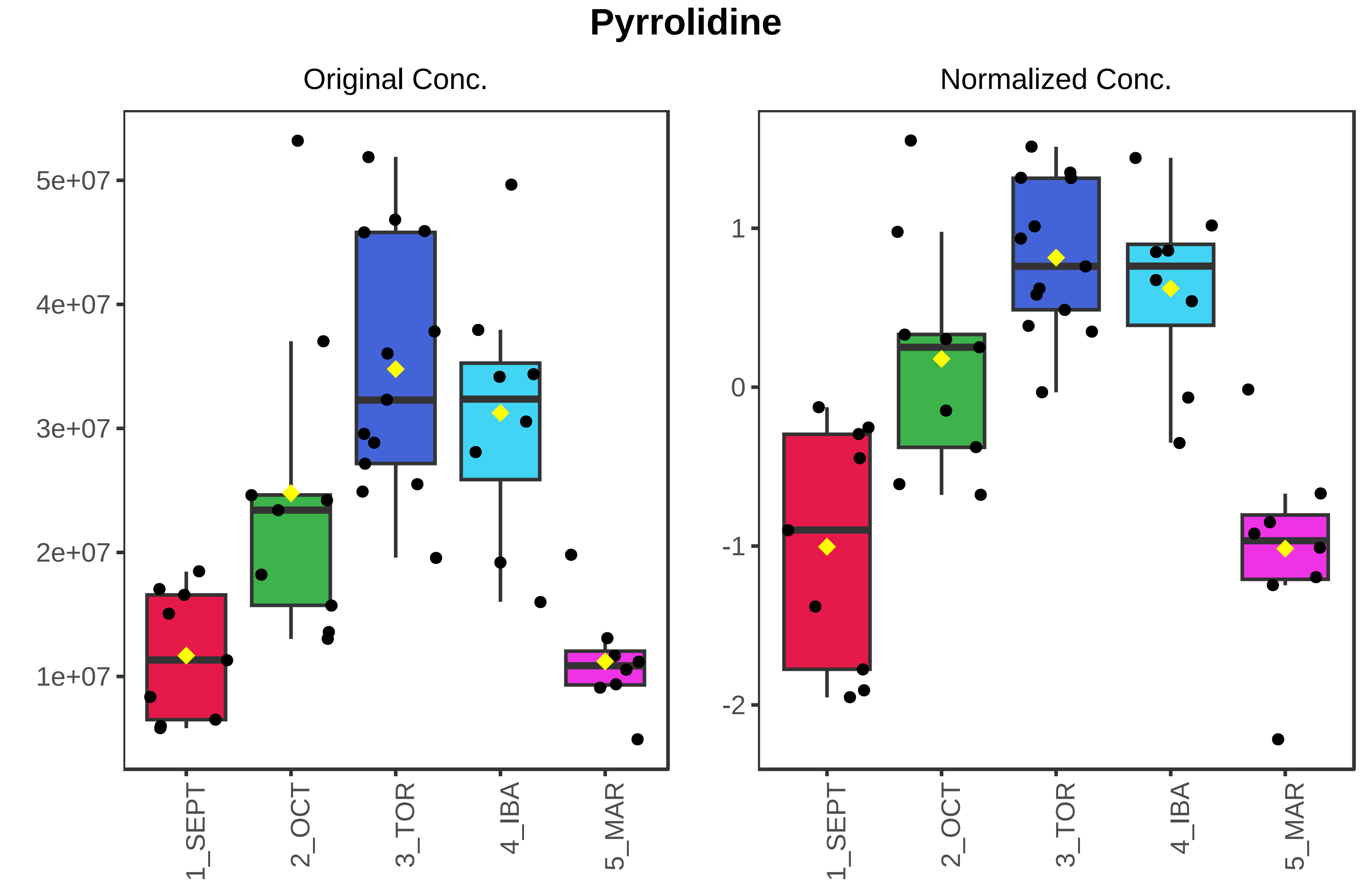
Pyrrolidine
Original Conc.
Normalized Conc.
5e+07
4e+07
3e+07
2e+07
1e+07
1
0
-1
-2
1_SEPT
2_OCT
3_TOR
4_IBA
5_MAR
1_SEPT
2_OCT
3_TOR
4_IBA
5_MAR

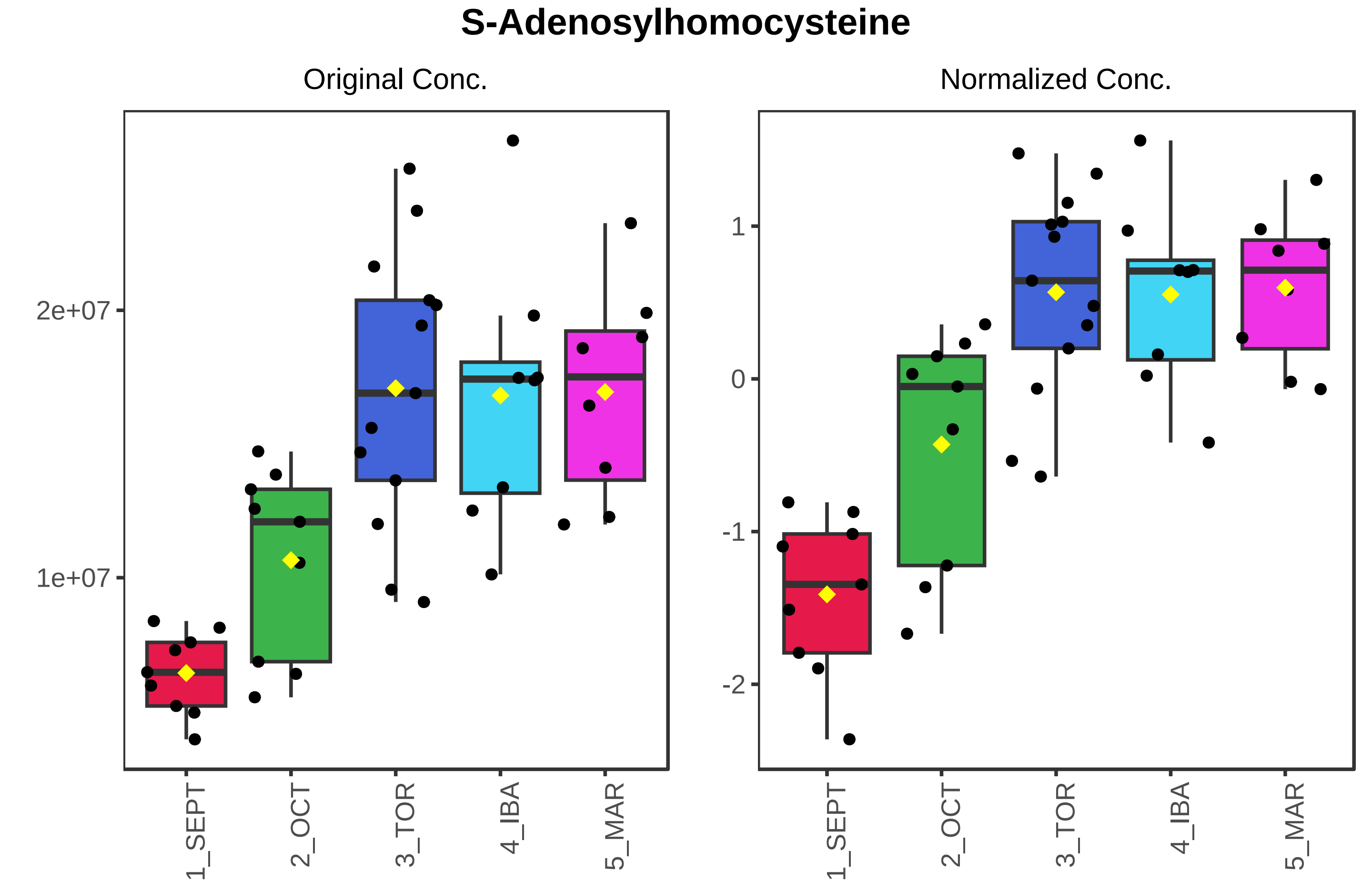

S-Adenosylhomocysteine
Original Conc.
Normalized Conc.
2e+07
1e+07
1
0
-1
-2
1_SEPT
2_OCT
3_TOR
4_IBA
5_MAR

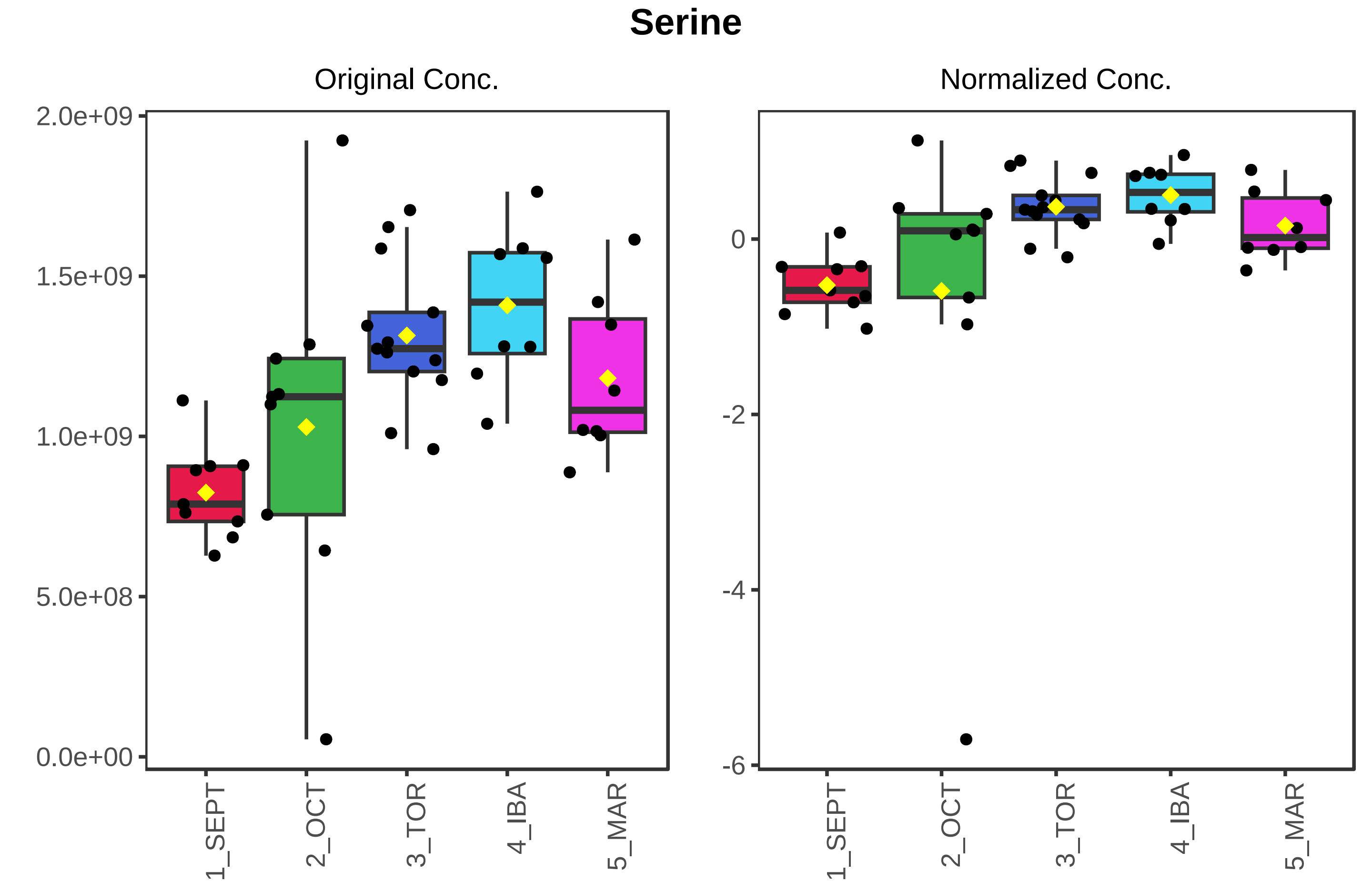
Serine
Original Conc.
Normalized Conc.
2.0e+09
1.5e+09
1.0e+09
5.0e+08
0.0e+00
0
-2
-4
-6
1_SEPT
2_OCT
3_TOR
4_IBA
5_MAR
1_SEPT
2_OCT
3_TOR
4_IBA
5_MAR

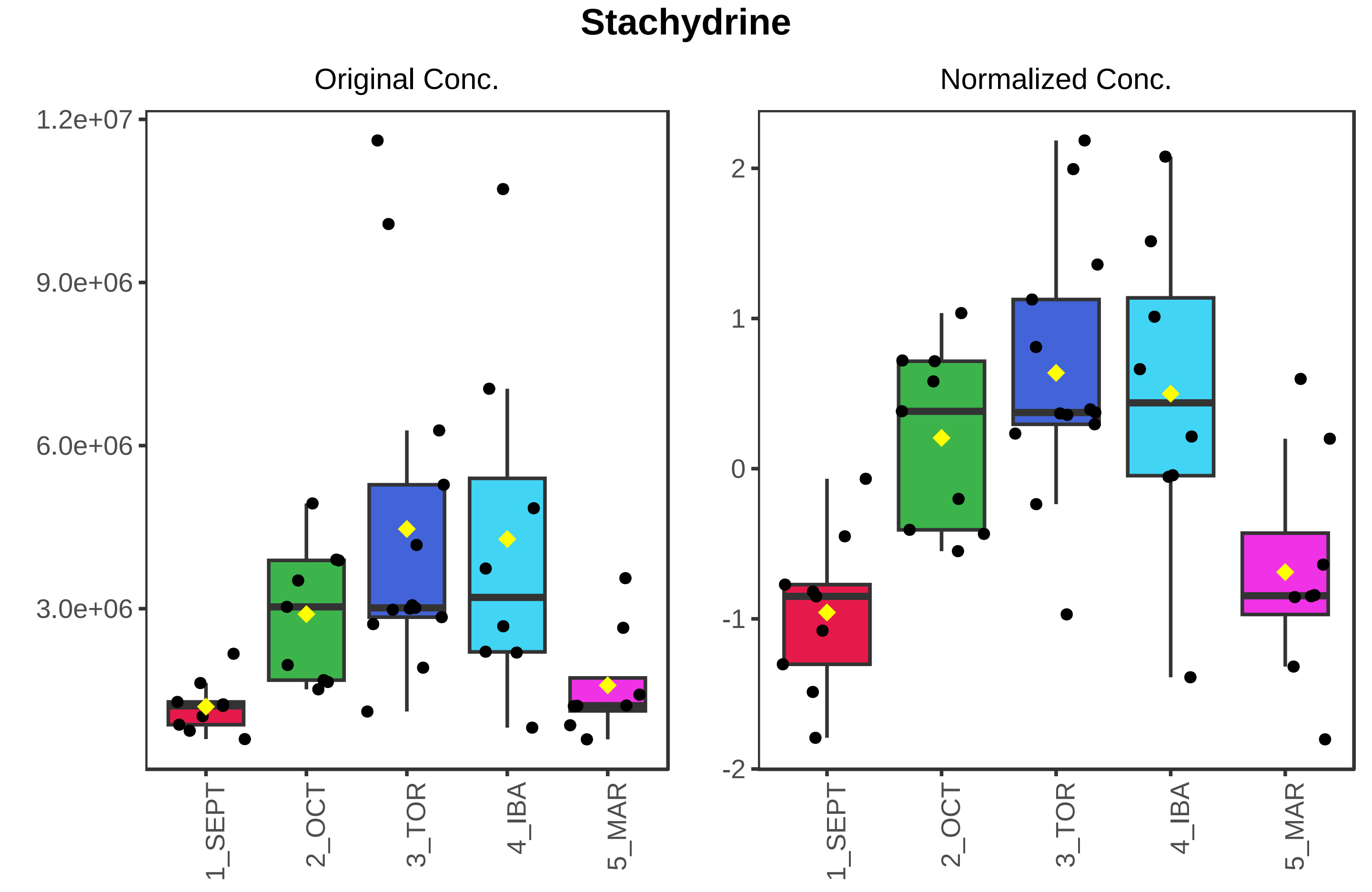

Stachydrine
Original Conc.
Normalized Conc.
1.2e+07
9.0e+06
6.0e+06
3.0e+06
2
1
0
-1
-2
1_SEPT
2_OCT
3_TOR
4_IBA
5_MAR

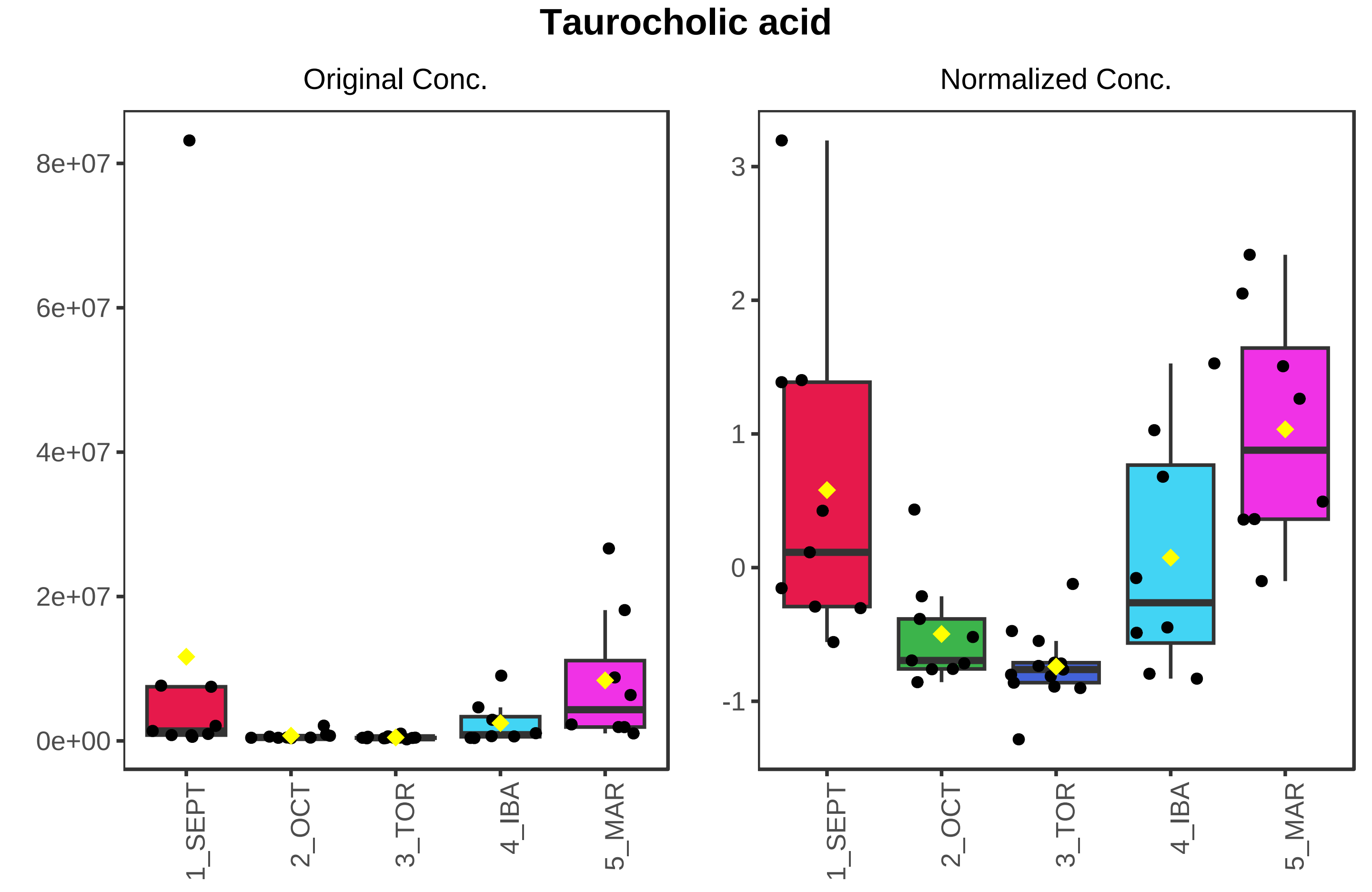

Taurocholic acid
Original Conc.
Normalized Conc.
8e+07
6e+07
4e+07
2e+07
0e+00
3
2
1
0
-1
1_SEPT
2_OCT
3_TOR
4_IBA
5_MAR

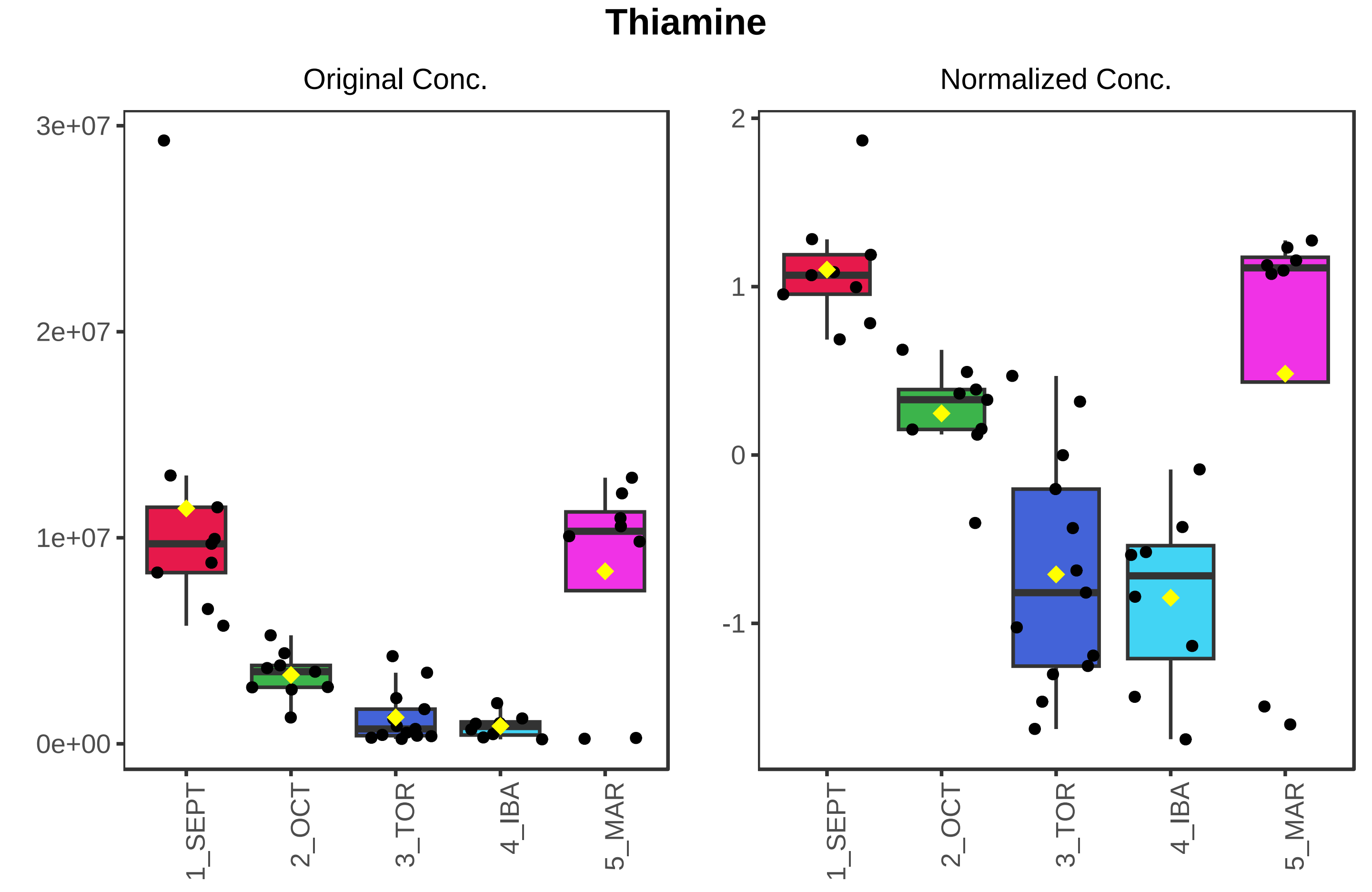
Thiamine
Original Conc.
Normalized Conc.
3e+07
2e+07
1e+07
0e+00
2
1
0
-1
1_SEPT
2_OCT
3_TOR
4_IBA
5_MAR

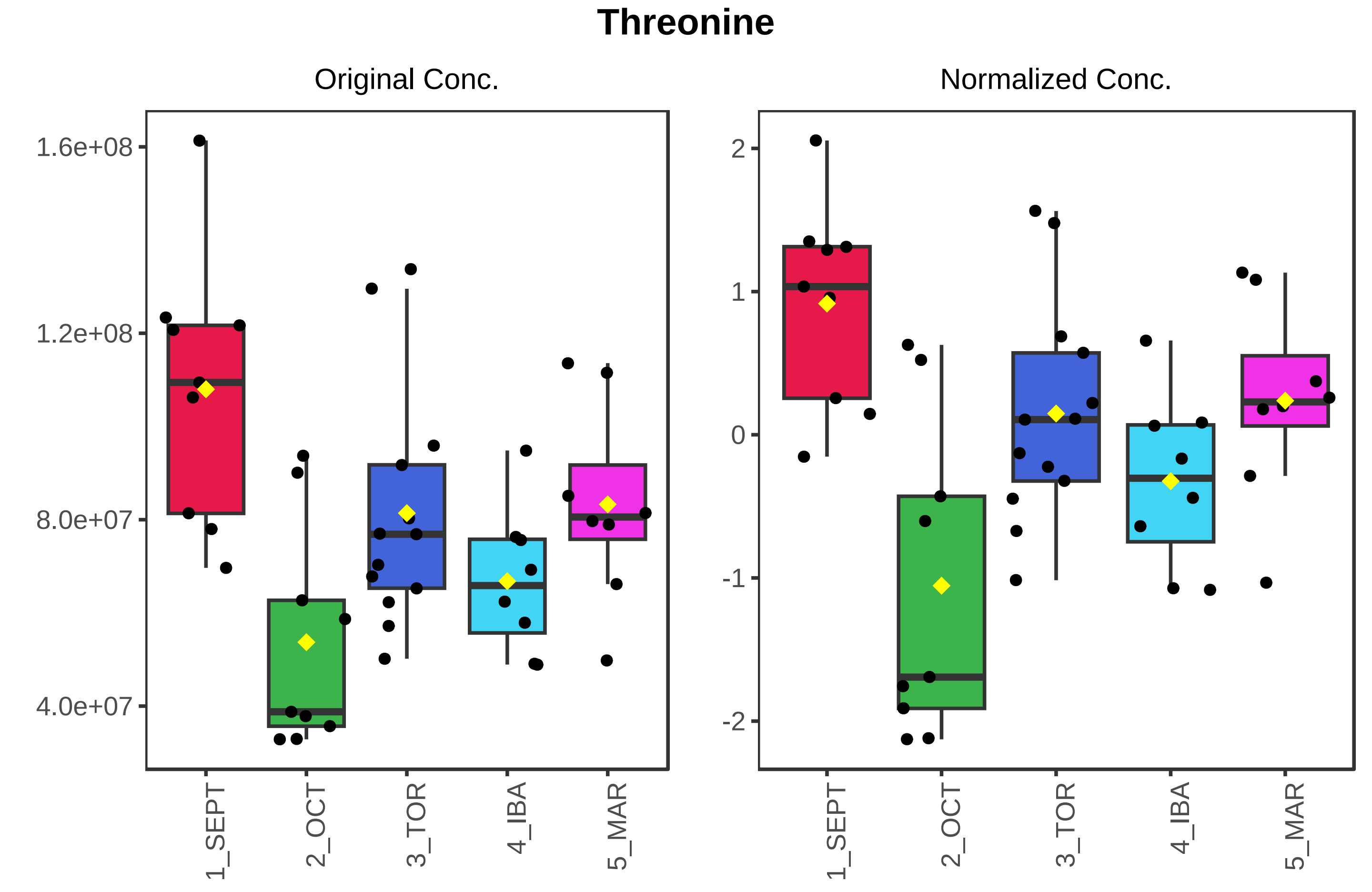

Threonine
Original Conc.
Normalized Conc.
1.6e+08
1.2e+08
8.0e+07
4.0e+07
2
1
0
-1
-2
1_SEPT
2_OCT
3_TOR
4_IBA
5_MAR

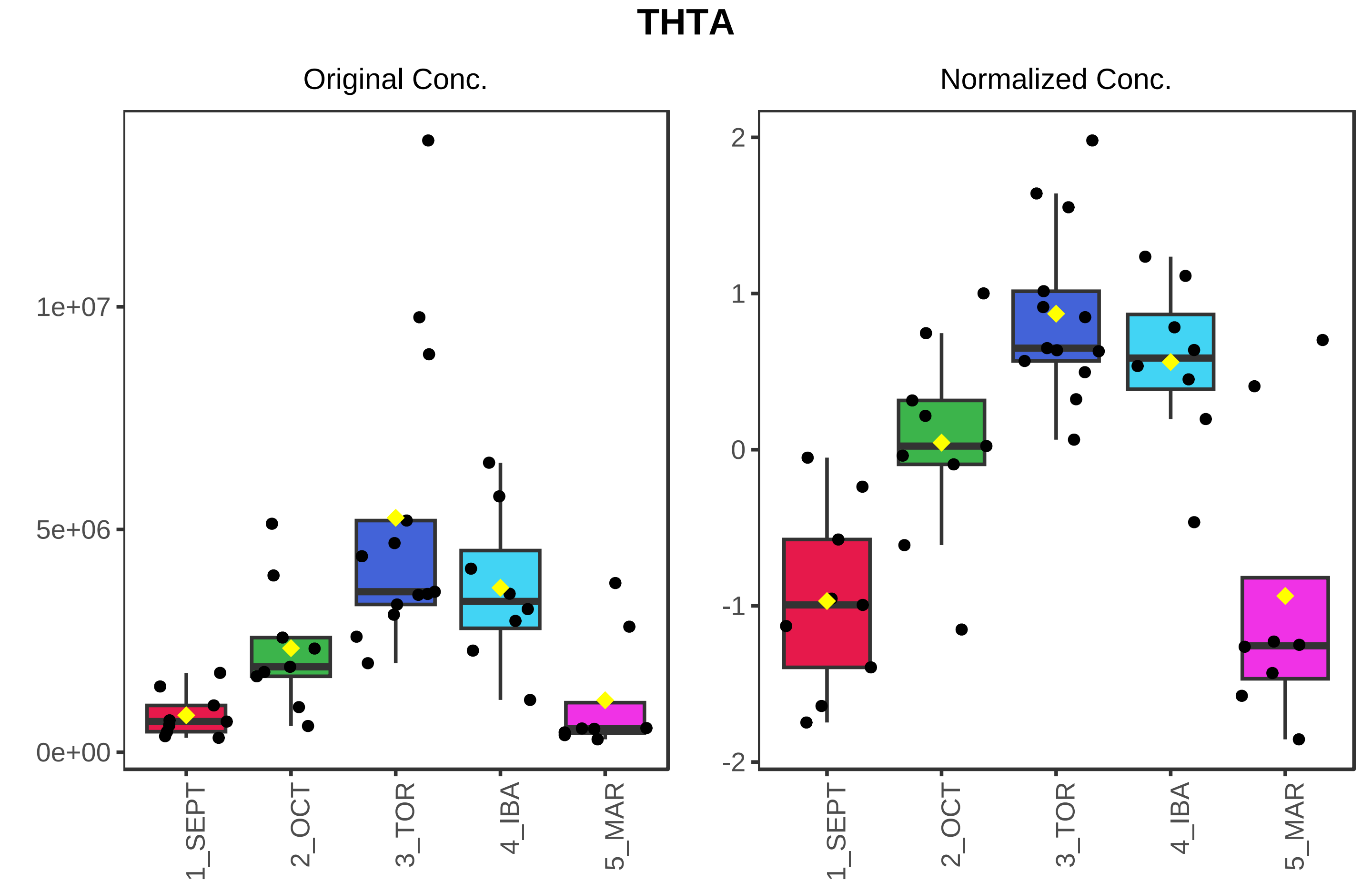
THTA
Original Conc.
Normalized Conc.
1e+07
5e+06
0e+00
2
1
0
-1
-2
1_SEPT
2_OCT
3_TOR
4_IBA
5_MAR
1_SEPT
2_OCT
3_TOR
4_IBA
5_MAR

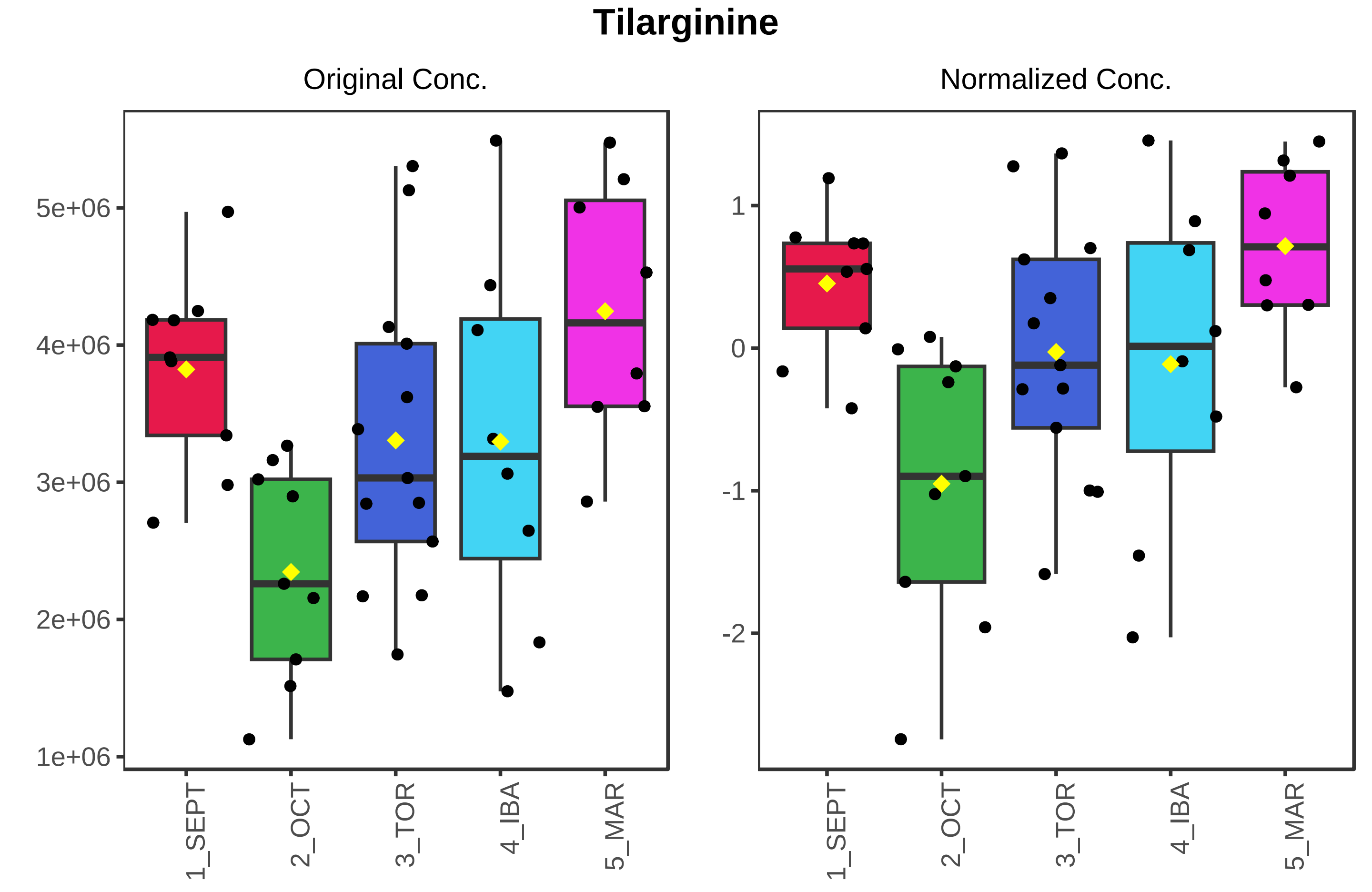
Tilarginine
Original Conc.
Normalized Conc.
5e+06
4e+06
3e+06
2e+06
1e+06
1
0
-1
-2
1_SEPT
2_OCT
3_TOR
4_IBA
5_MAR
1_SEPT
2_OCT
3_TOR
4_IBA
5_MAR

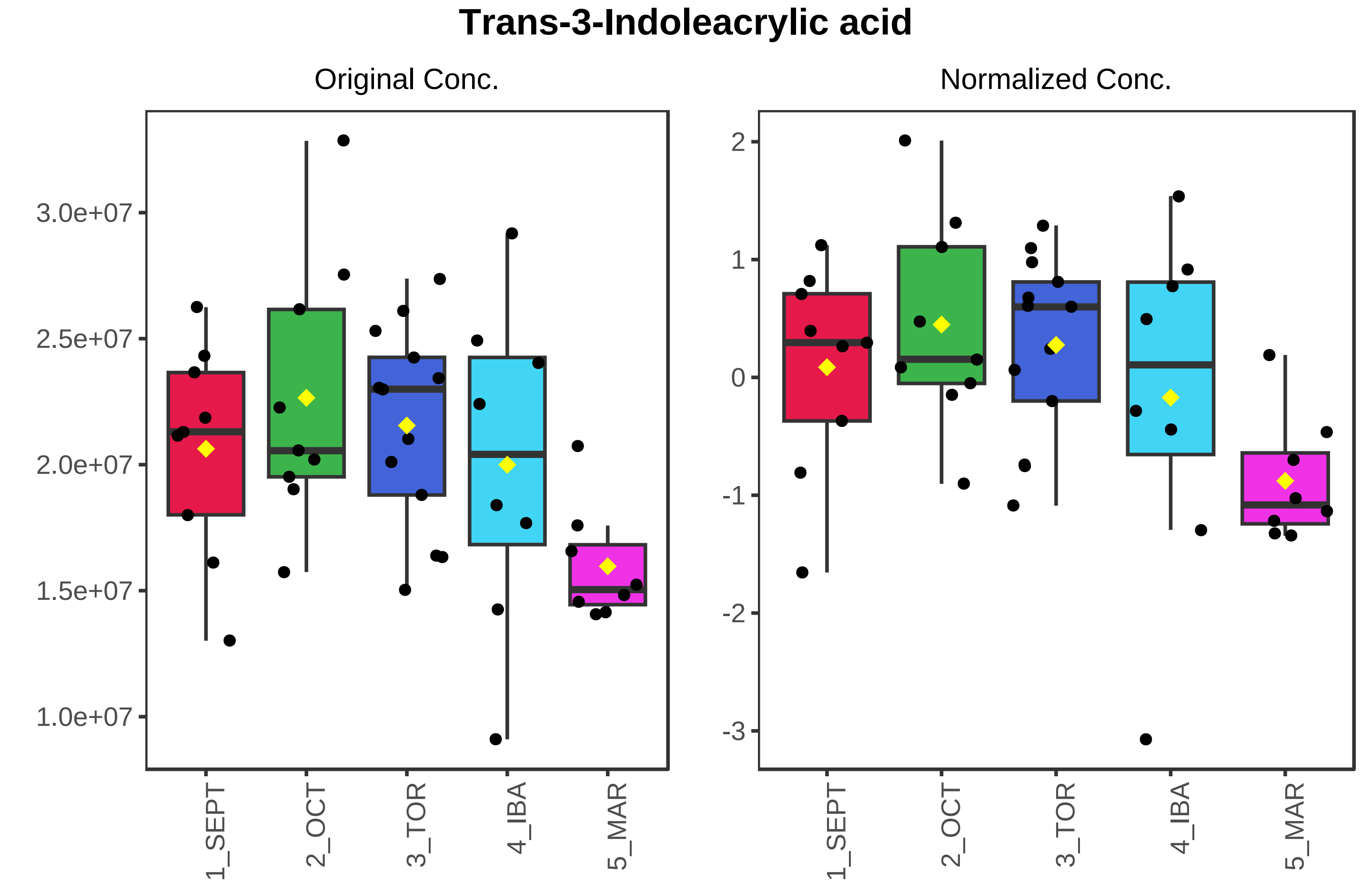
Trans-3-Indoleacrylic acid
Original Conc.
Normalized Conc.
3.0e+07
2.5e+07
2.0e+07
1.5e+07
1.0e+07
2
1
0
-1
-2
-3
1_SEPT
2_OCT
3_TOR
4_IBA
5_MAR

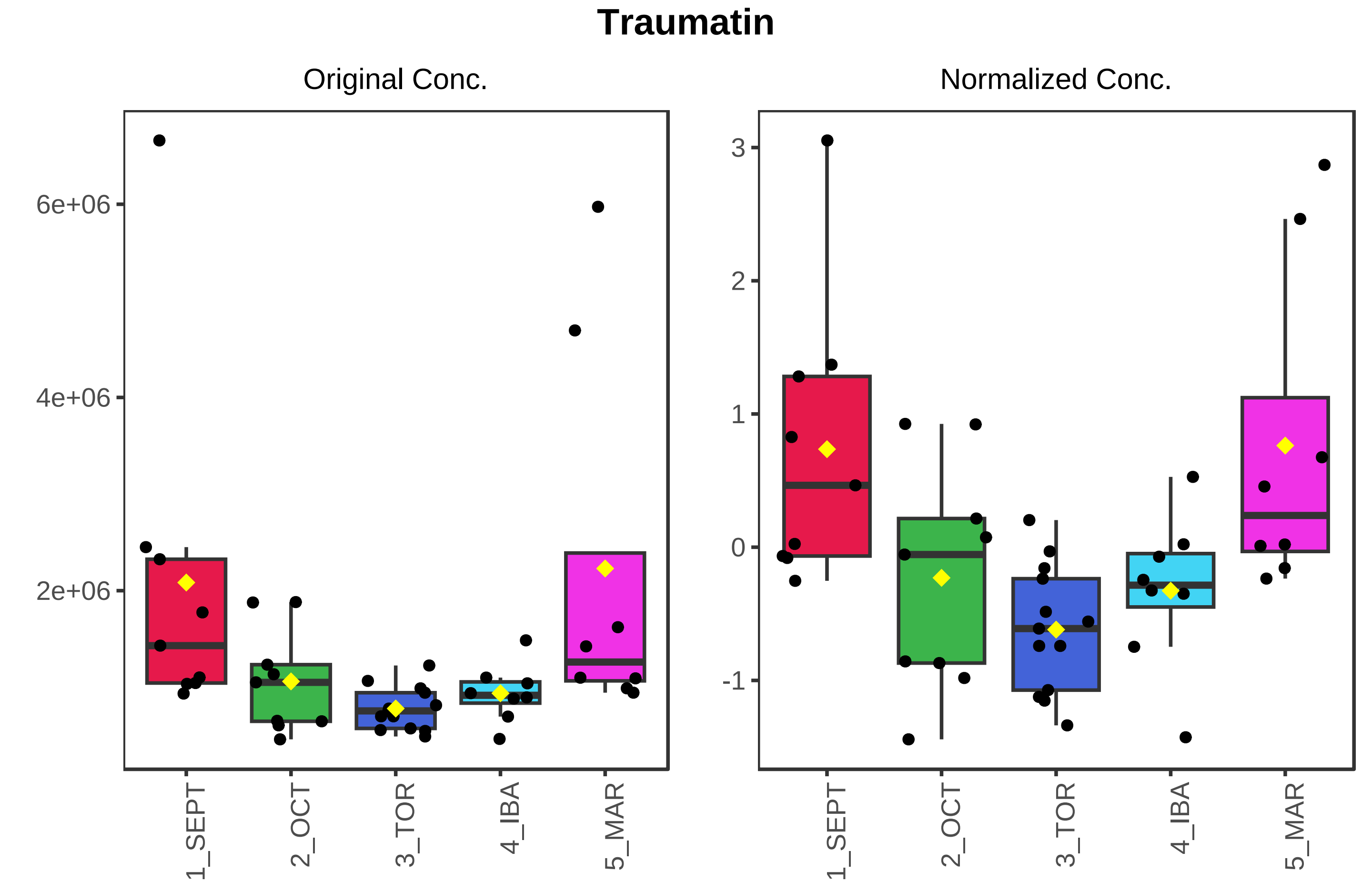

Traumatin
Original Conc.
Normalized Conc.
6e+06
4e+06
2e+06
3
2
1
0
-1
1_SEPT
2_OCT
3_TOR
4_IBA
5_MAR

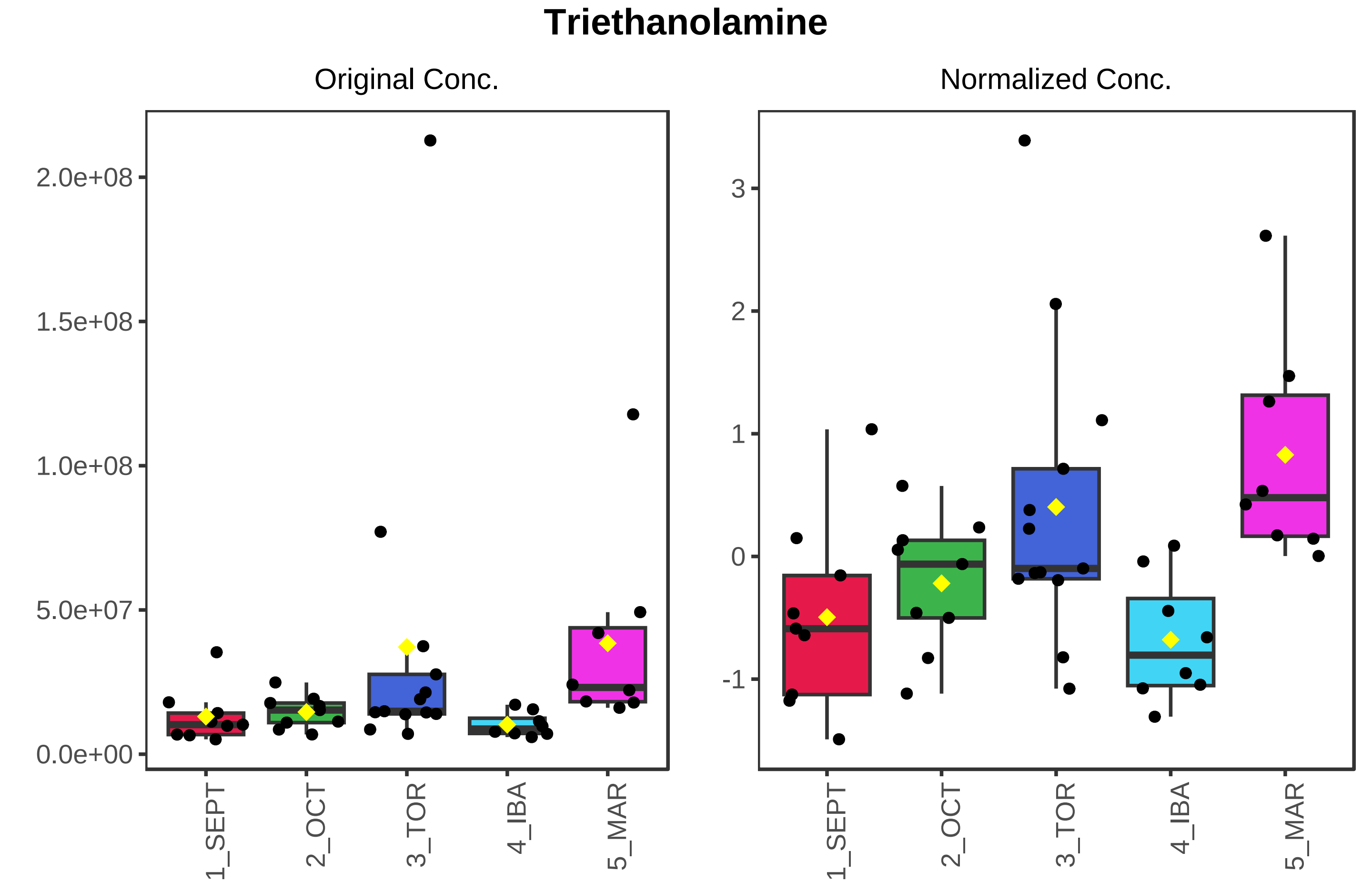

Triethanolamine
Original Conc.
Normalized Conc.
2.0e+08
1.5e+08
1.0e+08
5.0e+07
0.0e+00
3
2
1
0
-1
1_SEPT
2_OCT
3_TOR
4_IBA
5_MAR

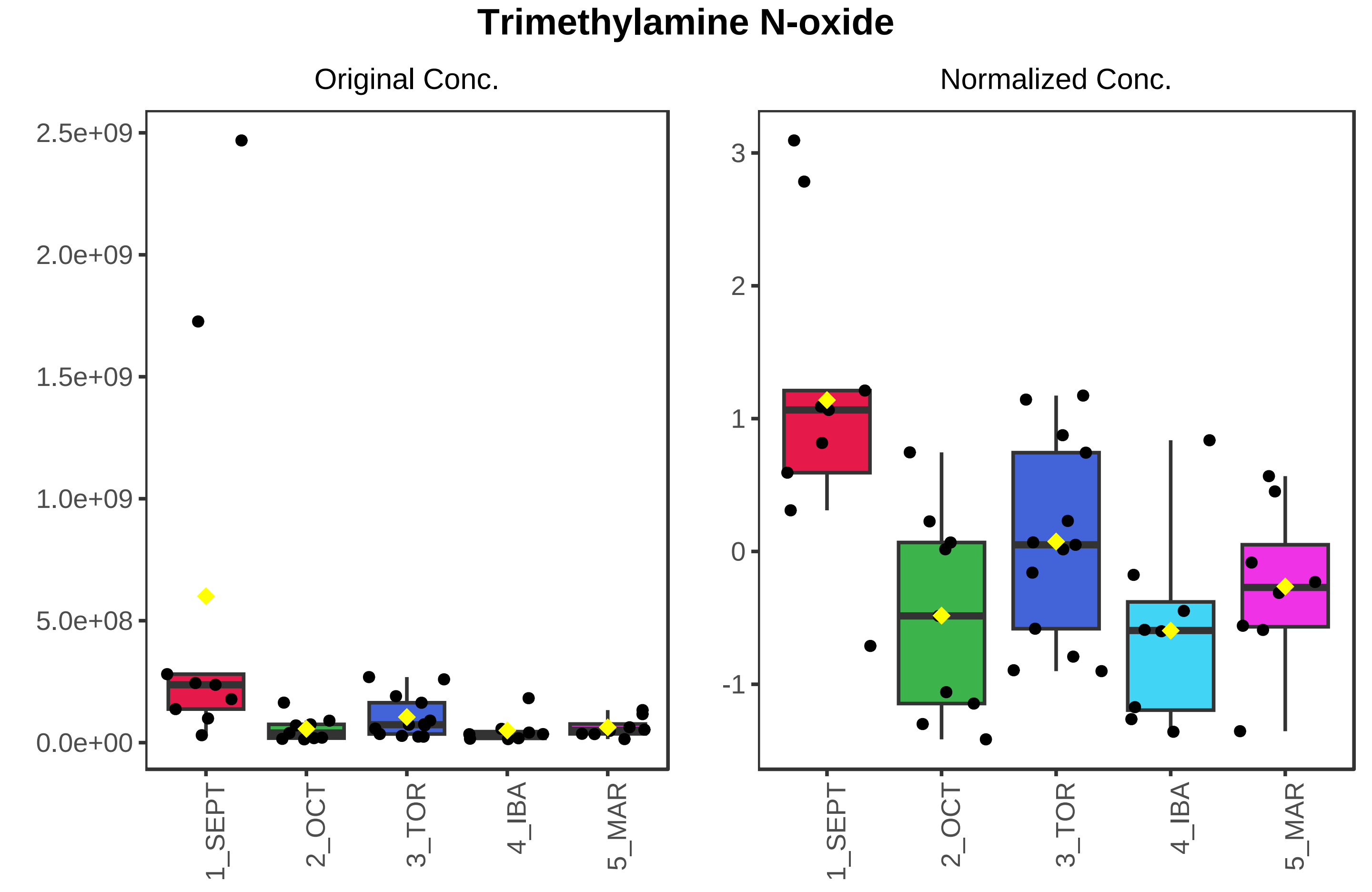

Trimethylamine N-oxide
Original Conc.
Normalized Conc.
2.5e+09
2.0e+09
1.5e+09
1.0e+09
5.0e+08
0.0e+00
3
2
1
0
-1
1_SEPT
2_OCT
3_TOR
4_IBA
5_MAR

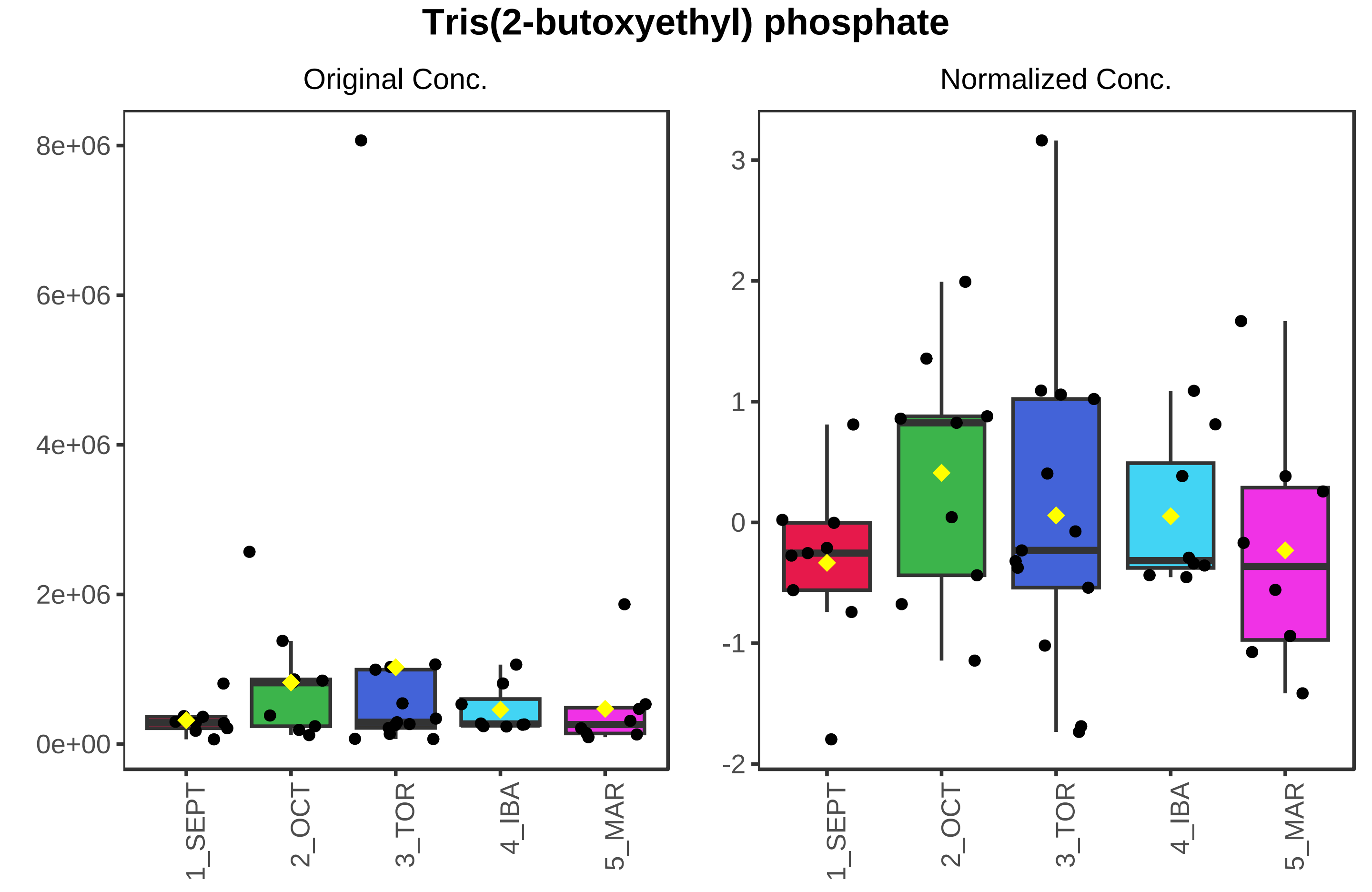

Tris(2-butoxyethyl) phosphate
Original Conc.
Normalized Conc.
8e+06
6e+06
4e+06
2e+06
0e+00
3
2
1
0
-1
-2
1_SEPT
2_OCT
3_TOR
4_IBA
5_MAR

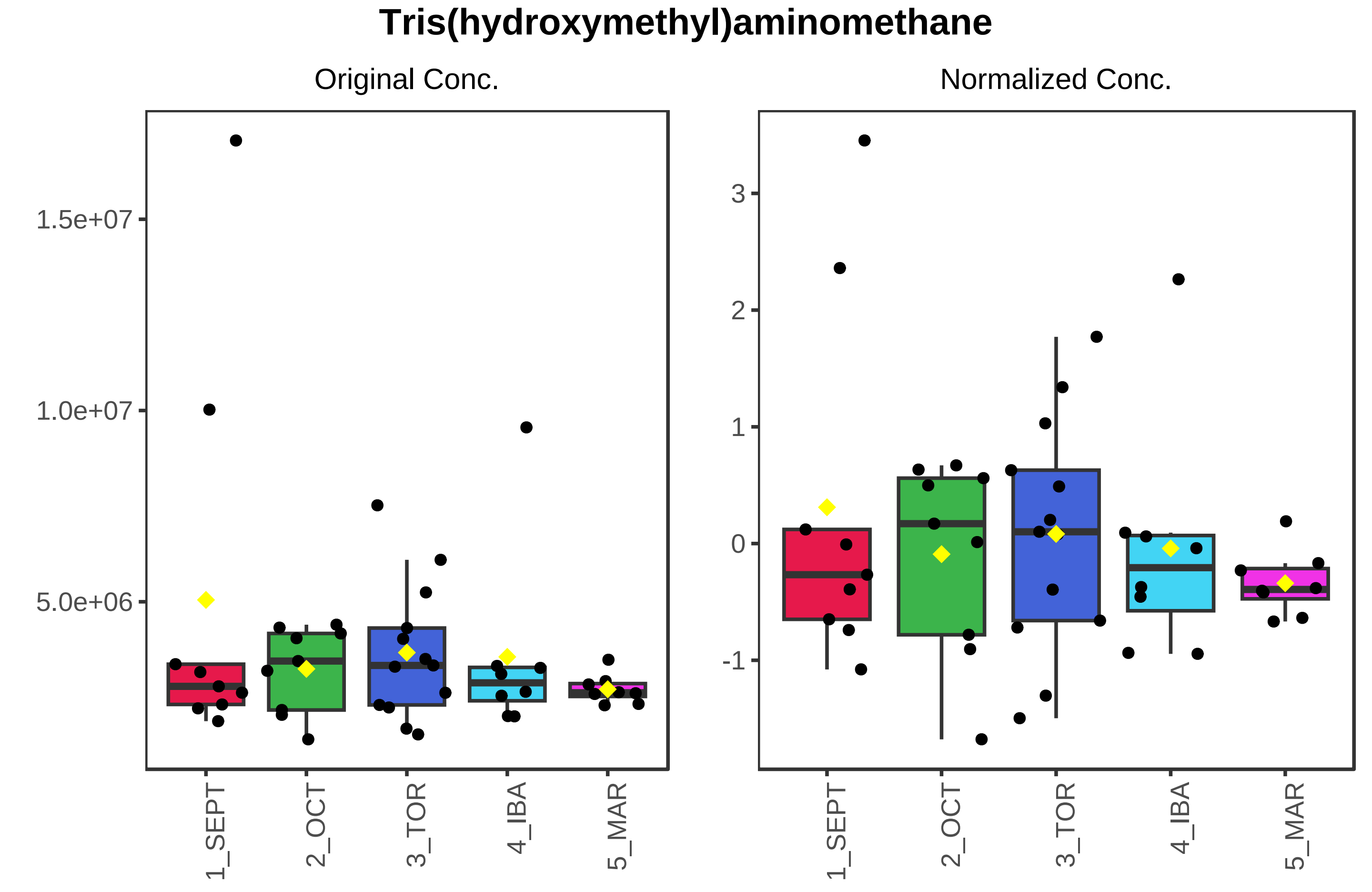
Tris(hydroxymethyl)aminomethane
Original Conc.
Normalized Conc.
1.5e+07
1.0e+07
5.0e+06
3
2
1
0
-1
1_SEPT
2_OCT
3_TOR
4_IBA
5_MAR

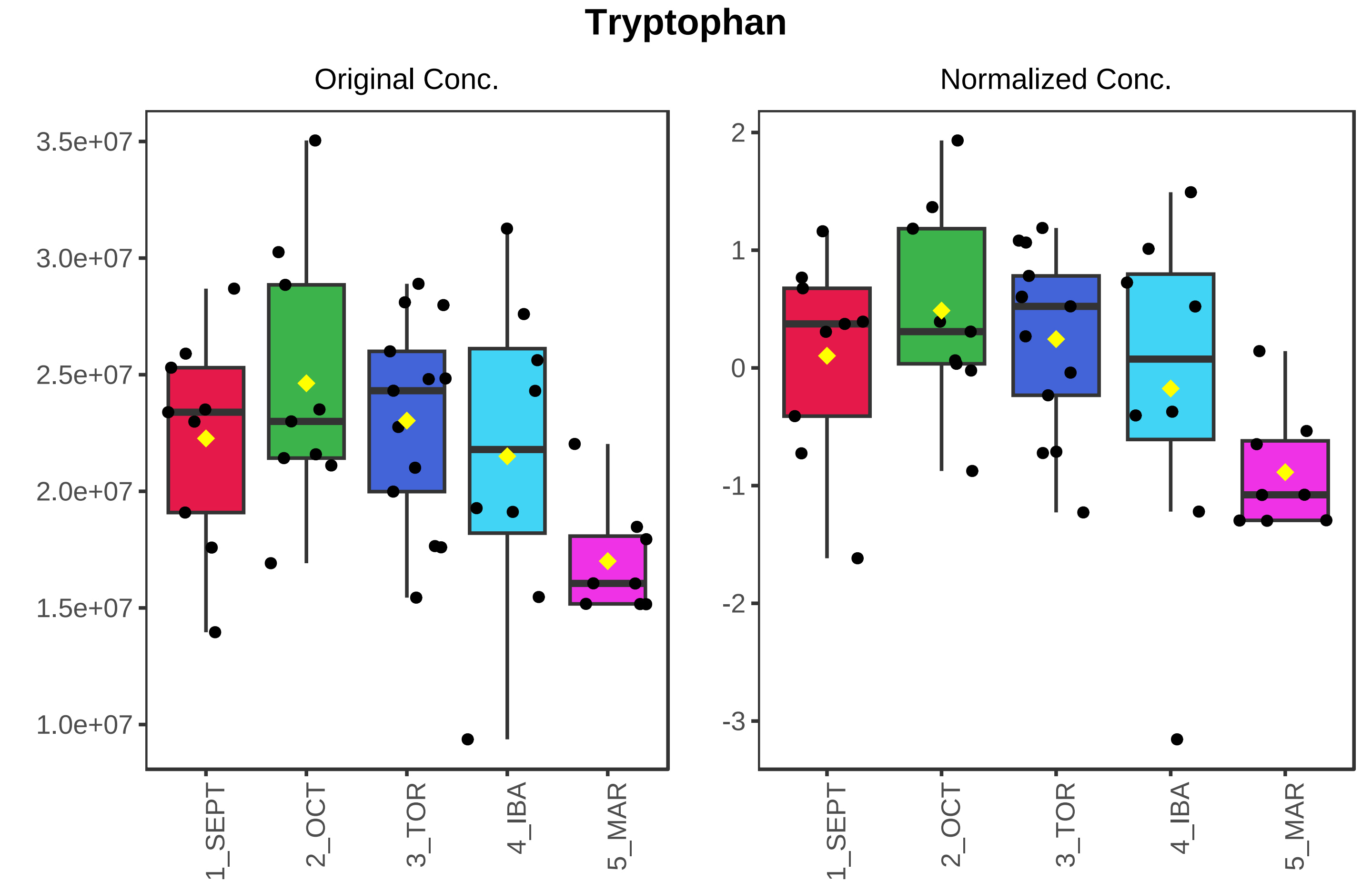

Tryptophan
Original Conc.
Normalized Conc.
3.5e+07
3.0e+07
2.5e+07
2.0e+07
1.5e+07
1.0e+07
2
1
0
-1
-2
-3
1_SEPT
2_OCT
3_TOR
4_IBA
5_MAR

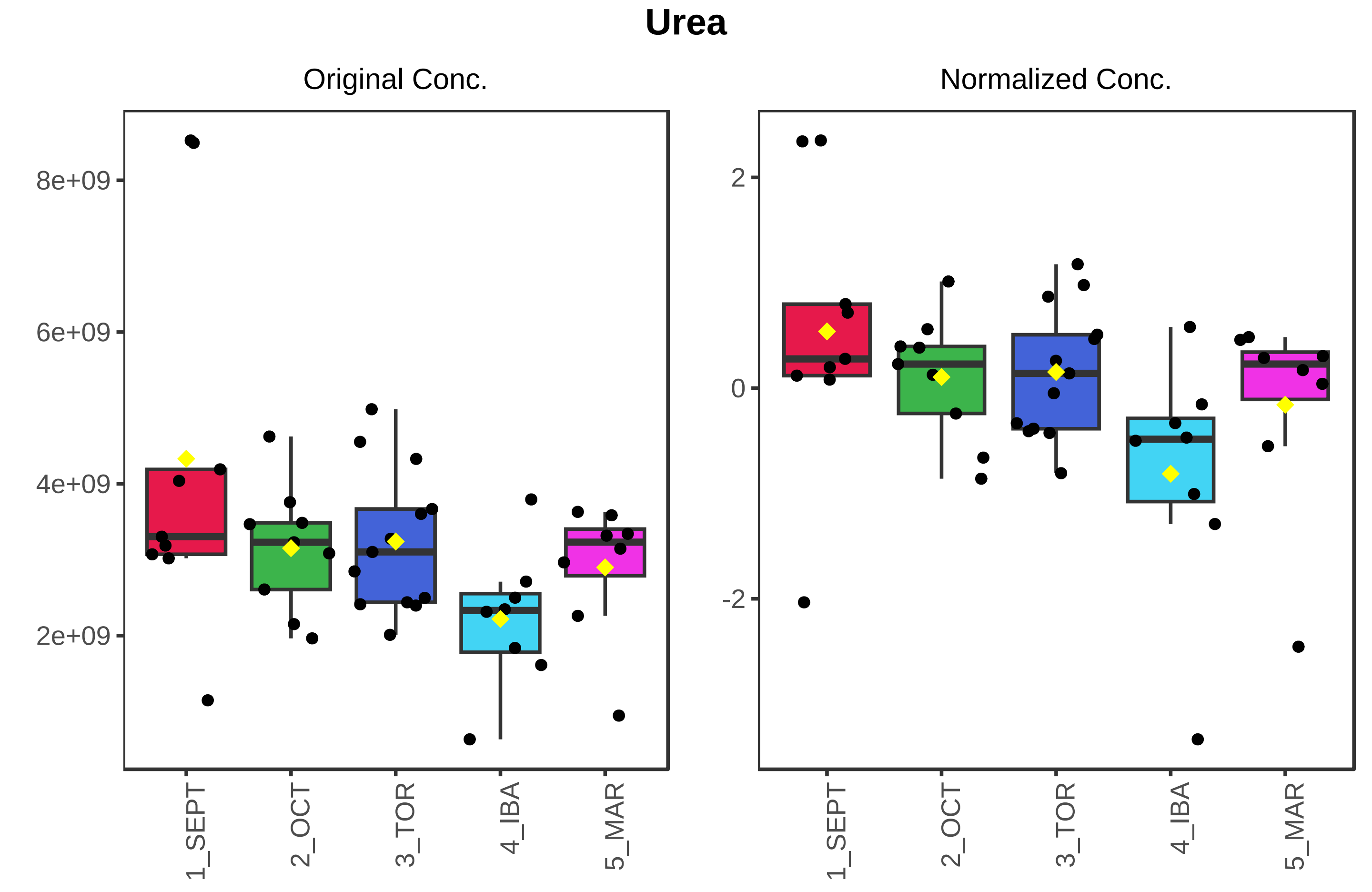

Urea
Original Conc.
Normalized Conc.
8e+09
6e+09
4e+09
2e+09
2
0
-2
1_SEPT
2_OCT
3_TOR
4_IBA
5_MAR

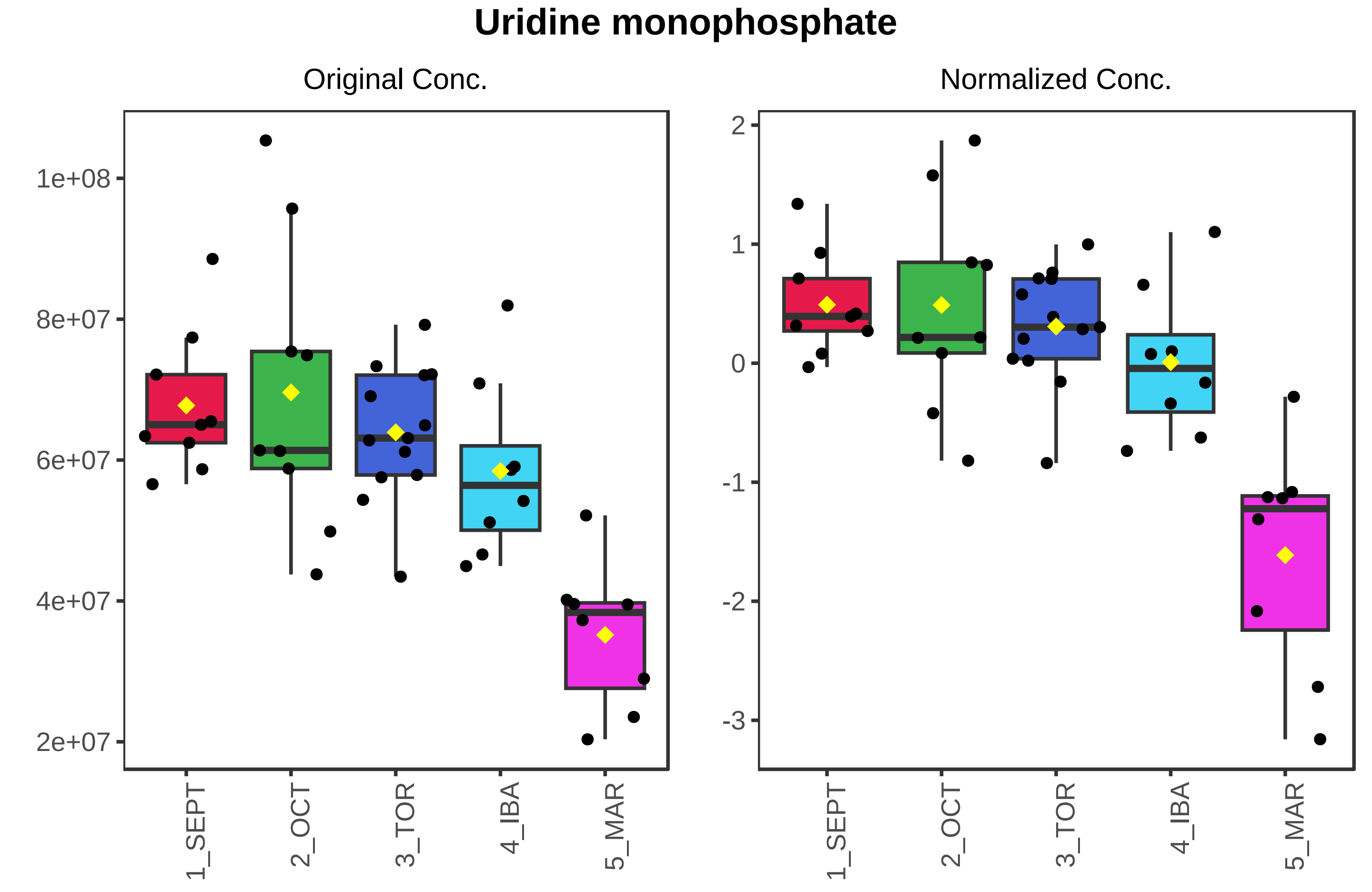
Uridine monophosphate
Original Conc.
Normalized Conc.
1e+08
8e+07
6e+07
4e+07
2e+07
2
1
0
-1
-2
-3
1_SEPT
2_OCT
3_TOR
4_IBA
5_MAR
1_SEPT
2_OCT
3_TOR
4_IBA
5_MAR

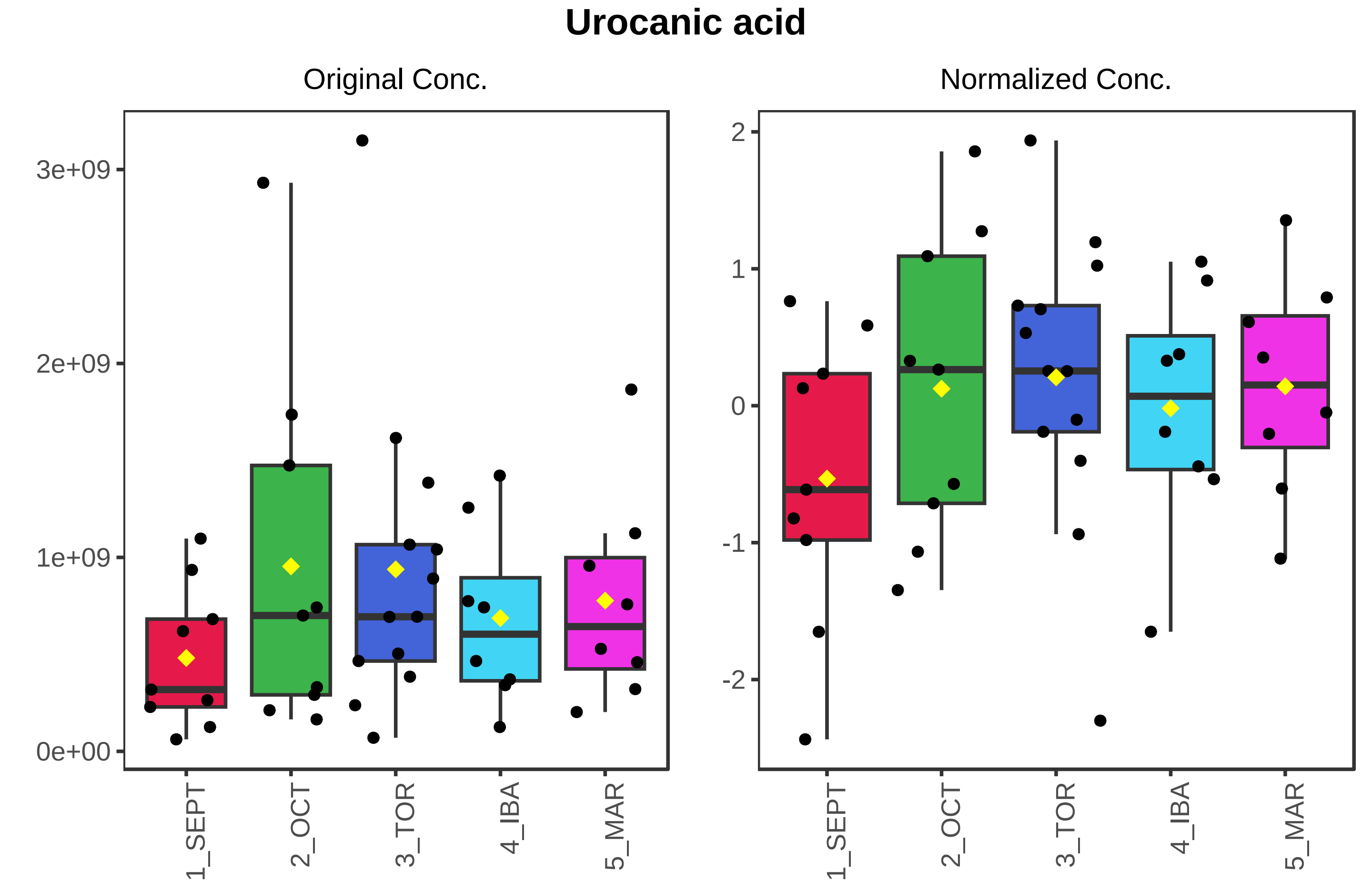

Urocanic acid
Original Conc.
Normalized Conc.
3e+09
2e+09
1e+09
0e+00
2
1
0
-1
-2
1_SEPT
2_OCT
3_TOR
4_IBA
5_MAR

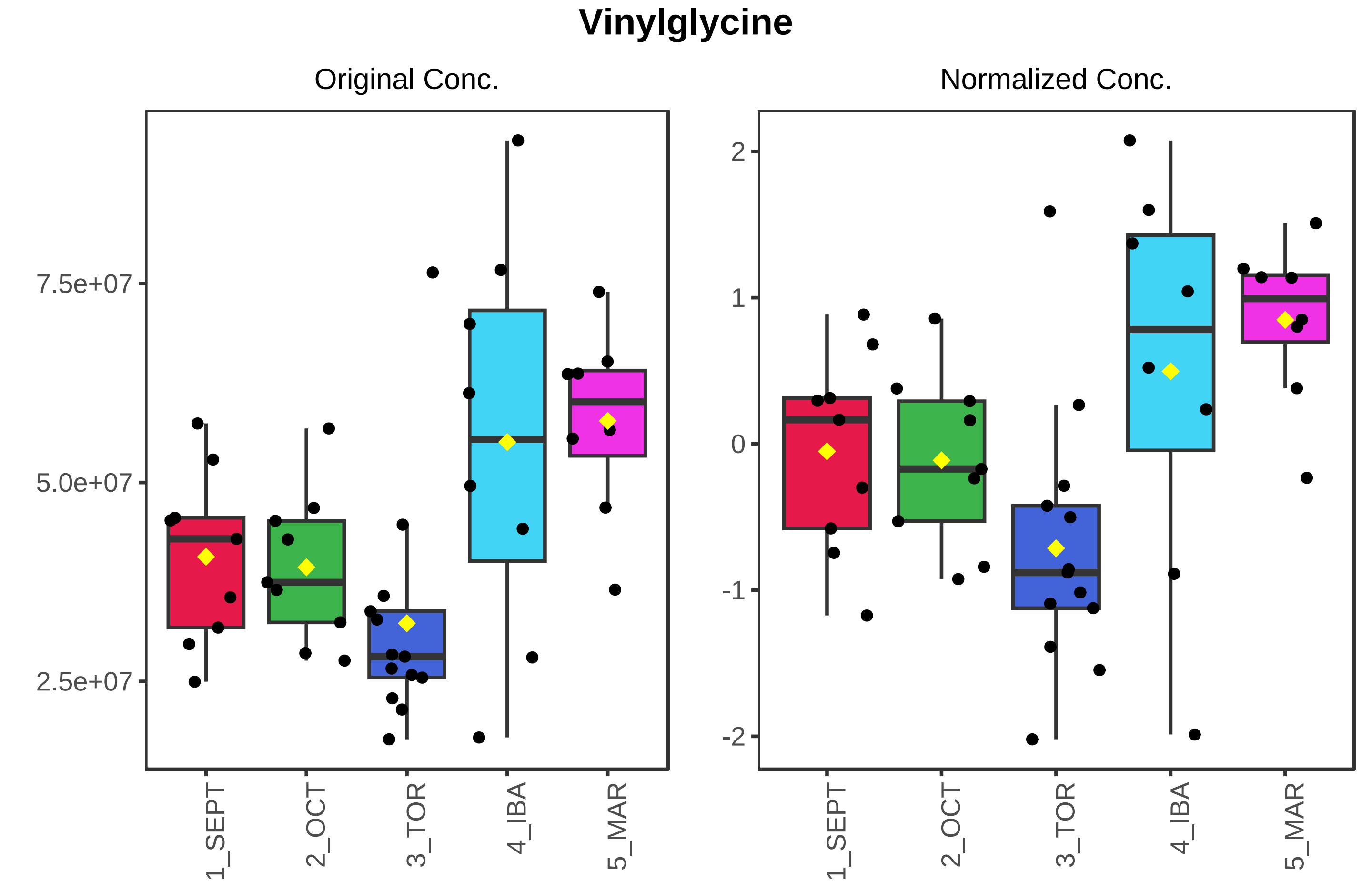

Vinylglycine
Original Conc.
Normalized Conc.
7.5e+07
5.0e+07
2.5e+07
2
1
0
-1
-2
1_SEPT
2_OCT
3_TOR
4_IBA
5_MAR
1_SEPT
2_OCT
3_TOR
4_IBA
5_MAR
