# Supplementary material for: Mass spectrometry of the white adipose metabolome in a hibernating mammal reveals seasonal changes in alternate fuels and carnitine derivatives
Source: Front Physiol. 2023 Jun 28;14:1214087. doi: 10.3389/fphys.2023.1214087 (PMC10337995; doi:10.3389/fphys.2023.1214087)
Supplement: Supplementary file 6 [file Image3.pdf]

Figure S3

Supplementary Figure 3: Boxplot Representations of all Annotated Features from the Negative Ion Mode

Collated feature intensity box plots for all annotated features in the negative ion mode. Left panel: original feature intensities from peak intensity table (Supplemental Table S2, tab 2). Right panel: feature intensities after $\log_{10}$ transformation and auto scaling (values mean-centered and divided by the standard deviation).

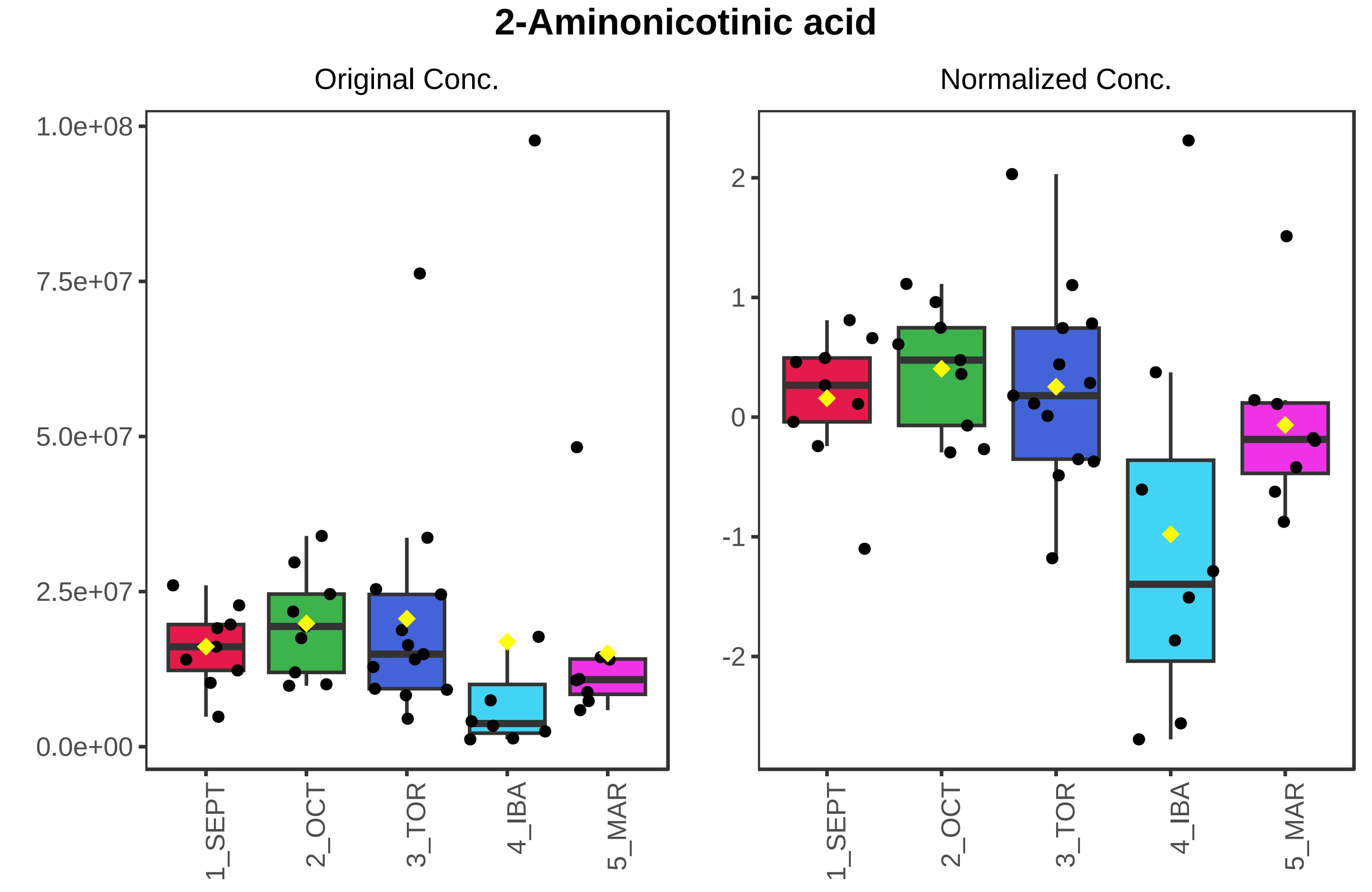

2-Aminonicotinic acid
Original Conc.
Normalized Conc.
1.0e+08
7.5e+07
5.0e+07
2.5e+07
0.0e+00
2
1
0
-1
-2
1_SEPT
2_OCT
3_TOR
4_IBA
5_MAR

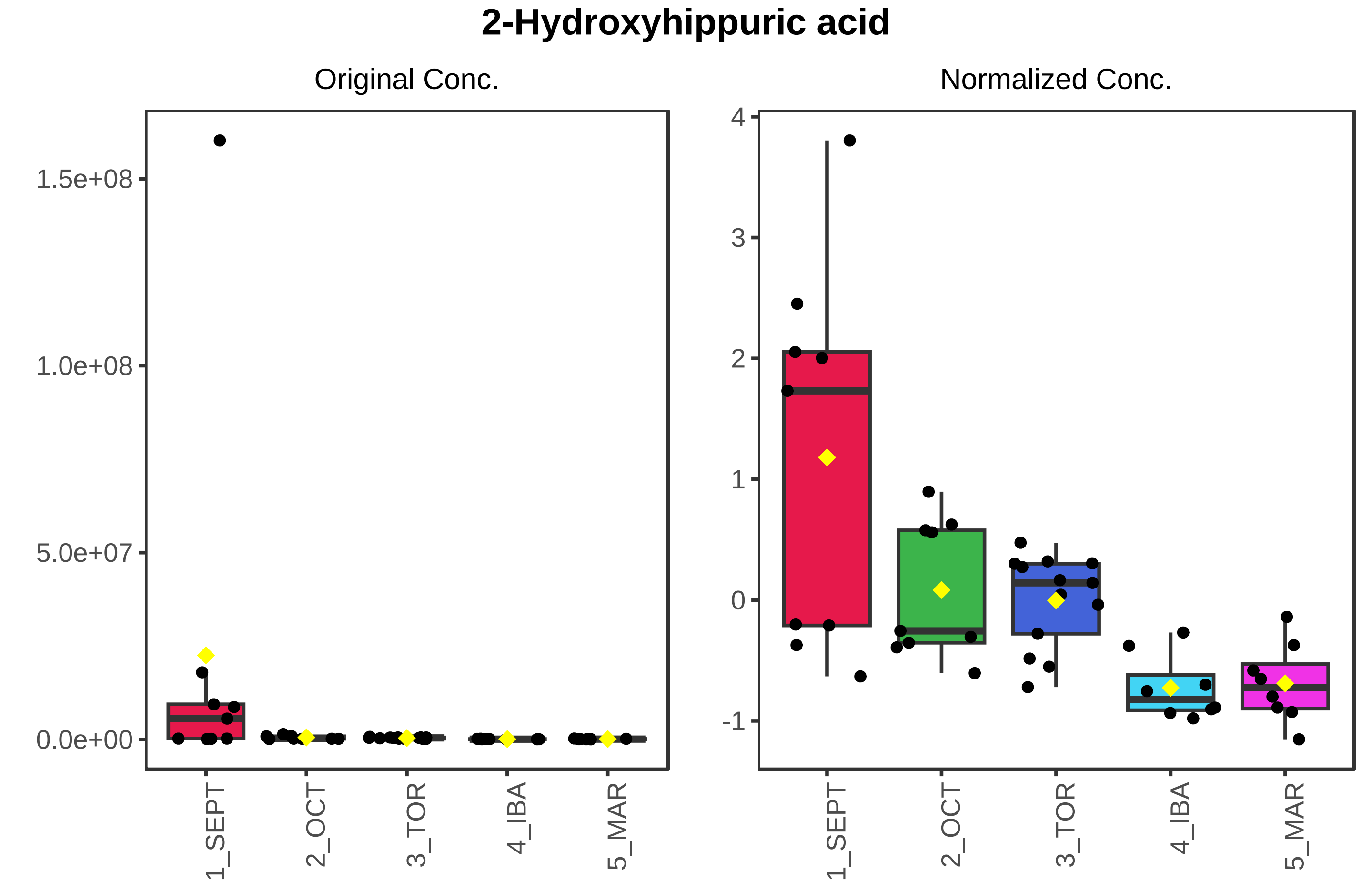

2-Hydroxyhippuric acid
Original Conc.
Normalized Conc.
1.5e+08
1.0e+08
5.0e+07
0.0e+00
4
3
2
1
0
-1
1_SEPT
2_OCT
3_TOR
4_IBA
5_MAR

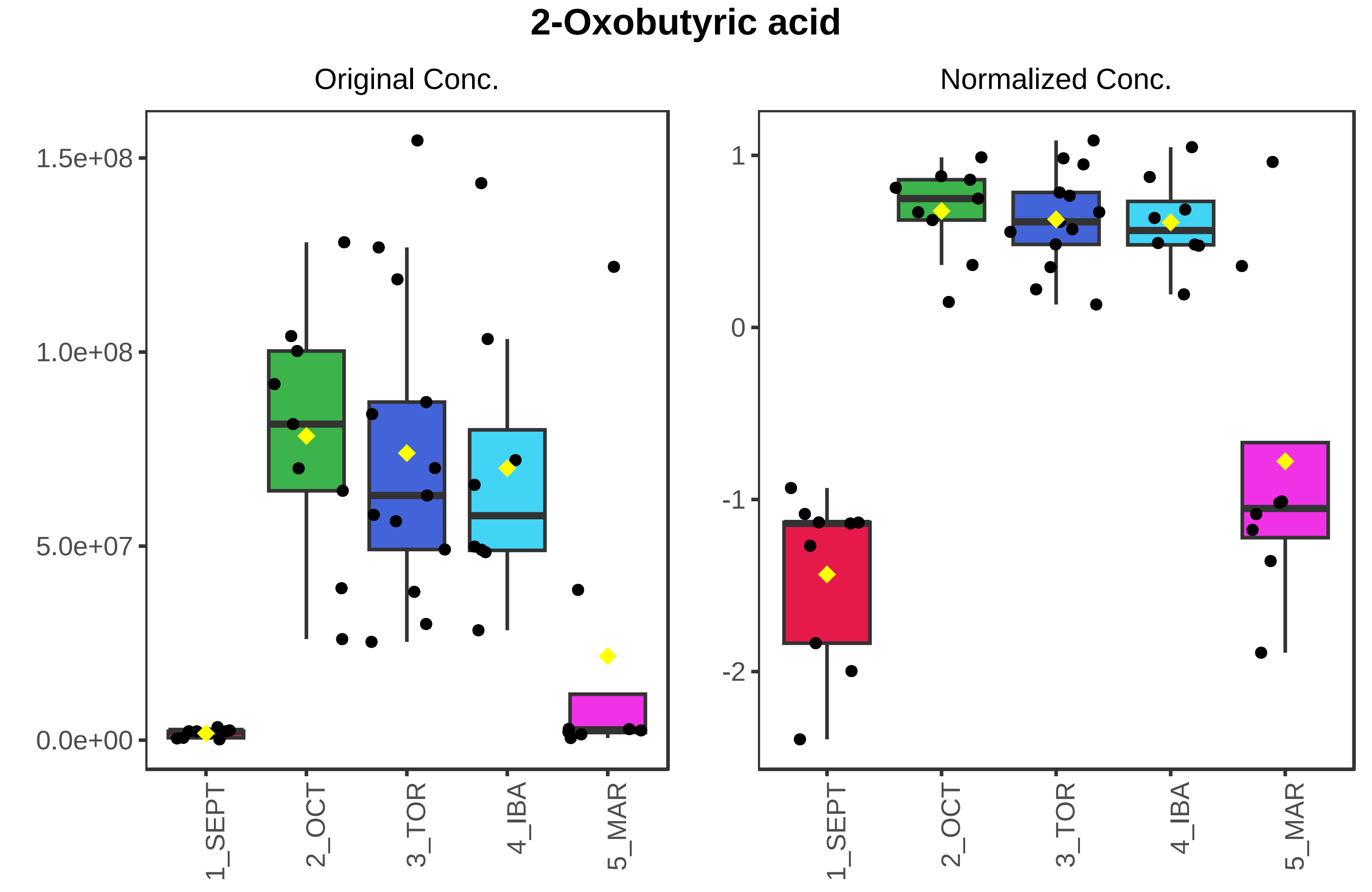

2-Oxobutyric acid
Original Conc.
Normalized Conc.
1.5e+08
1.0e+08
5.0e+07
0.0e+00
1
0
-1
-2
1_SEPT
2_OCT
3_TOR
4_IBA
5_MAR

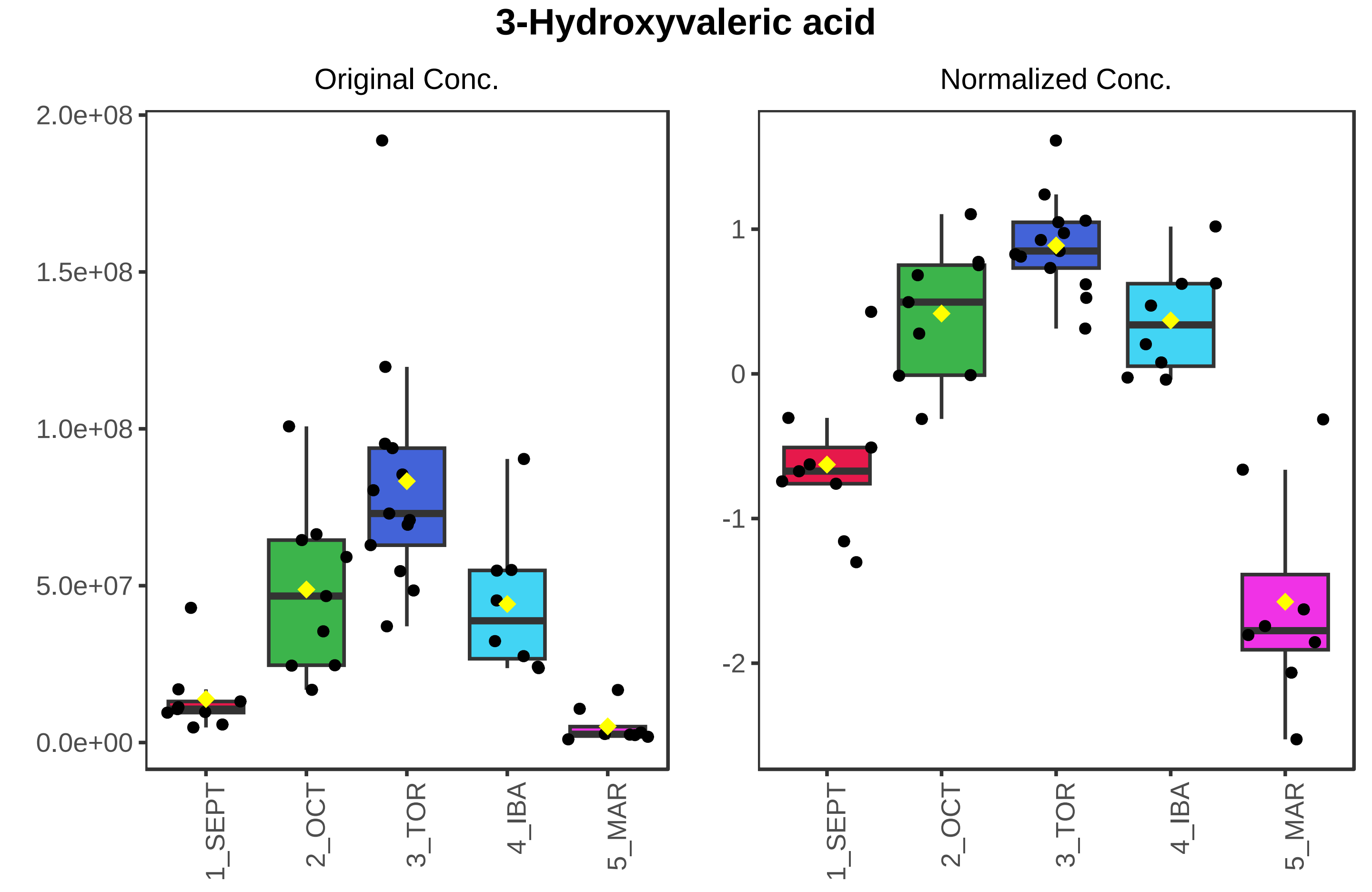
3-Hydroxyvaleric acid
Original Conc.
Normalized Conc.
2.0e+08
1.5e+08
1.0e+08
5.0e+07
0.0e+00
1
0
-1
-2
1_SEPT
2_OCT
3_TOR
4_IBA
5_MAR

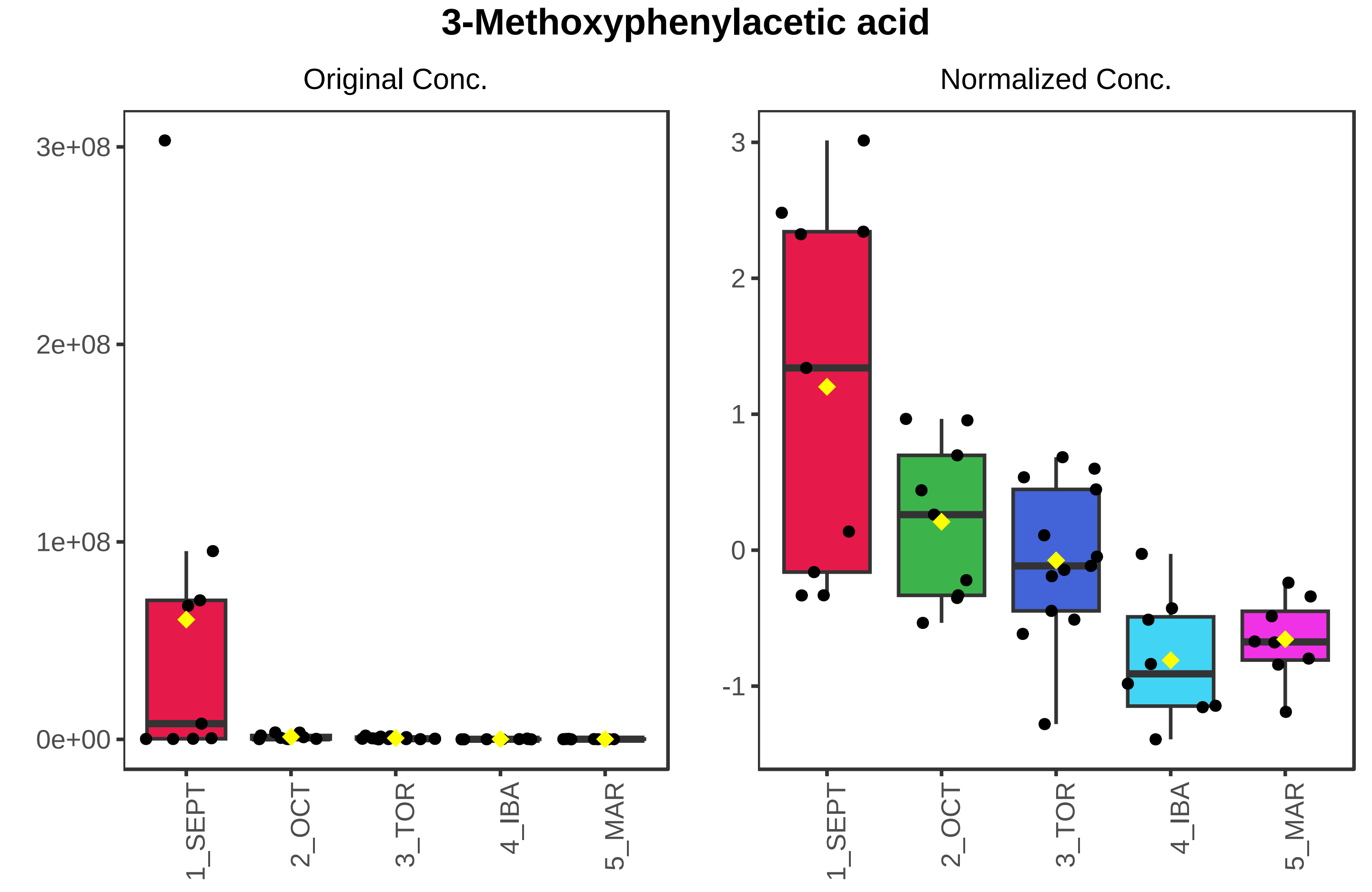
3-Methoxyphenylacetic acid
Original Conc.
Normalized Conc.
3e+08
2e+08
1e+08
0e+00
3
2
1
0
-1
1_SEPT
2_OCT
3_TOR
4_IBA
5_MAR

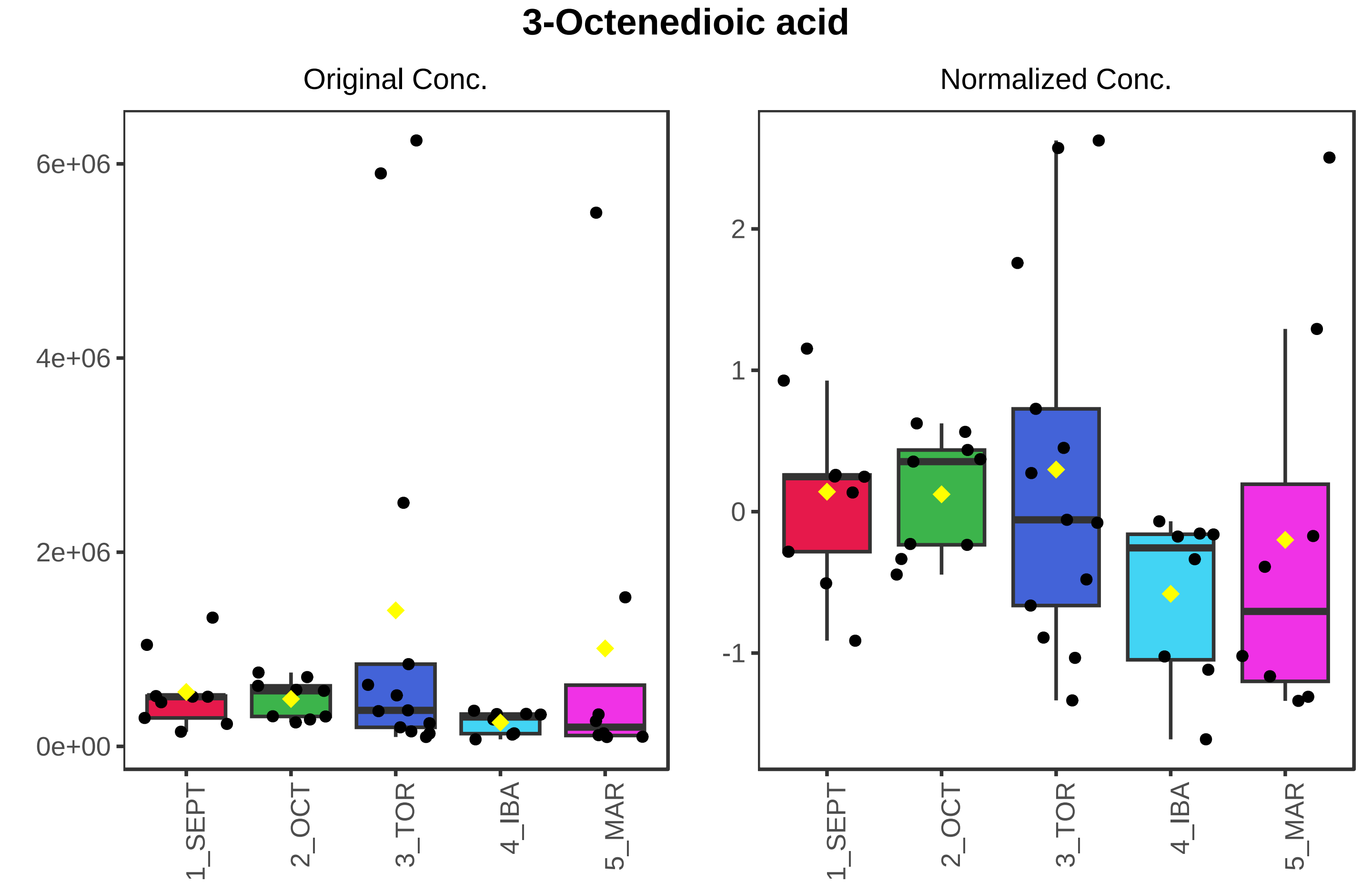
3-Octenedioic acid
Original Conc.
Normalized Conc.
6e+06
4e+06
2e+06
0e+00
2
1
0
-1
1_SEPT
2_OCT
3_TOR
4_IBA
5_MAR
1_SEPT
2_OCT
3_TOR
4_IBA
5_MAR

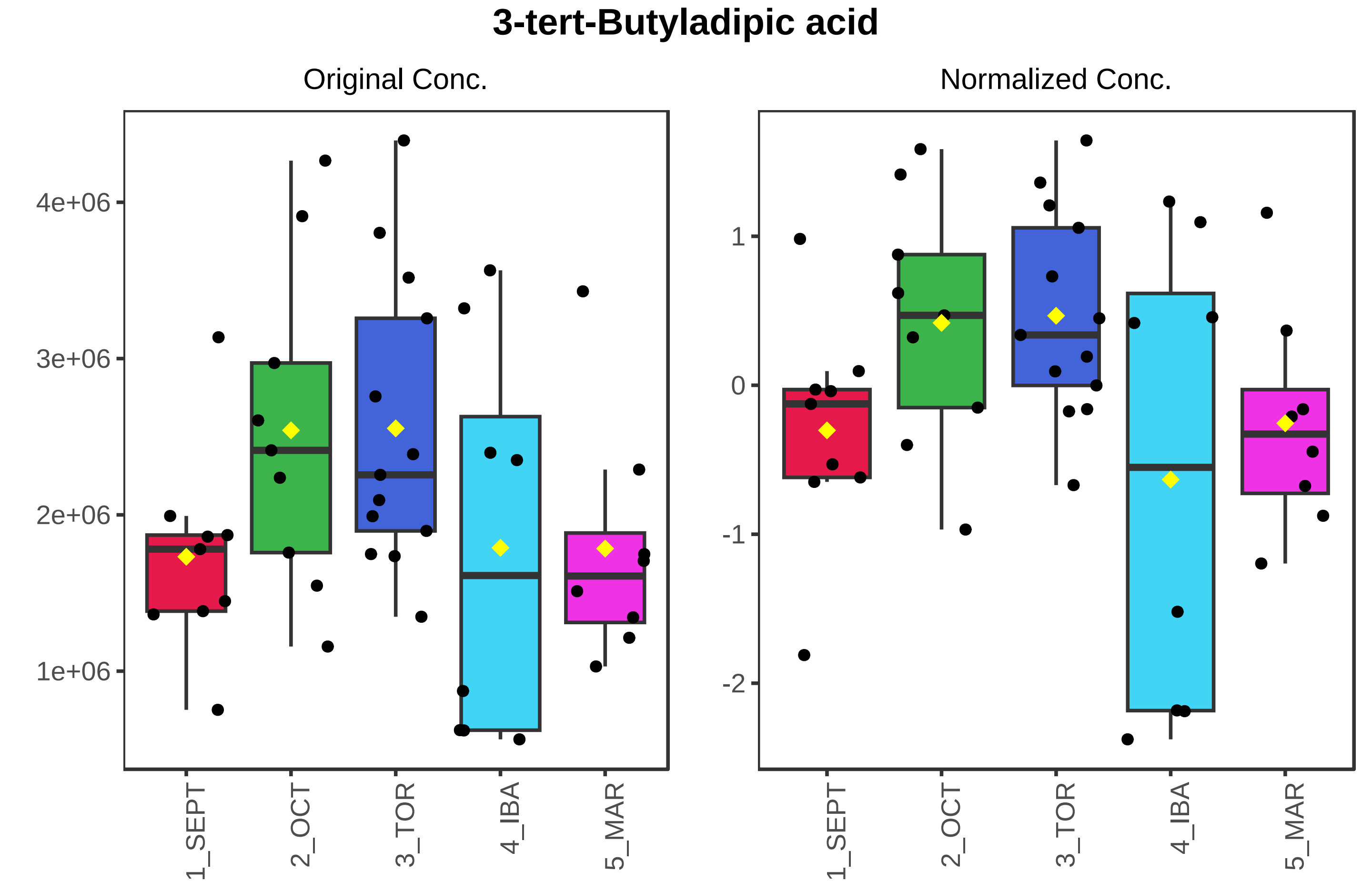

3-tert-Butyladipic acid
Original Conc.
Normalized Conc.
4e+06
3e+06
2e+06
1e+06
1
0
-1
-2
1_SEPT
2_OCT
3_TOR
4_IBA
5_MAR

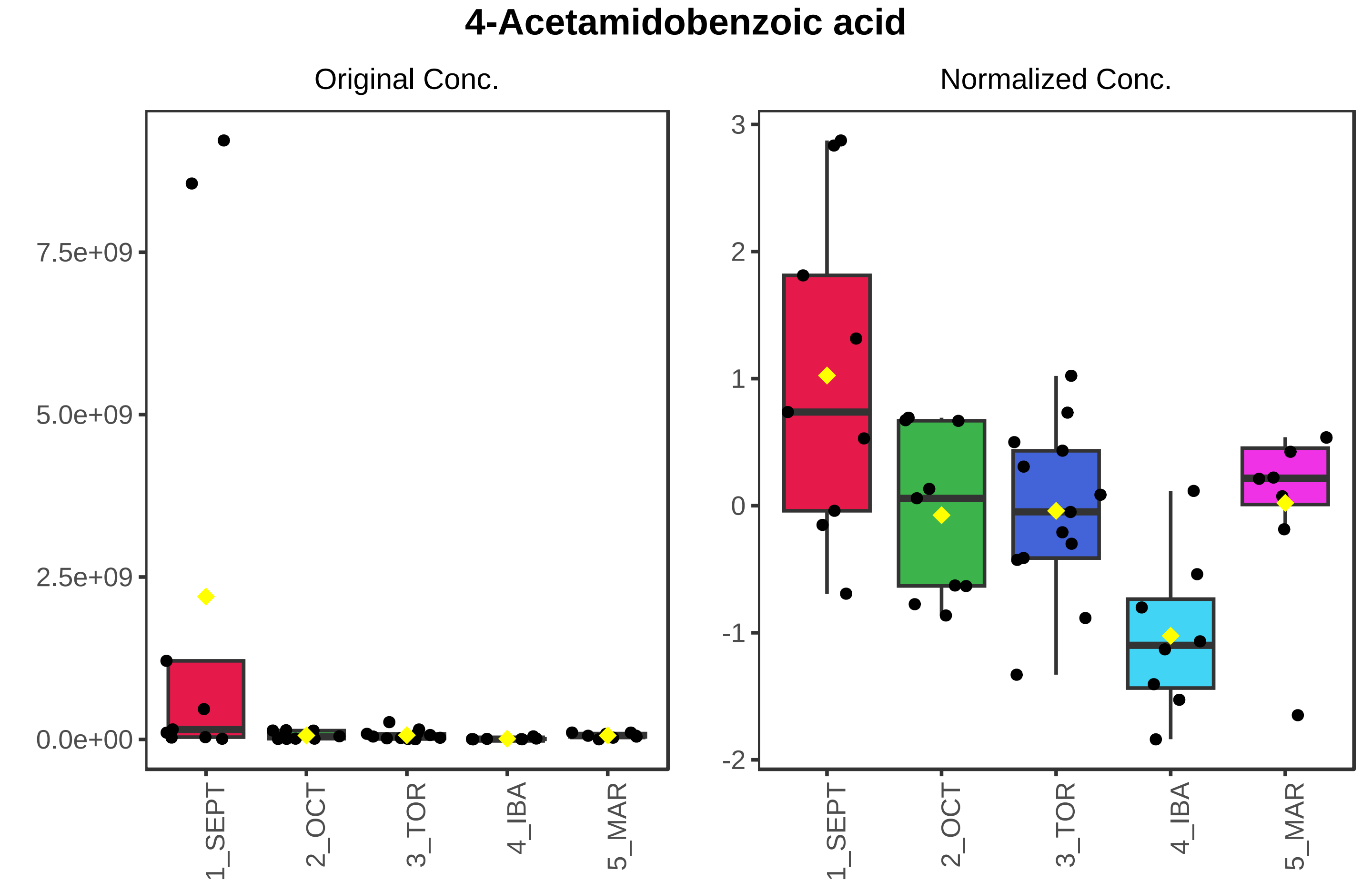
4-Acetamidobenzoic acid
Original Conc.
Normalized Conc.
7.5e+09
5.0e+09
2.5e+09
0.0e+00
3
2
1
0
-1
-2
1_SEPT
2_OCT
3_TOR
4_IBA
5_MAR

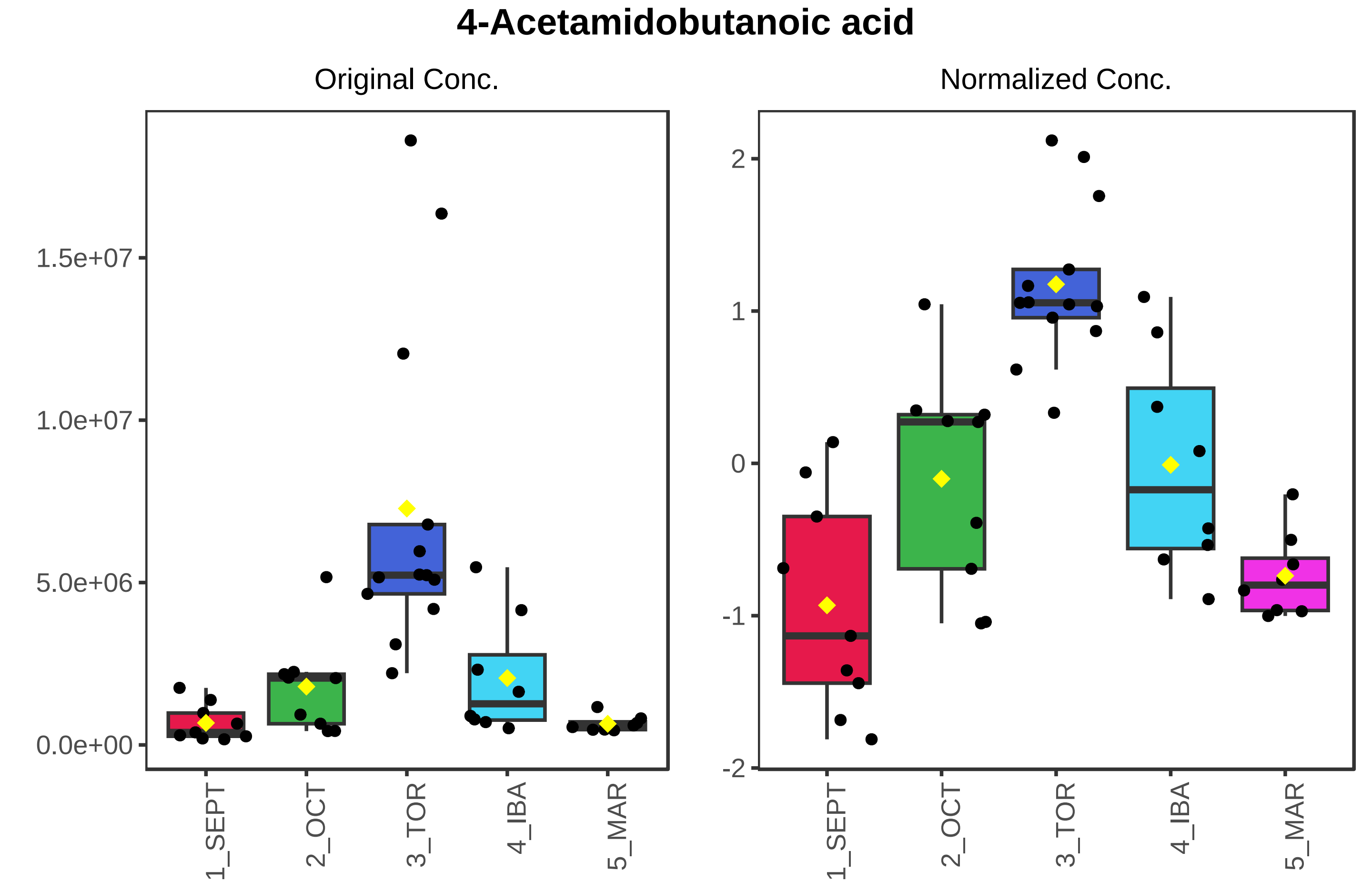
4-Acetamidobutanoic acid
Original Conc.
Normalized Conc.
1.5e+07
1.0e+07
5.0e+06
0.0e+00
2
1
0
-1
-2
1_SEPT
2_OCT
3_TOR
4_IBA
5_MAR
1_SEPT
2_OCT
3_TOR
4_IBA
5_MAR

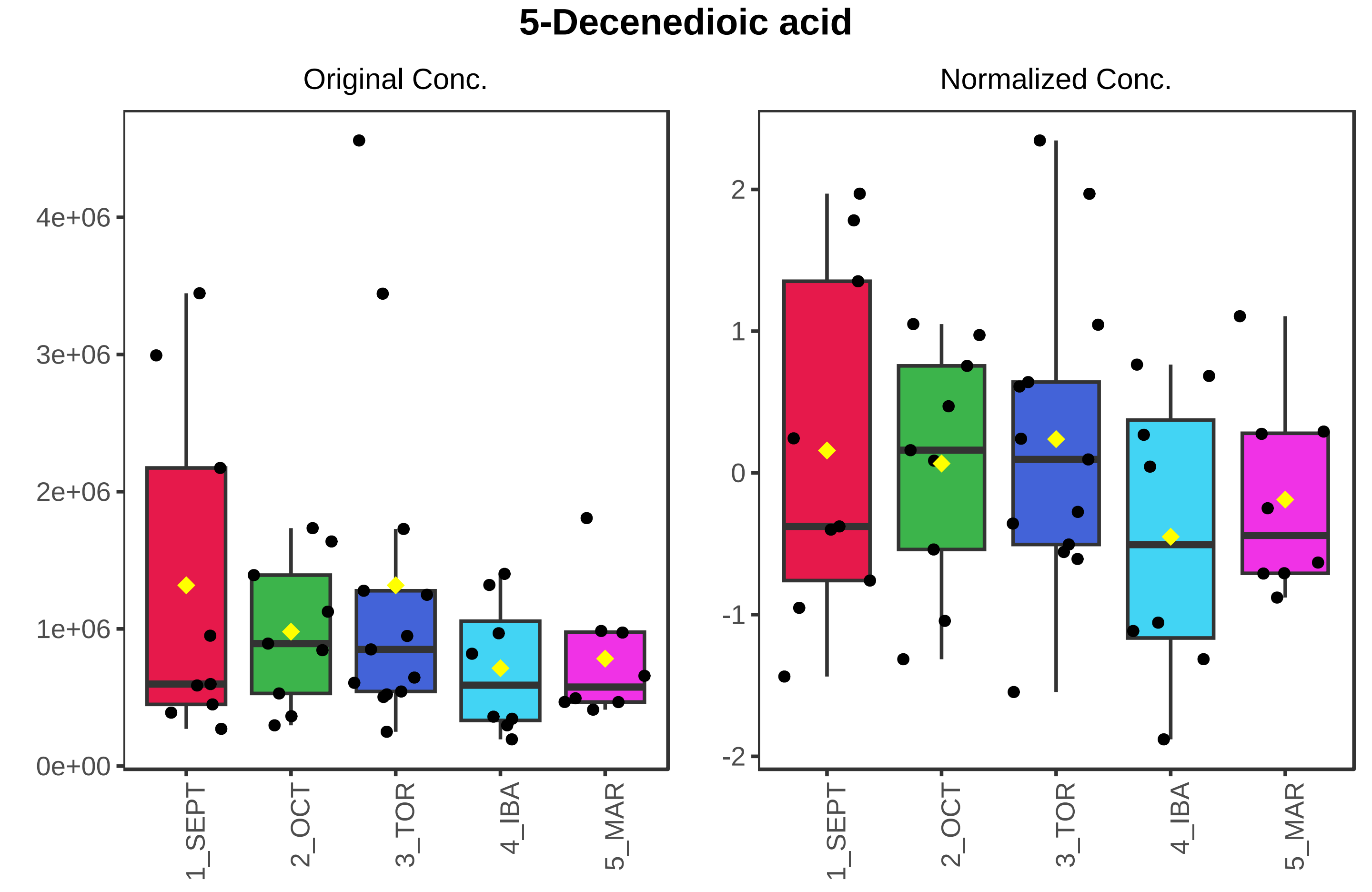

5-Decenedioic acid
Original Conc.
Normalized Conc.
4e+06
3e+06
2e+06
1e+06
0e+00
2
1
0
-1
-2
1_SEPT
2_OCT
3_TOR
4_IBA
5_MAR

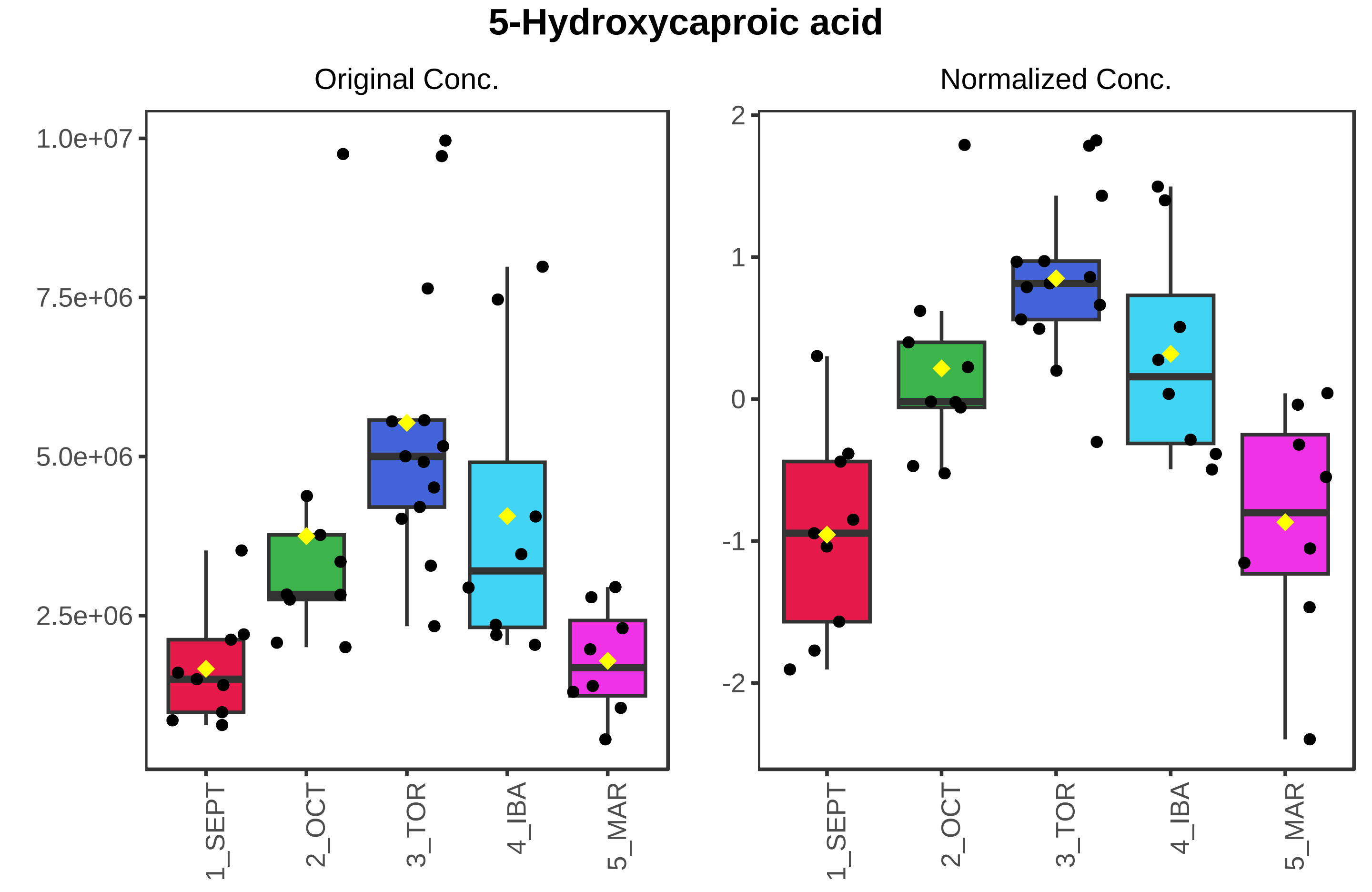
5-Hydroxycaproic acid
Original Conc.
Normalized Conc.
1.0e+07
7.5e+06
5.0e+06
2.5e+06
2
1
0
-1
-2
1_SEPT
2_OCT
3_TOR
4_IBA
5_MAR

5-Hydroxypipecolic acid

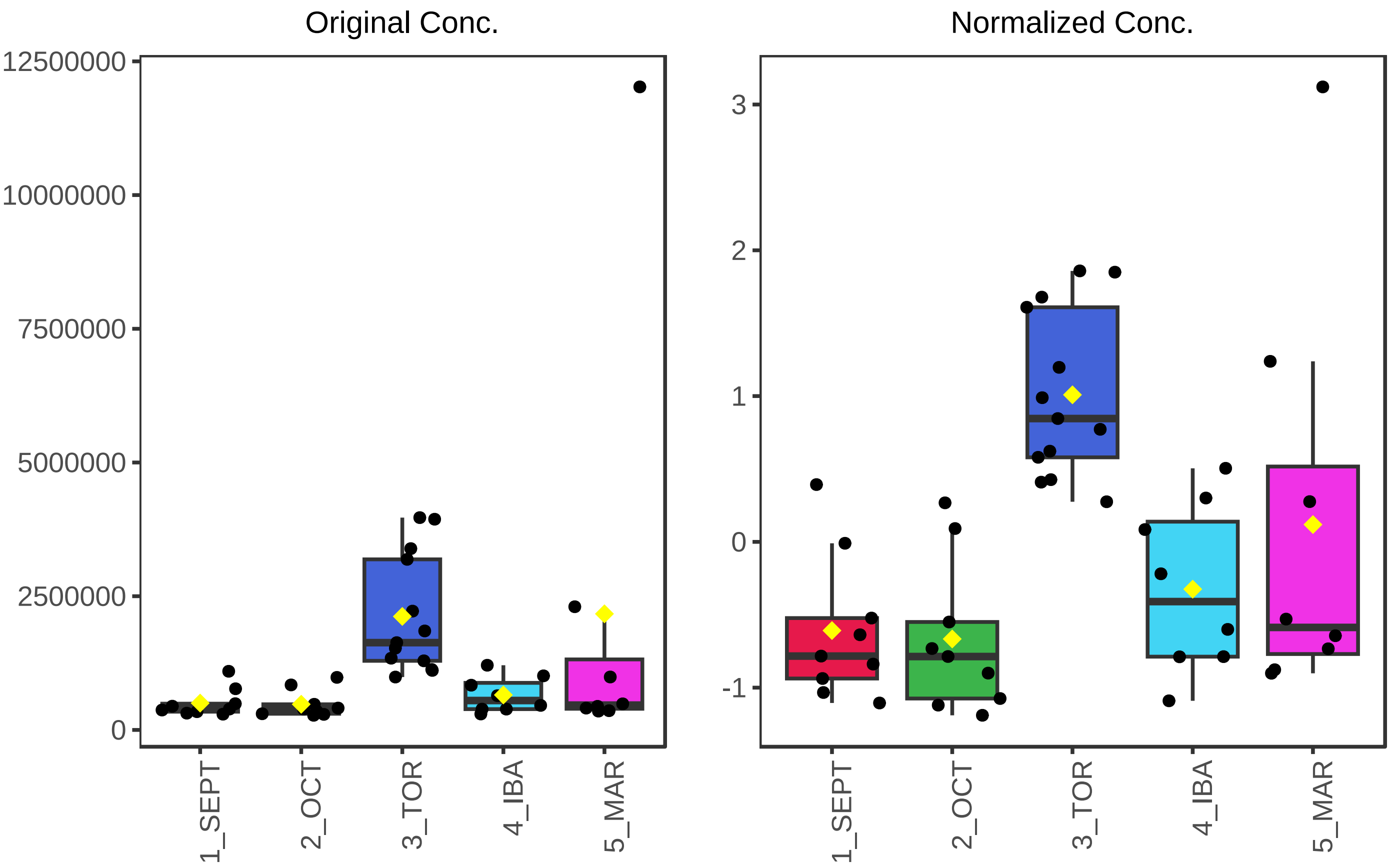

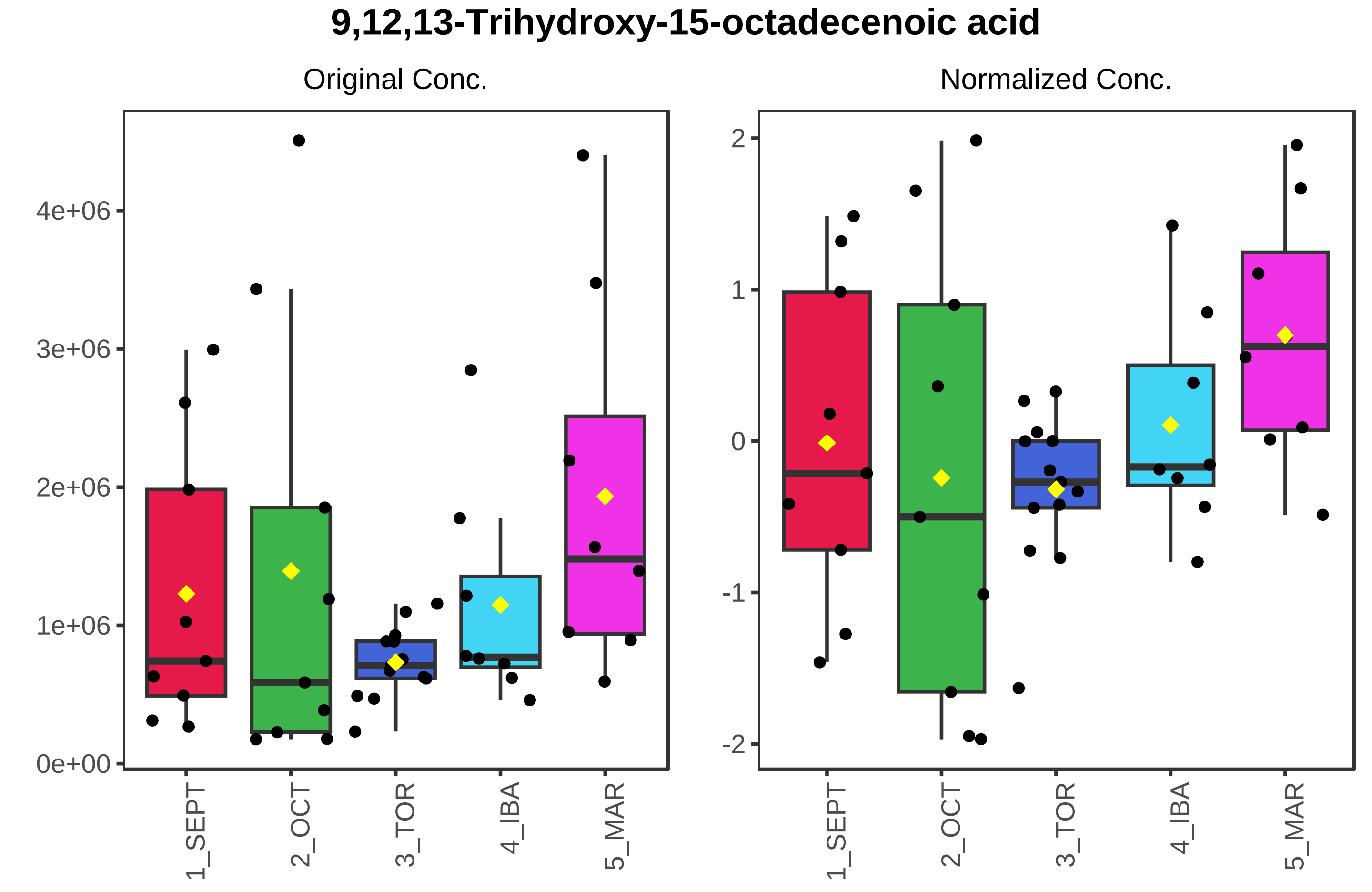
9,12,13-Trihydroxy-15-octadecenoic acid
Original Conc.
Normalized Conc.
4e+06
3e+06
2e+06
1e+06
0e+00
2
1
0
-1
-2
1_SEPT
2_OCT
3_TOR
4_IBA
5_MAR

Adenosine

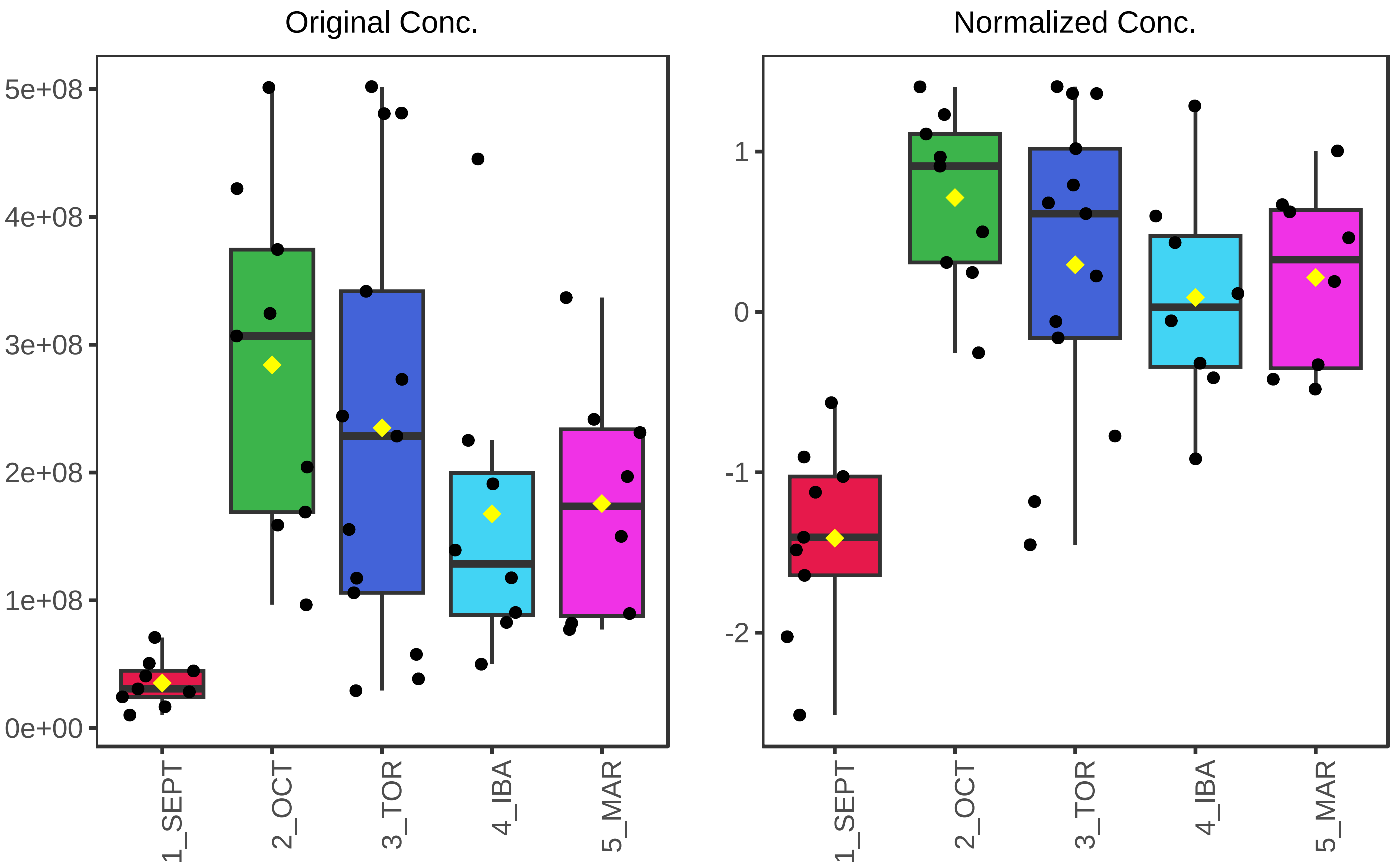

Original Conc.
Normalized Conc.
5e+08
4e+08
3e+08
2e+08
1e+08
0e+00
1
0
-1
-2
1_SEPT
2_OCT
3_TOR
4_IBA
5_MAR

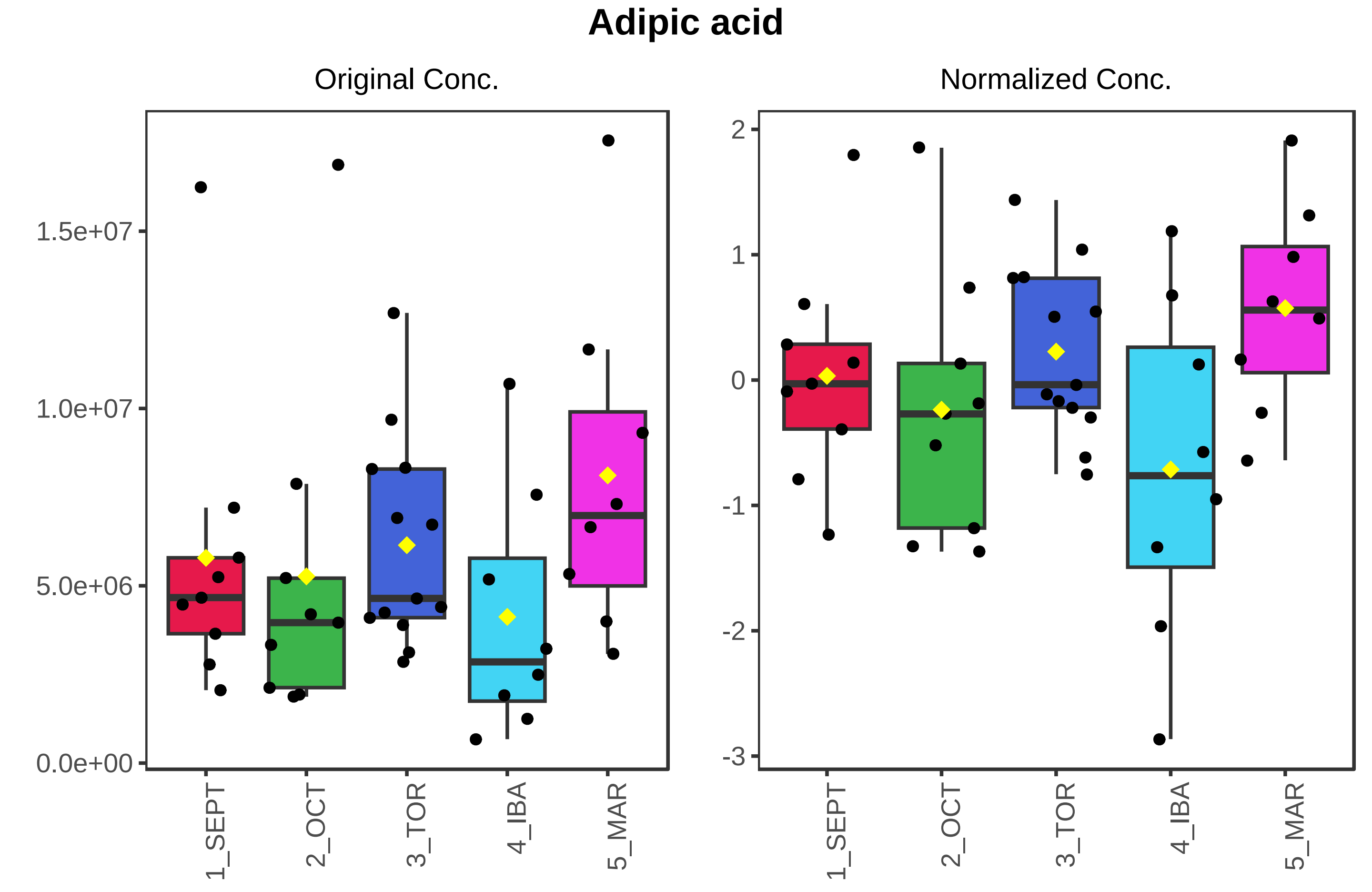

Adipic acid
Original Conc.
Normalized Conc.
1.5e+07
1.0e+07
5.0e+06
0.0e+00
2
1
0
-1
-2
-3
1_SEPT
2_OCT
3_TOR
4_IBA
5_MAR

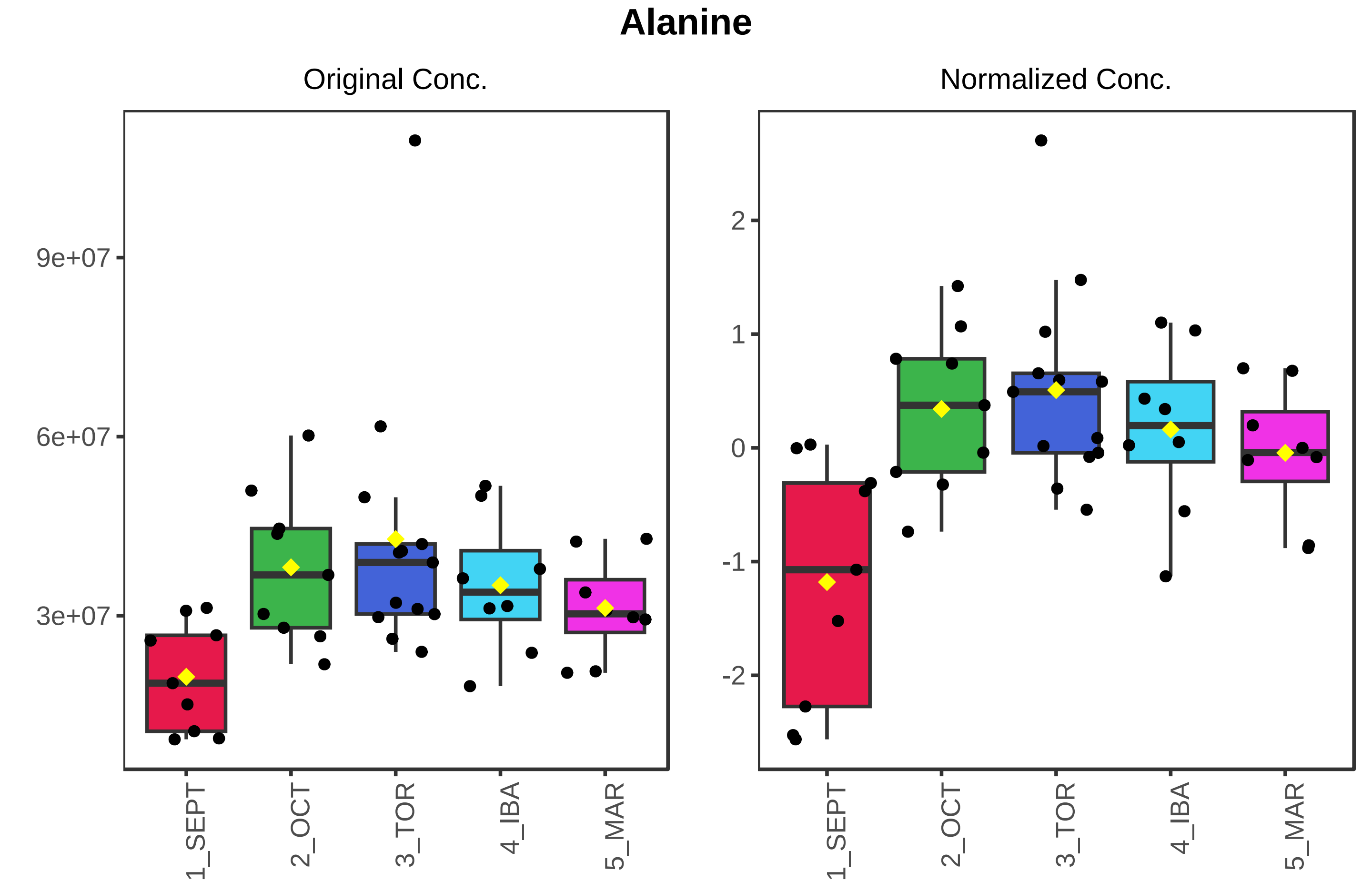
Alanine
Original Conc.
Normalized Conc.
9e+07
6e+07
3e+07
2
1
0
-1
-2
1_SEPT
2_OCT
3_TOR
4_IBA
5_MAR

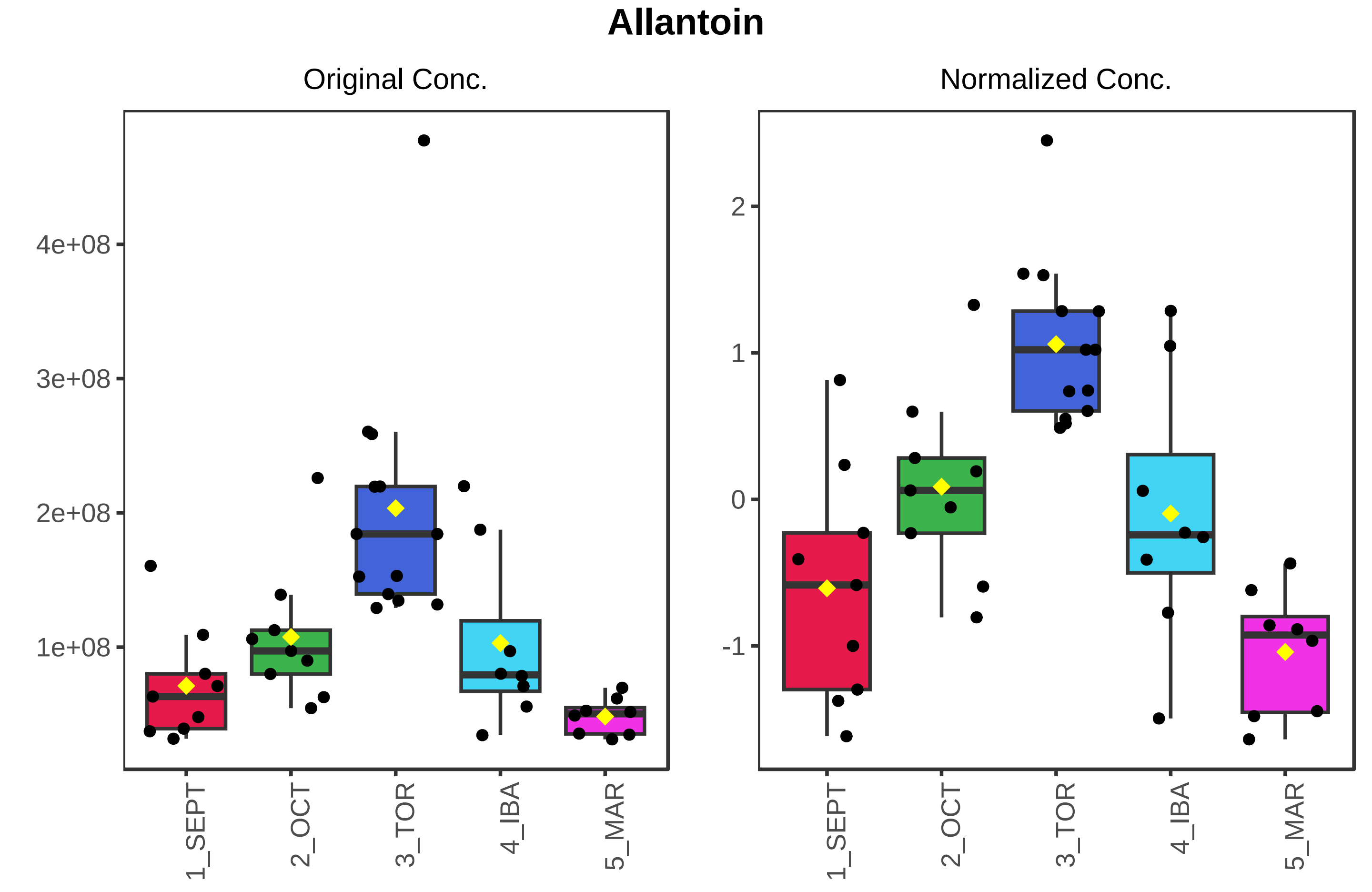

Allantoin
Original Conc.
Normalized Conc.
4e+08
3e+08
2e+08
1e+08
2
1
0
-1
1_SEPT
2_OCT
3_TOR
4_IBA
5_MAR

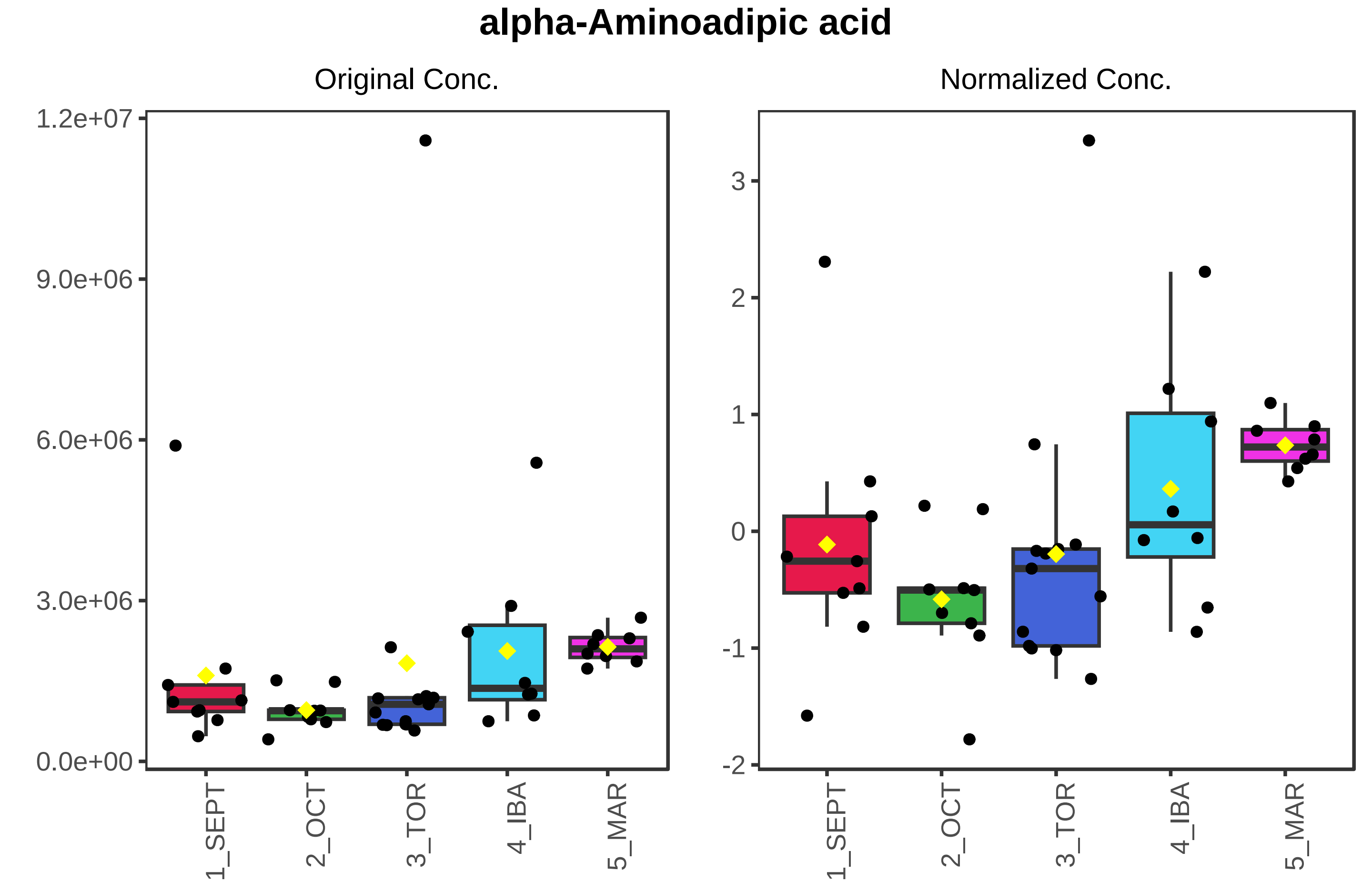
alpha-Aminoadipic acid
Original Conc.
Normalized Conc.
1.2e+07
9.0e+06
6.0e+06
3.0e+06
0.0e+00
3
2
1
0
-1
-2
1_SEPT
2_OCT
3_TOR
4_IBA
5_MAR

Aminolevulinic acid

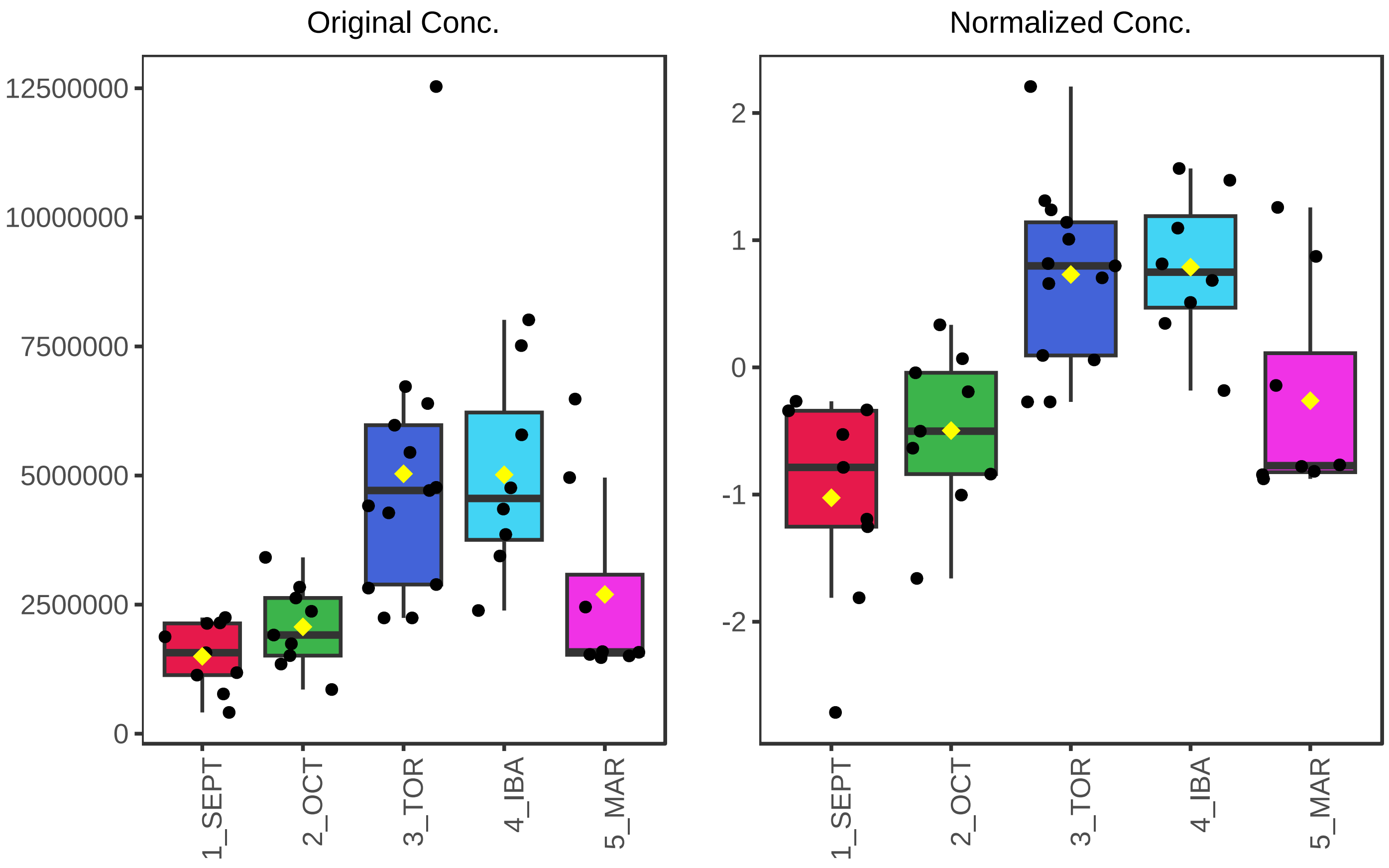

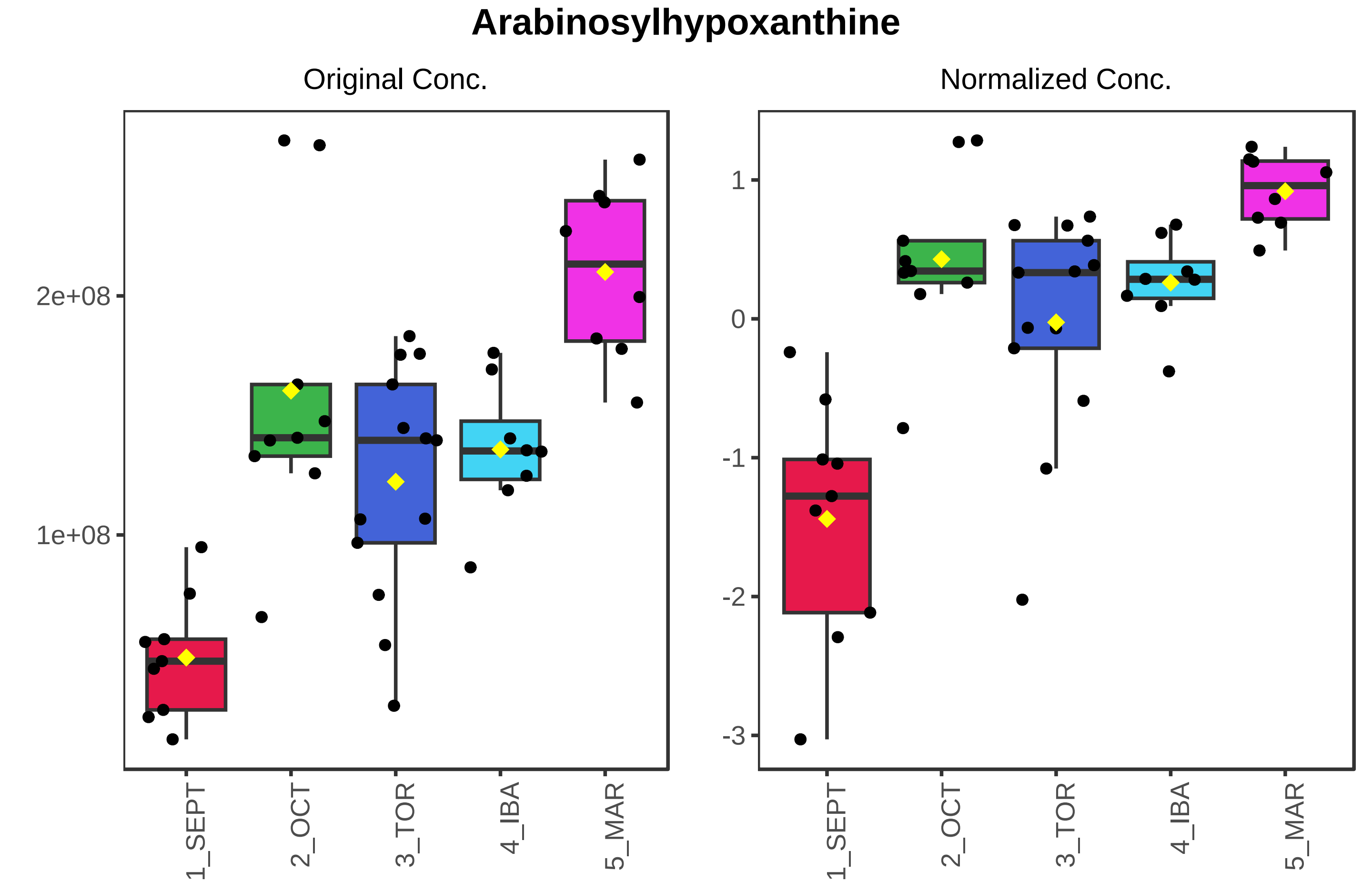
Arabinosylhypoxanthine
Original Conc.
Normalized Conc.
2e+08
1e+08
1
0
-1
-2
-3
1_SEPT
2_OCT
3_TOR
4_IBA
5_MAR
1_SEPT
2_OCT
3_TOR
4_IBA
5_MAR

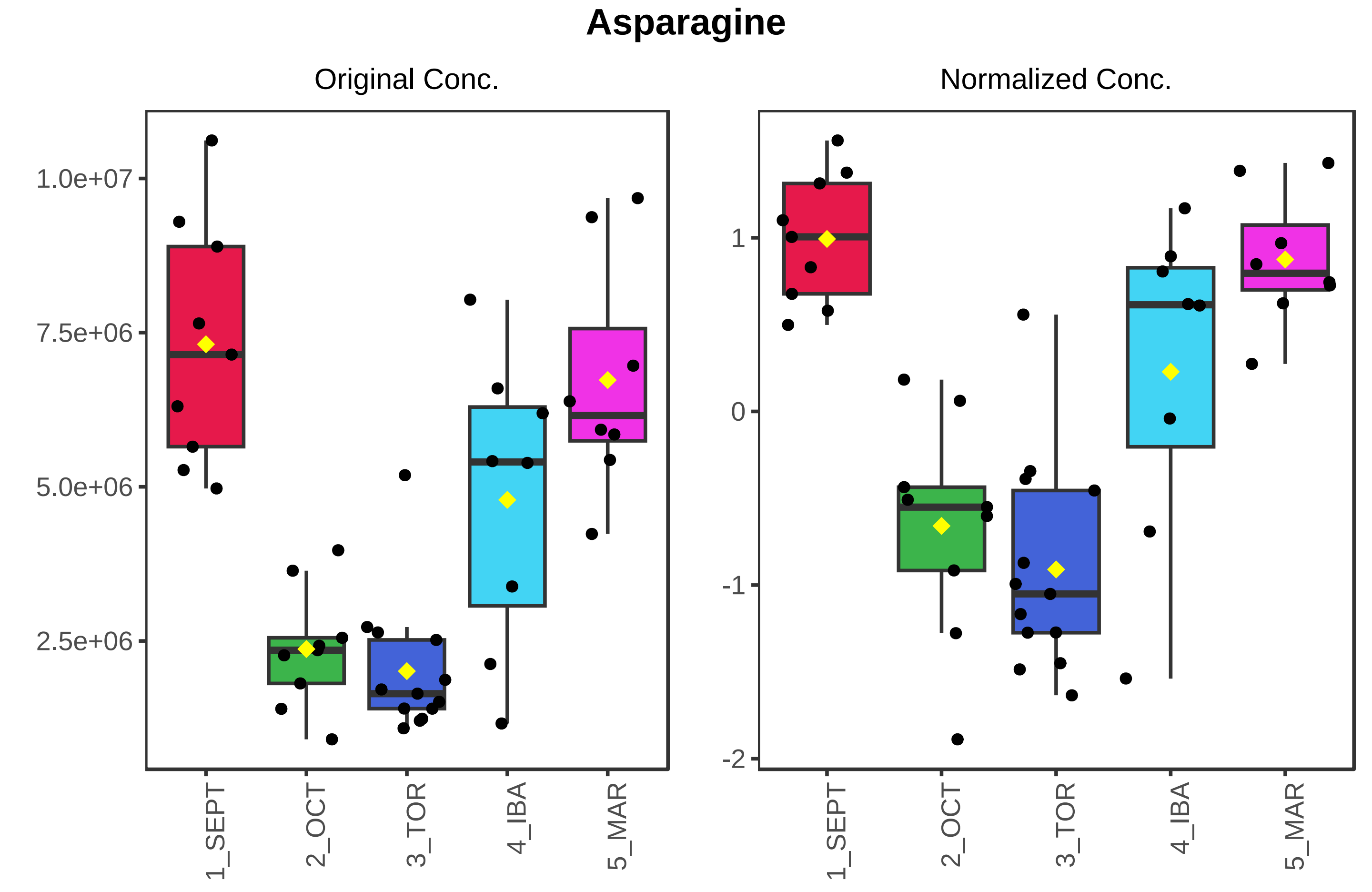

Asparagine
Original Conc.
Normalized Conc.
1.0e+07
7.5e+06
5.0e+06
2.5e+06
1
0
-1
-2
1_SEPT
2_OCT
3_TOR
4_IBA
5_MAR

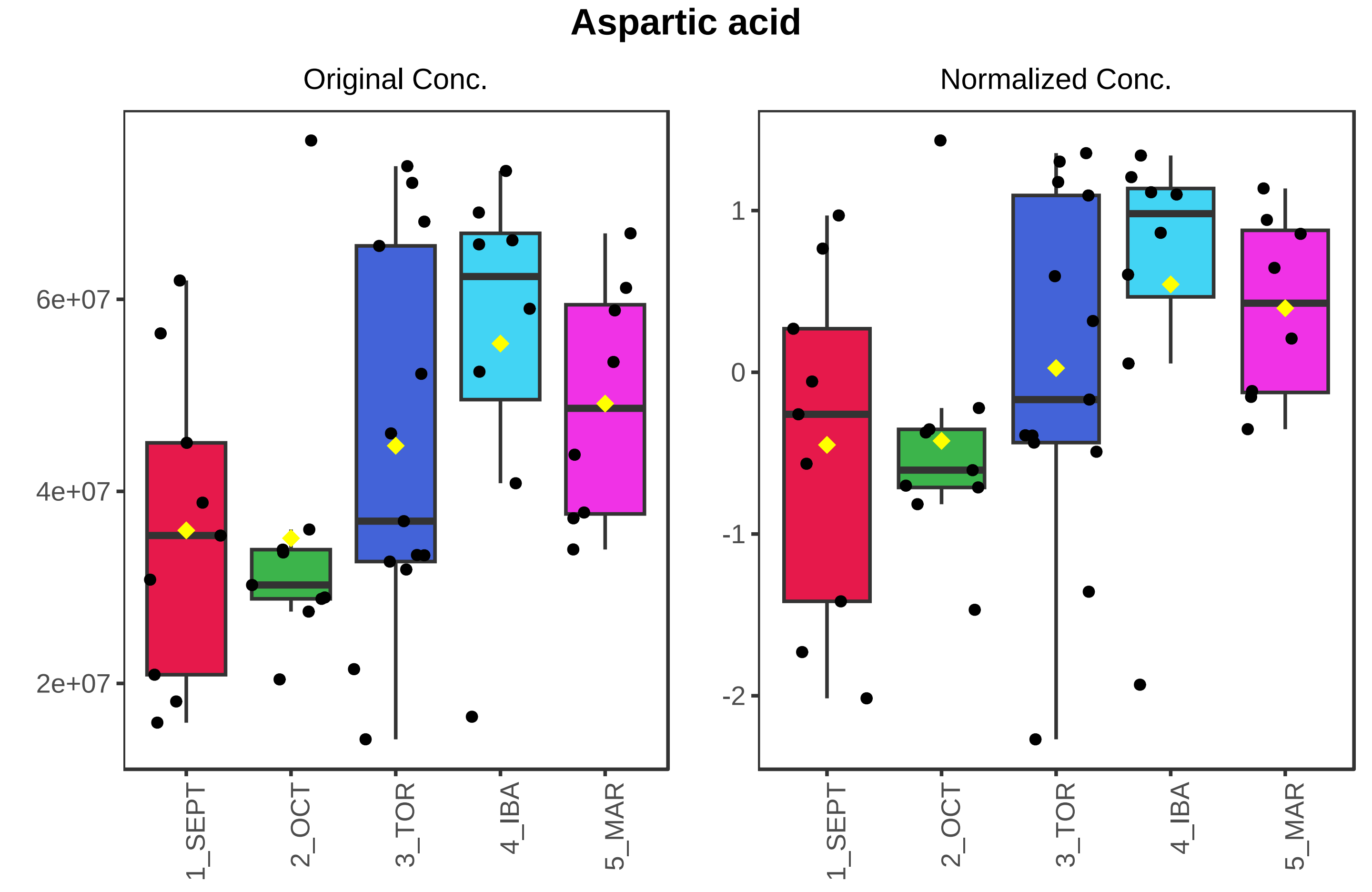

Aspartic acid
Original Conc.
Normalized Conc.
6e+07
4e+07
2e+07
1
0
-1
-2
1_SEPT
2_OCT
3_TOR
4_IBA
5_MAR

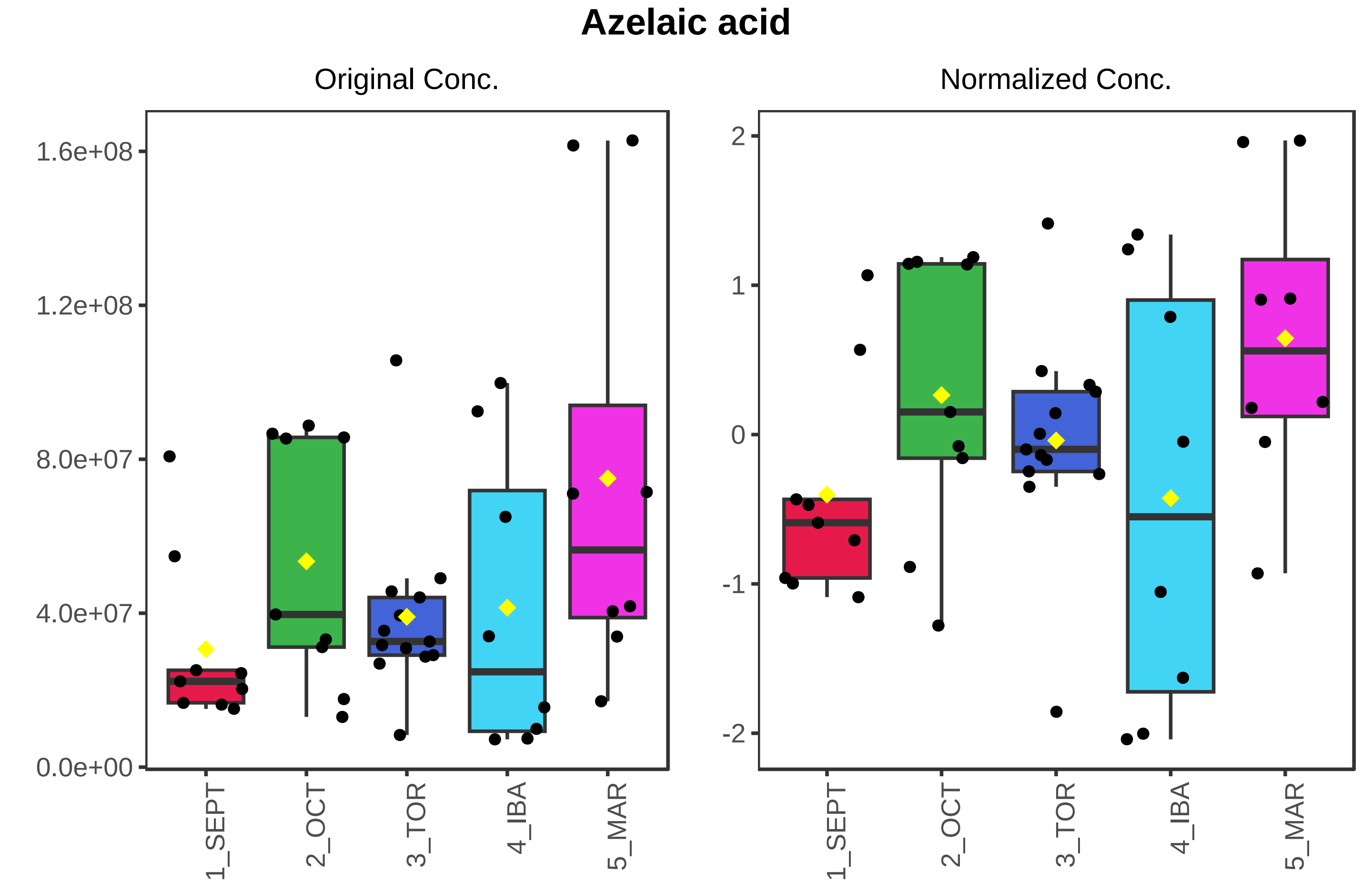

Azelaic acid
Original Conc.
Normalized Conc.
1.6e+08
1.2e+08
8.0e+07
4.0e+07
0.0e+00
2
1
0
-1
-2
1_SEPT
2_OCT
3_TOR
4_IBA
5_MAR

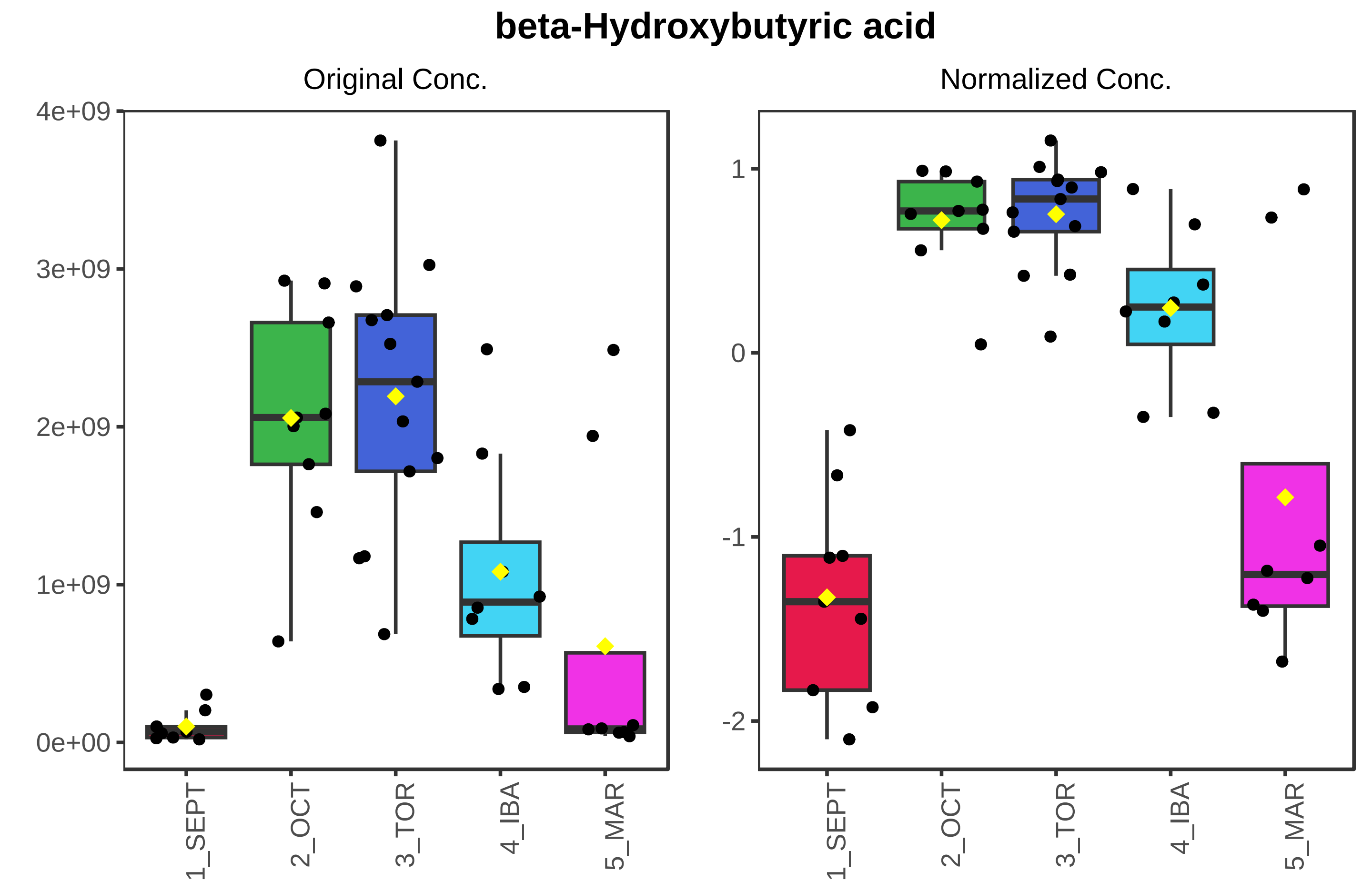
beta-Hydroxybutyric acid
Original Conc.
Normalized Conc.
4e+09
3e+09
2e+09
1e+09
0e+00
1
0
-1
-2
1_SEPT
2_OCT
3_TOR
4_IBA
5_MAR

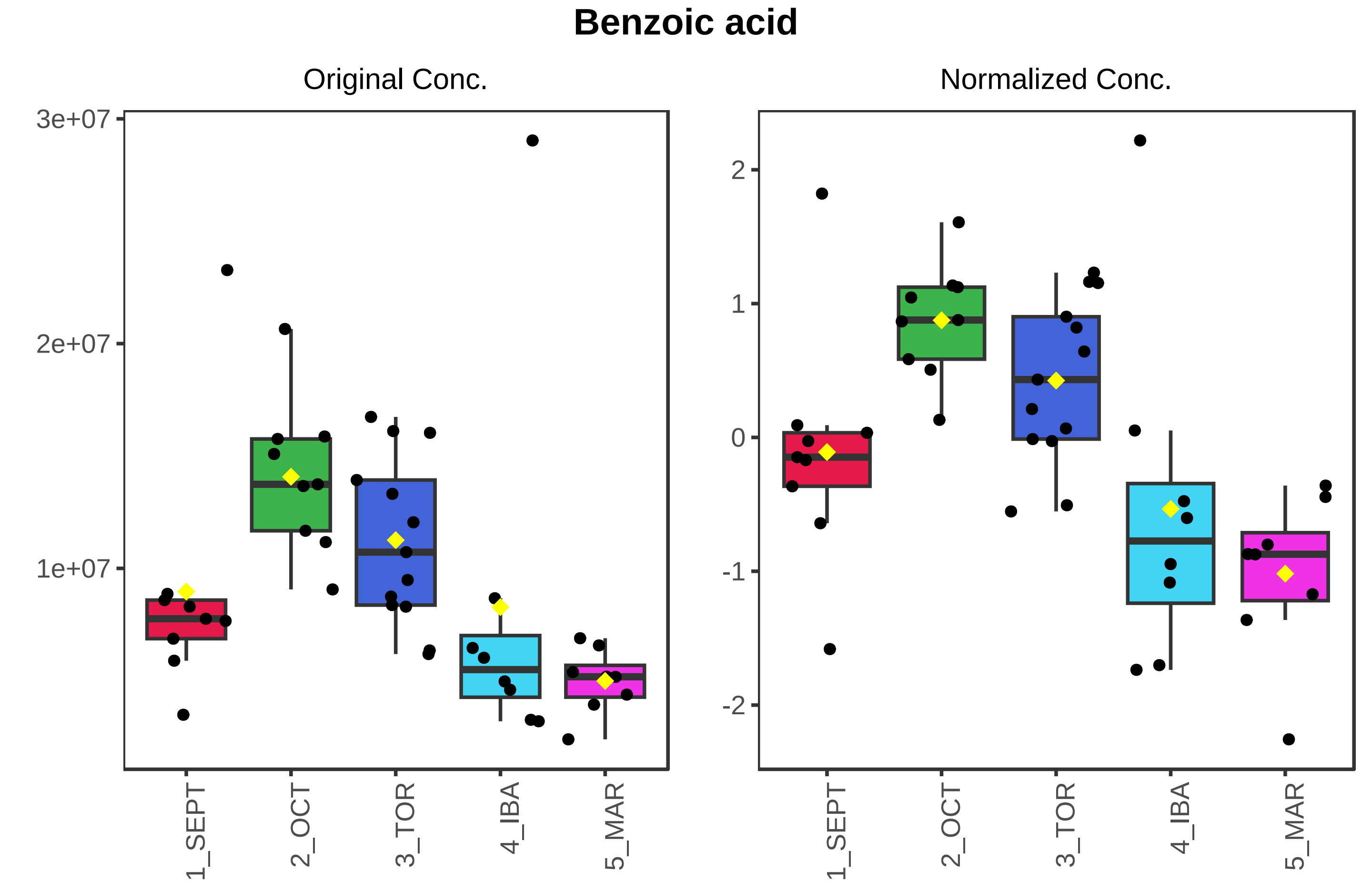

Benzoic acid
Original Conc.
Normalized Conc.
3e+07
2e+07
1e+07
2
1
0
-1
-2
1_SEPT
2_OCT
3_TOR
4_IBA
5_MAR

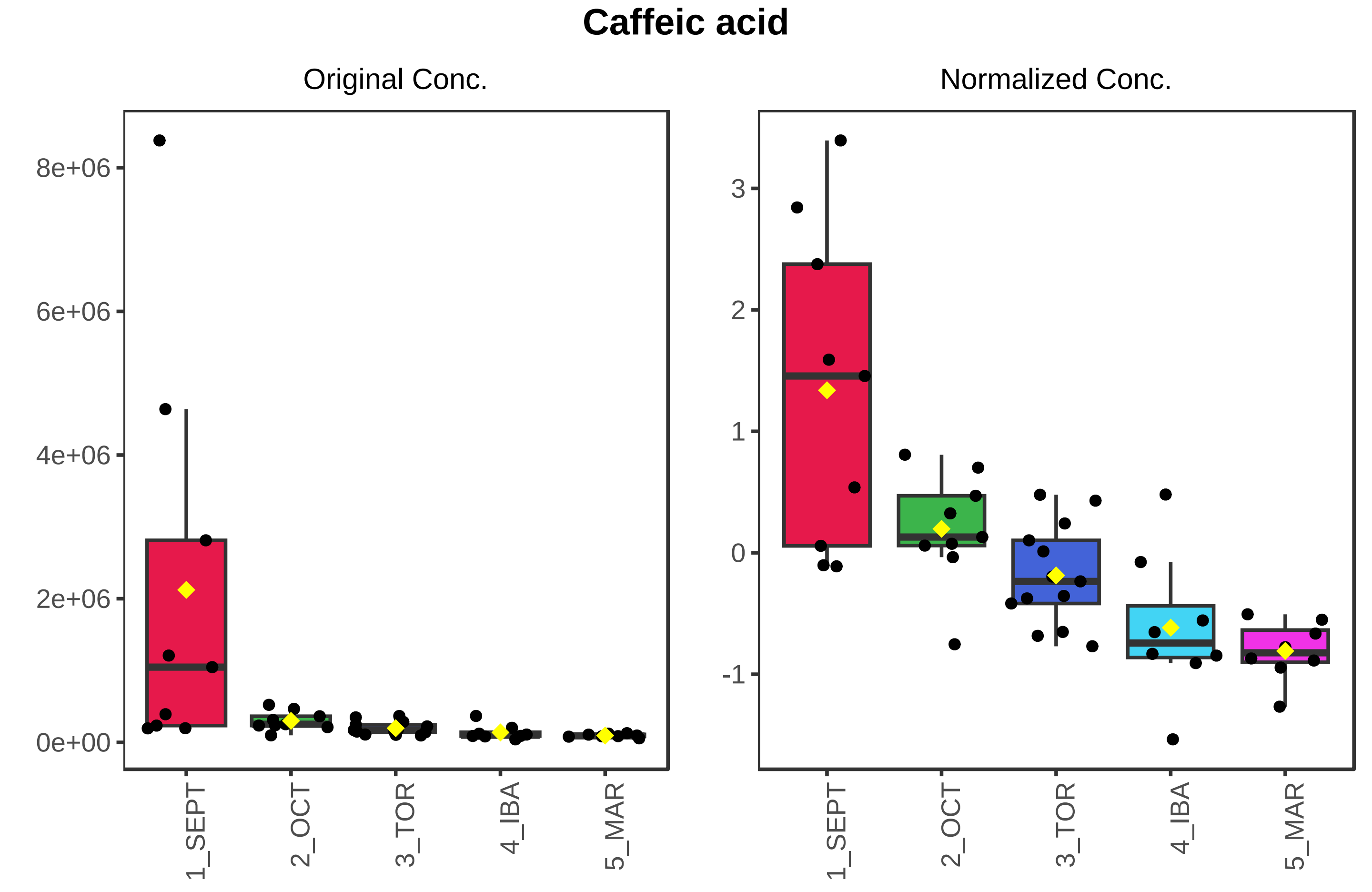
Caffeic acid
Original Conc.
Normalized Conc.
8e+06
6e+06
4e+06
2e+06
0e+00
3
2
1
0
-1
1_SEPT
2_OCT
3_TOR
4_IBA
5_MAR
1_SEPT
2_OCT
3_TOR
4_IBA
5_MAR

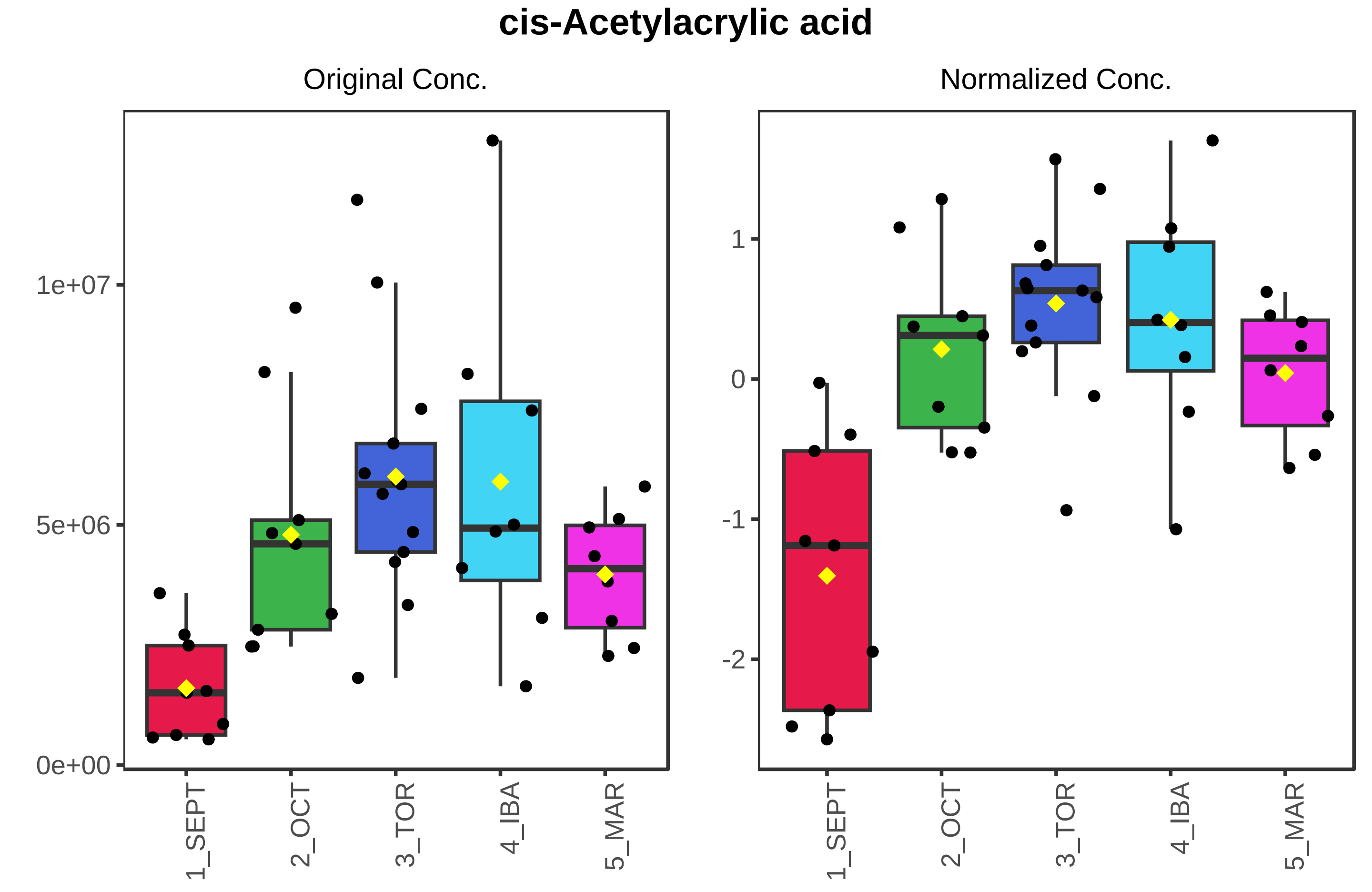
cis-Acetylacrylic acid
Original Conc.
Normalized Conc.
1e+07
5e+06
0e+00
1
0
-1
-2
1_SEPT
2_OCT
3_TOR
4_IBA
5_MAR

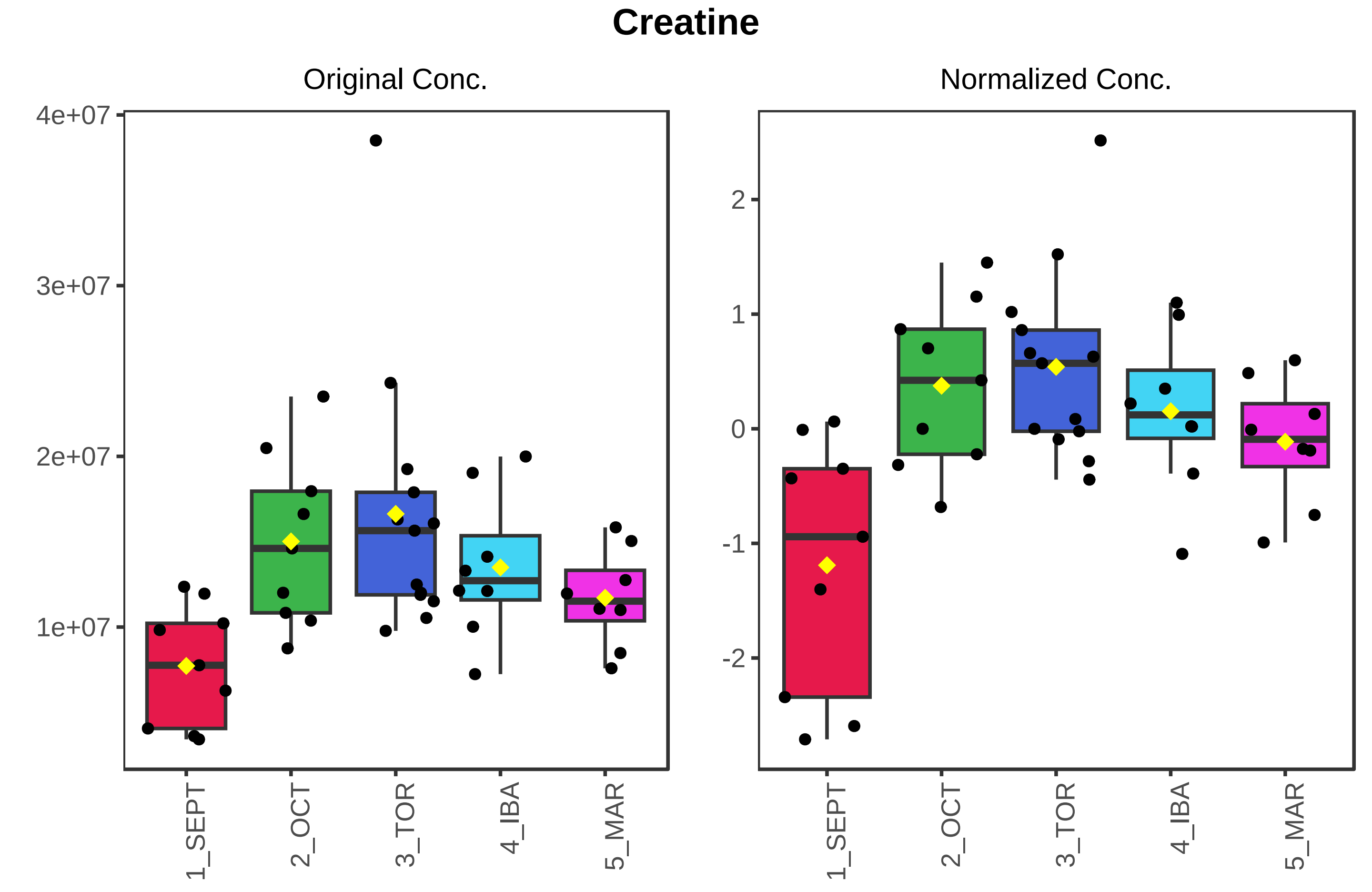
Creatine
Original Conc.
Normalized Conc.
4e+07
3e+07
2e+07
1e+07
2
1
0
-1
-2
1_SEPT
2_OCT
3_TOR
4_IBA
5_MAR

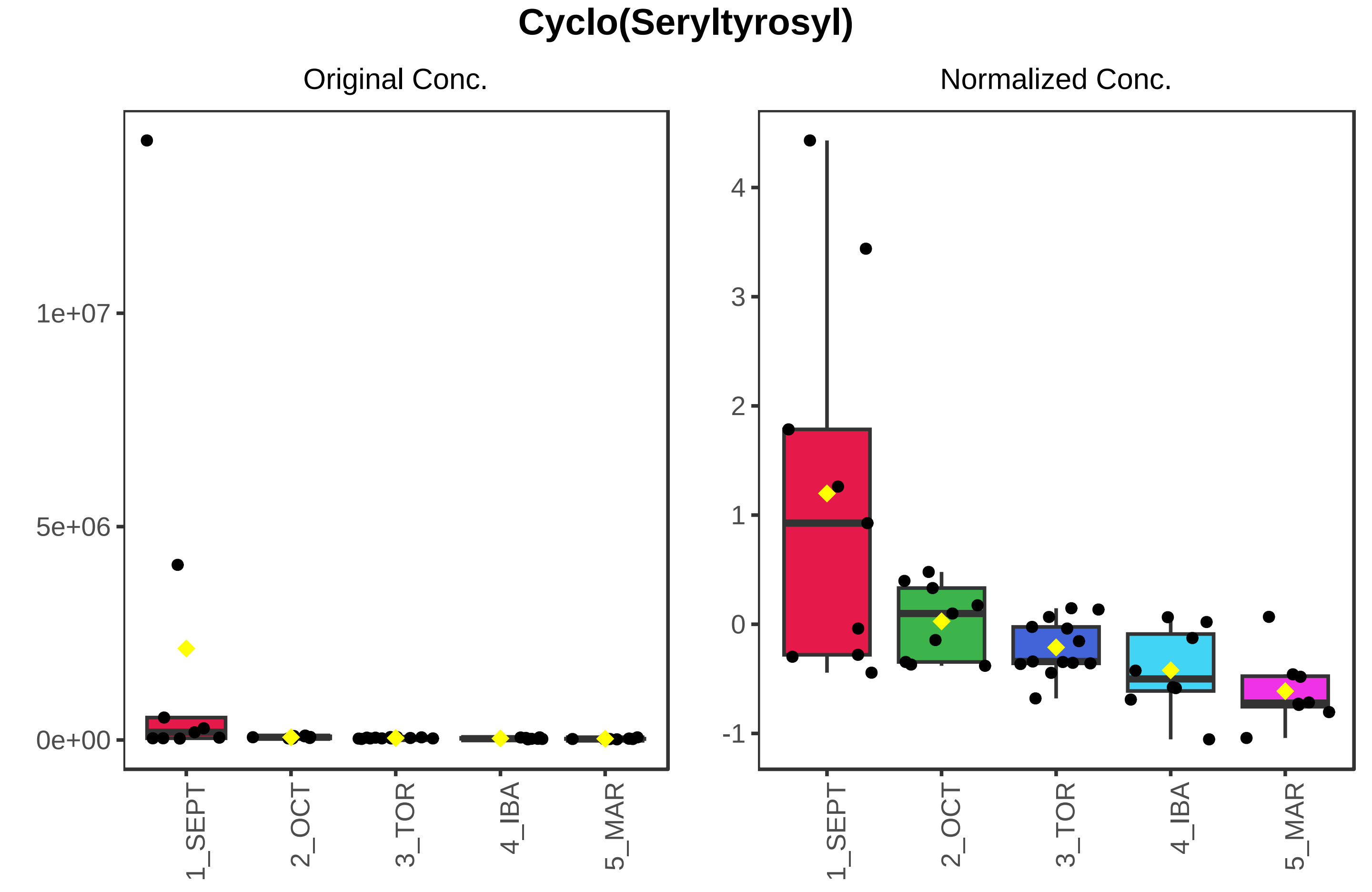

Cyclo(Seryltyrosyl)
Original Conc.
Normalized Conc.
1e+07
5e+06
0e+00
4
3
2
1
0
-1
1_SEPT
2_OCT
3_TOR
4_IBA
5_MAR

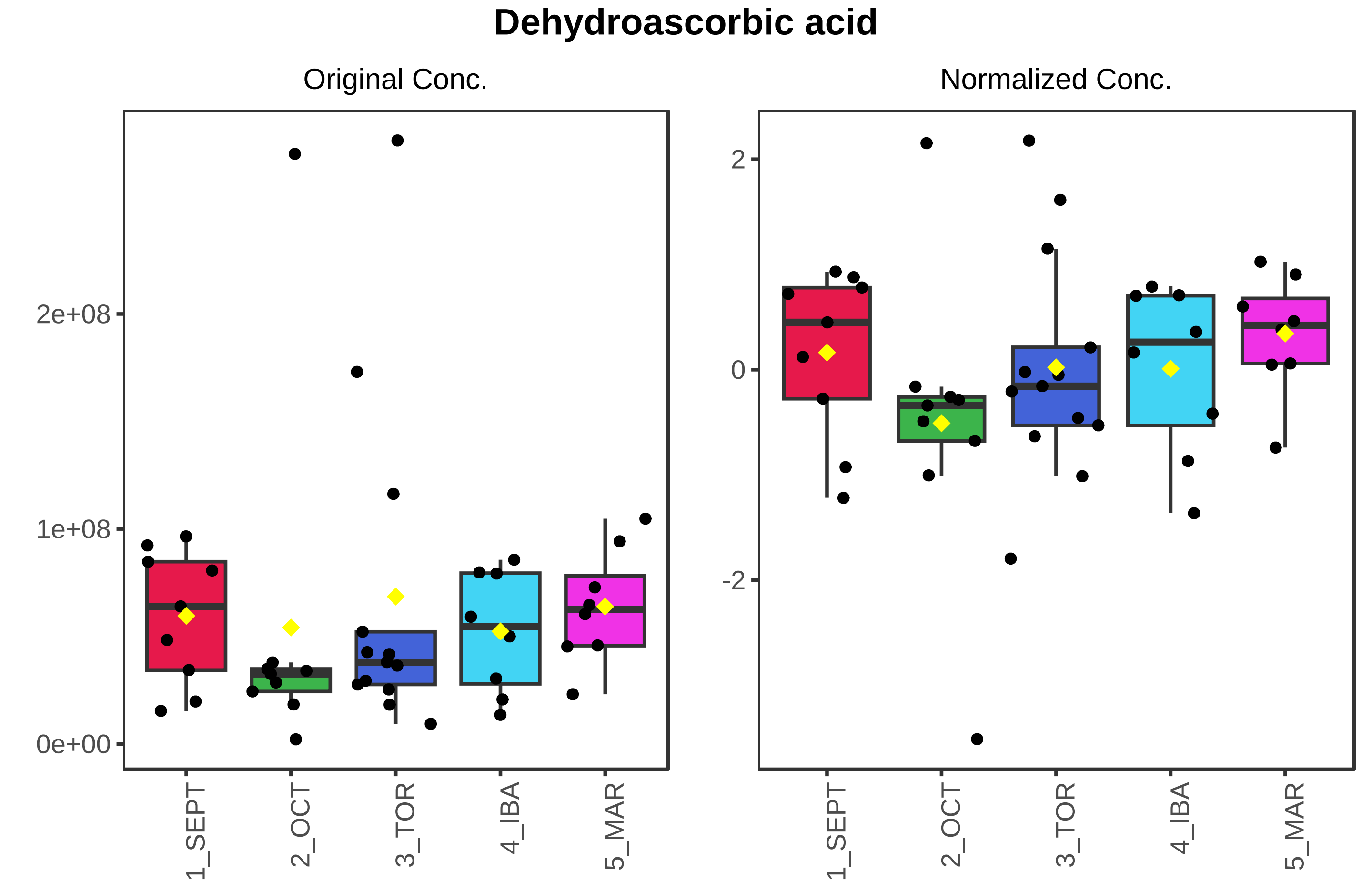
Dehydroascorbic acid
Original Conc.
Normalized Conc.
2e+08
1e+08
0e+00
2
0
-2
1_SEPT
2_OCT
3_TOR
4_IBA
5_MAR

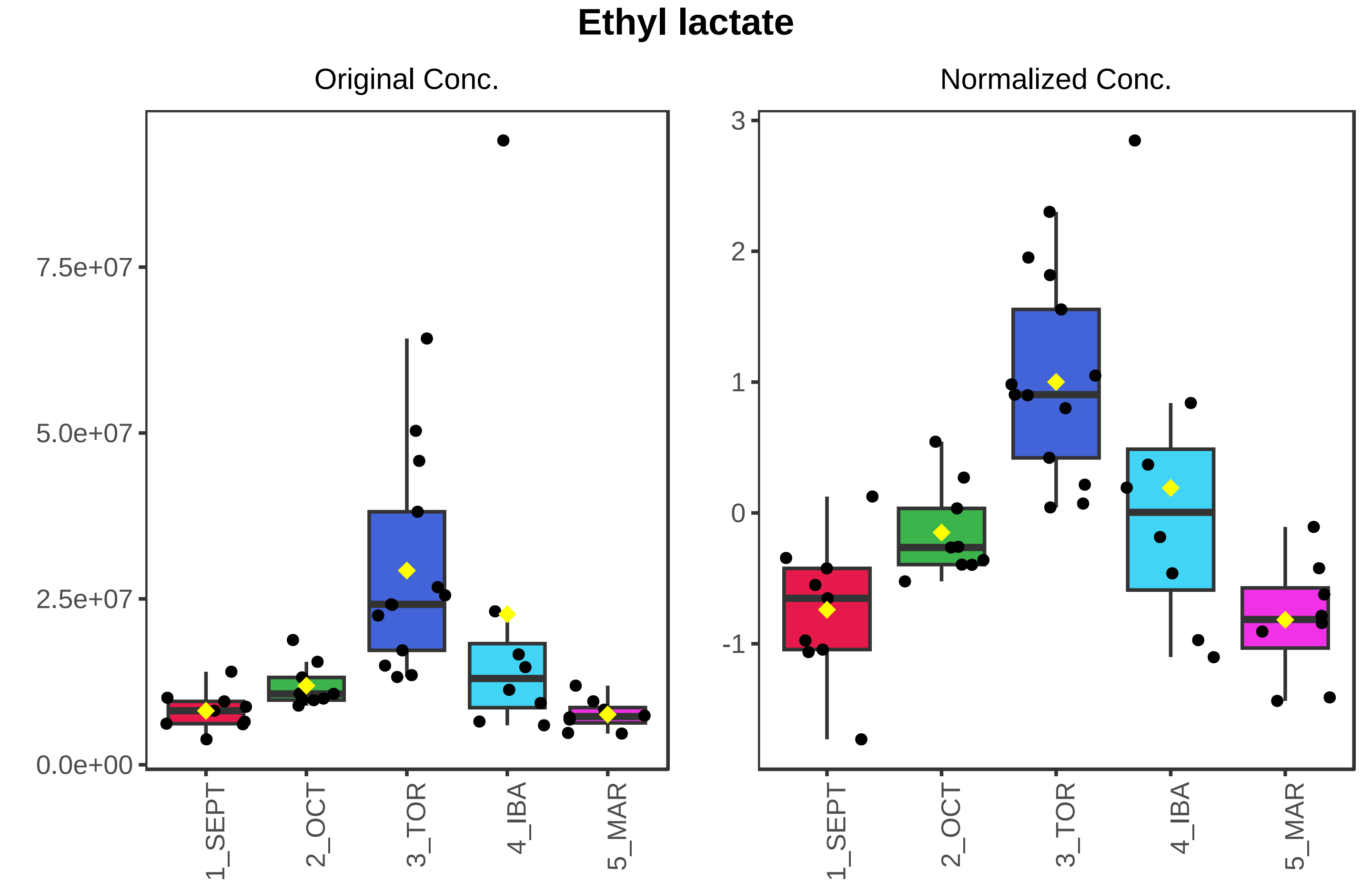
Ethyl lactate
Original Conc.
Normalized Conc.
7.5e+07
5.0e+07
2.5e+07
0.0e+00
3
2
1
0
-1
1_SEPT
2_OCT
3_TOR
4_IBA
5_MAR

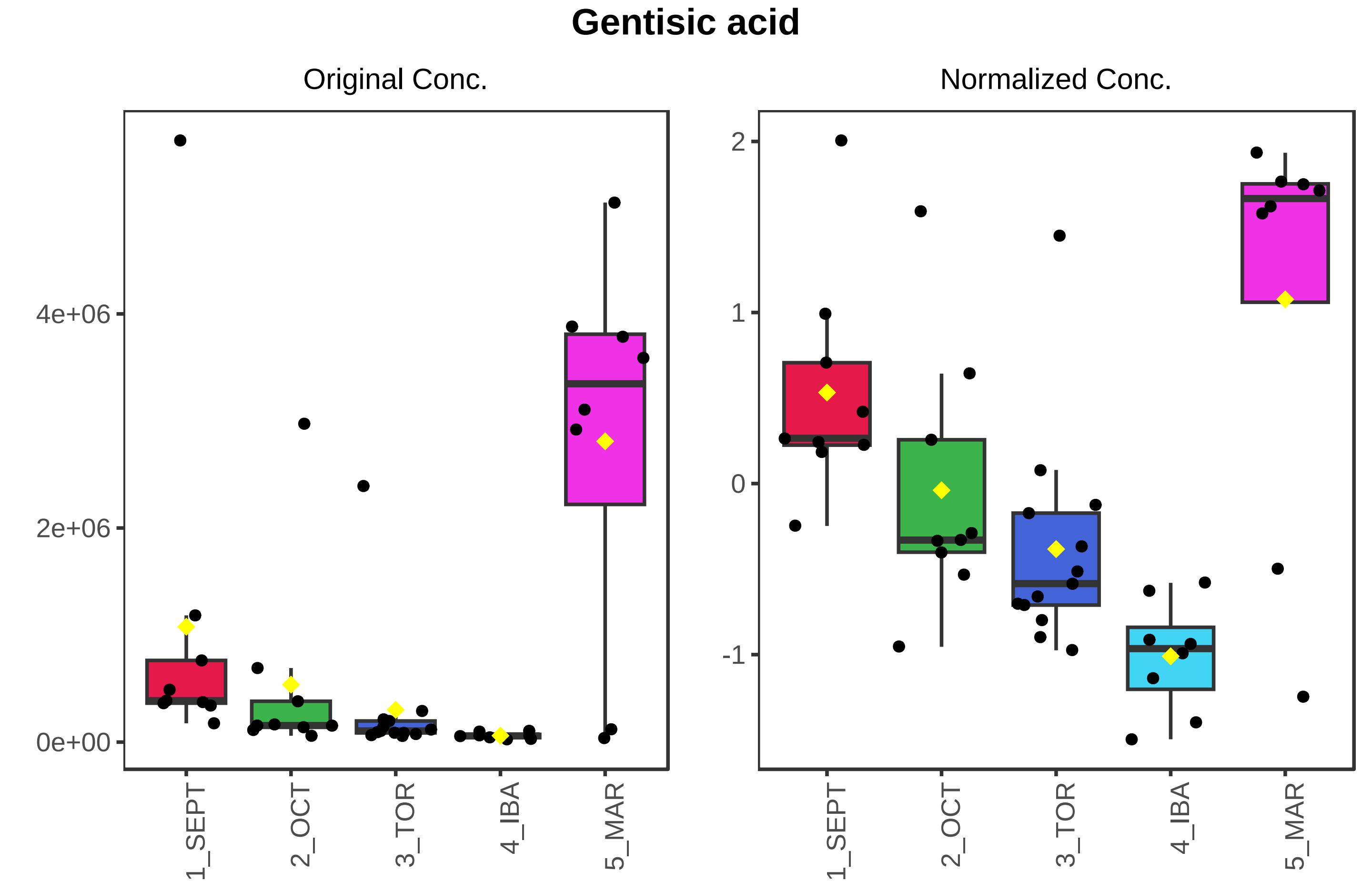

Gentisic acid
Original Conc.
Normalized Conc.
4e+06
2e+06
0e+00
2
1
0
-1
1_SEPT
2_OCT
3_TOR
4_IBA
5_MAR

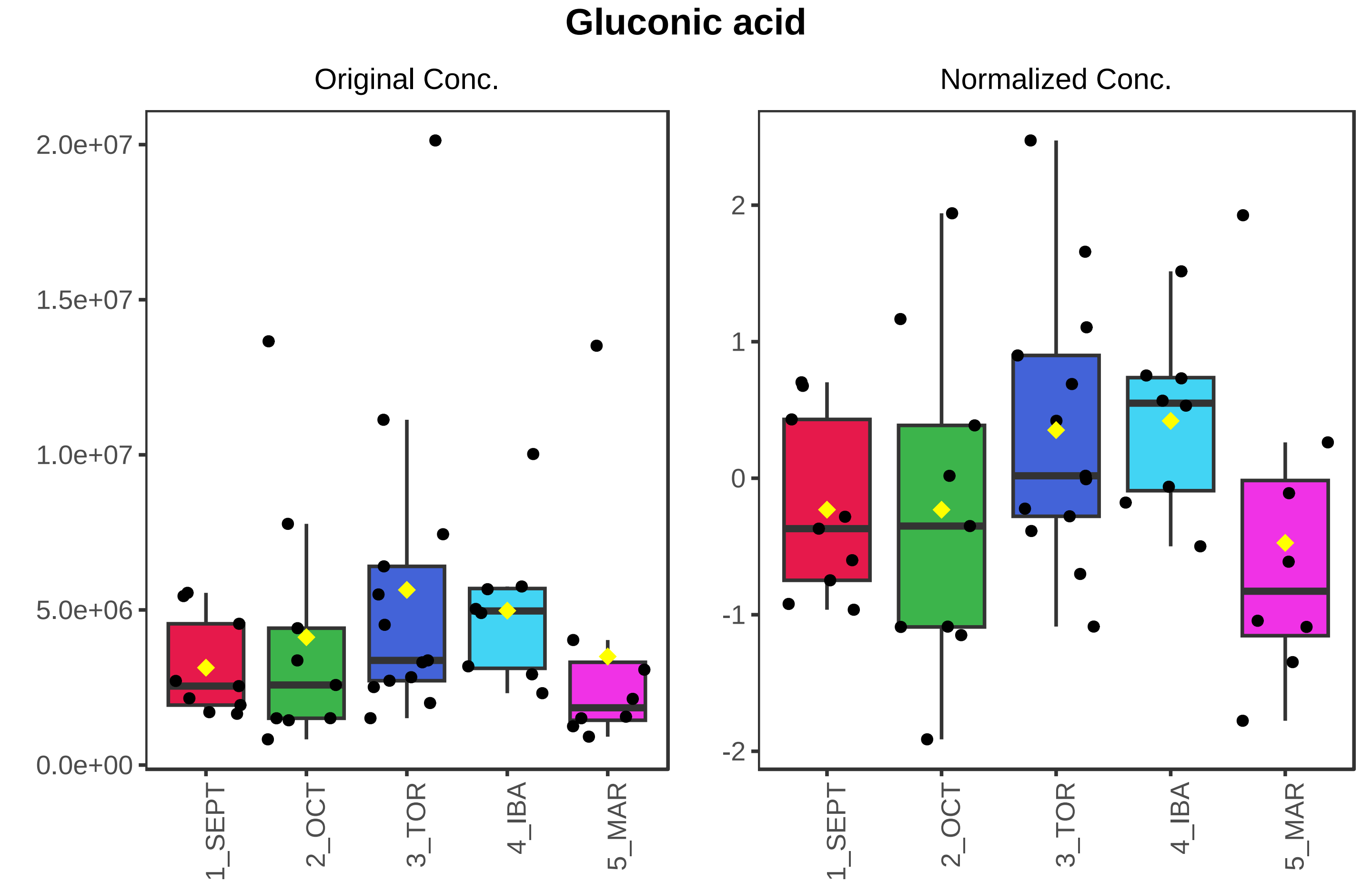
Gluconic acid
Original Conc.
Normalized Conc.
2.0e+07
1.5e+07
1.0e+07
5.0e+06
0.0e+00
2
1
0
-1
-2
1_SEPT
2_OCT
3_TOR
4_IBA
5_MAR

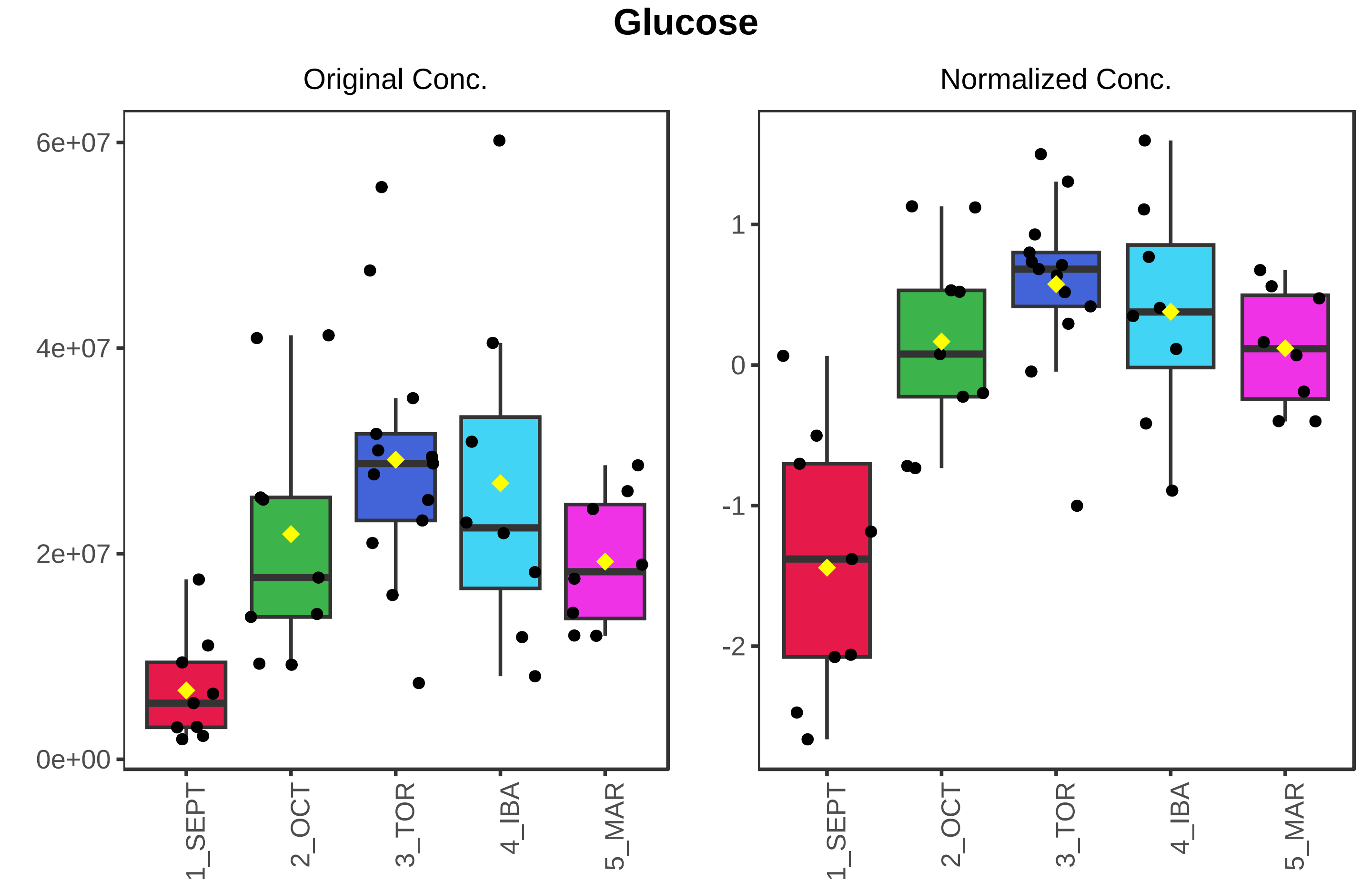
Glucose
Original Conc.
Normalized Conc.
6e+07
4e+07
2e+07
0e+00
1
0
-1
-2
1_SEPT
2_OCT
3_TOR
4_IBA
5_MAR

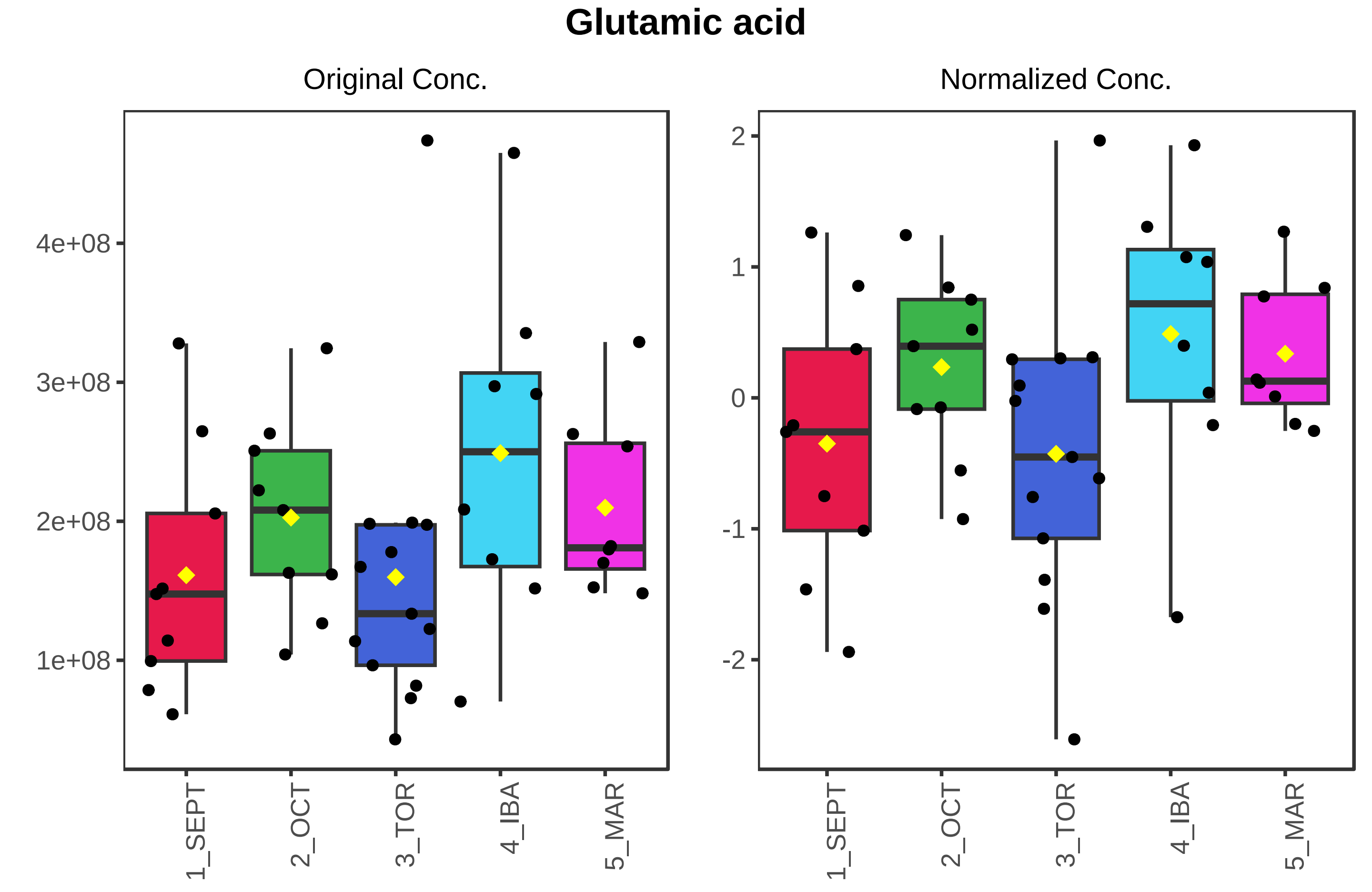

Glutamic acid
Original Conc.
Normalized Conc.
4e+08
3e+08
2e+08
1e+08
2
1
0
-1
-2
1_SEPT
2_OCT
3_TOR
4_IBA
5_MAR

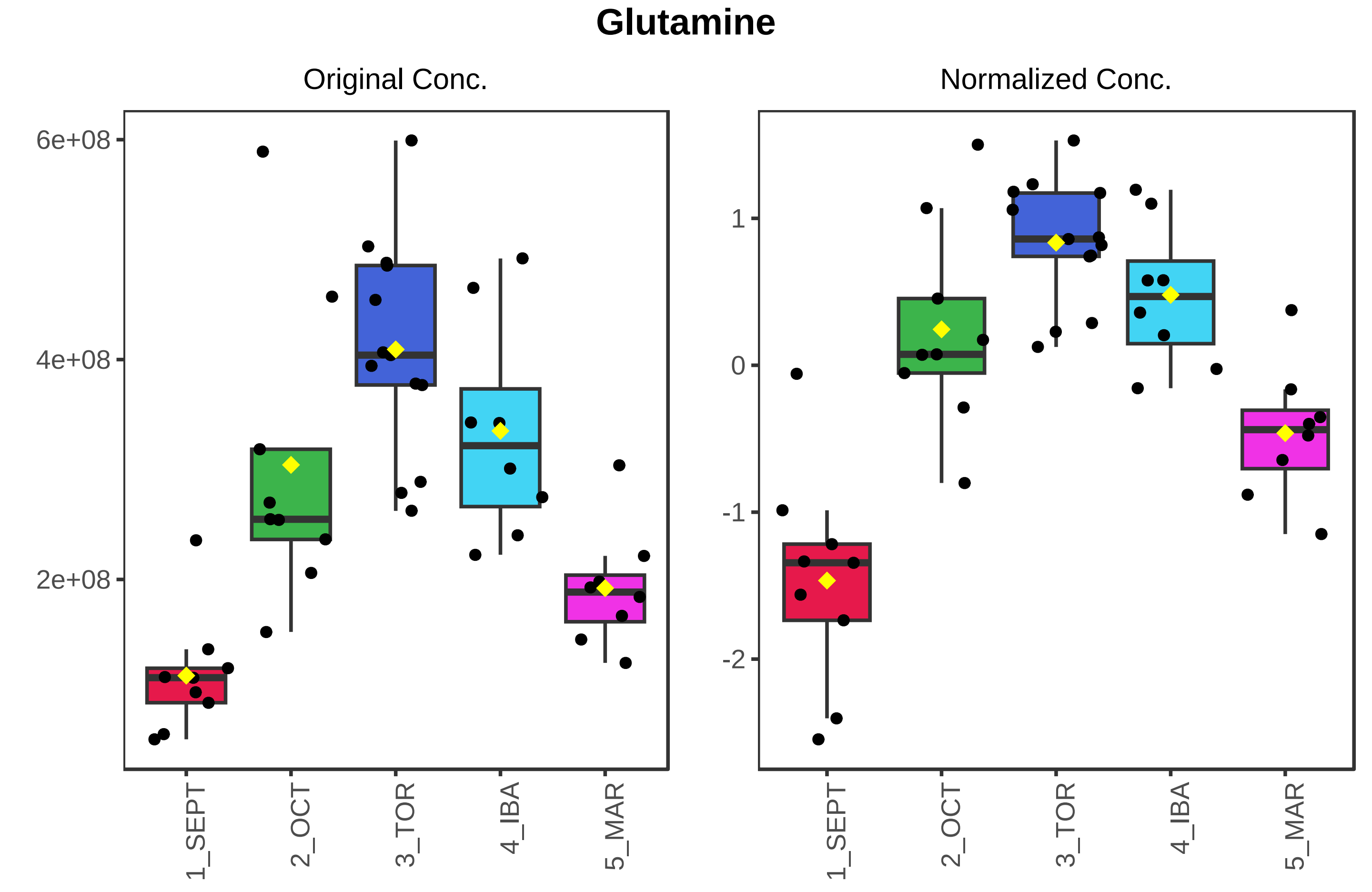

Glutamine
Original Conc.
Normalized Conc.
6e+08
4e+08
2e+08
1
0
-1
-2
1_SEPT
2_OCT
3_TOR
4_IBA
5_MAR

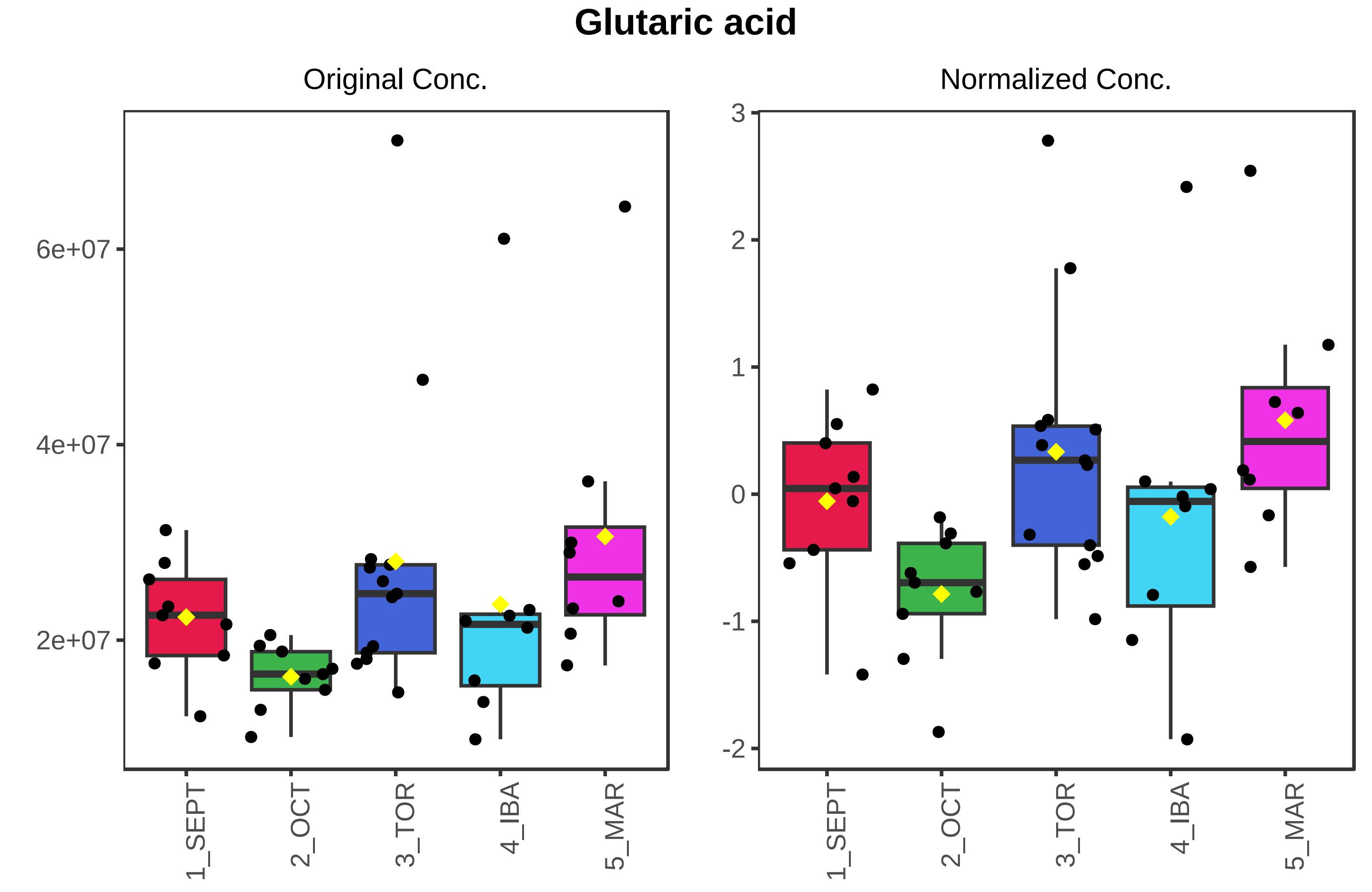

Glutaric acid
Original Conc.
Normalized Conc.
6e+07
4e+07
2e+07
3
2
1
0
-1
-2
1_SEPT
2_OCT
3_TOR
4_IBA
5_MAR

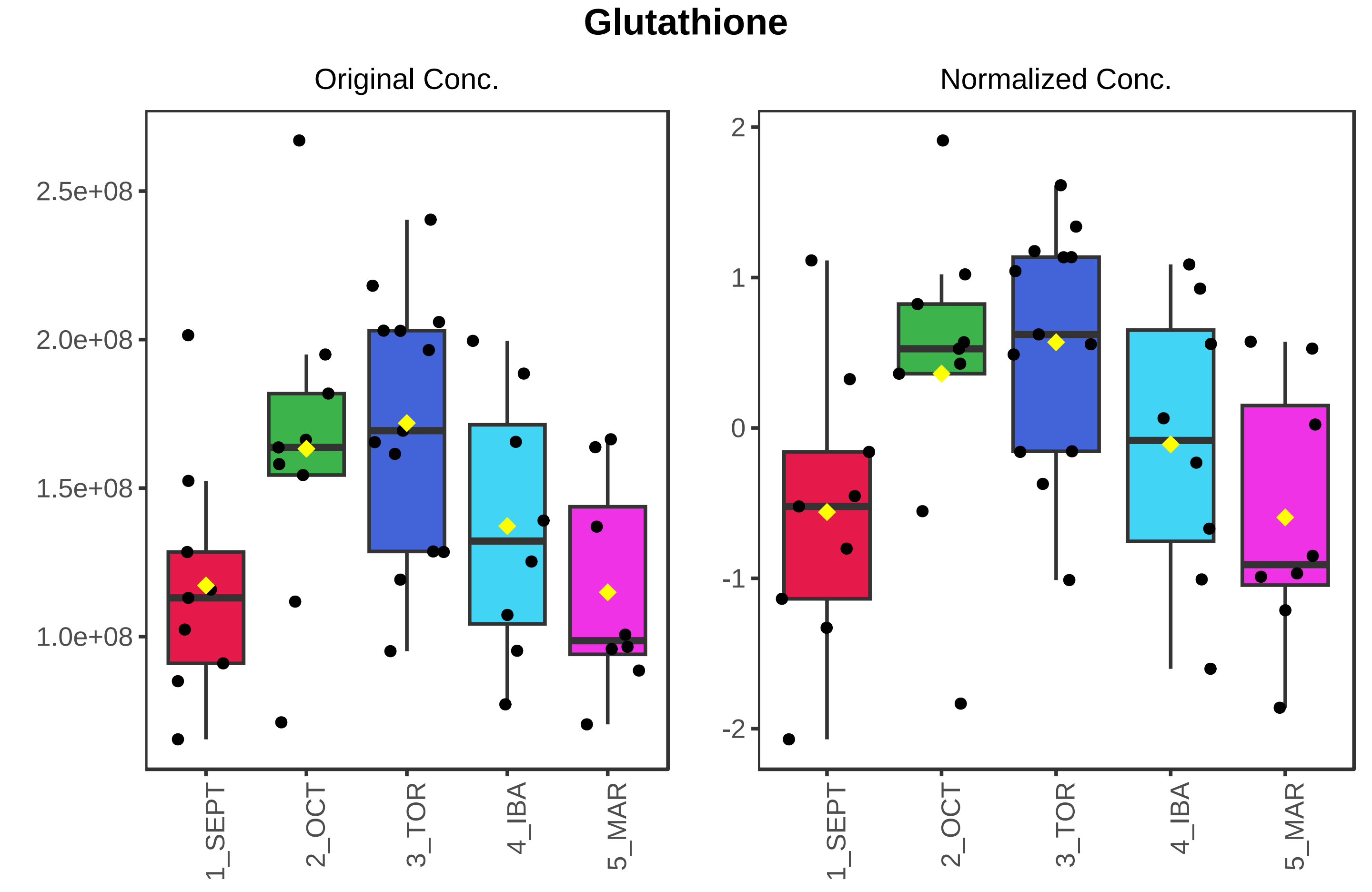

Glutathione
Original Conc.
Normalized Conc.
2.5e+08
2.0e+08
1.5e+08
1.0e+08
2
1
0
-1
-2
1_SEPT
2_OCT
3_TOR
4_IBA
5_MAR

Glycerol 3-phosphate

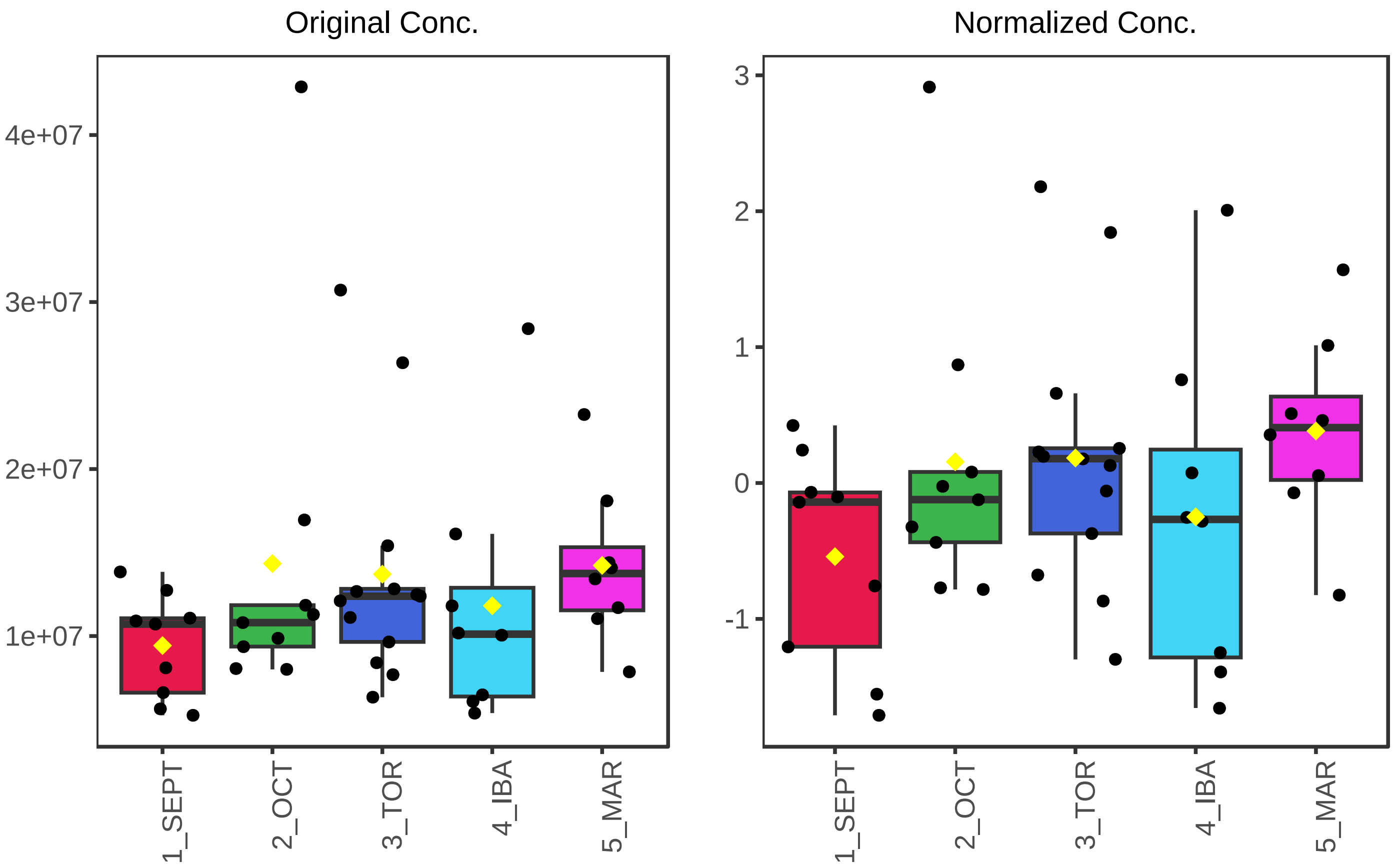

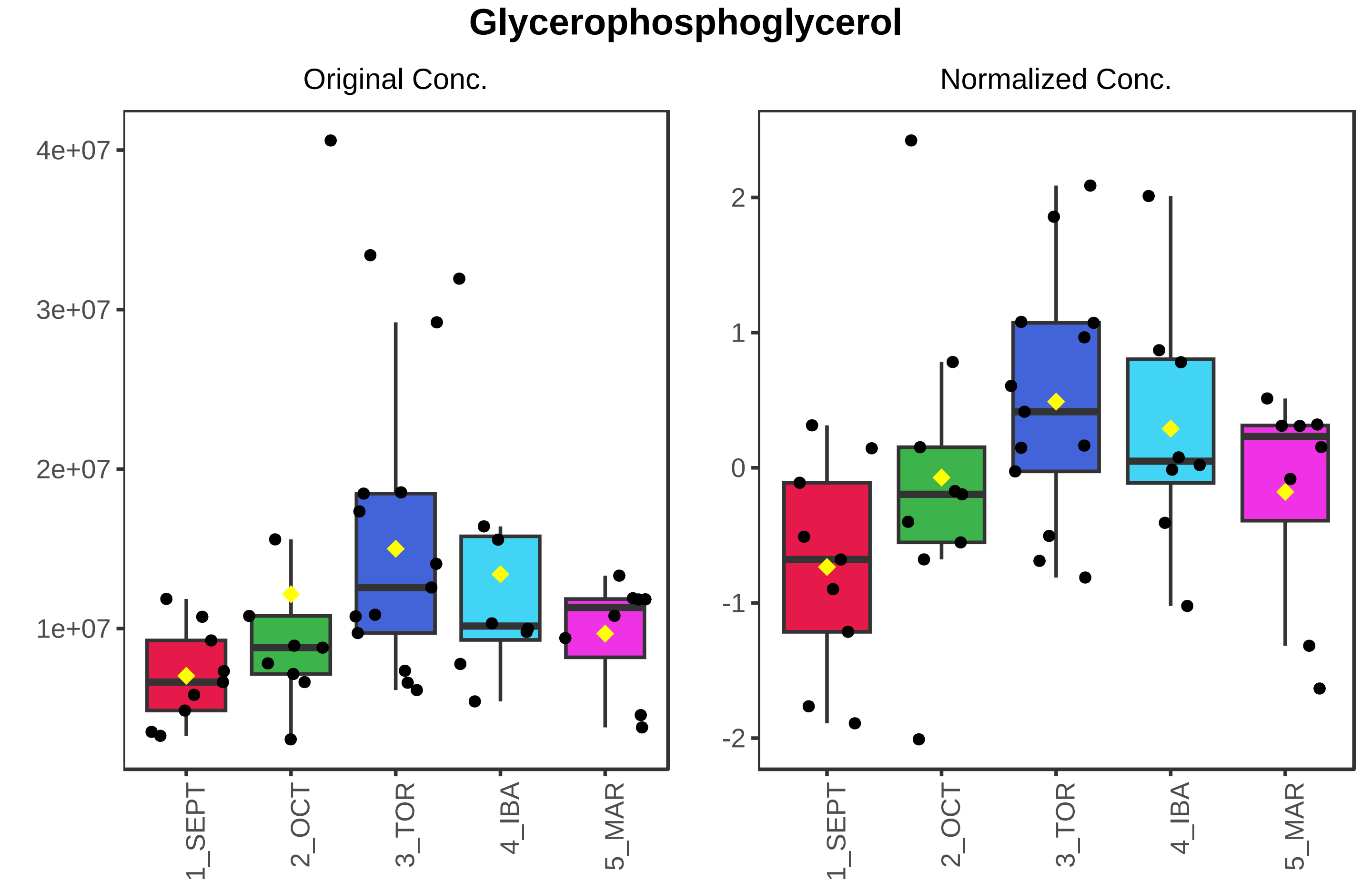
Glycerophosphoglycerol
Original Conc.
Normalized Conc.
4e+07
3e+07
2e+07
1e+07
2
1
0
-1
-2
1_SEPT
2_OCT
3_TOR
4_IBA
5_MAR

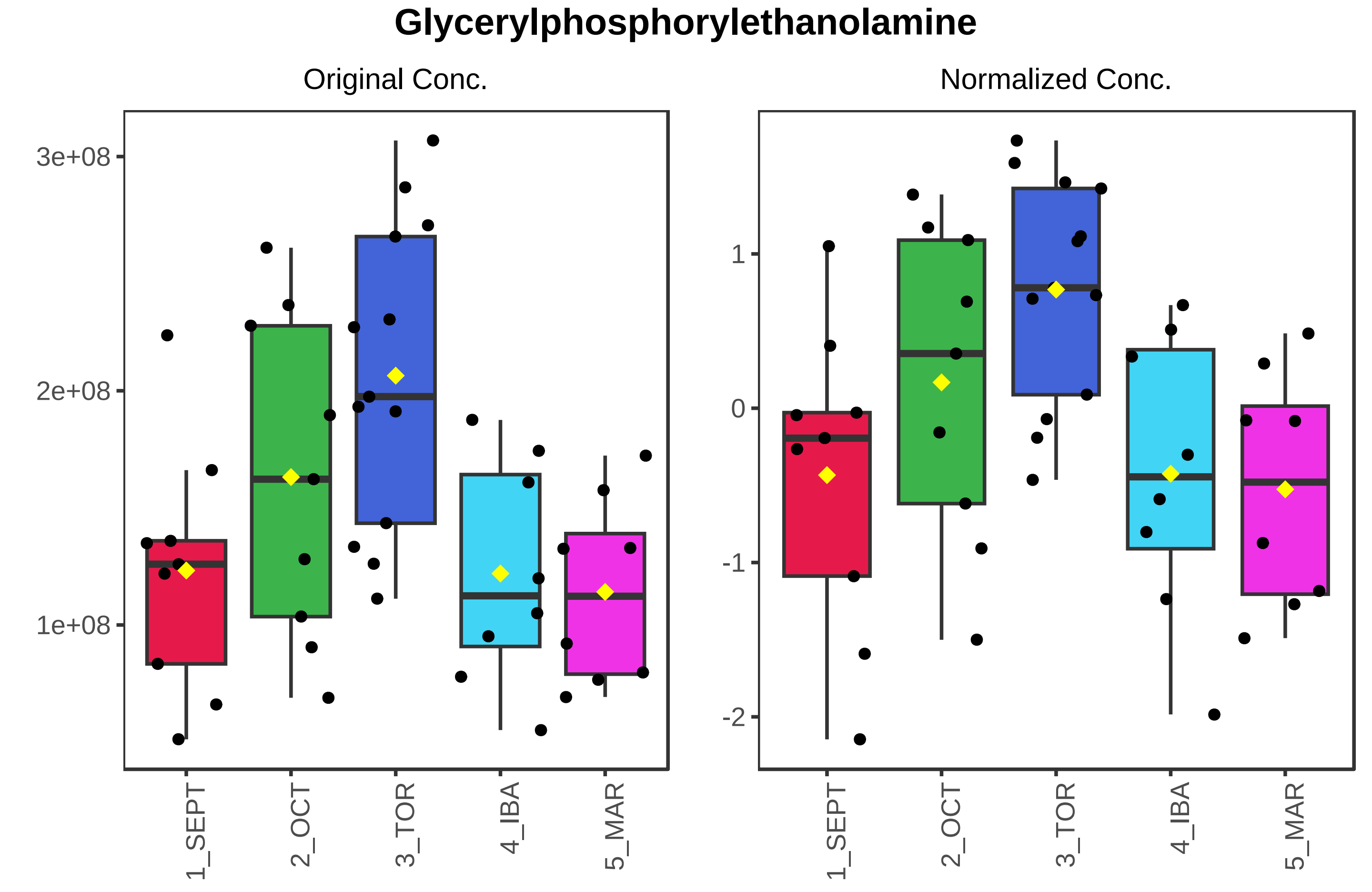
Glycerylphosphorylethanolamine
Original Conc.
Normalized Conc.
3e+08
2e+08
1e+08
1
0
-1
-2
1_SEPT
2_OCT
3_TOR
4_IBA
5_MAR

Glycine

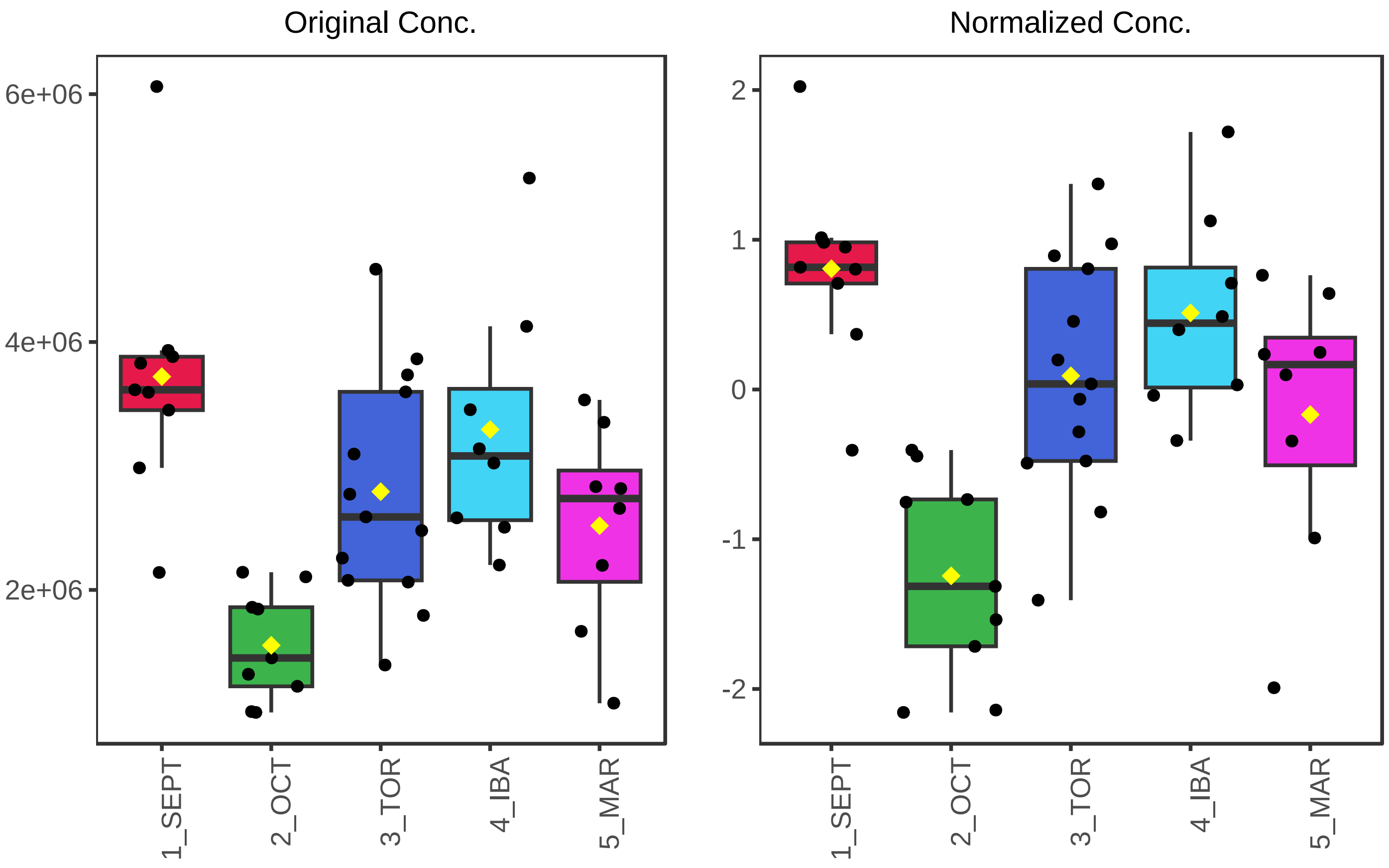

Original Conc.
Normalized Conc.
6e+06
4e+06
2e+06
2
1
0
-1
-2
1_SEPT
2_OCT
3_TOR
4_IBA
5_MAR

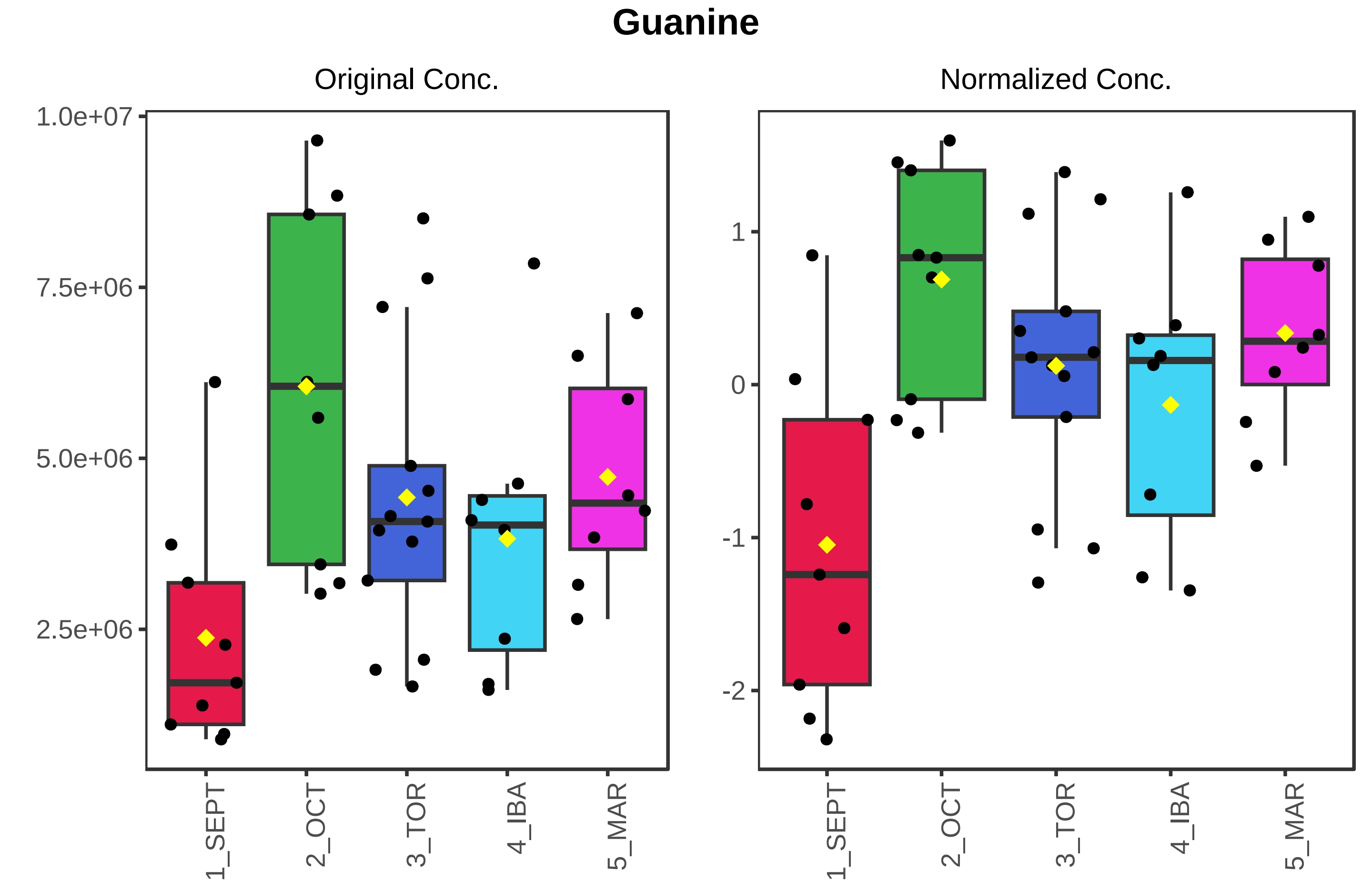
Guanine
Original Conc.
Normalized Conc.
1.0e+07
7.5e+06
5.0e+06
2.5e+06
1
0
-1
-2
1_SEPT
2_OCT
3_TOR
4_IBA
5_MAR

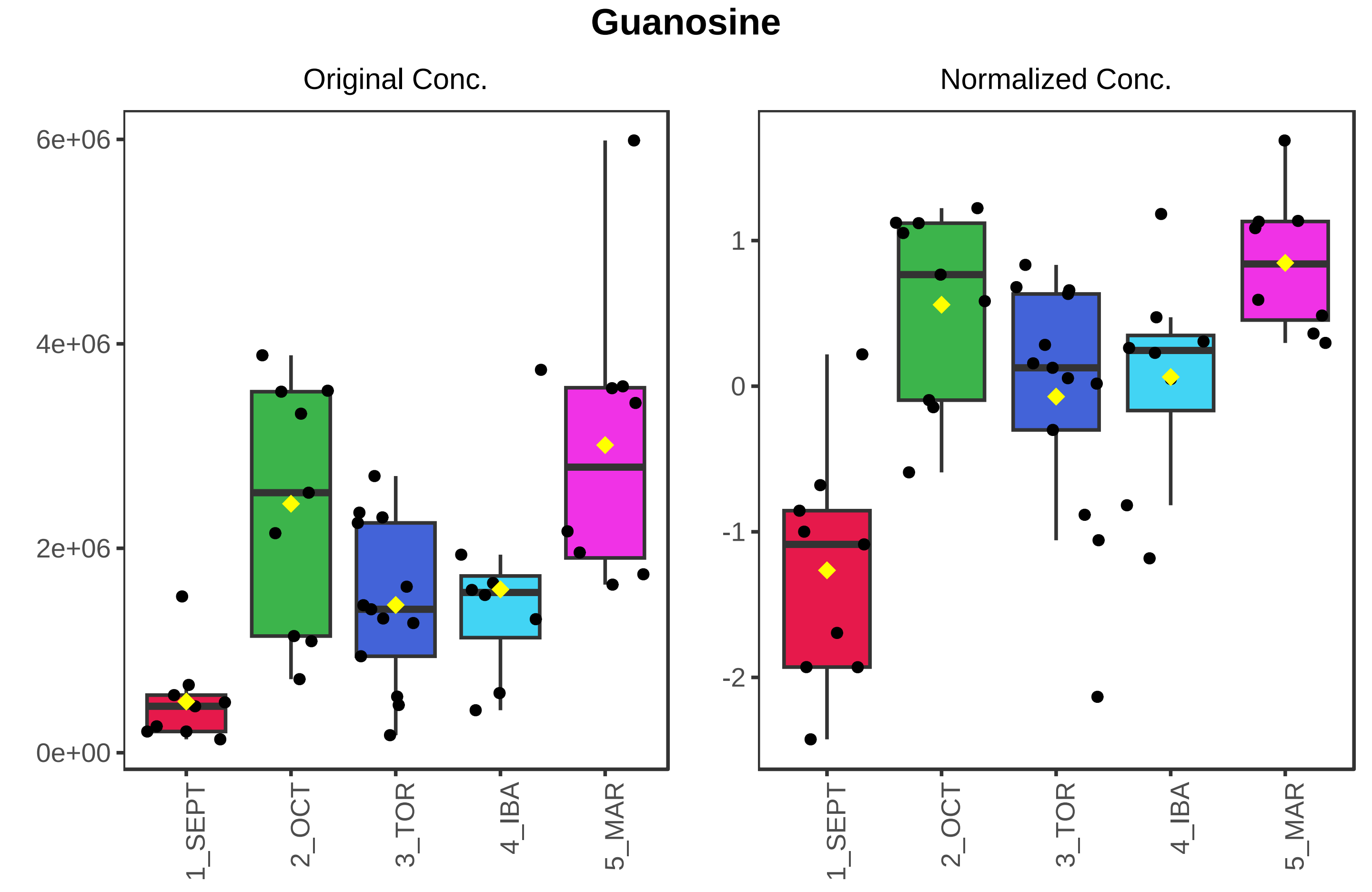

Guanosine
Original Conc.
Normalized Conc.
6e+06
4e+06
2e+06
0e+00
1
0
-1
-2
1_SEPT
2_OCT
3_TOR
4_IBA
5_MAR

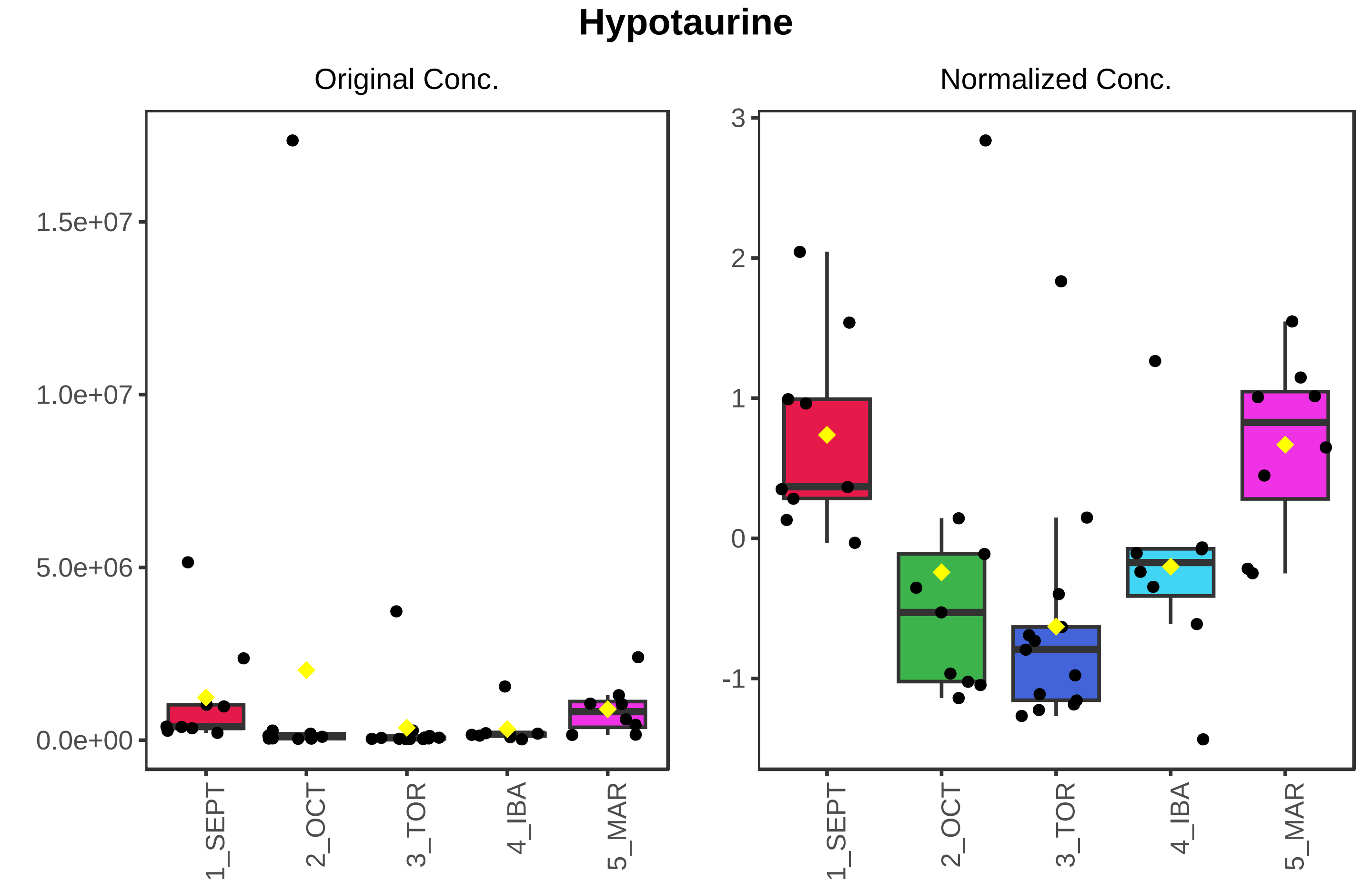

Hypotaurine
Original Conc.
Normalized Conc.
1.5e+07
1.0e+07
5.0e+06
0.0e+00
3
2
1
0
-1
1_SEPT
2_OCT
3_TOR
4_IBA
5_MAR

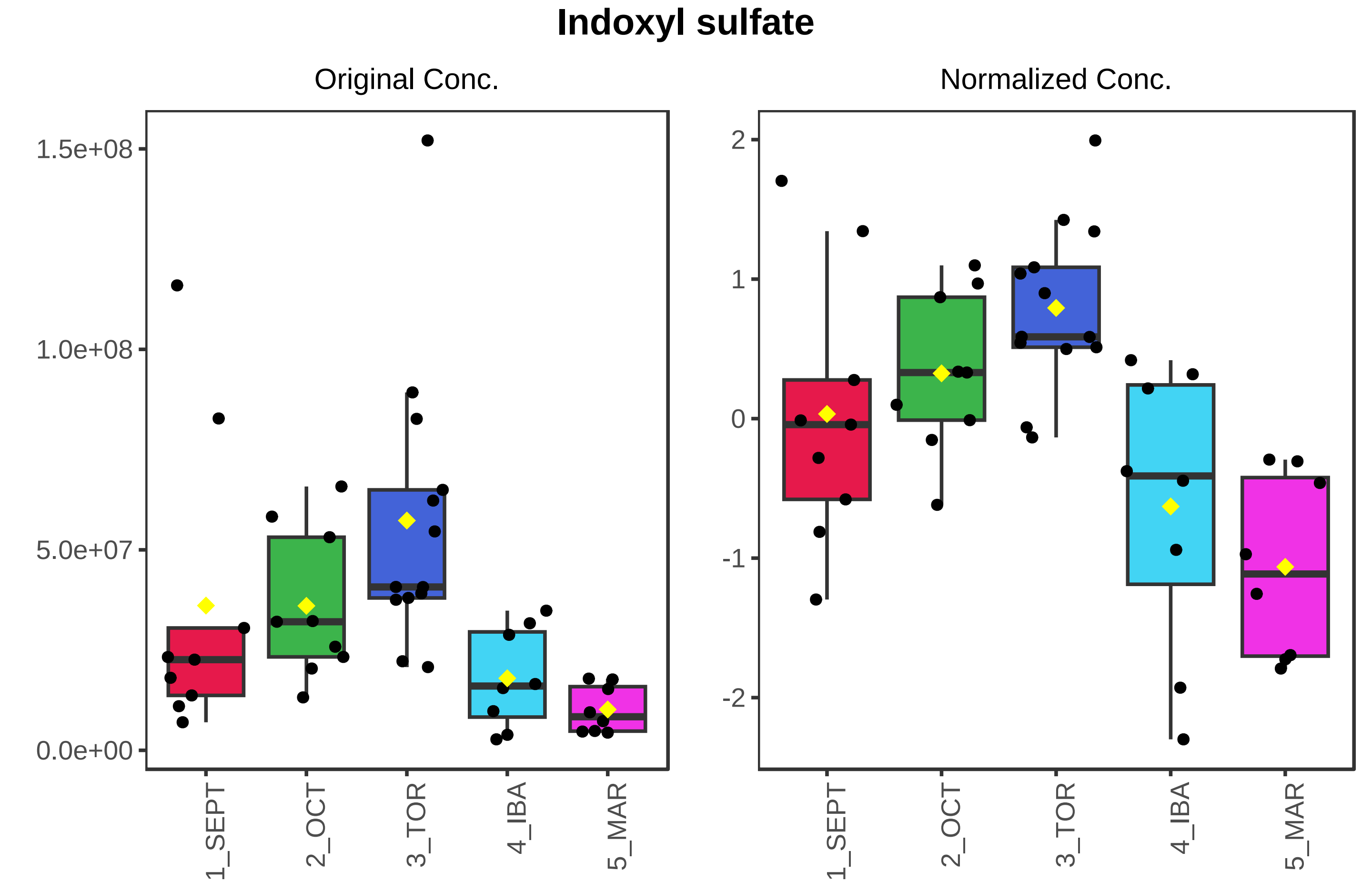

Indoxyl sulfate
Original Conc.
Normalized Conc.
1.5e+08
1.0e+08
5.0e+07
0.0e+00
2
1
0
-1
-2
1_SEPT
2_OCT
3_TOR
4_IBA
5_MAR

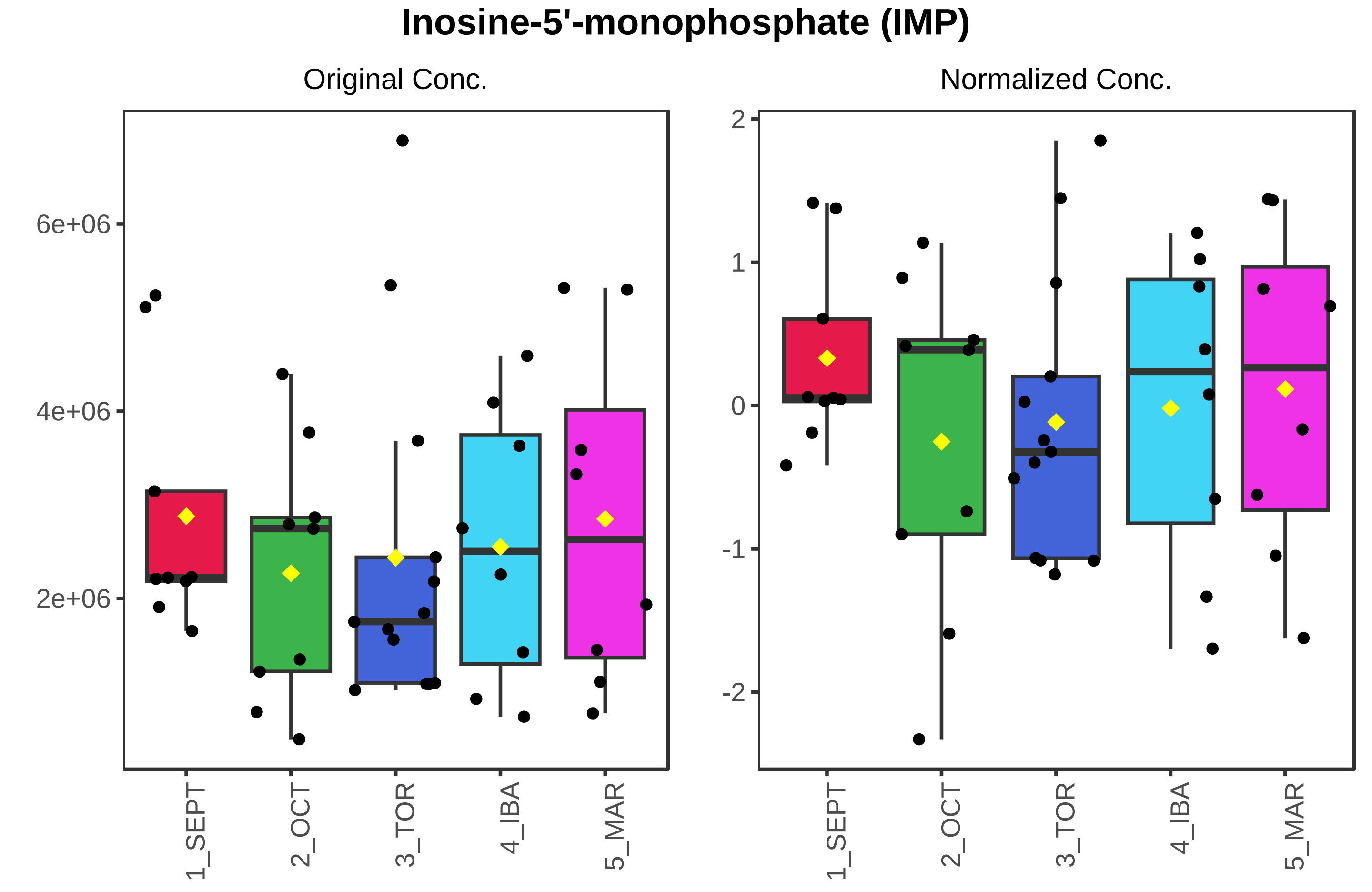
Inosine-5'-monophosphate (IMP)
Original Conc.
Normalized Conc.
6e+06
4e+06
2e+06
2
1
0
-1
-2
1_SEPT
2_OCT
3_TOR
4_IBA
5_MAR

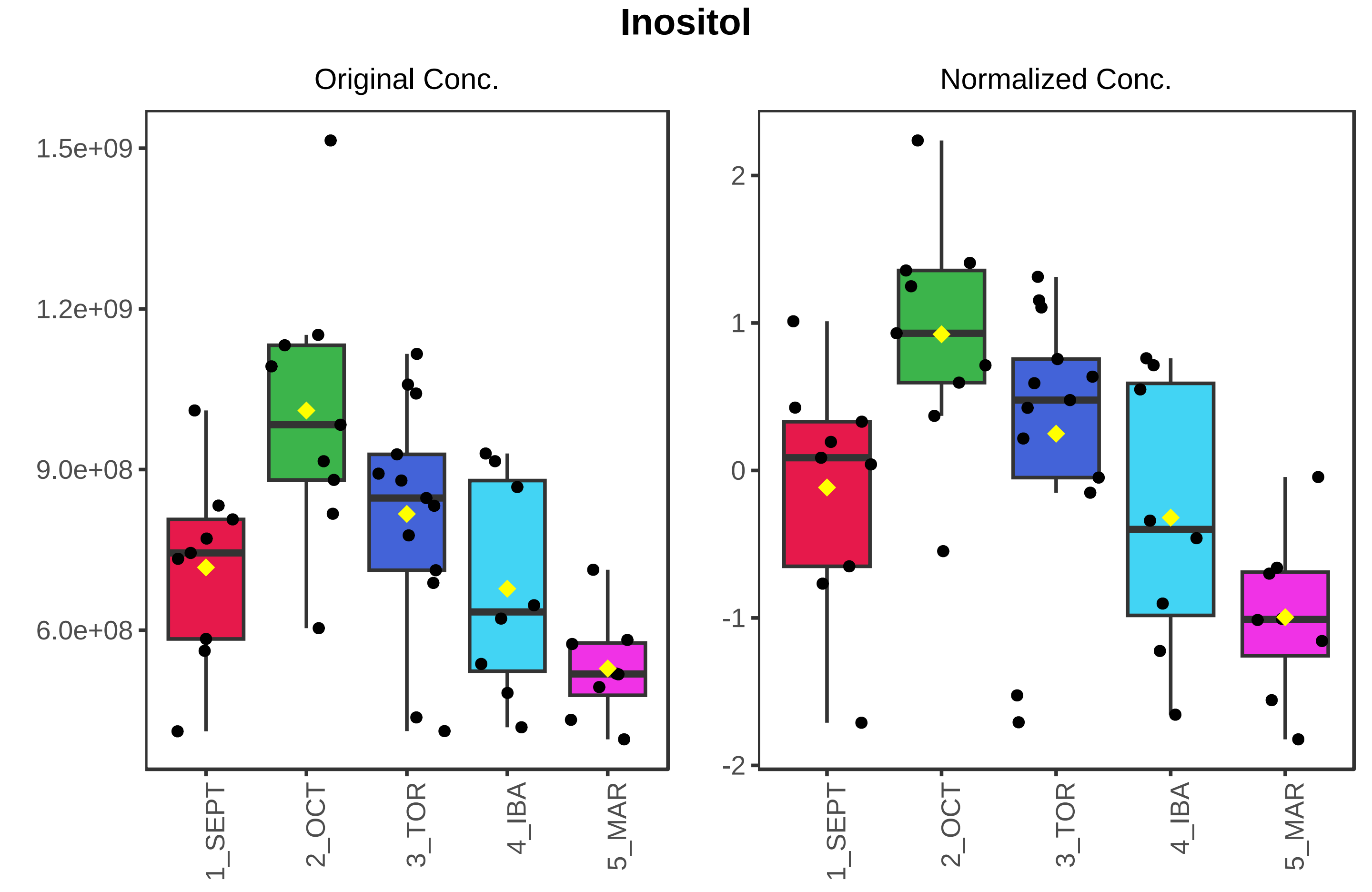
Inositol
Original Conc.
Normalized Conc.
1.5e+09
1.2e+09
9.0e+08
6.0e+08
2
1
0
-1
-2
1_SEPT
2_OCT
3_TOR
4_IBA
5_MAR

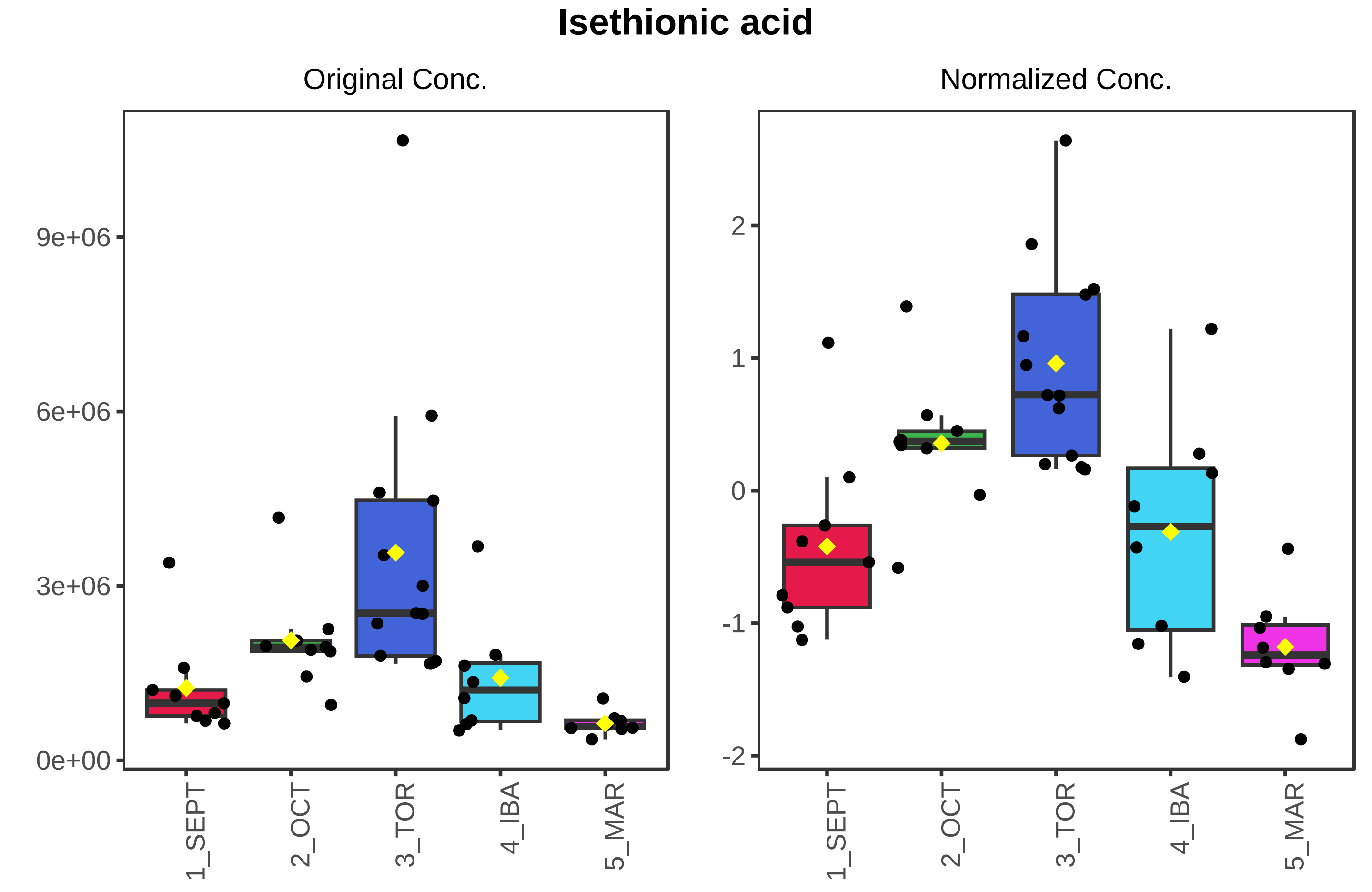

Isethionic acid
Original Conc.
Normalized Conc.
9e+06
6e+06
3e+06
0e+00
2
1
0
-1
-2
1_SEPT
2_OCT
3_TOR
4_IBA
5_MAR

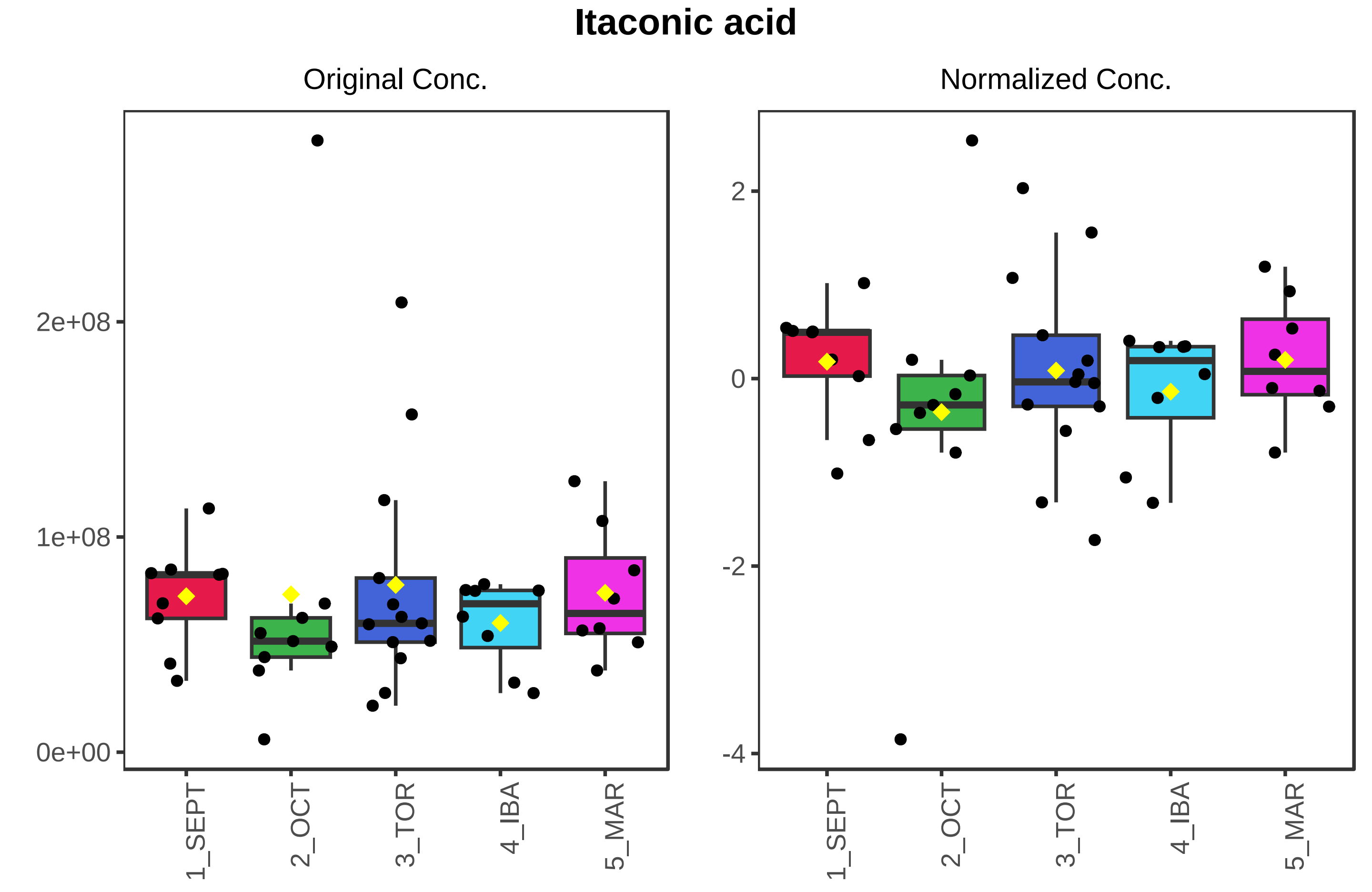

Itaconic acid
Original Conc.
Normalized Conc.
2e+08
1e+08
0e+00
2
0
-2
-4
1_SEPT
2_OCT
3_TOR
4_IBA
5_MAR

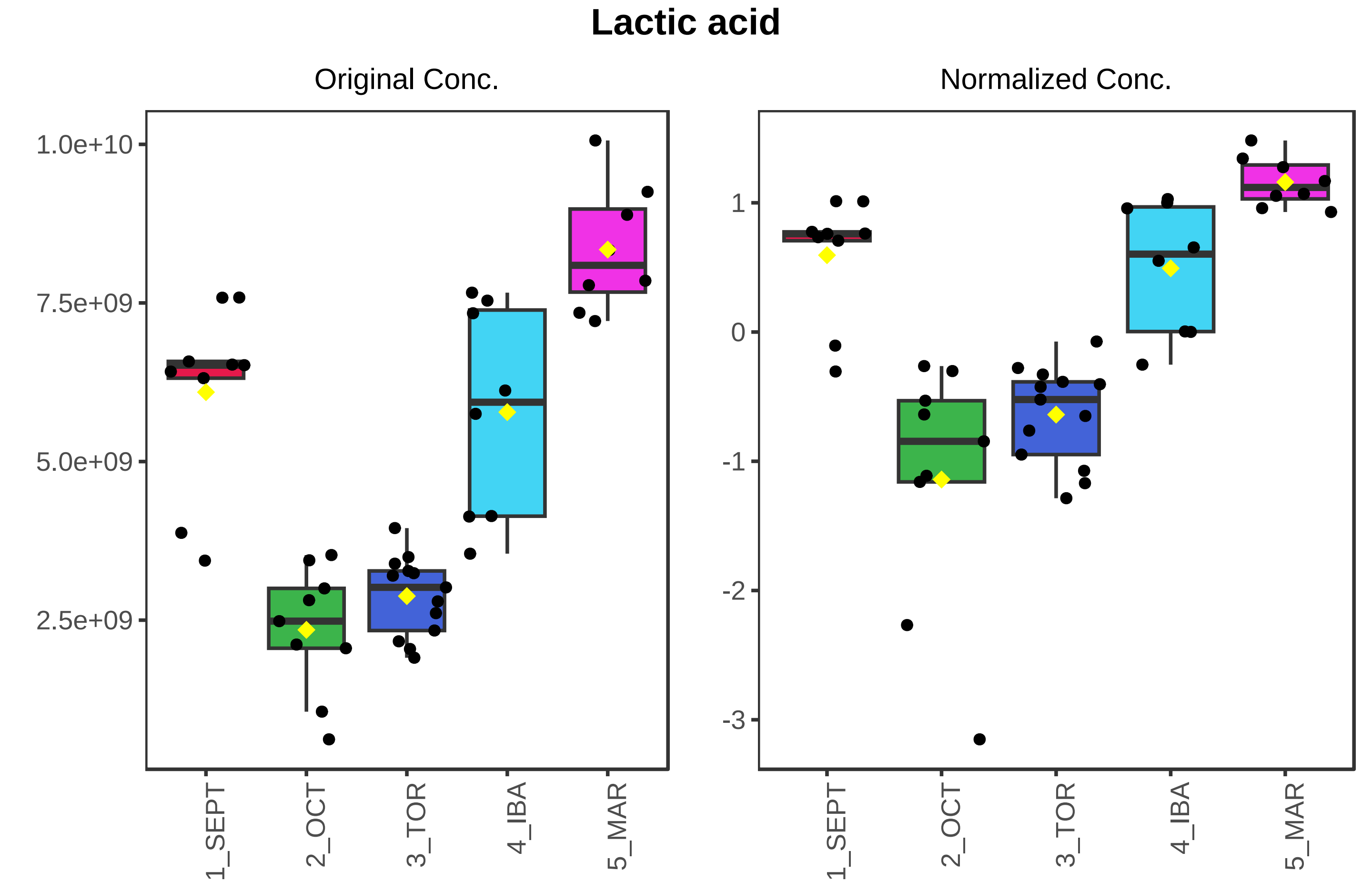

Lactic acid
Original Conc.
Normalized Conc.
1.0e+10
7.5e+09
5.0e+09
2.5e+09
1
0
-1
-2
-3
1_SEPT
2_OCT
3_TOR
4_IBA
5_MAR

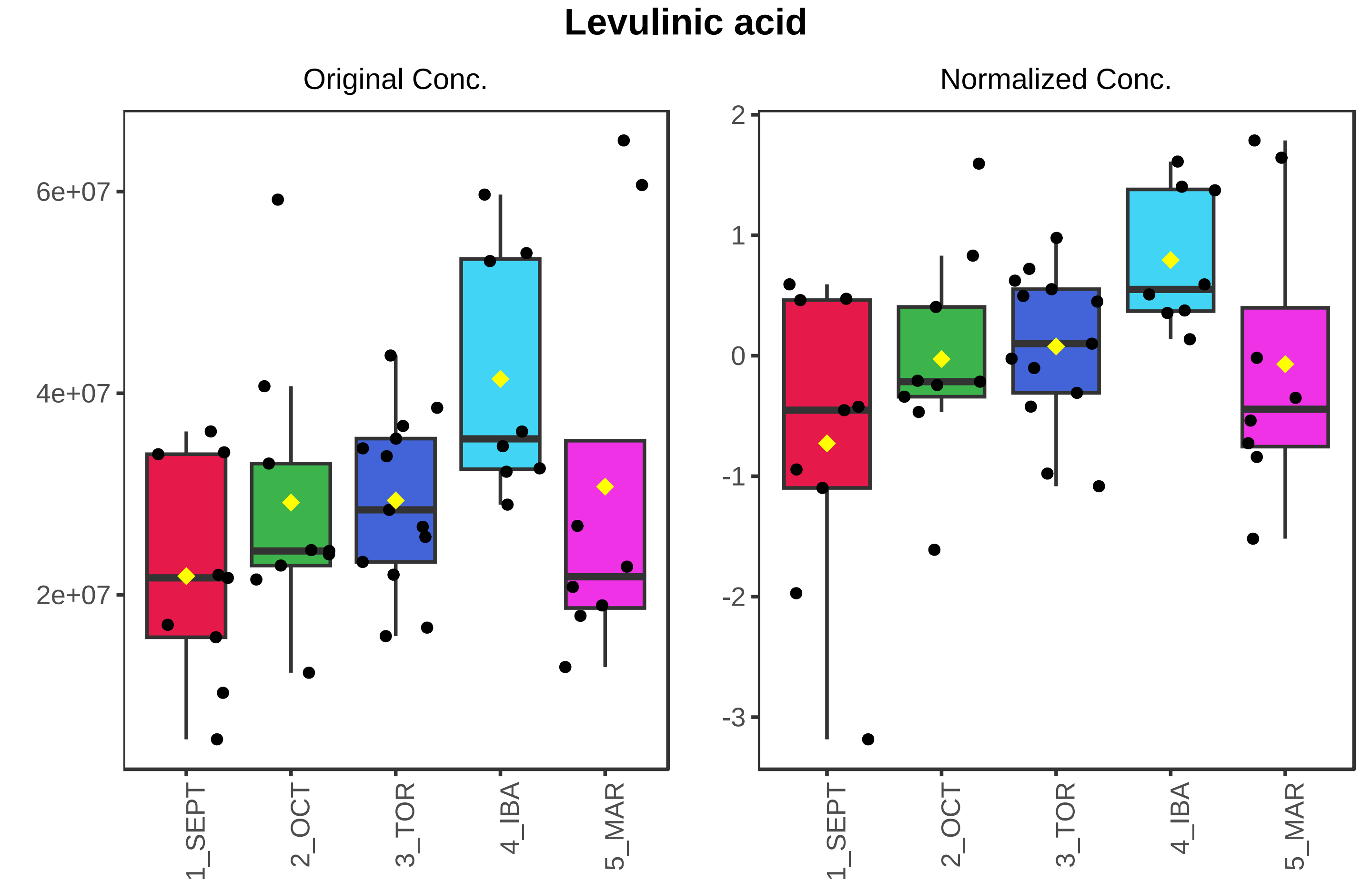

Levulinic acid
Original Conc.
Normalized Conc.
6e+07
4e+07
2e+07
2
1
0
-1
-2
-3
1_SEPT
2_OCT
3_TOR
4_IBA
5_MAR
1_SEPT
2_OCT
3_TOR
4_IBA
5_MAR

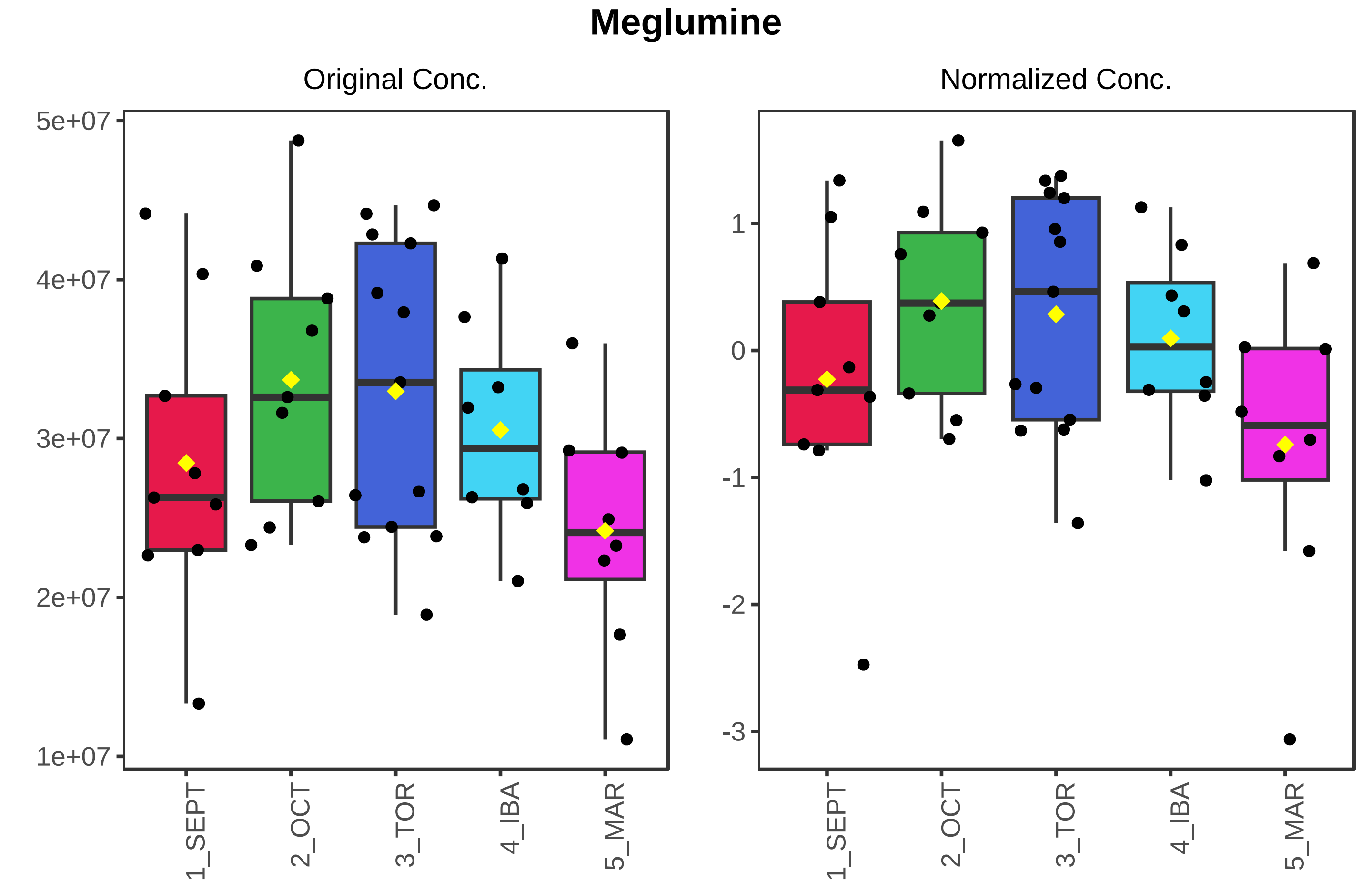

Meglumine
Original Conc.
Normalized Conc.
5e+07
4e+07
3e+07
2e+07
1e+07
1
0
-1
-2
-3
1_SEPT
2_OCT
3_TOR
4_IBA
5_MAR

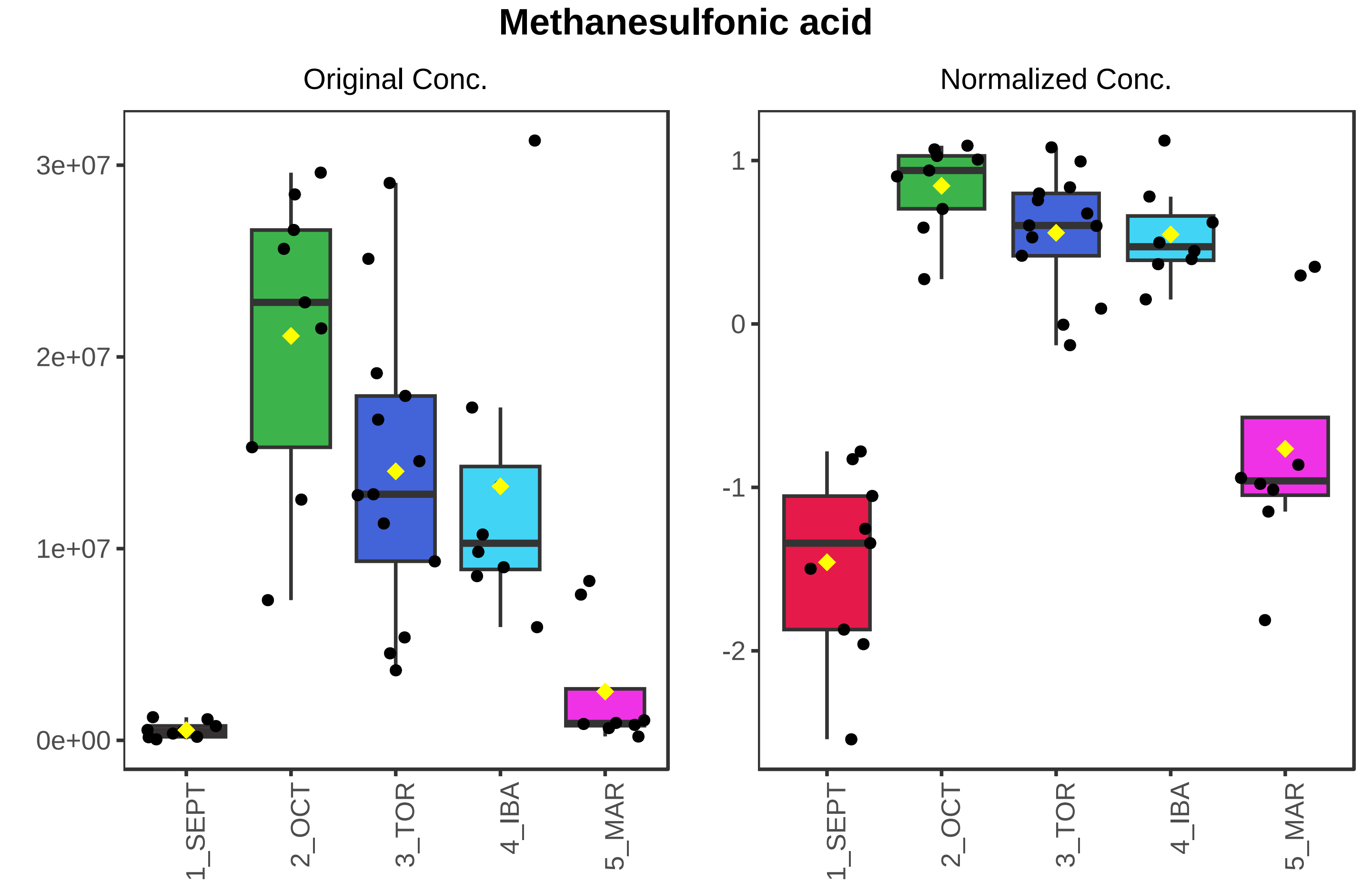

Methanesulfonic acid
Original Conc.
Normalized Conc.
3e+07
2e+07
1e+07
0e+00
1
0
-1
-2
1_SEPT
2_OCT
3_TOR
4_IBA
5_MAR

Methylmalonic acid

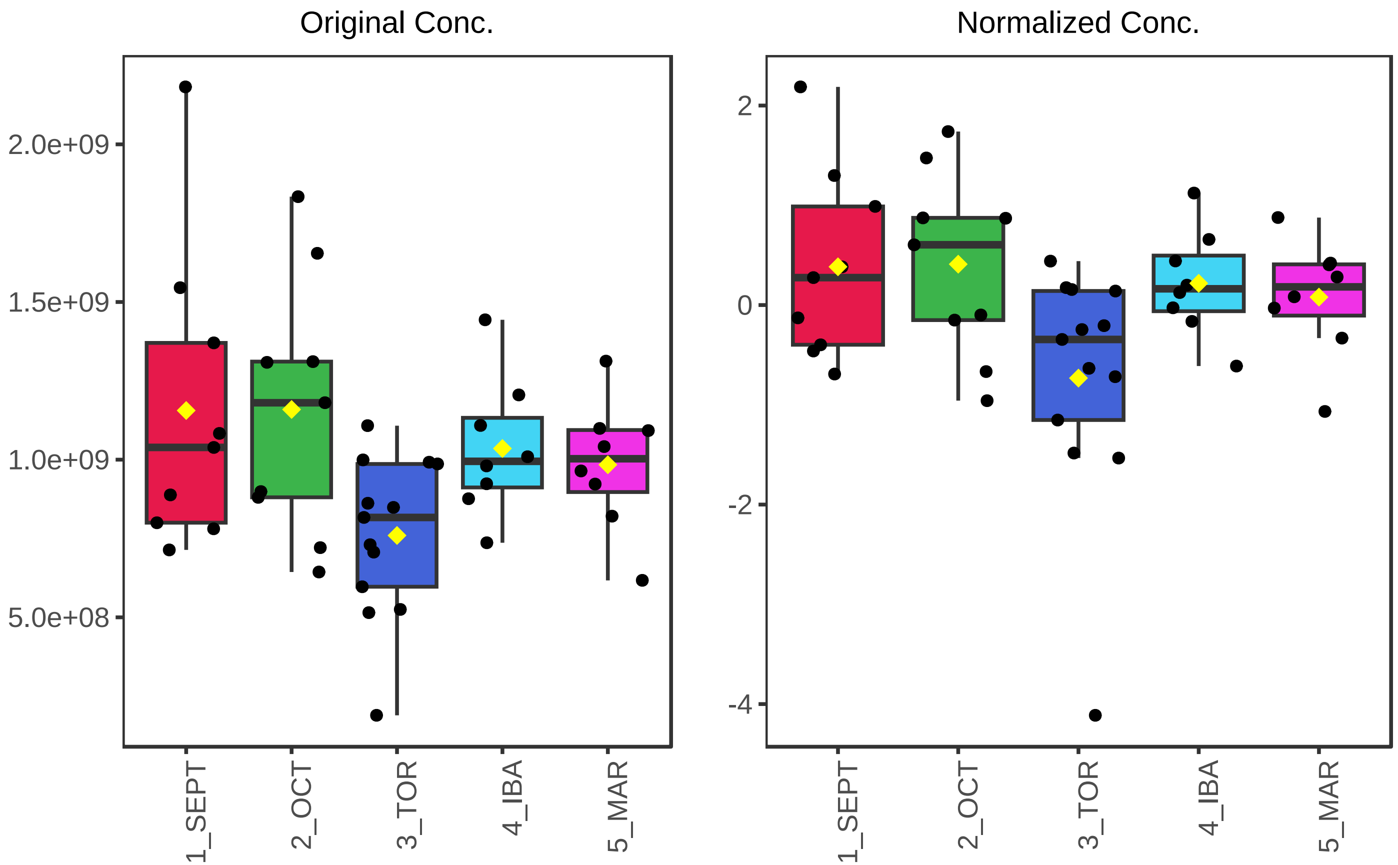

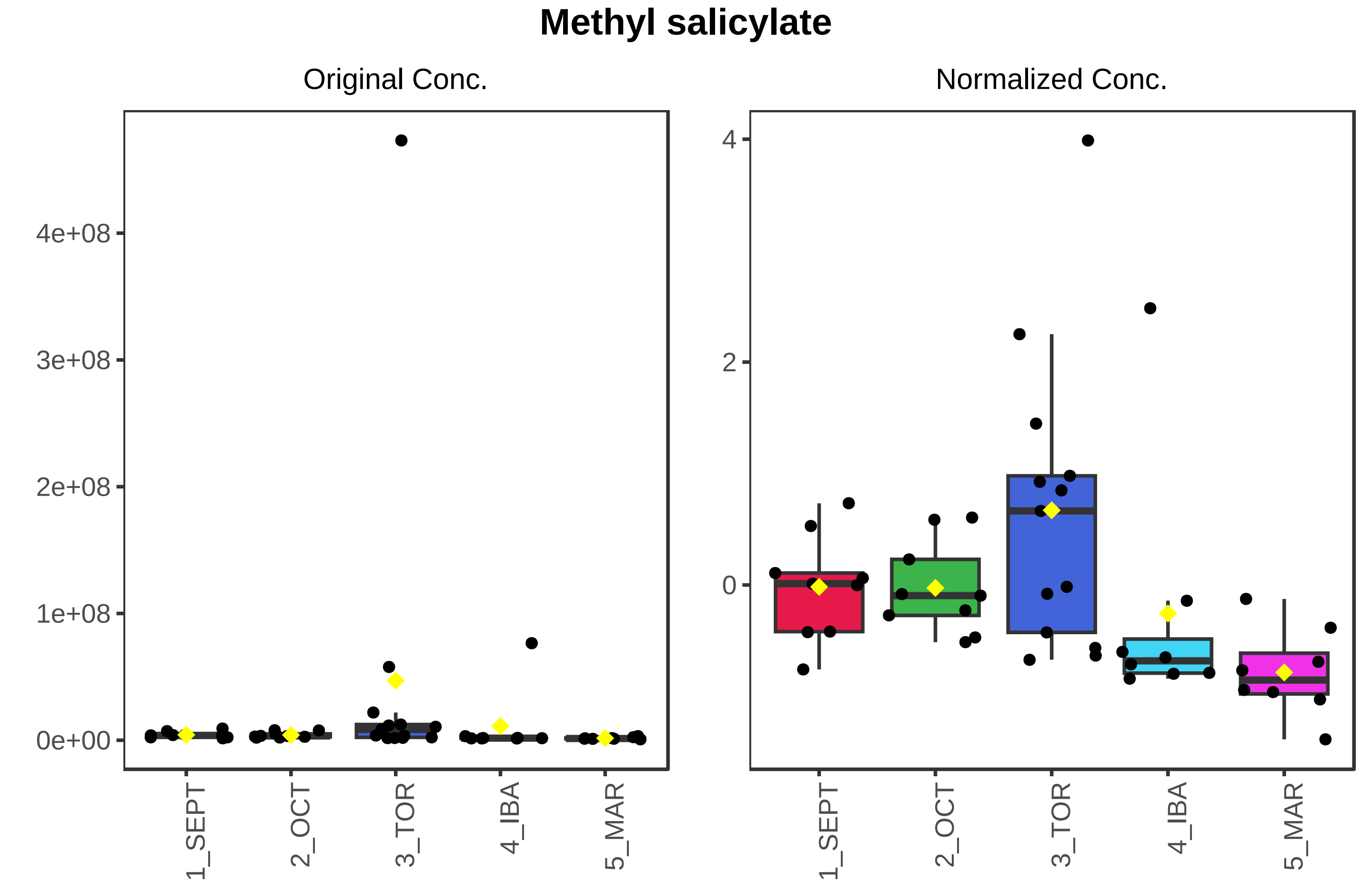

Methyl salicylate
Original Conc.
Normalized Conc.
4e+08
3e+08
2e+08
1e+08
0e+00
4
2
0
1_SEPT
2_OCT
3_TOR
4_IBA
5_MAR

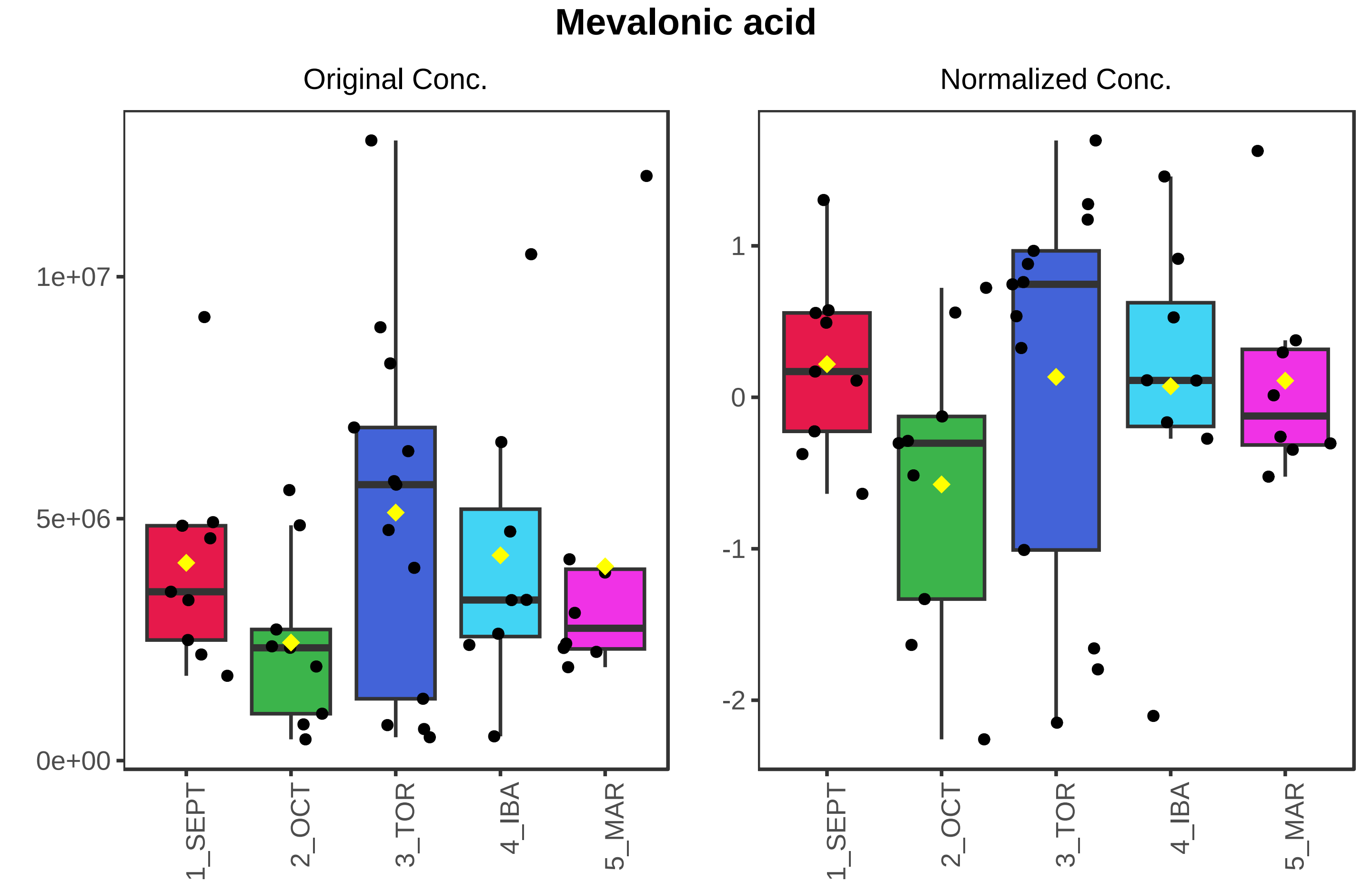

Mevalonic acid
Original Conc.
Normalized Conc.
1e+07
5e+06
0e+00
1
0
-1
-2
1_SEPT
2_OCT
3_TOR
4_IBA
5_MAR

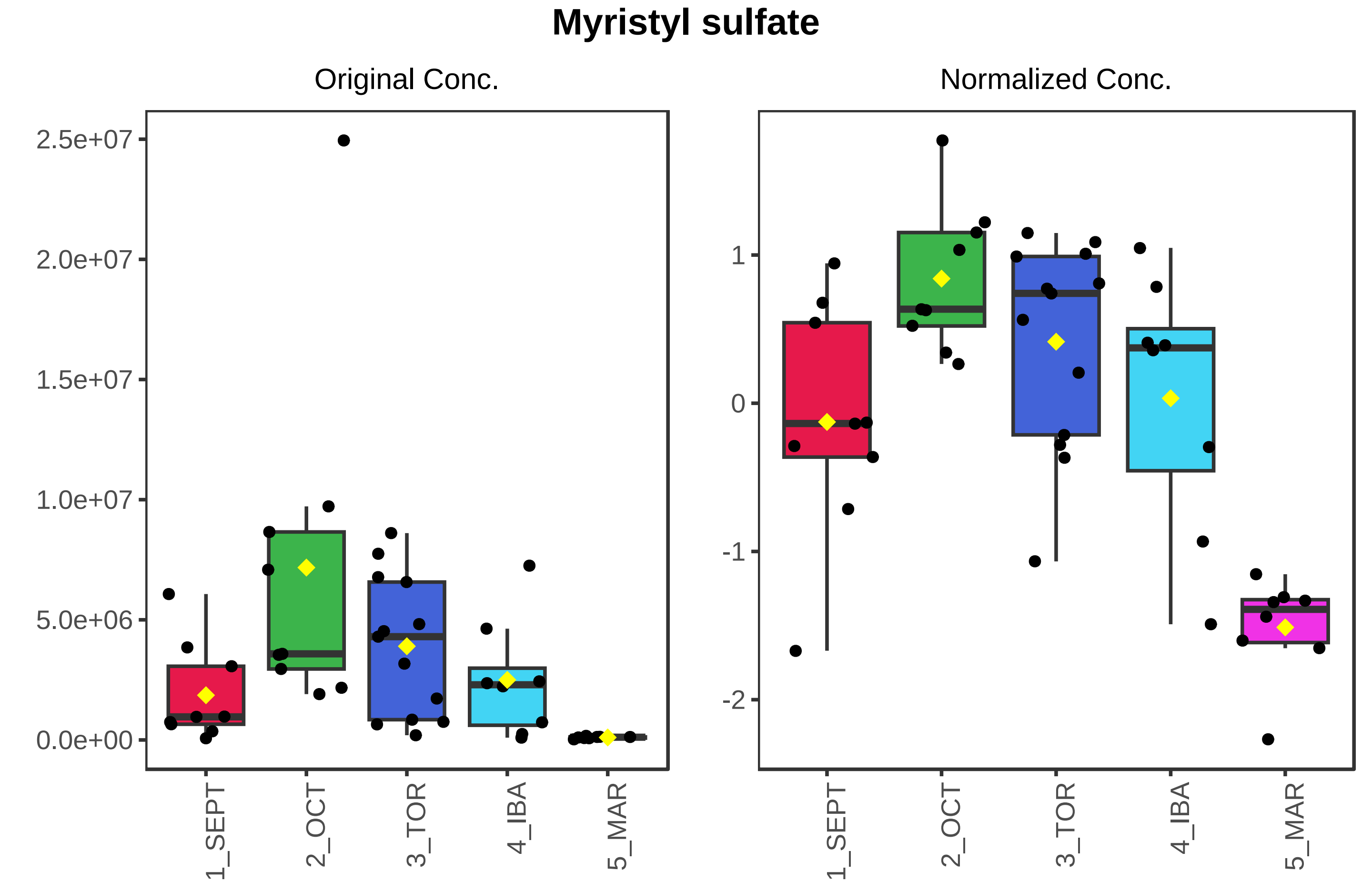
Myristyl sulfate
Original Conc.
Normalized Conc.
2.5e+07
2.0e+07
1.5e+07
1.0e+07
5.0e+06
0.0e+00
1
0
-1
-2
1_SEPT
2_OCT
3_TOR
4_IBA
5_MAR

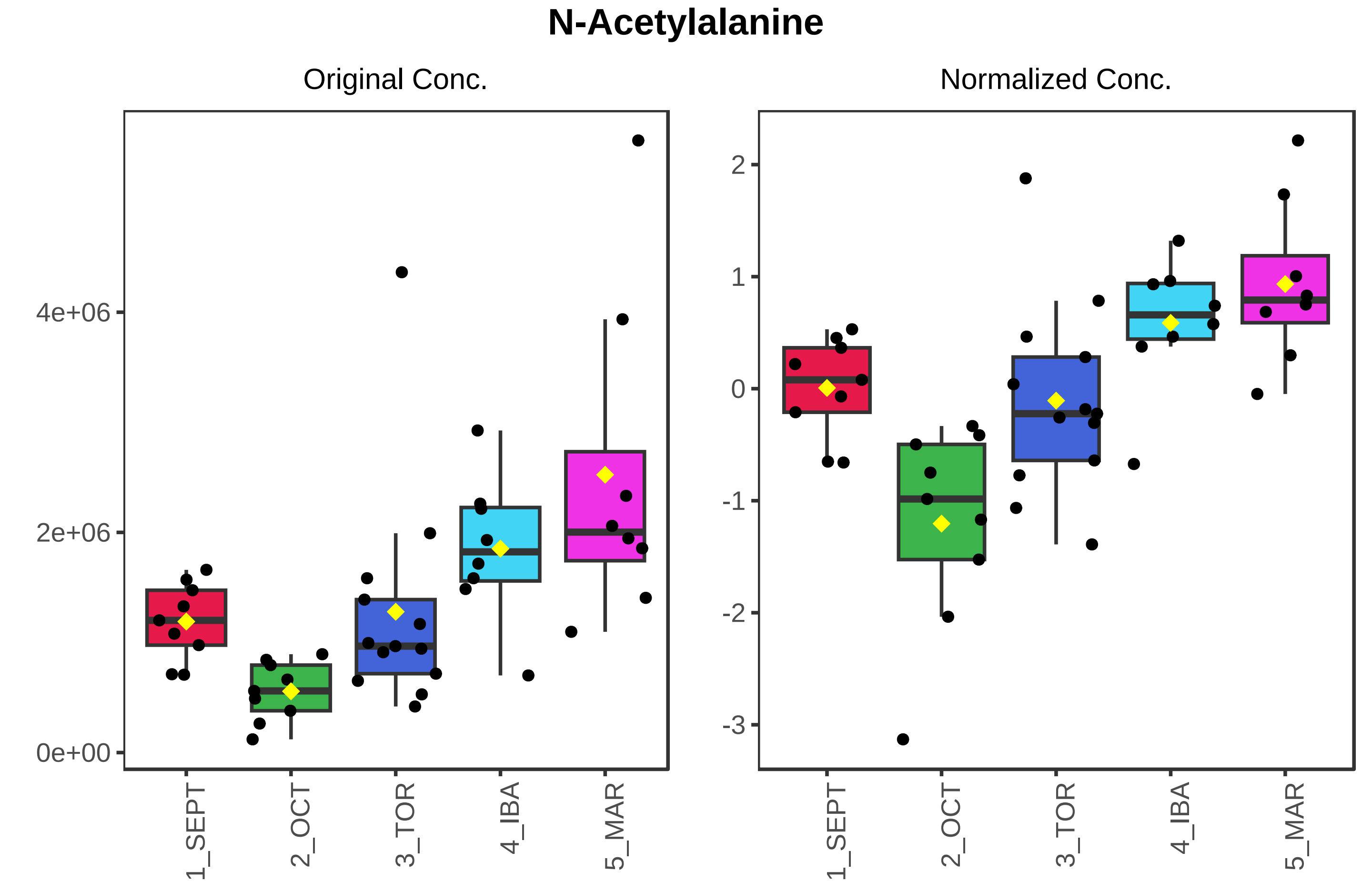

N-Acetylalanine
Original Conc.
Normalized Conc.
4e+06
2e+06
0e+00
2
1
0
-1
-2
-3
1_SEPT
2_OCT
3_TOR
4_IBA
5_MAR

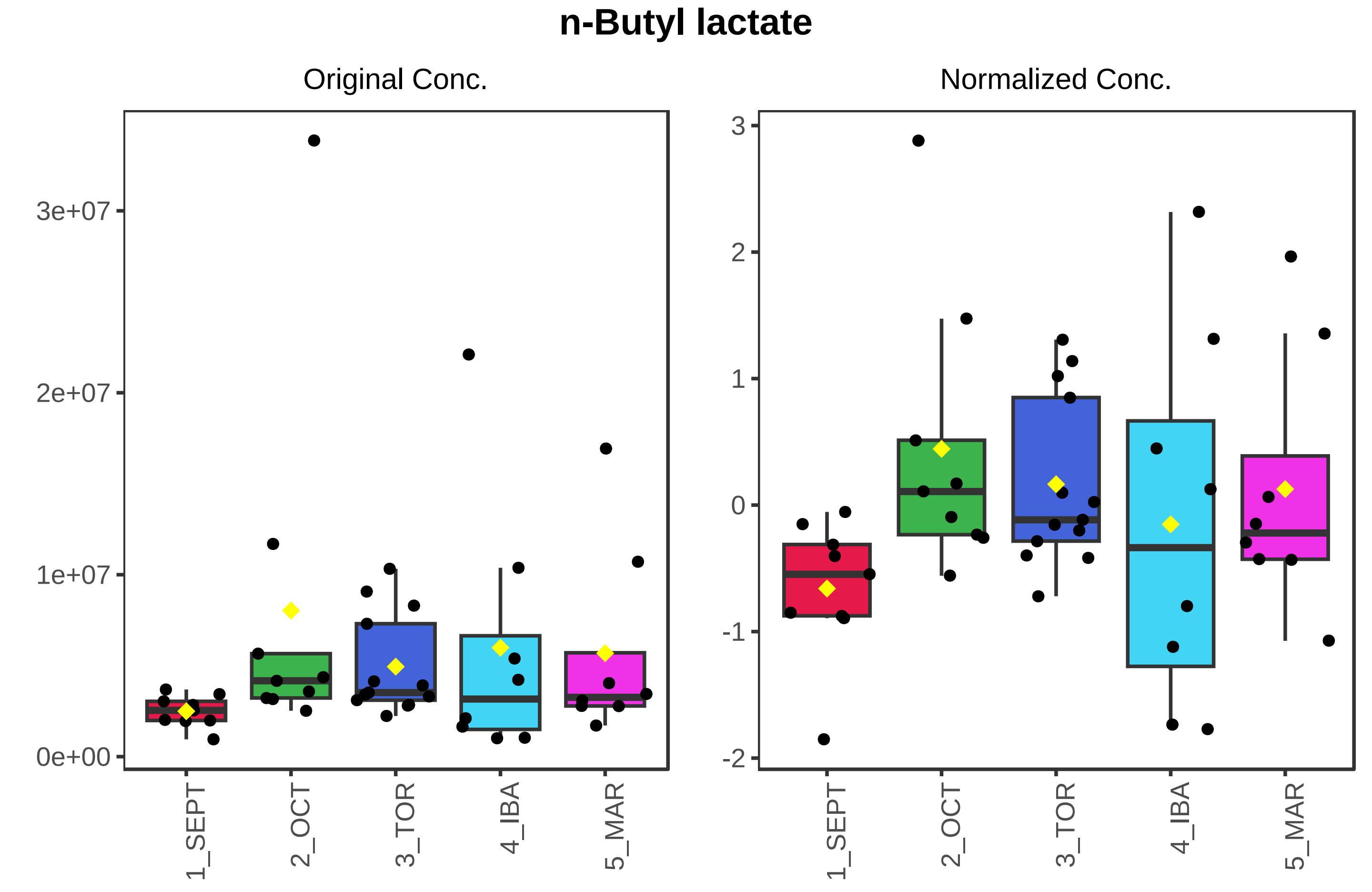

n-Butyl lactate
Original Conc.
Normalized Conc.
3e+07
2e+07
1e+07
0e+00
3
2
1
0
-1
-2
1_SEPT
2_OCT
3_TOR
4_IBA
5_MAR

N-Ethylglycine

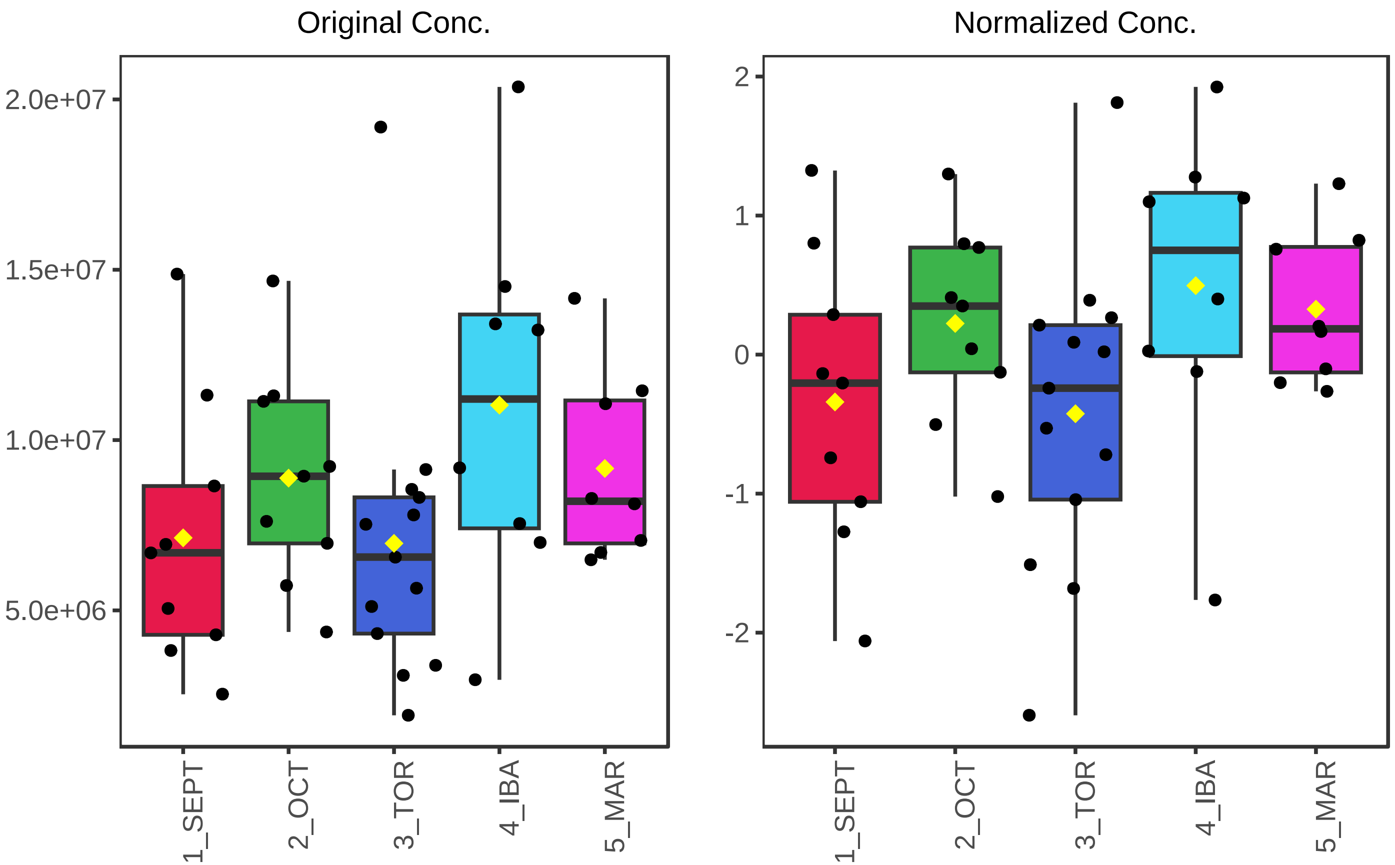

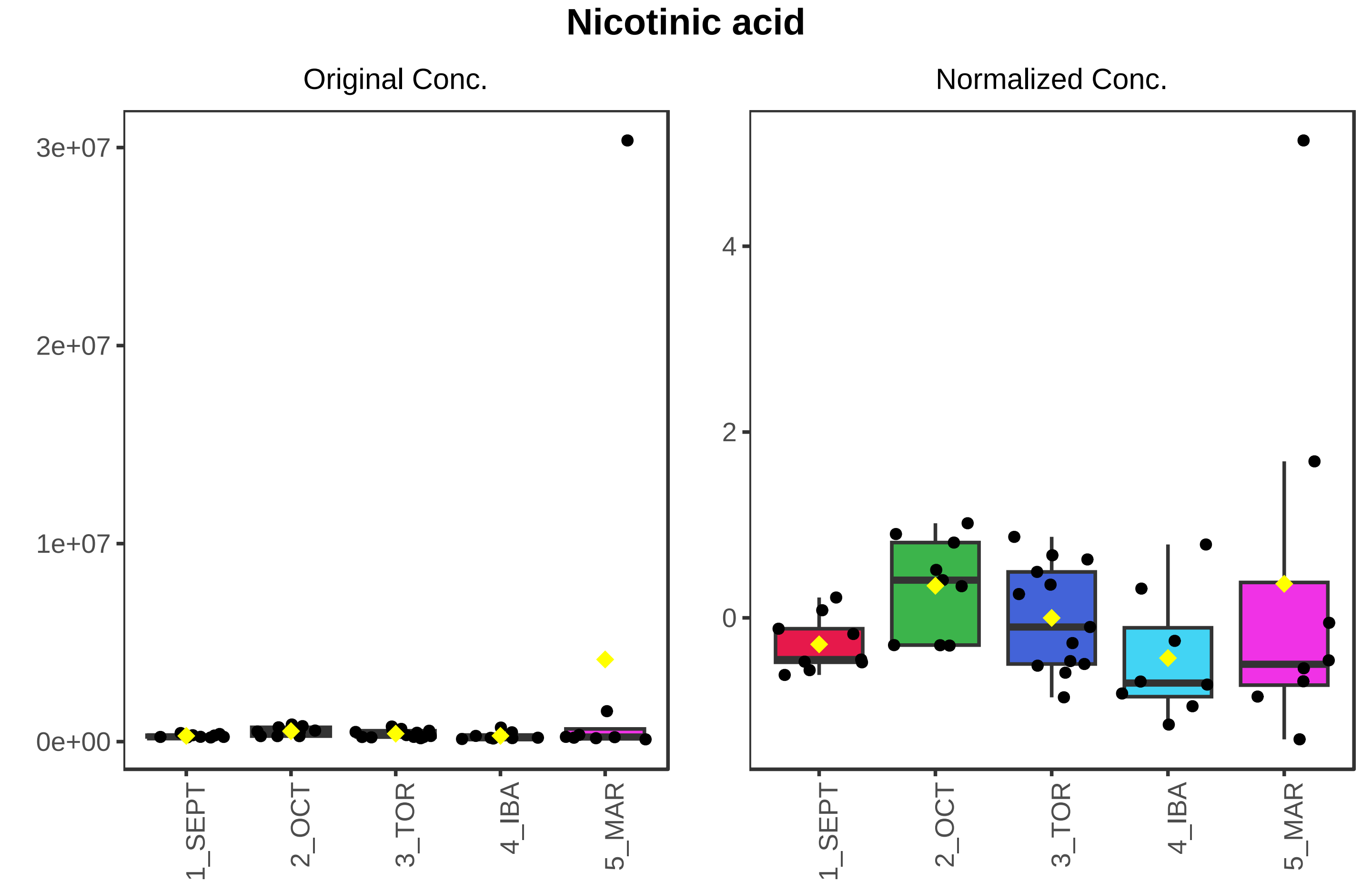
Nicotinic acid
Original Conc.
Normalized Conc.
3e+07
2e+07
1e+07
0e+00
4
2
0
1_SEPT
2_OCT
3_TOR
4_IBA
5_MAR

N-methyl-2-oxoglutaramic acid

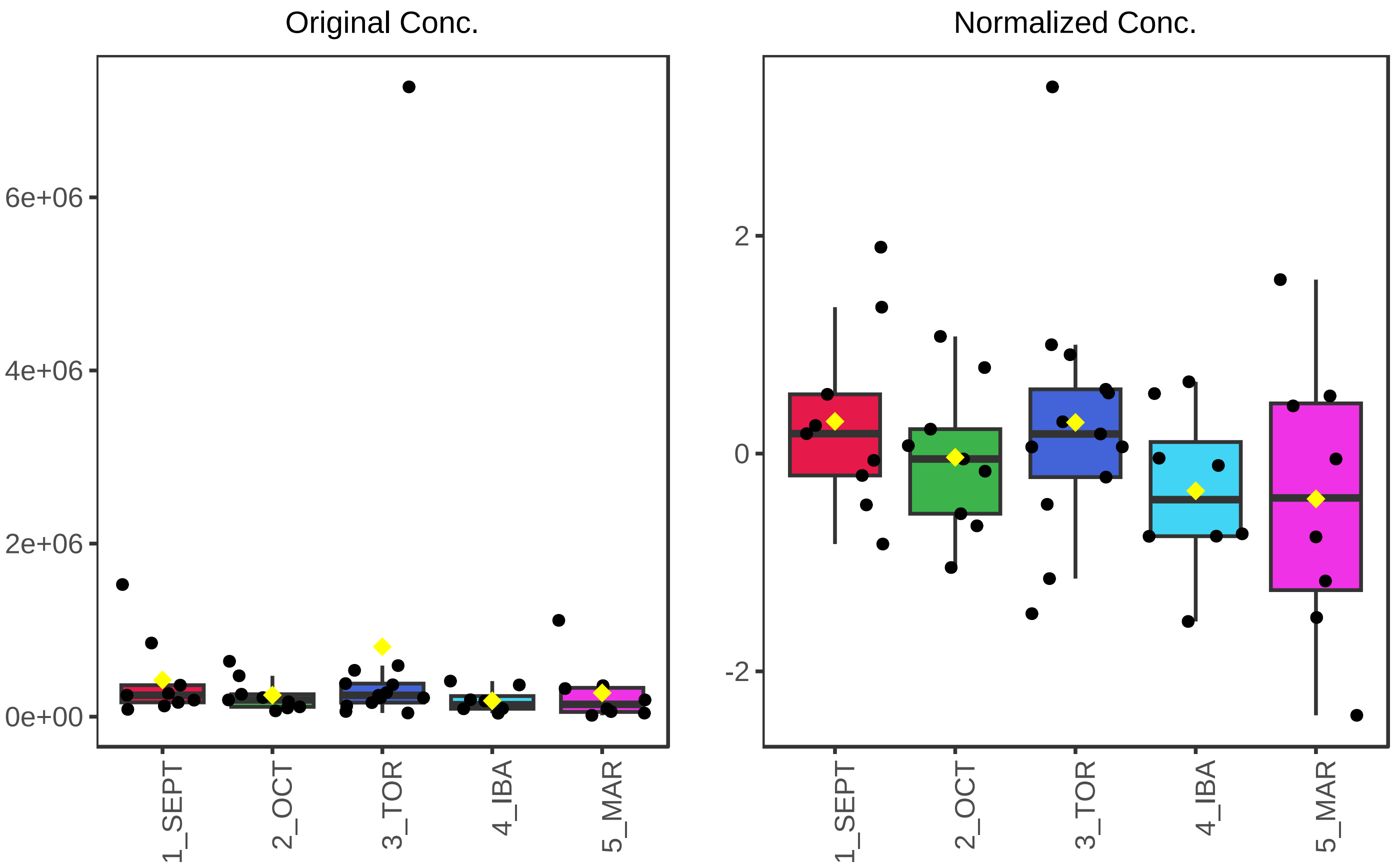

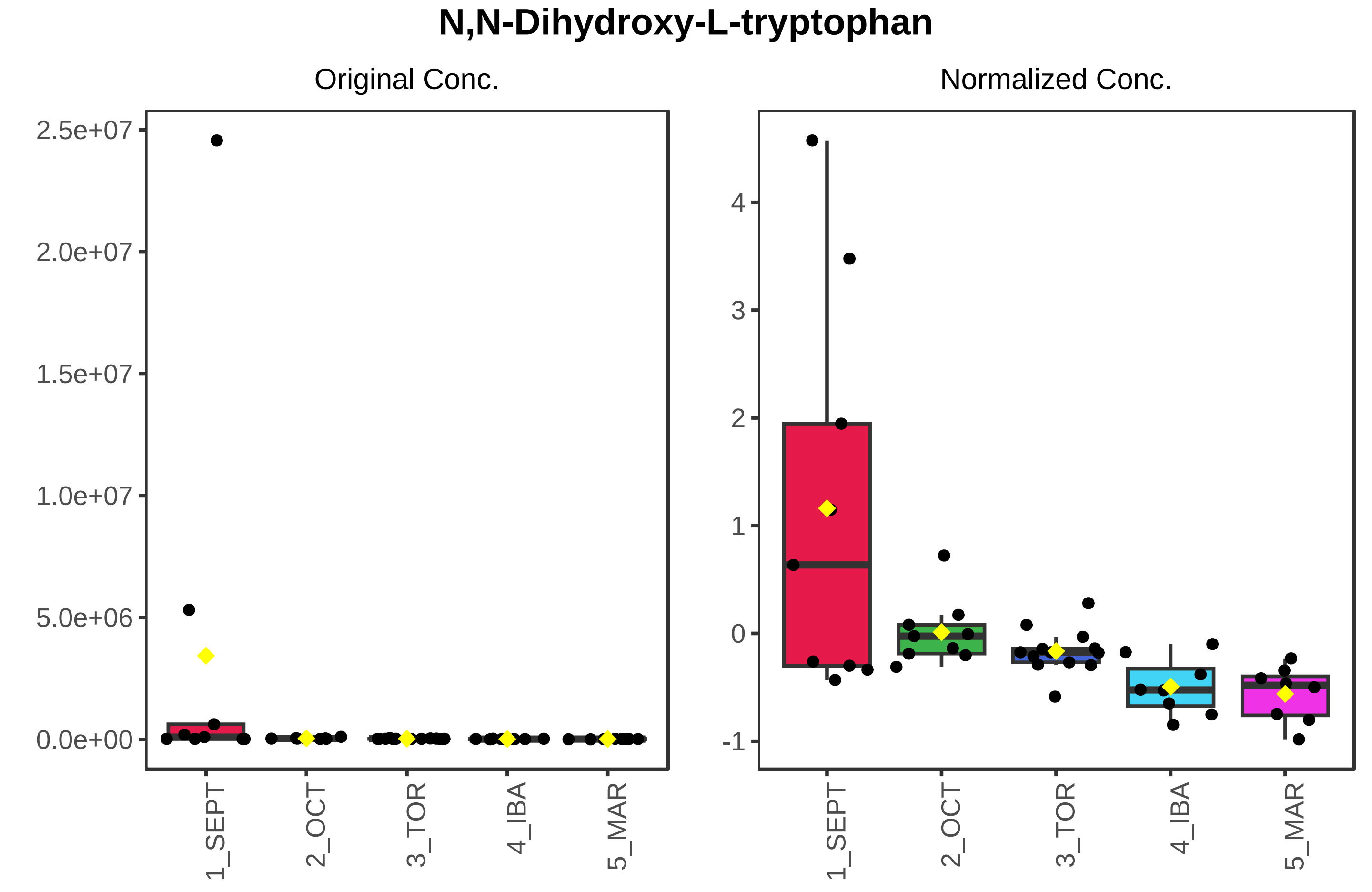
N,N-Dihydroxy-L-tryptophan
Original Conc.
Normalized Conc.
2.5e+07
2.0e+07
1.5e+07
1.0e+07
5.0e+06
0.0e+00
4
3
2
1
0
-1
1_SEPT
2_OCT
3_TOR
4_IBA
5_MAR

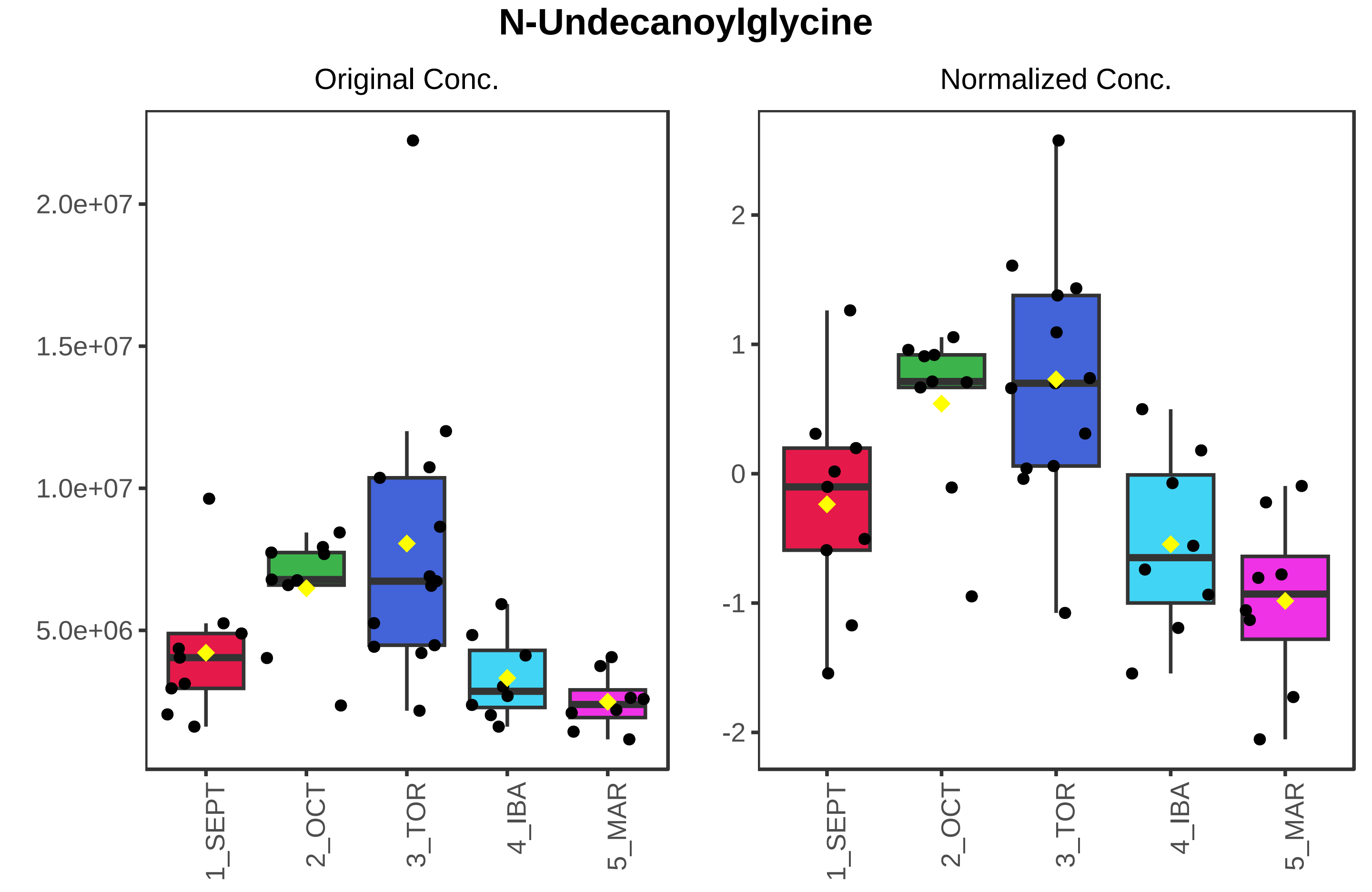

N-Undecanoylglycine
Original Conc.
Normalized Conc.
2.0e+07
1.5e+07
1.0e+07
5.0e+06
2
1
0
-1
-2
1_SEPT
2_OCT
3_TOR
4_IBA
5_MAR

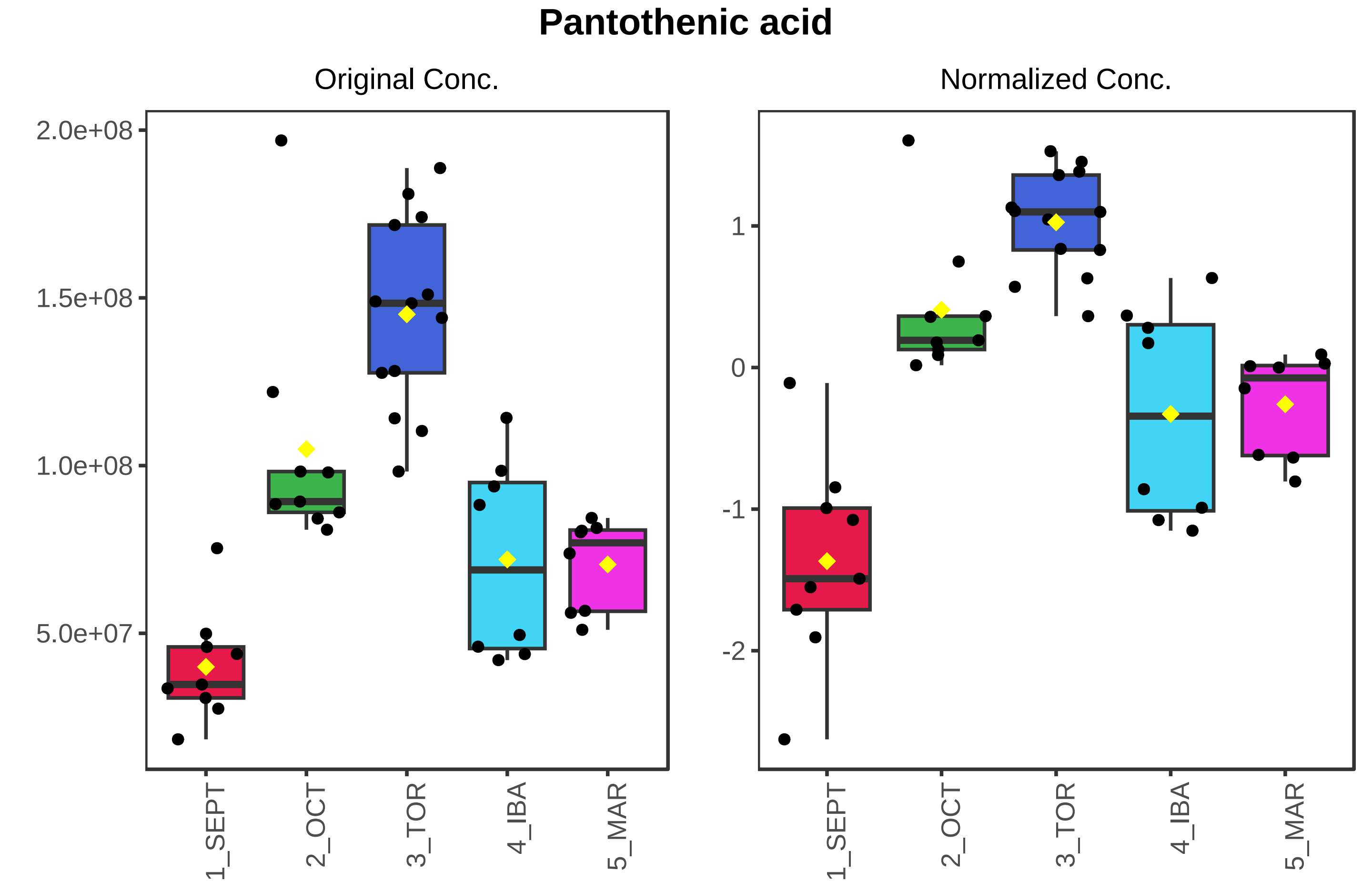

Pantothenic acid
Original Conc.
Normalized Conc.
2.0e+08
1.5e+08
1.0e+08
5.0e+07
1
0
-1
-2
1_SEPT
2_OCT
3_TOR
4_IBA
5_MAR

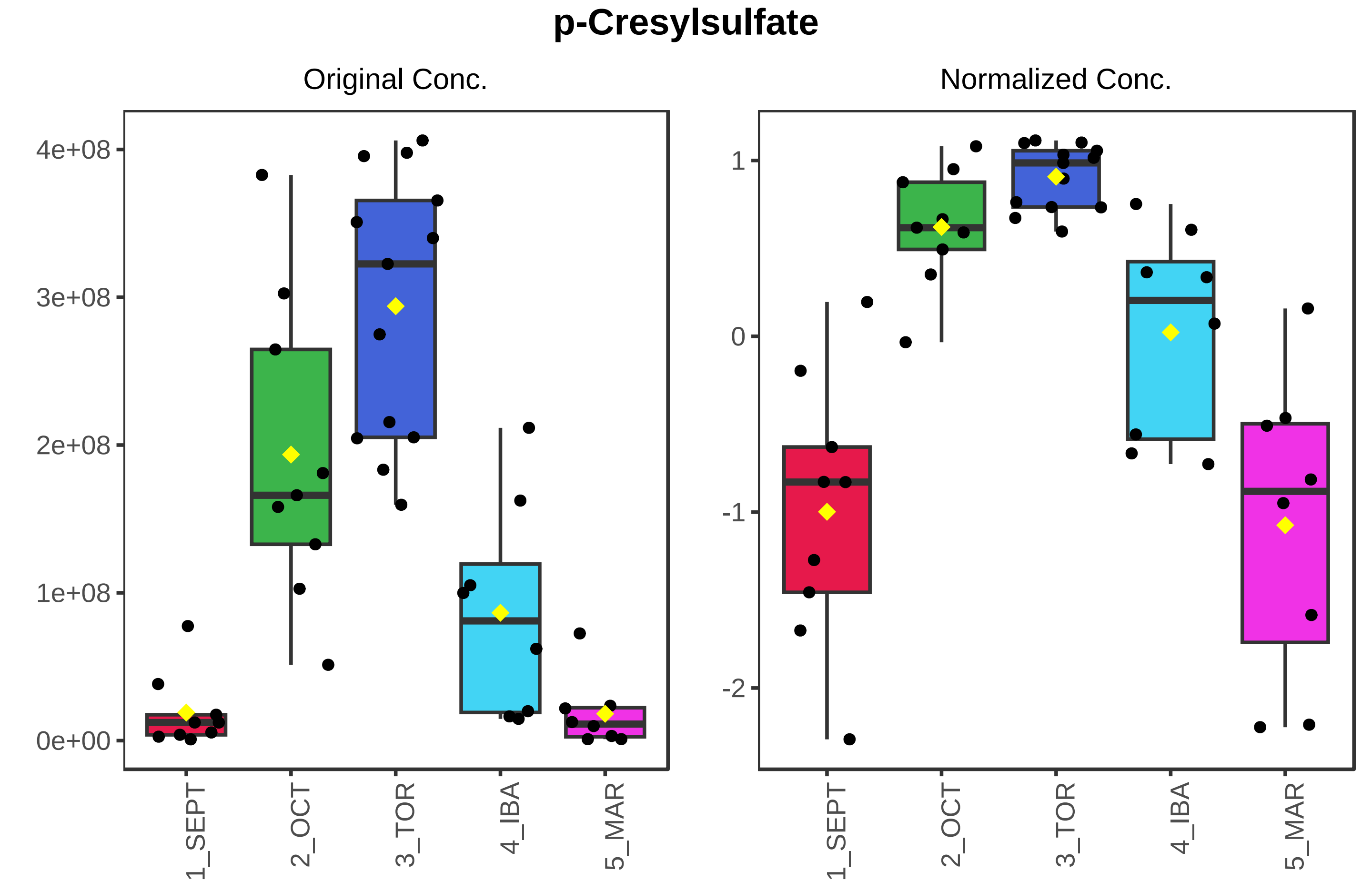
p-Cresylsulfate
Original Conc.
Normalized Conc.
4e+08
3e+08
2e+08
1e+08
0e+00
1
0
-1
-2
1_SEPT
2_OCT
3_TOR
4_IBA
5_MAR

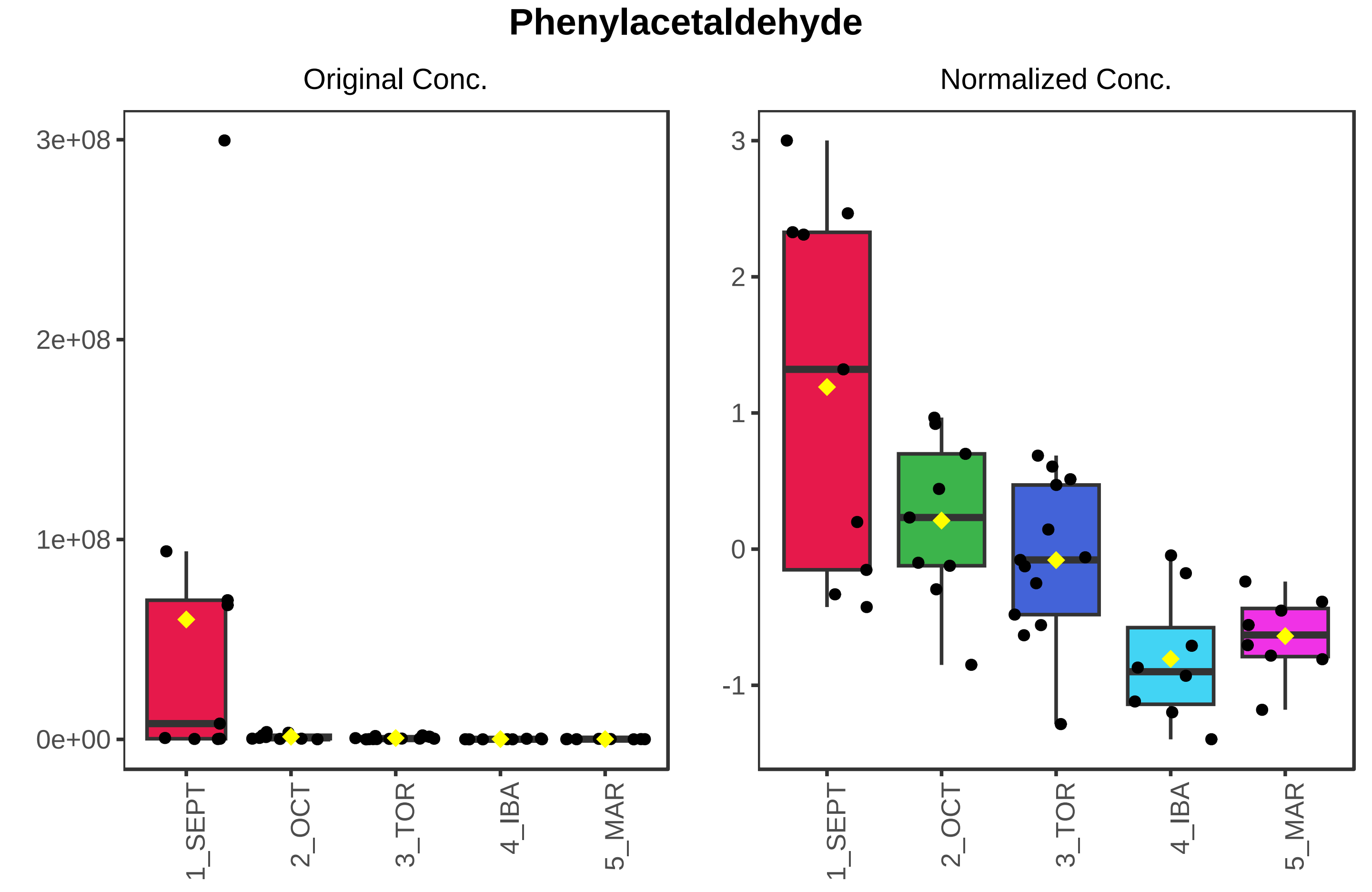
Phenylacetaldehyde
Original Conc.
Normalized Conc.
3e+08
2e+08
1e+08
0e+00
3
2
1
0
-1
1_SEPT
2_OCT
3_TOR
4_IBA
5_MAR

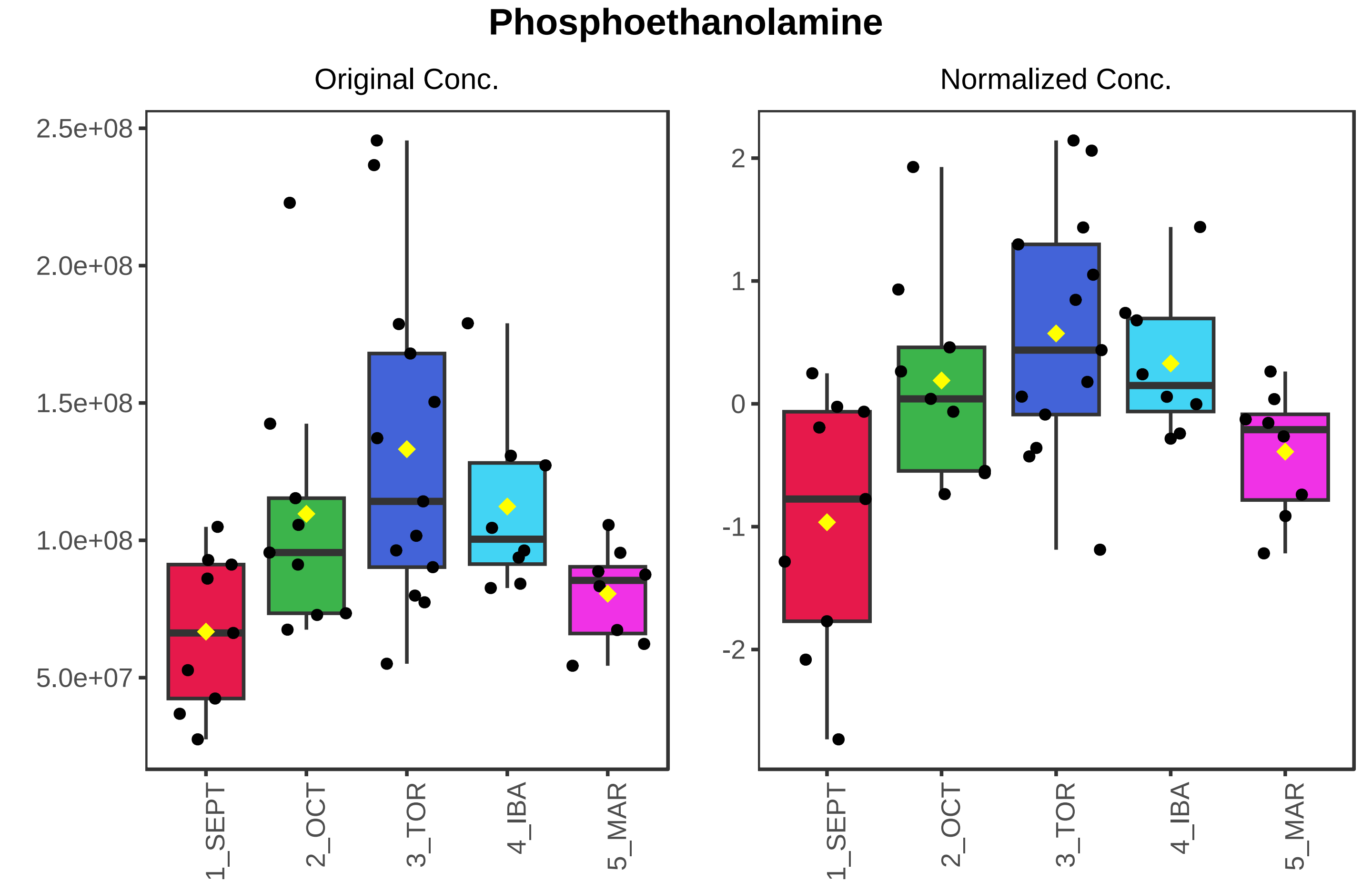
Phosphoethanolamine
Original Conc.
Normalized Conc.
2.5e+08
2.0e+08
1.5e+08
1.0e+08
5.0e+07
2
1
0
-1
-2
1_SEPT
2_OCT
3_TOR
4_IBA
5_MAR

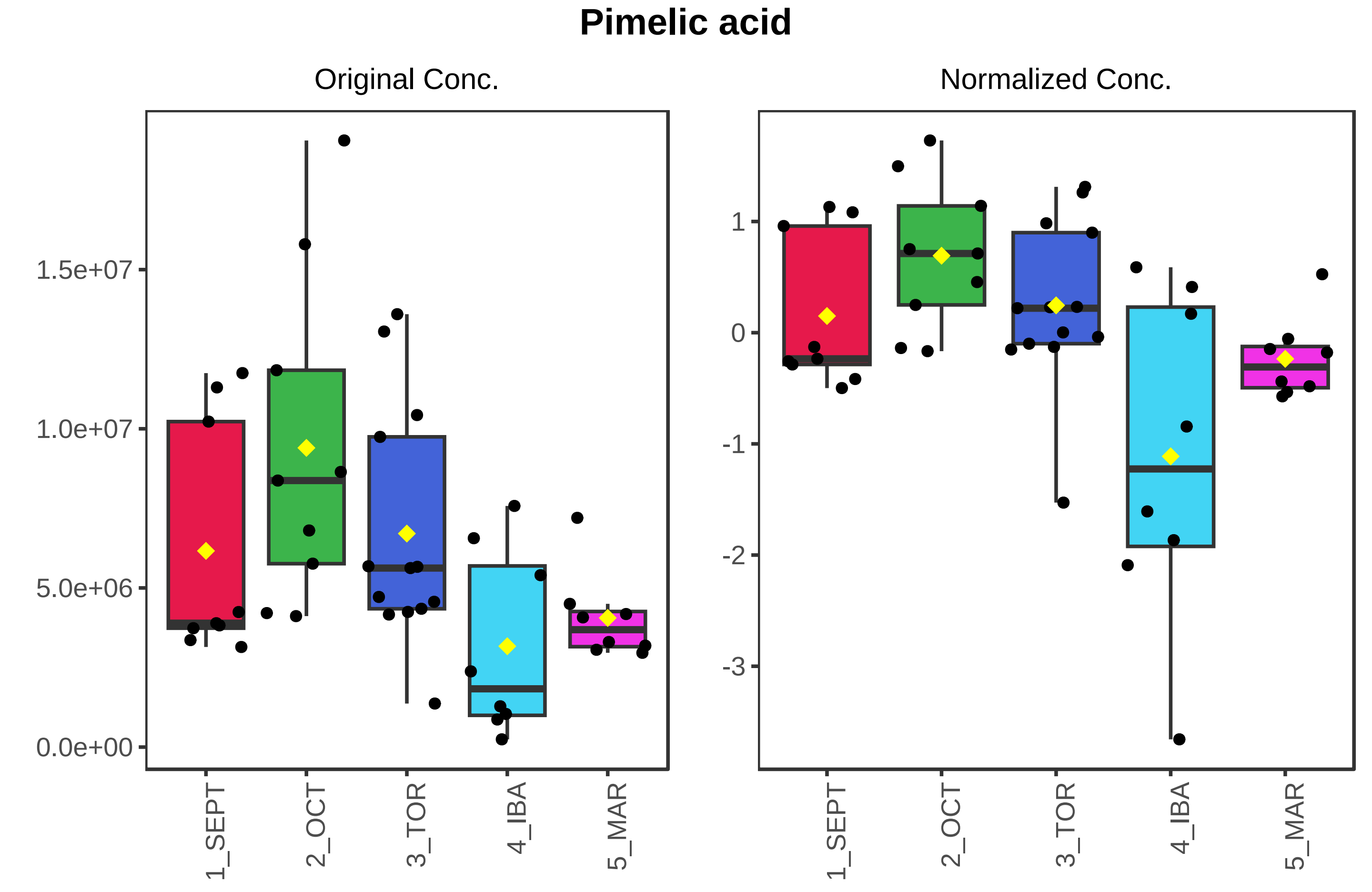

Pimelic acid
Original Conc.
Normalized Conc.
1.5e+07
1.0e+07
5.0e+06
0.0e+00
1
0
-1
-2
-3
1_SEPT
2_OCT
3_TOR
4_IBA
5_MAR

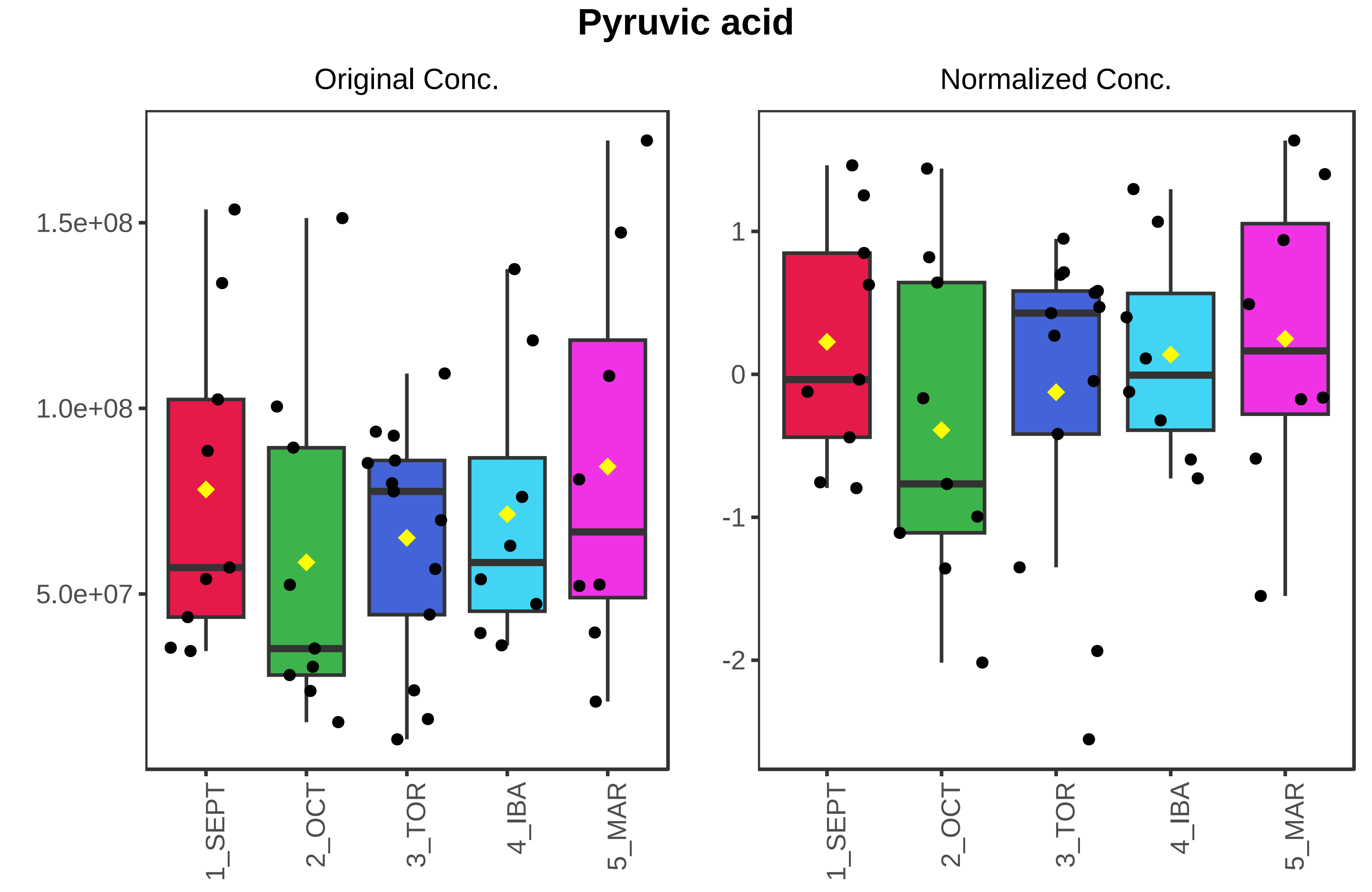

Pyruvic acid
Original Conc.
Normalized Conc.
1.5e+08
1.0e+08
5.0e+07
1
0
-1
-2
1_SEPT
2_OCT
3_TOR
4_IBA
5_MAR

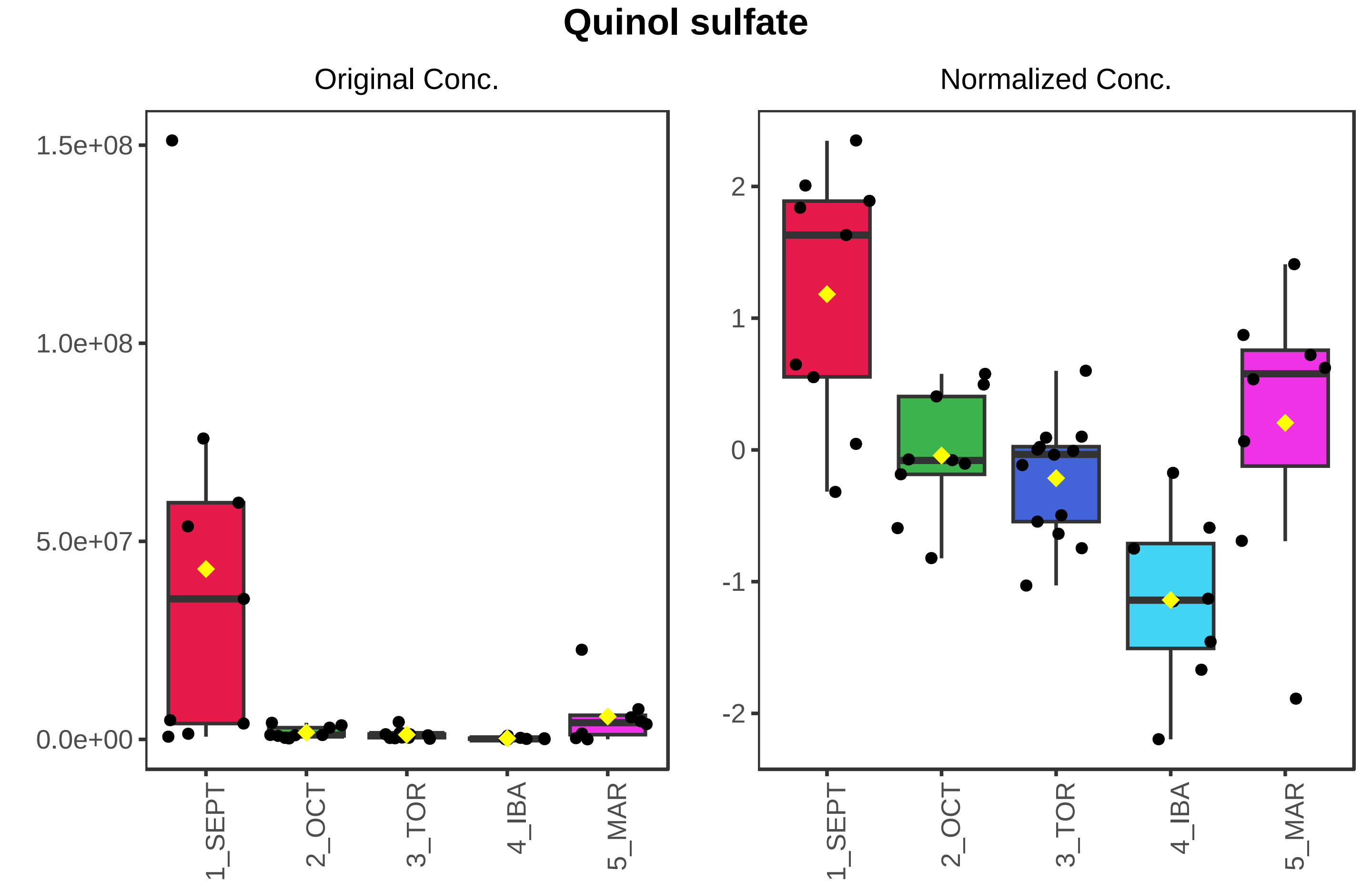

Quinol sulfate
Original Conc.
Normalized Conc.
1.5e+08
1.0e+08
5.0e+07
0.0e+00
2
1
0
-1
-2
1_SEPT
2_OCT
3_TOR
4_IBA
5_MAR

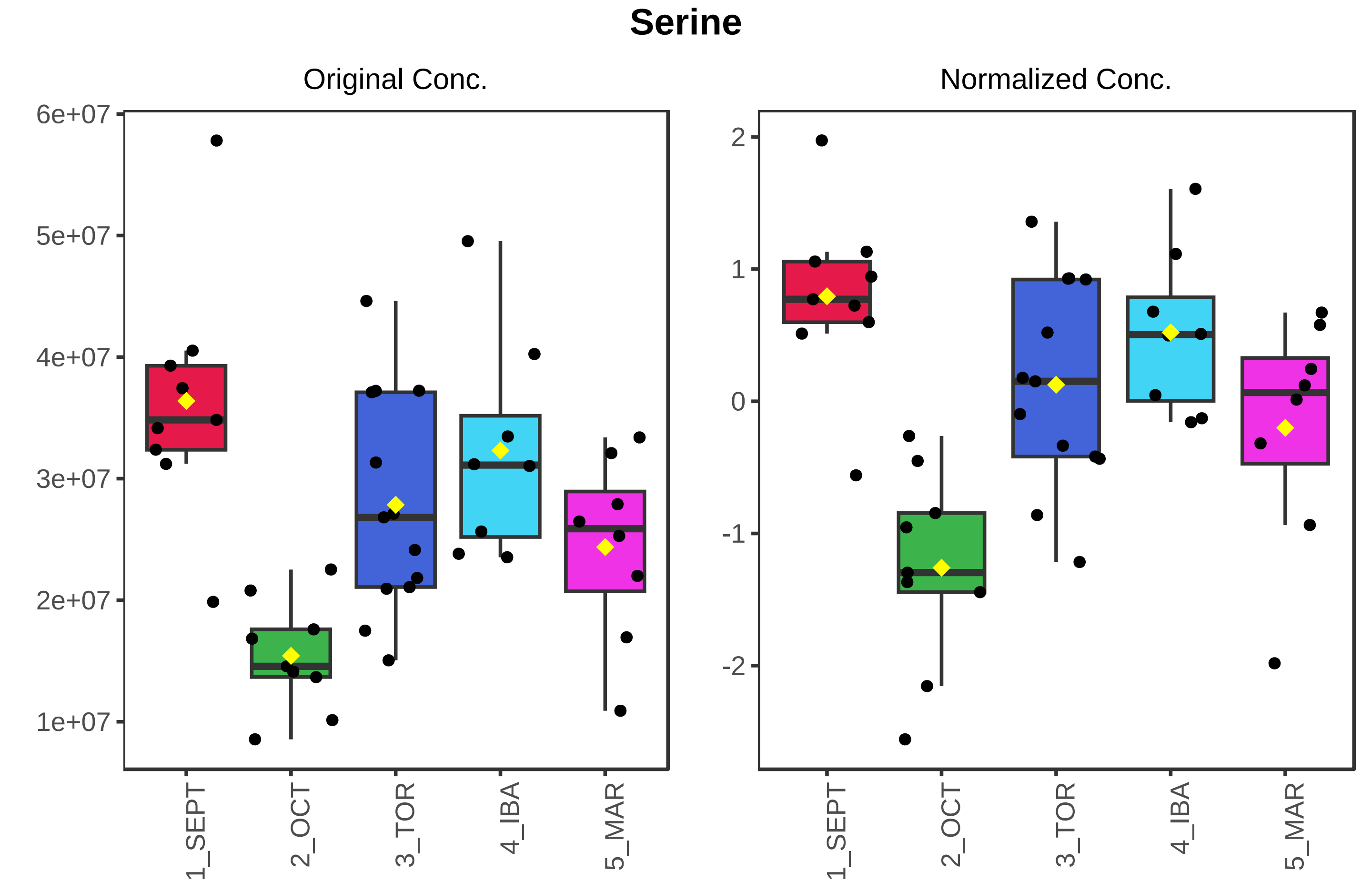

Serine
Original Conc.
Normalized Conc.
6e+07
5e+07
4e+07
3e+07
2e+07
1e+07
2
1
0
-1
-2
1_SEPT
2_OCT
3_TOR
4_IBA
5_MAR

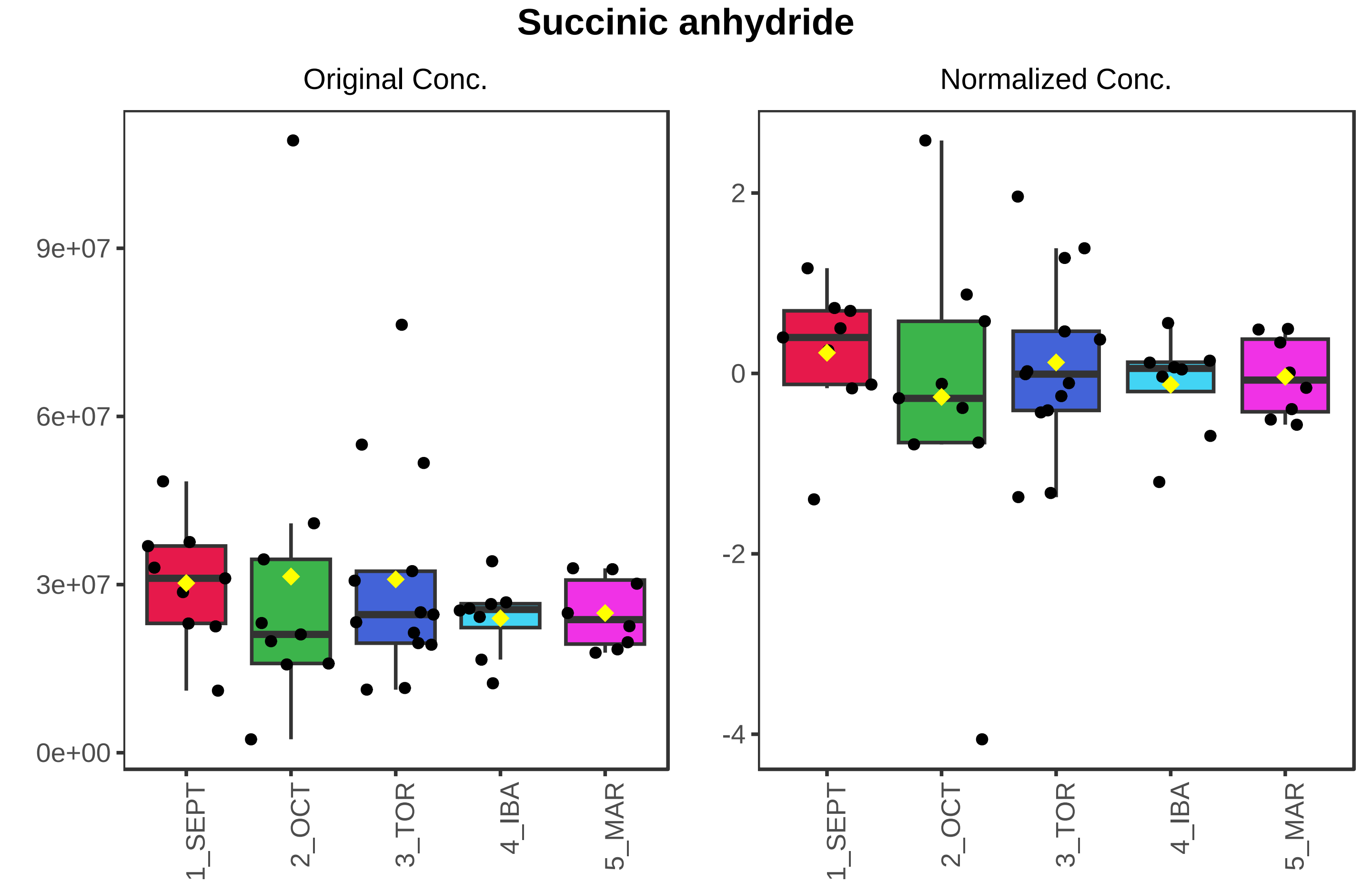

Succinic anhydride
Original Conc.
Normalized Conc.
9e+07
6e+07
3e+07
0e+00
2
0
-2
-4
1_SEPT
2_OCT
3_TOR
4_IBA
5_MAR

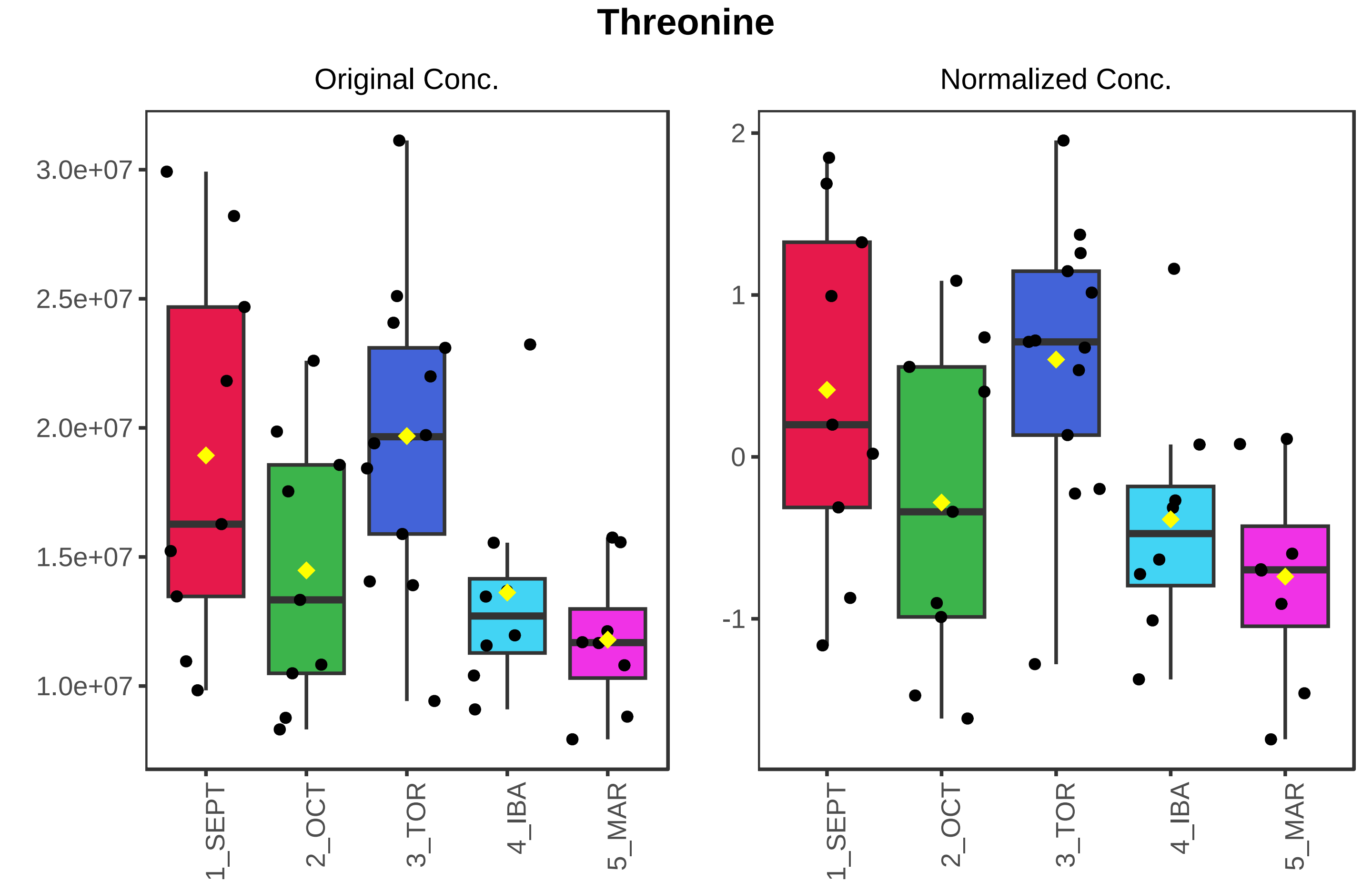
Threonine
Original Conc.
Normalized Conc.
3.0e+07
2.5e+07
2.0e+07
1.5e+07
1.0e+07
2
1
0
-1
1_SEPT
2_OCT
3_TOR
4_IBA
5_MAR

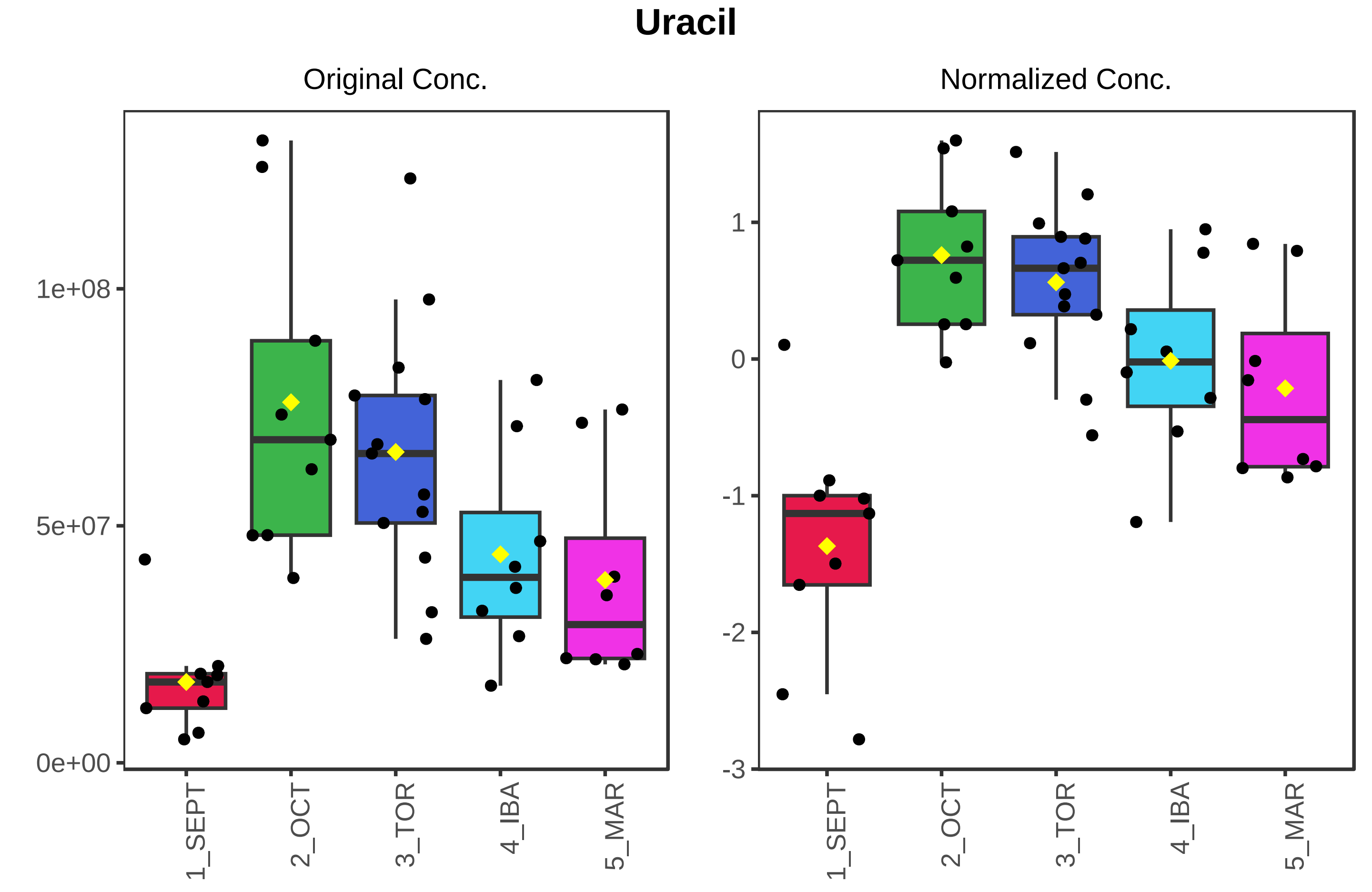

Uracil
Original Conc.
Normalized Conc.
1e+08
5e+07
0e+00
1
0
-1
-2
-3
1_SEPT
2_OCT
3_TOR
4_IBA
5_MAR

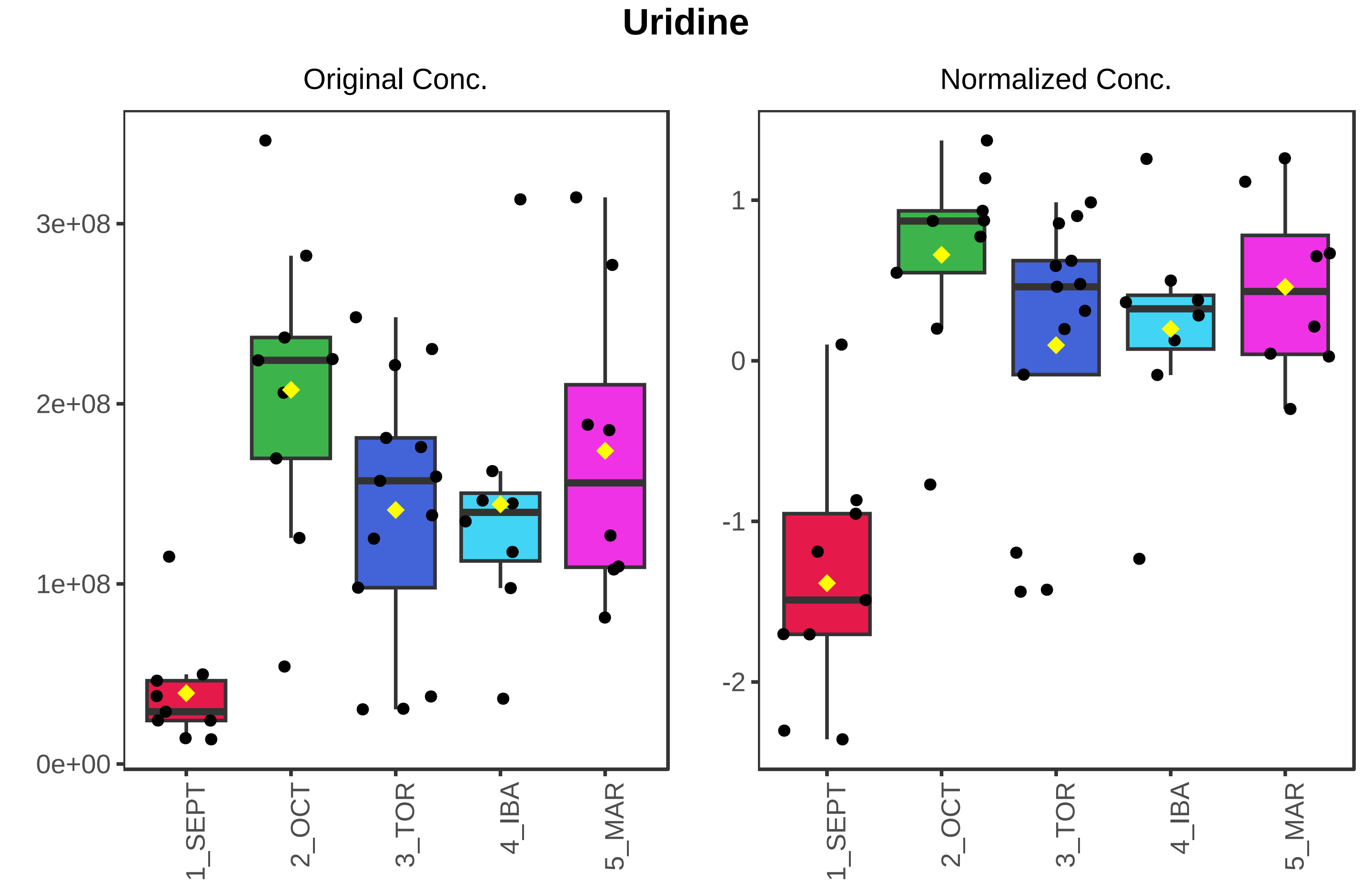

Uridine
Original Conc.
Normalized Conc.
3e+08
2e+08
1e+08
0e+00
1
0
-1
-2
1_SEPT
2_OCT
3_TOR
4_IBA
5_MAR

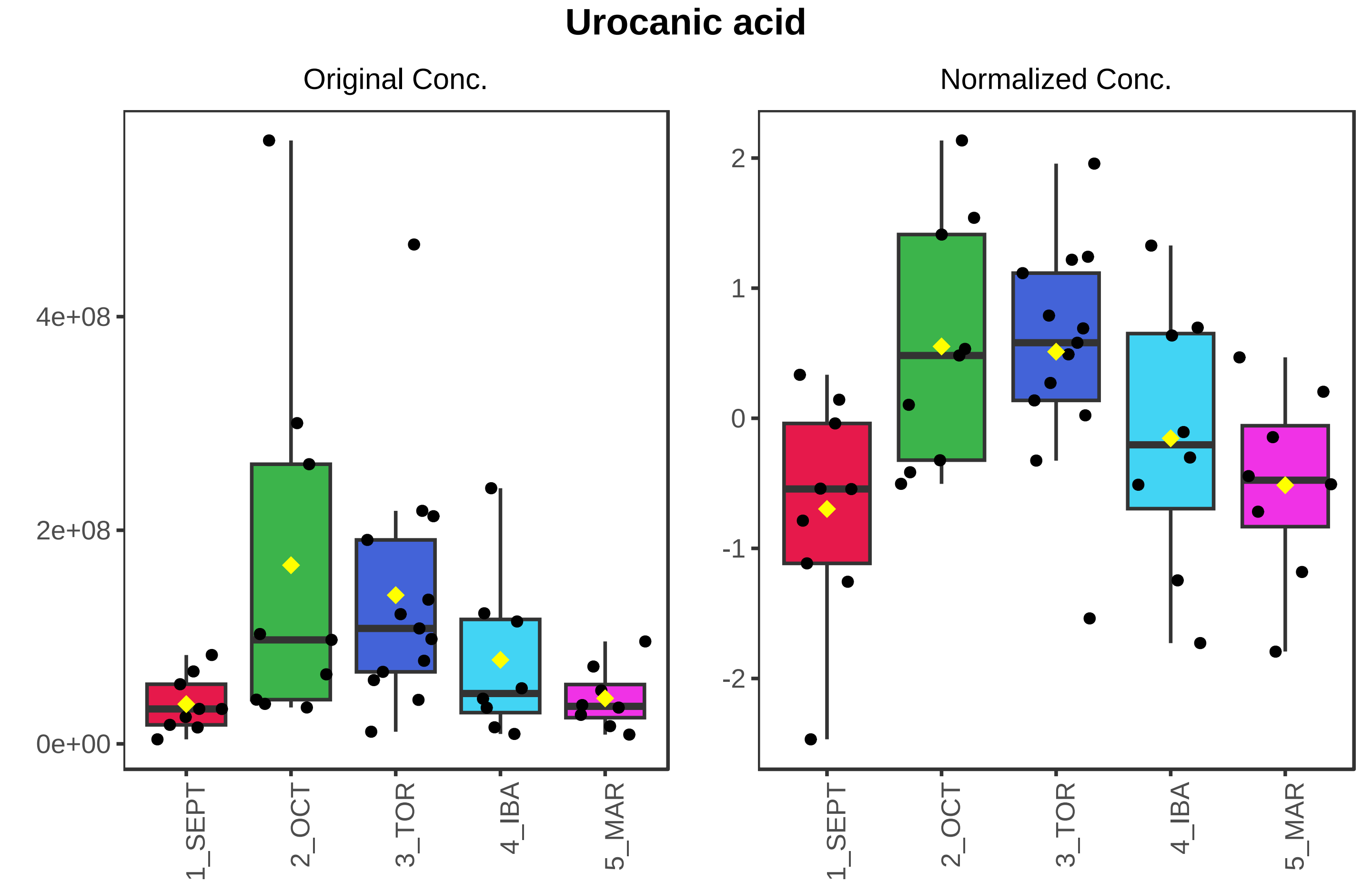
Urocanic acid
Original Conc.
Normalized Conc.
4e+08
2e+08
0e+00
2
1
0
-1
-2
1_SEPT
2_OCT
3_TOR
4_IBA
5_MAR
1_SEPT
2_OCT
3_TOR
4_IBA
5_MAR

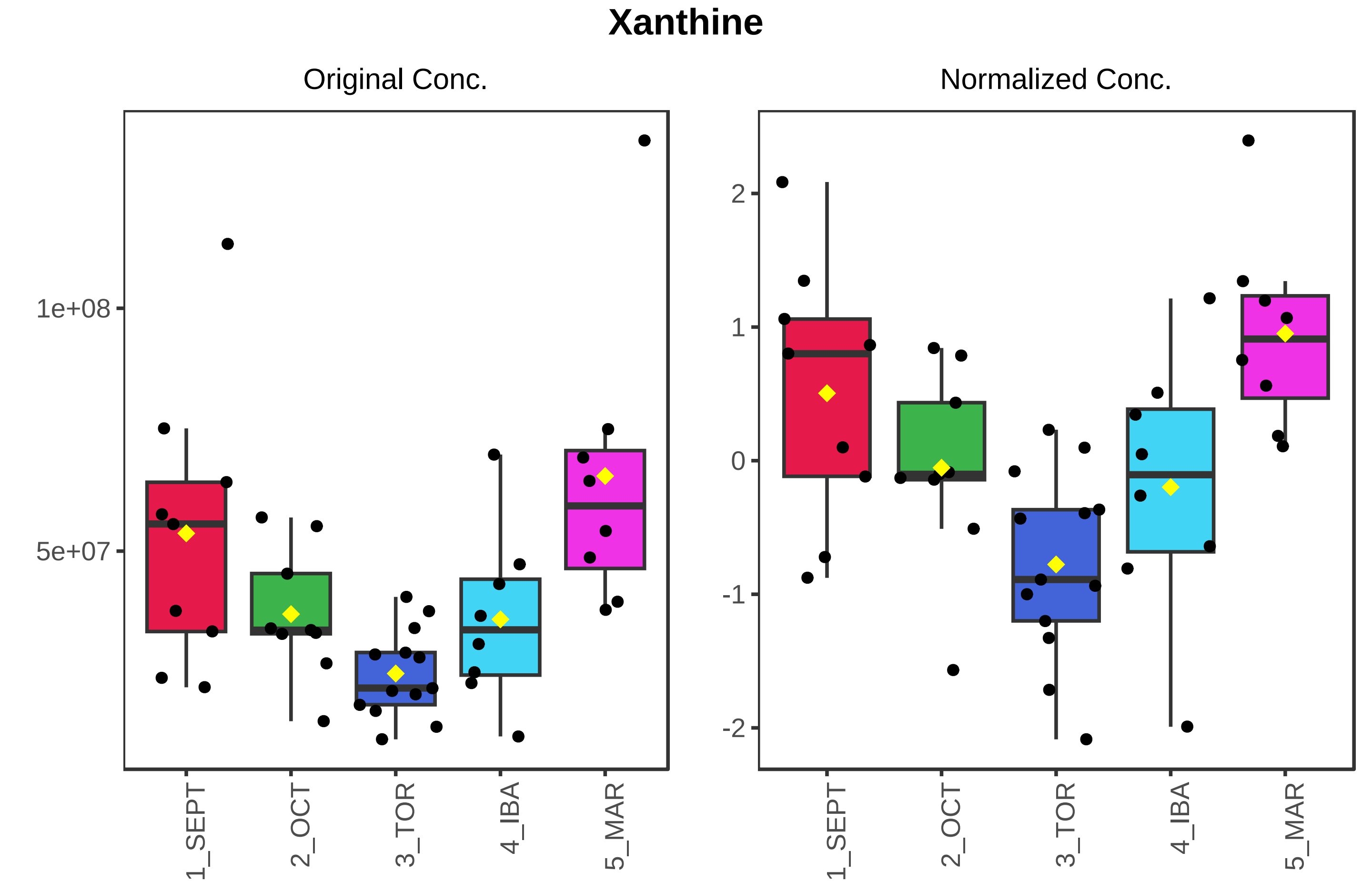
Xanthine
Original Conc.
Normalized Conc.
1e+08
5e+07
2
1
0
-1
-2
1_SEPT
2_OCT
3_TOR
4_IBA
5_MAR
